# Genetic Heterogeneity in Autism Spectrum Disorder: Diagnostic Yield, Recurrent Genes, and Rare Variant-Phenotype Associations from Whole-Exome Sequencing

**DOI:** 10.3390/ijms27156901

**Published:** 2026-08-01

**Authors:** Zainab Gaouzi, Giulia Spoto, Francesca Polito, Rihab Festali, Irene Gasparo, Laura Licitri, Anna Maria Mirabello, Silvia Romano, Vincenzo Macaione, Nouzha Dini, Elmostafa El Fahime, Saber Boutayeb, Yamna Kriouile, Idrissa Diawara, Gabriella di Rosa, Mhammed Aguennouz

**Affiliations:** 1 Mohammed VI University of Sciences and Health (UM6SS), Casablanca 82403, Morocco; zgaouzi@um6ss.ma (Z.G.);; 2 Mohammed VI Center for Research and Innovation (CM6RI), Rabat 10100, Morocco; 3 Department of Clinical and Experimental Medicine, University of Messina, 98122 Messina, Italy; 4 Unit of Child Neurology and Psychiatry, Department BIOMORF, University Hospital of Messina, 98125 Messina, Italy; giulia.spoto27@gmail.com (G.S.);; 5 Sheikh Khalifa International University Hospital, Faculty of Medicine, Mohammed VI University of Health Sciences, Casablanca 82403, Morocco; 6 Faculty of Medicine and Pharmacy, Rabat University of Mohammed V Rabat, Rabat 11000, Morocco

**Keywords:** autism, exome, genetics, variants, phenotype, neurodevelopment

## Abstract

Autism spectrum disorder (ASD) is genetically heterogeneous, involving rare and common variants that disrupt neurodevelopmental pathways. To explore this complexity, we performed whole-exome sequencing in children with ASD. Clinical phenotypes were systematically recorded, and severity was classified according to DSM-5 criteria. Variants were interpreted using ACMG guidelines, with recurrence analysis to identify genes shared across individuals and cohort enrichment testing against gnomAD. To examine genotype-phenotype relationships, we applied SKAT/SKAT-O across 16 phenotypes after covariate adjustment. Among 25 included individuals, pathogenic or likely pathogenic variants were found in 9, yielding a diagnostic yield of 36%. These involved genes linked to neurodevelopmental, epileptic, metabolic, and syndromic disorders. Recurrence analysis identified 586 genes present in at least two individuals, with PABPC1, GTF2I, PCLO, PKD1, and EP400 being the most frequent. SKAT/SKAT-O revealed the strongest burden associations for motor delay, aggressive behavior, mutism, anxiety, unresponsiveness to spoken voice, digestive disorder, and sleep disturbances, with limited overlap across phenotypes. Several recurrent genes also showed phenotype-specific associations. Overall, this integrative WES study provides clinically actionable diagnoses, highlights recurrent genes, and uncovers phenotype-specific signals, supporting convergent pathways with gene-level heterogeneity.

## 1. Introduction

Autism Spectrum Disorder (ASD) is a highly heterogeneous neurodevelopmental condition that results from the interaction of genetic susceptibility, environmental exposures, and epigenetic regulatory factors that influence early brain development [1]. ASD is characterized by persistent impairments in social-communication, restricted and repetitive behaviors and atypical sensory processing. However, its clinical presentation varies considerably among individuals in severity, developmental trajectory, and comorbid features [2]. This phenotypic variability reflects a multifactorial biological architecture in which both common and rare genetic variants, gene-environment interactions, and developmental timing contribute to the emergence of distinct clinical profiles. Over the last decade, the widespread implementation of genomic technologies, especially whole-exome sequencing (WES), has substantially expanded our understanding of ASD genetics, revealing hundreds of implicated genes across diverse neurobiological pathways, including synaptic function, chromatin remodeling, neuronal migration, and neuroimmune regulation [3].

In this study, we performed WES in a population of children with ASD stratified by clinical severity. The primary objectives were to characterize the genetic architecture of the cohort, identify recurrent rare variants and enriched genes, and explore whether specific genetic findings are associated with severity differences or other phenotypic features including intellectual disability, language disorder, and motor delay. By integrating genomic data with molecular biomarkers and detailed clinical profiles, this work aims to provide new insights into the biological basis of ASD heterogeneity. In addition, it aims to identify candidate genes and molecular pathways linked to specific phenotypic features, with potential implications for disease stratification, diagnosis, prognosis, and future personalized therapeutic strategies.

## 2. Results

### 2.1. Characteristics of the Patients

A total of 25 individuals with ASD were included in the study; ages ranged from 2 to 12 years (mean 7.08 ± 3.45), with 5 females and 20 males (male-to-female ratio 4:1). Based on standardized clinical evaluation, severity was classified using DSM-5 support levels: Level 1 (mild; n = 8), Level 2 (moderate; n = 9), and Level 3 (severe; n = 8). Most children had typical early development prior to symptom onset (72%), while 28% had ASD associated with antecedent neurological or early medical conditions. Several patients exhibited comorbidities including intellectual disability, epilepsy, language impairment, or developmental delay, although some participants presented an isolated condition. Intellectual delay was frequent (80%), and communication/language impairments were common (communication disorders 76%; language disorders 72%). Motor stereotypies (84%) and repetitive behaviors (92%) were highly prevalent, while motor delay affected nearly half of the population (48%). Epilepsy or seizure-related phenotypes were present in 24%, and additional behavioral comorbidities included ADHD (60%), aggressive behavior (48%), anxiety (32%), sleep disturbances (48%), echolalia (48%), and digestive disorders (20%) (Table 1).

### 2.2. Overview of Genetic Diagnoses

Whole-exome sequencing identified pathogenic or likely pathogenic variants in 9 out of 25 patients with ASD, corresponding to a diagnostic yield of 36%. A total of 9 pathogenic or likely pathogenic variants were identified, each affecting a distinct gene, and associated with neurodevelopmental, epileptic, metabolic, immunological, and syndromic disorders. According to ACMG criteria, 5 variants were classified as pathogenic (P) and 4 as likely pathogenic (LP) (Table 2).

The observed inheritance patterns included autosomal dominant disorders (N = 5) and autosomal recessive disorders (N = 3), while one case showed variable inheritance with incomplete penetrance. Autosomal recessive conditions were confirmed by homozygous variants inherited from heterozygous parents. Among the autosomal dominant cases, three pathogenic variants occurred de novo. Whenever parental samples were available, segregation analysis supported the expected mode of inheritance. One family carrying a pathogenic truncating variant in SCN2A (c.4303C>T; p.Arg1435Ter) provided further evidence of autosomal dominant inheritance. The variant was identified in the proband, the father, and an affected sibling, all of whom presented with neurodevelopmental impairments, including intellectual disability and delays in motor, language, and seizure-related development. The co-segregation of the variant with the phenotype across affected family members was consistent with autosomal dominant transmission. The PIK3R2 variant was observed in a child with prenatal hydrocephalus, macrocephaly, motor and language delay, intellectual delay, and seizures, consistent with the megalencephaly-polymicrogyria-postaxial polydactyly-hydrocephalus spectrum. The SHH variant was identified in a patient with epilepsy, motor delay, intellectual delay, anxiety, and communication difficulties. The POLD3 homozygous variant occurred in a child with neonatal respiratory distress, intestinal anemia, short stature, developmental delay, anxiety, stereotypies, and language impairment; the gene is associated with immunodeficiency, and recurrent infections were documented. The COA3 homozygous variant was observed in a patient with language delay, intellectual impairment, abnormal EEG findings, and neurodevelopmental regression, compatible with a mitochondrial-related neurodevelopmental presentation. For SLC6A1, the c.-92-1G>A variant was interpreted using transcript NM_003042.4 and corresponds to a canonical splice-acceptor variant at the -1 intronic position; it was rare in gnomAD, had supportive splice prediction evidence, and has been reported in ClinVar literature, supporting a likely pathogenic classification in the context of intellectual disability, communication disorder, mutism, language disorder, motor stereotypies, and ASD features. For ALDH5A1, the c.*1789C>T variant was interpreted using transcript NM_001080.4 as a rare 3′ UTR variant. Although it has not been previously described, the variant was homozygous in the affected patient and both parents were heterozygous carriers, consistent with autosomal recessive inheritance. The patient’s phenotype, including seizures, developmental delay, motor delay, intellectual delay, language disorder, and communication disorder, was consistent with succinic semialdehyde dehydrogenase deficiency, supporting likely pathogenic classification based on rarity, segregation, and phenotype specificity. Thus, integration of segregation data, transcript-level annotation, population frequency, splice prediction, and patient-level phenotypic correlation strengthened the interpretation of P/LP variants while identifying cases requiring cautious follow-up or additional functional validation.

The genetically established diagnoses included developmental and epileptic encephalopathies (SCN2A, KCNQ3), syndromic neurodevelopmental disorders (NSD1, PIK3R2, SHH), synaptic and neurotransmission-related disorders (SLC6A1), mitochondrial disease (COA3), metabolic disorder (ALDH5A1), immunodeficiency (POLD3), and an immune-related susceptibility locus (HLA-DRB1). Several variants had been previously reported in ClinVar, whereas others had not been previously described.

### 2.3. Identification of Recurrently Altered Genes in a WES Population

After filtration and aggregation across the 25 sequenced individuals, a total of 586 recurrent genes were identified, defined as genes harboring at least one qualifying variant in two or more individuals. The most frequently observed genes in this exploratory recurrence analysis were PABPC1, detected in 18 individuals, followed by GTF2I (15 individuals), and PCLO and PKD1 (13 individuals each). EP400 and MUC5B were each observed in 12 individuals, while SEPTIN9, FAT4, ZFHX3, and ACAN were recurrent in 11 individuals. Additional genes such as LRP2, DST, CELSR2, CENPE, and HTT appeared in 10 individuals, indicating consistent recurrence across the population. Many of the highly recurrent genes are associated with neurodevelopmental or multisystem disorders. Several genes with established ASD relevance, including PABPC1, GTF2I, PCLO, PKD1, EP400, ZFHX3, LRP2, and DST, were annotated as present in the SFARI Gene database, whereas other recurrent genes such as MUC5B, ACAN, FAT4, SEPTIN9, CELSR2, CENPE, and HTT were not listed in SFARI despite frequent recurrence. Table 3 summarizes the top 5% (30 genes) most recurrent genes ranked by the number of individuals in which they were detected.

**Figure 1:**
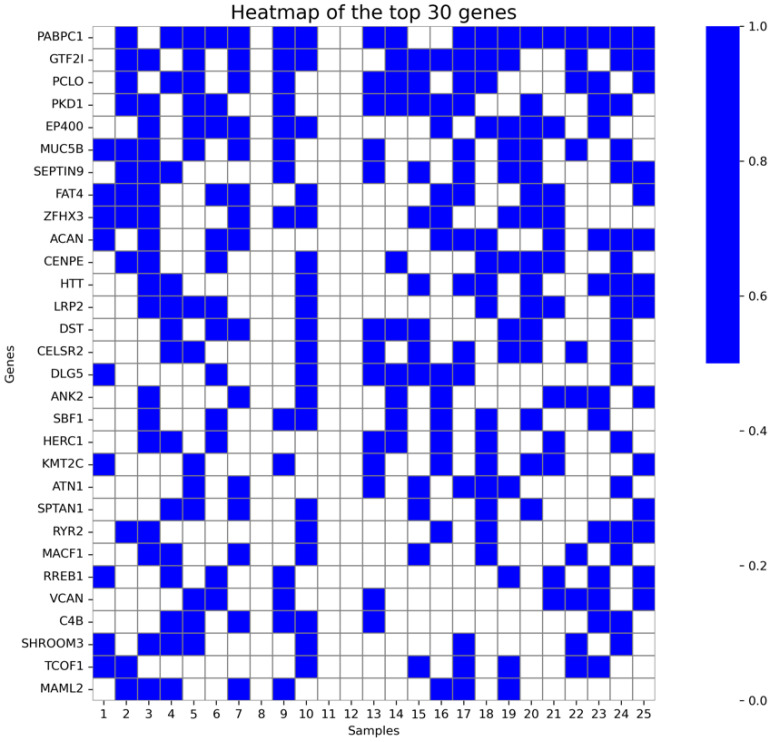
Heatmap of the top 5% recurrent genes.

Within these recurrent genes, 295 rare variants were identified across the 25 individuals (Table S1). Some individuals carried more than one rare variant within the same gene, suggesting intra-gene clustering of rare variation. The distribution of recurrent genes across individuals is illustrated in the heatmap (Figure 1), which highlights heterogeneous but recurrent patterns of gene presence; notably, patients 8, 11, and 12 did not harbor variants in the recurrent gene set displayed in the heatmap.

The five most recurrent genes are associated with neurodevelopmental or multisystem disorders and were all annotated as present in SFARI genes database. PABPC1 has been linked to developmental delay and is frequently reported in de novo dominant cases. GTF2I is associated with Williams syndrome and neurodevelopmental phenotypes overlapping ASD. PCLO has been implicated in pontocerebellar hypoplasia type 3 and severe neurodevelopmental impairment. PKD1 is primarily linked to autosomal dominant polycystic kidney disease, including syndromic forms with neurological involvement. EP400 has been associated with epilepsy and neurodevelopmental disorders.

### 2.4. Identification of Recurrently Altered Variant in a WES Population Through Population-Based Enrichment Testing

At the variant level, aggregation across the 25 sequenced individuals identified 220 shared variants present in more than one individual (Table S2). These variants were tested for cohort enrichment by comparing carrier frequencies with population allele frequencies from gnomAD using a one-sided Fisher’s exact test. Six variants showed nominal enrichment at *p* < 0.05 (Table 4). However, after applying a Benjamini–Hochberg false discovery rate (FDR) correction for multiple testing, only two variants remained significant. The most significant recurrent variant was PABPC1 (chr8:100706805:G>DEL), detected in 18 individuals, all heterozygous (carrier frequency 66.7%, *p* = 4.01 × 10^−7^, FDR = 8.82 × 10^−5^; gnomAD AF = 0). This variant spans an intronic region and is predicted to disrupt the splice polypyrimidine tract, suggesting a high functional impact on gene regulation. The second enriched variant that remained significant after correction was GTF2I (chr7:74753919:A>G), present in 14 individuals, all homozygous (carrier frequency 51.9%, *p* = 5.10 × 10^−5^, FDR = 0.0056; gnomAD AF = 0). This variant is synonymous and corresponds to c.2715A>G (p.Val905=). Although it does not alter the amino acid sequence, it was classified as a variant of uncertain significance (VUS) by Franklin and showed notable recurrence within the study.

The C4B variant (chr6:32034963:G>A) was identified in 7 individuals (6 heterozygous, 1 homozygous; carrier frequency 25.9%, *p* = 0.024, FDR = 1.0; gnomAD AF = 0). Three additional variants reached nominal significance but did not survive multiple-testing correction: OXCT1 (chr5:41850114:C>A), MAML2 (chr11:96092219:C>INS), and SET (chr9:128683961:G>T), each present in 6 individuals (carrier frequency 22.2%, *p* = 0.049, FDR = 1.0 for all). Among these, PABPC1, GTF2I, C4B and SET were annotated as present in SFARI genes, whereas MAML2 and OXCT1 were not.

### 2.5. Population Structure and PCA Adjustment

**Figure 2:**
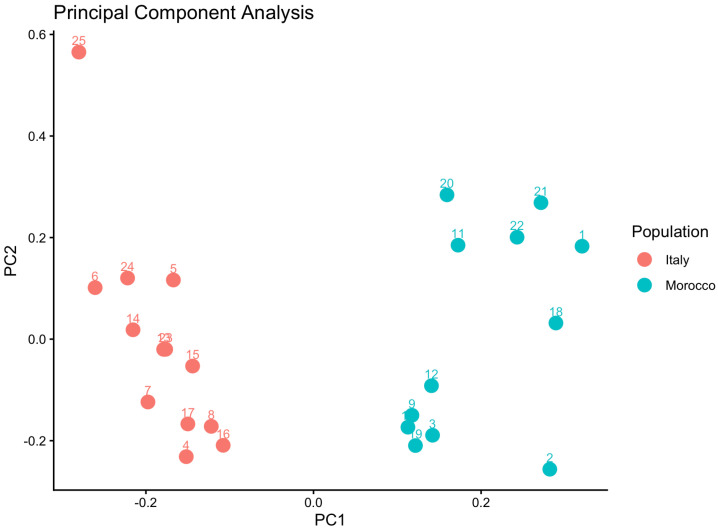
Principal component analysis (PCA) of the ASD cohort showing the genetic distribution of Moroccan and Italian participants.

Principal component analysis was performed on LD-pruned common variants to evaluate ancestry structure within the combined Moroccan/Italian cohort. The PCA showed clear separation between Moroccan and Italian samples, primarily along PC1, with additional dispersion along PC2. Accordingly, PC1 and PC2 were included as covariates in all SKAT/SKAT-O null models together with clinical covariates (Figure 2).

### 2.6. Genotype-Phenotype Association

Gene-based SKAT/SKAT-O analyses were conducted across 16 phenotype categories after adjustment for age at testing, sex, neonatal complications, prematurity, family history of autism, consanguinity, PC1, and PC2. Sample sizes ranged from 23 to 25 individuals, and 14,172–14,351 genes were tested per phenotype (Table 5).

At the nominal level, several phenotypes showed gene-level association signals, including echolalia (13,016 genes with *p* < 0.05), language disorder (7529), unresponsiveness to spoken voice (4587), anxiety (3487), motor delay (3444), sleep disturbances (3240), aggressive behavior (3049), mutism (2825), digestive disorder (2568), motor stereotypies (1530), and severity (622). No nominally significant genes were observed for ASD with typical early development prior to symptom onset, communication disorders, intellectual delay, seizure, or ADHD.

**Figure 3:**
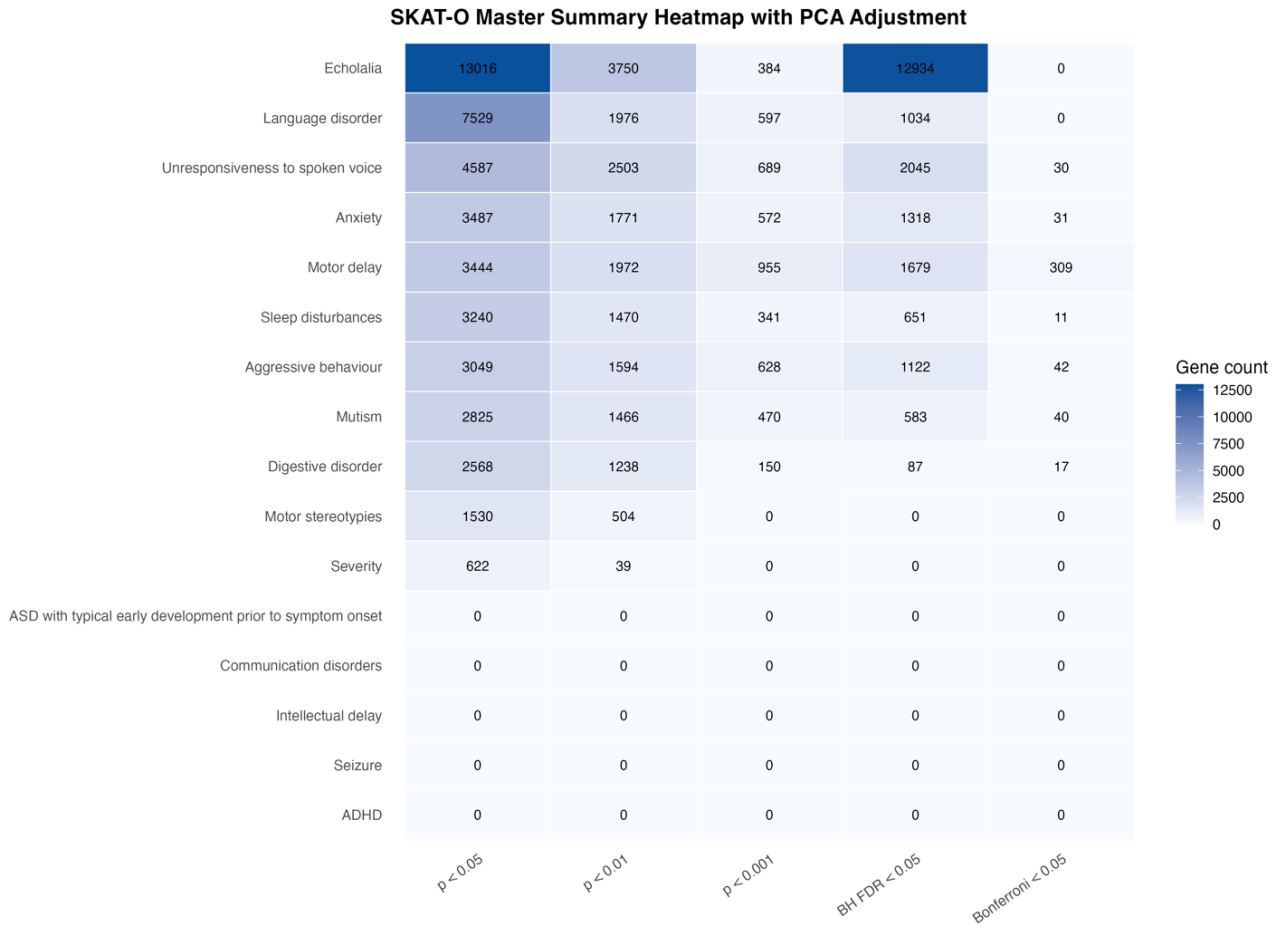
Heatmap showing the number of significant genes identified per phenotype across different statistical thresholds and correction methods (*p* < 0.05, *p* < 0.01, *p* < 0.001, BH-adjusted significance, Bonferroni-adjusted significance).

After Benjamini–Hochberg correction, the largest number of significant genes was observed for echolalia (12,934), followed by unresponsiveness to spoken voice (2045), motor delay (1679), anxiety (1318), aggressive behavior (1122), language disorder (1034), sleep disturbances (651), mutism (583), and digestive disorder (87). After Bonferroni correction, significant genes remained for motor delay (309), aggressive behavior (42), mutism (40), anxiety (31), unresponsiveness to spoken voice (30), digestive disorder (17), and sleep disturbances (11). Echolalia and language disorder retained Benjamini–Hochberg significant genes but did not retain Bonferroni-significant genes. Severity, ASD with typical early development prior to symptom onset, communication disorders, intellectual delay, motor stereotypies, seizure, and ADHD did not show corrected gene-level associations (Figure 3).

Together, these results indicate that after adjustment for population structure and clinical covariates, the strongest corrected gene-level burdens were concentrated in motor delay, aggressive behavior, mutism, anxiety, unresponsiveness to spoken voice, digestive disorder, and sleep disturbances. Full PCA-adjusted gene-level results, including raw *p*-values, Benjamini–Hochberg adjusted *p*-values, Bonferroni-adjusted *p*-values, variant counts, carrier counts, carrier percentages, and tested sample sizes, are provided in Table S3. Q-Q plots for each phenotype are provided in Figure S1.

### 2.7. Top Gene Hits

The most significant gene-level association, defined by the lowest *p*-value, was observed for motor delay with UNC5D, an axon guidance receptor involved in neuronal migration and connectivity (*p* = 1.57 × 10^−17^; BH-adjusted *p* = 1.06 × 10^−13^; Bonferroni-adjusted *p* = 2.22 × 10^−13^). For digestive disorder, the strongest association was identified for TMEM126B, a mitochondrial transmembrane protein potentially related to cellular energy metabolism (*p* = 1.16 × 10^−10^; BH-adjusted *p* = 1.66 × 10^−6^; Bonferroni-adjusted *p* = 1.66 × 10^−6^). Mutism showed a leading signal for OR51V1, an olfactory receptor gene with uncertain direct neurodevelopmental relevance (*p* = 2.21 × 10^−10^; BH-adjusted *p* = 3.17 × 10^−6^; Bonferroni-adjusted *p* = 3.17 × 10^−6^). Aggressive behavior was associated with PPP1R21, a protein phosphatase regulatory subunit potentially involved in intracellular signaling (*p* = 9.07 × 10^−10^; BH-adjusted *p* = 1.30 × 10^−5^; Bonferroni-adjusted *p* = 1.30 × 10^−5^). For unresponsiveness to spoken voice, the top hit was SSUH2 (*p* = 2.16 × 10^−9^; BH-adjusted *p* = 3.08 × 10^−5^; Bonferroni-adjusted *p* = 3.08 × 10^−5^). Anxiety was associated with BID, a gene involved in apoptotic signaling pathways (*p* = 6.69 × 10^−9^; BH-adjusted *p* = 9.59 × 10^−5^; Bonferroni-adjusted *p* = 9.59 × 10^−5^). Sleep disturbances showed a top signal for PTGER3, a prostaglandin E receptor involved in inflammatory and neurophysiological signaling (*p* = 8.03 × 10^−9^; BH-adjusted *p* = 1.15 × 10^−4^; Bonferroni-adjusted *p* = 1.15 × 10^−4^).

Among phenotypes with Benjamini–Hochberg but not Bonferroni significance, echolalia was led by BCS1L, a mitochondrial complex III assembly factor (*p* = 3.80 × 10^−4^; BH-adjusted *p* = 1.48 × 10^−2^; Bonferroni-adjusted *p* = 1.00), while language disorder was led by BCL2L2, an apoptosis-related gene from the BCL2 family (*p* = 1.70 × 10^−4^; BH-adjusted *p* = 1.02 × 10^−2^; Bonferroni-adjusted *p* = 1.00). Severity showed a top nominal signal for DNAJC27 (*p* = 1.92 × 10^−3^), but this did not survive Benjamini–Hochberg or Bonferroni correction. ASD with typical early development prior to symptom onset, communication disorders, intellectual delay, seizure, ADHD, and motor stereotypies did not show statistically significant top gene-level associations and multiple-testing correction. Because SKAT/SKAT-O is a gene-based variance-component test, beta coefficients and single effect directions were not estimated; therefore, gene-level results are reported using raw *p*-values, BH-adjusted *p*-values, Bonferroni-adjusted *p*-values, variant counts, and carrier counts (Table 5).

### 2.8. Shared vs. Unique Signals Across Phenotypes

No single gene was significant across all phenotypes at either Benjamini–Hochberg or Bonferroni thresholds. The maximum cross-phenotype overlap at the Benjamini–Hochberg level was five phenotypes, indicating that the corrected signals remained largely phenotype-specific, although partial sharing was observed across related clinical domains (Table S4). The genes reaching this maximum overlap included NRXN2, OR5H15, SPANXD, IFNB1, PHYH, RELA, ASPN, and OR3A4P. Among these, NRXN2 is of particular interest because neurexin genes are involved in synaptic organization and neuronal connectivity, whereas RELA and IFNB1 may reflect inflammatory or transcriptional regulatory mechanisms.

The highest pairwise sharing of Benjamini–Hochberg significant genes was observed between echolalia and unresponsiveness to spoken voice (1817 shared genes), followed by echolalia and motor delay (1440 shared genes), echolalia and anxiety (1119), echolalia and aggressive behavior (1063), echolalia and language disorder (877), echolalia and sleep disturbances (608), and echolalia and mutism (516). Additional pairwise overlap was observed between language disorder and motor delay (349 shared genes), motor delay and unresponsiveness to spoken voice (325), anxiety and mutism (294), anxiety and unresponsiveness to spoken voice (210), and sleep disturbances and unresponsiveness to spoken voice (169).

Focusing on the most strongly associated shared genes, the overlap between motor delay and unresponsiveness to spoken voice included ASB8 (motor delay: *p* = 2.42 × 10^−11^, BH-adjusted *p* = 2.70 × 10^−9^, Bonferroni-adjusted *p* = 3.43 × 10^−7^; unresponsiveness to spoken voice: *p* = 1.65 × 10^−7^, BH-adjusted *p* = 2.66 × 10^−4^, Bonferroni-adjusted *p* = 2.36 × 10^−3^), a member of the ankyrin repeat and SOCS box protein family involved in ubiquitin-mediated protein regulation; NDUFB9 (*p* = 4.21 × 10^−10^, BH-adjusted *p* = 3.66 × 10^−8^, Bonferroni-adjusted *p* = 5.97 × 10^−6^; *p* = 2.57 × 10^−7^, BH-adjusted *p* = 3.06 × 10^−4^, Bonferroni-adjusted *p* = 3.67 × 10^−3^), a mitochondrial complex I subunit involved in oxidative phosphorylation; CENPP (*p* = 3.15 × 10^−7^, BH-adjusted *p* = 1.83 × 10^−5^, Bonferroni-adjusted *p* = 4.47 × 10^−3^; *p* = 2.05 × 10^−7^, BH-adjusted *p* = 2.66 × 10^−4^, Bonferroni-adjusted *p* = 2.92 × 10^−3^), a centromere protein involved in chromosome segregation; and ARHGEF16 (*p* = 7.26 × 10^−10^, BH-adjusted *p* = 6.13 × 10^−8^, Bonferroni-adjusted *p* = 1.03 × 10^−5^; *p* = 4.65 × 10^−7^, BH-adjusted *p* = 3.80 × 10^−4^, Bonferroni-adjusted *p* = 6.64 × 10^−3^), a Rho guanine nucleotide exchange factor involved in cytoskeletal signaling. Together, these shared genes point toward ubiquitin-mediated regulation, mitochondrial function, chromosomal organization, and cytoskeletal signaling as possible shared mechanisms across motor and sensory-response phenotypes.

A second shared cluster between language disorder and motor delay included GLRX5 (language disorder: *p* = 1.71 × 10^−4^, BH-adjusted *p* = 1.02 × 10^−2^; motor delay: *p* = 1.57 × 10^−4^, BH-adjusted *p* = 3.68 × 10^−3^), a mitochondrial glutaredoxin involved in iron-sulfur cluster biogenesis; ATG7 (*p* = 1.77 × 10^−4^, BH-adjusted *p* = 1.02 × 10^−2^; *p* = 7.31 × 10^−5^, BH-adjusted *p* = 1.97 × 10^−3^), an autophagy-related gene essential for autophagosome formation and cellular quality control; ARHGAP25 (*p* = 1.77 × 10^−4^, BH-adjusted *p* = 1.02 × 10^−2^; *p* = 7.30 × 10^−5^, BH-adjusted *p* = 1.97 × 10^−3^), a Rho GTPase-activating protein involved in cytoskeletal regulation and immune-cell signaling; GRK1 (*p* = 1.77 × 10^−4^, BH-adjusted *p* = 1.02 × 10^−2^; *p* = 4.21 × 10^−5^, BH-adjusted *p* = 1.23 × 10^−3^), a G protein-coupled receptor kinase best known for sensory phototransduction; and MTHFD1L (*p* = 1.77 × 10^−4^, BH-adjusted *p* = 1.02 × 10^−2^; *p* = 1.75 × 10^−5^, BH-adjusted *p* = 6.44 × 10^−4^), a mitochondrial one-carbon metabolism enzyme relevant to folate-dependent nucleotide synthesis and cellular methylation pathways. Although these shared genes did not generally reach Bonferroni significance in both phenotypes, their Benjamini–Hochberg significance suggests partial convergence between language and motor domains through mitochondrial metabolism, autophagy, cytoskeletal regulation, GPCR signaling, and one-carbon metabolic pathways.

Unique-signal analysis reinforced phenotype-level heterogeneity. The largest set of unique Benjamini–Hochberg significant genes was observed for echolalia (6980 unique genes), followed by unresponsiveness to spoken voice (136), motor delay (125), anxiety (112), language disorder (95), aggressive behavior (44), mutism (19), sleep disturbances (15), and digestive disorder (2). Examination of the most prominent unique signals revealed distinct phenotype-specific profiles. Motor delay included MLH1 (*p* = 2.10 × 10^−11^, BH-adjusted *p* = 2.56 × 10^−9^, Bonferroni-adjusted *p* = 2.98 × 10^−7^), a DNA mismatch-repair gene involved in genomic stability; LCE4A (*p* = 2.85 × 10^−10^, BH-adjusted *p* = 2.52 × 10^−8^, Bonferroni-adjusted *p* = 4.04 × 10^−6^), a late cornified envelope gene with less direct neurodevelopmental characterization; and C3orf52 (*p* = 3.62 × 10^−10^, BH-adjusted *p* = 3.17 × 10^−8^, Bonferroni-adjusted *p* = 5.13 × 10^−6^), a poorly characterized locus requiring cautious interpretation. Anxiety was characterized by PTGER1 (*p* = 2.39 × 10^−7^, BH-adjusted *p* = 3.43 × 10^−4^, Bonferroni-adjusted *p* = 3.43 × 10^−3^), a prostaglandin E receptor involved in inflammatory and neurophysiological signaling, and IGSF21 (*p* = 3.97 × 10^−7^, BH-adjusted *p* = 4.07 × 10^−4^, Bonferroni-adjusted *p* = 5.69 × 10^−3^), an immunoglobulin superfamily gene with potential roles in cell adhesion and neurodevelopmental processes. Mutism showed unique corrected signals in OR51V1 (*p* = 2.21 × 10^−10^, BH-adjusted *p* = 3.17 × 10^−6^, Bonferroni-adjusted *p* = 3.17 × 10^−6^), an olfactory receptor gene that may reflect sensory-related signaling; FBXO41 (*p* = 3.28 × 10^−8^, BH-adjusted *p* = 6.35 × 10^−5^, Bonferroni-adjusted *p* = 4.71 × 10^−4^), an F-box protein involved in ubiquitin-mediated protein regulation and reported in neuronal development contexts; and DENND5A (*p* = 2.37 × 10^−6^, BH-adjusted *p* = 9.99 × 10^−4^, Bonferroni-adjusted *p* = 3.40 × 10^−2^), a DENN-domain protein implicated in vesicle trafficking and Rab GTPase regulation. Together, these findings indicate that many gene-level associations are phenotype-specific, with partial sharing across communication, sensory-response, behavioral, and motor domains.

### 2.9. Integration with the Top-30 Recurrent Genes

We next examined SKAT-O signals within the Top-30 recurrent genes identified in the recurrence analysis. Within this focused gene set, 27 of the 30 recurrent genes showed Benjamini–Hochberg significant association in at least one phenotype, although none reached Bonferroni-level significance. The Benjamini–Hochberg significant Top-30 signals were largely concentrated in echolalia, where 27 recurrent genes were significant, including ACAN, ANK2, ATN1, CELSR2, CENPE, DST, EP400, FAT4, GTF2I, HERC1, HTT, KMT2C, LRP2, MACF1, MUC5B, PABPC1, PCLO, PKD1, RREB1, RYR2, SBF1, SEPTIN9, SHROOM3, SPTAN1, TCOF1, VCAN, and ZFHX3. Outside echolalia, additional Benjamini–Hochberg significant Top-30 signals included DST in unresponsiveness to spoken voice (*p* = 3.71 × 10^−5^, BH-adjusted *p* = 5.94 × 10^−3^), RYR2 in anxiety (*p* = 7.07 × 10^−4^, BH-adjusted *p* = 2.09 × 10^−2^), ACAN in aggressive behavior (*p* = 1.03 × 10^−3^, BH-adjusted *p* = 2.33 × 10^−2^), SHROOM3 (*p* = 2.47 × 10^−3^, BH-adjusted *p* = 2.67 × 10^−2^), GTF2I (*p* = 3.32 × 10^−3^, BH-adjusted *p* = 3.29 × 10^−2^), PABPC1 (*p* = 3.55 × 10^−3^, BH-adjusted *p* = 3.47 × 10^−2^), and CENPE (*p* = 4.86 × 10^−3^, BH-adjusted *p* = 4.39 × 10^−2^) in motor delay, MUC5B (*p* = 2.06 × 10^−3^, BH-adjusted *p* = 3.60 × 10^−2^) and CELSR2 (*p* = 3.09 × 10^−3^, BH-adjusted *p* = 4.55 × 10^−2^) in language disorder, VCAN in unresponsiveness to spoken voice (*p* = 2.48 × 10^−3^, BH-adjusted *p* = 2.84 × 10^−2^), and CENPE in sleep disturbances (*p* = 1.68 × 10^−3^, BH-adjusted *p* = 4.36 × 10^−2^).

**Figure 4:**
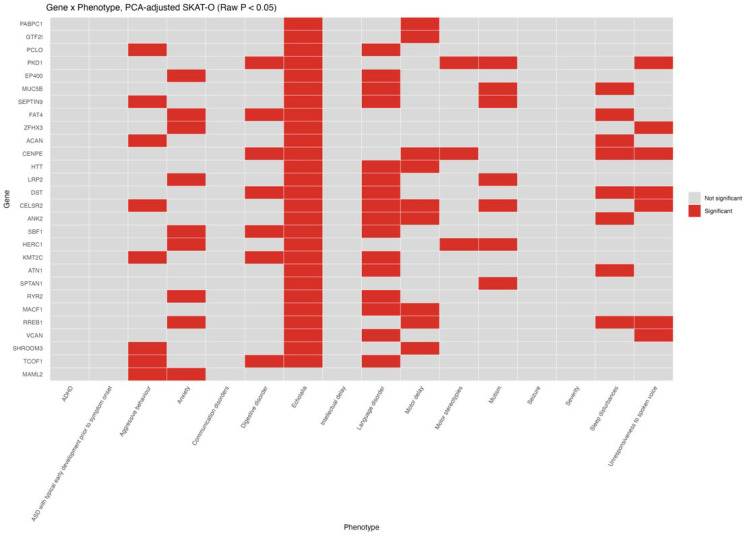
SKAT-O associations within the Top-30 recurrent genes across ASD phenotypes.

At the nominal level, several recurrent genes were implicated across multiple phenotypes. CELSR2 and CENPE each showed nominal association in six phenotypes, while DST, PKD1, and RREB1 were nominally associated in five phenotypes. Additional recurrent genes with nominal signals in four phenotypes included ANK2, FAT4, HERC1, KMT2C, LRP2, MUC5B, SBF1, SEPTIN9, and TCOF1. At the phenotype level, Top-30 recurrent genes were most frequently implicated in echolalia (27 genes), followed by language disorder (16), anxiety and motor delay (9 each), aggressive behavior and sleep disturbances (8 each), digestive disorder, mutism, and unresponsiveness to spoken voice (7 each), and motor stereotypies (3) (Figure 4). These findings suggest that recurrent genes identified at the variant-recurrence level also show phenotype-linked gene-based burden in the SKAT-O framework, particularly across echolalia, language, motor, sensory-response, and behavioral domains.

## 3. Discussion

This study combined clinical stratification, recurrence analysis, and gene-based association testing to investigate the genetic architecture of ASD in a well-characterized pediatric cohort. In line with large-scale ASD sequencing studies, our results reinforce the genetic heterogeneity of ASD and the involvement of multiple biological pathways, especially synaptic function, transcriptional regulation, and chromatin biology, which have repeatedly emerged as core themes in ASD risk gene discovery [4].

The diagnostic yield of pathogenic or likely pathogenic variants in our study (36%) was higher than yields reported in similar neurodevelopmental studies [5,6], reflecting enrichment for complex phenotypes (intellectual disability, seizures, developmental delay) and targeted recruitment of families with suspected genetic etiologies [7]. These results support the clinical utility of WES in phenotyped ASD cohorts and align with consensus statements that exome sequencing is a high-yield first-tier test in neurodevelopmental disorders [8].

Gene-recurrence exploratory analysis identified several genes that converge on biological processes repeatedly associated with ASD. PABPC1, the most recurrent gene in our study, encodes a poly(A)-binding protein central to mRNA stabilization and translation, linking recurrence to post-transcriptional regulation, a mechanism increasingly recognized in ASD [9,10]. The recurrence of GTF2I, a transcriptional regulator within the Williams-Beuren syndrome region, provides a transcriptional-control signal consistent with broader ASD gene architecture [11]. The identification of PCLO, a presynaptic cytomatrix protein important for synaptic organization and vesicle trafficking, anchors the recurrence signal within synaptic biology [12]. EP400, part of chromatin-modifying complexes, adds a chromatin remodeling dimension that mirrors major ASD gene-set enrichments [13,14]. In addition, FAT4 (planar cell polarity/atypical cadherin signaling) and DST (cytoskeletal integrity) introduce structural and cellular architecture pathways that can plausibly intersect with neurodevelopmental phenotypes [15,16]. The presence of multiple SFARI-annotated genes within the recurrent set further supports external validity of these signals.

Variant-level enrichment analysis identified a small number of recurrent variants with nominal significance. Given the small sample size, a lack of normalization for gene length, regional callability, or mutational constraint, these results should be interpreted lightly. Nevertheless, they provide potentially informative candidates for independent replication and functional validation studies particularly those variants observed in multiple unrelated individuals within the study.

SKAT/SKAT-O analyses allowed phenotype-specific gene-based testing of rare variation while accounting for both clinical covariates and population structure. This approach is well suited to rare-variant data because SKAT-O adaptively combines burden and variance-component testing, maintaining power when variants within the same gene may have heterogeneous effect directions or effect sizes [17]. Because PCA demonstrated clear ancestry structure within the combined Moroccan/Italian cohort, PC1 and PC2 were included in the null models together with age at testing, sex, neonatal complications, prematurity, family history of autism, and consanguinity. This adjustment provided a more controlled framework for interpreting gene-level burden across ASD-related phenotypic domains.

After PCA adjustment and multiple-testing correction, the strongest corrected gene-level burdens were observed for motor delay, aggressive behavior, mutism, anxiety, unresponsiveness to spoken voice, digestive disorder, and sleep disturbances. This distribution suggests that certain ASD-related domains may be driven by stronger rare-variant effects, whereas others may be more polygenic or influenced by non-coding/regulatory mechanisms, an interpretation consistent with broader ASD genetics studies [18,19,20].

Gene-level interpretation of phenotype signals points to biologically coherent themes. The top motor-delay signal in UNC5D, a netrin receptor implicated in axon guidance and neuronal migration, supports a neurodevelopmental wiring mechanism [18]. This is consistent with the broader role of axon guidance and synaptic connectivity pathways in ASD and related neurodevelopmental phenotypes.

The phenotype-specific signals also pointed to additional biological themes. Unresponsiveness to spoken voice was led by SSUH2, while anxiety was associated with BID, a gene involved in apoptotic signaling [19]. Sleep disturbances were led by PTGER3, a prostaglandin receptor implicated in inflammatory and neurophysiological signaling [20], and digestive disorder was led by TMEM126B, a mitochondrial transmembrane gene [21]. Mutism showed a strong corrected signal in OR51V1, an olfactory receptor gene whose direct neurodevelopmental relevance remains uncertain but may reflect sensory-related signaling [22]. These findings suggest that the PCA-adjusted gene-level burden is not uniform across all ASD traits, but is enriched in specific motor, behavioral, sensory-response, sleep, and systemic phenotypic domains.

The shared-gene analysis further supports a model of partial convergence with substantial phenotype specificity. No gene was significant across all phenotypes, and the maximum Benjamini–Hochberg overlap was limited to five phenotypes. Shared genes included NRXN2, OR5H15, SPANXD, IFNB1, PHYH, RELA, ASPN, and OR3A4P. Among these, NRXN2 is especially relevant because neurexins are synaptic adhesion molecules involved in synapse organization and neuronal connectivity, and NRXN2 disruption has been linked to ASD and related neurodevelopmental disorders [23]. RELA and IFNB1 may point toward inflammatory or immune-regulatory mechanisms, whereas PHYH and ASPN suggest lipid metabolism and extracellular-matrix contributions. However, olfactory receptor-related and poorly characterized genes should be interpreted cautiously.

The integration of recurrence analysis and SKAT-O provides convergent evidence for certain loci. Especially in echolalia and related communication or sensory-response phenotypes. DST, RYR2, ACAN, SHROOM3, GTF2I, PABPC1, CENPE, MUC5B, CELSR2, and VCAN showed corrected or nominal associations across selected phenotypes. These patterns suggest that recurrence-based prioritization and gene-based burden testing capture partly overlapping signals, particularly involving genes related to cellular architecture, extracellular matrix organization, cytoskeletal regulation, synaptic structure, and developmental signaling. These convergence points highlight candidates for deeper interpretation in future cohorts. Taken together, the recurrence and association results support a model of ASD that is genetically heterogeneous but organized around convergent pathways such as synaptic organization, transcriptional regulation, chromatin remodeling, and cellular architecture, consistent with large-scale ASD genomics [22].

Clinically, these results emphasize the value of integrating detailed phenotyping with genomic testing. The observation that distinct phenotypic domains showed different burdens of gene-level association suggests that clinical stratification can improve the interpretability of rare variants beyond a global ASD diagnosis. This is particularly relevant in cohorts enriched for associated neurodevelopmental features, where motor delay, seizures, intellectual disability, language impairment, sensory-response abnormalities, sleep disturbances, and gastrointestinal symptoms may reflect partly distinct genetic liabilities. In this study, the convergence of recurrent genes with phenotype-specific SKAT/SKAT-O signals provided a rational basis for prioritizing candidate genes and highlighted pathways that may inform future clinical surveillance, such as monitoring motor development, seizure risk, sleep disturbance, or systemic manifestations in patients carrying variants in relevant functional pathways.

This interpretation is consistent with large ASD exome studies and recent genotype-phenotype analyses, which have shown that autism is shaped by a broad spectrum of rare and common genetic influences, including de novo protein-truncating variants, damaging missense variants, inherited rare coding variants, copy-number variants, and polygenic background. These studies have identified high-confidence ASD risk genes enriched in chromatin remodeling, transcriptional regulation, synaptic organization, neuronal communication, and early brain developmental pathways [24,25,26,27,28]. They have also shown that rare variant burden is not evenly distributed across ASD, but is often associated with intellectual disability, developmental delay, epilepsy, reduced adaptive functioning, and other complex neurodevelopmental features [25,26,27,28]. In line with this broader model, our recurrence and SKAT/SKAT-O analyses support substantial gene-level heterogeneity with convergence on neurodevelopmental, synaptic, regulatory, cytoskeletal, mitochondrial, and extracellular-matrix pathways. The phenotype-specific burdens observed across motor, sensory-response, behavioral, sleep, and gastrointestinal domains further support the use of detailed clinical stratification to refine genotype-phenotype interpretation in ASD.

A key advantage of this study is its multi-layer design, which combines clinical diagnosis, recurrence analysis, enrichment testing, and gene-based association. External gene prioritization using SFARI Gene adds an evidence-based framework for interpreting recurrent signals, while SKAT-O provides a robust rare-variant association strategy. This study has several limitations, including the relatively modest sample size, which reduced the statistical power of the SKAT/SKAT-O analyses performed across multiple phenotypes and thousands of genes. Consequently, these analyses should be considered exploratory and hypothesis-generating rather than definitive gene-discovery analyses, and both false-positive and false-negative findings remain possible despite multiple-testing correction. Replication in larger independent cohorts will be essential to confirm the reported genotype-phenotype associations. A further limitation is the use of both a clinical sequencing workflow and an in-house analytical pipeline; however, this was unavoidable because complete VCF files were not available from the clinical platform for association testing. Despite these constraints, the integration of detailed clinical phenotyping with genomic analyses provides a valuable framework for future validation studies in larger ASD cohorts. In addition, the recurrent gene analysis was designed as a descriptive assessment of genes harboring rare qualifying variants across unrelated ASD probands rather than as a formal burden analysis. Gene recurrence was therefore not normalized for gene length, regional callability, or mutational constraint, factors that may contribute to the increased recurrence of large or highly polymorphic genes. Likewise, recurrent variants identified in technically challenging genomic regions, including genes such as PKD1, should be interpreted cautiously because they contain highly homologous duplicated regions that are challenging for short-read sequencing, and warrant confirmation using orthogonal approaches such as long-read sequencing or targeted validation. Future studies incorporating larger cohorts, gene-length-adjusted burden analyses, and independent validation will be important to establish the robustness of these findings. Furthermore, the absence of a matched control group limits our ability to directly distinguish ASD-specific rare variation from general population background noise; inclusion of such controls would have enabled a more straightforward comparison between ASD cases and the general population. Another limitation is the exclusive use of whole-exome sequencing (WES), which does not capture non-coding regulatory regions, structural variants, or repeat expansions that may contribute to ASD risk. As demonstrated by the study by Anna Grether et al., on patients with developmental or epileptic encephalopathies [29], whole-genome sequencing (WGS) can identify clinically relevant variants missed by WES, suggesting that our approach may underestimate the full genetic architecture underlying the observed phenotypes. Overall, this study demonstrates that WES analysis of a clinically stratified ASD population can yield actionable diagnoses, reveal recurrent genes enriched across individuals, and identify phenotype-specific gene associations. The results support a model in which ASD arises from convergent biological pathways but remains genetically heterogeneous at the gene level. Future studies involving larger cohorts, and functional validation will be essential to confirm these findings and to translate the most compelling candidate genes into clinical and biological insights.

## 4. Materials and Methods

### 4.1. Study Design

This was an observational, cross-sectional study conducted as part of a collaborative effort between the Mohammed VI University of Sciences and Health (UM6SS) in Casablanca, Morocco and the University of Messina in Italy. The study was carried out over a three-year period, from 2023 to 2025.

### 4.2. Patient Recruitment

In this study we included children with both ASD with typical early development prior to symptom onset and ASD with antecedent neurological insult or early medical condition associated with additional neurodevelopmental or medical conditions, from the Pediatric Neurology Department of the University Hospital Center (UHC) of Rabat and the Messina Polyclinic. A detailed family history was collected for all recruited subjects including: number of siblings; age of parents; family history of prior psychological or psychiatric disorders; consanguinity and developmental milestones. These clinical and familial features were used to support the interpretation and prioritization of genetic findings obtained through whole-exome sequencing (WES). Clinical severity was assessed using standardized diagnostic instruments, including the Autism Diagnostic Observation Schedule (ADOS), the Childhood Autism Rating Scale (CARS), and DSM-5 criteria. For segregation analysis, both parents of each proband were recruited whenever available. All participants underwent peripheral blood sampling for genomic DNA extraction. Written informed consent was obtained from parents or legal guardians in accordance with institutional and national guidelines. This study was conducted as part of biomedical research projects approved by the Ethics Committee of the University Hospital “G. Martino”, Messina, Italy (minutes No. 7 of the meeting held on 19 June 2017).

### 4.3. Phenotypic Stratification Strategy

To explore genotype-phenotype relationships beyond the global ASD diagnosis, patients were stratified according to clinically defined phenotypic domains extracted from standardized clinical records and neurological evaluations. Phenotypes were coded as binary variables indicating the presence or absence of each feature, except for ASD severity, which was analyzed as an ordinal/continuous phenotype based on clinical severity. The phenotypic domains included core ASD-related features, such as communication disorders, language disorder, mutism, echolalia, repetitive behavior, unresponsiveness to spoken voice, and motor stereotypies, as well as associated neurodevelopmental or medical features, including intellectual delay, motor delay, seizures, sleep disturbances, ADHD, anxiety, aggressive behavior, digestive disorder, and neonatal/perinatal complications. This stratification was designed to capture the clinical heterogeneity of ASD and to test whether rare variant burden differed across specific behavioral, cognitive, motor, sensory-response, and systemic domains.

### 4.4. Genomic DNA Extraction

Peripheral venous blood was collected in EDTA tubes from all participants. Genomic DNA was isolated using the PureLink Genomic DNA Mini Kit (Invitrogen, Thermo Fisher Scientific, USA) following the manufacturer’s protocol. DNA purity was assessed using a NanoDrop Lite spectrophotometer (Thermo Fisher Scientific, Waltham, MA, USA), and double-stranded DNA concentrations were measured with the Qubit 4 Fluorometer (Invitrogen, Thermo Fisher Scientific, Waltham, MA, USA). Whole-exome sequencing was performed using the library; preparation was performed using the Illumina DNA Prep with Exome 2.5 Enrichment Kit (Illumina, San Diego, CA, USA) to selectively capture coding regions of the human genome. Genomic DNA was enzymatically fragmented, end-repaired, A-tailed, and ligated to indexed adapters. Libraries were then hybridized with exon-specific probes, enriched via magnetic bead capture, and amplified. Library quality and fragment size were assessed using fluorometric quantification (Qubit 4 Fluorometer, Invitrogen, Waltham, MA, USA) and the Agilent TapeStation system (Agilent Technologies, Santa Clara, CA, USA). Qualified libraries were sequenced on an Illumina NextSeq 550 platform (Illumina, San Diego, CA, USA) in paired-end mode.

### 4.5. Bioinformatics Workflow Overview

Variant analysis was performed using two complementary approaches: (1) the Emedgene clinical genomics platform with Illumina DRAGEN 4.3 for clinical interpretation [30,31], and (2) an in-house WES pipeline to generate full VCF files for study-level analyses and genotype–phenotype association testing.

#### 4.5.1. Step 1: Clinical Platform Analysis

FASTQ files from each proband and, when available, parents were uploaded to Emedgene along with patient clinical data in Human Phenotype Ontology (HPO) terms. The DRAGEN pipeline performed quality control and adapter trimming, alignment to GRCh38, duplicate marking, base quality score recalibration, and variant calling. The system supported detection of SNVs, small indels, and copy-number/structural variants. Variants were annotated using public databases, literature evidence, and proprietary knowledge sources integrated in Emedgene (V36.0) [30].

#### 4.5.2. Step 2: Clinical Interpretation and Pathogenicity Assessment

Initial filtering retained coding and canonical splice-site variants (±2 bp) with read depth ≥ 10, genotype quality ≥ 20, and population allele frequency < 0.5% in gnomAD. Priority was given to variants affecting coding or splice regions, rare in the population, or previously reported as pathogenic/likely pathogenic in OMIM [32], Orphanet [33], ClinVar [34], HGMD, or CentoMD. Variants were classified as pathogenic, likely pathogenic, VUS, likely benign, or benign according to ACMG guidelines [35].

#### 4.5.3. Step 3: Recurrent Variant and Gene Enrichment

To identify recurrent variants and genes within the population, WES data from 25 individuals were filtered using predefined quality and frequency criteria. Variants were required to have a sequencing depth ≥ 10×, mapping quality (MQ) ≥ 45, and a gnomAD allele frequency ≤ 0.01. The analysis retained rare coding variants, including missense, predicted loss-of-function, splice-region/splice-site, and in-frame insertion/deletion variants. Gene-level recurrence was assessed by counting individuals carrying at least one variant per gene; genes present in only one individual were excluded. Unique variants per gene were aggregated along with their frequencies. Annotations (disease association, inheritance mode, functional prediction) were summarized by retaining the first non-missing entry per gene or variant. Genes were annotated for ASD relevance using the SFARI Gene database [36].

A secondary variant-level analysis calculated cohort allele frequencies by zygosity (heterozygous, homozygous, hemizygous). For variants shared across multiple individuals, cohort carrier frequencies were compared with gnomAD population frequencies using a one-sided Fisher’s exact test. Because of the limited sample size, comparisons were not stratified by ancestry, sex, or exome capture callability. All processing and analyses were performed in Python 3.14 (pandas, numpy, scipy).

#### 4.5.4. Step 4: In-House VCF Generation for Association Testing

Because the Emedgene platform provides limited variant export (CSV with a maximum number of variants and in theirs analysis they do not joint genotype of the patients because they treat the data probant by probant or in a trio), an in-house pipeline was used to generate full VCF files required for gene-based association testing. Reads were aligned to GRCh38 using BWA (v0.7.19) [37], converted and sorted with samtools (v1.22), and indexed. PCR duplicates were marked using Picard (MarkDuplicates) (v3.4). Base quality score recalibration was performed with GATK (v4.5) [38] BaseRecalibrator and ApplyBQSR using known-sites references. Coverage was assessed with bedtools (v2.31) coverage and GATK DepthOfCoverage using the Twist Bioscience/Illumina Exome 2.5 capture target BED file. Variants were called with GATK HaplotypeCaller in GVCF mode, followed by joint genotyping (CombineGVCFs and GenotypeGVCFs). Hard filters (based on variant quality, mapping quality, strand bias, and read-position bias) were applied separately to SNVs and indels using GATK VariantFiltration. Variants were annotated with rsIDs using dbSNP [39], and further annotated with ANNOVAR (v2024Oct24) (hg38) [40].

#### 4.5.5. Step 5: Genotype-Phenotype Association

VCFs were converted to PLINK format for SKAT/SKAT-O [17]. To assess ancestry and population structure within the combined Moroccan/Italian cohort, principal component analysis (PCA) was performed using common variants after linkage disequilibrium pruning in PLINK (v1.9.0-b.7.7). Variants with minor allele frequency ≥ 0.05 were retained, and independent variants were selected using a sliding-window LD pruning approach (--indep-pairwise 50 5 0.2). The first ten genetic principal components were generated, and PC1 and PC2 were visualized to evaluate clustering by population.

Gene-based SKAT/SKAT-O tests were then performed with adjustment for demographic, clinical, and ancestry covariates. For each phenotype, the null model included age at testing, sex, neonatal complications, prematurity, family history of autism, consanguinity, and the leading ancestry principal components. Each phenotype was tested separately using SKAT/SKAT-O. For binary phenotypes, affected individuals were compared with individuals in the cohort who did not present the corresponding feature. For ASD severity, the ranking variable was analyzed as a quantitative/ordinal outcome. Based on the observed PCA structure, PC1 and PC2 were included as covariates in the association models to account for population stratification.

Gene-level associations were tested using weighted linear-kernel SKAT-O, with fallback to SKAT when SKAT-O failed to converge. Only genes with at least two mapped variants were tested. Multiple testing correction was performed using both Bonferroni correction and the Benjamini–Hochberg false discovery rate procedure. Analyses were performed in R (v4.5.2) using the SKAT, data.table, BEDMatrix, and ggplot2 packages.

Family members were analyzed exclusively for segregation and inheritance assessment and were not included in the burden or other statistical analyses, which were restricted to unrelated ASD probands.

## 5. Conclusions

This study employed a multi-pronged approach, clinical stratification, recurrence analysis, and gene-based association testing, to delineate the genetic architecture of ASD in a pediatric population. WES achieved a diagnostic yield of 36% and identified both recurrent candidate genes and phenotype-specific association signals. Recurrent genes highlighted pathways linked to transcriptional regulation, synaptic organization, and neurodevelopment, while SKAT/SKAT-O revealed stronger corrected burdens for intellectual delay, seizures, motor delay, and unresponsiveness to spoken voice. Convergence between recurrence and association analyses supports several candidate genes for further investigation. Despite limitations of sample size and multiple testing, this integrated framework provides clinically relevant insights and a foundation for larger, ancestry-matched studies with functional validation and multi-omic analyses.

## Figures and Tables

**Table 1:** Clinical characteristics by Autism severity.

	Mild	Moderate	Severe	Total
	(N = 8)	(N = 9)	(N = 8)	(N = 25)
Age at testing (years)				
Mean (SD)	5.56 (3.16)	6.44 (3.91)	9.31 (2.12)	7.08 (3.45)
Median [Min, Max]	4.75 [2.00, 12.0]	6.00 [3.00, 12.0]	9.25 [6.00, 12.0]	7.00 [2.00, 12.0]
Gender				
Female	2 (25.0%)	0 (0%)	3 (37.5%)	5 (20.0%)
Male	6 (75.0%)	9 (100%)	5 (62.5%)	20 (80.0%)
Neonatal complications				
No	6 (75.0%)	8 (88.9%)	5 (62.5%)	19 (76.0%)
Yes	2 (25.0%)	1 (11.1%)	3 (37.5%)	6 (24.0%)
Premature birth				
No	7 (87.5%)	8 (88.9%)	6 (75.0%)	21 (84.0%)
Yes	1 (12.5%)	1 (11.1%)	2 (25.0%)	4 (16.0%)
Family history of autism				
No	6 (75.0%)	3 (33.3%)	5 (62.5%)	14 (56.0%)
Yes	2 (25.0%)	6 (66.7%)	3 (37.5%)	11 (44.0%)
Consanguinity				
Yes	1 (12.5%)	2 (22.2%)	1 (12.5%)	4 (16.0%)
No	7 (87.5%)	7 (77.8%)	7 (87.5%)	21 (84.0%)
Autism type				
ASD with typical early development prior to symptom onset	8 (100%)	7 (77.8%)	3 (37.5%)	18 (72.0%)
ASD with antecedent neurological insult or early medical condition	0 (0%)	2 (22.2%)	5 (62.5%)	7 (28.0%)
Aggressive behavior				
Yes	4 (50.0%)	4 (44.4%)	4 (50.0%)	12 (48.0%)
No	4 (50.0%)	5 (55.6%)	4 (50.0%)	13 (52.0%)
Anxiety				
No	6 (75.0%)	7 (77.8%)	4 (50.0%)	17 (68.0%)
Yes	2 (25.0%)	2 (22.2%)	4 (50.0%)	8 (32.0%)
Communication disorders				
Yes	4 (50.0%)	7 (77.8%)	8 (100%)	19 (76.0%)
No	4 (50.0%)	2 (22.2%)	0 (0%)	6 (24.0%)
Mutism				
No	7 (87.5%)	5 (55.6%)	4 (50.0%)	16 (64.0%)
Yes	1 (12.5%)	4 (44.4%)	4 (50.0%)	9 (36.0%)
Intellectual delay				
Yes	5 (62.5%)	7 (77.8%)	8 (100%)	20 (80.0%)
No	3 (37.5%)	2 (22.2%)	0 (0%)	5 (20.0%)
Language disorder				
Yes	3 (37.5%)	9 (100%)	6 (75.0%)	18 (72.0%)
No	5 (62.5%)	0 (0%)	2 (25.0%)	7 (28.0%)
Motor delay				
No	4 (50.0%)	6 (66.7%)	1 (12.5%)	11 (44.0%)
Yes	4 (50.0%)	3 (33.3%)	5 (62.5%)	12 (48.0%)
Missing	0 (0%)	0 (0%)	2 (25.0%)	2 (8.0%)
Motor stereotypies				
Yes	8 (100%)	7 (77.8%)	6 (75.0%)	21 (84.0%)
No	0 (0%)	2 (22.2%)	2 (25.0%)	4 (16.0%)
Seizure				
No	8 (100%)	7 (77.8%)	4 (50.0%)	19 (76.0%)
Yes	0 (0%)	2 (22.2%)	4 (50.0%)	6 (24.0%)
Sleep disturbances				
No	3 (37.5%)	4 (44.4%)	6 (75.0%)	13 (52.0%)
Yes	5 (62.5%)	5 (55.6%)	2 (25.0%)	12 (48.0%)
Echolalia				
Yes	4 (50.0%)	2 (22.2%)	6 (75.0%)	12 (48.0%)
No	4 (50.0%)	7 (77.8%)	0 (0%)	11 (44.0%)
Missing	0 (0%)	0 (0%)	2 (25.0%)	2 (8.0%)
Repetitive behavior				
Yes	7 (87.5%)	9 (100%)	7 (87.5%)	23 (92.0%)
No	1 (12.5%)	0 (0%)	1 (12.5%)	2 (8.0%)
Unresponsiveness to spoken voice				
No	7 (87.5%)	5 (55.6%)	6 (75.0%)	18 (72.0%)
Yes	1 (12.5%)	3 (33.3%)	2 (25.0%)	6 (24.0%)
Missing	0 (0%)	1 (11.1%)	0 (0%)	1 (4.0%)
ADHD				
Yes	4 (50.0%)	4 (44.4%)	7 (87.5%)	15 (60.0%)
No	4 (50.0%)	5 (55.6%)	0 (0%)	9 (36.0%)
Missing	0 (0%)	0 (0%)	1 (12.5%)	1 (4.0%)
Digestive disorder				
No	7 (87.5%)	7 (77.8%)	6 (75.0%)	20 (80.0%)
Yes	1 (12.5%)	2 (22.2%)	2 (25.0%)	5 (20.0%)

**Table 2:** WES-pathogenic and likely pathogenic variants reported.

Patient ID	Gene	Refseq_ID	Variants	Proband Zygosity	Parents Zygosity	Diseases	Disease Inheritance Mode	Variant Inheritance	Pathogenicity	Clinvar
2	SCN2A	NM_001040142.2	c.4303C>T (p.Arg1435Ter)	HET	Father: HET | Mother: REF | Brother: HET	Developmental and epileptic encephalopathy 11	AD|DN	Autosomal dominant	P	Described
3	KCNQ3	NM_004519.4	c.1657G>A (p.Gly553Arg)	HET	Mother: REF	Juvenile myoclonic epilepsy	AD	Autosomal dominant|Autosomal dominant partial penetrance	LP	Described
10	COA3	NM_001040431.3	c.271G>T (p.Glu91Ter)	HOM	Father: HET | Mother: HET	Mitochondrial complex IV deficiency, nuclear type 14	AR	Autosomal recessive homozygotes	LP	Not Described
11	POLD3	NM_006591.3	c.1118A>C (p.Lys373Thr)	HOM	Mother: HET	Immunodeficiency 122	AR	Autosomal recessive homozygotes	P	Described
12	PIK3R2	NM_005027.4	c.1117G>A (p.Gly373Arg)	HET	Father: REF | Mother: REF	Megalencephaly-polymicrogyria-postaxial polydactyly-hydrocephalus syndrome	AD	Autosomal Dominant de novo	LP	Described
13	SLC6A1	NM_001348250.2	c.-92-1G>A	HET	Father: REF | Mother: HET	Autosomal dominant non-syndromic intellectual disability	AD	Autosomal dominant partial penetrance	P	Described
18	SHH	NM_000193.4	c.764T>A (p.Ile255Asn)	HET	Mother: REF	Holoprosencephaly 3	AD	Autosomal dominant|Autosomal dominant partial penetrance	P	Not Described
21	NSD1	NM_022455.5	c.4967-2A>T	HET	Father: REF | Mother: REF | Brother: REF	Sotos syndrome	AD	Autosomal Dominant de novo	P	Described
22	ALDH5A1	NM_001080.3	c.*1789C>T	HOM	Father: HET | Mother: HET	Succinic semialdehyde dehydrogenase deficiency	AR	Autosomal recessive homozygotes	LP	Not Described

**Table 3:** Genes with rare variants frequently identified in the samples.

Gene	Sample_Count	Sfari_Presence	Diseases	Disease_Inheritance_Mode
PABPC1	18	Present	Developmental delay, Autosomal dominant de novo	DN
GTF2I	15	Present	Williams syndrome,	Unknown
PCLO	13	Present	Pontocerebellar hypoplasia, type 3, Autosomal recessive	AR
PKD1	13	Present	Polycystic kidney disease 1, Autosomal dominant | Polycystic kidney disease 1 with or without polycystic liver disease, AD | Autosomal dominant polycystic kidney disease, | Autosomal dominant polycystic kidney disease type 1 with tuberous sclerosis,	AD
EP400	12	Present	Epilepsy with neurodevelopmental disorders, Autosomal recessive	AR
MUC5B	12	Not present	Interstitial lung disease 2, Autosomal dominant | Diffuse panbronchiolitis, | Hypersensitivity pneumonitis| Idiopathic pulmonary fibrosis,	AD
SEPTIN9	11	Not present	Amyotrophy, hereditary neuralgic, Autosomal dominant | Neuralgic amyotrophy,	AD
FAT4	11	Not present	Hennekam lymphangiectasia-lymphedema syndrome 2, Autosomal recessive | Van Maldergem syndrome 2, Autosomal recessive | Cerebrofacioarticular syndrome, | Hennekam syndrome,	AR
ZFHX3	11	Present	Atrial fibrillation 8, susceptibility to, Autosomal dominant | Prostate cancer, somatic, Autosomal dominant, Somatic mutation | Spinocerebellar ataxia 4, Autosomal dominant | Childhood partial epilepsy and infantile spasms, Autosomal recessive | Syndromic intellectual disability (ZFHX3), Autosomal dominant de novo	AD|SO|AR|DN
ACAN	11	Not present	Short stature and advanced bone age, with or without early-onset osteoarthritis and/or osteochondritis dissecans, Autosomal dominant | Spondyloepimetaphyseal dysplasia, aggrecan type, Autosomal recessive | Spondyloepiphyseal dysplasia, Kimberley type, Autosomal dominant | Familial osteochondritis dissecans, Autosomal dominant | Short stature-advanced bone age-early-onset osteoarthritis syndrome,	AD|AR
LRP2	10	Present	Donnai–Barrow syndrome, Autosomal recessive | Faciooculoacousticorenal syndrome, AR	AR
CELSR2	10	Not present	Joubert Syndrome, Autosomal recessive	AR
DST	10	Present	Epidermolysis bullosa simplex 3, localized or generalized intermediate, with bp230 deficiency, Autosomal recessive | Neuropathy, hereditary sensory and autonomic, type VI, Autosomal recessive | Congenital myopathy, Autosomal recessive | Epidermolysis bullosa simplex due to BP230 deficiency, | Hereditary sensory and autonomic neuropathy type 6,	AR
HTT	10	Not present	Huntington disease, Autosomal dominant | Lopes-Maciel-Rodan syndrome, Autosomal recessive | Lopes-Maciel-Rodan syndrome (LOMARS), AD/AR | Juvenile Huntington disease, | Non-specific syndromic intellectual disability,	AD|STR|AR
CENPE	10	Not present	Microcephaly 13, primary, autosomal recessive, Autosomal recessive | Autosomal recessive primary microcephaly, | Seckel syndrome,	AR
DLG5	9	Not present	Yuksel-Vogel-Bauser syndrome, Autosomal recessive | Congenital Heart Disease, Congenital Anomalies including Ciliopathy Phenotypes, Autosomal dominant, Autosomal recessive	AR|AD
ANK2	9	Present	Cardiac arrhythmia, ankyrin-B-related, Autosomal dominant | Long QT syndrome 4, Autosomal dominant | Epilepsy, Autosomal dominant de novo | Romano-Ward syndrome,	AD|DN
SBF1	9	Present	Charcot-Marie-Tooth disease, type 4B3, Autosomal recessive	AR
HERC1	9	Present	Macrocephaly, dysmorphic facies, and psychomotor retardation, Autosomal recessive | Megalencephaly-severe kyphoscoliosis-overgrowth syndrome,	AR
KMT2C	9	Present	Kleefstra syndrome 2, Autosomal dominant | Neurodevelopmental disorder distinct from Kleefstra and Kabuki syndromes, Autosomal dominant, Autosomal dominant de novo | Kleefstra syndrome due to a point mutation,	AD|DN
TCOF1	8	Not present	Treacher Collins syndrome 1, Autosomal dominant | Treacher-Collins syndrome,	AD
RELN	8	Present	Epilepsy, familial temporal lobe, 7, Autosomal dominant | Lissencephaly 2 (Norman-Roberts type), Autosomal recessive | Lissencephaly 2, AD/AR | Epilepsy with auditory features, | Lissencephaly syndrome, Norman-Roberts type,	AD|AR
ANK3	8	Present	Intellectual developmental disorder, autosomal recessive 37, Autosomal recessive | ANK3-related intellectual disability-sleep disturbance syndrome,	AR
PRKDC	8	Present	Immunodeficiency 26, with or without neurologic abnormalities, Autosomal recessive | Severe combined immunodeficiency due to DNA-PKcs deficiency,	AR
RANBP2	8	Not present	Encephalopathy, acute, infection-induced, 3, susceptibility to, Autosomal dominant | Acute necrotizing encephalopathy of childhood, | Familial acute necrotizing encephalopathy, | Inflammatory myofibroblastic tumor,	AD
TENM4	8	Not present	Essential tremor, hereditary, 5, Autosomal dominant | Schizophrenia, Autosomal dominant | Tremor, hereditary essential, 5, AD	AD
MAML2	8	Not present	Mucoepidermoid salivary gland carcinoma,	Unknown
SPTAN1	8	Not present	Developmental and epileptic encephalopathy 5, Autosomal dominant | Developmental delay with or without epilepsy, Autosomal dominant | Neuronopathy, distal hereditary motor, autosomal dominant 11, Autosomal dominant | Spastic paraplegia 91, autosomal dominant, with or without cerebellar ataxia, Autosomal dominant | Distal myopathy, Autosomal dominant de novo, Autosomal dominant | Hereditary spastic paraplegia, Autosomal recessive | Spastic Paraplegia and Cerebellar Ataxia, Autosomal dominant, Autosomal dominant de novo | Neuronopathy, distal hereditary motor, 11, autosomal dominant, AD | Infantile epileptic spasms syndrome,	AD|DN|AR
SHROOM3	8	Not present	Heterotaxy, Autosomal recessive | Neural Tube Defects, Autosomal dominant	AR|AD

**Table 4:** Enriched variants with *p* > 0.05.

Variant Id	Gene	Patient Count	Carrier Freq Cohort	Het Count	Hom Count	GnomAD_af	*p*_Value	fdr_bh
chr8:100706805:G>DEL	PABPC1	18	0.66	18	0	0.0	4.011 × 10^−7^	8.823 × 10^−5^
chr7:74753919:A>G	GTF2I	14	0.51	0	14	0.0	5.096 × 10^−5^	0.005
chr6:32034963:G>A	C4B	7	0.25	6	1	0.0	0.024	1
chr5:41850114:C>A	OXCT1	6	0.22	6	0	0.004	0.049	1
chr11:96092219:C>INS	MAML2	6	0.22	2	4	0.0	0.049	1
chr9:128683961:G>T	SET	6	0.22	6	0	0.003	0.049	1

**Table 5:** Summary of the 16 phenotype categories.

Phenotype Categories	N_Samples	Total Genes	P_lt_0_05	P_lt_0_01	P_lt_0_001	Bonf_Sig	BH_Sig	Top_Gene	Top_*p*-Value
ASD with typical early development prior to symptom onset	25	14,351	0	0	0	0	0	PARL	4.99 × 10^−1^
Severity	25	14,351	622	39	0	0	0	DNAJC27	1.92 × 10^−3^
Aggressive behavior	25	14,351	3049	1594	628	42	1122	PPP1R21	9.07 × 10^−10^
Anxiety	25	14,351	3487	1771	572	31	1318	BID	6.69 × 10^−9^
Communication disorders	25	14,351	0	0	0	0	0	UTP6	4.99 × 10^−1^
Mutism	25	14,351	2825	1466	470	40	583	OR51V1	2.21 × 10^−10^
Intellectual delay	25	14,351	0	0	0	0	0	GSTO2	4.99 × 10^−1^
Language disorder	25	14,351	7529	1976	597	0	1034	BCL2L2	1.7 × 10^−4^
Motor delay	23	14,172	3444	1972	955	309	1679	UNC5D	1.57 × 10^−17^
Motor stereotypies	25	14,351	1530	504	0	0	0	ANGPTL1	1.05 × 10^−3^
Seizure	25	14,351	0	0	0	0	0	CST9	4.99 × 10^−1^
Sleep disturbances	25	14,351	3240	1470	341	11	651	PTGER3	8.03 × 10^−9^
Echolalia	23	14,172	13,016	3750	384	0	12,934	BCS1L	3.8 × 10^−4^
Unresponsiveness to spoken voice	24	14,258	4587	2503	689	30	2045	SSUH2	2.16 × 10^−9^
ADHD	24	14,217	0	0	0	0	0	SALRNA1	4.99 × 10^−1^
**Digestive disorder**	25	14,351	2568	1238	150	17	87	TMEM126B	1.16 × 10^−10^

## Data Availability

All relevant data are within the paper and its Supporting Information files. The datasets generated and/or analyzed during the current study are not publicly available due to the sensitive and confidential nature of patient data, including genetic and clinical information from individuals with autism spectrum disorder. However, de-identified data are available from the corresponding author on reasonable request, subject to institutional ethics approval and appropriate data transfer agreements.

## Supplementary Materials

The following supporting information can be downloaded at: https://www.mdpi.com/article/10.3390/ijms27156901/s1.
