# Supplementary material for: Genetic Heterogeneity in Autism Spectrum Disorder: Diagnostic Yield, Recurrent Genes, and Rare Variant-Phenotype Associations from Whole-Exome Sequencing
Source: Int J Mol Sci. 2026 Aug 1;27(15):6901. doi: 10.3390/ijms27156901 (PMC13467236; doi:10.3390/ijms27156901)
Supplement: Supplementary file 1 [file ijms-27-06901-s001.zip › Figure S1.pdf]

Figure S1: QQ plot of all phenotypes

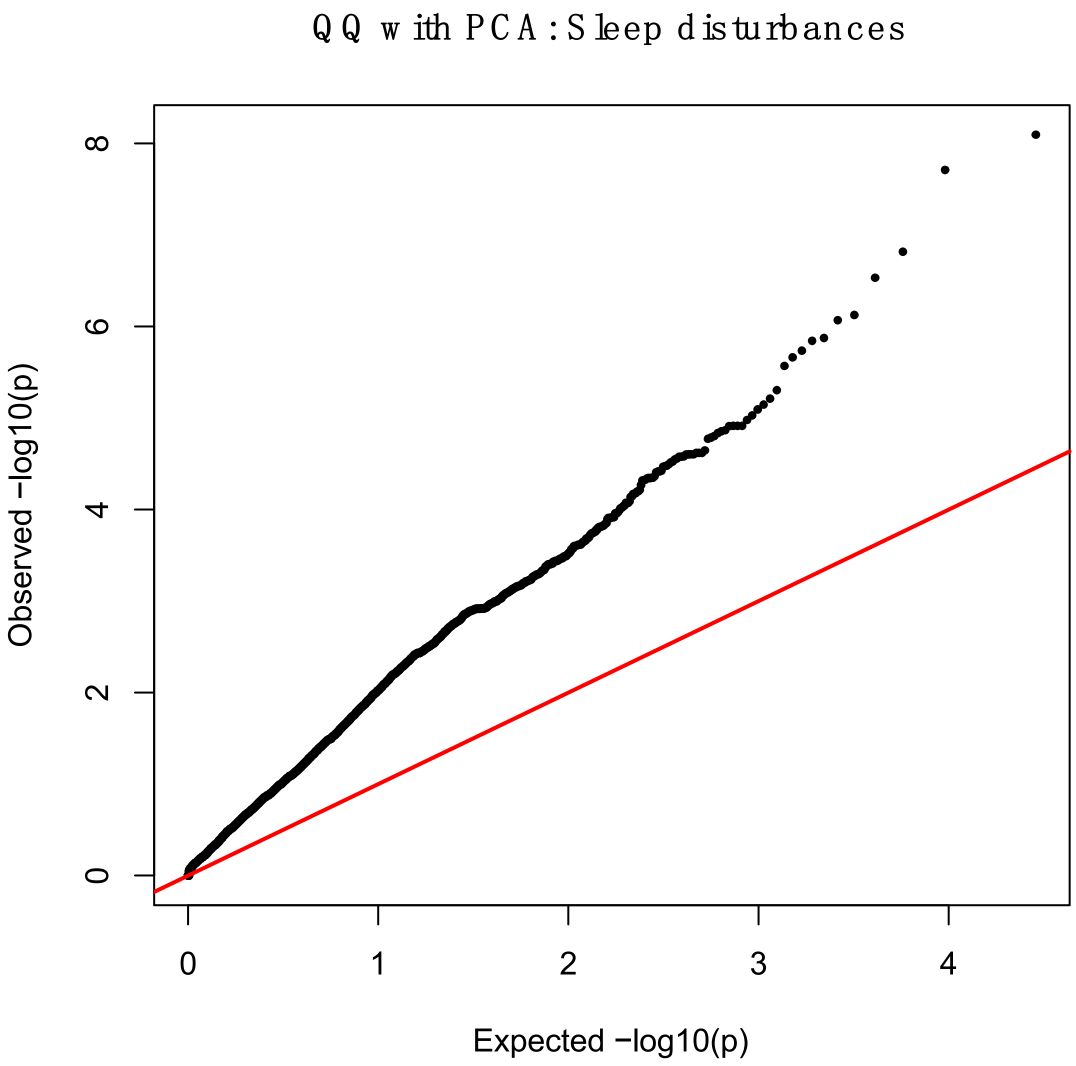

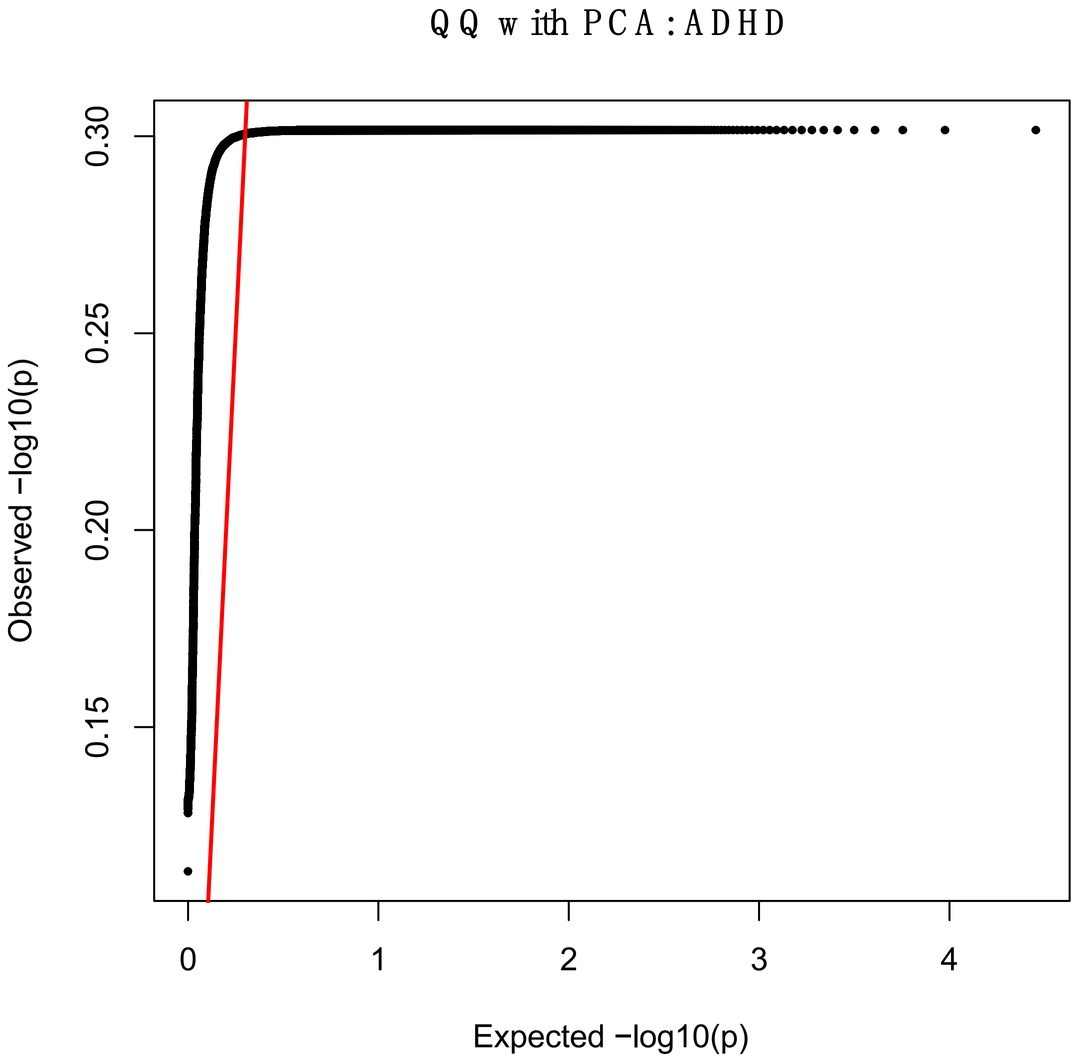
QQ with PCA:ADHD
Observed −log10(p)
0.30
0.25
0.20
0.15
0
1
2
3
4
Expected −log10(p)

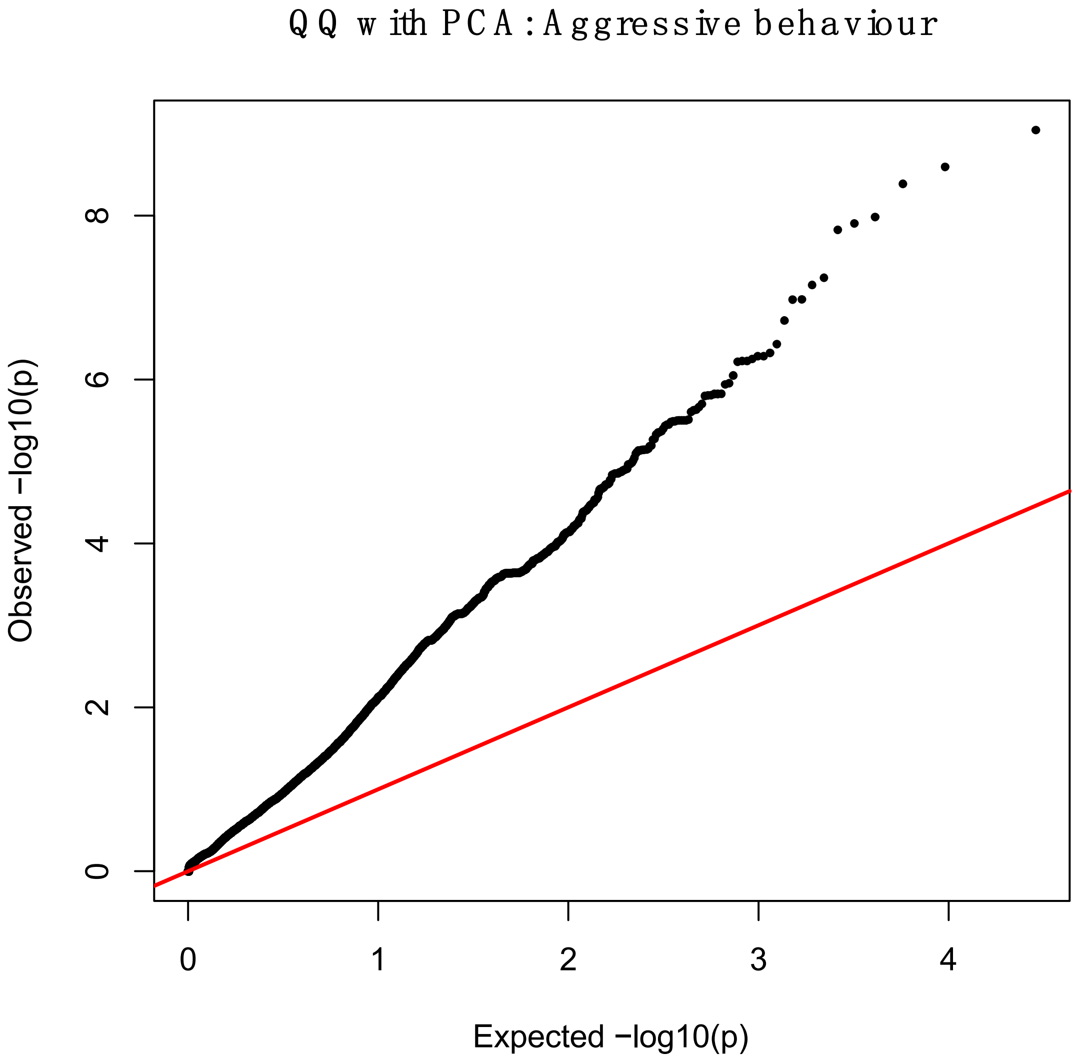

QQ with PCA : Aggressive behaviour
Observed −log10(p)
Expected −log10(p)
0
2
4
6
8
0
1
2
3
4

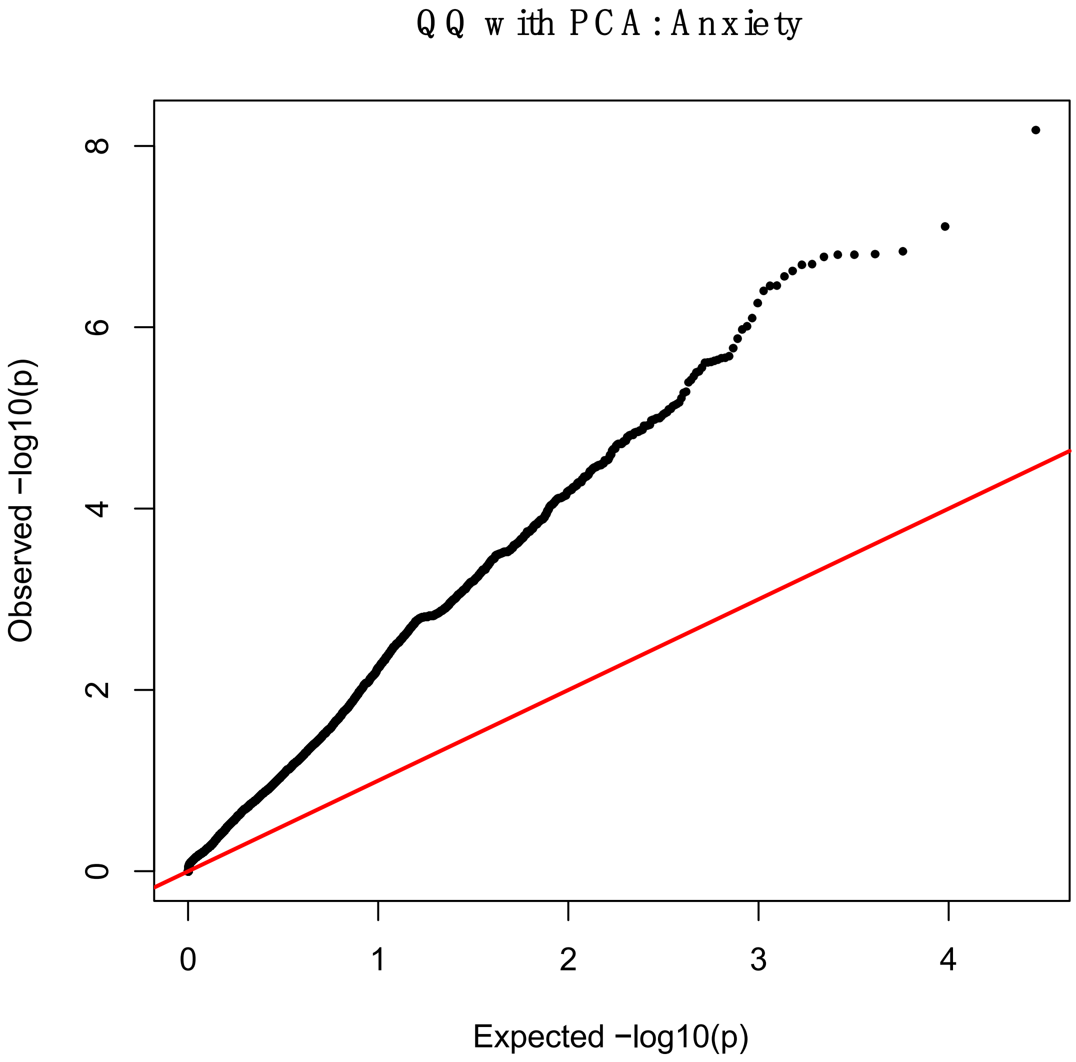
QQ with PCA:Anxiety
Observed −log10(p)
Expected −log10(p)
0
2
4
6
8
0
1
2
3
4

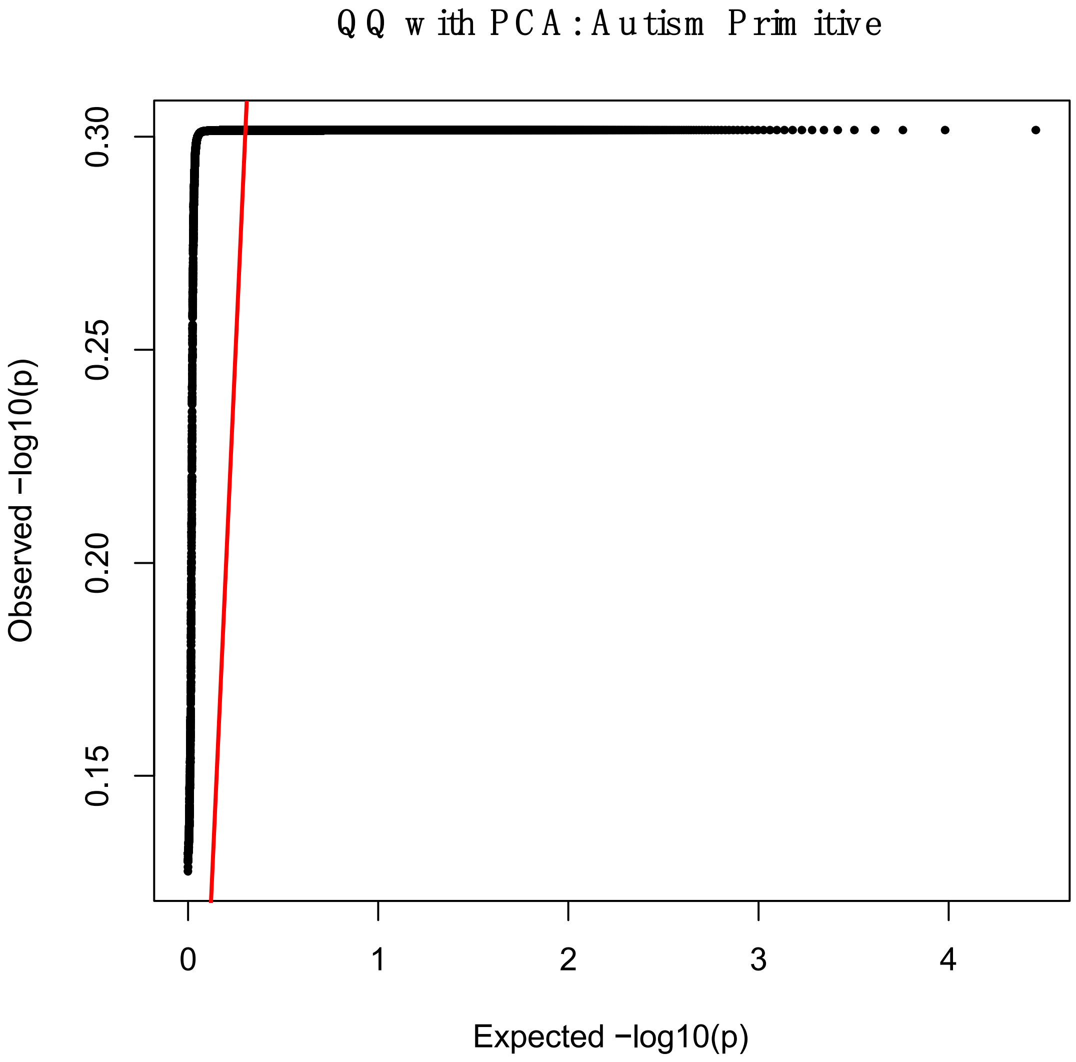
QQ with PCA: Autism Primitive
Observed −log10(p)
0.30
0.25
0.20
0.15
0
1
2
3
4
Expected −log10(p)

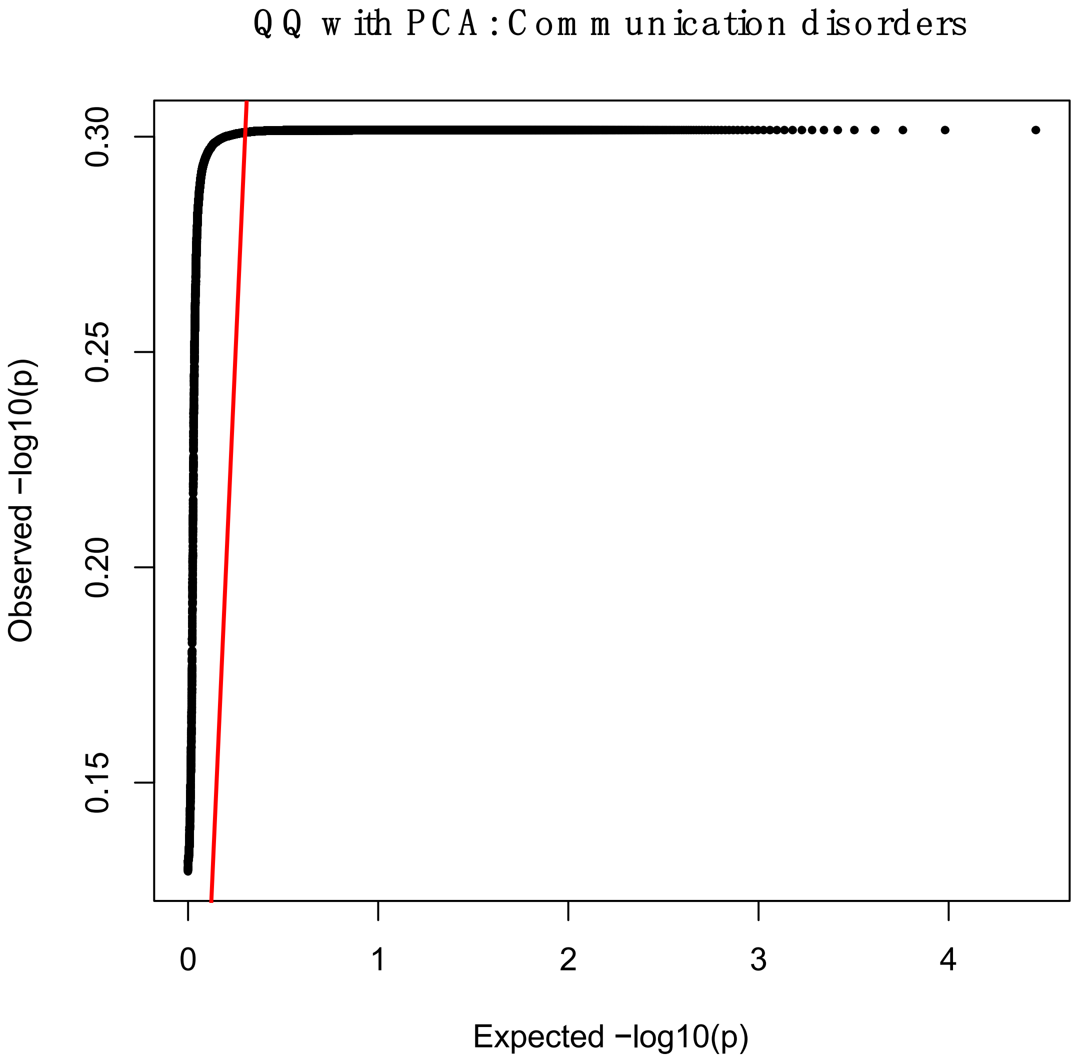

QQ with PCA: Communication disorders
0.30
0.25
0.20
0.15
Observed −log10(p)
0
1
2
3
4
Expected −log10(p)

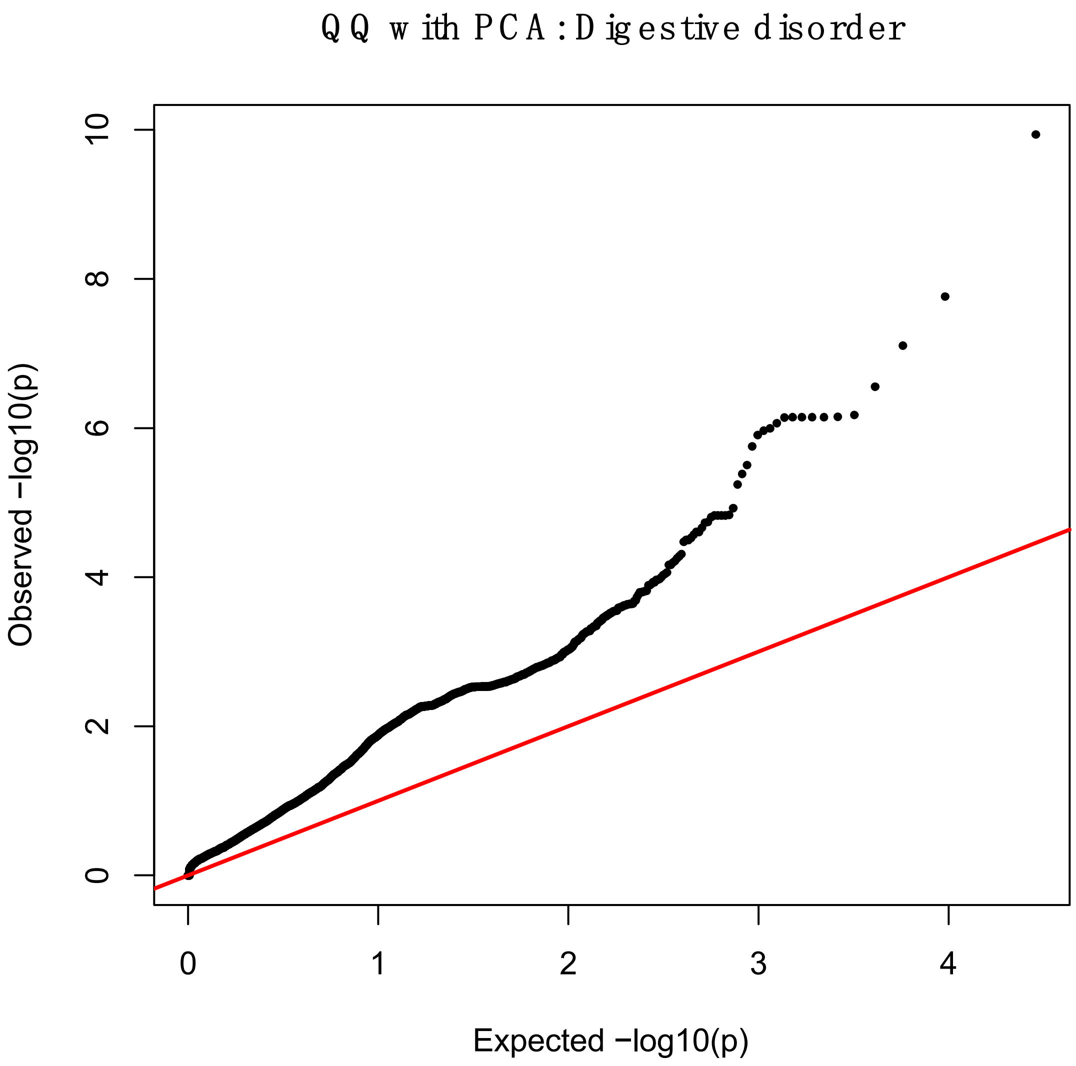
QQ with PCA:Digestive disorder
Observed −log10(p)
Expected −log10(p)
0
2
4
6
8
10
0
1
2
3
4

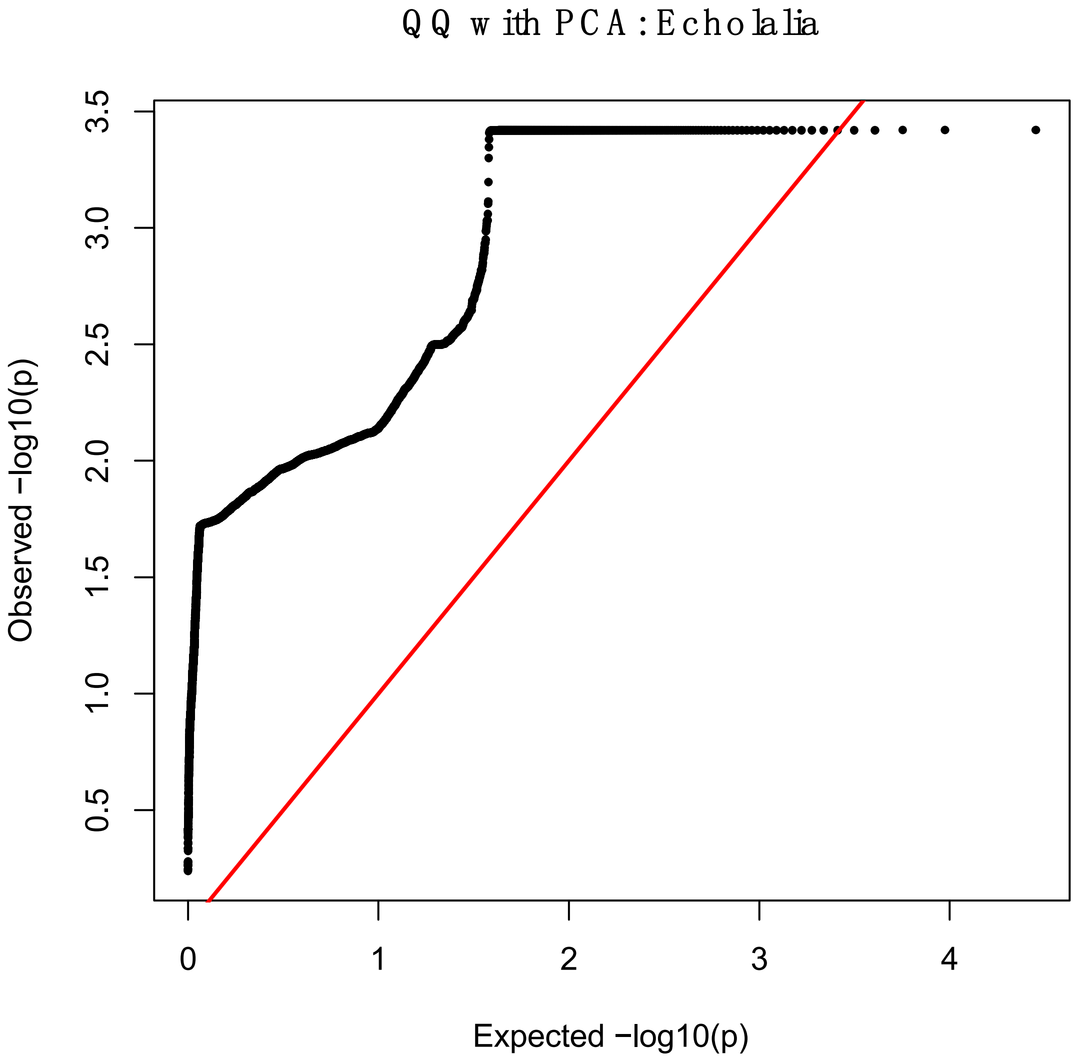

QQ with PCA:Echolalia
Observed −log10(p)
Expected −log10(p)
0.5
1.0
1.5
2.0
2.5
3.0
3.5
0
1
2
3
4

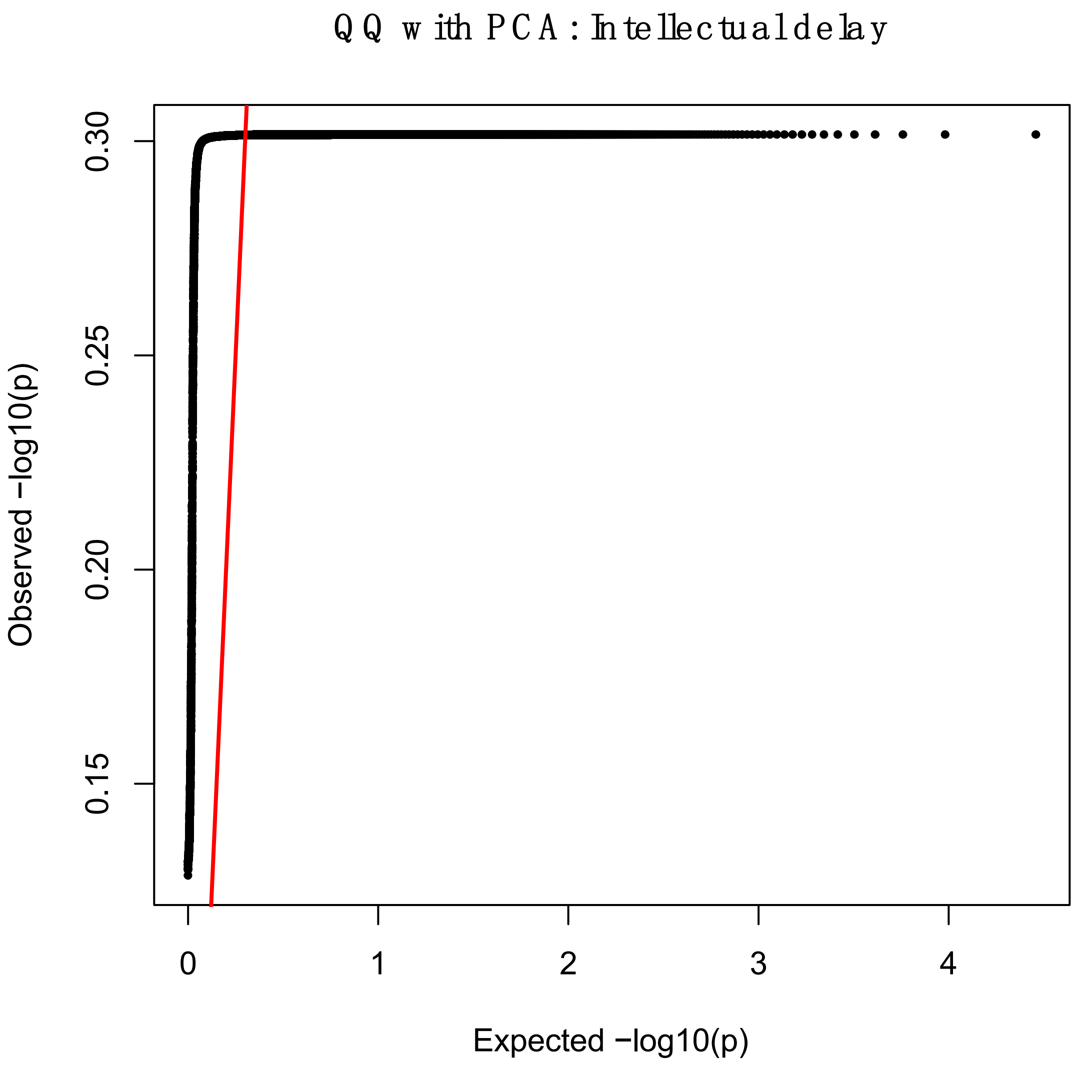
QQ with PCA: Intellectual delay
Observed −log10(p)
Expected −log10(p)
0.15
0.20
0.25
0.30
0
1
2
3
4

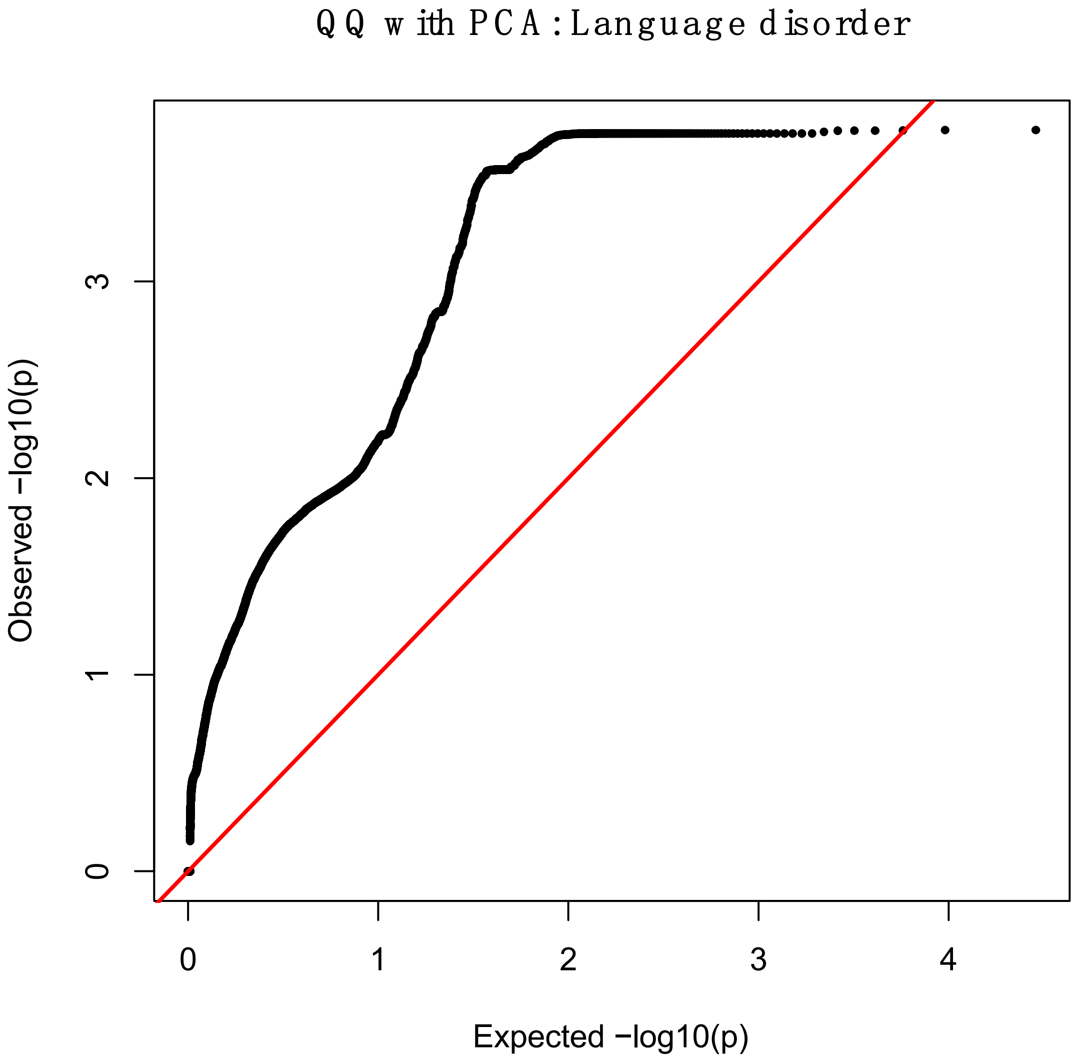

QQ with PCA: Language disorder
Observed −log10(p)
Expected −log10(p)
0
1
2
3
4

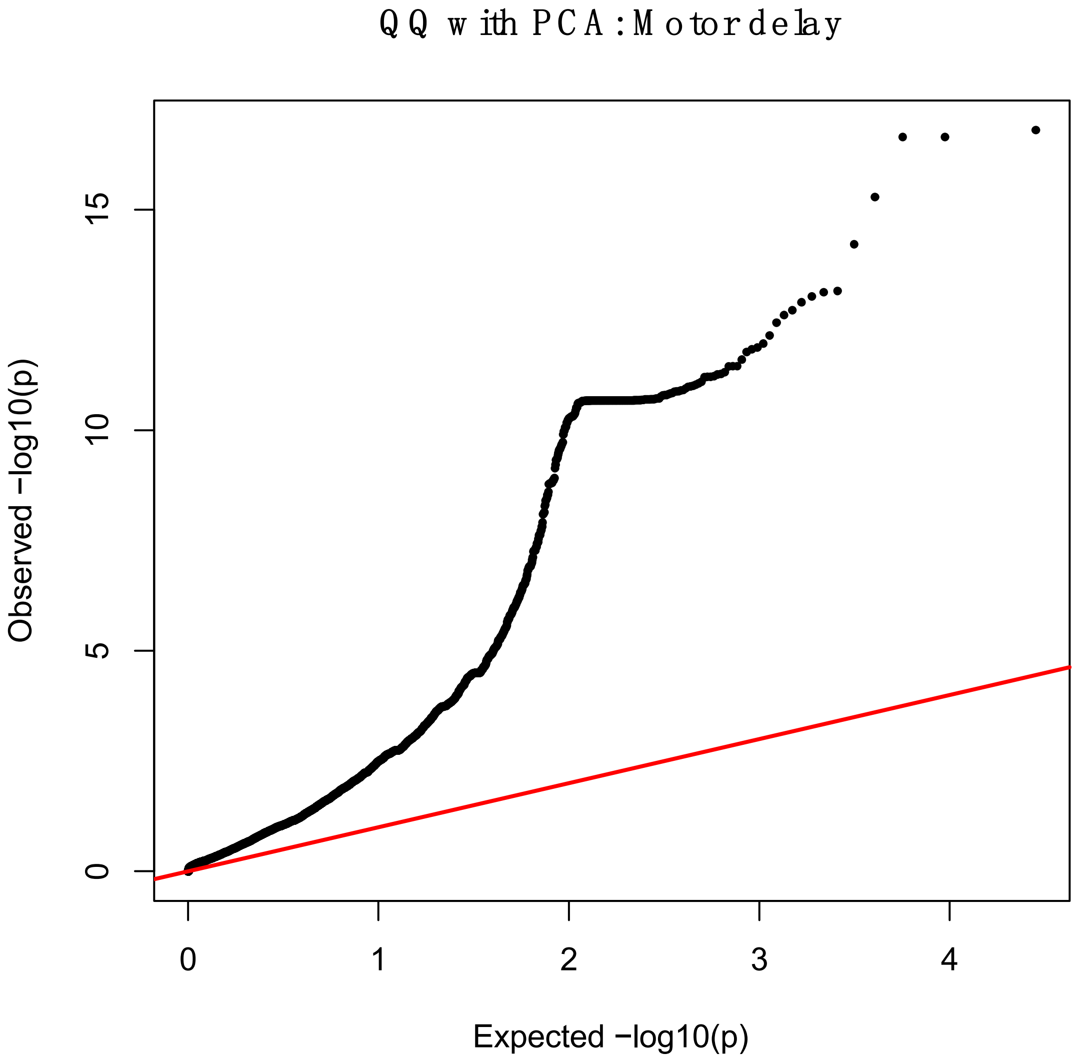

QQ with PCA:Motordelay
Observed −log10(p)
Expected −log10(p)
0
5
10
15
0
1
2
3
4

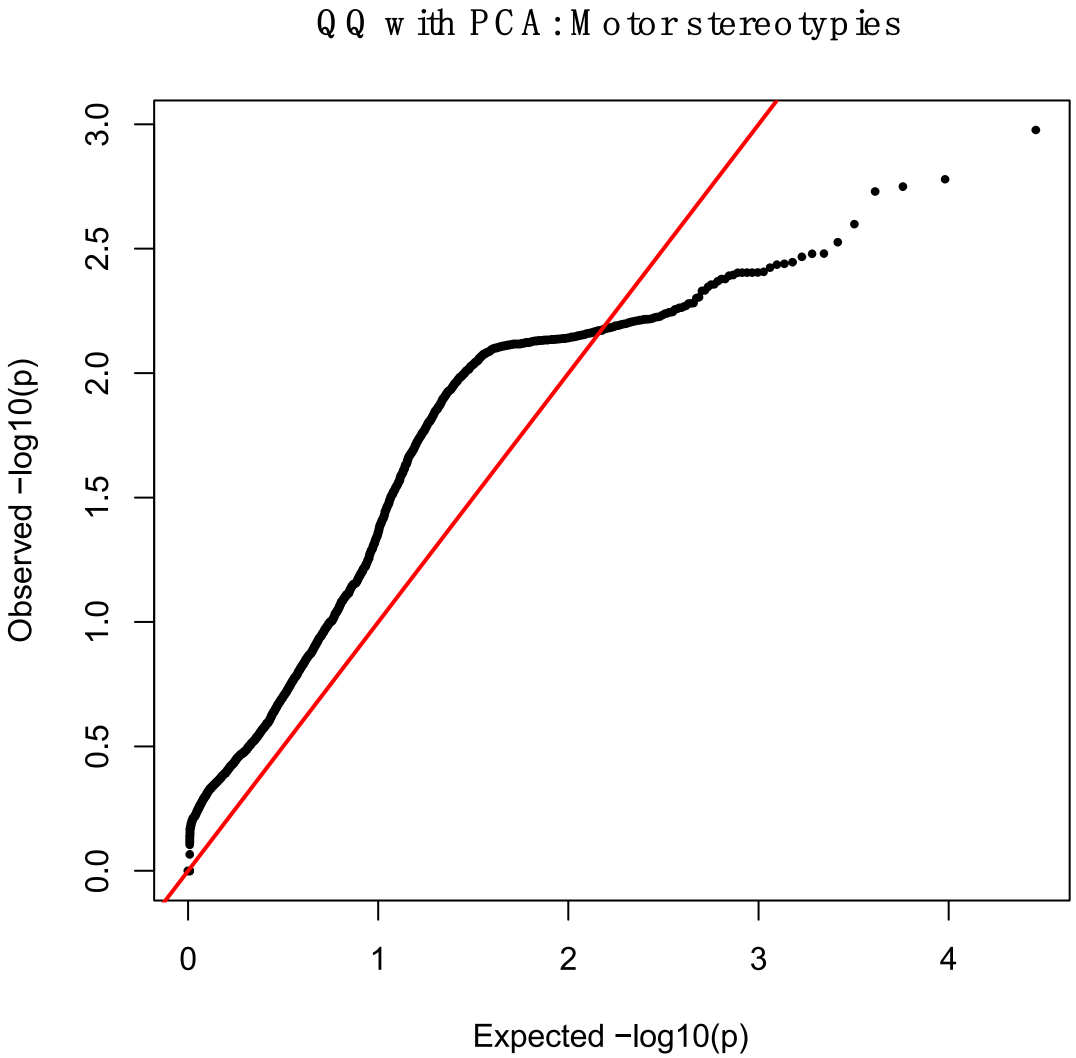

QQ with PCA: Motor stereotypies
Observed −log10(p)
Expected −log10(p)
0.0
0.5
1.0
1.5
2.0
2.5
3.0
0
1
2
3
4

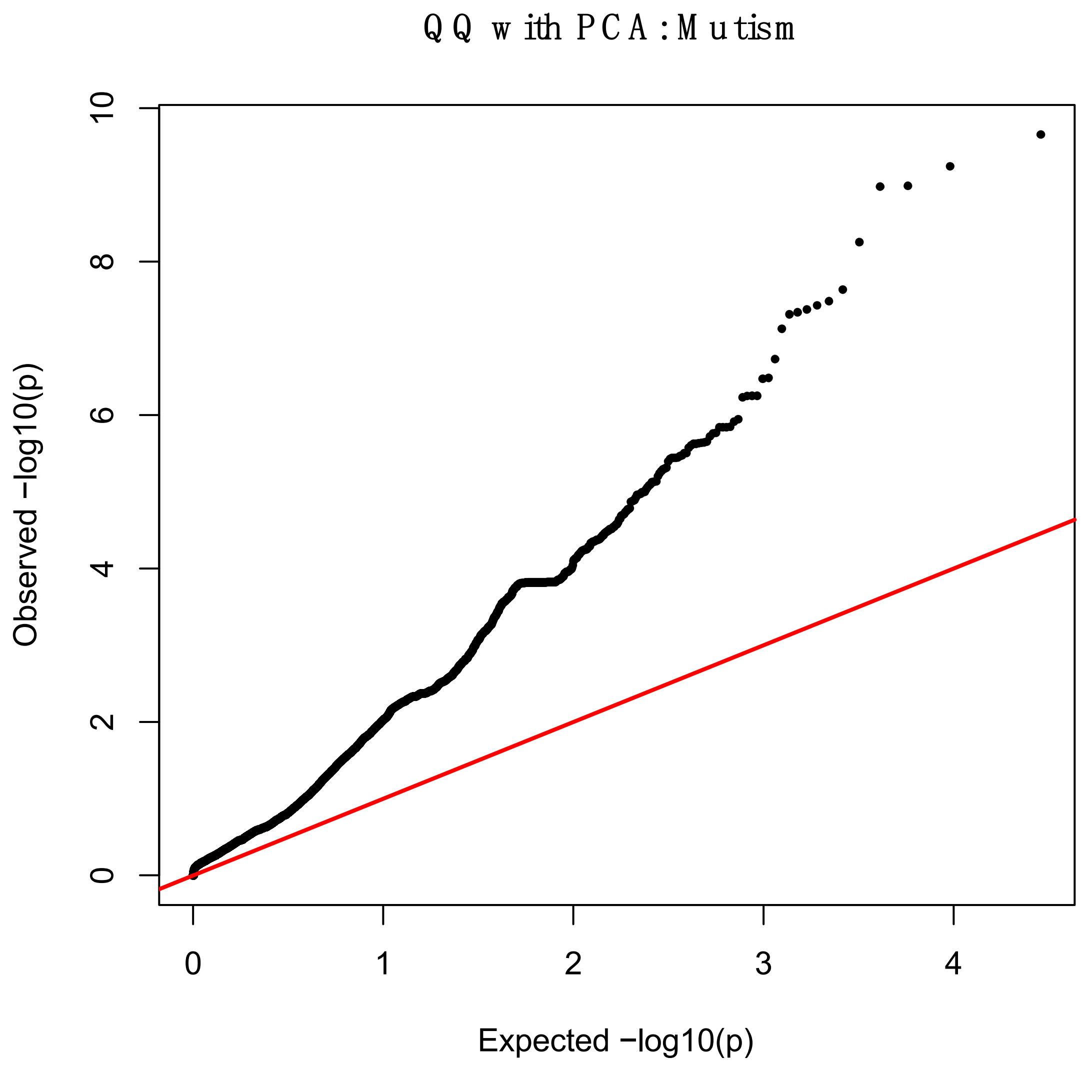
QQ with PCA: Mutism
Observed −log10(p)
Expected −log10(p)
0
2
4
6
8
10
0
1
2
3
4

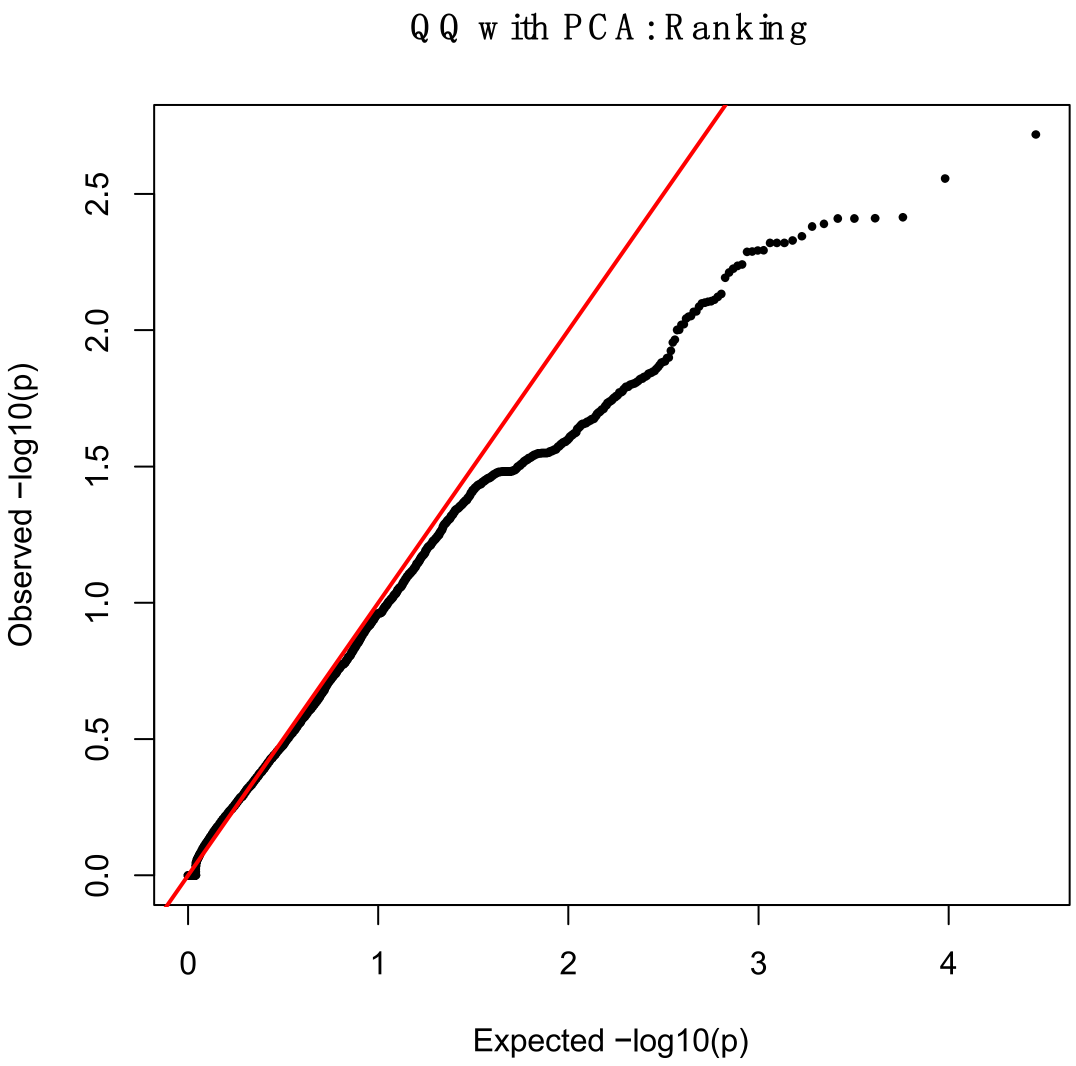

Q Q w ith P C A : R a n k in g
Observed −log10(p)
0.0
0.5
1.0
1.5
2.0
2.5
0
1
2
3
4
Expected −log10(p)

Q Q with PCA : Seizure

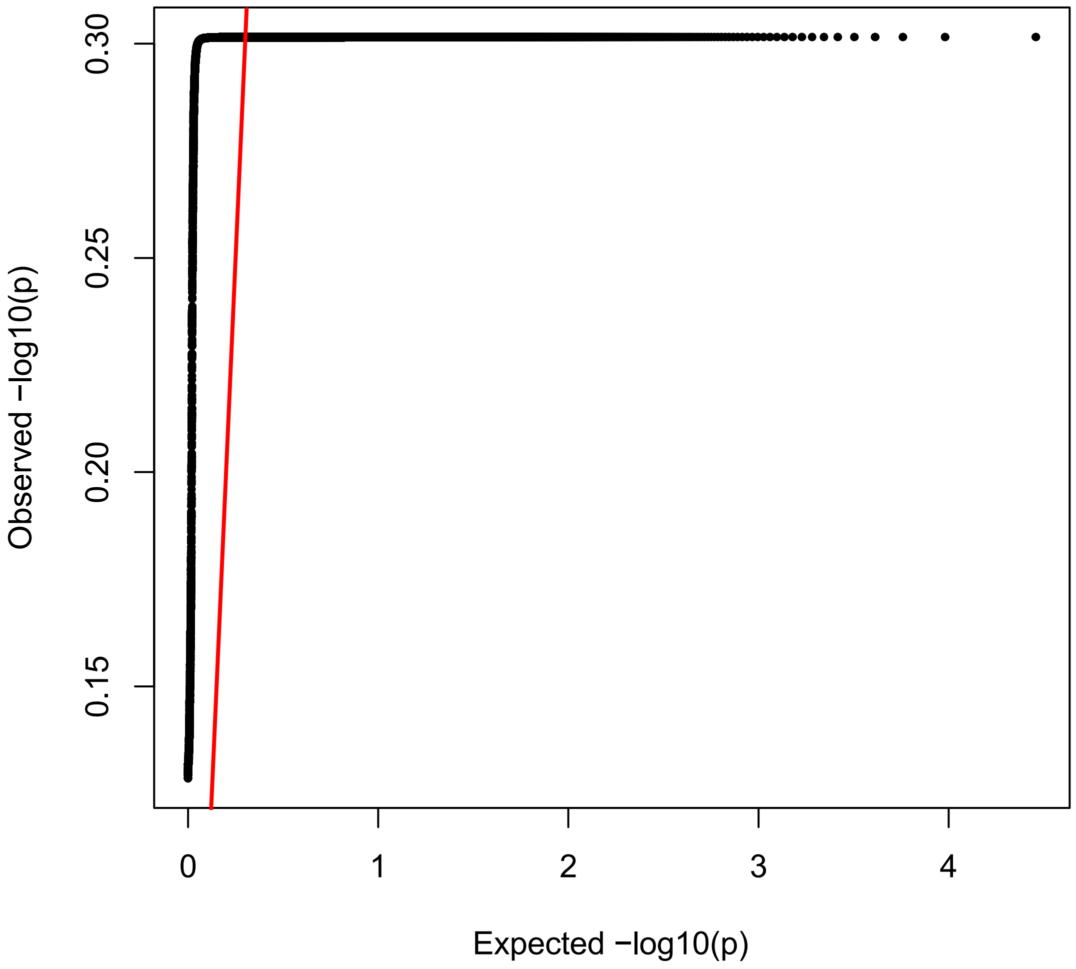

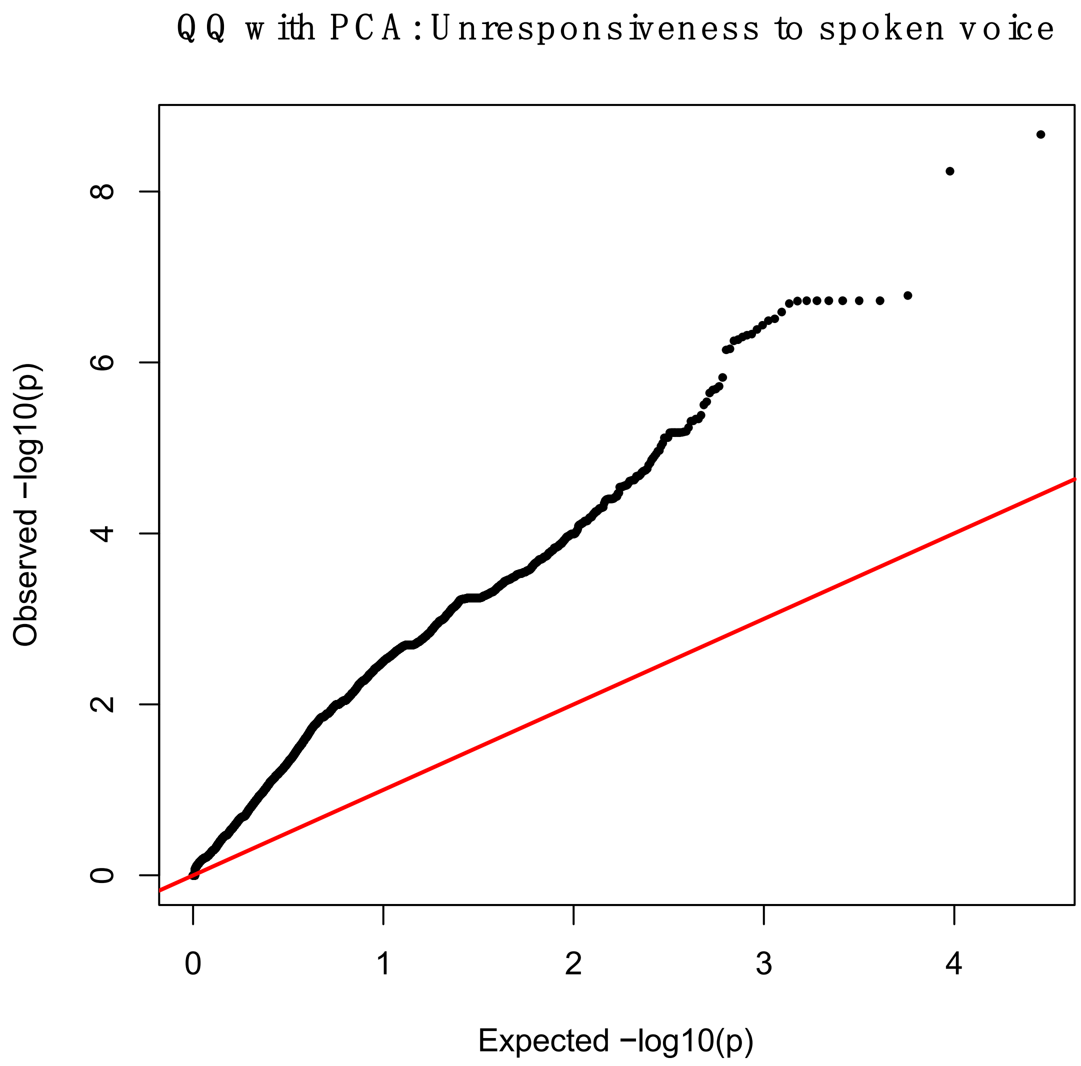

QQ with PCA: Unresponsiveness to spoken voice
Observed −log10(p)
Expected −log10(p)
0
2
4
6
8
0
1
2
3
4
