# Supplementary material for: Genetic Heterogeneity in Autism Spectrum Disorder: Diagnostic Yield, Recurrent Genes, and Rare Variant-Phenotype Associations from Whole-Exome Sequencing
Source: Int J Mol Sci. 2026 Aug 1;27(15):6901. doi: 10.3390/ijms27156901 (PMC13467236; doi:10.3390/ijms27156901)
Supplement: Supplementary file 1 [file ijms-27-06901-s001.zip › Table S4.pdf]

Table S4: Cross-phenotype sharing of BH-significant genes among ASD phenotypes

Gene	N_Phenotypes	Phenotypes
ASPN	5	Digestive disorder; Echolalia; Motor delay; Sleep disturbances; Unresponsiveness to spoken voice
IFNB1	5	Anxiety; Echolalia; Motor delay; Sleep disturbances; Unresponsiveness to spoken voice
NRXN2	5	Anxiety; Echolalia; Motor delay; Sleep disturbances; Unresponsiveness to spoken voice
OR3A4P	5	Digestive disorder; Echolalia; Motor delay; Sleep disturbances; Unresponsiveness to spoken voice
OR5H15	5	Anxiety; Echolalia; Motor delay; Sleep disturbances; Unresponsiveness to spoken voice
PHYH	5	Anxiety; Echolalia; Motor delay; Sleep disturbances; Unresponsiveness to spoken voice
RELA	5	Anxiety; Echolalia; Motor delay; Sleep disturbances; Unresponsiveness to spoken voice
SPANXD	5	Anxiety; Echolalia; Motor delay; Mutism; Sleep disturbances
ABRAXAS1	4	Anxiety; Echolalia; Motor delay; Unresponsiveness to spoken voice
AKR1B10	4	Anxiety; Echolalia; Mutism; Unresponsiveness to spoken voice
ANKRD34B	4	Anxiety; Echolalia; Mutism; Sleep disturbances
ANKRD66	4	Anxiety; Echolalia; Language disorder; Motor delay
APOBEC3H	4	Anxiety; Echolalia; Motor delay; Unresponsiveness to spoken voice
ARHGAP22	4	Aggressive behaviour; Echolalia; Sleep disturbances; Unresponsiveness to spoken voice
ASB8	4	Echolalia; Motor delay; Sleep disturbances; Unresponsiveness to spoken voice
ATAD3C	4	Anxiety; Echolalia; Motor delay; Sleep disturbances
AXDND1	4	Echolalia; Motor delay; Sleep disturbances; Unresponsiveness to spoken voice
BAHCC1	4	Echolalia; Motor delay; Mutism; Unresponsiveness to spoken voice
CD33	4	Aggressive behaviour; Anxiety; Echolalia; Unresponsiveness to spoken voice
CNOT11	4	Anxiety; Echolalia; Mutism; Sleep disturbances
CNR1	4	Digestive disorder; Echolalia; Motor delay; Unresponsiveness to spoken voice
CTTN	4	Anxiety; Echolalia; Mutism; Sleep disturbances
DACT1	4	Echolalia; Motor delay; Sleep disturbances; Unresponsiveness to spoken voice
DAO	4	Aggressive behaviour; Anxiety; Echolalia; Unresponsiveness to spoken voice
DCAF12	4	Echolalia; Motor delay; Sleep disturbances; Unresponsiveness to spoken voice
DCLK1	4	Digestive disorder; Echolalia; Motor delay; Sleep disturbances
DCTD	4	Anxiety; Echolalia; Mutism; Sleep disturbances
DNAAF5	4	Echolalia; Motor delay; Sleep disturbances; Unresponsiveness to spoken voice
DVL2	4	Anxiety; Echolalia; Motor delay; Unresponsiveness to spoken voice
EFEMP1	4	Anxiety; Echolalia; Motor delay; Unresponsiveness to spoken voice
EGR4	4	Anxiety; Echolalia; Mutism; Sleep disturbances
EHBP1L1	4	Anxiety; Echolalia; Motor delay; Unresponsiveness to spoken voice
ERICH1	4	Echolalia; Motor delay; Mutism; Unresponsiveness to spoken voice
FANCA	4	Aggressive behaviour; Echolalia; Language disorder; Unresponsiveness to spoken voice
FCRL2	4	Anxiety; Echolalia; Sleep disturbances; Unresponsiveness to spoken voice
FSTL5	4	Anxiety; Echolalia; Sleep disturbances; Unresponsiveness to spoken voice
FUBP1	4	Echolalia; Language disorder; Motor delay; Mutism
GBP3	4	Anxiety; Echolalia; Motor delay; Unresponsiveness to spoken voice
GBP5	4	Anxiety; Echolalia; Mutism; Sleep disturbances
GLB1L3	4	Anxiety; Echolalia; Mutism; Sleep disturbances
GLS2	4	Echolalia; Mutism; Sleep disturbances; Unresponsiveness to spoken voice
GSTM1	4	Echolalia; Motor delay; Sleep disturbances; Unresponsiveness to spoken voice
HSFX3	4	Anxiety; Echolalia; Mutism; Sleep disturbances

IGSF1	4	Anxiety; Echolalia; Mutism; Sleep disturbances
ITGA2	4	Anxiety; Echolalia; Motor delay; Unresponsiveness to spoken voice
KCTD8	4	Echolalia; Motor delay; Sleep disturbances; Unresponsiveness to spoken voice
KLC3	4	Anxiety; Echolalia; Motor delay; Sleep disturbances
LACC1	4	Anxiety; Echolalia; Language disorder; Motor delay
LAMB4	4	Aggressive behaviour; Anxiety; Echolalia; Unresponsiveness to spoken voice
LOC105371031	4	Anxiety; Echolalia; Motor delay; Unresponsiveness to spoken voice
MAN1B1	4	Echolalia; Motor delay; Sleep disturbances; Unresponsiveness to spoken voice
MAN2C1	4	Anxiety; Echolalia; Mutism; Sleep disturbances
MIEF1	4	Anxiety; Echolalia; Motor delay; Unresponsiveness to spoken voice
MIR5195	4	Echolalia; Language disorder; Motor delay; Unresponsiveness to spoken voice
MMRN1	4	Anxiety; Echolalia; Motor delay; Unresponsiveness to spoken voice
MST1R	4	Aggressive behaviour; Anxiety; Echolalia; Unresponsiveness to spoken voice
NOS2	4	Anxiety; Echolalia; Mutism; Unresponsiveness to spoken voice
NUSAP1	4	Echolalia; Motor delay; Sleep disturbances; Unresponsiveness to spoken voice
OR13C9	4	Echolalia; Motor delay; Sleep disturbances; Unresponsiveness to spoken voice
OR1D2	4	Echolalia; Motor delay; Sleep disturbances; Unresponsiveness to spoken voice
OXSM	4	Echolalia; Motor delay; Sleep disturbances; Unresponsiveness to spoken voice
P2RY6	4	Anxiety; Echolalia; Motor delay; Unresponsiveness to spoken voice
PDE9A	4	Anxiety; Echolalia; Mutism; Unresponsiveness to spoken voice
PLXDC2	4	Aggressive behaviour; Anxiety; Echolalia; Unresponsiveness to spoken voice
PTPN21	4	Aggressive behaviour; Anxiety; Echolalia; Unresponsiveness to spoken voice
RANBP10	4	Echolalia; Motor delay; Sleep disturbances; Unresponsiveness to spoken voice
RGR	4	Anxiety; Echolalia; Mutism; Sleep disturbances
RRP15	4	Aggressive behaviour; Anxiety; Echolalia; Unresponsiveness to spoken voice
RTKN2	4	Echolalia; Language disorder; Motor delay; Mutism
SCN3A	4	Echolalia; Motor delay; Mutism; Sleep disturbances
SEC16A	4	Anxiety; Echolalia; Motor delay; Unresponsiveness to spoken voice
SLC22A24	4	Anxiety; Echolalia; Motor delay; Unresponsiveness to spoken voice
SLC34A2	4	Anxiety; Echolalia; Motor delay; Sleep disturbances
SMC5	4	Echolalia; Motor delay; Sleep disturbances; Unresponsiveness to spoken voice
SMG9	4	Anxiety; Echolalia; Motor delay; Sleep disturbances
SPINK2	4	Aggressive behaviour; Echolalia; Motor delay; Mutism
SSTR1	4	Echolalia; Motor delay; Sleep disturbances; Unresponsiveness to spoken voice
STK39	4	Anxiety; Echolalia; Language disorder; Motor delay
TBX6	4	Anxiety; Motor delay; Sleep disturbances; Unresponsiveness to spoken voice
TBXAS1	4	Echolalia; Motor delay; Sleep disturbances; Unresponsiveness to spoken voice
TLE6	4	Anxiety; Echolalia; Mutism; Sleep disturbances
TMEM170A	4	Echolalia; Language disorder; Motor delay; Sleep disturbances
TMTC1	4	Echolalia; Motor delay; Mutism; Unresponsiveness to spoken voice
TOR3A	4	Anxiety; Echolalia; Mutism; Sleep disturbances
TREML2	4	Echolalia; Motor delay; Mutism; Unresponsiveness to spoken voice
TRIM37	4	Anxiety; Echolalia; Motor delay; Mutism
TSHZ2	4	Anxiety; Echolalia; Mutism; Sleep disturbances
UQCRHL	4	Echolalia; Motor delay; Sleep disturbances; Unresponsiveness to spoken voice
USP6	4	Echolalia; Motor delay; Sleep disturbances; Unresponsiveness to spoken voice

VPS51	4	Anxiety; Motor delay; Sleep disturbances; Unresponsiveness to spoken voice
ZNF226	4	Aggressive behaviour; Anxiety; Echolalia; Unresponsiveness to spoken voice
ZNF430	4	Anxiety; Echolalia; Mutism; Sleep disturbances
ZNF787	4	Anxiety; Echolalia; Motor delay; Unresponsiveness to spoken voice
ZUP1	4	Echolalia; Motor delay; Sleep disturbances; Unresponsiveness to spoken voice
A2ML1	3	Echolalia; Sleep disturbances; Unresponsiveness to spoken voice
A4GNT	3	Aggressive behaviour; Echolalia; Mutism
AADACL3	3	Anxiety; Echolalia; Unresponsiveness to spoken voice
ABCC12	3	Echolalia; Sleep disturbances; Unresponsiveness to spoken voice
ABCD2	3	Anxiety; Echolalia; Mutism
ABCF3	3	Anxiety; Echolalia; Unresponsiveness to spoken voice
ABHD14A-ACY1	3	Echolalia; Mutism; Sleep disturbances
ABHD14B	3	Anxiety; Echolalia; Mutism
ABHD17A	3	Anxiety; Echolalia; Unresponsiveness to spoken voice
ABLIM2	3	Echolalia; Language disorder; Motor delay
ACAD11	3	Echolalia; Language disorder; Motor delay
ACCSL	3	Anxiety; Echolalia; Unresponsiveness to spoken voice
ACP5	3	Anxiety; Echolalia; Mutism
ACRBP	3	Echolalia; Motor delay; Unresponsiveness to spoken voice
ACSF3	3	Echolalia; Language disorder; Unresponsiveness to spoken voice
ACSM6	3	Echolalia; Language disorder; Motor delay
ACTG1	3	Anxiety; Echolalia; Mutism
ACTR6	3	Aggressive behaviour; Echolalia; Unresponsiveness to spoken voice
ACTRT2	3	Echolalia; Motor delay; Unresponsiveness to spoken voice
ADAD1	3	Echolalia; Motor delay; Unresponsiveness to spoken voice
ADAMTS6	3	Echolalia; Motor delay; Unresponsiveness to spoken voice
ADAMTS8	3	Echolalia; Language disorder; Motor delay
ADCYAP1	3	Echolalia; Motor delay; Unresponsiveness to spoken voice
ADGRA2	3	Anxiety; Echolalia; Unresponsiveness to spoken voice
ADGRF4	3	Anxiety; Echolalia; Mutism
ADH4	3	Echolalia; Sleep disturbances; Unresponsiveness to spoken voice
ADH7	3	Aggressive behaviour; Anxiety; Echolalia
ADK	3	Echolalia; Motor delay; Unresponsiveness to spoken voice
ADM	3	Echolalia; Motor delay; Unresponsiveness to spoken voice
ADRB2	3	Echolalia; Language disorder; Motor delay
ADRB3	3	Echolalia; Motor delay; Unresponsiveness to spoken voice
AFG3L2	3	Echolalia; Language disorder; Motor delay
AGAP3	3	Echolalia; Motor delay; Unresponsiveness to spoken voice
AGMO	3	Anxiety; Echolalia; Unresponsiveness to spoken voice
AHSA1	3	Anxiety; Echolalia; Mutism
AHSP	3	Echolalia; Motor delay; Unresponsiveness to spoken voice
AIF1L	3	Echolalia; Language disorder; Motor delay
AIFM3	3	Echolalia; Motor delay; Unresponsiveness to spoken voice
AKR7A3	3	Echolalia; Mutism; Unresponsiveness to spoken voice
ALDH18A1	3	Aggressive behaviour; Anxiety; Echolalia
ALG1L2	3	Echolalia; Language disorder; Motor delay

ALG6	3	Echolalia; Motor delay; Unresponsiveness to spoken voice
ALKBH4	3	Anxiety; Echolalia; Unresponsiveness to spoken voice
AMBN	3	Echolalia; Language disorder; Motor delay
AMDHD1	3	Echolalia; Motor delay; Unresponsiveness to spoken voice
AMT	3	Anxiety; Echolalia; Mutism
ANKDD1A	3	Echolalia; Language disorder; Motor delay
ANKRD11	3	Echolalia; Language disorder; Mutism
ANKRD31	3	Echolalia; Motor delay; Unresponsiveness to spoken voice
ANKRD33B	3	Anxiety; Echolalia; Language disorder
ANKRD50	3	Echolalia; Language disorder; Motor delay
ANLN	3	Echolalia; Mutism; Sleep disturbances
ANO2	3	Anxiety; Echolalia; Motor delay
ANO3	3	Aggressive behaviour; Echolalia; Unresponsiveness to spoken voice
ANTKMT	3	Anxiety; Echolalia; Mutism
ANXA8L1	3	Anxiety; Echolalia; Unresponsiveness to spoken voice
AOC2	3	Aggressive behaviour; Echolalia; Unresponsiveness to spoken voice
APBB2	3	Anxiety; Echolalia; Mutism
APOL4	3	Anxiety; Echolalia; Unresponsiveness to spoken voice
APTX	3	Aggressive behaviour; Echolalia; Unresponsiveness to spoken voice
ARHGAP25	3	Echolalia; Language disorder; Motor delay
ARHGAP31	3	Echolalia; Language disorder; Motor delay
ARHGAP35	3	Aggressive behaviour; Echolalia; Motor delay
ARHGAP4	3	Anxiety; Echolalia; Unresponsiveness to spoken voice
ARHGEF10L	3	Echolalia; Motor delay; Unresponsiveness to spoken voice
ARHGEF16	3	Echolalia; Motor delay; Unresponsiveness to spoken voice
ARHGEF19	3	Anxiety; Echolalia; Mutism
ARHGEF38	3	Anxiety; Echolalia; Mutism
ARHGEF7	3	Anxiety; Echolalia; Sleep disturbances
ARL13B	3	Echolalia; Motor delay; Unresponsiveness to spoken voice
ARMC12	3	Aggressive behaviour; Echolalia; Unresponsiveness to spoken voice
ARMC3	3	Echolalia; Language disorder; Motor delay
ARMS2	3	Echolalia; Mutism; Sleep disturbances
ARRB1	3	Anxiety; Echolalia; Unresponsiveness to spoken voice
ASAP3	3	Echolalia; Language disorder; Motor delay
ASB5	3	Echolalia; Motor delay; Unresponsiveness to spoken voice
ASCC2	3	Echolalia; Motor delay; Mutism
ASPHD2	3	Echolalia; Motor delay; Unresponsiveness to spoken voice
ASPM	3	Echolalia; Language disorder; Motor delay
ATG7	3	Echolalia; Language disorder; Motor delay
ATP10D	3	Echolalia; Language disorder; Motor delay
ATP2A2	3	Aggressive behaviour; Echolalia; Motor delay
ATP2B2	3	Anxiety; Echolalia; Mutism
ATP4B	3	Anxiety; Echolalia; Mutism
ATP5F1A	3	Anxiety; Echolalia; Mutism
ATP6AP1	3	Echolalia; Language disorder; Motor delay
ATP6V1B2	3	Echolalia; Language disorder; Motor delay

ATP8B1	3	Aggressive behaviour; Echolalia; Unresponsiveness to spoken voice
ATP8B2	3	Anxiety; Echolalia; Unresponsiveness to spoken voice
AVPI1	3	Anxiety; Echolalia; Mutism
AWAT1	3	Echolalia; Motor delay; Unresponsiveness to spoken voice
B3GAT1	3	Aggressive behaviour; Anxiety; Echolalia
B3GNT4	3	Echolalia; Language disorder; Motor delay
B4GALNT1	3	Echolalia; Language disorder; Unresponsiveness to spoken voice
BANF2	3	Anxiety; Echolalia; Unresponsiveness to spoken voice
BATF3	3	Echolalia; Motor delay; Unresponsiveness to spoken voice
BBS2	3	Digestive disorder; Echolalia; Unresponsiveness to spoken voice
BCL2L2	3	Echolalia; Language disorder; Motor delay
BCL2L2-PABPN1	3	Echolalia; Language disorder; Motor delay
BICRA	3	Anxiety; Echolalia; Unresponsiveness to spoken voice
BIRC5	3	Aggressive behaviour; Anxiety; Echolalia
BIVM	3	Echolalia; Motor delay; Unresponsiveness to spoken voice
BNIP3	3	Anxiety; Echolalia; Unresponsiveness to spoken voice
BOD1L2	3	Anxiety; Echolalia; Mutism
BRD4	3	Anxiety; Echolalia; Mutism
BRIP1	3	Anxiety; Echolalia; Unresponsiveness to spoken voice
BSND	3	Echolalia; Sleep disturbances; Unresponsiveness to spoken voice
BSPRY	3	Echolalia; Language disorder; Motor delay
BTBD17	3	Echolalia; Motor delay; Unresponsiveness to spoken voice
BTBD6	3	Anxiety; Echolalia; Mutism
BUD13	3	Anxiety; Echolalia; Mutism
C11orf1	3	Echolalia; Language disorder; Motor delay
C11orf16	3	Echolalia; Language disorder; Motor delay
C11orf49	3	Anxiety; Echolalia; Unresponsiveness to spoken voice
C11orf53	3	Echolalia; Sleep disturbances; Unresponsiveness to spoken voice
C11orf95	3	Echolalia; Motor delay; Unresponsiveness to spoken voice
C12orf54	3	Anxiety; Echolalia; Mutism
C12orf71	3	Echolalia; Language disorder; Motor delay
C15orf39	3	Anxiety; Echolalia; Unresponsiveness to spoken voice
C17orf49	3	Echolalia; Language disorder; Motor delay
C19orf53	3	Anxiety; Echolalia; Unresponsiveness to spoken voice
C1GALT1C1L	3	Echolalia; Language disorder; Mutism
C1QTNF1	3	Aggressive behaviour; Echolalia; Unresponsiveness to spoken voice
C1orf109	3	Aggressive behaviour; Echolalia; Unresponsiveness to spoken voice
C1orf158	3	Anxiety; Echolalia; Unresponsiveness to spoken voice
C20orf173	3	Echolalia; Language disorder; Unresponsiveness to spoken voice
C2CD3	3	Anxiety; Echolalia; Mutism
C2CD4C	3	Anxiety; Echolalia; Mutism
C2CD4D	3	Aggressive behaviour; Echolalia; Unresponsiveness to spoken voice
C3orf62	3	Aggressive behaviour; Echolalia; Unresponsiveness to spoken voice
C7orf77	3	Anxiety; Echolalia; Mutism
C8orf33	3	Anxiety; Echolalia; Mutism
C9orf129	3	Echolalia; Language disorder; Motor delay

C9orf50	3	Echolalia; Language disorder; Motor delay
C9orf78	3	Anxiety; Echolalia; Unresponsiveness to spoken voice
C9orf92	3	Echolalia; Motor delay; Unresponsiveness to spoken voice
CABP5	3	Echolalia; Language disorder; Motor delay
CABS1	3	Aggressive behaviour; Echolalia; Motor delay
CACNA1C	3	Anxiety; Echolalia; Mutism
CACNB1	3	Anxiety; Echolalia; Unresponsiveness to spoken voice
CACNB4	3	Echolalia; Language disorder; Motor delay
CAGE1	3	Echolalia; Language disorder; Unresponsiveness to spoken voice
CALCR	3	Echolalia; Motor delay; Unresponsiveness to spoken voice
CAPN6	3	Anxiety; Echolalia; Unresponsiveness to spoken voice
CASP8AP2	3	Anxiety; Echolalia; Sleep disturbances
CASP9	3	Echolalia; Language disorder; Motor delay
CASZ1	3	Anxiety; Echolalia; Mutism
CATSPER3	3	Echolalia; Motor delay; Unresponsiveness to spoken voice
CATSPERG	3	Anxiety; Echolalia; Mutism
CBY2	3	Anxiety; Echolalia; Motor delay
CC2D2A	3	Echolalia; Motor delay; Unresponsiveness to spoken voice
CCBE1	3	Anxiety; Echolalia; Mutism
CCDC102A	3	Echolalia; Language disorder; Motor delay
CCDC115	3	Echolalia; Sleep disturbances; Unresponsiveness to spoken voice
CCDC121	3	Echolalia; Motor delay; Unresponsiveness to spoken voice
CCDC138	3	Echolalia; Language disorder; Motor delay
CCDC148	3	Anxiety; Echolalia; Mutism
CCDC15	3	Echolalia; Motor delay; Unresponsiveness to spoken voice
CCDC191	3	Echolalia; Language disorder; Motor delay
CCDC59	3	Anxiety; Echolalia; Motor delay
CCDC61	3	Aggressive behaviour; Echolalia; Motor delay
CCDC70	3	Anxiety; Sleep disturbances; Unresponsiveness to spoken voice
CCDC71L	3	Anxiety; Echolalia; Mutism
CCDC87	3	Anxiety; Echolalia; Mutism
CCNL1	3	Echolalia; Motor delay; Unresponsiveness to spoken voice
CCR2	3	Aggressive behaviour; Echolalia; Unresponsiveness to spoken voice
CD164L2	3	Anxiety; Echolalia; Unresponsiveness to spoken voice
CD1B	3	Echolalia; Motor delay; Sleep disturbances
CD248	3	Echolalia; Sleep disturbances; Unresponsiveness to spoken voice
CD27	3	Anxiety; Echolalia; Mutism
CD300LB	3	Echolalia; Sleep disturbances; Unresponsiveness to spoken voice
CD38	3	Aggressive behaviour; Echolalia; Unresponsiveness to spoken voice
CD55	3	Echolalia; Motor delay; Mutism
CDC42BPB	3	Echolalia; Language disorder; Motor delay
CDC42BPG	3	Echolalia; Language disorder; Motor delay
CDC42EP4	3	Anxiety; Echolalia; Mutism
CDH1	3	Echolalia; Motor delay; Unresponsiveness to spoken voice
CDH12	3	Anxiety; Echolalia; Mutism
CDH20	3	Aggressive behaviour; Echolalia; Motor delay

CDKAL1	3	Anxiety; Echolalia; Unresponsiveness to spoken voice
CDYL	3	Echolalia; Mutism; Sleep disturbances
CEACAM18	3	Echolalia; Language disorder; Motor delay
CEACAM6	3	Anxiety; Echolalia; Unresponsiveness to spoken voice
CELA3A	3	Echolalia; Motor delay; Unresponsiveness to spoken voice
CELA3B	3	Anxiety; Echolalia; Unresponsiveness to spoken voice
CENPE	3	Echolalia; Motor delay; Sleep disturbances
CENPF	3	Anxiety; Echolalia; Unresponsiveness to spoken voice
CENPP	3	Echolalia; Motor delay; Unresponsiveness to spoken voice
CEP57L1	3	Aggressive behaviour; Echolalia; Motor delay
CERS4	3	Anxiety; Echolalia; Mutism
CETN1	3	Anxiety; Echolalia; Unresponsiveness to spoken voice
CFH	3	Anxiety; Echolalia; Mutism
CFHR2	3	Echolalia; Sleep disturbances; Unresponsiveness to spoken voice
CFHR5	3	Echolalia; Language disorder; Motor delay
CFP	3	Echolalia; Motor delay; Unresponsiveness to spoken voice
CGB7	3	Echolalia; Mutism; Unresponsiveness to spoken voice
CHAD	3	Anxiety; Echolalia; Mutism
CHAT	3	Echolalia; Mutism; Unresponsiveness to spoken voice
CHM	3	Echolalia; Motor delay; Unresponsiveness to spoken voice
CHRM1	3	Echolalia; Language disorder; Mutism
CHRNB2	3	Echolalia; Motor delay; Unresponsiveness to spoken voice
CHST5	3	Echolalia; Motor delay; Unresponsiveness to spoken voice
CIITA	3	Aggressive behaviour; Echolalia; Unresponsiveness to spoken voice
CILK1	3	Echolalia; Language disorder; Motor delay
CKAP2	3	Echolalia; Language disorder; Motor delay
CKM	3	Echolalia; Language disorder; Motor delay
CLCNKB	3	Anxiety; Echolalia; Sleep disturbances
CLDN24	3	Echolalia; Language disorder; Motor delay
CLDND2	3	Aggressive behaviour; Anxiety; Echolalia
CLEC4E	3	Echolalia; Motor delay; Unresponsiveness to spoken voice
CLEC4G	3	Echolalia; Sleep disturbances; Unresponsiveness to spoken voice
CLIC6	3	Anxiety; Echolalia; Mutism
CLN3	3	Echolalia; Motor delay; Unresponsiveness to spoken voice
1 CLP	3	Echolalia; Sleep disturbances; Unresponsiveness to spoken voice
CLRN1	3	Echolalia; Motor delay; Unresponsiveness to spoken voice
CLRN2	3	Echolalia; Mutism; Unresponsiveness to spoken voice
CLUH	3	Anxiety; Echolalia; Mutism
CMAS	3	Anxiety; Echolalia; Mutism
CMTR1	3	Aggressive behaviour; Echolalia; Unresponsiveness to spoken voice
CNBD2	3	Aggressive behaviour; Echolalia; Unresponsiveness to spoken voice
CNDP2	3	Echolalia; Language disorder; Motor delay
CNGB3	3	Anxiety; Echolalia; Mutism
CNPY3	3	Anxiety; Echolalia; Mutism
CNST	3	Anxiety; Echolalia; Mutism
CNTN2	3	Anxiety; Echolalia; Language disorder

CNTNAP2	3	Echolalia; Sleep disturbances; Unresponsiveness to spoken voice
CNTNAP4	3	Echolalia; Mutism; Unresponsiveness to spoken voice
CNTRL	3	Echolalia; Language disorder; Motor delay
COL11A1	3	Echolalia; Language disorder; Motor delay
COL4A3	3	Echolalia; Language disorder; Motor delay
COL8A2	3	Echolalia; Language disorder; Motor delay
COPE	3	Anxiety; Echolalia; Unresponsiveness to spoken voice
COQ6	3	Echolalia; Language disorder; Motor delay
COX18	3	Echolalia; Motor delay; Unresponsiveness to spoken voice
CPNE4	3	Echolalia; Motor delay; Unresponsiveness to spoken voice
CPOX	3	Echolalia; Motor delay; Unresponsiveness to spoken voice
CPSF4	3	Echolalia; Sleep disturbances; Unresponsiveness to spoken voice
CPSF6	3	Anxiety; Echolalia; Unresponsiveness to spoken voice
CPT2	3	Echolalia; Sleep disturbances; Unresponsiveness to spoken voice
CR2	3	Echolalia; Language disorder; Motor delay
CRCT1	3	Echolalia; Motor delay; Unresponsiveness to spoken voice
CRK	3	Echolalia; Motor delay; Unresponsiveness to spoken voice
CROCC2	3	Anxiety; Echolalia; Mutism
CRTAM	3	Anxiety; Echolalia; Language disorder
CRYGB	3	Echolalia; Sleep disturbances; Unresponsiveness to spoken voice
CRYZ	3	Echolalia; Language disorder; Motor delay
CRYZL1	3	Aggressive behaviour; Echolalia; Unresponsiveness to spoken voice
CSF3	3	Echolalia; Motor delay; Unresponsiveness to spoken voice
CSH1	3	Echolalia; Motor delay; Unresponsiveness to spoken voice
CSNK2A3	3	Aggressive behaviour; Echolalia; Unresponsiveness to spoken voice
CST3	3	Anxiety; Echolalia; Sleep disturbances
CTAGE4	3	Echolalia; Language disorder; Motor delay
CTAGE6	3	Echolalia; Language disorder; Motor delay
CTAGE8	3	Echolalia; Language disorder; Motor delay
CTDSP1	3	Echolalia; Language disorder; Motor delay
CTHRC1	3	Digestive disorder; Echolalia; Unresponsiveness to spoken voice
CTNNB1	3	Echolalia; Motor delay; Unresponsiveness to spoken voice
CUL3	3	Aggressive behaviour; Echolalia; Motor delay
CUX1	3	Echolalia; Language disorder; Motor delay
CXCL16	3	Anxiety; Echolalia; Unresponsiveness to spoken voice
CYB5D1	3	Echolalia; Language disorder; Motor delay
CYBC1	3	Anxiety; Echolalia; Unresponsiveness to spoken voice
CYLC2	3	Digestive disorder; Echolalia; Sleep disturbances
CYP1A2	3	Anxiety; Echolalia; Sleep disturbances
CYP4F2	3	Echolalia; Sleep disturbances; Unresponsiveness to spoken voice
CYP4V2	3	Echolalia; Motor delay; Unresponsiveness to spoken voice
DAGLA	3	Anxiety; Echolalia; Unresponsiveness to spoken voice
DAOA	3	Echolalia; Motor delay; Unresponsiveness to spoken voice
DAPK2	3	Anxiety; Echolalia; Mutism
DAPL1	3	Anxiety; Echolalia; Unresponsiveness to spoken voice
DBP	3	Aggressive behaviour; Echolalia; Motor delay

DBR1	3	Echolalia; Language disorder; Motor delay
DBT	3	Anxiety; Mutism; Sleep disturbances
DBX1	3	Aggressive behaviour; Echolalia; Unresponsiveness to spoken voice
DCAF11	3	Anxiety; Echolalia; Mutism
DCAF5	3	Echolalia; Motor delay; Unresponsiveness to spoken voice
DCXR	3	Echolalia; Motor delay; Unresponsiveness to spoken voice
DDAH1	3	Echolalia; Sleep disturbances; Unresponsiveness to spoken voice
DDR2	3	Echolalia; Language disorder; Motor delay
DDRGK1	3	Echolalia; Motor delay; Unresponsiveness to spoken voice
DDX18	3	Anxiety; Echolalia; Unresponsiveness to spoken voice
DDX28	3	Digestive disorder; Echolalia; Sleep disturbances
DDX41	3	Echolalia; Motor delay; Unresponsiveness to spoken voice
DDX42	3	Anxiety; Echolalia; Unresponsiveness to spoken voice
DDX47	3	Echolalia; Sleep disturbances; Unresponsiveness to spoken voice
DDX59	3	Aggressive behaviour; Echolalia; Unresponsiveness to spoken voice
DEFA5	3	Echolalia; Language disorder; Motor delay
DEFB131A	3	Echolalia; Language disorder; Motor delay
DEFB136	3	Echolalia; Sleep disturbances; Unresponsiveness to spoken voice
DENND5B	3	Echolalia; Language disorder; Motor delay
DET1	3	Aggressive behaviour; Echolalia; Motor delay
DHRS7	3	Echolalia; Motor delay; Unresponsiveness to spoken voice
DHX32	3	Echolalia; Sleep disturbances; Unresponsiveness to spoken voice
DHX9	3	Echolalia; Language disorder; Motor delay
DIP2C	3	Echolalia; Language disorder; Unresponsiveness to spoken voice
4,00 DKK	3	Aggressive behaviour; Echolalia; Unresponsiveness to spoken voice
DKKL1	3	Echolalia; Language disorder; Motor delay
DLEU7	3	Echolalia; Language disorder; Motor delay
DMPK	3	Echolalia; Motor delay; Unresponsiveness to spoken voice
DMXL1	3	Anxiety; Echolalia; Mutism
DNAJB3	3	Echolalia; Language disorder; Motor delay
DNAJB4	3	Echolalia; Motor delay; Unresponsiveness to spoken voice
DNAJB9	3	Echolalia; Sleep disturbances; Unresponsiveness to spoken voice
DNAJC1	3	Anxiety; Echolalia; Mutism
DNAJC12	3	Anxiety; Echolalia; Mutism
DNAJC17	3	Echolalia; Motor delay; Unresponsiveness to spoken voice
DNMT1	3	Aggressive behaviour; Echolalia; Unresponsiveness to spoken voice
DOCK1	3	Aggressive behaviour; Echolalia; Unresponsiveness to spoken voice
DOCK6	3	Aggressive behaviour; Echolalia; Unresponsiveness to spoken voice
DOHH	3	Anxiety; Echolalia; Unresponsiveness to spoken voice
DOP1A	3	Anxiety; Mutism; Sleep disturbances
DPEP2	3	Echolalia; Sleep disturbances; Unresponsiveness to spoken voice
DPPA4	3	Anxiety; Echolalia; Mutism
DRICH1	3	Echolalia; Language disorder; Motor delay
DRP2	3	Echolalia; Motor delay; Unresponsiveness to spoken voice
DTL	3	Aggressive behaviour; Echolalia; Motor delay
DTX4	3	Anxiety; Echolalia; Mutism

DUOXA2	3	Echolalia; Language disorder; Motor delay
DYNC2LI1	3	Echolalia; Sleep disturbances; Unresponsiveness to spoken voice
DZIP1	3	Aggressive behaviour; Echolalia; Unresponsiveness to spoken voice
DZIP1L	3	Anxiety; Echolalia; Mutism
E2F7	3	Anxiety; Echolalia; Motor delay
E2F8	3	Echolalia; Motor delay; Unresponsiveness to spoken voice
EBF4	3	Anxiety; Echolalia; Mutism
ECM2	3	Echolalia; Motor delay; Unresponsiveness to spoken voice
EDARADD	3	Echolalia; Mutism; Sleep disturbances
EEF1AKMT4	3	Echolalia; Motor delay; Sleep disturbances
EEF2K	3	Aggressive behaviour; Echolalia; Unresponsiveness to spoken voice
EFCAB5	3	Anxiety; Echolalia; Motor delay
EGFL6	3	Echolalia; Sleep disturbances; Unresponsiveness to spoken voice
EHD1	3	Anxiety; Echolalia; Unresponsiveness to spoken voice
EHHADH	3	Echolalia; Language disorder; Motor delay
EIF3F	3	Anxiety; Echolalia; Unresponsiveness to spoken voice
EIF4E	3	Echolalia; Language disorder; Motor delay
EIF4EBP2	3	Echolalia; Sleep disturbances; Unresponsiveness to spoken voice
ELAC2	3	Echolalia; Language disorder; Motor delay
ELFN1	3	Echolalia; Motor delay; Unresponsiveness to spoken voice
ELMO2	3	Anxiety; Echolalia; Unresponsiveness to spoken voice
ELMOD1	3	Echolalia; Motor delay; Unresponsiveness to spoken voice
EML1	3	Anxiety; Echolalia; Unresponsiveness to spoken voice
EML5	3	Anxiety; Echolalia; Motor delay
ENPP3	3	Anxiety; Echolalia; Mutism
ERBB4	3	Anxiety; Echolalia; Unresponsiveness to spoken voice
ERO1B	3	Echolalia; Sleep disturbances; Unresponsiveness to spoken voice
ERVV-1	3	Anxiety; Echolalia; Unresponsiveness to spoken voice
ERVV-2	3	Echolalia; Language disorder; Motor delay
ESRP1	3	Anxiety; Echolalia; Unresponsiveness to spoken voice
ESS2	3	Echolalia; Mutism; Unresponsiveness to spoken voice
ETFB	3	Echolalia; Language disorder; Motor delay
ETNPPL	3	Echolalia; Sleep disturbances; Unresponsiveness to spoken voice
ETV4	3	Aggressive behaviour; Echolalia; Unresponsiveness to spoken voice
EXD1	3	Anxiety; Echolalia; Unresponsiveness to spoken voice
EXOC2	3	Echolalia; Motor delay; Sleep disturbances
EXT1	3	Anxiety; Echolalia; Motor delay
F13B	3	Echolalia; Language disorder; Motor delay
FAHD2A	3	Echolalia; Motor delay; Unresponsiveness to spoken voice
FAHD2B	3	Echolalia; Motor delay; Unresponsiveness to spoken voice
FAIM2	3	Anxiety; Motor delay; Unresponsiveness to spoken voice
FAM107A	3	Echolalia; Language disorder; Motor delay
FAM135B	3	Echolalia; Motor delay; Unresponsiveness to spoken voice
FAM153CP	3	Aggressive behaviour; Anxiety; Echolalia
FAM160B1	3	Digestive disorder; Echolalia; Unresponsiveness to spoken voice
FAM160B2	3	Echolalia; Sleep disturbances; Unresponsiveness to spoken voice

FAM161B	3	Echolalia; Language disorder; Motor delay
FAM178B	3	Echolalia; Motor delay; Unresponsiveness to spoken voice
FAM187A	3	Echolalia; Sleep disturbances; Unresponsiveness to spoken voice
FAM189A1	3	Anxiety; Echolalia; Unresponsiveness to spoken voice
FAM20A	3	Anxiety; Echolalia; Unresponsiveness to spoken voice
FAM222B	3	Echolalia; Sleep disturbances; Unresponsiveness to spoken voice
FAM234A	3	Echolalia; Motor delay; Unresponsiveness to spoken voice
FAM240A	3	Aggressive behaviour; Anxiety; Echolalia
FAM71F2	3	Anxiety; Echolalia; Unresponsiveness to spoken voice
FAM98C	3	Anxiety; Echolalia; Mutism
FARSA	3	Echolalia; Language disorder; Motor delay
FAS	3	Echolalia; Motor delay; Unresponsiveness to spoken voice
FBXL22	3	Echolalia; Language disorder; Mutism
FBXO21	3	Aggressive behaviour; Echolalia; Unresponsiveness to spoken voice
FBXO32	3	Aggressive behaviour; Echolalia; Motor delay
FBXO6	3	Echolalia; Language disorder; Motor delay
FCGR3B	3	Echolalia; Language disorder; Motor delay
FCRL3	3	Anxiety; Mutism; Sleep disturbances
FCRL6	3	Aggressive behaviour; Echolalia; Unresponsiveness to spoken voice
FEM1A	3	Echolalia; Language disorder; Motor delay
FER1L5	3	Echolalia; Sleep disturbances; Unresponsiveness to spoken voice
FGD6	3	Anxiety; Echolalia; Motor delay
FHL2	3	Anxiety; Echolalia; Mutism
FIZ1	3	Echolalia; Language disorder; Mutism
FLJ20712	3	Anxiety; Echolalia; Unresponsiveness to spoken voice
FMNL2	3	Echolalia; Language disorder; Mutism
FOXD4L1	3	Echolalia; Language disorder; Unresponsiveness to spoken voice
FOXF1	3	Anxiety; Echolalia; Mutism
FOXJ1	3	Echolalia; Motor delay; Unresponsiveness to spoken voice
FOXN4	3	Echolalia; Motor delay; Unresponsiveness to spoken voice
FPGT-TNNI3K	3	Echolalia; Sleep disturbances; Unresponsiveness to spoken voice
FRA10AC1	3	Echolalia; Motor delay; Unresponsiveness to spoken voice
FREM3	3	Echolalia; Language disorder; Motor delay
FRMPD4	3	Digestive disorder; Echolalia; Sleep disturbances
FRYL	3	Aggressive behaviour; Echolalia; Unresponsiveness to spoken voice
FTHL17	3	Echolalia; Language disorder; Motor delay
GABRA1	3	Anxiety; Echolalia; Unresponsiveness to spoken voice
GABRA4	3	Anxiety; Echolalia; Mutism
GABRE	3	Echolalia; Language disorder; Motor delay
GABRP	3	Anxiety; Echolalia; Unresponsiveness to spoken voice
GABRR1	3	Anxiety; Echolalia; Unresponsiveness to spoken voice
GAD2	3	Echolalia; Language disorder; Motor delay
GAGE1	3	Echolalia; Sleep disturbances; Unresponsiveness to spoken voice
GAL3ST3	3	Anxiety; Echolalia; Mutism
GALNT8	3	Anxiety; Echolalia; Unresponsiveness to spoken voice
GALNTL6	3	Echolalia; Language disorder; Motor delay

GALR3	3	Aggressive behaviour; Echolalia; Unresponsiveness to spoken voice
GART	3	Echolalia; Motor delay; Unresponsiveness to spoken voice
GAS1	3	Anxiety; Echolalia; Mutism
GATAD2A	3	Echolalia; Language disorder; Motor delay
GATM	3	Echolalia; Motor delay; Unresponsiveness to spoken voice
GCA	3	Echolalia; Mutism; Sleep disturbances
GCGR	3	Echolalia; Motor delay; Unresponsiveness to spoken voice
GDAP1	3	Aggressive behaviour; Echolalia; Unresponsiveness to spoken voice
GDAP2	3	Anxiety; Echolalia; Unresponsiveness to spoken voice
GDF9	3	Echolalia; Language disorder; Motor delay
GDPGP1	3	Echolalia; Language disorder; Mutism
GFOD2	3	Echolalia; Motor delay; Unresponsiveness to spoken voice
GFPT2	3	Echolalia; Language disorder; Motor delay
GGT8P	3	Echolalia; Sleep disturbances; Unresponsiveness to spoken voice
GH2	3	Echolalia; Motor delay; Unresponsiveness to spoken voice
GIGYF1	3	Echolalia; Sleep disturbances; Unresponsiveness to spoken voice
GIMAP4	3	Aggressive behaviour; Echolalia; Mutism
GIPC1	3	Echolalia; Sleep disturbances; Unresponsiveness to spoken voice
GJE1	3	Echolalia; Language disorder; Motor delay
GKN2	3	Aggressive behaviour; Echolalia; Unresponsiveness to spoken voice
GLG1	3	Echolalia; Language disorder; Motor delay
GLI4	3	Anxiety; Sleep disturbances; Unresponsiveness to spoken voice
GLRX5	3	Echolalia; Language disorder; Motor delay
GMPPB	3	Echolalia; Sleep disturbances; Unresponsiveness to spoken voice
GNA13	3	Anxiety; Echolalia; Mutism
GNAT2	3	Echolalia; Motor delay; Unresponsiveness to spoken voice
GNPAT	3	Echolalia; Motor delay; Unresponsiveness to spoken voice
GNRH2	3	Echolalia; Sleep disturbances; Unresponsiveness to spoken voice
GOLGA5	3	Echolalia; Language disorder; Motor delay
GOLGA6D	3	Anxiety; Echolalia; Unresponsiveness to spoken voice
GOLGB1	3	Echolalia; Language disorder; Motor delay
GPC1	3	Anxiety; Echolalia; Language disorder
GPC2	3	Echolalia; Sleep disturbances; Unresponsiveness to spoken voice
GPR135	3	Anxiety; Echolalia; Mutism
GPR158	3	Echolalia; Motor delay; Unresponsiveness to spoken voice
GPR174	3	Echolalia; Sleep disturbances; Unresponsiveness to spoken voice
GPR18	3	Echolalia; Language disorder; Motor delay
GPR183	3	Aggressive behaviour; Echolalia; Motor delay
GPR39	3	Echolalia; Language disorder; Motor delay
GPR63	3	Anxiety; Echolalia; Unresponsiveness to spoken voice
GPR83	3	Echolalia; Motor delay; Unresponsiveness to spoken voice
GPT	3	Anxiety; Echolalia; Unresponsiveness to spoken voice
GRAMD1B	3	Echolalia; Sleep disturbances; Unresponsiveness to spoken voice
GRB7	3	Echolalia; Motor delay; Unresponsiveness to spoken voice
GRHL3	3	Anxiety; Echolalia; Mutism
GRIA1	3	Aggressive behaviour; Echolalia; Unresponsiveness to spoken voice

GRIK4	3	Anxiety; Echolalia; Mutism
GRIN2B	3	Digestive disorder; Echolalia; Unresponsiveness to spoken voice
GRK1	3	Echolalia; Language disorder; Motor delay
GSTM3	3	Echolalia; Motor delay; Unresponsiveness to spoken voice
GSTP1	3	Echolalia; Motor delay; Unresponsiveness to spoken voice
GVQW3	3	Anxiety; Echolalia; Mutism
GYPB	3	Echolalia; Sleep disturbances; Unresponsiveness to spoken voice
H1-3	3	Anxiety; Echolalia; Mutism
H2BC18	3	Aggressive behaviour; Anxiety; Echolalia
H2BW1	3	Echolalia; Language disorder; Motor delay
H3C3	3	Anxiety; Echolalia; Mutism
H4C5	3	Anxiety; Echolalia; Mutism
H6PD	3	Echolalia; Language disorder; Unresponsiveness to spoken voice
HACE1	3	Echolalia; Language disorder; Motor delay
HAVCR1	3	Echolalia; Motor delay; Unresponsiveness to spoken voice
HBP1	3	Anxiety; Echolalia; Mutism
HCFC1	3	Anxiety; Echolalia; Mutism
HDLBP	3	Aggressive behaviour; Echolalia; Unresponsiveness to spoken voice
HEMK1	3	Aggressive behaviour; Echolalia; Unresponsiveness to spoken voice
HERC2	3	Anxiety; Echolalia; Mutism
HERC5	3	Anxiety; Echolalia; Motor delay
HFM1	3	Aggressive behaviour; Echolalia; Unresponsiveness to spoken voice
HGD	3	Echolalia; Language disorder; Motor delay
HHIPL2	3	Echolalia; Language disorder; Unresponsiveness to spoken voice
HLA-DRB5	3	Echolalia; Sleep disturbances; Unresponsiveness to spoken voice
HLX	3	Echolalia; Sleep disturbances; Unresponsiveness to spoken voice
HMGA2-AS1	3	Echolalia; Sleep disturbances; Unresponsiveness to spoken voice
HNRNPA3P1	3	Anxiety; Echolalia; Mutism
HOXA4	3	Anxiety; Echolalia; Mutism
HOXB5	3	Echolalia; Language disorder; Unresponsiveness to spoken voice
HOXC9	3	Echolalia; Mutism; Unresponsiveness to spoken voice
HPCAL1	3	Aggressive behaviour; Echolalia; Unresponsiveness to spoken voice
HPCAL4	3	Aggressive behaviour; Anxiety; Echolalia
HPF1	3	Echolalia; Motor delay; Unresponsiveness to spoken voice
HPGDS	3	Aggressive behaviour; Echolalia; Unresponsiveness to spoken voice
HRC	3	Echolalia; Mutism; Unresponsiveness to spoken voice
HS3ST4	3	Echolalia; Sleep disturbances; Unresponsiveness to spoken voice
HSD3B1	3	Echolalia; Motor delay; Unresponsiveness to spoken voice
HSPA12B	3	Echolalia; Language disorder; Motor delay
HSPA9	3	Anxiety; Echolalia; Unresponsiveness to spoken voice
HSPBAP1	3	Echolalia; Motor delay; Unresponsiveness to spoken voice
HTR1F	3	Aggressive behaviour; Echolalia; Unresponsiveness to spoken voice
HTR4	3	Anxiety; Echolalia; Mutism
HYAL4	3	Anxiety; Echolalia; Mutism
ICA1	3	Echolalia; Language disorder; Motor delay
ID3	3	Echolalia; Motor delay; Unresponsiveness to spoken voice

IDH3A	3	Anxiety; Echolalia; Mutism
IFFO2	3	Anxiety; Mutism; Sleep disturbances
IFIT1B	3	Echolalia; Motor delay; Unresponsiveness to spoken voice
IFNA21	3	Echolalia; Sleep disturbances; Unresponsiveness to spoken voice
IFNL4	3	Anxiety; Echolalia; Unresponsiveness to spoken voice
IFRD1	3	Echolalia; Mutism; Sleep disturbances
IFT46	3	Anxiety; Echolalia; Mutism
IFT80	3	Echolalia; Motor delay; Unresponsiveness to spoken voice
IFT81	3	Aggressive behaviour; Echolalia; Unresponsiveness to spoken voice
IGDCC4	3	Echolalia; Motor delay; Unresponsiveness to spoken voice
IGFBP1	3	Echolalia; Sleep disturbances; Unresponsiveness to spoken voice
IGSF10	3	Echolalia; Language disorder; Motor delay
IGSF3	3	Echolalia; Language disorder; Motor delay
IHH	3	Echolalia; Language disorder; Motor delay
IKBKB	3	Echolalia; Sleep disturbances; Unresponsiveness to spoken voice
IL15RA	3	Echolalia; Mutism; Unresponsiveness to spoken voice
IL7R	3	Echolalia; Motor delay; Unresponsiveness to spoken voice
IMMP2L	3	Echolalia; Sleep disturbances; Unresponsiveness to spoken voice
ING5	3	Anxiety; Echolalia; Mutism
INHBE	3	Echolalia; Language disorder; Motor delay
INO80D	3	Anxiety; Echolalia; Unresponsiveness to spoken voice
INPP5E	3	Echolalia; Motor delay; Unresponsiveness to spoken voice
INSR	3	Echolalia; Language disorder; Motor delay
INTS2	3	Anxiety; Echolalia; Mutism
IPO9	3	Anxiety; Echolalia; Mutism
IQCB1	3	Echolalia; Language disorder; Motor delay
IQCN	3	Echolalia; Sleep disturbances; Unresponsiveness to spoken voice
IQGAP3	3	Anxiety; Echolalia; Language disorder
IQSEC2	3	Anxiety; Echolalia; Motor delay
IRAK2	3	Aggressive behaviour; Anxiety; Echolalia
ISM2	3	Echolalia; Mutism; Sleep disturbances
ISYNA1	3	Anxiety; Echolalia; Unresponsiveness to spoken voice
ITGA11	3	Aggressive behaviour; Anxiety; Echolalia
ITGA8	3	Echolalia; Language disorder; Motor delay
ITGAL	3	Echolalia; Language disorder; Motor delay
ITGB7	3	Anxiety; Echolalia; Mutism
ITK	3	Echolalia; Language disorder; Motor delay
ITSN2	3	Echolalia; Language disorder; Motor delay
JADE2	3	Echolalia; Motor delay; Unresponsiveness to spoken voice
JAML	3	Echolalia; Motor delay; Unresponsiveness to spoken voice
JCAD	3	Echolalia; Language disorder; Motor delay
KANK2	3	Aggressive behaviour; Echolalia; Unresponsiveness to spoken voice
KANSL2	3	Echolalia; Sleep disturbances; Unresponsiveness to spoken voice
KAT8	3	Echolalia; Motor delay; Unresponsiveness to spoken voice
KAZN-AS1	3	Anxiety; Echolalia; Mutism
KCNA7	3	Echolalia; Motor delay; Unresponsiveness to spoken voice

KCNG2	3	Echolalia; Sleep disturbances; Unresponsiveness to spoken voice
KCNK4	3	Echolalia; Sleep disturbances; Unresponsiveness to spoken voice
KCNN4	3	Echolalia; Sleep disturbances; Unresponsiveness to spoken voice
KCNV2	3	Echolalia; Language disorder; Motor delay
KDF1	3	Echolalia; Motor delay; Unresponsiveness to spoken voice
KDM1B	3	Echolalia; Language disorder; Unresponsiveness to spoken voice
KDM3A	3	Echolalia; Language disorder; Motor delay
KDM4E	3	Anxiety; Echolalia; Mutism
KDR	3	Anxiety; Echolalia; Mutism
KIAA0040	3	Echolalia; Sleep disturbances; Unresponsiveness to spoken voice
KIAA0319	3	Anxiety; Echolalia; Mutism
KIAA0895L	3	Aggressive behaviour; Echolalia; Unresponsiveness to spoken voice
KIF20A	3	Aggressive behaviour; Echolalia; Unresponsiveness to spoken voice
KIF20B	3	Echolalia; Mutism; Unresponsiveness to spoken voice
KIF21A	3	Aggressive behaviour; Anxiety; Echolalia
KIF21B	3	Anxiety; Echolalia; Mutism
KIF22	3	Echolalia; Motor delay; Sleep disturbances
KLC1	3	Anxiety; Echolalia; Unresponsiveness to spoken voice
KLC4	3	Echolalia; Language disorder; Motor delay
KLF18	3	Anxiety; Echolalia; Unresponsiveness to spoken voice
KLHL14	3	Echolalia; Motor delay; Unresponsiveness to spoken voice
KLHL40	3	Echolalia; Motor delay; Unresponsiveness to spoken voice
KLHL41	3	Echolalia; Motor delay; Unresponsiveness to spoken voice
KLK10	3	Anxiety; Echolalia; Mutism
KLK4	3	Echolalia; Sleep disturbances; Unresponsiveness to spoken voice
KLRC2	3	Echolalia; Language disorder; Motor delay
KLRD1	3	Echolalia; Mutism; Unresponsiveness to spoken voice
KNL1	3	Digestive disorder; Echolalia; Sleep disturbances
KRAS	3	Echolalia; Language disorder; Motor delay
KRBA2	3	Anxiety; Echolalia; Motor delay
KRT10	3	Anxiety; Echolalia; Mutism
KRT18	3	Echolalia; Sleep disturbances; Unresponsiveness to spoken voice
KRT24	3	Anxiety; Echolalia; Mutism
KRT28	3	Anxiety; Echolalia; Mutism
KRT36	3	Echolalia; Sleep disturbances; Unresponsiveness to spoken voice
KRT6A	3	Anxiety; Echolalia; Unresponsiveness to spoken voice
KRT77	3	Echolalia; Language disorder; Unresponsiveness to spoken voice
KRT82	3	Anxiety; Echolalia; Unresponsiveness to spoken voice
KRT83	3	Aggressive behaviour; Echolalia; Motor delay
KRTAP10-12	3	Echolalia; Mutism; Sleep disturbances
KRTAP10-4	3	Anxiety; Echolalia; Mutism
KRTAP10-6	3	Aggressive behaviour; Echolalia; Unresponsiveness to spoken voice
KRTAP12-1	3	Echolalia; Language disorder; Motor delay
KRTAP2-2	3	Anxiety; Echolalia; Mutism
KRTAP4-11	3	Anxiety; Echolalia; Mutism
KRTAP4-5	3	Aggressive behaviour; Echolalia; Unresponsiveness to spoken voice

KRTAP9-7	3	Anxiety; Echolalia; Mutism
KY	3	Aggressive behaviour; Echolalia; Mutism
KYNU	3	Anxiety; Echolalia; Motor delay
L3MBTL4	3	Anxiety; Echolalia; Unresponsiveness to spoken voice
LAMB3	3	Echolalia; Motor delay; Unresponsiveness to spoken voice
LARGE2	3	Echolalia; Motor delay; Mutism
LARP7	3	Aggressive behaviour; Anxiety; Echolalia
LASP1	3	Echolalia; Language disorder; Motor delay
LATS1	3	Anxiety; Echolalia; Mutism
LDAH	3	Aggressive behaviour; Echolalia; Unresponsiveness to spoken voice
LECT2	3	Anxiety; Echolalia; Mutism
LEMD2	3	Anxiety; Echolalia; Unresponsiveness to spoken voice
LEMD3	3	Anxiety; Echolalia; Mutism
LGALS9C	3	Echolalia; Language disorder; Motor delay
LHFPL1	3	Echolalia; Motor delay; Unresponsiveness to spoken voice
LIME1	3	Anxiety; Echolalia; Unresponsiveness to spoken voice
LINC00205	3	Anxiety; Echolalia; Mutism
LINC00452	3	Anxiety; Echolalia; Unresponsiveness to spoken voice
LINC00619	3	Anxiety; Echolalia; Mutism
LINC01711	3	Echolalia; Language disorder; Motor delay
LINC01865	3	Anxiety; Echolalia; Mutism
LINC02801	3	Anxiety; Echolalia; Unresponsiveness to spoken voice
LIPF	3	Anxiety; Echolalia; Mutism
LIPH	3	Aggressive behaviour; Echolalia; Motor delay
LLGL1	3	Anxiety; Echolalia; Motor delay
LLGL2	3	Echolalia; Motor delay; Unresponsiveness to spoken voice
LMOD1	3	Anxiety; Echolalia; Mutism
LMTK2	3	Aggressive behaviour; Echolalia; Language disorder
LMX1A	3	Anxiety; Echolalia; Unresponsiveness to spoken voice
LOC100129434	3	Echolalia; Language disorder; Motor delay
LOC102723968	3	Anxiety; Echolalia; Mutism
LOC102724159	3	Echolalia; Sleep disturbances; Unresponsiveness to spoken voice
LOC107985184	3	Echolalia; Motor delay; Unresponsiveness to spoken voice
LOC107985524	3	Anxiety; Echolalia; Mutism
LOC107985770	3	Echolalia; Mutism; Unresponsiveness to spoken voice
LOC107986453	3	Echolalia; Motor delay; Unresponsiveness to spoken voice
LOC390877	3	Anxiety; Echolalia; Unresponsiveness to spoken voice
LOC400710	3	Echolalia; Motor delay; Unresponsiveness to spoken voice
LOC643802	3	Echolalia; Motor delay; Unresponsiveness to spoken voice
LPO	3	Anxiety; Echolalia; Unresponsiveness to spoken voice
LRR1	3	Aggressive behaviour; Echolalia; Unresponsiveness to spoken voice
LRRC1	3	Echolalia; Motor delay; Unresponsiveness to spoken voice
LRRC14	3	Anxiety; Echolalia; Mutism
LRRC18	3	Anxiety; Echolalia; Mutism
LRRC32	3	Aggressive behaviour; Echolalia; Mutism
LRRC37A2	3	Echolalia; Language disorder; Motor delay

LRRC3C	3	Anxiety; Echolalia; Mutism
LRRC58	3	Echolalia; Sleep disturbances; Unresponsiveness to spoken voice
LRRC6	3	Anxiety; Echolalia; Language disorder
LRRC74B	3	Echolalia; Language disorder; Motor delay
LRRC8E	3	Aggressive behaviour; Echolalia; Unresponsiveness to spoken voice
LRRTM1	3	Echolalia; Language disorder; Motor delay
LTA4H	3	Echolalia; Language disorder; Motor delay
LY75	3	Anxiety; Echolalia; Mutism
LY75-CD302	3	Anxiety; Echolalia; Mutism
LY86	3	Echolalia; Sleep disturbances; Unresponsiveness to spoken voice
LYAR	3	Echolalia; Language disorder; Motor delay
LYN	3	Echolalia; Sleep disturbances; Unresponsiveness to spoken voice
LYPD3	3	Echolalia; Mutism; Unresponsiveness to spoken voice
LZTS1	3	Anxiety; Echolalia; Unresponsiveness to spoken voice
MACO1	3	Anxiety; Echolalia; Mutism
MACROH2A2	3	Anxiety; Echolalia; Mutism
MAFB	3	Echolalia; Language disorder; Motor delay
MAGEA11	3	Echolalia; Motor delay; Unresponsiveness to spoken voice
MAGEA9	3	Echolalia; Motor delay; Unresponsiveness to spoken voice
MAGEA9B	3	Echolalia; Motor delay; Unresponsiveness to spoken voice
MAGEB2	3	Echolalia; Language disorder; Motor delay
MAGED2	3	Anxiety; Echolalia; Mutism
MAMDC4	3	Anxiety; Echolalia; Language disorder
MAN2B1	3	Anxiety; Echolalia; Mutism
MANSC4	3	Anxiety; Echolalia; Unresponsiveness to spoken voice
MAOA	3	Echolalia; Motor delay; Unresponsiveness to spoken voice
MAP3K10	3	Anxiety; Echolalia; Mutism
MAP3K6	3	Echolalia; Motor delay; Unresponsiveness to spoken voice
MAP3K9	3	Aggressive behaviour; Echolalia; Unresponsiveness to spoken voice
MAP4K4	3	Echolalia; Language disorder; Motor delay
MAPK6	3	Anxiety; Echolalia; Sleep disturbances
MAPT	3	Anxiety; Echolalia; Sleep disturbances
MARCHF4	3	Echolalia; Sleep disturbances; Unresponsiveness to spoken voice
MAT1A	3	Anxiety; Echolalia; Mutism
MATN4	3	Anxiety; Echolalia; Mutism
MBD3L2	3	Anxiety; Echolalia; Unresponsiveness to spoken voice
MCCC2	3	Anxiety; Echolalia; Mutism
MCF2L	3	Anxiety; Echolalia; Unresponsiveness to spoken voice
MCOLN3	3	Anxiety; Echolalia; Unresponsiveness to spoken voice
MDFI	3	Echolalia; Motor delay; Unresponsiveness to spoken voice
MDGA1	3	Echolalia; Motor delay; Unresponsiveness to spoken voice
MDH1B	3	Anxiety; Echolalia; Mutism
MED15	3	Anxiety; Echolalia; Mutism
MED17	3	Echolalia; Language disorder; Motor delay
MEFV	3	Anxiety; Echolalia; Sleep disturbances
MEIOB	3	Echolalia; Language disorder; Motor delay

METTL11B	3	Echolalia; Language disorder; Motor delay
METTL18	3	Echolalia; Language disorder; Motor delay
METTL27	3	Anxiety; Echolalia; Mutism
METTL3	3	Echolalia; Sleep disturbances; Unresponsiveness to spoken voice
METTL7B	3	Anxiety; Mutism; Sleep disturbances
MFF	3	Echolalia; Motor delay; Unresponsiveness to spoken voice
MGAT4B	3	Echolalia; Mutism; Unresponsiveness to spoken voice
MGAT5B	3	Anxiety; Echolalia; Unresponsiveness to spoken voice
MGME1	3	Echolalia; Motor delay; Unresponsiveness to spoken voice
MICAL2	3	Echolalia; Motor delay; Unresponsiveness to spoken voice
MICALCL	3	Echolalia; Language disorder; Unresponsiveness to spoken voice
MICOS10	3	Echolalia; Motor delay; Unresponsiveness to spoken voice
MIR1199	3	Anxiety; Echolalia; Mutism
MIR5093	3	Echolalia; Mutism; Unresponsiveness to spoken voice
MISP3	3	Anxiety; Echolalia; Mutism
MKRN1	3	Anxiety; Echolalia; Unresponsiveness to spoken voice
MKS1	3	Anxiety; Echolalia; Language disorder
MLXIP	3	Anxiety; Echolalia; Mutism
MNS1	3	Anxiety; Echolalia; Mutism
MOCS1	3	Echolalia; Language disorder; Motor delay
MORN1	3	Echolalia; Mutism; Sleep disturbances
MORN4	3	Anxiety; Echolalia; Mutism
MPPED1	3	Echolalia; Sleep disturbances; Unresponsiveness to spoken voice
MRGPRE	3	Echolalia; Language disorder; Motor delay
MRGPRF	3	Anxiety; Echolalia; Unresponsiveness to spoken voice
MRNIP	3	Anxiety; Echolalia; Mutism
MROH1	3	Anxiety; Echolalia; Mutism
MRPL40	3	Echolalia; Sleep disturbances; Unresponsiveness to spoken voice
MRPS10	3	Echolalia; Language disorder; Motor delay
MRPS12	3	Anxiety; Echolalia; Mutism
MRPS2	3	Anxiety; Echolalia; Unresponsiveness to spoken voice
MRPS28	3	Aggressive behaviour; Echolalia; Unresponsiveness to spoken voice
MRTFA	3	Aggressive behaviour; Echolalia; Unresponsiveness to spoken voice
MS4A13	3	Echolalia; Language disorder; Motor delay
MST1	3	Echolalia; Motor delay; Unresponsiveness to spoken voice
MST1L	3	Anxiety; Echolalia; Unresponsiveness to spoken voice
MT1E	3	Aggressive behaviour; Echolalia; Unresponsiveness to spoken voice
MT1H	3	Echolalia; Language disorder; Motor delay
MTARC2	3	Aggressive behaviour; Echolalia; Unresponsiveness to spoken voice
MTG2	3	Echolalia; Language disorder; Unresponsiveness to spoken voice
MTHFD1L	3	Echolalia; Language disorder; Motor delay
MTNR1A	3	Aggressive behaviour; Echolalia; Motor delay
MUC13	3	Anxiety; Echolalia; Motor delay
MUC17	3	Echolalia; Sleep disturbances; Unresponsiveness to spoken voice
MXRA5	3	Echolalia; Language disorder; Motor delay
MYBPHL	3	Echolalia; Mutism; Unresponsiveness to spoken voice

MYCBPAP	3	Aggressive behaviour; Echolalia; Motor delay
MYD88	3	Anxiety; Echolalia; Unresponsiveness to spoken voice
MYL6	3	Motor delay; Sleep disturbances; Unresponsiveness to spoken voice
MYNN	3	Echolalia; Sleep disturbances; Unresponsiveness to spoken voice
MYO1A	3	Anxiety; Echolalia; Mutism
MYOM3	3	Echolalia; Motor delay; Sleep disturbances
MYOZ3	3	Anxiety; Echolalia; Mutism
MYSM1	3	Echolalia; Language disorder; Motor delay
N4BP2L2	3	Anxiety; Echolalia; Mutism
NAALADL1	3	Echolalia; Language disorder; Motor delay
NAIF1	3	Aggressive behaviour; Echolalia; Unresponsiveness to spoken voice
NANOS1	3	Echolalia; Motor delay; Unresponsiveness to spoken voice
NAPSA	3	Echolalia; Sleep disturbances; Unresponsiveness to spoken voice
NARS1	3	Anxiety; Echolalia; Motor delay
NAT8	3	Echolalia; Motor delay; Unresponsiveness to spoken voice
NAV2	3	Aggressive behaviour; Echolalia; Unresponsiveness to spoken voice
NCALD	3	Echolalia; Sleep disturbances; Unresponsiveness to spoken voice
NCAPD3	3	Anxiety; Echolalia; Motor delay
NCF2	3	Echolalia; Language disorder; Unresponsiveness to spoken voice
NCKAP1L	3	Echolalia; Language disorder; Mutism
NCKAP5	3	Echolalia; Mutism; Unresponsiveness to spoken voice
NCOA3	3	Echolalia; Sleep disturbances; Unresponsiveness to spoken voice
NCR2	3	Aggressive behaviour; Echolalia; Motor delay
NCR3LG1	3	Echolalia; Language disorder; Motor delay
NDUFAB1	3	Echolalia; Motor delay; Unresponsiveness to spoken voice
NDUFB9	3	Echolalia; Motor delay; Unresponsiveness to spoken voice
NDUFV2	3	Echolalia; Sleep disturbances; Unresponsiveness to spoken voice
NEFH	3	Echolalia; Language disorder; Motor delay
NEK8	3	Anxiety; Echolalia; Unresponsiveness to spoken voice
NET1	3	Aggressive behaviour; Echolalia; Unresponsiveness to spoken voice
NEXMIF	3	Echolalia; Language disorder; Motor delay
NFKBIA	3	Echolalia; Mutism; Sleep disturbances
NFYC	3	Anxiety; Echolalia; Mutism
NGDN	3	Echolalia; Language disorder; Motor delay
NHLRC2	3	Anxiety; Echolalia; Unresponsiveness to spoken voice
NIBAN3	3	Echolalia; Sleep disturbances; Unresponsiveness to spoken voice
NIF3L1	3	Echolalia; Motor delay; Unresponsiveness to spoken voice
NIPAL1	3	Echolalia; Language disorder; Motor delay
NIPAL2	3	Anxiety; Echolalia; Sleep disturbances
NIPBL	3	Echolalia; Language disorder; Motor delay
NKAPL	3	Anxiety; Echolalia; Mutism
NKX2-3	3	Echolalia; Motor delay; Unresponsiveness to spoken voice
NLGN4X	3	Echolalia; Language disorder; Motor delay
NLRP3	3	Echolalia; Mutism; Unresponsiveness to spoken voice
NOCT	3	Anxiety; Echolalia; Unresponsiveness to spoken voice
NOL11	3	Echolalia; Language disorder; Motor delay

NOLC1	3	Anxiety; Language disorder; Motor delay
NOP2	3	Aggressive behaviour; Echolalia; Unresponsiveness to spoken voice
NOS1AP	3	Echolalia; Language disorder; Motor delay
NOSIP	3	Echolalia; Language disorder; Motor delay
NPAP1	3	Echolalia; Sleep disturbances; Unresponsiveness to spoken voice
NPAS3	3	Anxiety; Echolalia; Unresponsiveness to spoken voice
NPC1	3	Echolalia; Motor delay; Unresponsiveness to spoken voice
NPEPPS	3	Echolalia; Language disorder; Motor delay
NPHS2	3	Aggressive behaviour; Echolalia; Unresponsiveness to spoken voice
NPIPB2	3	Anxiety; Echolalia; Mutism
NPPA	3	Echolalia; Language disorder; Motor delay
3,00 NPR	3	Echolalia; Language disorder; Motor delay
NPTX1	3	Echolalia; Motor delay; Unresponsiveness to spoken voice
NPY	3	Anxiety; Echolalia; Unresponsiveness to spoken voice
NQO2	3	Echolalia; Motor delay; Unresponsiveness to spoken voice
NR0B1	3	Echolalia; Sleep disturbances; Unresponsiveness to spoken voice
NRK	3	Echolalia; Sleep disturbances; Unresponsiveness to spoken voice
NRP2	3	Aggressive behaviour; Echolalia; Unresponsiveness to spoken voice
NT5E	3	Aggressive behaviour; Echolalia; Unresponsiveness to spoken voice
NTPCR	3	Echolalia; Motor delay; Unresponsiveness to spoken voice
NUBP1	3	Echolalia; Sleep disturbances; Unresponsiveness to spoken voice
NUFIP2	3	Echolalia; Sleep disturbances; Unresponsiveness to spoken voice
NUMB	3	Anxiety; Echolalia; Mutism
NUP214	3	Echolalia; Language disorder; Motor delay
NUP42	3	Aggressive behaviour; Anxiety; Echolalia
NUTM1	3	Aggressive behaviour; Echolalia; Unresponsiveness to spoken voice
NUTM2F	3	Echolalia; Language disorder; Motor delay
NXPE2	3	Echolalia; Motor delay; Unresponsiveness to spoken voice
OARD1	3	Echolalia; Language disorder; Motor delay
OBP2A	3	Echolalia; Motor delay; Sleep disturbances
OBSCN	3	Echolalia; Motor delay; Sleep disturbances
ONECUT2	3	Aggressive behaviour; Echolalia; Unresponsiveness to spoken voice
OR10AD1	3	Echolalia; Motor delay; Unresponsiveness to spoken voice
OR10H3	3	Echolalia; Sleep disturbances; Unresponsiveness to spoken voice
OR10Q1	3	Echolalia; Sleep disturbances; Unresponsiveness to spoken voice
OR10W1	3	Echolalia; Motor delay; Unresponsiveness to spoken voice
OR11H4	3	Echolalia; Motor delay; Unresponsiveness to spoken voice
OR13C3	3	Echolalia; Motor delay; Unresponsiveness to spoken voice
OR13C8	3	Echolalia; Motor delay; Unresponsiveness to spoken voice
OR13H1	3	Echolalia; Mutism; Unresponsiveness to spoken voice
OR1A1	3	Aggressive behaviour; Anxiety; Echolalia
OR1A2	3	Digestive disorder; Echolalia; Motor delay
OR1J1	3	Echolalia; Language disorder; Mutism
OR2A7	3	Echolalia; Mutism; Unresponsiveness to spoken voice
OR2AG1	3	Echolalia; Motor delay; Unresponsiveness to spoken voice
OR2D3	3	Aggressive behaviour; Echolalia; Unresponsiveness to spoken voice

OR2M3	3	Anxiety; Echolalia; Mutism
OR4M1	3	Echolalia; Sleep disturbances; Unresponsiveness to spoken voice
OR4X1	3	Echolalia; Language disorder; Motor delay
OR51B5	3	Echolalia; Mutism; Sleep disturbances
OR51J1	3	Anxiety; Echolalia; Sleep disturbances
OR51S1	3	Anxiety; Echolalia; Unresponsiveness to spoken voice
OR52B6	3	Echolalia; Motor delay; Unresponsiveness to spoken voice
OR52I2	3	Aggressive behaviour; Echolalia; Unresponsiveness to spoken voice
OR52M1	3	Aggressive behaviour; Echolalia; Unresponsiveness to spoken voice
OR52N2	3	Anxiety; Echolalia; Mutism
OR52N4	3	Anxiety; Echolalia; Mutism
OR52N5	3	Anxiety; Echolalia; Mutism
OR5A2	3	Echolalia; Motor delay; Unresponsiveness to spoken voice
OR5B12	3	Anxiety; Motor delay; Unresponsiveness to spoken voice
OR5D16	3	Aggressive behaviour; Echolalia; Unresponsiveness to spoken voice
OR5H1	3	Anxiety; Echolalia; Mutism
OR5H14	3	Anxiety; Echolalia; Mutism
OR5H6	3	Anxiety; Echolalia; Mutism
OR5M9	3	Aggressive behaviour; Echolalia; Unresponsiveness to spoken voice
OR6C6	3	Echolalia; Language disorder; Motor delay
OR6C70	3	Anxiety; Echolalia; Sleep disturbances
OR6N1	3	Aggressive behaviour; Echolalia; Unresponsiveness to spoken voice
OR6P1	3	Echolalia; Motor delay; Unresponsiveness to spoken voice
OR6T1	3	Echolalia; Mutism; Unresponsiveness to spoken voice
OR7E156P	3	Anxiety; Echolalia; Mutism
OR8B4	3	Aggressive behaviour; Echolalia; Unresponsiveness to spoken voice
OR8D1	3	Aggressive behaviour; Echolalia; Unresponsiveness to spoken voice
OR8D4	3	Anxiety; Echolalia; Mutism
ORAI1	3	Anxiety; Echolalia; Mutism
ORC2	3	Anxiety; Echolalia; Unresponsiveness to spoken voice
OSBPL6	3	Anxiety; Echolalia; Mutism
OSMR-AS1	3	Aggressive behaviour; Echolalia; Unresponsiveness to spoken voice
OTOG	3	Aggressive behaviour; Echolalia; Unresponsiveness to spoken voice
P2RX2	3	Aggressive behaviour; Echolalia; Unresponsiveness to spoken voice
PABPC3	3	Echolalia; Language disorder; Motor delay
PAFAH1B1	3	Anxiety; Echolalia; Mutism
PANX3	3	Echolalia; Language disorder; Motor delay
PAQR9	3	Echolalia; Language disorder; Motor delay
PARM1	3	Echolalia; Sleep disturbances; Unresponsiveness to spoken voice
PARP16	3	Echolalia; Sleep disturbances; Unresponsiveness to spoken voice
PBLD	3	Anxiety; Echolalia; Motor delay
PCBP3	3	Echolalia; Language disorder; Motor delay
PCDH9	3	Echolalia; Language disorder; Motor delay
PCDHA10	3	Anxiety; Echolalia; Mutism
PCDHB5	3	Anxiety; Echolalia; Unresponsiveness to spoken voice
PCDHB7	3	Aggressive behaviour; Anxiety; Echolalia

PCDHGC3	3	Anxiety; Echolalia; Mutism
PCP2	3	Echolalia; Motor delay; Unresponsiveness to spoken voice
PCSK1N	3	Echolalia; Motor delay; Unresponsiveness to spoken voice
PCSK4	3	Echolalia; Language disorder; Motor delay
PCSK5	3	Echolalia; Mutism; Unresponsiveness to spoken voice
PCTP	3	Echolalia; Language disorder; Motor delay
PDCD6IP	3	Echolalia; Language disorder; Motor delay
PDE3B	3	Echolalia; Motor delay; Unresponsiveness to spoken voice
PDE4C	3	Anxiety; Echolalia; Mutism
PDE6A	3	Echolalia; Language disorder; Motor delay
PDE8B	3	Echolalia; Language disorder; Motor delay
PDGFA	3	Echolalia; Language disorder; Motor delay
PDHA2	3	Anxiety; Echolalia; Mutism
PDHB	3	Echolalia; Motor delay; Unresponsiveness to spoken voice
PECR	3	Echolalia; Motor delay; Unresponsiveness to spoken voice
PEPD	3	Echolalia; Language disorder; Motor delay
PEX11A	3	Echolalia; Sleep disturbances; Unresponsiveness to spoken voice
PGAM2	3	Aggressive behaviour; Echolalia; Unresponsiveness to spoken voice
PGBD1	3	Anxiety; Echolalia; Mutism
PHF7	3	Digestive disorder; Echolalia; Motor delay
PHGR1	3	Echolalia; Sleep disturbances; Unresponsiveness to spoken voice
PI4K2A	3	Anxiety; Echolalia; Mutism
PIF1	3	Aggressive behaviour; Echolalia; Unresponsiveness to spoken voice
PIGA	3	Anxiety; Echolalia; Mutism
PIH1D2	3	Anxiety; Echolalia; Sleep disturbances
PIK3CA	3	Echolalia; Language disorder; Motor delay
PIK3IP1	3	Echolalia; Motor delay; Unresponsiveness to spoken voice
PIK3R1	3	Aggressive behaviour; Echolalia; Unresponsiveness to spoken voice
PINK1	3	Echolalia; Motor delay; Unresponsiveness to spoken voice
PITRM1	3	Anxiety; Echolalia; Unresponsiveness to spoken voice
PLA2G3	3	Anxiety; Echolalia; Mutism
PLAC4	3	Anxiety; Echolalia; Unresponsiveness to spoken voice
PLAT	3	Echolalia; Motor delay; Unresponsiveness to spoken voice
PLAU	3	Echolalia; Mutism; Unresponsiveness to spoken voice
PLCB2	3	Anxiety; Echolalia; Unresponsiveness to spoken voice
PLCD1	3	Echolalia; Language disorder; Motor delay
PLEKHB1	3	Echolalia; Language disorder; Motor delay
PLEKHG5	3	Echolalia; Language disorder; Motor delay
PLOD3	3	Echolalia; Language disorder; Motor delay
PLSCR4	3	Echolalia; Motor delay; Unresponsiveness to spoken voice
PLXNA1	3	Echolalia; Motor delay; Unresponsiveness to spoken voice
PMEL	3	Aggressive behaviour; Echolalia; Unresponsiveness to spoken voice
PMFBP1	3	Echolalia; Mutism; Unresponsiveness to spoken voice
PNMA5	3	Echolalia; Motor delay; Unresponsiveness to spoken voice
PNPLA3	3	Echolalia; Language disorder; Motor delay
POC1A	3	Anxiety; Echolalia; Mutism

PODXL	3	Anxiety; Echolalia; Mutism
POLA2	3	Echolalia; Language disorder; Motor delay
POLDIP3	3	Anxiety; Echolalia; Mutism
POLH	3	Echolalia; Sleep disturbances; Unresponsiveness to spoken voice
POLR1G	3	Anxiety; Echolalia; Mutism
POLR2A	3	Anxiety; Echolalia; Mutism
POLR2J3	3	Aggressive behaviour; Echolalia; Unresponsiveness to spoken voice
POLR3B	3	Aggressive behaviour; Echolalia; Motor delay
POM121C	3	Anxiety; Echolalia; Mutism
POTEF	3	Echolalia; Motor delay; Unresponsiveness to spoken voice
PPIAL4C	3	Echolalia; Language disorder; Motor delay
PPIL2	3	Anxiety; Echolalia; Mutism
PPP1CC	3	Anxiety; Echolalia; Unresponsiveness to spoken voice
PPP1R7	3	Anxiety; Echolalia; Mutism
PPP2R5C	3	Echolalia; Language disorder; Mutism
PPP4R1	3	Echolalia; Language disorder; Motor delay
PRB1	3	Anxiety; Echolalia; Mutism
PRB2	3	Anxiety; Echolalia; Mutism
PRC1	3	Anxiety; Echolalia; Mutism
PRDM14	3	Echolalia; Motor delay; Unresponsiveness to spoken voice
PREB	3	Anxiety; Echolalia; Mutism
PRELP	3	Echolalia; Mutism; Unresponsiveness to spoken voice
PRKAG3	3	Anxiety; Echolalia; Mutism
PRKCG	3	Echolalia; Language disorder; Motor delay
PRKG1	3	Aggressive behaviour; Echolalia; Unresponsiveness to spoken voice
PRNP	3	Anxiety; Echolalia; Unresponsiveness to spoken voice
PRODH2	3	Echolalia; Motor delay; Unresponsiveness to spoken voice
PROS1	3	Anxiety; Echolalia; Mutism
PRR15	3	Echolalia; Language disorder; Motor delay
PRSS36	3	Echolalia; Sleep disturbances; Unresponsiveness to spoken voice
PRSS54	3	Echolalia; Language disorder; Motor delay
PRSS56	3	Echolalia; Language disorder; Mutism
PRSS57	3	Echolalia; Motor delay; Unresponsiveness to spoken voice
PRUNE2	3	Echolalia; Sleep disturbances; Unresponsiveness to spoken voice
PSEN2	3	Anxiety; Echolalia; Sleep disturbances
PSG7	3	Echolalia; Language disorder; Motor delay
PSMB4	3	Anxiety; Echolalia; Unresponsiveness to spoken voice
PTF1A	3	Echolalia; Motor delay; Unresponsiveness to spoken voice
PTGFR	3	Echolalia; Sleep disturbances; Unresponsiveness to spoken voice
PTMA	3	Anxiety; Echolalia; Unresponsiveness to spoken voice
PTOV1	3	Aggressive behaviour; Echolalia; Unresponsiveness to spoken voice
PTPN18	3	Echolalia; Language disorder; Motor delay
PTPN7	3	Echolalia; Motor delay; Unresponsiveness to spoken voice
PTPRE	3	Aggressive behaviour; Anxiety; Echolalia
PTPRR	3	Anxiety; Echolalia; Mutism
PUM1	3	Aggressive behaviour; Echolalia; Motor delay

PUS3	3	Anxiety; Echolalia; Mutism
PWP2	3	Echolalia; Sleep disturbances; Unresponsiveness to spoken voice
PXMP4	3	Echolalia; Sleep disturbances; Unresponsiveness to spoken voice
PYDC2	3	Echolalia; Motor delay; Unresponsiveness to spoken voice
QPRT	3	Echolalia; Motor delay; Unresponsiveness to spoken voice
R3HDML	3	Echolalia; Language disorder; Motor delay
RAB11B	3	Anxiety; Echolalia; Unresponsiveness to spoken voice
RAB18	3	Motor delay; Sleep disturbances; Unresponsiveness to spoken voice
RAB40A	3	Echolalia; Language disorder; Motor delay
RAB5A	3	Echolalia; Motor delay; Unresponsiveness to spoken voice
RAD18	3	Echolalia; Language disorder; Motor delay
RALB	3	Anxiety; Echolalia; Mutism
RALGAPA1	3	Echolalia; Language disorder; Motor delay
RAP1GAP	3	Echolalia; Motor delay; Unresponsiveness to spoken voice
RAP1GAP2	3	Echolalia; Sleep disturbances; Unresponsiveness to spoken voice
RAPGEF1	3	Echolalia; Language disorder; Motor delay
RAPSN	3	Echolalia; Motor delay; Unresponsiveness to spoken voice
RASA4	3	Aggressive behaviour; Echolalia; Unresponsiveness to spoken voice
RASSF2	3	Echolalia; Language disorder; Motor delay
RASSF6	3	Anxiety; Echolalia; Unresponsiveness to spoken voice
RAVER1	3	Aggressive behaviour; Echolalia; Unresponsiveness to spoken voice
RAX2	3	Aggressive behaviour; Echolalia; Motor delay
RBAK	3	Echolalia; Language disorder; Motor delay
RBM12	3	Aggressive behaviour; Anxiety; Echolalia
RBM27	3	Echolalia; Language disorder; Motor delay
RBMS1	3	Echolalia; Language disorder; Motor delay
RBP1	3	Echolalia; Language disorder; Motor delay
RBPJL	3	Anxiety; Mutism; Sleep disturbances
RBSN	3	Echolalia; Sleep disturbances; Unresponsiveness to spoken voice
RECQL	3	Echolalia; Motor delay; Unresponsiveness to spoken voice
REEP4	3	Anxiety; Echolalia; Unresponsiveness to spoken voice
REST	3	Anxiety; Echolalia; Motor delay
RETNLB	3	Aggressive behaviour; Echolalia; Motor delay
REX1BD	3	Anxiety; Echolalia; Mutism
RFX7	3	Aggressive behaviour; Echolalia; Unresponsiveness to spoken voice
RGMB	3	Echolalia; Language disorder; Motor delay
RGP1	3	Anxiety; Echolalia; Mutism
RGS19	3	Echolalia; Language disorder; Motor delay
RGS4	3	Anxiety; Echolalia; Mutism
RGS8	3	Anxiety; Echolalia; Mutism
RHOQ	3	Anxiety; Echolalia; Mutism
RIOX2	3	Anxiety; Language disorder; Mutism
RIT2	3	Anxiety; Echolalia; Mutism
RLF	3	Echolalia; Mutism; Unresponsiveness to spoken voice
RLN1	3	Anxiety; Echolalia; Mutism
RMC1	3	Echolalia; Sleep disturbances; Unresponsiveness to spoken voice

RMI2	3	Anxiety; Echolalia; Mutism
RNF149	3	Anxiety; Echolalia; Mutism
RNF150	3	Aggressive behaviour; Echolalia; Unresponsiveness to spoken voice
RNF165	3	Echolalia; Language disorder; Motor delay
RNF181	3	Aggressive behaviour; Echolalia; Unresponsiveness to spoken voice
RNF213-AS1	3	Echolalia; Motor delay; Unresponsiveness to spoken voice
RNF223	3	Echolalia; Motor delay; Unresponsiveness to spoken voice
RNPEP	3	Aggressive behaviour; Echolalia; Mutism
ROGDI	3	Anxiety; Echolalia; Mutism
ROPN1L	3	Anxiety; Echolalia; Unresponsiveness to spoken voice
RPARP-AS1	3	Anxiety; Motor delay; Mutism
RPL13	3	Anxiety; Echolalia; Unresponsiveness to spoken voice
RPTN	3	Echolalia; Language disorder; Motor delay
RPTOR	3	Anxiety; Echolalia; Motor delay
RRAGD	3	Echolalia; Motor delay; Unresponsiveness to spoken voice
RRP1B	3	Echolalia; Language disorder; Motor delay
RSL24D1	3	Anxiety; Echolalia; Mutism
RTL3	3	Anxiety; Echolalia; Unresponsiveness to spoken voice
RTN4IP1	3	Echolalia; Motor delay; Unresponsiveness to spoken voice
RUFY1	3	Anxiety; Echolalia; Mutism
RUSC1-AS1	3	Echolalia; Motor delay; Unresponsiveness to spoken voice
RWDD4	3	Aggressive behaviour; Motor delay; Unresponsiveness to spoken voice
RXRA	3	Echolalia; Language disorder; Motor delay
S100A7	3	Echolalia; Language disorder; Motor delay
S1PR4	3	Echolalia; Language disorder; Mutism
SAA4	3	Echolalia; Language disorder; Motor delay
SAMD1	3	Anxiety; Echolalia; Unresponsiveness to spoken voice
SAMD10	3	Echolalia; Mutism; Unresponsiveness to spoken voice
SAMD15	3	Anxiety; Echolalia; Mutism
SAR1B	3	Echolalia; Language disorder; Motor delay
SBK3	3	Echolalia; Language disorder; Motor delay
SCAF8	3	Aggressive behaviour; Echolalia; Unresponsiveness to spoken voice
SCN5A	3	Echolalia; Language disorder; Motor delay
SCRN3	3	Anxiety; Echolalia; Sleep disturbances
SDF4	3	Echolalia; Language disorder; Motor delay
SDR42E2	3	Echolalia; Sleep disturbances; Unresponsiveness to spoken voice
SEPSECS	3	Echolalia; Motor delay; Unresponsiveness to spoken voice
SEPTIN2	3	Anxiety; Echolalia; Mutism
SEPTIN4	3	Echolalia; Language disorder; Motor delay
SERPINA11	3	Echolalia; Language disorder; Unresponsiveness to spoken voice
SERPINA12	3	Anxiety; Mutism; Sleep disturbances
SERPINA5	3	Echolalia; Motor delay; Unresponsiveness to spoken voice
SERPINB11	3	Echolalia; Language disorder; Motor delay
SERPINB5	3	Anxiety; Echolalia; Mutism
SERPINB6	3	Echolalia; Language disorder; Motor delay
SERPINB7	3	Echolalia; Language disorder; Motor delay

SERTAD3	3	Echolalia; Language disorder; Motor delay
SETD1B	3	Anxiety; Echolalia; Mutism
SEZ6	3	Anxiety; Echolalia; Mutism
SF3B2	3	Echolalia; Motor delay; Unresponsiveness to spoken voice
SFMBT1	3	Echolalia; Language disorder; Motor delay
SFPQ	3	Echolalia; Language disorder; Motor delay
SFXN5	3	Anxiety; Echolalia; Mutism
SGIP1	3	Echolalia; Language disorder; Motor delay
SGPL1	3	Anxiety; Echolalia; Mutism
SGSH	3	Echolalia; Motor delay; Unresponsiveness to spoken voice
SH2B1	3	Echolalia; Language disorder; Motor delay
SH2D2A	3	Anxiety; Echolalia; Mutism
SH2D3A	3	Anxiety; Echolalia; Mutism
SH2D4B	3	Digestive disorder; Echolalia; Motor delay
SH2D6	3	Echolalia; Language disorder; Motor delay
SH3D21	3	Anxiety; Echolalia; Unresponsiveness to spoken voice
SHB	3	Anxiety; Echolalia; Unresponsiveness to spoken voice
SHD	3	Echolalia; Language disorder; Motor delay
SIGMAR1	3	Anxiety; Echolalia; Mutism
SIRT2	3	Anxiety; Echolalia; Mutism
SIX1	3	Anxiety; Echolalia; Unresponsiveness to spoken voice
SKIL	3	Echolalia; Sleep disturbances; Unresponsiveness to spoken voice
SKOR1	3	Anxiety; Echolalia; Unresponsiveness to spoken voice
SLAIN2	3	Echolalia; Mutism; Unresponsiveness to spoken voice
SLAMF6	3	Echolalia; Sleep disturbances; Unresponsiveness to spoken voice
SLC10A1	3	Echolalia; Motor delay; Unresponsiveness to spoken voice
SLC11A1	3	Aggressive behaviour; Echolalia; Unresponsiveness to spoken voice
SLC13A2	3	Anxiety; Echolalia; Mutism
SLC14A1	3	Echolalia; Mutism; Sleep disturbances
SLC15A3	3	Echolalia; Motor delay; Unresponsiveness to spoken voice
SLC16A11	3	Anxiety; Echolalia; Mutism
SLC17A6	3	Echolalia; Mutism; Sleep disturbances
SLC18A3	3	Echolalia; Motor delay; Unresponsiveness to spoken voice
SLC22A13	3	Anxiety; Mutism; Sleep disturbances
SLC22A31	3	Aggressive behaviour; Echolalia; Unresponsiveness to spoken voice
SLC24A4	3	Echolalia; Motor delay; Sleep disturbances
SLC25A10	3	Echolalia; Motor delay; Unresponsiveness to spoken voice
SLC25A13	3	Anxiety; Echolalia; Mutism
SLC25A2	3	Echolalia; Mutism; Unresponsiveness to spoken voice
SLC25A27	3	Anxiety; Echolalia; Mutism
SLC25A29	3	Anxiety; Echolalia; Unresponsiveness to spoken voice
SLC2A13	3	Echolalia; Motor delay; Unresponsiveness to spoken voice
SLC30A4	3	Echolalia; Sleep disturbances; Unresponsiveness to spoken voice
SLC31A1	3	Echolalia; Language disorder; Motor delay
SLC35G3	3	Digestive disorder; Echolalia; Unresponsiveness to spoken voice
SLC37A2	3	Echolalia; Sleep disturbances; Unresponsiveness to spoken voice

SLC38A7	3	Echolalia; Language disorder; Motor delay
SLC39A12	3	Anxiety; Echolalia; Mutism
SLC39A13	3	Echolalia; Sleep disturbances; Unresponsiveness to spoken voice
SLC39A2	3	Anxiety; Echolalia; Mutism
SLC41A2	3	Echolalia; Language disorder; Motor delay
SLC43A3	3	Echolalia; Motor delay; Unresponsiveness to spoken voice
SLC4A5	3	Echolalia; Language disorder; Motor delay
SLC4A7	3	Anxiety; Echolalia; Mutism
SLC4A9	3	Anxiety; Echolalia; Mutism
SLC5A11	3	Echolalia; Motor delay; Unresponsiveness to spoken voice
SLC5A12	3	Echolalia; Language disorder; Motor delay
SLC66A1	3	Anxiety; Echolalia; Motor delay
SLC66A1L	3	Aggressive behaviour; Echolalia; Unresponsiveness to spoken voice
SLC7A14	3	Echolalia; Sleep disturbances; Unresponsiveness to spoken voice
SLC9A5	3	Anxiety; Echolalia; Mutism
SLC9A7	3	Echolalia; Language disorder; Motor delay
SLITRK6	3	Anxiety; Echolalia; Unresponsiveness to spoken voice
SMAD7	3	Echolalia; Language disorder; Motor delay
SMARCAL1	3	Aggressive behaviour; Anxiety; Echolalia
SMCO1	3	Anxiety; Echolalia; Mutism
SMCO2	3	Echolalia; Language disorder; Unresponsiveness to spoken voice
SMCR8	3	Echolalia; Language disorder; Motor delay
SMG1P7	3	Echolalia; Sleep disturbances; Unresponsiveness to spoken voice
SMR3A	3	Echolalia; Language disorder; Motor delay
SMTNL1	3	Echolalia; Motor delay; Unresponsiveness to spoken voice
SNAP29	3	Echolalia; Language disorder; Motor delay
SNAPC5	3	Echolalia; Sleep disturbances; Unresponsiveness to spoken voice
SNED1	3	Echolalia; Sleep disturbances; Unresponsiveness to spoken voice
SNHG22	3	Aggressive behaviour; Echolalia; Motor delay
SNX4	3	Echolalia; Sleep disturbances; Unresponsiveness to spoken voice
SOCS5	3	Echolalia; Motor delay; Unresponsiveness to spoken voice
SOCS6	3	Echolalia; Language disorder; Motor delay
SORBS2	3	Echolalia; Motor delay; Unresponsiveness to spoken voice
SOX17	3	Echolalia; Mutism; Unresponsiveness to spoken voice
SOX5	3	Anxiety; Echolalia; Unresponsiveness to spoken voice
SP2	3	Echolalia; Language disorder; Motor delay
SP3	3	Echolalia; Language disorder; Motor delay
SP4	3	Anxiety; Mutism; Sleep disturbances
SPAG11B	3	Echolalia; Sleep disturbances; Unresponsiveness to spoken voice
SPANXN2	3	Echolalia; Language disorder; Motor delay
SPATA3-AS1	3	Mutism; Sleep disturbances; Unresponsiveness to spoken voice
SPATA31D1	3	Anxiety; Echolalia; Mutism
SPATA31D4	3	Anxiety; Echolalia; Mutism
SPATA48	3	Echolalia; Mutism; Unresponsiveness to spoken voice
SPATA7	3	Aggressive behaviour; Anxiety; Unresponsiveness to spoken voice
SPDYE3	3	Echolalia; Language disorder; Motor delay

SPDYE4	3	Aggressive behaviour; Echolalia; Unresponsiveness to spoken voice
SPDYE5	3	Anxiety; Echolalia; Sleep disturbances
SPINK8	3	Anxiety; Echolalia; Unresponsiveness to spoken voice
SPNS3	3	Echolalia; Motor delay; Unresponsiveness to spoken voice
SPOUT1	3	Echolalia; Sleep disturbances; Unresponsiveness to spoken voice
SPPL2C	3	Anxiety; Echolalia; Unresponsiveness to spoken voice
SQSTM1	3	Anxiety; Echolalia; Mutism
SRCIN1	3	Anxiety; Echolalia; Mutism
SRP19	3	Anxiety; Mutism; Sleep disturbances
SRRM3	3	Aggressive behaviour; Anxiety; Echolalia
SRSF6	3	Echolalia; Language disorder; Motor delay
SSC4D	3	Aggressive behaviour; Echolalia; Unresponsiveness to spoken voice
SSRP1	3	Anxiety; Echolalia; Unresponsiveness to spoken voice
SSTR4	3	Echolalia; Motor delay; Unresponsiveness to spoken voice
SSUH2	3	Echolalia; Motor delay; Unresponsiveness to spoken voice
ST8SIA5	3	Anxiety; Echolalia; Mutism
STAC	3	Echolalia; Language disorder; Motor delay
STARD3NL	3	Echolalia; Mutism; Sleep disturbances
STK17A	3	Aggressive behaviour; Echolalia; Unresponsiveness to spoken voice
STOX2	3	Aggressive behaviour; Echolalia; Unresponsiveness to spoken voice
STPG3	3	Echolalia; Language disorder; Motor delay
STX16-NPEPL1	3	Echolalia; Language disorder; Motor delay
SULT6B1	3	Anxiety; Echolalia; Mutism
SUMF2	3	Echolalia; Language disorder; Motor delay
SUOX	3	Anxiety; Echolalia; Unresponsiveness to spoken voice
SYCE1	3	Echolalia; Language disorder; Motor delay
SYDE2	3	Echolalia; Motor delay; Unresponsiveness to spoken voice
SYT16	3	Anxiety; Echolalia; Unresponsiveness to spoken voice
SYVN1	3	Echolalia; Language disorder; Motor delay
SZRD1	3	Anxiety; Echolalia; Unresponsiveness to spoken voice
TAC3	3	Anxiety; Echolalia; Mutism
TACC1	3	Anxiety; Echolalia; Unresponsiveness to spoken voice
TACSTD2	3	Echolalia; Language disorder; Motor delay
TAF10	3	Anxiety; Echolalia; Mutism
TAF1L	3	Aggressive behaviour; Echolalia; Unresponsiveness to spoken voice
TAF6L	3	Aggressive behaviour; Echolalia; Language disorder
TANC1	3	Echolalia; Language disorder; Motor delay
TARS2	3	Echolalia; Language disorder; Motor delay
TBATA	3	Echolalia; Motor delay; Unresponsiveness to spoken voice
TBC1D26	3	Echolalia; Motor delay; Unresponsiveness to spoken voice
TBC1D3B	3	Echolalia; Motor delay; Unresponsiveness to spoken voice
TBC1D3D	3	Echolalia; Motor delay; Unresponsiveness to spoken voice
TBC1D3G	3	Echolalia; Motor delay; Unresponsiveness to spoken voice
TBC1D3H	3	Echolalia; Motor delay; Unresponsiveness to spoken voice
TBC1D3I	3	Echolalia; Motor delay; Unresponsiveness to spoken voice
TBC1D3L	3	Echolalia; Motor delay; Unresponsiveness to spoken voice

TBCE	3	Aggressive behaviour; Echolalia; Unresponsiveness to spoken voice
TCEANC	3	Echolalia; Sleep disturbances; Unresponsiveness to spoken voice
TCERG1L	3	Anxiety; Echolalia; Mutism
TCF15	3	Anxiety; Echolalia; Mutism
TCFL5	3	Echolalia; Language disorder; Motor delay
TCP11	3	Aggressive behaviour; Echolalia; Unresponsiveness to spoken voice
TCTN1	3	Echolalia; Motor delay; Unresponsiveness to spoken voice
TDG	3	Echolalia; Sleep disturbances; Unresponsiveness to spoken voice
TDRD10	3	Echolalia; Mutism; Unresponsiveness to spoken voice
TDRD7	3	Anxiety; Echolalia; Mutism
TDRP	3	Anxiety; Echolalia; Mutism
TEF	3	Echolalia; Language disorder; Motor delay
TEKT4	3	Echolalia; Language disorder; Motor delay
TENM1	3	Echolalia; Language disorder; Motor delay
TERF1	3	Anxiety; Echolalia; Mutism
TEX28	3	Echolalia; Language disorder; Motor delay
TFDP3	3	Anxiety; Echolalia; Mutism
THNSL1	3	Echolalia; Mutism; Unresponsiveness to spoken voice
THSD4	3	Anxiety; Echolalia; Mutism
TIMM17A	3	Anxiety; Echolalia; Mutism
TIMP2	3	Echolalia; Motor delay; Unresponsiveness to spoken voice
TJAP1	3	Echolalia; Sleep disturbances; Unresponsiveness to spoken voice
TLCD3B	3	Echolalia; Motor delay; Unresponsiveness to spoken voice
TLE7	3	Echolalia; Sleep disturbances; Unresponsiveness to spoken voice
TMEFF1	3	Echolalia; Sleep disturbances; Unresponsiveness to spoken voice
TMEM121B	3	Echolalia; Language disorder; Motor delay
TMEM134	3	Echolalia; Language disorder; Motor delay
TMEM135	3	Aggressive behaviour; Echolalia; Motor delay
TMEM144	3	Echolalia; Language disorder; Motor delay
TMEM145	3	Anxiety; Echolalia; Unresponsiveness to spoken voice
TMEM177	3	Echolalia; Motor delay; Unresponsiveness to spoken voice
TMEM191B	3	Anxiety; Echolalia; Mutism
TMEM191C	3	Echolalia; Mutism; Unresponsiveness to spoken voice
TMEM201	3	Anxiety; Echolalia; Unresponsiveness to spoken voice
TMEM205	3	Anxiety; Mutism; Sleep disturbances
TMEM214	3	Echolalia; Motor delay; Unresponsiveness to spoken voice
TMEM241	3	Echolalia; Sleep disturbances; Unresponsiveness to spoken voice
TMEM26	3	Echolalia; Language disorder; Motor delay
TMEM270	3	Echolalia; Language disorder; Motor delay
TMEM38B	3	Echolalia; Language disorder; Motor delay
TMEM45A	3	Echolalia; Language disorder; Motor delay
TMEM63C	3	Anxiety; Echolalia; Mutism
TMEM67	3	Anxiety; Echolalia; Sleep disturbances
TMEM92	3	Aggressive behaviour; Echolalia; Unresponsiveness to spoken voice
TMPRSS15	3	Aggressive behaviour; Echolalia; Unresponsiveness to spoken voice
TNFAIP8	3	Echolalia; Motor delay; Unresponsiveness to spoken voice

TNFRSF10A	3	Echolalia; Language disorder; Motor delay
TNFRSF11A	3	Echolalia; Language disorder; Motor delay
TNFSF12	3	Echolalia; Motor delay; Unresponsiveness to spoken voice
TNFSF13B	3	Anxiety; Echolalia; Mutism
TOR1AIP1	3	Digestive disorder; Echolalia; Sleep disturbances
TP73	3	Echolalia; Language disorder; Motor delay
TPRN	3	Aggressive behaviour; Echolalia; Unresponsiveness to spoken voice
TRAF3IP3	3	Echolalia; Motor delay; Unresponsiveness to spoken voice
TRAFD1	3	Aggressive behaviour; Echolalia; Unresponsiveness to spoken voice
TREX2	3	Anxiety; Echolalia; Mutism
TRG-AS1	3	Echolalia; Motor delay; Unresponsiveness to spoken voice
TRIB1	3	Anxiety; Echolalia; Unresponsiveness to spoken voice
TRIM16L	3	Anxiety; Echolalia; Unresponsiveness to spoken voice
TRIM2	3	Echolalia; Language disorder; Motor delay
TRIM25	3	Echolalia; Language disorder; Motor delay
TRIM34	3	Anxiety; Echolalia; Mutism
TRIM46	3	Echolalia; Motor delay; Unresponsiveness to spoken voice
TRIM72	3	Echolalia; Sleep disturbances; Unresponsiveness to spoken voice
TRIM73	3	Anxiety; Echolalia; Mutism
TRIM74	3	Anxiety; Echolalia; Mutism
TRIM9	3	Anxiety; Echolalia; Mutism
TRIT1	3	Echolalia; Language disorder; Motor delay
TRPA1	3	Echolalia; Mutism; Unresponsiveness to spoken voice
TRPC4	3	Anxiety; Echolalia; Mutism
TRPC4AP	3	Echolalia; Language disorder; Motor delay
TRPM7	3	Echolalia; Motor delay; Unresponsiveness to spoken voice
TSHZ3	3	Echolalia; Language disorder; Motor delay
TSKU	3	Anxiety; Echolalia; Mutism
TTC3	3	Aggressive behaviour; Echolalia; Unresponsiveness to spoken voice
TTC34	3	Echolalia; Motor delay; Unresponsiveness to spoken voice
TTC6	3	Echolalia; Language disorder; Motor delay
TTK	3	Echolalia; Motor delay; Unresponsiveness to spoken voice
TTLL10	3	Anxiety; Echolalia; Mutism
TUBB3	3	Anxiety; Echolalia; Unresponsiveness to spoken voice
TXNDC2	3	Echolalia; Motor delay; Unresponsiveness to spoken voice
TYMP	3	Anxiety; Echolalia; Unresponsiveness to spoken voice
TYMSOS	3	Anxiety; Echolalia; Unresponsiveness to spoken voice
UBALD1	3	Echolalia; Language disorder; Motor delay
UBAP1L	3	Echolalia; Language disorder; Motor delay
UBE2N	3	Echolalia; Sleep disturbances; Unresponsiveness to spoken voice
UBE2Q2L	3	Aggressive behaviour; Anxiety; Echolalia
UBE2S	3	Echolalia; Language disorder; Motor delay
UBE3A	3	Echolalia; Sleep disturbances; Unresponsiveness to spoken voice
UBQLN4	3	Echolalia; Language disorder; Motor delay
UBR1	3	Echolalia; Language disorder; Motor delay
UCP1	3	Aggressive behaviour; Echolalia; Unresponsiveness to spoken voice

UCP2	3	Aggressive behaviour; Echolalia; Unresponsiveness to spoken voice
UGT1A6	3	Echolalia; Motor delay; Unresponsiveness to spoken voice
UGT1A7	3	Echolalia; Motor delay; Unresponsiveness to spoken voice
UGT1A9	3	Echolalia; Motor delay; Unresponsiveness to spoken voice
UGT2B7	3	Echolalia; Language disorder; Motor delay
UHRF1BP1	3	Anxiety; Echolalia; Mutism
UHRF2	3	Anxiety; Echolalia; Unresponsiveness to spoken voice
UNC80	3	Echolalia; Motor delay; Unresponsiveness to spoken voice
UNQ6494	3	Echolalia; Language disorder; Motor delay
USP17L17	3	Echolalia; Language disorder; Motor delay
USP17L4	3	Anxiety; Echolalia; Motor delay
USP17L6P	3	Echolalia; Language disorder; Motor delay
USP26	3	Aggressive behaviour; Echolalia; Unresponsiveness to spoken voice
USP30	3	Echolalia; Motor delay; Unresponsiveness to spoken voice
UTF1	3	Echolalia; Language disorder; Unresponsiveness to spoken voice
VASP	3	Echolalia; Language disorder; Motor delay
VAT1	3	Echolalia; Language disorder; Motor delay
VGLL2	3	Echolalia; Mutism; Unresponsiveness to spoken voice
VIRMA	3	Echolalia; Language disorder; Motor delay
VMO1	3	Echolalia; Sleep disturbances; Unresponsiveness to spoken voice
VN1R1	3	Echolalia; Motor delay; Unresponsiveness to spoken voice
VPS26A	3	Anxiety; Echolalia; Unresponsiveness to spoken voice
VRK2	3	Aggressive behaviour; Echolalia; Unresponsiveness to spoken voice
VRK3	3	Echolalia; Motor delay; Unresponsiveness to spoken voice
VSTM2B	3	Echolalia; Motor delay; Unresponsiveness to spoken voice
VTA1	3	Anxiety; Echolalia; Mutism
VWA5A	3	Echolalia; Sleep disturbances; Unresponsiveness to spoken voice
VWA5B2	3	Aggressive behaviour; Echolalia; Unresponsiveness to spoken voice
VWF	3	Anxiety; Echolalia; Unresponsiveness to spoken voice
WDR18	3	Echolalia; Motor delay; Unresponsiveness to spoken voice
WDR27	3	Echolalia; Language disorder; Motor delay
WDR3	3	Echolalia; Language disorder; Motor delay
WDR38	3	Echolalia; Language disorder; Motor delay
WDR4	3	Echolalia; Motor delay; Unresponsiveness to spoken voice
WDR45B	3	Anxiety; Echolalia; Unresponsiveness to spoken voice
WDR53	3	Anxiety; Echolalia; Mutism
WDR72	3	Echolalia; Motor delay; Unresponsiveness to spoken voice
WDR86	3	Echolalia; Motor delay; Unresponsiveness to spoken voice
WFDC5	3	Anxiety; Echolalia; Unresponsiveness to spoken voice
WIPF2	3	Echolalia; Language disorder; Mutism
WIPI1	3	Anxiety; Echolalia; Unresponsiveness to spoken voice
WNT6	3	Echolalia; Motor delay; Unresponsiveness to spoken voice
WT1	3	Aggressive behaviour; Echolalia; Unresponsiveness to spoken voice
WWC1	3	Aggressive behaviour; Echolalia; Unresponsiveness to spoken voice
XAB2	3	Aggressive behaviour; Anxiety; Echolalia
XKR3	3	Echolalia; Language disorder; Motor delay

**XPNPEP3**	3	Echolalia; Motor delay; Unresponsiveness to spoken voice
**XRCC4**	3	Anxiety; Echolalia; Mutism
**YTHDF1**	3	Aggressive behaviour; Echolalia; Motor delay
**ZBED3**	3	Echolalia; Motor delay; Unresponsiveness to spoken voice
**ZBTB1**	3	Anxiety; Echolalia; Unresponsiveness to spoken voice
**ZBTB2**	3	Echolalia; Language disorder; Motor delay
**ZBTB3**	3	Echolalia; Sleep disturbances; Unresponsiveness to spoken voice
**ZBTB38**	3	Aggressive behaviour; Echolalia; Unresponsiveness to spoken voice
**ZBTB41**	3	Anxiety; Echolalia; Mutism
**ZC3H18**	3	Aggressive behaviour; Echolalia; Unresponsiveness to spoken voice
**ZC3H7A**	3	Aggressive behaviour; Echolalia; Unresponsiveness to spoken voice
**ZCCHC2**	3	Echolalia; Mutism; Sleep disturbances
**ZDHHC6**	3	Echolalia; Sleep disturbances; Unresponsiveness to spoken voice
**ZFP14**	3	Echolalia; Language disorder; Unresponsiveness to spoken voice
**ZFP69B**	3	Echolalia; Mutism; Unresponsiveness to spoken voice
**ZFYVE28**	3	Aggressive behaviour; Echolalia; Unresponsiveness to spoken voice
**ZG16**	3	Echolalia; Motor delay; Unresponsiveness to spoken voice
**ZNF10**	3	Anxiety; Echolalia; Mutism
**ZNF135**	3	Echolalia; Sleep disturbances; Unresponsiveness to spoken voice
**ZNF155**	3	Anxiety; Echolalia; Motor delay
**ZNF160**	3	Aggressive behaviour; Echolalia; Language disorder
**ZNF200**	3	Echolalia; Sleep disturbances; Unresponsiveness to spoken voice
**ZNF217**	3	Echolalia; Language disorder; Motor delay
**ZNF219**	3	Anxiety; Echolalia; Unresponsiveness to spoken voice
**ZNF227**	3	Anxiety; Echolalia; Unresponsiveness to spoken voice
**ZNF229**	3	Aggressive behaviour; Echolalia; Unresponsiveness to spoken voice
**ZNF23**	3	Echolalia; Language disorder; Motor delay
**ZNF230**	3	Anxiety; Echolalia; Unresponsiveness to spoken voice
**ZNF254**	3	Aggressive behaviour; Echolalia; Mutism
**ZNF263**	3	Anxiety; Echolalia; Mutism
**ZNF264**	3	Aggressive behaviour; Echolalia; Motor delay
**ZNF276**	3	Echolalia; Mutism; Sleep disturbances
**ZNF277**	3	Echolalia; Language disorder; Motor delay
**ZNF281**	3	Anxiety; Echolalia; Mutism
**ZNF285**	3	Aggressive behaviour; Anxiety; Echolalia
**ZNF286B**	3	Anxiety; Echolalia; Mutism
**ZNF354A**	3	Echolalia; Motor delay; Unresponsiveness to spoken voice
**ZNF384**	3	Anxiety; Echolalia; Unresponsiveness to spoken voice
**ZNF385D-AS2**	3	Echolalia; Motor delay; Unresponsiveness to spoken voice
**ZNF395**	3	Echolalia; Mutism; Unresponsiveness to spoken voice
**ZNF417**	3	Aggressive behaviour; Anxiety; Echolalia
**ZNF449**	3	Anxiety; Echolalia; Mutism
**ZNF473**	3	Echolalia; Motor delay; Unresponsiveness to spoken voice
**ZNF502**	3	Anxiety; Echolalia; Unresponsiveness to spoken voice
**ZNF530**	3	Echolalia; Sleep disturbances; Unresponsiveness to spoken voice
**ZNF543**	3	Echolalia; Mutism; Unresponsiveness to spoken voice

ZNF549	3	Echolalia; Sleep disturbances; Unresponsiveness to spoken voice
ZNF571	3	Anxiety; Echolalia; Mutism
ZNF606	3	Aggressive behaviour; Echolalia; Language disorder
ZNF613	3	Echolalia; Sleep disturbances; Unresponsiveness to spoken voice
ZNF624	3	Echolalia; Sleep disturbances; Unresponsiveness to spoken voice
ZNF626	3	Aggressive behaviour; Echolalia; Motor delay
ZNF674	3	Echolalia; Motor delay; Unresponsiveness to spoken voice
ZNF675	3	Anxiety; Echolalia; Unresponsiveness to spoken voice
ZNF696	3	Echolalia; Language disorder; Motor delay
ZNF720	3	Echolalia; Motor delay; Unresponsiveness to spoken voice
ZNF76	3	Anxiety; Echolalia; Unresponsiveness to spoken voice
ZNF761	3	Echolalia; Language disorder; Motor delay
ZNF765-ZNF761	3	Echolalia; Language disorder; Motor delay
ZNF773	3	Anxiety; Echolalia; Sleep disturbances
ZNF783	3	Anxiety; Echolalia; Mutism
ZNF786	3	Echolalia; Language disorder; Motor delay
ZNF789	3	Anxiety; Echolalia; Mutism
ZNRF4	3	Anxiety; Echolalia; Unresponsiveness to spoken voice
ZPLD1	3	Anxiety; Echolalia; Unresponsiveness to spoken voice
ZSCAN2	3	Aggressive behaviour; Echolalia; Unresponsiveness to spoken voice
ZSCAN4	3	Echolalia; Language disorder; Motor delay
ZSCAN5A	3	Echolalia; Motor delay; Unresponsiveness to spoken voice
ZSWIM5	3	Digestive disorder; Echolalia; Motor delay
ZXDA	3	Echolalia; Sleep disturbances; Unresponsiveness to spoken voice
AANAT	2	Echolalia; Motor delay
AARSD1	2	Aggressive behaviour; Echolalia
AASDH	2	Echolalia; Unresponsiveness to spoken voice
AATK	2	Echolalia; Motor delay
ABAT	2	Echolalia; Motor delay
ABCA12	2	Aggressive behaviour; Echolalia
ABCA13	2	Echolalia; Unresponsiveness to spoken voice
ABCA17P	2	Echolalia; Motor delay
ABCA2	2	Echolalia; Unresponsiveness to spoken voice
ABCA9	2	Echolalia; Motor delay
ABCB6	2	Anxiety; Echolalia
ABCC1	2	Echolalia; Motor delay
ABCC11	2	Echolalia; Motor delay
ABCC2	2	Echolalia; Motor delay
ABCD3	2	Echolalia; Language disorder
ABCG1	2	Aggressive behaviour; Echolalia
ABCG5	2	Echolalia; Unresponsiveness to spoken voice
ABHD11	2	Anxiety; Mutism
ABHD12B	2	Echolalia; Language disorder
ABHD14A	2	Echolalia; Mutism
ABHD15	2	Anxiety; Echolalia
ABHD2	2	Motor delay; Unresponsiveness to spoken voice

ABL2	2	Anxiety; Echolalia
ABO	2	Echolalia; Unresponsiveness to spoken voice
ABR	2	Aggressive behaviour; Echolalia
ABTB1	2	Echolalia; Unresponsiveness to spoken voice
ACAD10	2	Motor delay; Unresponsiveness to spoken voice
ACADM	2	Echolalia; Language disorder
ACADSB	2	Echolalia; Motor delay
ACAN	2	Aggressive behaviour; Echolalia
ACBD4	2	Echolalia; Unresponsiveness to spoken voice
ACCS	2	Echolalia; Unresponsiveness to spoken voice
ACER2	2	Echolalia; Unresponsiveness to spoken voice
ACKR2	2	Aggressive behaviour; Echolalia
ACLY	2	Echolalia; Motor delay
ACMSD	2	Echolalia; Sleep disturbances
ACO1	2	Echolalia; Motor delay
ACOT1	2	Aggressive behaviour; Echolalia
ACOT12	2	Echolalia; Language disorder
ACOT2	2	Aggressive behaviour; Echolalia
ACOT6	2	Anxiety; Echolalia
ACOT7	2	Anxiety; Echolalia
ACOT9	2	Echolalia; Unresponsiveness to spoken voice
ACP1	2	Echolalia; Language disorder
ACP3	2	Aggressive behaviour; Echolalia
ACP4	2	Echolalia; Motor delay
ACP7	2	Echolalia; Unresponsiveness to spoken voice
ACSBG1	2	Aggressive behaviour; Echolalia
ACSL3	2	Echolalia; Motor delay
ACSL6	2	Digestive disorder; Echolalia
ACSM1	2	Echolalia; Language disorder
ACSM4	2	Echolalia; Unresponsiveness to spoken voice
ACSM5	2	Aggressive behaviour; Echolalia
ACSS2	2	Aggressive behaviour; Echolalia
ACTBL2	2	Echolalia; Sleep disturbances
ACTG2	2	Echolalia; Language disorder
ACTL8	2	Echolalia; Unresponsiveness to spoken voice
ACTL9	2	Digestive disorder; Echolalia
ACTN1	2	Echolalia; Unresponsiveness to spoken voice
ACTR1B	2	Echolalia; Motor delay
ACY3	2	Echolalia; Sleep disturbances
ADA	2	Echolalia; Mutism
ADAM11	2	Echolalia; Motor delay
ADAM15	2	Anxiety; Echolalia
ADAM17	2	Echolalia; Language disorder
ADAM19	2	Echolalia; Unresponsiveness to spoken voice
ADAM22	2	Aggressive behaviour; Echolalia
ADAM23	2	Anxiety; Echolalia

ADAM29	2	Echolalia; Unresponsiveness to spoken voice
ADAM30	2	Echolalia; Unresponsiveness to spoken voice
ADAM33	2	Anxiety; Echolalia
ADAMTS1	2	Echolalia; Motor delay
ADAMTS12	2	Echolalia; Motor delay
ADAMTS17	2	Aggressive behaviour; Echolalia
ADAMTS19	2	Echolalia; Language disorder
ADAMTS3	2	Anxiety; Echolalia
ADAMTS4	2	Echolalia; Unresponsiveness to spoken voice
ADAMTS5	2	Echolalia; Unresponsiveness to spoken voice
ADAMTS9	2	Aggressive behaviour; Echolalia
ADAMTSL1	2	Echolalia; Unresponsiveness to spoken voice
ADAMTSL2	2	Echolalia; Unresponsiveness to spoken voice
ADAP1	2	Echolalia; Unresponsiveness to spoken voice
ADAR	2	Anxiety; Echolalia
ADAT1	2	Echolalia; Motor delay
ADAT3	2	Anxiety; Echolalia
ADCK2	2	Echolalia; Language disorder
ADCK5	2	Anxiety; Echolalia
ADCY1	2	Echolalia; Motor delay
ADCY10	2	Echolalia; Sleep disturbances
ADCY5	2	Anxiety; Echolalia
ADCY7	2	Echolalia; Unresponsiveness to spoken voice
ADGB	2	Echolalia; Motor delay
ADGRB3	2	Echolalia; Unresponsiveness to spoken voice
ADGRE1	2	Anxiety; Echolalia
ADGRF1	2	Anxiety; Echolalia
ADGRF2	2	Echolalia; Language disorder
ADGRF3	2	Echolalia; Unresponsiveness to spoken voice
ADGRL4	2	Echolalia; Language disorder
ADGRV1	2	Aggressive behaviour; Echolalia
ADH1C	2	Aggressive behaviour; Echolalia
ADIPOQ	2	Anxiety; Echolalia
ADM5	2	Echolalia; Sleep disturbances
ADNP2	2	Anxiety; Echolalia
ADORA2A	2	Digestive disorder; Echolalia
ADPRH	2	Aggressive behaviour; Echolalia
ADRA1B	2	Aggressive behaviour; Echolalia
ADRM1	2	Echolalia; Unresponsiveness to spoken voice
ADSS1	2	Echolalia; Language disorder
AEBP2	2	Echolalia; Language disorder
AEN	2	Echolalia; Motor delay
AFAP1	2	Echolalia; Language disorder
AFF2	2	Aggressive behaviour; Echolalia
AFM	2	Aggressive behaviour; Echolalia
AFP	2	Echolalia; Mutism

AGA	2	Aggressive behaviour; Echolalia
AGAP11	2	Echolalia; Unresponsiveness to spoken voice
AGAP14P	2	Echolalia; Motor delay
AGAP5	2	Echolalia; Unresponsiveness to spoken voice
AGBL3	2	Aggressive behaviour; Echolalia
AGFG1	2	Echolalia; Motor delay
AGFG2	2	Echolalia; Motor delay
AHDC1	2	Anxiety; Echolalia
AHI1	2	Echolalia; Motor delay
AHNAK	2	Echolalia; Motor delay
AHR	2	Echolalia; Language disorder
AHRR	2	Echolalia; Unresponsiveness to spoken voice
AIPL1	2	Anxiety; Echolalia
AJAP1	2	Echolalia; Mutism
AJUBA	2	Aggressive behaviour; Echolalia
AK4	2	Echolalia; Unresponsiveness to spoken voice
AK8	2	Echolalia; Unresponsiveness to spoken voice
AKAP12	2	Echolalia; Language disorder
AKAP3	2	Echolalia; Language disorder
AKAP4	2	Echolalia; Language disorder
AKAP8	2	Echolalia; Mutism
AKNAD1	2	Echolalia; Language disorder
AKR1A1	2	Anxiety; Motor delay
AKR1C1	2	Anxiety; Echolalia
AKR1E2	2	Aggressive behaviour; Echolalia
AKR7L	2	Echolalia; Unresponsiveness to spoken voice
AKT1	2	Echolalia; Motor delay
ALAD	2	Echolalia; Unresponsiveness to spoken voice
ALDH16A1	2	Echolalia; Motor delay
ALDH1A2	2	Echolalia; Unresponsiveness to spoken voice
ALDH3A1	2	Echolalia; Mutism
ALDH3B1	2	Echolalia; Unresponsiveness to spoken voice
ALDH3B2	2	Anxiety; Motor delay
ALDH8A1	2	Aggressive behaviour; Echolalia
ALG1	2	Echolalia; Motor delay
ALG14	2	Language disorder; Mutism
ALG8	2	Echolalia; Language disorder
ALG9	2	Anxiety; Unresponsiveness to spoken voice
ALKBH1	2	Echolalia; Motor delay
ALKBH7	2	Echolalia; Motor delay
ALMS1	2	Anxiety; Echolalia
ALOX12-AS1	2	Language disorder; Motor delay
ALOX15B	2	Anxiety; Echolalia
ALPK1	2	Echolalia; Motor delay
ALPL	2	Echolalia; Motor delay
ALS2CL	2	Echolalia; Sleep disturbances

ALX1	2	Anxiety; Unresponsiveness to spoken voice
ALX4	2	Aggressive behaviour; Echolalia
ALYREF	2	Anxiety; Echolalia
AMACR	2	Echolalia; Motor delay
1,00 AMD	2	Anxiety; Echolalia
AMDHD2	2	Aggressive behaviour; Echolalia
AMELX	2	Aggressive behaviour; Echolalia
AMER2	2	Echolalia; Unresponsiveness to spoken voice
AMH	2	Echolalia; Motor delay
AMIGO2	2	Echolalia; Unresponsiveness to spoken voice
AMIGO3	2	Aggressive behaviour; Echolalia
AMN	2	Echolalia; Unresponsiveness to spoken voice
AMPD2	2	Aggressive behaviour; Echolalia
AMY1A	2	Echolalia; Language disorder
AMY1B	2	Echolalia; Language disorder
AMY1C	2	Echolalia; Language disorder
AMY2B	2	Aggressive behaviour; Echolalia
AMZ1	2	Anxiety; Echolalia
ANAPC1P6	2	Echolalia; Unresponsiveness to spoken voice
ANAPC5	2	Echolalia; Unresponsiveness to spoken voice
ANGEL1	2	Aggressive behaviour; Echolalia
ANGEL2	2	Anxiety; Unresponsiveness to spoken voice
ANGPTL2	2	Echolalia; Language disorder
ANK1	2	Aggressive behaviour; Echolalia
ANKAR	2	Aggressive behaviour; Echolalia
ANKDD1B	2	Echolalia; Motor delay
ANKEF1	2	Echolalia; Language disorder
ANKFN1	2	Aggressive behaviour; Echolalia
ANKH	2	Echolalia; Motor delay
ANKMY1	2	Echolalia; Language disorder
ANKRD10	2	Echolalia; Sleep disturbances
ANKRD13A	2	Echolalia; Sleep disturbances
ANKRD17	2	Echolalia; Language disorder
ANKRD18B	2	Echolalia; Unresponsiveness to spoken voice
ANKRD20A1	2	Echolalia; Unresponsiveness to spoken voice
ANKRD23	2	Echolalia; Unresponsiveness to spoken voice
ANKRD28	2	Motor delay; Unresponsiveness to spoken voice
ANKRD33	2	Echolalia; Language disorder
ANKRD34C	2	Anxiety; Mutism
ANKRD35	2	Echolalia; Language disorder
ANKRD42	2	Echolalia; Unresponsiveness to spoken voice
ANKRD52	2	Echolalia; Unresponsiveness to spoken voice
ANKRD60	2	Echolalia; Motor delay
ANKS1A	2	Echolalia; Unresponsiveness to spoken voice
ANKS3	2	Echolalia; Motor delay
ANKS6	2	Echolalia; Unresponsiveness to spoken voice

ANKUB1	2	Echolalia; Language disorder
ANO1	2	Echolalia; Sleep disturbances
ANO4	2	Echolalia; Unresponsiveness to spoken voice
ANO8	2	Echolalia; Unresponsiveness to spoken voice
ANOS1	2	Echolalia; Motor delay
ANP32C	2	Echolalia; Language disorder
ANPEP	2	Anxiety; Echolalia
ANTXR2	2	Aggressive behaviour; Echolalia
ANTXRL	2	Echolalia; Unresponsiveness to spoken voice
ANXA1	2	Aggressive behaviour; Echolalia
ANXA3	2	Echolalia; Motor delay
ANXA4	2	Aggressive behaviour; Echolalia
ANXA9	2	Echolalia; Mutism
AOC3	2	Anxiety; Echolalia
AOX1	2	Aggressive behaviour; Echolalia
AP1G1	2	Aggressive behaviour; Echolalia
AP1S3	2	Anxiety; Unresponsiveness to spoken voice
AP3D1	2	Aggressive behaviour; Echolalia
AP3M1	2	Digestive disorder; Echolalia
AP3S1	2	Echolalia; Unresponsiveness to spoken voice
AP4E1	2	Aggressive behaviour; Echolalia
AP5B1	2	Echolalia; Motor delay
APAF1	2	Echolalia; Unresponsiveness to spoken voice
APBA3	2	Aggressive behaviour; Echolalia
APBB3	2	Anxiety; Echolalia
APMAP	2	Echolalia; Unresponsiveness to spoken voice
APOA5	2	Echolalia; Motor delay
APOBEC3B-AS1	2	Echolalia; Sleep disturbances
APOBEC3C	2	Echolalia; Unresponsiveness to spoken voice
APOC4	2	Language disorder; Motor delay
APOE	2	Echolalia; Unresponsiveness to spoken voice
APOF	2	Anxiety; Echolalia
APOL6	2	Echolalia; Motor delay
AQP1	2	Echolalia; Unresponsiveness to spoken voice
AQP10	2	Echolalia; Language disorder
AQP11	2	Anxiety; Echolalia
AQP12A	2	Echolalia; Language disorder
AQP12B	2	Echolalia; Language disorder
AQR	2	Echolalia; Sleep disturbances
ARAP1	2	Echolalia; Mutism
ARAP3	2	Echolalia; Sleep disturbances
ARC	2	Aggressive behaviour; Echolalia
ARFGAP3	2	Motor delay; Mutism
ARFGEF2	2	Echolalia; Unresponsiveness to spoken voice
ARG2	2	Echolalia; Unresponsiveness to spoken voice
ARGFX	2	Echolalia; Motor delay

ARHGAP24	2	Echolalia; Motor delay
ARHGAP28	2	Echolalia; Unresponsiveness to spoken voice
ARHGAP29	2	Aggressive behaviour; Echolalia
ARHGAP30	2	Echolalia; Language disorder
ARHGAP42	2	Echolalia; Language disorder
ARHGAP45	2	Aggressive behaviour; Echolalia
ARHGAP5	2	Aggressive behaviour; Echolalia
ARHGAP6	2	Aggressive behaviour; Echolalia
ARHGAP9	2	Echolalia; Sleep disturbances
ARHGEF11	2	Aggressive behaviour; Echolalia
ARHGEF17	2	Echolalia; Unresponsiveness to spoken voice
ARHGEF18	2	Echolalia; Unresponsiveness to spoken voice
ARHGEF33	2	Anxiety; Echolalia
ARHGEF5	2	Echolalia; Unresponsiveness to spoken voice
ARID2	2	Aggressive behaviour; Echolalia
ARID3A	2	Echolalia; Motor delay
ARID3C	2	Echolalia; Language disorder
ARID4A	2	Echolalia; Sleep disturbances
ARID4B	2	Aggressive behaviour; Echolalia
ARL13A	2	Echolalia; Sleep disturbances
ARL14EPL	2	Aggressive behaviour; Echolalia
ARL5C	2	Anxiety; Echolalia
9,00 ARL	2	Echolalia; Unresponsiveness to spoken voice
ARMC10	2	Echolalia; Language disorder
ARMH4	2	Echolalia; Sleep disturbances
ARNT	2	Anxiety; Echolalia
ARNTL2	2	Echolalia; Unresponsiveness to spoken voice
ARSF	2	Echolalia; Language disorder
ARSH	2	Aggressive behaviour; Echolalia
ARSL	2	Echolalia; Language disorder
ART1	2	Echolalia; Unresponsiveness to spoken voice
ART4	2	Echolalia; Language disorder
ASAH1	2	Echolalia; Language disorder
ASAH2	2	Echolalia; Motor delay
ASAH2B	2	Echolalia; Language disorder
ASAP2	2	Echolalia; Unresponsiveness to spoken voice
ASB15	2	Echolalia; Unresponsiveness to spoken voice
ASCC3	2	Echolalia; Sleep disturbances
ASGR1	2	Echolalia; Unresponsiveness to spoken voice
ASIC3	2	Anxiety; Echolalia
ASIC4	2	Echolalia; Sleep disturbances
ASPRV1	2	Motor delay; Unresponsiveness to spoken voice
ASPSCR1	2	Aggressive behaviour; Echolalia
ASS1	2	Echolalia; Motor delay
ASTN2	2	Anxiety; Echolalia
ASXL1	2	Echolalia; Unresponsiveness to spoken voice

ASXL3	2	Anxiety; Echolalia
ASZ1	2	Echolalia; Language disorder
ATAD5	2	Echolalia; Mutism
ATF7	2	Anxiety; Echolalia
ATF7-NPFF	2	Anxiety; Echolalia
ATF7IP2	2	Echolalia; Language disorder
ATG12	2	Echolalia; Motor delay
ATG16L1	2	Echolalia; Sleep disturbances
ATG2A	2	Echolalia; Motor delay
ATG3	2	Echolalia; Motor delay
ATG4B	2	Echolalia; Motor delay
ATG5	2	Echolalia; Motor delay
ATG9A	2	Echolalia; Unresponsiveness to spoken voice
ATG9B	2	Aggressive behaviour; Echolalia
ATL1	2	Aggressive behaviour; Echolalia
ATL2	2	Aggressive behaviour; Echolalia
ATM	2	Aggressive behaviour; Echolalia
ATP10B	2	Anxiety; Echolalia
ATP11A	2	Echolalia; Language disorder
ATP13A1	2	Echolalia; Sleep disturbances
ATP13A4	2	Echolalia; Language disorder
ATP1A2	2	Echolalia; Unresponsiveness to spoken voice
ATP1B2	2	Echolalia; Mutism
ATP2A1	2	Echolalia; Motor delay
ATP2A3	2	Aggressive behaviour; Echolalia
ATP2B1	2	Echolalia; Unresponsiveness to spoken voice
ATP6AP2	2	Echolalia; Mutism
ATP6V0E2	2	Mutism; Sleep disturbances
ATP6V1C1	2	Anxiety; Echolalia
ATP6V1FNB	2	Echolalia; Motor delay
ATP7A	2	Aggressive behaviour; Echolalia
ATP8A1	2	Echolalia; Language disorder
ATP8B4	2	Anxiety; Echolalia
ATPAF1	2	Echolalia; Unresponsiveness to spoken voice
ATPAF2	2	Echolalia; Language disorder
ATRIP	2	Echolalia; Motor delay
ATXN2L	2	Echolalia; Motor delay
ATXN7	2	Echolalia; Sleep disturbances
ATXN7L1	2	Echolalia; Unresponsiveness to spoken voice
ATXN7L2	2	Aggressive behaviour; Anxiety
AUTS2	2	Aggressive behaviour; Echolalia
AVEN	2	Aggressive behaviour; Echolalia
AVIL	2	Echolalia; Unresponsiveness to spoken voice
AVL9	2	Echolalia; Language disorder
B3GALT5	2	Echolalia; Unresponsiveness to spoken voice
B3GNT2	2	Aggressive behaviour; Echolalia

B3GNT3	2	Echolalia; Unresponsiveness to spoken voice
B3GNT8	2	Echolalia; Unresponsiveness to spoken voice
B3GNTL1	2	Anxiety; Echolalia
B4GALT2	2	Echolalia; Sleep disturbances
B4GALT3	2	Echolalia; Unresponsiveness to spoken voice
B4GALT5	2	Aggressive behaviour; Echolalia
B4GALT6	2	Echolalia; Motor delay
BACE1	2	Echolalia; Unresponsiveness to spoken voice
BACH1	2	Aggressive behaviour; Echolalia
BACH2	2	Aggressive behaviour; Echolalia
BAG2	2	Echolalia; Motor delay
BAG3	2	Echolalia; Mutism
BAGE2	2	Language disorder; Motor delay
BAHD1	2	Echolalia; Language disorder
BAIAP2L1	2	Anxiety; Echolalia
BAIAP2L2	2	Echolalia; Unresponsiveness to spoken voice
BAIAP3	2	Echolalia; Unresponsiveness to spoken voice
BAK1	2	Aggressive behaviour; Echolalia
BANP	2	Echolalia; Sleep disturbances
BARX1	2	Echolalia; Unresponsiveness to spoken voice
BAX	2	Language disorder; Motor delay
BAZ1B	2	Echolalia; Language disorder
BAZ2A	2	Echolalia; Language disorder
BBOF1	2	Echolalia; Unresponsiveness to spoken voice
BBOX1	2	Echolalia; Unresponsiveness to spoken voice
BBS12	2	Echolalia; Motor delay
BBS4	2	Echolalia; Language disorder
BBS7	2	Echolalia; Sleep disturbances
BCAN	2	Echolalia; Language disorder
BCAR3	2	Echolalia; Motor delay
BCAS3	2	Aggressive behaviour; Echolalia
BCAS4	2	Echolalia; Motor delay
BCKDHB	2	Echolalia; Sleep disturbances
BCL11A	2	Echolalia; Motor delay
BCL2	2	Echolalia; Unresponsiveness to spoken voice
BCL6	2	Echolalia; Motor delay
BCO1	2	Echolalia; Sleep disturbances
BCO2	2	Echolalia; Motor delay
BCR	2	Echolalia; Sleep disturbances
BDH2	2	Anxiety; Echolalia
BDNF-AS	2	Echolalia; Motor delay
BEAN1	2	Echolalia; Unresponsiveness to spoken voice
BECN2	2	Echolalia; Motor delay
BEGAIN	2	Echolalia; Language disorder
BEND5	2	Aggressive behaviour; Echolalia
BEST1	2	Echolalia; Motor delay

BEST3	2	Echolalia; Motor delay
BFSP1	2	Echolalia; Language disorder
BFSP2	2	Echolalia; Language disorder
BHLHE22	2	Echolalia; Language disorder
BHLHE40	2	Echolalia; Motor delay
BHLHE41	2	Echolalia; Sleep disturbances
BHMT	2	Language disorder; Motor delay
BICDL2	2	Anxiety; Echolalia
BID	2	Anxiety; Echolalia
BIN2	2	Echolalia; Language disorder
BIRC6	2	Echolalia; Language disorder
BLM	2	Echolalia; Sleep disturbances
BLVRB	2	Echolalia; Unresponsiveness to spoken voice
BMP10	2	Anxiety; Echolalia
BMP15	2	Aggressive behaviour; Echolalia
BMP2	2	Digestive disorder; Echolalia
BMPER	2	Aggressive behaviour; Echolalia
BMS1	2	Echolalia; Sleep disturbances
BNC2	2	Anxiety; Echolalia
BNIP2	2	Aggressive behaviour; Echolalia
BNIP5	2	Echolalia; Unresponsiveness to spoken voice
BOD1	2	Echolalia; Sleep disturbances
BOD1L1	2	Echolalia; Motor delay
1,00 BOP	2	Anxiety; Echolalia
BPI	2	Aggressive behaviour; Echolalia
BPIFA3	2	Echolalia; Motor delay
BPIFB2	2	Aggressive behaviour; Echolalia
BPIFB3	2	Aggressive behaviour; Echolalia
BPIFC	2	Echolalia; Unresponsiveness to spoken voice
BRAF	2	Echolalia; Language disorder
BRAT1	2	Echolalia; Unresponsiveness to spoken voice
BRCA1	2	Anxiety; Echolalia
BRCA2	2	Anxiety; Echolalia
BRD3	2	Echolalia; Sleep disturbances
BRF1	2	Echolalia; Language disorder
BRICD5	2	Aggressive behaviour; Echolalia
BRIX1	2	Echolalia; Sleep disturbances
BRMS1	2	Echolalia; Sleep disturbances
BROX	2	Motor delay; Unresponsiveness to spoken voice
BRPF1	2	Aggressive behaviour; Echolalia
BRPF3	2	Echolalia; Unresponsiveness to spoken voice
BRSK1	2	Anxiety; Echolalia
BRSK2	2	Aggressive behaviour; Echolalia
BSCL2	2	Digestive disorder; Echolalia
BSPH1	2	Echolalia; Motor delay
BTAF1	2	Aggressive behaviour; Echolalia

BTC	2	Echolalia; Motor delay
BTF3	2	Echolalia; Unresponsiveness to spoken voice
BTG4	2	Echolalia; Unresponsiveness to spoken voice
BTK	2	Echolalia; Unresponsiveness to spoken voice
BTN2A1	2	Echolalia; Sleep disturbances
BTN2A2	2	Echolalia; Motor delay
BTN3A2	2	Echolalia; Language disorder
BTNL10	2	Echolalia; Unresponsiveness to spoken voice
BTRC	2	Echolalia; Unresponsiveness to spoken voice
BUB1B	2	Echolalia; Sleep disturbances
C10orf120	2	Echolalia; Motor delay
C10orf82	2	Echolalia; Unresponsiveness to spoken voice
C10orf90	2	Echolalia; Language disorder
C11orf96	2	Echolalia; Unresponsiveness to spoken voice
C11orf97	2	Aggressive behaviour; Echolalia
C12orf4	2	Language disorder; Mutism
C12orf40	2	Echolalia; Unresponsiveness to spoken voice
C12orf42	2	Digestive disorder; Echolalia
C12orf65	2	Echolalia; Sleep disturbances
C12orf73	2	Echolalia; Motor delay
C14orf177	2	Echolalia; Language disorder
C15orf48	2	Echolalia; Sleep disturbances
C16orf82	2	Digestive disorder; Echolalia
C16orf89	2	Echolalia; Language disorder
C17orf58	2	Echolalia; Motor delay
C17orf67	2	Echolalia; Sleep disturbances
C17orf77	2	Echolalia; Unresponsiveness to spoken voice
C17orf80	2	Echolalia; Language disorder
C17orf97	2	Echolalia; Language disorder
C17orf99	2	Echolalia; Mutism
C19orf38	2	Aggressive behaviour; Echolalia
C19orf47	2	Echolalia; Language disorder
C1QL2	2	Echolalia; Motor delay
C1QL4	2	Echolalia; Unresponsiveness to spoken voice
C1QTNF12	2	Echolalia; Language disorder
C1QTNF3-AMACR	2	Aggressive behaviour; Echolalia
C1RL	2	Echolalia; Language disorder
C1S	2	Echolalia; Unresponsiveness to spoken voice
C1orf127	2	Echolalia; Language disorder
C1orf232	2	Aggressive behaviour; Echolalia
C1orf52	2	Echolalia; Mutism
C1orf74	2	Echolalia; Language disorder
C1orf94	2	Echolalia; Unresponsiveness to spoken voice
C20orf194	2	Echolalia; Motor delay
C2CD2	2	Echolalia; Unresponsiveness to spoken voice

C2CD4A	2	Aggressive behaviour; Echolalia
C2CD5	2	Motor delay; Mutism
C2CD6	2	Echolalia; Unresponsiveness to spoken voice
C2orf50	2	Echolalia; Unresponsiveness to spoken voice
C2orf69	2	Echolalia; Unresponsiveness to spoken voice
C2orf73	2	Echolalia; Language disorder
C2orf76	2	Echolalia; Sleep disturbances
C2orf81	2	Echolalia; Sleep disturbances
C2orf83	2	Echolalia; Motor delay
C3	2	Anxiety; Echolalia
C3orf18	2	Echolalia; Unresponsiveness to spoken voice
C3orf35	2	Anxiety; Echolalia
C3orf70	2	Echolalia; Motor delay
C3orf84	2	Language disorder; Mutism
C4orf45	2	Aggressive behaviour; Echolalia
C5orf46	2	Echolalia; Language disorder
C5orf49	2	Echolalia; Mutism
C5orf52	2	Echolalia; Motor delay
C5orf60	2	Aggressive behaviour; Echolalia
C5orf63	2	Echolalia; Sleep disturbances
C5orf67	2	Aggressive behaviour; Echolalia
C6orf118	2	Echolalia; Unresponsiveness to spoken voice
C6orf120	2	Language disorder; Motor delay
C6orf132	2	Echolalia; Sleep disturbances
C6orf163	2	Aggressive behaviour; Echolalia
C6orf201	2	Echolalia; Unresponsiveness to spoken voice
C6orf52	2	Echolalia; Sleep disturbances
C7orf26	2	Echolalia; Unresponsiveness to spoken voice
C7orf61	2	Motor delay; Unresponsiveness to spoken voice
C8B	2	Aggressive behaviour; Echolalia
C8orf34	2	Aggressive behaviour; Echolalia
C8orf37	2	Echolalia; Unresponsiveness to spoken voice
C8orf82	2	Anxiety; Echolalia
C9orf139	2	Anxiety; Echolalia
C9orf24	2	Aggressive behaviour; Echolalia
C9orf57	2	Echolalia; Unresponsiveness to spoken voice
C9orf64	2	Anxiety; Echolalia
C9orf72	2	Echolalia; Mutism
CA5A	2	Echolalia; Unresponsiveness to spoken voice
CA8	2	Anxiety; Mutism
CABP2	2	Language disorder; Motor delay
CABYR	2	Anxiety; Echolalia
CACFD1	2	Echolalia; Motor delay
CACHD1	2	Echolalia; Language disorder
CACNA1A	2	Echolalia; Unresponsiveness to spoken voice
CACNA1G	2	Anxiety; Echolalia

CACNA1I	2	Aggressive behaviour; Echolalia
CACNA2D3	2	Anxiety; Echolalia
CACNB3	2	Echolalia; Sleep disturbances
CACNG2	2	Echolalia; Unresponsiveness to spoken voice
CACNG5	2	Aggressive behaviour; Echolalia
CACUL1	2	Anxiety; Unresponsiveness to spoken voice
CAD	2	Echolalia; Unresponsiveness to spoken voice
CADM3	2	Echolalia; Unresponsiveness to spoken voice
CADPS2	2	Echolalia; Language disorder
CALCOCO2	2	Echolalia; Unresponsiveness to spoken voice
CALR3	2	Echolalia; Unresponsiveness to spoken voice
CAMK1D	2	Echolalia; Unresponsiveness to spoken voice
CAMK2A	2	Echolalia; Sleep disturbances
CAMK2B	2	Echolalia; Motor delay
CAMK4	2	Echolalia; Motor delay
CANT1	2	Echolalia; Unresponsiveness to spoken voice
CANX	2	Echolalia; Unresponsiveness to spoken voice
CAP2	2	Echolalia; Language disorder
CAPN1	2	Digestive disorder; Echolalia
CAPN11	2	Echolalia; Motor delay
CAPN12	2	Echolalia; Motor delay
CAPN8	2	Echolalia; Motor delay
CAPRIN2	2	Echolalia; Unresponsiveness to spoken voice
CAPS2	2	Echolalia; Unresponsiveness to spoken voice
CAPZB	2	Aggressive behaviour; Echolalia
CARD14	2	Echolalia; Motor delay
CARD17	2	Echolalia; Sleep disturbances
CARD6	2	Aggressive behaviour; Echolalia
CARD8	2	Aggressive behaviour; Echolalia
CARD9	2	Aggressive behaviour; Echolalia
CARF	2	Echolalia; Unresponsiveness to spoken voice
CARMIL3	2	Anxiety; Echolalia
CASP1	2	Echolalia; Motor delay
CASP10	2	Echolalia; Unresponsiveness to spoken voice
CASP12	2	Aggressive behaviour; Echolalia
CASP2	2	Aggressive behaviour; Echolalia
CASP4	2	Aggressive behaviour; Echolalia
CASQ2	2	Echolalia; Unresponsiveness to spoken voice
CASR	2	Echolalia; Unresponsiveness to spoken voice
CASS4	2	Echolalia; Motor delay
CAST	2	Echolalia; Unresponsiveness to spoken voice
CASTOR1	2	Echolalia; Sleep disturbances
CASTOR3	2	Echolalia; Sleep disturbances
CATIP	2	Aggressive behaviour; Echolalia
CATSPER1	2	Echolalia; Sleep disturbances
CATSPER2	2	Echolalia; Motor delay

CAVIN3	2	Echolalia; Unresponsiveness to spoken voice
CBARP	2	Aggressive behaviour; Echolalia
CBFB	2	Aggressive behaviour; Echolalia
CBLB	2	Echolalia; Motor delay
CBR1	2	Echolalia; Sleep disturbances
CBR4	2	Anxiety; Echolalia
CBS	2	Aggressive behaviour; Echolalia
CBSL	2	Aggressive behaviour; Echolalia
CBWD2	2	Echolalia; Unresponsiveness to spoken voice
CBWD3	2	Echolalia; Unresponsiveness to spoken voice
CBWD5	2	Echolalia; Sleep disturbances
CBX8	2	Echolalia; Motor delay
CCDC102B	2	Aggressive behaviour; Echolalia
CCDC12	2	Echolalia; Unresponsiveness to spoken voice
CCDC122	2	Echolalia; Motor delay
CCDC136	2	Echolalia; Unresponsiveness to spoken voice
CCDC137	2	Anxiety; Echolalia
CCDC14	2	Aggressive behaviour; Echolalia
CCDC141	2	Echolalia; Language disorder
CCDC144A	2	Echolalia; Unresponsiveness to spoken voice
CCDC153	2	Anxiety; Echolalia
CCDC162P	2	Aggressive behaviour; Echolalia
CCDC170	2	Echolalia; Motor delay
CCDC171	2	Echolalia; Unresponsiveness to spoken voice
CCDC174	2	Echolalia; Motor delay
CCDC177	2	Aggressive behaviour; Echolalia
CCDC183	2	Aggressive behaviour; Echolalia
CCDC28A	2	Echolalia; Unresponsiveness to spoken voice
CCDC33	2	Aggressive behaviour; Echolalia
CCDC39	2	Echolalia; Unresponsiveness to spoken voice
CCDC40	2	Echolalia; Motor delay
CCDC42	2	Aggressive behaviour; Echolalia
CCDC47	2	Echolalia; Language disorder
CCDC54	2	Digestive disorder; Echolalia
CCDC68	2	Echolalia; Motor delay
CCDC71	2	Aggressive behaviour; Echolalia
CCDC74B	2	Echolalia; Motor delay
CCDC77	2	Aggressive behaviour; Echolalia
CCDC78	2	Anxiety; Echolalia
CCDC81	2	Echolalia; Unresponsiveness to spoken voice
CCDC85A	2	Echolalia; Unresponsiveness to spoken voice
CCDC86	2	Echolalia; Language disorder
CCDC88C	2	Anxiety; Echolalia
CCDC96	2	Aggressive behaviour; Echolalia
CCDC9B	2	Echolalia; Language disorder
CCER2	2	Aggressive behaviour; Echolalia

CCHCR1	2	Echolalia; Unresponsiveness to spoken voice
CCKAR	2	Aggressive behaviour; Echolalia
CCKBR	2	Aggressive behaviour; Echolalia
CCL20	2	Echolalia; Unresponsiveness to spoken voice
CCL26	2	Anxiety; Echolalia
CCM2L	2	Anxiety; Echolalia
CCN5	2	Anxiety; Echolalia
CCNA1	2	Echolalia; Unresponsiveness to spoken voice
CCNB1IP1	2	Motor delay; Unresponsiveness to spoken voice
CCNF	2	Echolalia; Language disorder
CCNH	2	Echolalia; Mutism
CCNI2	2	Echolalia; Unresponsiveness to spoken voice
CCNT1	2	Language disorder; Motor delay
CCP110	2	Echolalia; Motor delay
CCR4	2	Echolalia; Motor delay
CCR5AS	2	Aggressive behaviour; Echolalia
CCSER1	2	Echolalia; Sleep disturbances
CCSER2	2	Echolalia; Mutism
CCT4	2	Echolalia; Motor delay
CCT5	2	Echolalia; Motor delay
CCT6B	2	Aggressive behaviour; Echolalia
CCT7	2	Aggressive behaviour; Echolalia
CCT8	2	Echolalia; Unresponsiveness to spoken voice
CCZ1B	2	Aggressive behaviour; Echolalia
CD180	2	Aggressive behaviour; Echolalia
CD1C	2	Echolalia; Language disorder
CD1E	2	Aggressive behaviour; Echolalia
CD2	2	Aggressive behaviour; Echolalia
CD207	2	Echolalia; Unresponsiveness to spoken voice
CD24	2	Echolalia; Unresponsiveness to spoken voice
CD244	2	Anxiety; Echolalia
CD28	2	Echolalia; Language disorder
CD300A	2	Echolalia; Language disorder
CD300E	2	Echolalia; Unresponsiveness to spoken voice
CD300LD	2	Echolalia; Motor delay
CD300LG	2	Echolalia; Motor delay
CD302	2	Echolalia; Sleep disturbances
CD36	2	Mutism; Unresponsiveness to spoken voice
CD3E	2	Aggressive behaviour; Echolalia
CD4	2	Anxiety; Echolalia
CD40	2	Echolalia; Unresponsiveness to spoken voice
CD44	2	Echolalia; Sleep disturbances
CD58	2	Echolalia; Sleep disturbances
CD80	2	Anxiety; Echolalia
CD82	2	Echolalia; Motor delay
CD84	2	Echolalia; Unresponsiveness to spoken voice

CD8A	2	Echolalia; Mutism
CD8B	2	Echolalia; Mutism
CDC14B	2	Anxiety; Echolalia
CDC20	2	Aggressive behaviour; Echolalia
CDC20B	2	Echolalia; Unresponsiveness to spoken voice
CDC34	2	Echolalia; Language disorder
CDC37L1	2	Echolalia; Language disorder
CDC45	2	Echolalia; Unresponsiveness to spoken voice
CDC5L	2	Echolalia; Language disorder
CDC6	2	Aggressive behaviour; Echolalia
CDCA7L	2	Echolalia; Language disorder
CDCP1	2	Aggressive behaviour; Echolalia
CDCP2	2	Aggressive behaviour; Echolalia
CDH11	2	Aggressive behaviour; Echolalia
CDH15	2	Echolalia; Unresponsiveness to spoken voice
CDH17	2	Aggressive behaviour; Echolalia
CDH19	2	Echolalia; Motor delay
CDH2	2	Echolalia; Unresponsiveness to spoken voice
CDH5	2	Echolalia; Sleep disturbances
CDH8	2	Echolalia; Unresponsiveness to spoken voice
CDHR2	2	Echolalia; Motor delay
CDHR5	2	Echolalia; Language disorder
CDK11B	2	Echolalia; Unresponsiveness to spoken voice
CDK13	2	Echolalia; Motor delay
CDK16	2	Echolalia; Language disorder
CDK3	2	Echolalia; Unresponsiveness to spoken voice
CDK5RAP2	2	Aggressive behaviour; Echolalia
CDK9	2	Echolalia; Sleep disturbances
CDKL3	2	Echolalia; Language disorder
CDKN1B	2	Echolalia; Unresponsiveness to spoken voice
CDKN2AIP	2	Echolalia; Unresponsiveness to spoken voice
CDKN2B-AS1	2	Echolalia; Unresponsiveness to spoken voice
CDR2L	2	Echolalia; Language disorder
CEACAM5	2	Anxiety; Echolalia
CEBPA	2	Echolalia; Unresponsiveness to spoken voice
CECR2	2	Anxiety; Echolalia
CEL	2	Aggressive behaviour; Echolalia
CELA2B	2	Echolalia; Mutism
CELF5	2	Aggressive behaviour; Echolalia
CELSR2	2	Echolalia; Language disorder
CEMIP2	2	Anxiety; Echolalia
CENPJ	2	Echolalia; Unresponsiveness to spoken voice
CENPL	2	Aggressive behaviour; Echolalia
CENPO	2	Aggressive behaviour; Echolalia
CENPQ	2	Echolalia; Motor delay
CENPS-CORT	2	Anxiety; Motor delay

CENPT	2	Digestive disorder; Echolalia
CENPU	2	Aggressive behaviour; Echolalia
CEP112	2	Aggressive behaviour; Echolalia
CEP131	2	Anxiety; Echolalia
CEP192	2	Aggressive behaviour; Echolalia
CEP68	2	Aggressive behaviour; Echolalia
CEP76	2	Echolalia; Sleep disturbances
CEP78	2	Echolalia; Motor delay
CEP85L	2	Echolalia; Sleep disturbances
CER1	2	Echolalia; Motor delay
CERCAM	2	Aggressive behaviour; Echolalia
CERK	2	Echolalia; Language disorder
CERS1	2	Anxiety; Echolalia
CERS2	2	Echolalia; Unresponsiveness to spoken voice
CERS6	2	Anxiety; Echolalia
CES2	2	Aggressive behaviour; Echolalia
CFAP100	2	Echolalia; Mutism
CFAP36	2	Echolalia; Motor delay
CFAP44	2	Echolalia; Language disorder
CFAP53	2	Aggressive behaviour; Echolalia
CFAP58	2	Anxiety; Echolalia
CFAP65	2	Echolalia; Motor delay
CFAP69	2	Echolalia; Unresponsiveness to spoken voice
CFAP91	2	Aggressive behaviour; Echolalia
CFAP92	2	Echolalia; Unresponsiveness to spoken voice
CFAP94	2	Echolalia; Sleep disturbances
CFAP97	2	Echolalia; Language disorder
CFDP1	2	Echolalia; Sleep disturbances
CFHR3	2	Echolalia; Unresponsiveness to spoken voice
CFHR4	2	Echolalia; Unresponsiveness to spoken voice
CFLAR	2	Echolalia; Motor delay
CGB2	2	Echolalia; Motor delay
CGNL1	2	Echolalia; Language disorder
CGREF1	2	Echolalia; Unresponsiveness to spoken voice
CGRRF1	2	Echolalia; Sleep disturbances
CHCHD3	2	Echolalia; Mutism
CHCHD6	2	Echolalia; Unresponsiveness to spoken voice
CHD1L	2	Echolalia; Motor delay
CHD3	2	Echolalia; Sleep disturbances
CHD4	2	Aggressive behaviour; Echolalia
CHEK2P2	2	Echolalia; Sleep disturbances
CHERP	2	Echolalia; Sleep disturbances
CHI3L1	2	Echolalia; Language disorder
CHKB	2	Echolalia; Sleep disturbances
CHL1	2	Echolalia; Language disorder
CHL1-AS2	2	Echolalia; Unresponsiveness to spoken voice

CHMP1A	2	Anxiety; Echolalia
CHN1	2	Aggressive behaviour; Echolalia
CHN2	2	Echolalia; Sleep disturbances
CHORDC1	2	Echolalia; Unresponsiveness to spoken voice
CHPT1	2	Anxiety; Echolalia
CHRD	2	Echolalia; Motor delay
CHRDL2	2	Anxiety; Echolalia
CHRM5	2	Anxiety; Mutism
CHRNA3	2	Echolalia; Unresponsiveness to spoken voice
CHRNA5	2	Echolalia; Motor delay
CHRNB3	2	Echolalia; Sleep disturbances
CHRND	2	Anxiety; Echolalia
CHST1	2	Echolalia; Language disorder
CHST13	2	Echolalia; Unresponsiveness to spoken voice
CHST15	2	Echolalia; Motor delay
CHST8	2	Echolalia; Unresponsiveness to spoken voice
CHSY1	2	Aggressive behaviour; Echolalia
CHSY3	2	Echolalia; Unresponsiveness to spoken voice
CHUK	2	Echolalia; Motor delay
CHURC1-FNTB	2	Aggressive behaviour; Echolalia
CIART	2	Echolalia; Language disorder
CIB1	2	Echolalia; Unresponsiveness to spoken voice
CIB2	2	Anxiety; Echolalia
CINP	2	Echolalia; Sleep disturbances
CISH	2	Aggressive behaviour; Echolalia
CITED2	2	Echolalia; Motor delay
CITED4	2	Echolalia; Unresponsiveness to spoken voice
CKMT2	2	Echolalia; Sleep disturbances
CLASP2	2	Echolalia; Unresponsiveness to spoken voice
CLASRP	2	Echolalia; Language disorder
CLBA1	2	Anxiety; Echolalia
CLCA1	2	Echolalia; Motor delay
CLCF1	2	Language disorder; Motor delay
CLCN7	2	Echolalia; Language disorder
CLDN14	2	Echolalia; Unresponsiveness to spoken voice
CLDN3	2	Aggressive behaviour; Echolalia
CLEC17A	2	Echolalia; Motor delay
CLEC18C	2	Echolalia; Sleep disturbances
CLEC19A	2	Echolalia; Motor delay
CLEC2A	2	Echolalia; Unresponsiveness to spoken voice
CLEC2L	2	Echolalia; Sleep disturbances
CLEC3A	2	Echolalia; Unresponsiveness to spoken voice
CLEC4A	2	Aggressive behaviour; Echolalia
CLEC4O	2	Echolalia; Unresponsiveness to spoken voice
CLEC6A	2	Echolalia; Unresponsiveness to spoken voice
CLEC9A	2	Motor delay; Unresponsiveness to spoken voice

CLIC5	2	Echolalia; Motor delay
CLIP4	2	Aggressive behaviour; Echolalia
CLLU1	2	Echolalia; Unresponsiveness to spoken voice
CLLU1-AS1	2	Echolalia; Unresponsiveness to spoken voice
CLMN	2	Echolalia; Motor delay
CLMP	2	Echolalia; Sleep disturbances
CLN5	2	Aggressive behaviour; Echolalia
CLN6	2	Language disorder; Motor delay
CLPB	2	Echolalia; Language disorder
CLPS	2	Echolalia; Language disorder
CLPSL2	2	Echolalia; Sleep disturbances
CLPTM1	2	Echolalia; Unresponsiveness to spoken voice
CLSPN	2	Echolalia; Sleep disturbances
CLSTN1	2	Echolalia; Unresponsiveness to spoken voice
CLYBL	2	Echolalia; Unresponsiveness to spoken voice
CMA1	2	Language disorder; Motor delay
CMPK1	2	Echolalia; Language disorder
CMTM8	2	Echolalia; Unresponsiveness to spoken voice
CMYA5	2	Echolalia; Unresponsiveness to spoken voice
CNGA2	2	Echolalia; Motor delay
CNGB1	2	Echolalia; Motor delay
CNKSR1	2	Echolalia; Unresponsiveness to spoken voice
CNMD	2	Echolalia; Language disorder
CNNM3	2	Echolalia; Unresponsiveness to spoken voice
CNRIP1	2	Aggressive behaviour; Echolalia
CNTD1	2	Anxiety; Echolalia
CNTLN	2	Aggressive behaviour; Echolalia
CNTN5	2	Aggressive behaviour; Echolalia
CNTN6	2	Aggressive behaviour; Echolalia
CNTNAP1	2	Echolalia; Unresponsiveness to spoken voice
CNTNAP3B	2	Aggressive behaviour; Echolalia
CNTNAP5	2	Echolalia; Motor delay
CNTROB	2	Echolalia; Motor delay
COA3	2	Echolalia; Unresponsiveness to spoken voice
COA8	2	Echolalia; Sleep disturbances
COBL	2	Aggressive behaviour; Echolalia
COCH	2	Echolalia; Motor delay
COG1	2	Echolalia; Mutism
COG2	2	Echolalia; Motor delay
COG4	2	Digestive disorder; Echolalia
COG6	2	Anxiety; Echolalia
COG7	2	Aggressive behaviour; Echolalia
COL16A1	2	Anxiety; Echolalia
COL19A1	2	Aggressive behaviour; Echolalia
COL1A1	2	Echolalia; Mutism
COL21A1	2	Echolalia; Unresponsiveness to spoken voice

COL24A1	2	Aggressive behaviour; Echolalia
COL3A1	2	Echolalia; Motor delay
COL4A2	2	Echolalia; Sleep disturbances
COL5A2	2	Aggressive behaviour; Echolalia
COL6A5	2	Anxiety; Echolalia
COL6A6	2	Echolalia; Unresponsiveness to spoken voice
COL9A1	2	Echolalia; Motor delay
COL9A2	2	Echolalia; Unresponsiveness to spoken voice
COL9A3	2	Echolalia; Unresponsiveness to spoken voice
COLGALT1	2	Echolalia; Motor delay
COMMD1	2	Echolalia; Unresponsiveness to spoken voice
COMMD2	2	Echolalia; Motor delay
COMMD4	2	Anxiety; Echolalia
COMMD4P2	2	Echolalia; Motor delay
COMT	2	Echolalia; Language disorder
1 COP	2	Echolalia; Unresponsiveness to spoken voice
COPG1	2	Aggressive behaviour; Echolalia
COPS2	2	Echolalia; Language disorder
COPS7A	2	Echolalia; Unresponsiveness to spoken voice
COQ4	2	Echolalia; Language disorder
CORO6	2	Echolalia; Unresponsiveness to spoken voice
CORT	2	Anxiety; Motor delay
COX11	2	Anxiety; Echolalia
CPA1	2	Echolalia; Unresponsiveness to spoken voice
CPAMD8	2	Aggressive behaviour; Echolalia
CPB1	2	Echolalia; Unresponsiveness to spoken voice
CPB2	2	Echolalia; Language disorder
CPEB3	2	Echolalia; Unresponsiveness to spoken voice
CPED1	2	Echolalia; Unresponsiveness to spoken voice
CPLANE2	2	Language disorder; Motor delay
CPN1	2	Echolalia; Motor delay
CPNE6	2	Echolalia; Unresponsiveness to spoken voice
CPNE8	2	Echolalia; Language disorder
CPS1	2	Anxiety; Echolalia
CPSF4L	2	Aggressive behaviour; Echolalia
CPT1A	2	Echolalia; Language disorder
CPXM2	2	Echolalia; Unresponsiveness to spoken voice
CR1L	2	Echolalia; Mutism
CRABP1	2	Echolalia; Motor delay
CRACR2A	2	Echolalia; Unresponsiveness to spoken voice
CRACR2B	2	Echolalia; Language disorder
CRADD	2	Echolalia; Motor delay
CRAMP1	2	Echolalia; Unresponsiveness to spoken voice
CRBN	2	Echolalia; Unresponsiveness to spoken voice
CREB3	2	Aggressive behaviour; Echolalia
CREB3L1	2	Echolalia; Motor delay

CREBBP	2	Echolalia; Sleep disturbances
CREBZF	2	Echolalia; Motor delay
CRELD1	2	Echolalia; Motor delay
CRELD2	2	Aggressive behaviour; Echolalia
CRISP1	2	Echolalia; Unresponsiveness to spoken voice
CRISP2	2	Echolalia; Unresponsiveness to spoken voice
CRISP3	2	Echolalia; Motor delay
CRISPLD2	2	Aggressive behaviour; Echolalia
CRMP1	2	Anxiety; Echolalia
CRTC1	2	Echolalia; Unresponsiveness to spoken voice
CRY1	2	Anxiety; Echolalia
CRYAA	2	Aggressive behaviour; Echolalia
CRYAA2	2	Aggressive behaviour; Echolalia
CRYBG1	2	Echolalia; Unresponsiveness to spoken voice
CRYGN	2	Echolalia; Motor delay
CRYL1	2	Echolalia; Unresponsiveness to spoken voice
CRYZL2P-SEC16B	2	Anxiety; Echolalia
CSAG1	2	Echolalia; Sleep disturbances
CSF1	2	Echolalia; Motor delay
CSF1R	2	Anxiety; Echolalia
CSF2	2	Aggressive behaviour; Echolalia
CSF3R	2	Anxiety; Echolalia
CSGALNACT1	2	Echolalia; Language disorder
CSH2	2	Anxiety; Echolalia
CSHL1	2	Anxiety; Echolalia
CSKMT	2	Echolalia; Unresponsiveness to spoken voice
CSN3	2	Aggressive behaviour; Echolalia
CSNK1A1L	2	Anxiety; Echolalia
CSNK1G1	2	Anxiety; Echolalia
CSNKA2IP	2	Echolalia; Unresponsiveness to spoken voice
CSPG4	2	Echolalia; Motor delay
CSRNP1	2	Echolalia; Language disorder
CSRNP2	2	Echolalia; Sleep disturbances
CST11	2	Echolalia; Unresponsiveness to spoken voice
CST4	2	Aggressive behaviour; Echolalia
CST5	2	Echolalia; Motor delay
CSTL1	2	Echolalia; Motor delay
CTBP1	2	Echolalia; Mutism
CTBP2	2	Echolalia; Language disorder
CTCFL	2	Aggressive behaviour; Echolalia
CTD-2194D22.4	2	Aggressive behaviour; Echolalia
CTDP1	2	Aggressive behaviour; Echolalia
CTH	2	Echolalia; Sleep disturbances
CTNNA2	2	Echolalia; Mutism
CTNNA3	2	Echolalia; Mutism
CTNNAL1	2	Echolalia; Unresponsiveness to spoken voice

CTNND1	2	Aggressive behaviour; Echolalia
CTNS	2	Digestive disorder; Echolalia
CTPS1	2	Aggressive behaviour; Echolalia
CTRB2	2	Aggressive behaviour; Echolalia
CTRC	2	Language disorder; Motor delay
CTSB	2	Aggressive behaviour; Echolalia
CTSC	2	Echolalia; Mutism
CTSF	2	Language disorder; Motor delay
CTSS	2	Echolalia; Motor delay
CTSV	2	Echolalia; Sleep disturbances
CTSW	2	Echolalia; Sleep disturbances
CTXN3	2	Echolalia; Sleep disturbances
CUX2	2	Echolalia; Unresponsiveness to spoken voice
CXADR	2	Aggressive behaviour; Echolalia
CXCL12	2	Echolalia; Sleep disturbances
CXCL5	2	Echolalia; Motor delay
CXCR4	2	Echolalia; Unresponsiveness to spoken voice
CYB561	2	Echolalia; Motor delay
CYB5A	2	Language disorder; Motor delay
CYB5R3	2	Digestive disorder; Echolalia
CYBA	2	Echolalia; Motor delay
CYBRD1	2	Echolalia; Sleep disturbances
CYFIP2	2	Anxiety; Echolalia
CYHR1	2	Echolalia; Sleep disturbances
CYLC1	2	Motor delay; Unresponsiveness to spoken voice
CYP11B1	2	Echolalia; Sleep disturbances
CYP19A1	2	Anxiety; Echolalia
CYP1B1	2	Echolalia; Sleep disturbances
CYP26C1	2	Aggressive behaviour; Echolalia
CYP27A1	2	Echolalia; Language disorder
CYP27C1	2	Echolalia; Unresponsiveness to spoken voice
CYP2A6	2	Anxiety; Echolalia
CYP2B6	2	Anxiety; Echolalia
CYP2B7P	2	Echolalia; Unresponsiveness to spoken voice
CYP2C18	2	Anxiety; Echolalia
CYP2C8	2	Echolalia; Language disorder
20,00 CYP	2	Echolalia; Unresponsiveness to spoken voice
CYP2F1	2	Echolalia; Language disorder
CYP3A4	2	Echolalia; Unresponsiveness to spoken voice
CYP3A7	2	Echolalia; Motor delay
CYP4A11	2	Echolalia; Language disorder
CYP4A22	2	Echolalia; Language disorder
CYP4F12	2	Echolalia; Unresponsiveness to spoken voice
CYP4F22	2	Echolalia; Unresponsiveness to spoken voice
CYP8B1	2	Aggressive behaviour; Echolalia
CYS1	2	Aggressive behaviour; Echolalia

CYTH2	2	Aggressive behaviour; Echolalia
CYTL1	2	Echolalia; Language disorder
DAAM1	2	Echolalia; Motor delay
DAB2	2	Anxiety; Echolalia
DACH1	2	Aggressive behaviour; Echolalia
DACT3	2	Echolalia; Unresponsiveness to spoken voice
DAGLB	2	Echolalia; Mutism
DAPK1	2	Echolalia; Language disorder
DARS2	2	Echolalia; Mutism
DAW1	2	Echolalia; Unresponsiveness to spoken voice
DAZAP2	2	Aggressive behaviour; Echolalia
DBH	2	Echolalia; Sleep disturbances
DBI	2	Echolalia; Motor delay
DCAF13	2	Echolalia; Motor delay
DCAF8L1	2	Digestive disorder; Echolalia
DCAKD	2	Anxiety; Echolalia
DCBLD2	2	Echolalia; Unresponsiveness to spoken voice
DCHS1	2	Aggressive behaviour; Echolalia
DCHS2	2	Anxiety; Echolalia
DCLK2	2	Echolalia; Unresponsiveness to spoken voice
DCLRE1B	2	Aggressive behaviour; Echolalia
DCLRE1C	2	Aggressive behaviour; Echolalia
DCP1B	2	Aggressive behaviour; Echolalia
DCP2	2	Aggressive behaviour; Echolalia
DCPS	2	Echolalia; Unresponsiveness to spoken voice
DCTN1	2	Echolalia; Motor delay
DCTN4	2	Anxiety; Echolalia
DCUN1D3	2	Echolalia; Unresponsiveness to spoken voice
DCUN1D4	2	Echolalia; Mutism
DDB1	2	Echolalia; Unresponsiveness to spoken voice
DDB2	2	Aggressive behaviour; Motor delay
DDC	2	Echolalia; Motor delay
DDHD1	2	Echolalia; Unresponsiveness to spoken voice
DDI1	2	Echolalia; Unresponsiveness to spoken voice
DDOST	2	Echolalia; Motor delay
DDX19A	2	Aggressive behaviour; Echolalia
DDX21	2	Echolalia; Language disorder
DDX4	2	Anxiety; Echolalia
DDX43	2	Anxiety; Echolalia
DDX51	2	Echolalia; Unresponsiveness to spoken voice
DDX58	2	Echolalia; Mutism
DEF8	2	Aggressive behaviour; Echolalia
DEFB1	2	Echolalia; Unresponsiveness to spoken voice
DEFB108B	2	Echolalia; Unresponsiveness to spoken voice
DEFB118	2	Echolalia; Motor delay
DEFB119	2	Echolalia; Motor delay

DEFB127	2	Echolalia; Motor delay
DEFB132	2	Echolalia; Language disorder
DENND1B	2	Echolalia; Motor delay
DENND1C	2	Anxiety; Echolalia
DENND2A	2	Echolalia; Unresponsiveness to spoken voice
DENND2D	2	Aggressive behaviour; Echolalia
DENND3	2	Anxiety; Echolalia
DENND4A	2	Echolalia; Unresponsiveness to spoken voice
DEPDC4	2	Aggressive behaviour; Echolalia
DEPTOR	2	Echolalia; Language disorder
DERPC	2	Aggressive behaviour; Echolalia
DES	2	Echolalia; Motor delay
DEUP1	2	Echolalia; Unresponsiveness to spoken voice
DFFB	2	Aggressive behaviour; Echolalia
DGAT1	2	Echolalia; Motor delay
DGCR6L	2	Echolalia; Sleep disturbances
DGKG	2	Echolalia; Motor delay
DGKK	2	Echolalia; Motor delay
DGKZ	2	Echolalia; Motor delay
DHCR24	2	Anxiety; Mutism
DHRS7C	2	Echolalia; Mutism
DHRS9	2	Anxiety; Echolalia
DHX29	2	Echolalia; Unresponsiveness to spoken voice
DHX36	2	Echolalia; Sleep disturbances
DHX38	2	Echolalia; Language disorder
DHX40	2	Echolalia; Language disorder
DHX58	2	Echolalia; Unresponsiveness to spoken voice
DHX8	2	Echolalia; Motor delay
DIABLO	2	Aggressive behaviour; Echolalia
DIAPH1	2	Echolalia; Unresponsiveness to spoken voice
DIAPH2	2	Echolalia; Unresponsiveness to spoken voice
DIAPH3	2	Echolalia; Language disorder
DIPK1A	2	Motor delay; Unresponsiveness to spoken voice
DIPK1B	2	Echolalia; Language disorder
DIS3L	2	Echolalia; Mutism
DISC1	2	Echolalia; Motor delay
DISP1	2	Echolalia; Language disorder
DISP2	2	Echolalia; Language disorder
DIXDC1	2	Echolalia; Unresponsiveness to spoken voice
2,00 DKK	2	Echolalia; Sleep disturbances
3,00 DKK	2	Echolalia; Language disorder
DLAT	2	Anxiety; Echolalia
DLEC1	2	Echolalia; Unresponsiveness to spoken voice
DMAC2	2	Echolalia; Unresponsiveness to spoken voice
DMBT1	2	Echolalia; Motor delay
DMBT1L1	2	Aggressive behaviour; Echolalia

DMD	2	Echolalia; Unresponsiveness to spoken voice
DMRT2	2	Aggressive behaviour; Echolalia
DMWD	2	Echolalia; Unresponsiveness to spoken voice
DMXL2	2	Echolalia; Unresponsiveness to spoken voice
DNAAF1	2	Aggressive behaviour; Echolalia
DNAH10	2	Echolalia; Unresponsiveness to spoken voice
DNAH11	2	Echolalia; Unresponsiveness to spoken voice
DNAH14	2	Echolalia; Sleep disturbances
DNAH5	2	Echolalia; Motor delay
DNAH7	2	Aggressive behaviour; Echolalia
DNAI2	2	Echolalia; Language disorder
DNAJB8	2	Echolalia; Mutism
DNAJC15	2	Echolalia; Motor delay
DNAJC16	2	Anxiety; Echolalia
DNAJC18	2	Aggressive behaviour; Echolalia
DNAJC25	2	Aggressive behaviour; Echolalia
DNAJC30	2	Echolalia; Language disorder
DNAL4	2	Echolalia; Language disorder
DNASE1	2	Echolalia; Unresponsiveness to spoken voice
DNASE1L1	2	Anxiety; Echolalia
DNASE2B	2	Echolalia; Unresponsiveness to spoken voice
DNM1	2	Echolalia; Motor delay
DNMBP	2	Aggressive behaviour; Echolalia
DNPEP	2	Echolalia; Unresponsiveness to spoken voice
DOCK10	2	Aggressive behaviour; Echolalia
DOCK5	2	Echolalia; Unresponsiveness to spoken voice
DOK3	2	Echolalia; Motor delay
DOK6	2	Aggressive behaviour; Echolalia
DOK7	2	Aggressive behaviour; Echolalia
DOP1B	2	Echolalia; Unresponsiveness to spoken voice
DPCD	2	Echolalia; Mutism
DPF1	2	Echolalia; Language disorder
DPP10	2	Echolalia; Language disorder
DPP3	2	Anxiety; Echolalia
DPP7	2	Echolalia; Sleep disturbances
DPPA3	2	Aggressive behaviour; Echolalia
DPY19L2P3	2	Echolalia; Motor delay
DPY19L3	2	Language disorder; Motor delay
DPY19L4	2	Aggressive behaviour; Echolalia
DPYS	2	Echolalia; Unresponsiveness to spoken voice
DPYSL5	2	Aggressive behaviour; Echolalia
DRAXIN	2	Echolalia; Unresponsiveness to spoken voice
DRC1	2	Echolalia; Unresponsiveness to spoken voice
DRC3	2	Echolalia; Mutism
DRD2	2	Echolalia; Language disorder
DRD3	2	Echolalia; Unresponsiveness to spoken voice

DRD5	2	Digestive disorder; Echolalia
DSCR4	2	Echolalia; Sleep disturbances
DSG3	2	Aggressive behaviour; Echolalia
DSG4	2	Echolalia; Unresponsiveness to spoken voice
DST	2	Echolalia; Unresponsiveness to spoken voice
DTNB	2	Echolalia; Motor delay
DTNBP1	2	Echolalia; Language disorder
DTWD2	2	Anxiety; Echolalia
DTX3L	2	Aggressive behaviour; Echolalia
DUSP11	2	Aggressive behaviour; Echolalia
DUSP16	2	Echolalia; Unresponsiveness to spoken voice
DUSP2	2	Aggressive behaviour; Echolalia
DUSP22	2	Echolalia; Sleep disturbances
DUSP7	2	Motor delay; Unresponsiveness to spoken voice
DUXB	2	Echolalia; Motor delay
DVL1	2	Anxiety; Echolalia
DYM	2	Echolalia; Unresponsiveness to spoken voice
DYNC1LI1	2	Echolalia; Unresponsiveness to spoken voice
DYNC2I1	2	Anxiety; Echolalia
DYRK2	2	Echolalia; Unresponsiveness to spoken voice
DYRK4	2	Anxiety; Echolalia
DYTN	2	Anxiety; Echolalia
DZIP3	2	Echolalia; Unresponsiveness to spoken voice
E4F1	2	Echolalia; Unresponsiveness to spoken voice
EBLN1	2	Echolalia; Sleep disturbances
EBP	2	Echolalia; Motor delay
ECE1	2	Aggressive behaviour; Echolalia
ECE2	2	Language disorder; Motor delay
ECI2	2	Aggressive behaviour; Echolalia
ECM1	2	Anxiety; Echolalia
ECSIT	2	Echolalia; Mutism
ECT2L	2	Echolalia; Language disorder
EDAR	2	Echolalia; Language disorder
EDC4	2	Echolalia; Language disorder
EDN1	2	Echolalia; Sleep disturbances
EDN2	2	Aggressive behaviour; Echolalia
EDNRB	2	Echolalia; Motor delay
EDRF1	2	Echolalia; Mutism
EEF1A2	2	Echolalia; Mutism
EEF1AKMT4-ECE2	2	Language disorder; Motor delay
EEF1AKNMT	2	Echolalia; Unresponsiveness to spoken voice
EEF1B2	2	Anxiety; Mutism
EEF2KMT	2	Aggressive behaviour; Echolalia
EEFSEC	2	Echolalia; Motor delay
EEPD1	2	Aggressive behaviour; Echolalia

EFCAB10	2	Echolalia; Motor delay
EFCAB11	2	Echolalia; Unresponsiveness to spoken voice
EFCAB6	2	Anxiety; Echolalia
EFCAB8	2	Echolalia; Language disorder
EFHB	2	Aggressive behaviour; Echolalia
EFHC2	2	Echolalia; Unresponsiveness to spoken voice
EFNA2	2	Aggressive behaviour; Echolalia
EGFL7	2	Aggressive behaviour; Echolalia
EGFR	2	Echolalia; Unresponsiveness to spoken voice
EGR3	2	Echolalia; Motor delay
EHD2	2	Echolalia; Unresponsiveness to spoken voice
EHD3	2	Echolalia; Sleep disturbances
EHMT1	2	Echolalia; Motor delay
EID2	2	Echolalia; Motor delay
EIF2AK1	2	Echolalia; Mutism
EIF2B4	2	Motor delay; Unresponsiveness to spoken voice
EIF2S3B	2	Anxiety; Echolalia
EIF3B	2	Echolalia; Sleep disturbances
EIF3G	2	Anxiety; Motor delay
EIF4A1	2	Echolalia; Unresponsiveness to spoken voice
EIF4E1B	2	Aggressive behaviour; Echolalia
EIF5AL1	2	Echolalia; Unresponsiveness to spoken voice
ELAVL3	2	Echolalia; Language disorder
ELF3	2	Anxiety; Mutism
ELF4	2	Echolalia; Motor delay
ELFN2	2	Echolalia; Unresponsiveness to spoken voice
ELK3	2	Echolalia; Unresponsiveness to spoken voice
ELMO3	2	Echolalia; Unresponsiveness to spoken voice
ELOA2	2	Echolalia; Motor delay
ELOB	2	Echolalia; Sleep disturbances
ELOVL4	2	Aggressive behaviour; Echolalia
ELP5	2	Echolalia; Unresponsiveness to spoken voice
EMC1	2	Aggressive behaviour; Echolalia
EMC3	2	Echolalia; Language disorder
EME1	2	Echolalia; Mutism
EMID1	2	Echolalia; Unresponsiveness to spoken voice
EMILIN1	2	Aggressive behaviour; Echolalia
EMILIN2	2	Aggressive behaviour; Echolalia
EML2	2	Echolalia; Unresponsiveness to spoken voice
EML4	2	Echolalia; Motor delay
EMP2	2	Aggressive behaviour; Echolalia
EMSY	2	Aggressive behaviour; Echolalia
EN2	2	Aggressive behaviour; Echolalia
ENC1	2	Echolalia; Language disorder
ENDOG	2	Echolalia; Sleep disturbances
ENDOU	2	Sleep disturbances; Unresponsiveness to spoken voice

ENO1	2	Anxiety; Motor delay
ENO3	2	Echolalia; Sleep disturbances
ENO4	2	Anxiety; Echolalia
ENPP4	2	Echolalia; Sleep disturbances
ENPP6	2	Aggressive behaviour; Echolalia
ENTHD1	2	Echolalia; Unresponsiveness to spoken voice
ENTPD2	2	Aggressive behaviour; Echolalia
ENTPD6	2	Echolalia; Unresponsiveness to spoken voice
EOGT	2	Echolalia; Unresponsiveness to spoken voice
EPAS1	2	Echolalia; Mutism
EPB41L2	2	Echolalia; Language disorder
EPB41L4A	2	Anxiety; Echolalia
EPB41L4B	2	Echolalia; Motor delay
EPB42	2	Echolalia; Motor delay
EPDR1	2	Echolalia; Sleep disturbances
EPHA1	2	Echolalia; Unresponsiveness to spoken voice
EPHA2	2	Echolalia; Motor delay
EPHA4	2	Aggressive behaviour; Echolalia
EPHB1	2	Echolalia; Language disorder
EPN2	2	Echolalia; Unresponsiveness to spoken voice
EPN3	2	Aggressive behaviour; Echolalia
EPO	2	Mutism; Sleep disturbances
EPOR	2	Echolalia; Unresponsiveness to spoken voice
EPPK1	2	Aggressive behaviour; Echolalia
EPRS1	2	Echolalia; Unresponsiveness to spoken voice
EPS15L1	2	Anxiety; Echolalia
EPS8L3	2	Motor delay; Unresponsiveness to spoken voice
EPSTI1	2	Echolalia; Motor delay
EQTN	2	Aggressive behaviour; Echolalia
ERBB2	2	Aggressive behaviour; Echolalia
ERC1	2	Echolalia; Unresponsiveness to spoken voice
ERC2	2	Echolalia; Unresponsiveness to spoken voice
ERCC1	2	Anxiety; Echolalia
ERCC4	2	Echolalia; Motor delay
ERCC5	2	Aggressive behaviour; Echolalia
ERCC6	2	Aggressive behaviour; Echolalia
ERCC6L	2	Echolalia; Unresponsiveness to spoken voice
EREG	2	Echolalia; Unresponsiveness to spoken voice
ERF	2	Echolalia; Unresponsiveness to spoken voice
ERFL	2	Aggressive behaviour; Echolalia
ERI1	2	Echolalia; Unresponsiveness to spoken voice
ERICH2	2	Aggressive behaviour; Echolalia
ERICH3	2	Aggressive behaviour; Echolalia
ERICH3-AS1	2	Echolalia; Unresponsiveness to spoken voice
ERICH5	2	Echolalia; Unresponsiveness to spoken voice
ERLEC1	2	Aggressive behaviour; Echolalia

ERMP1	2	Echolalia; Language disorder
ERO1A	2	Echolalia; Language disorder
ERP27	2	Echolalia; Unresponsiveness to spoken voice
ERV3-1	2	Aggressive behaviour; Echolalia
ERV3-1-ZNF117	2	Aggressive behaviour; Echolalia
ERVH48-1	2	Echolalia; Motor delay
ERVMER34-1	2	Echolalia; Unresponsiveness to spoken voice
ESAM	2	Aggressive behaviour; Echolalia
ESPL1	2	Echolalia; Language disorder
ESPN	2	Echolalia; Language disorder
ESR1	2	Aggressive behaviour; Echolalia
ESX1	2	Anxiety; Echolalia
ESYT1	2	Language disorder; Motor delay
ESYT3	2	Echolalia; Unresponsiveness to spoken voice
ETHE1	2	Aggressive behaviour; Echolalia
ETNK1	2	Echolalia; Unresponsiveness to spoken voice
ETV1	2	Anxiety; Echolalia
EVA1C	2	Digestive disorder; Echolalia
EVC	2	Echolalia; Motor delay
EVI5	2	Echolalia; Motor delay
EVPLL	2	Echolalia; Motor delay
EXD2	2	Echolalia; Motor delay
EXO1	2	Echolalia; Motor delay
EXOC1	2	Echolalia; Unresponsiveness to spoken voice
EXOC3L2	2	Aggressive behaviour; Echolalia
EXOC7	2	Aggressive behaviour; Echolalia
EXOC8	2	Language disorder; Motor delay
EXOSC4	2	Aggressive behaviour; Echolalia
EXOSC6	2	Echolalia; Sleep disturbances
EXTL3	2	Echolalia; Language disorder
EZH2	2	Echolalia; Unresponsiveness to spoken voice
EZR	2	Motor delay; Unresponsiveness to spoken voice
F11	2	Anxiety; Echolalia
F2	2	Digestive disorder; Echolalia
F2RL1	2	Echolalia; Unresponsiveness to spoken voice
F3	2	Echolalia; Mutism
F5	2	Anxiety; Echolalia
F7	2	Echolalia; Unresponsiveness to spoken voice
FABP1	2	Aggressive behaviour; Echolalia
FAHD2CP	2	Echolalia; Unresponsiveness to spoken voice
FAM104A	2	Echolalia; Language disorder
FAM107B	2	Echolalia; Motor delay
FAM110D	2	Anxiety; Echolalia
FAM114A1	2	Echolalia; Unresponsiveness to spoken voice
FAM120AOS	2	Echolalia; Unresponsiveness to spoken voice
FAM122A	2	Aggressive behaviour; Echolalia

FAM122C	2	Echolalia; Unresponsiveness to spoken voice
FAM124B	2	Echolalia; Language disorder
FAM126A	2	Echolalia; Unresponsiveness to spoken voice
FAM13A	2	Echolalia; Language disorder
FAM13C	2	Echolalia; Motor delay
FAM151B	2	Anxiety; Echolalia
FAM155A	2	Anxiety; Echolalia
FAM155B	2	Aggressive behaviour; Echolalia
FAM157B	2	Aggressive behaviour; Echolalia
FAM161A	2	Aggressive behaviour; Echolalia
FAM167B	2	Aggressive behaviour; Echolalia
FAM168A	2	Echolalia; Unresponsiveness to spoken voice
FAM169A	2	Mutism; Sleep disturbances
FAM170A	2	Anxiety; Echolalia
FAM170B	2	Echolalia; Motor delay
FAM171A1	2	Aggressive behaviour; Echolalia
FAM171A2	2	Echolalia; Motor delay
FAM174A	2	Aggressive behaviour; Echolalia
FAM174B	2	Anxiety; Echolalia
FAM184B	2	Aggressive behaviour; Echolalia
FAM186A	2	Anxiety; Echolalia
FAM193A	2	Anxiety; Echolalia
FAM193B	2	Anxiety; Motor delay
FAM200B	2	Echolalia; Unresponsiveness to spoken voice
FAM205C	2	Echolalia; Sleep disturbances
FAM209B	2	Anxiety; Echolalia
FAM20B	2	Digestive disorder; Echolalia
FAM214A	2	Echolalia; Motor delay
FAM217A	2	Echolalia; Language disorder
FAM220A	2	Echolalia; Language disorder
FAM236C	2	Echolalia; Language disorder
FAM236D	2	Echolalia; Language disorder
FAM24B-CUZD1	2	Aggressive behaviour; Echolalia
FAM43A	2	Echolalia; Mutism
FAM47B	2	Anxiety; Echolalia
FAM47E-STBD1	2	Echolalia; Motor delay
FAM71A	2	Echolalia; Motor delay
FAM71C	2	Aggressive behaviour; Echolalia
FAM71D	2	Echolalia; Language disorder
FAM71E2	2	Anxiety; Echolalia
FAM72D	2	Echolalia; Motor delay
FAM78B	2	Echolalia; Motor delay
FAM81A	2	Echolalia; Unresponsiveness to spoken voice
FAM81B	2	Echolalia; Sleep disturbances
FAM83A	2	Anxiety; Echolalia
FAM83B	2	Echolalia; Mutism

FAM83C	2	Anxiety; Echolalia
FAM83F	2	Echolalia; Language disorder
FAM85A	2	Echolalia; Motor delay
FAM86B2	2	Echolalia; Language disorder
FAM95C	2	Aggressive behaviour; Echolalia
FANCC	2	Anxiety; Echolalia
FANCF	2	Echolalia; Language disorder
FANK1	2	Echolalia; Unresponsiveness to spoken voice
FARP2	2	Anxiety; Echolalia
FARS2	2	Echolalia; Mutism
FARSB	2	Aggressive behaviour; Echolalia
FASLG	2	Echolalia; Sleep disturbances
FASTKD5	2	Aggressive behaviour; Echolalia
FAXC	2	Anxiety; Echolalia
FBLN1	2	Echolalia; Sleep disturbances
FBLN5	2	Anxiety; Echolalia
FBLN7	2	Echolalia; Language disorder
FBN3	2	Aggressive behaviour; Echolalia
FBP1	2	Echolalia; Sleep disturbances
FBRS	2	Echolalia; Language disorder
FBXL12	2	Echolalia; Unresponsiveness to spoken voice
FBXL17	2	Echolalia; Unresponsiveness to spoken voice
FBXL4	2	Echolalia; Sleep disturbances
FBXL6	2	Anxiety; Echolalia
FBXO10	2	Digestive disorder; Echolalia
FBXO11	2	Digestive disorder; Echolalia
FBXO15	2	Echolalia; Language disorder
FBXO30	2	Echolalia; Motor delay
FBXO30-DT	2	Echolalia; Unresponsiveness to spoken voice
FBXO4	2	Anxiety; Motor delay
FBXO46	2	Aggressive behaviour; Echolalia
FBXO47	2	Echolalia; Motor delay
FBXO7	2	Echolalia; Language disorder
FBXW10	2	Echolalia; Unresponsiveness to spoken voice
FBXW12	2	Echolalia; Sleep disturbances
FBXW5	2	Aggressive behaviour; Echolalia
FBXW8	2	Echolalia; Language disorder
FBXW9	2	Echolalia; Language disorder
FCGBP	2	Echolalia; Unresponsiveness to spoken voice
FCGR2B	2	Anxiety; Echolalia
FCGR2C	2	Anxiety; Echolalia
FCGR3A	2	Anxiety; Echolalia
FCGRT	2	Anxiety; Echolalia
FCHO1	2	Echolalia; Sleep disturbances
FCRL1	2	Echolalia; Motor delay
FCRL5	2	Echolalia; Mutism

FCRLA	2	Echolalia; Sleep disturbances
FCSK	2	Echolalia; Sleep disturbances
FDFT1	2	Echolalia; Unresponsiveness to spoken voice
FDPS	2	Echolalia; Language disorder
FDX2	2	Echolalia; Unresponsiveness to spoken voice
FER	2	Anxiety; Echolalia
FERMT1	2	Echolalia; Language disorder
FEZ2	2	Aggressive behaviour; Echolalia
FEZF2	2	Echolalia; Language disorder
FGD4	2	Echolalia; Motor delay
FGF2	2	Echolalia; Sleep disturbances
FGF3	2	Language disorder; Motor delay
FGF9	2	Echolalia; Unresponsiveness to spoken voice
FGFBP1	2	Aggressive behaviour; Echolalia
FGFBP2	2	Anxiety; Echolalia
FGFBP3	2	Echolalia; Mutism
FGFRL1	2	Anxiety; Echolalia
FGL2	2	Anxiety; Echolalia
FH	2	Anxiety; Echolalia
FHDC1	2	Echolalia; Motor delay
FHOD1	2	Aggressive behaviour; Echolalia
FIBCD1	2	Language disorder; Motor delay
FICD	2	Echolalia; Unresponsiveness to spoken voice
FJX1	2	Echolalia; Mutism
FKBP15	2	Echolalia; Sleep disturbances
FKBP9	2	Echolalia; Unresponsiveness to spoken voice
FKTN	2	Anxiety; Echolalia
FLCN	2	Echolalia; Language disorder
FLG	2	Anxiety; Echolalia
FLII	2	Echolalia; Language disorder
FLJ46906	2	Echolalia; Unresponsiveness to spoken voice
FLNC	2	Echolalia; Motor delay
FLRT1	2	Anxiety; Echolalia
FLYWCH2	2	Aggressive behaviour; Echolalia
FMC1-LUC7L2	2	Echolalia; Language disorder
FN1	2	Aggressive behaviour; Echolalia
FNBP4	2	Anxiety; Echolalia
FNDC8	2	Echolalia; Motor delay
FNDC9	2	Echolalia; Motor delay
FNIP1	2	Anxiety; Echolalia
FOCAD	2	Echolalia; Unresponsiveness to spoken voice
FOLR3	2	Echolalia; Sleep disturbances
FOS	2	Echolalia; Motor delay
FOSL1	2	Echolalia; Sleep disturbances
FOSL2	2	Digestive disorder; Echolalia
FOXA2	2	Anxiety; Echolalia

FOXD1	2	Echolalia; Unresponsiveness to spoken voice
FOXD4L4	2	Echolalia; Mutism
FOXI1	2	Echolalia; Language disorder
FOXJ2	2	Echolalia; Sleep disturbances
FOXM1	2	Echolalia; Motor delay
FOXN1	2	Echolalia; Language disorder
FOXN2	2	Echolalia; Motor delay
FOXO1	2	Echolalia; Unresponsiveness to spoken voice
FOXO3B	2	Anxiety; Echolalia
FOXQ1	2	Echolalia; Language disorder
FOXRED2	2	Aggressive behaviour; Echolalia
FPGT	2	Echolalia; Unresponsiveness to spoken voice
FRAS1	2	Echolalia; Unresponsiveness to spoken voice
FRAT2	2	Echolalia; Sleep disturbances
FREM1	2	Echolalia; Unresponsiveness to spoken voice
FRG1BP	2	Anxiety; Echolalia
FRMD1	2	Echolalia; Unresponsiveness to spoken voice
FRMPD1	2	Echolalia; Unresponsiveness to spoken voice
FRMPD2	2	Echolalia; Language disorder
FRS2	2	Echolalia; Motor delay
FRS3	2	Anxiety; Echolalia
FRZB	2	Echolalia; Unresponsiveness to spoken voice
FSCB	2	Echolalia; Motor delay
FSCN2	2	Echolalia; Unresponsiveness to spoken voice
FSD1	2	Echolalia; Unresponsiveness to spoken voice
FSD2	2	Aggressive behaviour; Echolalia
FSIP2	2	Anxiety; Echolalia
FSTL4	2	Echolalia; Mutism
FTCDNL1	2	Anxiety; Echolalia
FTO	2	Echolalia; Motor delay
FUNDC1	2	Echolalia; Motor delay
FUS	2	Echolalia; Sleep disturbances
FUT5	2	Aggressive behaviour; Echolalia
FUT6	2	Anxiety; Echolalia
FUZ	2	Echolalia; Motor delay
FYB1	2	Aggressive behaviour; Echolalia
FYB2	2	Echolalia; Motor delay
FZD1	2	Aggressive behaviour; Echolalia
FZD6	2	Anxiety; Echolalia
FZD8	2	Echolalia; Unresponsiveness to spoken voice
GAA	2	Echolalia; Motor delay
GABRA5	2	Aggressive behaviour; Echolalia
GABRB3	2	Aggressive behaviour; Echolalia
GABRG2	2	Echolalia; Motor delay
GABRG3	2	Echolalia; Mutism
GABRR2	2	Echolalia; Motor delay

GAD1	2	Echolalia; Unresponsiveness to spoken voice
GADD45B	2	Aggressive behaviour; Echolalia
GAL3ST1	2	Echolalia; Motor delay
GAL3ST4	2	Echolalia; Language disorder
GALC	2	Echolalia; Language disorder
GALNT14	2	Aggressive behaviour; Echolalia
GALNT15	2	Echolalia; Motor delay
GALNT17	2	Echolalia; Sleep disturbances
GALNT2	2	Echolalia; Sleep disturbances
GALNT3	2	Echolalia; Sleep disturbances
GANAB	2	Echolalia; Sleep disturbances
GAPT	2	Echolalia; Sleep disturbances
GAREM2	2	Echolalia; Sleep disturbances
GARNL3	2	Echolalia; Unresponsiveness to spoken voice
GAS2	2	Aggressive behaviour; Echolalia
GAS2L3	2	Echolalia; Sleep disturbances
GAS8	2	Anxiety; Echolalia
GASK1A	2	Aggressive behaviour; Echolalia
GATA5	2	Aggressive behaviour; Echolalia
GBA2	2	Echolalia; Unresponsiveness to spoken voice
GBF1	2	Anxiety; Echolalia
GBGT1	2	Echolalia; Unresponsiveness to spoken voice
GBP1	2	Echolalia; Unresponsiveness to spoken voice
GBP4	2	Echolalia; Unresponsiveness to spoken voice
GBP6	2	Echolalia; Sleep disturbances
GC	2	Anxiety; Echolalia
GCC2	2	Echolalia; Language disorder
GCFC2	2	Digestive disorder; Echolalia
GCNT7	2	Echolalia; Motor delay
GCSAM	2	Language disorder; Motor delay
GDAP1L1	2	Echolalia; Unresponsiveness to spoken voice
GDF15	2	Echolalia; Language disorder
GDF6	2	Echolalia; Language disorder
GDI2	2	Sleep disturbances; Unresponsiveness to spoken voice
GDPD2	2	Echolalia; Language disorder
GEM	2	Echolalia; Unresponsiveness to spoken voice
GEN1	2	Anxiety; Echolalia
GFAP	2	Echolalia; Motor delay
GFI1	2	Echolalia; Mutism
GFM2	2	Anxiety; Echolalia
GFPT1	2	Echolalia; Motor delay
GFRA4	2	Echolalia; Motor delay
GGA1	2	Echolalia; Unresponsiveness to spoken voice
GGA2	2	Echolalia; Language disorder
GGA3	2	Aggressive behaviour; Echolalia
GGACT	2	Echolalia; Sleep disturbances

GGH	2	Echolalia; Motor delay
GGT2	2	Echolalia; Sleep disturbances
GGT5	2	Echolalia; Mutism
GGT6	2	Echolalia; Language disorder
GHRHR	2	Echolalia; Motor delay
GIGYF2	2	Aggressive behaviour; Echolalia
GIMAP1-GIMAP5	2	Echolalia; Unresponsiveness to spoken voice
GIMAP2	2	Anxiety; Echolalia
GINS2	2	Echolalia; Motor delay
GINS4	2	Echolalia; Unresponsiveness to spoken voice
GIPC3	2	Echolalia; Motor delay
GIT1	2	Echolalia; Sleep disturbances
GJA3	2	Echolalia; Unresponsiveness to spoken voice
GJA9	2	Echolalia; Language disorder
GJB4	2	Aggressive behaviour; Echolalia
GJB5	2	Echolalia; Motor delay
GK5	2	Echolalia; Language disorder
GLIPR1L2	2	Echolalia; Motor delay
GLIS3	2	Echolalia; Unresponsiveness to spoken voice
GLO1	2	Aggressive behaviour; Echolalia
GLOD4	2	Echolalia; Language disorder
GLOD5	2	Echolalia; Unresponsiveness to spoken voice
GLRA2	2	Echolalia; Unresponsiveness to spoken voice
GLRB	2	Aggressive behaviour; Echolalia
GLRX3	2	Echolalia; Motor delay
GLUL	2	Aggressive behaviour; Echolalia
GLYCTK	2	Digestive disorder; Echolalia
GMCL1	2	Aggressive behaviour; Echolalia
GMPPA	2	Aggressive behaviour; Echolalia
GMPR	2	Mutism; Unresponsiveness to spoken voice
GNAI1	2	Echolalia; Motor delay
GNAL	2	Aggressive behaviour; Echolalia
GNAS	2	Echolalia; Mutism
GNAT3	2	Echolalia; Motor delay
GNB1L	2	Anxiety; Echolalia
GNB3	2	Echolalia; Motor delay
GNB5	2	Anxiety; Echolalia
GNG7	2	Echolalia; Sleep disturbances
GNL3	2	Anxiety; Echolalia
GOLGA6C	2	Anxiety; Echolalia
GOLGA6L4	2	Anxiety; Echolalia
GOLGA6L9	2	Aggressive behaviour; Echolalia
GOLGA8A	2	Anxiety; Echolalia
GOLGA8B	2	Aggressive behaviour; Echolalia
GOLGA8EP	2	Aggressive behaviour; Echolalia
GOLGA8K	2	Echolalia; Motor delay

GOLGA8M	2	Echolalia; Mutism
GOLGA8O	2	Echolalia; Sleep disturbances
GOLIM4	2	Echolalia; Unresponsiveness to spoken voice
GON4L	2	Echolalia; Unresponsiveness to spoken voice
GOT2	2	Echolalia; Motor delay
GP1BA	2	Echolalia; Language disorder
GP5	2	Echolalia; Motor delay
GPATCH1	2	Echolalia; Unresponsiveness to spoken voice
GPATCH2L	2	Anxiety; Echolalia
GPC4	2	Anxiety; Echolalia
GPC6	2	Aggressive behaviour; Echolalia
GPIHBP1	2	Echolalia; Motor delay
GPM6A	2	Echolalia; Motor delay
GPNMB	2	Aggressive behaviour; Echolalia
GPR108	2	Anxiety; Echolalia
GPR139	2	Aggressive behaviour; Echolalia
GPR142	2	Aggressive behaviour; Echolalia
GPR148	2	Echolalia; Sleep disturbances
GPR153	2	Aggressive behaviour; Echolalia
GPR155	2	Aggressive behaviour; Echolalia
GPR156	2	Echolalia; Unresponsiveness to spoken voice
GPR160	2	Anxiety; Echolalia
GPR161	2	Echolalia; Unresponsiveness to spoken voice
GPR173	2	Echolalia; Sleep disturbances
GPR45	2	Anxiety; Echolalia
GPR61	2	Anxiety; Echolalia
GPR62	2	Echolalia; Mutism
GPR78	2	Echolalia; Language disorder
GPR85	2	Echolalia; Sleep disturbances
GPRC5C	2	Echolalia; Unresponsiveness to spoken voice
GPRC6A	2	Aggressive behaviour; Echolalia
GPRIN1	2	Echolalia; Unresponsiveness to spoken voice
GPRIN3	2	Echolalia; Unresponsiveness to spoken voice
GPX3	2	Echolalia; Motor delay
GPX4	2	Anxiety; Echolalia
GRAMD1A	2	Echolalia; Language disorder
GRAMD1C	2	Anxiety; Echolalia
GRAMD2B	2	Anxiety; Echolalia
GREB1	2	Anxiety; Echolalia
GRHL2	2	Echolalia; Unresponsiveness to spoken voice
GRIA2	2	Echolalia; Motor delay
GRID1	2	Aggressive behaviour; Echolalia
GRID2IP	2	Echolalia; Language disorder
GRIK2	2	Echolalia; Sleep disturbances
GRIK5	2	Echolalia; Motor delay
GRIN1	2	Echolalia; Sleep disturbances

GRIN3A	2	Echolalia; Motor delay
GRIN3B	2	Echolalia; Unresponsiveness to spoken voice
GRIP1	2	Aggressive behaviour; Echolalia
GRK5	2	Echolalia; Unresponsiveness to spoken voice
GRK6	2	Aggressive behaviour; Echolalia
GRM1	2	Aggressive behaviour; Echolalia
GRM8	2	Anxiety; Echolalia
GRN	2	Echolalia; Language disorder
GRP	2	Echolalia; Unresponsiveness to spoken voice
GRSF1	2	Aggressive behaviour; Echolalia
GRWD1	2	Echolalia; Motor delay
GRXCR1	2	Echolalia; Sleep disturbances
GRXCR2	2	Echolalia; Unresponsiveness to spoken voice
GSDMA	2	Echolalia; Unresponsiveness to spoken voice
GSDMB	2	Anxiety; Echolalia
GSDME	2	Echolalia; Unresponsiveness to spoken voice
GSE1	2	Echolalia; Unresponsiveness to spoken voice
GSR	2	Echolalia; Motor delay
GSS	2	Anxiety; Mutism
GSTA1	2	Aggressive behaviour; Echolalia
GSTA2	2	Echolalia; Mutism
GSTA5	2	Echolalia; Language disorder
GSTM4	2	Aggressive behaviour; Echolalia
GSTT2	2	Language disorder; Motor delay
GSTT2B	2	Language disorder; Motor delay
GSTZ1	2	Echolalia; Unresponsiveness to spoken voice
GTF2H5	2	Anxiety; Echolalia
GTF2I	2	Echolalia; Motor delay
GTF2IP7	2	Anxiety; Unresponsiveness to spoken voice
GTF3C1	2	Echolalia; Mutism
GTF3C4	2	Echolalia; Unresponsiveness to spoken voice
GTF3C5	2	Aggressive behaviour; Echolalia
GTPBP2	2	Anxiety; Echolalia
GTPBP3	2	Echolalia; Unresponsiveness to spoken voice
GTPBP4	2	Echolalia; Sleep disturbances
GTSCR1	2	Aggressive behaviour; Echolalia
GUCA1B	2	Anxiety; Unresponsiveness to spoken voice
GUCY2C	2	Echolalia; Motor delay
GYG1	2	Echolalia; Motor delay
GYG2	2	Echolalia; Language disorder
GZMA	2	Echolalia; Sleep disturbances
GZMB	2	Anxiety; Echolalia
H1-4	2	Aggressive behaviour; Echolalia
H1-6	2	Echolalia; Language disorder
H2AC15	2	Aggressive behaviour; Echolalia
H2BC12	2	Anxiety; Echolalia

H2BC6	2	Echolalia; Language disorder
H2BC9	2	Anxiety; Echolalia
H2BE1	2	Aggressive behaviour; Echolalia
H3C2	2	Anxiety; Echolalia
H3C8	2	Echolalia; Language disorder
H3Y1	2	Echolalia; Motor delay
H3Y2	2	Echolalia; Motor delay
H4C2	2	Aggressive behaviour; Echolalia
H4C6	2	Echolalia; Unresponsiveness to spoken voice
H4C8	2	Echolalia; Unresponsiveness to spoken voice
HABP4	2	Anxiety; Echolalia
HACD4	2	Aggressive behaviour; Echolalia
HADH	2	Echolalia; Language disorder
HADHA	2	Echolalia; Language disorder
HADHB	2	Aggressive behaviour; Echolalia
HAP1	2	Anxiety; Echolalia
HAPLN4	2	Echolalia; Sleep disturbances
HARS1	2	Echolalia; Unresponsiveness to spoken voice
HAS2	2	Aggressive behaviour; Echolalia
HASPIN	2	Echolalia; Motor delay
HAUS6	2	Anxiety; Echolalia
HAVCR2	2	Echolalia; Unresponsiveness to spoken voice
HBS1L	2	Anxiety; Echolalia
HCAR1	2	Echolalia; Language disorder
HCAR3	2	Echolalia; Motor delay
HCK	2	Echolalia; Unresponsiveness to spoken voice
HCLS1	2	Aggressive behaviour; Echolalia
HCN4	2	Echolalia; Sleep disturbances
HCST	2	Aggressive behaviour; Echolalia
HDAC10	2	Echolalia; Sleep disturbances
HDAC11	2	Echolalia; Unresponsiveness to spoken voice
HDC	2	Echolalia; Motor delay
HDGFL2	2	Echolalia; Mutism
HDGFL3	2	Echolalia; Language disorder
HDX	2	Echolalia; Motor delay
HEATR1	2	Echolalia; Motor delay
HEATR5B	2	Echolalia; Motor delay
HECA	2	Aggressive behaviour; Echolalia
HECTD2	2	Echolalia; Sleep disturbances
HECTD3	2	Echolalia; Motor delay
HECW1	2	Anxiety; Echolalia
HELQ	2	Anxiety; Echolalia
HENMT1	2	Echolalia; Unresponsiveness to spoken voice
HEPH	2	Echolalia; Motor delay
HERC2P7	2	Aggressive behaviour; Echolalia
HERC3	2	Echolalia; Motor delay

HES1	2	Aggressive behaviour; Echolalia
HES2	2	Echolalia; Sleep disturbances
HES7	2	Echolalia; Motor delay
HEXD	2	Echolalia; Unresponsiveness to spoken voice
HEXIM2	2	Aggressive behaviour; Echolalia
HGF	2	Echolalia; Motor delay
HGS	2	Anxiety; Echolalia
HHATL	2	Echolalia; Motor delay
HHIP	2	Echolalia; Sleep disturbances
HIF3A	2	Aggressive behaviour; Echolalia
HIGD1B	2	Aggressive behaviour; Echolalia
HIGD1C	2	Echolalia; Mutism
HINFP	2	Echolalia; Language disorder
HIP1R	2	Anxiety; Echolalia
HIPK2	2	Echolalia; Sleep disturbances
HIPK4	2	Aggressive behaviour; Echolalia
HIVEP1	2	Echolalia; Sleep disturbances
HIVEP2	2	Echolalia; Motor delay
HIVEP3	2	Echolalia; Unresponsiveness to spoken voice
HJURP	2	Anxiety; Echolalia
HJV	2	Echolalia; Unresponsiveness to spoken voice
HK1	2	Echolalia; Sleep disturbances
HK2	2	Aggressive behaviour; Echolalia
HLA-A	2	Echolalia; Unresponsiveness to spoken voice
HLA-B	2	Echolalia; Motor delay
HLTF	2	Aggressive behaviour; Echolalia
HMG20B	2	Echolalia; Language disorder
HMGCL	2	Aggressive behaviour; Echolalia
HMGN1	2	Echolalia; Motor delay
HMGXB3	2	Echolalia; Sleep disturbances
HMGXB4	2	Echolalia; Motor delay
HMMR	2	Aggressive behaviour; Echolalia
HNF4A	2	Aggressive behaviour; Echolalia
HNRNPA1L2	2	Echolalia; Unresponsiveness to spoken voice
HNRNPUL1	2	Echolalia; Mutism
HOGA1	2	Echolalia; Motor delay
HOMER1	2	Echolalia; Unresponsiveness to spoken voice
HOMER3	2	Echolalia; Unresponsiveness to spoken voice
HOMEZ	2	Aggressive behaviour; Echolalia
HOOK2	2	Echolalia; Unresponsiveness to spoken voice
HOXA7	2	Echolalia; Unresponsiveness to spoken voice
HOXA9	2	Anxiety; Unresponsiveness to spoken voice
HOXB1	2	Aggressive behaviour; Echolalia
HOXB13	2	Aggressive behaviour; Echolalia
HOXB2	2	Echolalia; Unresponsiveness to spoken voice
HOXB6	2	Motor delay; Unresponsiveness to spoken voice

HOXC11	2	Aggressive behaviour; Echolalia
HOXD1	2	Aggressive behaviour; Echolalia
HOXD11	2	Echolalia; Unresponsiveness to spoken voice
HP1BP3	2	Echolalia; Language disorder
HPD	2	Echolalia; Unresponsiveness to spoken voice
HPR	2	Echolalia; Language disorder
HPSE	2	Echolalia; Motor delay
HPX	2	Echolalia; Motor delay
HRCT1	2	Echolalia; Sleep disturbances
HRH1	2	Echolalia; Language disorder
HS1BP3	2	Anxiety; Echolalia
HS3ST2	2	Anxiety; Echolalia
HS3ST3A1	2	Aggressive behaviour; Echolalia
HS3ST3B1	2	Aggressive behaviour; Echolalia
HS3ST5	2	Echolalia; Unresponsiveness to spoken voice
HS6ST1	2	Echolalia; Language disorder
HSD11B1L	2	Echolalia; Unresponsiveness to spoken voice
HSD17B1	2	Echolalia; Motor delay
HSD17B11	2	Anxiety; Unresponsiveness to spoken voice
HSD17B12	2	Anxiety; Echolalia
HSD17B14	2	Echolalia; Language disorder
HSP90AA1	2	Echolalia; Unresponsiveness to spoken voice
HSP90AB1	2	Echolalia; Unresponsiveness to spoken voice
HSPA2	2	Echolalia; Motor delay
HSPA4L	2	Echolalia; Language disorder
HSPA6	2	Aggressive behaviour; Echolalia
HSPA8	2	Echolalia; Language disorder
HSPB3	2	Aggressive behaviour; Echolalia
HSPB7	2	Echolalia; Sleep disturbances
HSPH1	2	Aggressive behaviour; Echolalia
HTR1B	2	Aggressive behaviour; Echolalia
HTR3C	2	Echolalia; Motor delay
HTRA4	2	Aggressive behaviour; Echolalia
HUS1	2	Echolalia; Unresponsiveness to spoken voice
HUS1B	2	Anxiety; Echolalia
HUWE1	2	Echolalia; Motor delay
HYI	2	Aggressive behaviour; Echolalia
HYKK	2	Echolalia; Unresponsiveness to spoken voice
HYLS1	2	Aggressive behaviour; Echolalia
IAH1	2	Echolalia; Sleep disturbances
IAPP	2	Echolalia; Unresponsiveness to spoken voice
IARS1	2	Echolalia; Unresponsiveness to spoken voice
IBA57	2	Echolalia; Motor delay
IBTK	2	Echolalia; Unresponsiveness to spoken voice
ICAM3	2	Aggressive behaviour; Echolalia
ICE1	2	Echolalia; Unresponsiveness to spoken voice

ICE2	2	Anxiety; Echolalia
ID1	2	Anxiety; Mutism
IDH2	2	Anxiety; Echolalia
IFI44	2	Aggressive behaviour; Echolalia
IFIH1	2	Echolalia; Sleep disturbances
IFITM2	2	Echolalia; Language disorder
IFITM3	2	Aggressive behaviour; Echolalia
IFNA1	2	Echolalia; Motor delay
IFNE	2	Anxiety; Echolalia
IFNGR1	2	Echolalia; Unresponsiveness to spoken voice
IFNK	2	Anxiety; Echolalia
IFNL2	2	Echolalia; Unresponsiveness to spoken voice
IFT43	2	Echolalia; Unresponsiveness to spoken voice
IFT52	2	Aggressive behaviour; Echolalia
IFT57	2	Echolalia; Motor delay
IFT74	2	Echolalia; Unresponsiveness to spoken voice
IGF1R	2	Anxiety; Echolalia
IGF2BP1	2	Language disorder; Mutism
IGF2BP3	2	Echolalia; Unresponsiveness to spoken voice
IGFLR1	2	Echolalia; Unresponsiveness to spoken voice
IGFN1	2	Echolalia; Mutism
IGHV1OR15-3	2	Echolalia; Language disorder
IGLL1	2	Echolalia; Language disorder
IGSF11	2	Echolalia; Motor delay
IK	2	Anxiety; Echolalia
IKZF1	2	Echolalia; Language disorder
IL12RB2	2	Aggressive behaviour; Echolalia
IL13RA1	2	Echolalia; Unresponsiveness to spoken voice
IL17RB	2	Echolalia; Unresponsiveness to spoken voice
IL17RE	2	Echolalia; Unresponsiveness to spoken voice
IL18R1	2	Echolalia; Language disorder
IL1A	2	Echolalia; Unresponsiveness to spoken voice
IL1RL1	2	Aggressive behaviour; Echolalia
IL1RN	2	Echolalia; Motor delay
IL2	2	Anxiety; Echolalia
IL22RA1	2	Aggressive behaviour; Echolalia
IL23R	2	Echolalia; Mutism
IL27RA	2	Echolalia; Mutism
IL3	2	Anxiety; Echolalia
IL32	2	Echolalia; Unresponsiveness to spoken voice
IL36A	2	Aggressive behaviour; Echolalia
IL4	2	Aggressive behaviour; Echolalia
IL4I1	2	Aggressive behaviour; Echolalia
IL5RA	2	Echolalia; Language disorder
IL6-AS1	2	Echolalia; Motor delay
ILDR1	2	Aggressive behaviour; Echolalia

ILDR2	2	Aggressive behaviour; Echolalia
ILVBL	2	Echolalia; Mutism
IMMT	2	Echolalia; Unresponsiveness to spoken voice
IMP4	2	Echolalia; Motor delay
IMPDH1	2	Echolalia; Sleep disturbances
INAFM1	2	Aggressive behaviour; Echolalia
ING4	2	Echolalia; Unresponsiveness to spoken voice
INHA	2	Echolalia; Unresponsiveness to spoken voice
INMT	2	Echolalia; Unresponsiveness to spoken voice
INPP4A	2	Echolalia; Sleep disturbances
INPP5A	2	Echolalia; Unresponsiveness to spoken voice
INS	2	Aggressive behaviour; Echolalia
INS-IGF2	2	Aggressive behaviour; Echolalia
INSM1	2	Aggressive behaviour; Echolalia
INSM2	2	Aggressive behaviour; Echolalia
INSYN2A	2	Anxiety; Echolalia
INTS1	2	Echolalia; Mutism
INTS12	2	Echolalia; Language disorder
INTS4	2	Echolalia; Language disorder
INTS7	2	Motor delay; Unresponsiveness to spoken voice
IPO7	2	Aggressive behaviour; Echolalia
IPO8	2	Echolalia; Unresponsiveness to spoken voice
IQCA1L	2	Echolalia; Sleep disturbances
IQCE	2	Echolalia; Motor delay
IQCF1	2	Echolalia; Motor delay
IQCF2	2	Anxiety; Echolalia
IQCF6	2	Aggressive behaviour; Echolalia
IQCM	2	Echolalia; Unresponsiveness to spoken voice
IQGAP1	2	Anxiety; Echolalia
IQGAP2	2	Echolalia; Motor delay
IQSEC1	2	Echolalia; Unresponsiveness to spoken voice
IQUB	2	Anxiety; Echolalia
IRF2BP2	2	Echolalia; Unresponsiveness to spoken voice
IRF5	2	Language disorder; Motor delay
IRGM	2	Aggressive behaviour; Echolalia
IRS4	2	Echolalia; Motor delay
ISCU	2	Echolalia; Unresponsiveness to spoken voice
ISG15	2	Anxiety; Echolalia
ISG20L2	2	Echolalia; Language disorder
ISL1	2	Echolalia; Unresponsiveness to spoken voice
ISLR	2	Echolalia; Sleep disturbances
ISLR2	2	Aggressive behaviour; Echolalia
IST1	2	Echolalia; Mutism
ITCH	2	Anxiety; Echolalia
ITGA6	2	Aggressive behaviour; Echolalia
ITGA7	2	Echolalia; Unresponsiveness to spoken voice

ITGAD	2	Anxiety; Echolalia
ITGAE	2	Aggressive behaviour; Echolalia
ITGAV	2	Echolalia; Unresponsiveness to spoken voice
ITGB2	2	Echolalia; Motor delay
ITGB8	2	Echolalia; Mutism
ITGBL1	2	Echolalia; Sleep disturbances
ITIH1	2	Echolalia; Language disorder
ITIH2	2	Anxiety; Echolalia
ITLN1	2	Anxiety; Echolalia
ITLN2	2	Echolalia; Motor delay
ITPA	2	Echolalia; Unresponsiveness to spoken voice
ITPR1	2	Echolalia; Unresponsiveness to spoken voice
ITPRID1	2	Echolalia; Unresponsiveness to spoken voice
IVD	2	Echolalia; Language disorder
IVL	2	Echolalia; Unresponsiveness to spoken voice
IZUMO3	2	Aggressive behaviour; Echolalia
IZUMO4	2	Anxiety; Echolalia
JAK2	2	Anxiety; Echolalia
JMJD7	2	Echolalia; Sleep disturbances
JMJD7-PLA2G4B	2	Echolalia; Sleep disturbances
JPH3	2	Echolalia; Sleep disturbances
JSRP1	2	Echolalia; Sleep disturbances
JUN	2	Anxiety; Echolalia
KANK1	2	Echolalia; Sleep disturbances
KANK3	2	Echolalia; Motor delay
KANSL3	2	Anxiety; Echolalia
KARS1	2	Anxiety; Unresponsiveness to spoken voice
KASH5	2	Echolalia; Motor delay
KAT14	2	Echolalia; Unresponsiveness to spoken voice
KAT2B	2	Aggressive behaviour; Echolalia
KAT6A	2	Echolalia; Unresponsiveness to spoken voice
KATNIP	2	Echolalia; Motor delay
KBTBD13	2	Echolalia; Language disorder
KBTBD7	2	Echolalia; Sleep disturbances
KCNA10	2	Echolalia; Motor delay
KCNA2	2	Anxiety; Echolalia
KCNA4	2	Echolalia; Unresponsiveness to spoken voice
KCNAB2	2	Echolalia; Motor delay
KCNB2	2	Anxiety; Echolalia
KCNC4	2	Anxiety; Echolalia
KCNG1	2	Echolalia; Unresponsiveness to spoken voice
KCNG4	2	Echolalia; Sleep disturbances
KCNH2	2	Echolalia; Sleep disturbances
KCNH6	2	Echolalia; Unresponsiveness to spoken voice
KCNJ11	2	Echolalia; Unresponsiveness to spoken voice
KCNJ12	2	Echolalia; Motor delay

KCNJ13	2	Aggressive behaviour; Echolalia
KCNJ14	2	Anxiety; Echolalia
KCNJ15	2	Language disorder; Motor delay
KCNJ16	2	Anxiety; Sleep disturbances
KCNK13	2	Anxiety; Echolalia
KCNK18	2	Echolalia; Unresponsiveness to spoken voice
KCNK5	2	Aggressive behaviour; Echolalia
KCNMB2-AS1	2	Motor delay; Unresponsiveness to spoken voice
KCNMB3	2	Echolalia; Language disorder
KCNN3	2	Echolalia; Language disorder
KCNS1	2	Aggressive behaviour; Echolalia
KCNS3	2	Echolalia; Sleep disturbances
KCNT2	2	Anxiety; Echolalia
KCNU1	2	Echolalia; Motor delay
KCP	2	Anxiety; Echolalia
KCTD15	2	Echolalia; Unresponsiveness to spoken voice
KCTD18	2	Aggressive behaviour; Echolalia
KCTD7	2	Echolalia; Motor delay
KCTD9	2	Aggressive behaviour; Echolalia
KDM1A	2	Echolalia; Unresponsiveness to spoken voice
KDM2A	2	Echolalia; Unresponsiveness to spoken voice
KDM2B	2	Echolalia; Mutism
KDM3B	2	Aggressive behaviour; Echolalia
KDM5A	2	Echolalia; Motor delay
KDM7A	2	Motor delay; Unresponsiveness to spoken voice
KEAP1	2	Echolalia; Motor delay
KHDC1	2	Aggressive behaviour; Echolalia
KHDC1L	2	Anxiety; Echolalia
KHDC3L	2	Echolalia; Unresponsiveness to spoken voice
KHDRBS2	2	Echolalia; Motor delay
KIAA0100	2	Echolalia; Unresponsiveness to spoken voice
KIAA0319L	2	Aggressive behaviour; Echolalia
KIAA0408	2	Echolalia; Language disorder
KIAA0586	2	Echolalia; Motor delay
KIAA0753	2	Echolalia; Mutism
KIAA0825	2	Echolalia; Unresponsiveness to spoken voice
KIAA0895	2	Aggressive behaviour; Echolalia
KIAA1210	2	Anxiety; Echolalia
KIAA1217	2	Echolalia; Unresponsiveness to spoken voice
KIAA1549	2	Echolalia; Language disorder
KIAA1958	2	Anxiety; Echolalia
KIAA2013	2	Aggressive behaviour; Echolalia
KIF13B	2	Echolalia; Language disorder
KIF15	2	Anxiety; Echolalia
KIF17	2	Echolalia; Language disorder
KIF18A	2	Echolalia; Motor delay

KIF19	2	Echolalia; Language disorder
KIF1A	2	Anxiety; Echolalia
KIF1C	2	Anxiety; Echolalia
KIF25	2	Echolalia; Unresponsiveness to spoken voice
KIF26A	2	Anxiety; Echolalia
KIF3A	2	Echolalia; Unresponsiveness to spoken voice
KIF3B	2	Echolalia; Unresponsiveness to spoken voice
KIF4A	2	Echolalia; Unresponsiveness to spoken voice
KIF4B	2	Aggressive behaviour; Echolalia
KIRREL2	2	Anxiety; Echolalia
KIRREL3	2	Echolalia; Motor delay
KLF17	2	Echolalia; Motor delay
KLHDC10	2	Aggressive behaviour; Unresponsiveness to spoken voice
KLHDC7A	2	Echolalia; Language disorder
KLHDC7B	2	Echolalia; Unresponsiveness to spoken voice
KLHDC9	2	Aggressive behaviour; Echolalia
KLHL10	2	Echolalia; Sleep disturbances
KLHL11	2	Echolalia; Mutism
KLHL13	2	Echolalia; Unresponsiveness to spoken voice
KLHL24	2	Motor delay; Unresponsiveness to spoken voice
KLHL26	2	Echolalia; Motor delay
KLHL29	2	Aggressive behaviour; Echolalia
KLHL30	2	Aggressive behaviour; Echolalia
KLHL4	2	Echolalia; Sleep disturbances
KLK1	2	Echolalia; Language disorder
KLK7	2	Echolalia; Motor delay
KLKB1	2	Echolalia; Sleep disturbances
KLRC4-KLRK1	2	Echolalia; Unresponsiveness to spoken voice
KLRK1	2	Echolalia; Unresponsiveness to spoken voice
KMT2A	2	Echolalia; Sleep disturbances
KMT2B	2	Echolalia; Unresponsiveness to spoken voice
KMT2D	2	Echolalia; Unresponsiveness to spoken voice
KNOP1	2	Echolalia; Unresponsiveness to spoken voice
KNSTRN	2	Echolalia; Motor delay
KPNA3	2	Echolalia; Unresponsiveness to spoken voice
KPNB1	2	Aggressive behaviour; Echolalia
KPRP	2	Echolalia; Motor delay
KRBOX4	2	Anxiety; Unresponsiveness to spoken voice
KRI1	2	Echolalia; Motor delay
KRT1	2	Echolalia; Mutism
KRT14	2	Echolalia; Unresponsiveness to spoken voice
KRT26	2	Aggressive behaviour; Echolalia
KRT27	2	Echolalia; Unresponsiveness to spoken voice
KRT3	2	Echolalia; Motor delay
KRT4	2	Anxiety; Echolalia
KRT5	2	Echolalia; Language disorder

KRT6B	2	Echolalia; Mutism
KRT6C	2	Aggressive behaviour; Echolalia
KRT7	2	Anxiety; Echolalia
KRT71	2	Echolalia; Mutism
KRT76	2	Echolalia; Motor delay
KRT81	2	Echolalia; Unresponsiveness to spoken voice
KRTAP10-2	2	Echolalia; Unresponsiveness to spoken voice
KRTAP10-5	2	Echolalia; Unresponsiveness to spoken voice
KRTAP10-8	2	Motor delay; Unresponsiveness to spoken voice
KRTAP10-9	2	Echolalia; Unresponsiveness to spoken voice
KRTAP15-1	2	Digestive disorder; Motor delay
KRTAP22-1	2	Echolalia; Motor delay
KRTAP4-2	2	Echolalia; Language disorder
KRTAP4-3	2	Echolalia; Sleep disturbances
KRTAP4-4	2	Echolalia; Sleep disturbances
KRTAP4-6	2	Echolalia; Unresponsiveness to spoken voice
KRTAP5-11	2	Echolalia; Language disorder
KRTAP5-7	2	Echolalia; Unresponsiveness to spoken voice
KRTAP5-8	2	Echolalia; Unresponsiveness to spoken voice
KRTAP5-9	2	Echolalia; Language disorder
KTI12	2	Aggressive behaviour; Echolalia
KYAT3	2	Aggressive behaviour; Anxiety
L3MBTL2	2	Anxiety; Echolalia
LACTB	2	Aggressive behaviour; Echolalia
LACTBL1	2	Echolalia; Unresponsiveness to spoken voice
LAD1	2	Echolalia; Motor delay
LALBA	2	Echolalia; Unresponsiveness to spoken voice
LAMA3	2	Anxiety; Echolalia
LAMA4	2	Echolalia; Mutism
LAMB1	2	Echolalia; Unresponsiveness to spoken voice
LAMC1	2	Anxiety; Echolalia
LAMC2	2	Echolalia; Unresponsiveness to spoken voice
LAMP1	2	Anxiety; Echolalia
LAMP3	2	Echolalia; Motor delay
LANCL3	2	Aggressive behaviour; Echolalia
LAP3	2	Aggressive behaviour; Echolalia
LAPTM4B	2	Echolalia; Motor delay
LARS2	2	Echolalia; Mutism
LBH	2	Digestive disorder; Echolalia
LBHD2	2	Aggressive behaviour; Echolalia
LBP	2	Aggressive behaviour; Echolalia
LBR	2	Echolalia; Unresponsiveness to spoken voice
LBX2	2	Echolalia; Unresponsiveness to spoken voice
LCE3D	2	Echolalia; Motor delay
LCLAT1	2	Aggressive behaviour; Echolalia
LCMT1	2	Echolalia; Motor delay

LCMT2	2	Echolalia; Mutism
LCN8	2	Aggressive behaviour; Echolalia
LDB3	2	Anxiety; Echolalia
LDHD	2	Echolalia; Unresponsiveness to spoken voice
LEFTY1	2	Echolalia; Motor delay
LEKR1	2	Aggressive behaviour; Echolalia
LEXM	2	Aggressive behaviour; Echolalia
LFNG	2	Echolalia; Language disorder
LGALS14	2	Echolalia; Unresponsiveness to spoken voice
LGALS3BP	2	Aggressive behaviour; Echolalia
LGALS8	2	Echolalia; Motor delay
LGALS9	2	Echolalia; Language disorder
LGI1	2	Anxiety; Echolalia
LGR6	2	Aggressive behaviour; Echolalia
LHB	2	Aggressive behaviour; Echolalia
LHFPL5	2	Motor delay; Unresponsiveness to spoken voice
LHPP	2	Echolalia; Motor delay
LHX4	2	Echolalia; Unresponsiveness to spoken voice
LHX6	2	Mutism; Unresponsiveness to spoken voice
LHX9	2	Echolalia; Sleep disturbances
LIMCH1	2	Echolalia; Language disorder
LIMS1	2	Echolalia; Unresponsiveness to spoken voice
LIN7A	2	Motor delay; Unresponsiveness to spoken voice
LIN9	2	Aggressive behaviour; Echolalia
LINC00293	2	Echolalia; Language disorder
LINC00469	2	Echolalia; Language disorder
LINC00552	2	Aggressive behaviour; Echolalia
LINC00687	2	Aggressive behaviour; Echolalia
LINC01080	2	Aggressive behaviour; Echolalia
LINC01145	2	Echolalia; Motor delay
LINC01370	2	Echolalia; Unresponsiveness to spoken voice
LINC01597	2	Anxiety; Echolalia
LINC01770	2	Echolalia; Unresponsiveness to spoken voice
LINC01819	2	Anxiety; Echolalia
LINC01884	2	Aggressive behaviour; Echolalia
LINC02141	2	Echolalia; Unresponsiveness to spoken voice
LINC02490	2	Anxiety; Language disorder
LINC02578	2	Language disorder; Motor delay
LINC02580	2	Anxiety; Echolalia
LINC02860	2	Echolalia; Unresponsiveness to spoken voice
LINC02874	2	Anxiety; Echolalia
LINCR-0001	2	Echolalia; Motor delay
LINGO1	2	Aggressive behaviour; Echolalia
LIPI	2	Echolalia; Unresponsiveness to spoken voice
LIPK	2	Echolalia; Mutism
LIPN	2	Digestive disorder; Echolalia

LIPT2	2	Echolalia; Sleep disturbances
LITAF	2	Echolalia; Unresponsiveness to spoken voice
LMLN	2	Echolalia; Language disorder
LMNB1	2	Echolalia; Unresponsiveness to spoken voice
LMNB2	2	Echolalia; Language disorder
LMNTD2	2	Anxiety; Echolalia
LMO1	2	Aggressive behaviour; Echolalia
LMOD2	2	Echolalia; Sleep disturbances
LMOD3	2	Echolalia; Language disorder
LNP1	2	Echolalia; Sleep disturbances
LOC100128108	2	Echolalia; Language disorder
LOC100129484	2	Echolalia; Motor delay
LOC100130357	2	Echolalia; Motor delay
LOC100133286	2	Echolalia; Sleep disturbances
LOC100287846	2	Echolalia; Language disorder
LOC100287896	2	Echolalia; Unresponsiveness to spoken voice
LOC100289473	2	Aggressive behaviour; Echolalia
LOC100421372	2	Echolalia; Motor delay
LOC100505841	2	Aggressive behaviour; Echolalia
LOC100506207	2	Echolalia; Sleep disturbances
LOC100506858	2	Aggressive behaviour; Echolalia
LOC100996637	2	Echolalia; Unresponsiveness to spoken voice
LOC101927050	2	Aggressive behaviour; Echolalia
LOC101927322	2	Anxiety; Unresponsiveness to spoken voice
LOC101927509	2	Echolalia; Sleep disturbances
LOC101928416	2	Aggressive behaviour; Echolalia
LOC101928841	2	Echolalia; Language disorder
LOC101929294	2	Anxiety; Echolalia
LOC101929578	2	Anxiety; Echolalia
LOC102723730	2	Echolalia; Language disorder
LOC102723899	2	Anxiety; Echolalia
LOC102723996	2	Echolalia; Mutism
LOC102724788	2	Anxiety; Echolalia
LOC102725191	2	Aggressive behaviour; Echolalia
LOC105369140	2	Echolalia; Motor delay
LOC105369201	2	Echolalia; Motor delay
LOC105370295	2	Echolalia; Language disorder
LOC105370579	2	Echolalia; Unresponsiveness to spoken voice
LOC105375938	2	Echolalia; Sleep disturbances
LOC105378577	2	Echolalia; Motor delay
LOC105378949	2	Aggressive behaviour; Echolalia
LOC107984282	2	Echolalia; Language disorder
LOC107984507	2	Echolalia; Sleep disturbances
LOC107985728	2	Anxiety; Echolalia
LOC112268136	2	Echolalia; Motor delay
LOC339260	2	Echolalia; Motor delay

LOC339593	2	Aggressive behaviour; Echolalia
LOC374443	2	Echolalia; Unresponsiveness to spoken voice
LOC388780	2	Echolalia; Unresponsiveness to spoken voice
LOC389602	2	Aggressive behaviour; Echolalia
LOC400499	2	Anxiety; Echolalia
LOC403312	2	Echolalia; Motor delay
LOC408186	2	Echolalia; Sleep disturbances
LOC644215	2	Echolalia; Unresponsiveness to spoken voice
LOC645177	2	Echolalia; Unresponsiveness to spoken voice
LOC646588	2	Echolalia; Unresponsiveness to spoken voice
LOC653513	2	Aggressive behaviour; Echolalia
LOC728485	2	Aggressive behaviour; Echolalia
LOC729966	2	Echolalia; Unresponsiveness to spoken voice
LOC729968	2	Digestive disorder; Echolalia
LOC730234	2	Echolalia; Unresponsiveness to spoken voice
LOC780529	2	Echolalia; Motor delay
LOC91450	2	Echolalia; Sleep disturbances
LONP1	2	Echolalia; Sleep disturbances
LONP2	2	Echolalia; Sleep disturbances
LONRF2	2	Echolalia; Unresponsiveness to spoken voice
LONRF3	2	Echolalia; Unresponsiveness to spoken voice
LOX	2	Echolalia; Motor delay
LOXL1	2	Echolalia; Motor delay
LOXL3	2	Anxiety; Echolalia
LPAR2	2	Language disorder; Motor delay
LPCAT2	2	Aggressive behaviour; Echolalia
LPCAT4	2	Aggressive behaviour; Echolalia
LPIN2	2	Echolalia; Unresponsiveness to spoken voice
LRCH1	2	Aggressive behaviour; Echolalia
LRCH4	2	Echolalia; Language disorder
LRCOL1	2	Anxiety; Echolalia
LRFN1	2	Echolalia; Mutism
LRFN5	2	Echolalia; Unresponsiveness to spoken voice
LRG1	2	Aggressive behaviour; Echolalia
LRIG1	2	Echolalia; Unresponsiveness to spoken voice
LRIT2	2	Anxiety; Echolalia
LRIT3	2	Echolalia; Language disorder
LRP5	2	Digestive disorder; Echolalia
LRP6	2	Echolalia; Unresponsiveness to spoken voice
LRP8	2	Echolalia; Unresponsiveness to spoken voice
LRPAP1	2	Aggressive behaviour; Echolalia
LRRC14B	2	Echolalia; Unresponsiveness to spoken voice
LRRC15	2	Echolalia; Unresponsiveness to spoken voice
LRRC24	2	Anxiety; Echolalia
LRRC26	2	Echolalia; Motor delay
LRRC29	2	Aggressive behaviour; Echolalia

LRRC31	2	Aggressive behaviour; Echolalia
LRRC36	2	Echolalia; Motor delay
LRRC38	2	Echolalia; Motor delay
LRRC4	2	Echolalia; Motor delay
LRRC47	2	Echolalia; Sleep disturbances
LRRC4B	2	Echolalia; Language disorder
LRRC55	2	Echolalia; Unresponsiveness to spoken voice
LRRC69	2	Echolalia; Unresponsiveness to spoken voice
LRRC72	2	Echolalia; Language disorder
LRRC74A	2	Echolalia; Mutism
LRRC75B	2	Echolalia; Mutism
LRRC8C	2	Aggressive behaviour; Echolalia
LRRC9	2	Echolalia; Motor delay
LRRFIP2	2	Echolalia; Language disorder
LRRIQ4	2	Echolalia; Sleep disturbances
LRRK1	2	Anxiety; Echolalia
LRRK2	2	Anxiety; Echolalia
LRRN4	2	Anxiety; Echolalia
LRSAM1	2	Echolalia; Language disorder
LRTM2	2	Echolalia; Unresponsiveness to spoken voice
LSP1P4	2	Aggressive behaviour; Echolalia
LTBP3	2	Digestive disorder; Echolalia
LTBP4	2	Echolalia; Motor delay
LTC4S	2	Echolalia; Motor delay
LTF	2	Echolalia; Unresponsiveness to spoken voice
LUC7L2	2	Echolalia; Language disorder
LUZP1	2	Anxiety; Echolalia
LUZP4	2	Echolalia; Unresponsiveness to spoken voice
LVRN	2	Echolalia; Motor delay
LXN	2	Echolalia; Mutism
LY6K	2	Echolalia; Unresponsiveness to spoken voice
LY9	2	Aggressive behaviour; Echolalia
LYPD5	2	Echolalia; Language disorder
LYRM9	2	Anxiety; Echolalia
LYSMD3	2	Echolalia; Unresponsiveness to spoken voice
LYZL2	2	Motor delay; Unresponsiveness to spoken voice
LZTS3	2	Echolalia; Motor delay
MAB21L4	2	Echolalia; Unresponsiveness to spoken voice
MACIR	2	Echolalia; Unresponsiveness to spoken voice
MACROD2	2	Echolalia; Sleep disturbances
1,00 MAF	2	Anxiety; Echolalia
MAFA	2	Aggressive behaviour; Echolalia
MAGEE1	2	Aggressive behaviour; Echolalia
MAGEL2	2	Aggressive behaviour; Echolalia
MAGI1	2	Aggressive behaviour; Echolalia
MAGI2	2	Aggressive behaviour; Echolalia

MALL	2	Echolalia; Mutism
MAML1	2	Echolalia; Language disorder
MAML3	2	Echolalia; Unresponsiveness to spoken voice
MAMLD1	2	Aggressive behaviour; Echolalia
MAN1C1	2	Echolalia; Motor delay
MANEA	2	Aggressive behaviour; Echolalia
MANSC1	2	Echolalia; Unresponsiveness to spoken voice
MAOB	2	Mutism; Sleep disturbances
MAP1A	2	Echolalia; Unresponsiveness to spoken voice
MAP1S	2	Aggressive behaviour; Echolalia
MAP2K1	2	Echolalia; Mutism
MAP3K1	2	Echolalia; Motor delay
MAP3K13	2	Echolalia; Unresponsiveness to spoken voice
MAP3K19	2	Aggressive behaviour; Echolalia
MAP3K5	2	Echolalia; Language disorder
MAP7D1	2	Echolalia; Unresponsiveness to spoken voice
MAP7D2	2	Echolalia; Language disorder
MAP7D3	2	Echolalia; Unresponsiveness to spoken voice
MAP9	2	Echolalia; Unresponsiveness to spoken voice
MAPK13	2	Echolalia; Language disorder
MAPKAP1	2	Echolalia; Unresponsiveness to spoken voice
MARCHF2	2	Aggressive behaviour; Echolalia
MASP1	2	Echolalia; Sleep disturbances
MAST3	2	Anxiety; Echolalia
MATN2	2	Digestive disorder; Echolalia
MAU2	2	Echolalia; Unresponsiveness to spoken voice
MAZ	2	Echolalia; Unresponsiveness to spoken voice
MBD1	2	Aggressive behaviour; Echolalia
MBD3L3	2	Aggressive behaviour; Echolalia
MBD3L5	2	Aggressive behaviour; Echolalia
MBIP	2	Echolalia; Unresponsiveness to spoken voice
MBOAT2	2	Aggressive behaviour; Echolalia
MBTPS1	2	Digestive disorder; Echolalia
MC2R	2	Aggressive behaviour; Echolalia
MC4R	2	Aggressive behaviour; Echolalia
MCAM	2	Echolalia; Unresponsiveness to spoken voice
MCC	2	Anxiety; Echolalia
MCEMP1	2	Echolalia; Unresponsiveness to spoken voice
MCM2	2	Aggressive behaviour; Echolalia
MCM5	2	Aggressive behaviour; Echolalia
MCM6	2	Anxiety; Echolalia
MCM7	2	Echolalia; Unresponsiveness to spoken voice
MCMDC2	2	Echolalia; Language disorder
MCRS1	2	Echolalia; Unresponsiveness to spoken voice
MDK	2	Echolalia; Language disorder
MDM1	2	Echolalia; Unresponsiveness to spoken voice

ME1	2	Echolalia; Language disorder
MECR	2	Echolalia; Sleep disturbances
MED1	2	Anxiety; Unresponsiveness to spoken voice
MED24	2	Echolalia; Motor delay
MED25	2	Echolalia; Motor delay
MED26	2	Digestive disorder; Echolalia
MEF2D	2	Echolalia; Motor delay
MEIG1	2	Echolalia; Language disorder
MEIOSIN	2	Echolalia; Motor delay
MEP1B	2	Echolalia; Unresponsiveness to spoken voice
METAP2	2	Echolalia; Unresponsiveness to spoken voice
METTL16	2	Aggressive behaviour; Echolalia
METTL21A	2	Echolalia; Motor delay
METTL24	2	Aggressive behaviour; Echolalia
METTL25	2	Echolalia; Motor delay
MFAP3	2	Echolalia; Motor delay
MFN2	2	Echolalia; Motor delay
MFRP	2	Anxiety; Echolalia
MFSD10	2	Echolalia; Motor delay
MFSD14B	2	Aggressive behaviour; Echolalia
MFSD14C	2	Anxiety; Echolalia
MGA	2	Echolalia; Motor delay
MGAT1	2	Anxiety; Echolalia
MGAT2	2	Echolalia; Unresponsiveness to spoken voice
MGAT4D	2	Anxiety; Echolalia
MIA3	2	Aggressive behaviour; Echolalia
MIDEAS	2	Anxiety; Echolalia
MIEF2	2	Language disorder; Motor delay
MIER2	2	Echolalia; Motor delay
MIER3	2	Echolalia; Language disorder
MIMT1	2	Aggressive behaviour; Echolalia
MINDY1	2	Echolalia; Unresponsiveness to spoken voice
MINDY4	2	Aggressive behaviour; Echolalia
MIOS	2	Echolalia; Unresponsiveness to spoken voice
MIPEP	2	Aggressive behaviour; Echolalia
MIPOL1	2	Anxiety; Echolalia
MIR1-1HG	2	Echolalia; Motor delay
MIR3118-2	2	Echolalia; Language disorder
MIR4426	2	Echolalia; Unresponsiveness to spoken voice
MIR4771-1	2	Echolalia; Language disorder
MIR548I2	2	Digestive disorder; Echolalia
MIR650	2	Echolalia; Mutism
MIRLET7A1	2	Echolalia; Unresponsiveness to spoken voice
MISP	2	Aggressive behaviour; Echolalia
MKI67	2	Anxiety; Echolalia
MKNK1	2	Echolalia; Motor delay

MKX	2	Echolalia; Motor delay
MLANA	2	Anxiety; Echolalia
MLLT1	2	Anxiety; Echolalia
MMAB	2	Echolalia; Unresponsiveness to spoken voice
MMADHC	2	Echolalia; Motor delay
MMP10	2	Anxiety; Echolalia
MMP16	2	Echolalia; Motor delay
MMP19	2	Anxiety; Unresponsiveness to spoken voice
MMP9	2	Echolalia; Motor delay
MMUT	2	Aggressive behaviour; Echolalia
MN1	2	Echolalia; Unresponsiveness to spoken voice
MND1	2	Aggressive behaviour; Anxiety
MNX1	2	Aggressive behaviour; Echolalia
MOBP	2	Aggressive behaviour; Echolalia
MOCOS	2	Anxiety; Echolalia
MOCS2	2	Echolalia; Motor delay
MOGAT1	2	Anxiety; Echolalia
MOGS	2	Anxiety; Echolalia
MON1A	2	Aggressive behaviour; Echolalia
MON1B	2	Echolalia; Mutism
MON2	2	Echolalia; Language disorder
MORC2	2	Echolalia; Unresponsiveness to spoken voice
MORN2	2	Aggressive behaviour; Echolalia
MORN3	2	Echolalia; Sleep disturbances
MOXD1	2	Echolalia; Motor delay
MPEG1	2	Anxiety; Echolalia
MPHOSPH10	2	Echolalia; Motor delay
MPP2	2	Echolalia; Motor delay
MPP4	2	Anxiety; Echolalia
MPPE1	2	Echolalia; Motor delay
MPZL3	2	Aggressive behaviour; Echolalia
MR1	2	Anxiety; Echolalia
MRAP2	2	Anxiety; Echolalia
MRC2	2	Echolalia; Motor delay
MREG	2	Echolalia; Motor delay
MRGPRG	2	Aggressive behaviour; Echolalia
MRGPRX4	2	Aggressive behaviour; Echolalia
MRO	2	Echolalia; Sleep disturbances
MROH2B	2	Echolalia; Sleep disturbances
MROH7	2	Echolalia; Unresponsiveness to spoken voice
MROH8	2	Echolalia; Sleep disturbances
MRPL14	2	Echolalia; Unresponsiveness to spoken voice
MRPL16	2	Echolalia; Sleep disturbances
MRPL28	2	Anxiety; Echolalia
MRPL36	2	Echolalia; Unresponsiveness to spoken voice
MRPL37	2	Echolalia; Sleep disturbances

MRPL43	2	Aggressive behaviour; Echolalia
MRPL45	2	Echolalia; Motor delay
MRPL47	2	Echolalia; Sleep disturbances
MRPL50	2	Aggressive behaviour; Echolalia
MRPL52	2	Echolalia; Motor delay
MRPS18A	2	Echolalia; Unresponsiveness to spoken voice
MRPS21	2	Anxiety; Echolalia
MRPS27	2	Echolalia; Motor delay
MRPS31	2	Echolalia; Unresponsiveness to spoken voice
MRPS7	2	Echolalia; Unresponsiveness to spoken voice
MRPS9	2	Echolalia; Language disorder
MRTFB	2	Aggressive behaviour; Echolalia
MRTO4	2	Echolalia; Unresponsiveness to spoken voice
MS4A12	2	Echolalia; Language disorder
MS4A15	2	Anxiety; Echolalia
MS4A4A	2	Echolalia; Sleep disturbances
MS4A5	2	Echolalia; Unresponsiveness to spoken voice
MS4A6E	2	Aggressive behaviour; Echolalia
MSANTD1	2	Echolalia; Language disorder
MSRA	2	Echolalia; Language disorder
MSRB2	2	Aggressive behaviour; Echolalia
MSTO1	2	Echolalia; Unresponsiveness to spoken voice
MSX1	2	Aggressive behaviour; Echolalia
MT3	2	Aggressive behaviour; Echolalia
MTARC1	2	Aggressive behaviour; Echolalia
MTG1	2	Sleep disturbances; Unresponsiveness to spoken voice
MTHFD1	2	Anxiety; Echolalia
MTHFS	2	Echolalia; Motor delay
MTHFSD	2	Echolalia; Language disorder
MTIF2	2	Echolalia; Sleep disturbances
MTMR3	2	Language disorder; Motor delay
MTMR6	2	Echolalia; Language disorder
MTMR7	2	Echolalia; Mutism
MTO1	2	Echolalia; Language disorder
MTRNR2L9	2	Anxiety; Echolalia
MTSS1	2	Echolalia; Unresponsiveness to spoken voice
MTUS2	2	Echolalia; Unresponsiveness to spoken voice
MTX3	2	Echolalia; Motor delay
MUC19	2	Echolalia; Unresponsiveness to spoken voice
MUC3A	2	Echolalia; Language disorder
MUC5B	2	Echolalia; Language disorder
MUCL1	2	Echolalia; Motor delay
MVB12B	2	Anxiety; Echolalia
MVK	2	Aggressive behaviour; Echolalia
MX1	2	Echolalia; Language disorder
MXD3	2	Echolalia; Motor delay

MYBPC3	2	Echolalia; Unresponsiveness to spoken voice
MYC	2	Echolalia; Language disorder
MYCN	2	Echolalia; Motor delay
MYDGF	2	Aggressive behaviour; Echolalia
MYEOV	2	Echolalia; Motor delay
MYF6	2	Echolalia; Sleep disturbances
MYH16	2	Echolalia; Sleep disturbances
MYH3	2	Echolalia; Sleep disturbances
MYH4	2	Aggressive behaviour; Echolalia
MYH7B	2	Echolalia; Sleep disturbances
MYL5	2	Echolalia; Unresponsiveness to spoken voice
MYLIP	2	Aggressive behaviour; Echolalia
MYLK2	2	Echolalia; Unresponsiveness to spoken voice
MYLK4	2	Echolalia; Language disorder
MYMX	2	Aggressive behaviour; Echolalia
MYO18B	2	Echolalia; Unresponsiveness to spoken voice
MYO1B	2	Anxiety; Echolalia
MYO3B	2	Echolalia; Sleep disturbances
MYO5A	2	Aggressive behaviour; Echolalia
MYO5B	2	Aggressive behaviour; Echolalia
MYO5C	2	Anxiety; Echolalia
MYO7A	2	Anxiety; Echolalia
MYO7B	2	Aggressive behaviour; Echolalia
MYO9A	2	Aggressive behaviour; Echolalia
MYOC	2	Aggressive behaviour; Echolalia
MYOF	2	Echolalia; Motor delay
MYOG	2	Echolalia; Language disorder
MYOM1	2	Echolalia; Language disorder
MYOZ1	2	Aggressive behaviour; Echolalia
MYRFL	2	Echolalia; Motor delay
MYZAP	2	Anxiety; Echolalia
MZT2B	2	Echolalia; Motor delay
N4BP2	2	Echolalia; Language disorder
N4BP3	2	Anxiety; Echolalia
NAA38	2	Echolalia; Language disorder
NAA40	2	Echolalia; Unresponsiveness to spoken voice
NAA60	2	Aggressive behaviour; Echolalia
NAAA	2	Echolalia; Motor delay
NAALAD2	2	Aggressive behaviour; Echolalia
NACA	2	Echolalia; Language disorder
NACA2	2	Anxiety; Echolalia
NACAD	2	Echolalia; Motor delay
NADK	2	Echolalia; Unresponsiveness to spoken voice
NADK2	2	Sleep disturbances; Unresponsiveness to spoken voice
NAGK	2	Echolalia; Language disorder
NAGLU	2	Echolalia; Motor delay

NAGPA	2	Motor delay; Unresponsiveness to spoken voice
NARF	2	Anxiety; Unresponsiveness to spoken voice
NARS2	2	Echolalia; Motor delay
NAT1	2	Aggressive behaviour; Echolalia
NAT10	2	Digestive disorder; Echolalia
NAT2	2	Echolalia; Mutism
NAT9	2	Anxiety; Echolalia
NAV1	2	Anxiety; Echolalia
NAV3	2	Aggressive behaviour; Echolalia
NBPF11	2	Echolalia; Language disorder
NBPF12	2	Aggressive behaviour; Echolalia
NBPF15	2	Anxiety; Echolalia
NBPF3	2	Echolalia; Motor delay
NCAN	2	Anxiety; Echolalia
NCAPD2	2	Echolalia; Unresponsiveness to spoken voice
NCAPG2	2	Aggressive behaviour; Echolalia
NCAPH	2	Aggressive behaviour; Echolalia
NCBP2L	2	Echolalia; Unresponsiveness to spoken voice
NCEH1	2	Language disorder; Motor delay
NCF1	2	Echolalia; Unresponsiveness to spoken voice
NCL	2	Echolalia; Unresponsiveness to spoken voice
NCOA4	2	Echolalia; Sleep disturbances
NCOA5	2	Echolalia; Sleep disturbances
NCOA6	2	Anxiety; Echolalia
NDC1	2	Anxiety; Echolalia
NDC80	2	Echolalia; Sleep disturbances
NDNF	2	Echolalia; Language disorder
NDRG2	2	Anxiety; Echolalia
NDST1	2	Anxiety; Echolalia
NDST4	2	Aggressive behaviour; Echolalia
NDUFA10	2	Echolalia; Unresponsiveness to spoken voice
NDUFA11	2	Echolalia; Sleep disturbances
NDUFA9	2	Echolalia; Unresponsiveness to spoken voice
NDUFB10	2	Aggressive behaviour; Echolalia
NDUFS1	2	Echolalia; Motor delay
NDUFS3	2	Aggressive behaviour; Echolalia
NDUFS7	2	Echolalia; Unresponsiveness to spoken voice
NDUFV3	2	Aggressive behaviour; Echolalia
NEDD9	2	Anxiety; Echolalia
NEFM	2	Aggressive behaviour; Echolalia
NEK11	2	Echolalia; Unresponsiveness to spoken voice
NEK2	2	Echolalia; Motor delay
NEK6	2	Echolalia; Language disorder
NELFB	2	Echolalia; Language disorder
NEMP1	2	Echolalia; Language disorder
NEMP2	2	Echolalia; Unresponsiveness to spoken voice

NES	2	Echolalia; Sleep disturbances
NEU3	2	Echolalia; Unresponsiveness to spoken voice
NEURL1B	2	Echolalia; Sleep disturbances
NEURL3	2	Aggressive behaviour; Echolalia
NEURL4	2	Echolalia; Motor delay
NEUROD1	2	Echolalia; Motor delay
NEUROG3	2	Anxiety; Echolalia
NF1	2	Aggressive behaviour; Echolalia
NFATC3	2	Echolalia; Sleep disturbances
NFATC4	2	Aggressive behaviour; Echolalia
NFE2L1	2	Anxiety; Echolalia
NFE4	2	Aggressive behaviour; Echolalia
NFIL3	2	Aggressive behaviour; Echolalia
NFKBID	2	Aggressive behaviour; Echolalia
NFRKB	2	Anxiety; Echolalia
NGEF	2	Echolalia; Unresponsiveness to spoken voice
NHLRC3	2	Anxiety; Unresponsiveness to spoken voice
NHLRC4	2	Echolalia; Sleep disturbances
NHS	2	Echolalia; Unresponsiveness to spoken voice
NHSL1	2	Anxiety; Echolalia
NIBAN2	2	Anxiety; Unresponsiveness to spoken voice
NINL	2	Anxiety; Echolalia
NIPAL3	2	Echolalia; Motor delay
NIPAL4	2	Echolalia; Language disorder
NISCH	2	Echolalia; Language disorder
NKAIN3	2	Echolalia; Language disorder
NKAIN4	2	Anxiety; Echolalia
NKD1	2	Aggressive behaviour; Echolalia
NKPD1	2	Echolalia; Unresponsiveness to spoken voice
NKX2-1	2	Anxiety; Echolalia
NKX2-2	2	Anxiety; Echolalia
NKX2-4	2	Anxiety; Echolalia
NKX2-6	2	Aggressive behaviour; Echolalia
NKX3-2	2	Aggressive behaviour; Echolalia
NLE1	2	Anxiety; Echolalia
NLGN1	2	Echolalia; Unresponsiveness to spoken voice
NLN	2	Echolalia; Sleep disturbances
NLRP11	2	Echolalia; Unresponsiveness to spoken voice
NLRP13	2	Echolalia; Unresponsiveness to spoken voice
NLRP4	2	Echolalia; Unresponsiveness to spoken voice
NLRP6	2	Echolalia; Motor delay
NLRP9	2	Aggressive behaviour; Echolalia
NME5	2	Aggressive behaviour; Echolalia
NME6	2	Anxiety; Echolalia
NME8	2	Echolalia; Unresponsiveness to spoken voice
NMI	2	Echolalia; Unresponsiveness to spoken voice

NMNAT3	2	Language disorder; Motor delay
NMU	2	Echolalia; Motor delay
NMUR1	2	Echolalia; Motor delay
NMUR2	2	Aggressive behaviour; Echolalia
NOC2LP2	2	Aggressive behaviour; Echolalia
NOC4L	2	Echolalia; Language disorder
NOL10	2	Aggressive behaviour; Echolalia
NOM1	2	Echolalia; Unresponsiveness to spoken voice
NOP16	2	Aggressive behaviour; Echolalia
NOP53	2	Echolalia; Sleep disturbances
NOS3	2	Echolalia; Language disorder
NOTCH1	2	Echolalia; Motor delay
NOTCH2	2	Aggressive behaviour; Echolalia
NOTO	2	Aggressive behaviour; Echolalia
NOTUM	2	Aggressive behaviour; Echolalia
NOX4	2	Echolalia; Motor delay
NPAS2	2	Echolalia; Unresponsiveness to spoken voice
NPAT	2	Aggressive behaviour; Echolalia
NPDC1	2	Echolalia; Sleep disturbances
NPFFR1	2	Echolalia; Unresponsiveness to spoken voice
NPHP1	2	Anxiety; Echolalia
NPHP3	2	Echolalia; Motor delay
NPIPB3	2	Echolalia; Motor delay
NPIPB4	2	Echolalia; Motor delay
NPIPB8	2	Echolalia; Mutism
NPLOC4	2	Echolalia; Unresponsiveness to spoken voice
1,00 NPR	2	Echolalia; Motor delay
NPS	2	Echolalia; Language disorder
NPW	2	Anxiety; Echolalia
NR0B2	2	Echolalia; Unresponsiveness to spoken voice
NR1D1	2	Echolalia; Sleep disturbances
NR1H3	2	Echolalia; Motor delay
NR1I2	2	Aggressive behaviour; Echolalia
NR2C2	2	Echolalia; Motor delay
NR2E3	2	Echolalia; Motor delay
NR3C2	2	Aggressive behaviour; Echolalia
NR5A2	2	Echolalia; Unresponsiveness to spoken voice
NRBP2	2	Echolalia; Motor delay
NRCAM	2	Echolalia; Motor delay
NRG2	2	Echolalia; Unresponsiveness to spoken voice
NRIP1	2	Aggressive behaviour; Echolalia
NRN1L	2	Echolalia; Sleep disturbances
NSD2	2	Aggressive behaviour; Echolalia
NSFL1C	2	Echolalia; Motor delay
NSL1	2	Echolalia; Unresponsiveness to spoken voice
NSUN5	2	Anxiety; Echolalia

NT5DC2	2	Echolalia; Motor delay
NTMT1	2	Echolalia; Motor delay
NTN1	2	Echolalia; Unresponsiveness to spoken voice
NTN4	2	Echolalia; Unresponsiveness to spoken voice
NTN5	2	Echolalia; Motor delay
NTNG2	2	Anxiety; Echolalia
NTRK2	2	Anxiety; Echolalia
NTRK3	2	Echolalia; Unresponsiveness to spoken voice
NTSR1	2	Echolalia; Unresponsiveness to spoken voice
NUAK2	2	Echolalia; Unresponsiveness to spoken voice
NUDCD3	2	Anxiety; Echolalia
NUDT1	2	Echolalia; Sleep disturbances
NUDT16	2	Echolalia; Unresponsiveness to spoken voice
NUDT19	2	Echolalia; Language disorder
NUGGC	2	Aggressive behaviour; Echolalia
NUMA1	2	Echolalia; Language disorder
NUP107	2	Echolalia; Motor delay
NUP153	2	Echolalia; Language disorder
NUP160	2	Anxiety; Echolalia
NUP210	2	Echolalia; Unresponsiveness to spoken voice
NUP35	2	Aggressive behaviour; Echolalia
NUP50	2	Echolalia; Unresponsiveness to spoken voice
NUP54	2	Aggressive behaviour; Echolalia
NUPR1	2	Echolalia; Language disorder
NUTM2G	2	Echolalia; Motor delay
NVL	2	Aggressive behaviour; Echolalia
NWD1	2	Echolalia; Unresponsiveness to spoken voice
NXF5	2	Echolalia; Language disorder
NXN	2	Anxiety; Echolalia
NXNL1	2	Echolalia; Motor delay
NXPE1	2	Echolalia; Sleep disturbances
NXPE4	2	Echolalia; Sleep disturbances
OACYLP	2	Echolalia; Unresponsiveness to spoken voice
OAS2	2	Echolalia; Mutism
OAS3	2	Echolalia; Motor delay
OBP2B	2	Echolalia; Unresponsiveness to spoken voice
OC90	2	Anxiety; Echolalia
OCA2	2	Anxiety; Echolalia
OCSTAMP	2	Digestive disorder; Echolalia
ODF1	2	Anxiety; Echolalia
ODF3B	2	Echolalia; Unresponsiveness to spoken voice
ODF4	2	Anxiety; Echolalia
ODR4	2	Echolalia; Mutism
OGDHL	2	Echolalia; Unresponsiveness to spoken voice
OGFOD2	2	Echolalia; Unresponsiveness to spoken voice
OLFM1	2	Echolalia; Unresponsiveness to spoken voice

OLIG3	2	Aggressive behaviour; Echolalia
OMP	2	Echolalia; Language disorder
OOSP1	2	Echolalia; Motor delay
OOSP2	2	Echolalia; Sleep disturbances
OOSP4B	2	Anxiety; Echolalia
OPA1	2	Echolalia; Language disorder
OPLAH	2	Anxiety; Echolalia
OPRL1	2	Echolalia; Unresponsiveness to spoken voice
OPTC	2	Echolalia; Unresponsiveness to spoken voice
OPTN	2	Aggressive behaviour; Echolalia
OR10A4	2	Echolalia; Unresponsiveness to spoken voice
OR10A5	2	Aggressive behaviour; Echolalia
OR10AC1	2	Anxiety; Echolalia
OR10AG1	2	Echolalia; Unresponsiveness to spoken voice
OR10D3	2	Echolalia; Language disorder
OR10G2	2	Echolalia; Language disorder
OR10G4	2	Echolalia; Motor delay
OR10G6	2	Echolalia; Motor delay
OR10G8	2	Echolalia; Sleep disturbances
OR10G9	2	Echolalia; Motor delay
OR10H2	2	Aggressive behaviour; Echolalia
OR10H4	2	Anxiety; Echolalia
OR10H5	2	Echolalia; Sleep disturbances
OR10J3	2	Aggressive behaviour; Echolalia
OR10J4	2	Aggressive behaviour; Echolalia
OR10K1	2	Anxiety; Echolalia
OR10P1	2	Anxiety; Echolalia
OR10T2	2	Echolalia; Unresponsiveness to spoken voice
OR11H1	2	Echolalia; Unresponsiveness to spoken voice
OR11H12	2	Echolalia; Language disorder
OR11H6	2	Echolalia; Motor delay
OR11H7	2	Echolalia; Motor delay
OR13C5	2	Echolalia; Unresponsiveness to spoken voice
OR13J1	2	Aggressive behaviour; Echolalia
OR14A2	2	Language disorder; Motor delay
OR14K1	2	Echolalia; Unresponsiveness to spoken voice
OR1B1	2	Echolalia; Unresponsiveness to spoken voice
OR1E3	2	Echolalia; Unresponsiveness to spoken voice
OR1F1	2	Motor delay; Unresponsiveness to spoken voice
OR1G1	2	Motor delay; Unresponsiveness to spoken voice
OR1I1	2	Anxiety; Echolalia
OR1K1	2	Echolalia; Sleep disturbances
OR1L4	2	Echolalia; Language disorder
OR1M1	2	Echolalia; Sleep disturbances
OR1N1	2	Echolalia; Unresponsiveness to spoken voice
OR1Q1	2	Echolalia; Motor delay

OR2A2	2	Anxiety; Echolalia
OR2A4	2	Echolalia; Language disorder
OR2AE1	2	Echolalia; Motor delay
OR2AG2	2	Echolalia; Motor delay
OR2AP1	2	Echolalia; Mutism
OR2B11	2	Echolalia; Unresponsiveness to spoken voice
OR2F1	2	Aggressive behaviour; Echolalia
OR2G6	2	Anxiety; Echolalia
OR2L13	2	Aggressive behaviour; Echolalia
OR2L8	2	Echolalia; Unresponsiveness to spoken voice
OR2M4	2	Echolalia; Mutism
OR2M5	2	Aggressive behaviour; Echolalia
OR2T11	2	Aggressive behaviour; Echolalia
OR2T29	2	Echolalia; Mutism
OR2T33	2	Echolalia; Unresponsiveness to spoken voice
OR2T34	2	Echolalia; Motor delay
OR2T35	2	Echolalia; Motor delay
OR2T5	2	Echolalia; Mutism
OR2T8	2	Echolalia; Unresponsiveness to spoken voice
OR2V1	2	Echolalia; Sleep disturbances
OR2V2	2	Echolalia; Motor delay
OR2W3	2	Echolalia; Sleep disturbances
OR3A2	2	Echolalia; Motor delay
OR4A15	2	Echolalia; Language disorder
OR4A16	2	Echolalia; Language disorder
OR4A47	2	Echolalia; Unresponsiveness to spoken voice
OR4B1	2	Echolalia; Language disorder
OR4C11	2	Echolalia; Unresponsiveness to spoken voice
OR4C15	2	Echolalia; Unresponsiveness to spoken voice
OR4D2	2	Echolalia; Language disorder
OR4D5	2	Echolalia; Language disorder
OR4F17	2	Echolalia; Unresponsiveness to spoken voice
OR4K14	2	Echolalia; Language disorder
OR4N4C	2	Echolalia; Unresponsiveness to spoken voice
OR4P4	2	Echolalia; Unresponsiveness to spoken voice
OR4Q3	2	Echolalia; Unresponsiveness to spoken voice
OR4S2	2	Echolalia; Unresponsiveness to spoken voice
OR4X2	2	Echolalia; Motor delay
OR51A2	2	Echolalia; Motor delay
OR51B2	2	Anxiety; Echolalia
OR51B4	2	Anxiety; Echolalia
OR51B6	2	Anxiety; Echolalia
OR51E2	2	Echolalia; Mutism
OR51F1	2	Anxiety; Echolalia
OR51G2	2	Echolalia; Mutism
OR51H1	2	Echolalia; Mutism

OR51I1	2	Echolalia; Mutism
OR51I2	2	Anxiety; Echolalia
OR52A5	2	Aggressive behaviour; Echolalia
OR52B2	2	Anxiety; Echolalia
OR52E5	2	Aggressive behaviour; Echolalia
OR52E6	2	Echolalia; Motor delay
OR52H1	2	Echolalia; Motor delay
OR52K1	2	Aggressive behaviour; Echolalia
OR52N1	2	Echolalia; Language disorder
OR52R1	2	Anxiety; Echolalia
OR56B1	2	Echolalia; Mutism
OR56B4	2	Aggressive behaviour; Echolalia
OR5A1	2	Aggressive behaviour; Echolalia
OR5B2	2	Echolalia; Language disorder
OR5D13	2	Echolalia; Unresponsiveness to spoken voice
OR5D14	2	Anxiety; Echolalia
OR5D18	2	Anxiety; Echolalia
OR5I1	2	Echolalia; Unresponsiveness to spoken voice
OR5L1	2	Echolalia; Motor delay
OR5M1	2	Echolalia; Language disorder
OR5M3	2	Echolalia; Language disorder
OR5R1	2	Echolalia; Unresponsiveness to spoken voice
OR5W2	2	Echolalia; Unresponsiveness to spoken voice
OR6B2	2	Echolalia; Motor delay
OR6C2	2	Echolalia; Motor delay
OR6C3	2	Aggressive behaviour; Echolalia
OR6C76	2	Echolalia; Unresponsiveness to spoken voice
OR6F1	2	Echolalia; Language disorder
OR6J1	2	Aggressive behaviour; Echolalia
OR6K3	2	Echolalia; Motor delay
OR6K6	2	Aggressive behaviour; Echolalia
OR7A5	2	Echolalia; Motor delay
OR7D4	2	Echolalia; Motor delay
OR7E24	2	Echolalia; Motor delay
OR7G2	2	Aggressive behaviour; Echolalia
OR8B3	2	Echolalia; Motor delay
OR8B8	2	Aggressive behaviour; Echolalia
OR8G2P	2	Echolalia; Language disorder
OR8H2	2	Echolalia; Unresponsiveness to spoken voice
OR8I2	2	Echolalia; Unresponsiveness to spoken voice
OR8J3	2	Echolalia; Unresponsiveness to spoken voice
OR8S1	2	Echolalia; Unresponsiveness to spoken voice
OR8U1	2	Echolalia; Unresponsiveness to spoken voice
OR8U8	2	Echolalia; Unresponsiveness to spoken voice
OR9G1	2	Echolalia; Language disorder
OR9G9	2	Echolalia; Language disorder

OR9Q1	2	Echolalia; Mutism
OR9Q2	2	Echolalia; Unresponsiveness to spoken voice
ORM1	2	Echolalia; Unresponsiveness to spoken voice
OSBP2	2	Echolalia; Unresponsiveness to spoken voice
OSBPL10	2	Echolalia; Unresponsiveness to spoken voice
OSBPL1A	2	Echolalia; Language disorder
OSBPL2	2	Echolalia; Sleep disturbances
OSGEP	2	Echolalia; Language disorder
OSM	2	Echolalia; Unresponsiveness to spoken voice
OSMR	2	Echolalia; Language disorder
OSTF1	2	Anxiety; Echolalia
OTOGL	2	Anxiety; Echolalia
OTOL1	2	Echolalia; Sleep disturbances
OTOR	2	Echolalia; Unresponsiveness to spoken voice
OTOS	2	Aggressive behaviour; Anxiety
OTUD7B	2	Aggressive behaviour; Echolalia
OVCA2	2	Language disorder; Mutism
OVGP1	2	Aggressive behaviour; Echolalia
OXCT2	2	Aggressive behaviour; Echolalia
OXER1	2	Echolalia; Motor delay
OXR1	2	Echolalia; Language disorder
P2RX7	2	Echolalia; Unresponsiveness to spoken voice
P4HB	2	Echolalia; Motor delay
PABPC1	2	Echolalia; Motor delay
PABPC1L	2	Echolalia; Unresponsiveness to spoken voice
PABPC4L	2	Echolalia; Motor delay
PABPN1L	2	Aggressive behaviour; Echolalia
PACERR	2	Aggressive behaviour; Echolalia
PADI1	2	Anxiety; Echolalia
PADI2	2	Anxiety; Echolalia
PAF1	2	Aggressive behaviour; Echolalia
PAIP2B	2	Digestive disorder; Echolalia
PAK1IP1	2	Echolalia; Unresponsiveness to spoken voice
PAK4	2	Echolalia; Mutism
PALLD	2	Digestive disorder; Echolalia
PALMD	2	Echolalia; Motor delay
PAM	2	Echolalia; Language disorder
PAN3	2	Echolalia; Sleep disturbances
PANK1	2	Echolalia; Mutism
PANK2	2	Digestive disorder; Echolalia
PANX2	2	Echolalia; Unresponsiveness to spoken voice
PAOX	2	Echolalia; Motor delay
PAPLN	2	Echolalia; Unresponsiveness to spoken voice
PAPOLB	2	Echolalia; Motor delay
PAQR7	2	Aggressive behaviour; Echolalia
PARP3	2	Mutism; Unresponsiveness to spoken voice

PARP8	2	Anxiety; Echolalia
PARPBP	2	Anxiety; Echolalia
PARS2	2	Echolalia; Mutism
PARVB	2	Echolalia; Unresponsiveness to spoken voice
PARVG	2	Echolalia; Motor delay
PASD1	2	Echolalia; Language disorder
PAWR	2	Anxiety; Echolalia
PAX1	2	Anxiety; Echolalia
PBK	2	Echolalia; Unresponsiveness to spoken voice
PBXIP1	2	Anxiety; Echolalia
PCCB	2	Echolalia; Unresponsiveness to spoken voice
PCDH18	2	Echolalia; Unresponsiveness to spoken voice
PCDH19	2	Echolalia; Unresponsiveness to spoken voice
PCDHA11	2	Echolalia; Motor delay
PCDHA12	2	Echolalia; Motor delay
PCDHA6	2	Aggressive behaviour; Echolalia
PCDHAC2	2	Aggressive behaviour; Echolalia
PCDHB1	2	Echolalia; Mutism
PCDHB11	2	Aggressive behaviour; Echolalia
PCDHB12	2	Anxiety; Echolalia
PCDHB14	2	Echolalia; Sleep disturbances
PCDHB6	2	Echolalia; Unresponsiveness to spoken voice
PCDHB8	2	Anxiety; Echolalia
PCDHB9	2	Aggressive behaviour; Echolalia
PCDHGB4	2	Echolalia; Unresponsiveness to spoken voice
PCDHGB7	2	Aggressive behaviour; Echolalia
PCDHGC4	2	Echolalia; Motor delay
PCF11	2	Anxiety; Echolalia
PCIF1	2	Digestive disorder; Echolalia
PCK2	2	Echolalia; Language disorder
PCMT1	2	Aggressive behaviour; Echolalia
PCSK1	2	Echolalia; Motor delay
PCYOX1	2	Aggressive behaviour; Echolalia
PCYT2	2	Echolalia; Unresponsiveness to spoken voice
PDCD11	2	Anxiety; Echolalia
PDCD5	2	Aggressive behaviour; Anxiety
PDCL3P4	2	Echolalia; Language disorder
PDE1C	2	Echolalia; Mutism
PDE2A	2	Aggressive behaviour; Echolalia
PDE3A	2	Echolalia; Motor delay
PDE4A	2	Echolalia; Unresponsiveness to spoken voice
PDE4DIPP2	2	Anxiety; Echolalia
PDE5A	2	Aggressive behaviour; Echolalia
PDE6B	2	Echolalia; Unresponsiveness to spoken voice
PDE6C	2	Echolalia; Motor delay
PDE6G	2	Echolalia; Motor delay

PDE7B	2	Echolalia; Motor delay
PDGFRB	2	Aggressive behaviour; Echolalia
PDGFRL	2	Digestive disorder; Echolalia
PDHA1	2	Echolalia; Unresponsiveness to spoken voice
PDHX	2	Echolalia; Motor delay
PDIA6	2	Aggressive behaviour; Echolalia
PDK1	2	Aggressive behaviour; Echolalia
PDK2	2	Echolalia; Unresponsiveness to spoken voice
PDLIM4	2	Aggressive behaviour; Echolalia
PDP1	2	Aggressive behaviour; Echolalia
PDPK1	2	Echolalia; Unresponsiveness to spoken voice
PDXK	2	Echolalia; Unresponsiveness to spoken voice
PDZD2	2	Echolalia; Motor delay
PDZD3	2	Aggressive behaviour; Echolalia
PDZD7	2	Echolalia; Motor delay
PDZD8	2	Echolalia; Sleep disturbances
PDZRN4	2	Aggressive behaviour; Echolalia
PEAK1	2	Echolalia; Motor delay
PEAK3	2	Digestive disorder; Echolalia
PEAR1	2	Anxiety; Echolalia
PEF1	2	Echolalia; Language disorder
PEG10	2	Anxiety; Echolalia
PER3	2	Echolalia; Motor delay
PEX1	2	Aggressive behaviour; Echolalia
PEX10	2	Echolalia; Motor delay
PEX11G	2	Aggressive behaviour; Echolalia
PEX16	2	Echolalia; Motor delay
PEX26	2	Echolalia; Sleep disturbances
PFKFB3	2	Echolalia; Motor delay
PFKM	2	Echolalia; Motor delay
PFKP	2	Echolalia; Motor delay
PGA3	2	Echolalia; Unresponsiveness to spoken voice
PGAP3	2	Echolalia; Mutism
PGAP6	2	Echolalia; Unresponsiveness to spoken voice
PGC	2	Anxiety; Mutism
PGD	2	Echolalia; Sleep disturbances
PGLS	2	Anxiety; Unresponsiveness to spoken voice
PGLYRP3	2	Echolalia; Unresponsiveness to spoken voice
PGLYRP4	2	Echolalia; Unresponsiveness to spoken voice
PGM1	2	Aggressive behaviour; Echolalia
PGM2L1	2	Echolalia; Language disorder
PGM5	2	Echolalia; Sleep disturbances
PHC1	2	Echolalia; Unresponsiveness to spoken voice
PHETA1	2	Aggressive behaviour; Echolalia
PHF11	2	Echolalia; Unresponsiveness to spoken voice
PHF14	2	Echolalia; Language disorder

PHF20	2	Echolalia; Unresponsiveness to spoken voice
PHF21B	2	Echolalia; Unresponsiveness to spoken voice
PHF3	2	Aggressive behaviour; Echolalia
PHKG1	2	Echolalia; Language disorder
PHLDB1	2	Anxiety; Echolalia
PHLDB3	2	Echolalia; Motor delay
PHTF2	2	Echolalia; Unresponsiveness to spoken voice
PHYHIP	2	Aggressive behaviour; Echolalia
PI3	2	Aggressive behaviour; Echolalia
PIBF1	2	Echolalia; Language disorder
PICK1	2	Echolalia; Unresponsiveness to spoken voice
PICSAR	2	Aggressive behaviour; Echolalia
PID1	2	Aggressive behaviour; Echolalia
PIGF	2	Anxiety; Echolalia
PIGH	2	Echolalia; Unresponsiveness to spoken voice
PIGN	2	Echolalia; Motor delay
PIGO	2	Echolalia; Unresponsiveness to spoken voice
PIH1D1	2	Echolalia; Unresponsiveness to spoken voice
PIK3AP1	2	Echolalia; Mutism
PIK3CD	2	Echolalia; Motor delay
PIK3R4	2	Echolalia; Unresponsiveness to spoken voice
PIK3R6	2	Anxiety; Echolalia
PILRA	2	Echolalia; Motor delay
PINLYP	2	Anxiety; Echolalia
PIP	2	Echolalia; Unresponsiveness to spoken voice
PIP4K2C	2	Echolalia; Motor delay
PIP5K1A	2	Aggressive behaviour; Echolalia
PIP5K1C	2	Aggressive behaviour; Echolalia
PIP5KL1	2	Aggressive behaviour; Echolalia
PISD	2	Echolalia; Language disorder
PITPNA	2	Aggressive behaviour; Echolalia
PITPNM2	2	Echolalia; Unresponsiveness to spoken voice
PITPNM3	2	Aggressive behaviour; Echolalia
PITX2	2	Echolalia; Motor delay
PIWIL4	2	Aggressive behaviour; Echolalia
PJA1	2	Echolalia; Motor delay
PJA2	2	Aggressive behaviour; Echolalia
PKD1L1	2	Echolalia; Unresponsiveness to spoken voice
PKD1L3	2	Echolalia; Sleep disturbances
PKD2L2	2	Aggressive behaviour; Echolalia
PKDCC	2	Anxiety; Echolalia
PKM	2	Aggressive behaviour; Echolalia
PKN2	2	Echolalia; Motor delay
PKN3	2	Aggressive behaviour; Echolalia
PLA2G15	2	Echolalia; Sleep disturbances
PLA2G4A	2	Echolalia; Language disorder

PLA2G4C	2	Echolalia; Unresponsiveness to spoken voice
PLA2G4E	2	Echolalia; Sleep disturbances
PLA2G6	2	Echolalia; Sleep disturbances
PLA2G7	2	Echolalia; Motor delay
PLAAT5	2	Echolalia; Motor delay
PLAC8L1	2	Anxiety; Echolalia
PLCB1	2	Echolalia; Motor delay
PLCB3	2	Echolalia; Motor delay
PLCB4	2	Echolalia; Motor delay
PLCZ1	2	Anxiety; Echolalia
PLD6	2	Anxiety; Echolalia
PLEC	2	Aggressive behaviour; Echolalia
PLEKHA4	2	Echolalia; Unresponsiveness to spoken voice
PLEKHD1	2	Echolalia; Sleep disturbances
PLEKHG1	2	Anxiety; Echolalia
PLEKHG2	2	Echolalia; Language disorder
PLEKHG4	2	Echolalia; Unresponsiveness to spoken voice
PLEKHG7	2	Echolalia; Motor delay
PLEKHH2	2	Echolalia; Motor delay
PLEKHM2	2	Echolalia; Language disorder
PLEKHM3	2	Aggressive behaviour; Echolalia
PLEKHO1	2	Aggressive behaviour; Echolalia
PLIN3	2	Echolalia; Unresponsiveness to spoken voice
PLIN4	2	Echolalia; Sleep disturbances
PLIN5	2	Aggressive behaviour; Echolalia
PLK2	2	Echolalia; Motor delay
PLOD2	2	Echolalia; Motor delay
PLPP2	2	Echolalia; Motor delay
PLPP5	2	Echolalia; Unresponsiveness to spoken voice
PLPPR2	2	Anxiety; Mutism
PLPPR3	2	Echolalia; Unresponsiveness to spoken voice
PLPPR4	2	Echolalia; Unresponsiveness to spoken voice
PLRG1	2	Echolalia; Unresponsiveness to spoken voice
PLSCR5	2	Echolalia; Motor delay
PLTP	2	Echolalia; Sleep disturbances
PLXNA2	2	Echolalia; Unresponsiveness to spoken voice
PLXNA4	2	Echolalia; Unresponsiveness to spoken voice
PLXNB3	2	Echolalia; Mutism
PLXNC1	2	Echolalia; Unresponsiveness to spoken voice
PM20D1	2	Echolalia; Language disorder
PMM1	2	Echolalia; Sleep disturbances
PMM2	2	Aggressive behaviour; Echolalia
PMPCA	2	Echolalia; Unresponsiveness to spoken voice
PMS1	2	Aggressive behaviour; Echolalia
PMVK	2	Mutism; Sleep disturbances
PNKD	2	Echolalia; Unresponsiveness to spoken voice

PNKP	2	Aggressive behaviour; Echolalia
PNLDC1	2	Digestive disorder; Echolalia
PNLIP	2	Echolalia; Sleep disturbances
PNLIPRP1	2	Echolalia; Motor delay
PNLIPRP2	2	Anxiety; Echolalia
PNMA6A	2	Echolalia; Motor delay
PNMA6E	2	Echolalia; Language disorder
PNMA8A	2	Aggressive behaviour; Echolalia
PNMA8B	2	Echolalia; Motor delay
PNP	2	Anxiety; Echolalia
PNPLA8	2	Echolalia; Unresponsiveness to spoken voice
PODNL1	2	Aggressive behaviour; Echolalia
POF1B	2	Echolalia; Unresponsiveness to spoken voice
POFUT1	2	Echolalia; Unresponsiveness to spoken voice
POFUT2	2	Echolalia; Mutism
POGLUT1	2	Aggressive behaviour; Echolalia
POLD3	2	Echolalia; Sleep disturbances
POLDIP2	2	Echolalia; Motor delay
POLM	2	Aggressive behaviour; Echolalia
POLQ	2	Echolalia; Motor delay
POLR1B	2	Echolalia; Unresponsiveness to spoken voice
POLR2D	2	Aggressive behaviour; Echolalia
POLR2H	2	Anxiety; Echolalia
POLRMT	2	Anxiety; Echolalia
POM121L12	2	Echolalia; Motor delay
POMGNT2	2	Echolalia; Motor delay
POMT1	2	Echolalia; Motor delay
PON1	2	Echolalia; Unresponsiveness to spoken voice
PON2	2	Echolalia; Sleep disturbances
PON3	2	Echolalia; Unresponsiveness to spoken voice
POPDC3	2	Echolalia; Sleep disturbances
POR	2	Anxiety; Echolalia
PORCN	2	Echolalia; Language disorder
POTEB	2	Anxiety; Echolalia
POTEB2	2	Anxiety; Echolalia
POTEH	2	Echolalia; Language disorder
POTEI	2	Echolalia; Sleep disturbances
POTEJ	2	Echolalia; Unresponsiveness to spoken voice
POU2AF1	2	Echolalia; Unresponsiveness to spoken voice
POU4F2	2	Aggressive behaviour; Echolalia
PP2D1	2	Echolalia; Unresponsiveness to spoken voice
PPAN	2	Echolalia; Unresponsiveness to spoken voice
PPAN-P2RY11	2	Echolalia; Motor delay
PPARGC1A	2	Echolalia; Motor delay
PPARGC1B	2	Echolalia; Language disorder
PPEF2	2	Anxiety; Echolalia

PPFIA1	2	Aggressive behaviour; Echolalia
PPFIBP1	2	Echolalia; Unresponsiveness to spoken voice
PPFIBP2	2	Echolalia; Unresponsiveness to spoken voice
PPIB	2	Anxiety; Echolalia
PPIC	2	Echolalia; Unresponsiveness to spoken voice
PPID	2	Aggressive behaviour; Echolalia
PPIH	2	Echolalia; Unresponsiveness to spoken voice
PPIL1	2	Aggressive behaviour; Echolalia
PPM1N	2	Echolalia; Unresponsiveness to spoken voice
PPOX	2	Echolalia; Unresponsiveness to spoken voice
PPP1R12A	2	Aggressive behaviour; Echolalia
PPP1R13B	2	Anxiety; Echolalia
PPP1R16B	2	Echolalia; Unresponsiveness to spoken voice
PPP1R21	2	Aggressive behaviour; Echolalia
PPP1R32	2	Echolalia; Unresponsiveness to spoken voice
PPP1R3A	2	Echolalia; Sleep disturbances
PPP1R3B	2	Aggressive behaviour; Echolalia
PPP2R3A	2	Echolalia; Motor delay
PPP2R5A	2	Echolalia; Motor delay
PPP4R3C	2	Echolalia; Unresponsiveness to spoken voice
PPP5D1	2	Echolalia; Mutism
PPP6R2	2	Aggressive behaviour; Echolalia
PPRC1	2	Anxiety; Echolalia
PPT1	2	Echolalia; Language disorder
PRAC2	2	Echolalia; Motor delay
PRAMEF13	2	Echolalia; Unresponsiveness to spoken voice
PRAMEF18	2	Echolalia; Motor delay
PRAMEF22	2	Echolalia; Motor delay
PRAMENP	2	Anxiety; Echolalia
PRC1-AS1	2	Echolalia; Motor delay
PRDM11	2	Anxiety; Echolalia
PRDM16	2	Anxiety; Echolalia
PREP	2	Echolalia; Unresponsiveness to spoken voice
PREPL	2	Echolalia; Motor delay
PRF1	2	Echolalia; Motor delay
PRICKLE1	2	Anxiety; Echolalia
PRICKLE4	2	Anxiety; Echolalia
PRIMPOL	2	Aggressive behaviour; Echolalia
PRKAR1A	2	Echolalia; Unresponsiveness to spoken voice
PRKAR1B	2	Echolalia; Unresponsiveness to spoken voice
PRKAR2B	2	Anxiety; Echolalia
PRKCA	2	Echolalia; Motor delay
PRKCB	2	Echolalia; Unresponsiveness to spoken voice
PRKCD	2	Aggressive behaviour; Echolalia
PRKCI	2	Echolalia; Language disorder
PRKCQ	2	Aggressive behaviour; Echolalia

PRKCSH	2	Echolalia; Motor delay
PRKCZ	2	Echolalia; Motor delay
PRKCZ-AS1	2	Aggressive behaviour; Echolalia
PRKD1	2	Language disorder; Motor delay
PRKD3	2	Echolalia; Language disorder
PRKDC	2	Echolalia; Motor delay
PRKG2	2	Echolalia; Unresponsiveness to spoken voice
PRKX	2	Echolalia; Unresponsiveness to spoken voice
PRMT6	2	Echolalia; Sleep disturbances
PRMT9	2	Echolalia; Motor delay
PRODH	2	Anxiety; Echolalia
PROM2	2	Echolalia; Unresponsiveness to spoken voice
PROX2	2	Digestive disorder; Echolalia
PRPF31	2	Anxiety; Echolalia
PRPF40A	2	Language disorder; Unresponsiveness to spoken voice
PRPF40B	2	Echolalia; Unresponsiveness to spoken voice
PRPH2	2	Aggressive behaviour; Echolalia
PRR14	2	Echolalia; Motor delay
PRR16	2	Echolalia; Motor delay
PRR25	2	Aggressive behaviour; Echolalia
PRR27	2	Aggressive behaviour; Echolalia
PRR35	2	Echolalia; Unresponsiveness to spoken voice
PRR36	2	Echolalia; Unresponsiveness to spoken voice
PRRC2C	2	Digestive disorder; Echolalia
PRRT2	2	Aggressive behaviour; Echolalia
PRRT3	2	Echolalia; Language disorder
PRRT4	2	Echolalia; Unresponsiveness to spoken voice
PRSS3	2	Anxiety; Echolalia
PRSS35	2	Echolalia; Unresponsiveness to spoken voice
PRSS38	2	Echolalia; Language disorder
PRSS43P	2	Anxiety; Echolalia
PRSS45P	2	Echolalia; Unresponsiveness to spoken voice
PRSS46P	2	Aggressive behaviour; Echolalia
PRSS55	2	Echolalia; Motor delay
PRTG	2	Aggressive behaviour; Echolalia
PSD	2	Echolalia; Mutism
PSD2	2	Echolalia; Unresponsiveness to spoken voice
PSG1	2	Echolalia; Unresponsiveness to spoken voice
PSG2	2	Echolalia; Motor delay
PSG4	2	Echolalia; Motor delay
PSG5	2	Echolalia; Sleep disturbances
PSKH2	2	Aggressive behaviour; Echolalia
PSMA4	2	Anxiety; Echolalia
PSMA7	2	Echolalia; Motor delay
PSMB5	2	Aggressive behaviour; Echolalia
PSMC3IP	2	Language disorder; Motor delay

PSMC5	2	Echolalia; Unresponsiveness to spoken voice
PSMD8	2	Aggressive behaviour; Echolalia
PSME4	2	Echolalia; Motor delay
PSMF1	2	Echolalia; Motor delay
PSORS1C1	2	Anxiety; Echolalia
PTAR1	2	Echolalia; Unresponsiveness to spoken voice
PTBP1	2	Echolalia; Unresponsiveness to spoken voice
PTCD3	2	Anxiety; Echolalia
PTCH2	2	Echolalia; Motor delay
PTCHD4	2	Echolalia; Language disorder
PTCRA	2	Aggressive behaviour; Echolalia
PTEN	2	Echolalia; Motor delay
PTGDR	2	Aggressive behaviour; Echolalia
PTGER2	2	Aggressive behaviour; Echolalia
PTGER3	2	Echolalia; Sleep disturbances
PTGES	2	Anxiety; Echolalia
PTGES2	2	Echolalia; Unresponsiveness to spoken voice
PTGR2	2	Language disorder; Motor delay
PTH2R	2	Anxiety; Echolalia
PTK2	2	Echolalia; Motor delay
PTK6	2	Anxiety; Echolalia
PTPA	2	Echolalia; Unresponsiveness to spoken voice
PTPDC1	2	Echolalia; Motor delay
PTPN23	2	Echolalia; Unresponsiveness to spoken voice
PTPRB	2	Echolalia; Language disorder
PTPRN	2	Aggressive behaviour; Echolalia
PTPRO	2	Anxiety; Echolalia
PTPRT	2	Echolalia; Unresponsiveness to spoken voice
PTTG2	2	Echolalia; Unresponsiveness to spoken voice
PTX4	2	Echolalia; Unresponsiveness to spoken voice
PUDP	2	Echolalia; Language disorder
PURA	2	Echolalia; Unresponsiveness to spoken voice
PUS7L	2	Echolalia; Language disorder
PVR	2	Echolalia; Language disorder
PVRIG	2	Echolalia; Motor delay
PWWP3A	2	Anxiety; Echolalia
PXDN	2	Echolalia; Language disorder
PXDNL	2	Echolalia; Motor delay
PXN	2	Echolalia; Motor delay
PXYLP1	2	Anxiety; Echolalia
PYCR1	2	Echolalia; Motor delay
PYY	2	Aggressive behaviour; Anxiety
PZP	2	Anxiety; Echolalia
QARS1	2	Digestive disorder; Echolalia
QRFP	2	Echolalia; Unresponsiveness to spoken voice
QRICH1	2	Anxiety; Echolalia

QRSL1	2	Echolalia; Motor delay
QSER1	2	Echolalia; Mutism
R3HCC1	2	Aggressive behaviour; Echolalia
R3HDM4	2	Aggressive behaviour; Echolalia
RAB1B	2	Aggressive behaviour; Echolalia
RAB21	2	Echolalia; Motor delay
RAB23	2	Echolalia; Motor delay
RAB2B	2	Echolalia; Motor delay
RAB34	2	Echolalia; Motor delay
RAB35	2	Echolalia; Sleep disturbances
RAB38	2	Anxiety; Echolalia
RAB3GAP1	2	Echolalia; Sleep disturbances
RAB3GAP2	2	Echolalia; Motor delay
RAB40AL	2	Echolalia; Motor delay
RAB42	2	Echolalia; Motor delay
RAB7B	2	Echolalia; Sleep disturbances
RAB8A	2	Anxiety; Echolalia
RAB8B	2	Aggressive behaviour; Echolalia
RABEP2	2	Echolalia; Motor delay
RABEPK	2	Echolalia; Motor delay
RABGAP1	2	Anxiety; Echolalia
RABGAP1L	2	Echolalia; Unresponsiveness to spoken voice
RABL6	2	Echolalia; Sleep disturbances
RAC3	2	Echolalia; Unresponsiveness to spoken voice
RACK1	2	Echolalia; Language disorder
RAD23B	2	Echolalia; Language disorder
RAD51AP1	2	Aggressive behaviour; Echolalia
RAD51B	2	Echolalia; Motor delay
RAD54B	2	Echolalia; Sleep disturbances
RAMP3	2	Aggressive behaviour; Echolalia
RANBP2	2	Echolalia; Motor delay
RANGAP1	2	Echolalia; Unresponsiveness to spoken voice
RAPGEF3	2	Digestive disorder; Echolalia
RAPGEF5	2	Anxiety; Echolalia
RAPH1	2	Aggressive behaviour; Echolalia
RASD2	2	Aggressive behaviour; Echolalia
RASEF	2	Echolalia; Motor delay
RASGEF1B	2	Anxiety; Unresponsiveness to spoken voice
RASGRP2	2	Echolalia; Unresponsiveness to spoken voice
RASL12	2	Anxiety; Echolalia
RASSF10	2	Echolalia; Sleep disturbances
RASSF4	2	Echolalia; Unresponsiveness to spoken voice
RASSF7	2	Aggressive behaviour; Echolalia
RASSF8	2	Echolalia; Sleep disturbances
RBAK-RBAKDN	2	Aggressive behaviour; Echolalia
RBM10	2	Aggressive behaviour; Echolalia

RBM15B	2	Echolalia; Unresponsiveness to spoken voice
RBM20	2	Echolalia; Motor delay
RBM23	2	Echolalia; Language disorder
RBM26	2	Echolalia; Sleep disturbances
RBM28	2	Echolalia; Unresponsiveness to spoken voice
RBM3	2	Aggressive behaviour; Echolalia
RBM43	2	Echolalia; Unresponsiveness to spoken voice
RBM5	2	Aggressive behaviour; Echolalia
RBMS2	2	Echolalia; Sleep disturbances
RBMX	2	Echolalia; Unresponsiveness to spoken voice
RBMXL1	2	Aggressive behaviour; Echolalia
RBMXL2	2	Echolalia; Motor delay
RCAN1	2	Anxiety; Echolalia
RCC1L	2	Anxiety; Echolalia
RCC2	2	Echolalia; Motor delay
RCCD1	2	Echolalia; Unresponsiveness to spoken voice
RCE1	2	Digestive disorder; Unresponsiveness to spoken voice
RCN3	2	Aggressive behaviour; Echolalia
RCOR1	2	Echolalia; Unresponsiveness to spoken voice
RCOR3	2	Echolalia; Motor delay
RCSD1	2	Anxiety; Echolalia
RDH8	2	Echolalia; Sleep disturbances
REC114	2	Echolalia; Sleep disturbances
RECQL4	2	Anxiety; Echolalia
RECQL5	2	Echolalia; Unresponsiveness to spoken voice
REELD1	2	Aggressive behaviour; Echolalia
REEP1	2	Echolalia; Language disorder
REEP6	2	Echolalia; Unresponsiveness to spoken voice
REL	2	Aggressive behaviour; Unresponsiveness to spoken voice
RELB	2	Echolalia; Unresponsiveness to spoken voice
RELCH	2	Echolalia; Mutism
RELL1	2	Echolalia; Motor delay
RELT	2	Echolalia; Unresponsiveness to spoken voice
REN	2	Anxiety; Echolalia
RET	2	Echolalia; Sleep disturbances
RETREG1	2	Echolalia; Motor delay
REV1	2	Digestive disorder; Echolalia
REV3L	2	Echolalia; Mutism
RFC1	2	Aggressive behaviour; Echolalia
RFC3	2	Anxiety; Unresponsiveness to spoken voice
RFLNB	2	Echolalia; Language disorder
RFPL1	2	Echolalia; Motor delay
RFPL2	2	Echolalia; Motor delay
RFT1	2	Echolalia; Unresponsiveness to spoken voice
RFX6	2	Echolalia; Unresponsiveness to spoken voice
RFX8	2	Echolalia; Language disorder

RFXANK	2	Aggressive behaviour; Echolalia
RGL4	2	Echolalia; Unresponsiveness to spoken voice
RGS17	2	Echolalia; Language disorder
RGS2	2	Aggressive behaviour; Echolalia
RGS22	2	Echolalia; Sleep disturbances
RGS3	2	Aggressive behaviour; Echolalia
RGS6	2	Echolalia; Language disorder
RGSL1	2	Anxiety; Echolalia
RHBDD2	2	Echolalia; Language disorder
RHBDF1	2	Aggressive behaviour; Echolalia
RHBDF2	2	Aggressive behaviour; Echolalia
RHBG	2	Aggressive behaviour; Echolalia
RHOBTB3	2	Echolalia; Language disorder
RHOG	2	Echolalia; Language disorder
RHOT1	2	Echolalia; Unresponsiveness to spoken voice
RIC1	2	Echolalia; Mutism
RIC3	2	Echolalia; Language disorder
RILPL1	2	Echolalia; Unresponsiveness to spoken voice
RIMBP3	2	Echolalia; Motor delay
RIMBP3B	2	Anxiety; Echolalia
RIMKLA	2	Motor delay; Unresponsiveness to spoken voice
RIN2	2	Echolalia; Motor delay
RINT1	2	Echolalia; Motor delay
RIOK2	2	Echolalia; Language disorder
RIPK1	2	Echolalia; Sleep disturbances
RIPK4	2	Echolalia; Unresponsiveness to spoken voice
RIPOR1	2	Aggressive behaviour; Echolalia
RLN3	2	Echolalia; Sleep disturbances
RMDN1	2	Echolalia; Language disorder
RMDN2-AS1	2	Echolalia; Motor delay
RMI1	2	Aggressive behaviour; Echolalia
RMND1	2	Echolalia; Language disorder
RNASE11	2	Echolalia; Language disorder
RNASE13	2	Aggressive behaviour; Echolalia
RNF10	2	Echolalia; Language disorder
RNF103	2	Echolalia; Motor delay
RNF112	2	Echolalia; Motor delay
RNF125	2	Echolalia; Motor delay
RNF126	2	Echolalia; Sleep disturbances
RNF14	2	Anxiety; Mutism
RNF166	2	Echolalia; Unresponsiveness to spoken voice
RNF167	2	Echolalia; Sleep disturbances
RNF175	2	Echolalia; Unresponsiveness to spoken voice
RNF183	2	Echolalia; Unresponsiveness to spoken voice
RNF207	2	Digestive disorder; Echolalia
RNF213	2	Echolalia; Motor delay

RNF222	2	Echolalia; Unresponsiveness to spoken voice
RNF227	2	Echolalia; Motor delay
RNF31	2	Anxiety; Echolalia
RNF44	2	Echolalia; Unresponsiveness to spoken voice
RNFT1	2	Echolalia; Language disorder
RNLS	2	Echolalia; Motor delay
RNMT	2	Echolalia; Unresponsiveness to spoken voice
RNPC3	2	Echolalia; Motor delay
ROBO2	2	Echolalia; Motor delay
ROCK2	2	Echolalia; Language disorder
ROR1	2	Aggressive behaviour; Echolalia
RORA	2	Echolalia; Language disorder
RORC	2	Aggressive behaviour; Echolalia
ROS1	2	Echolalia; Sleep disturbances
RP1L1	2	Echolalia; Unresponsiveness to spoken voice
RPA1	2	Echolalia; Language disorder
RPA4	2	Echolalia; Language disorder
RPAP1	2	Anxiety; Echolalia
RPAP2	2	Echolalia; Sleep disturbances
RPGRIP1	2	Anxiety; Echolalia
RPL11	2	Echolalia; Unresponsiveness to spoken voice
RPL13A	2	Echolalia; Mutism
RPL24	2	Echolalia; Language disorder
RPL27A	2	Aggressive behaviour; Echolalia
RPL8	2	Anxiety; Echolalia
RPLP0	2	Echolalia; Motor delay
RPN2	2	Echolalia; Unresponsiveness to spoken voice
RPP38	2	Echolalia; Motor delay
RPS21	2	Anxiety; Echolalia
RPS24	2	Echolalia; Unresponsiveness to spoken voice
RPS4Y2	2	Echolalia; Sleep disturbances
RPSA	2	Echolalia; Sleep disturbances
RPUSD2	2	Echolalia; Language disorder
RRH	2	Echolalia; Unresponsiveness to spoken voice
RRN3	2	Echolalia; Sleep disturbances
RRP12	2	Anxiety; Echolalia
RSPH3	2	Echolalia; Language disorder
RSPH6A	2	Anxiety; Unresponsiveness to spoken voice
RTBDN	2	Echolalia; Language disorder
RTKN	2	Aggressive behaviour; Echolalia
RTL4	2	Echolalia; Unresponsiveness to spoken voice
RTN3	2	Echolalia; Unresponsiveness to spoken voice
RTP1	2	Motor delay; Unresponsiveness to spoken voice
RTP2	2	Digestive disorder; Echolalia
RTP4	2	Echolalia; Sleep disturbances
RTTN	2	Echolalia; Language disorder

RUBCNL	2	Echolalia; Mutism
RUNDC1	2	Anxiety; Unresponsiveness to spoken voice
RUNDC3B	2	Echolalia; Language disorder
RUNX1	2	Echolalia; Unresponsiveness to spoken voice
RUVBL2	2	Echolalia; Motor delay
RWDD3	2	Echolalia; Unresponsiveness to spoken voice
RXFP2	2	Anxiety; Echolalia
RYR2	2	Anxiety; Echolalia
S100A5	2	Echolalia; Unresponsiveness to spoken voice
S1PR2	2	Echolalia; Language disorder
SAA2-SAA4	2	Echolalia; Motor delay
SAAL1	2	Echolalia; Unresponsiveness to spoken voice
SAFB	2	Echolalia; Unresponsiveness to spoken voice
SAG	2	Aggressive behaviour; Echolalia
SALL2	2	Echolalia; Unresponsiveness to spoken voice
SALL4	2	Echolalia; Language disorder
SAMD11	2	Echolalia; Sleep disturbances
SAMD3	2	Aggressive behaviour; Echolalia
SAMD4A	2	Echolalia; Unresponsiveness to spoken voice
SAMD7	2	Aggressive behaviour; Echolalia
SAMD9	2	Echolalia; Motor delay
SAMSN1	2	Echolalia; Language disorder
SAP30BP	2	Echolalia; Language disorder
SAPCD2	2	Echolalia; Unresponsiveness to spoken voice
SARAF	2	Aggressive behaviour; Echolalia
SARS1	2	Echolalia; Mutism
SARS2	2	Anxiety; Echolalia
SART1	2	Motor delay; Unresponsiveness to spoken voice
SASS6	2	Echolalia; Motor delay
SAXO1	2	Echolalia; Unresponsiveness to spoken voice
SBF2	2	Echolalia; Unresponsiveness to spoken voice
SBK1	2	Aggressive behaviour; Echolalia
SBNO1	2	Echolalia; Unresponsiveness to spoken voice
SCAMP5	2	Aggressive behaviour; Echolalia
SCAPER	2	Aggressive behaviour; Echolalia
SCARB2	2	Anxiety; Echolalia
SCCPDH	2	Echolalia; Unresponsiveness to spoken voice
SCD	2	Anxiety; Mutism
SCG2	2	Anxiety; Echolalia
SCG3	2	Echolalia; Sleep disturbances
SCGB1C1	2	Aggressive behaviour; Echolalia
SCGB2B2	2	Aggressive behaviour; Echolalia
SCIMP	2	Aggressive behaviour; Echolalia
SCLY	2	Echolalia; Unresponsiveness to spoken voice
SCN2B	2	Echolalia; Motor delay
SCN3B	2	Echolalia; Unresponsiveness to spoken voice

SCNN1D	2	Echolalia; Unresponsiveness to spoken voice
SCP2	2	Echolalia; Unresponsiveness to spoken voice
SCUBE1	2	Anxiety; Echolalia
SCUBE2	2	Echolalia; Unresponsiveness to spoken voice
SCUBE3	2	Anxiety; Echolalia
SDC1	2	Echolalia; Unresponsiveness to spoken voice
SDCBP2	2	Echolalia; Unresponsiveness to spoken voice
SDE2	2	Echolalia; Unresponsiveness to spoken voice
SDHAF1	2	Aggressive behaviour; Echolalia
SDHAF4	2	Echolalia; Unresponsiveness to spoken voice
SDHD	2	Echolalia; Unresponsiveness to spoken voice
SDR42E1	2	Echolalia; Unresponsiveness to spoken voice
SEC11C	2	Echolalia; Unresponsiveness to spoken voice
SEC13	2	Aggressive behaviour; Echolalia
SEC16B	2	Anxiety; Echolalia
SEC22B	2	Echolalia; Motor delay
SEC23IP	2	Aggressive behaviour; Echolalia
SEC24A	2	Echolalia; Sleep disturbances
SEC24C	2	Echolalia; Motor delay
SEC31A	2	Echolalia; Unresponsiveness to spoken voice
SEL1L3	2	Echolalia; Mutism
SELENOI	2	Anxiety; Echolalia
SELENOV	2	Echolalia; Mutism
SELL	2	Echolalia; Unresponsiveness to spoken voice
SELPLG	2	Anxiety; Echolalia
SEM1	2	Echolalia; Unresponsiveness to spoken voice
SEMA3D	2	Aggressive behaviour; Echolalia
SEMA3E	2	Anxiety; Echolalia
SEMA3G	2	Echolalia; Unresponsiveness to spoken voice
SEMA4F	2	Aggressive behaviour; Echolalia
SEMA6A	2	Anxiety; Echolalia
SEMA6B	2	Aggressive behaviour; Echolalia
SEMG1	2	Echolalia; Sleep disturbances
SENP1	2	Echolalia; Unresponsiveness to spoken voice
SENP5	2	Anxiety; Echolalia
SENP7	2	Echolalia; Unresponsiveness to spoken voice
SEPTIN12	2	Aggressive behaviour; Echolalia
SEPTIN8	2	Anxiety; Echolalia
SERINC5	2	Echolalia; Unresponsiveness to spoken voice
SERPINA3	2	Echolalia; Unresponsiveness to spoken voice
SERPINA4	2	Aggressive behaviour; Echolalia
SERPINA9	2	Aggressive behaviour; Echolalia
SERPINB10	2	Echolalia; Unresponsiveness to spoken voice
SERPINB3	2	Echolalia; Motor delay
SERPINC1	2	Echolalia; Sleep disturbances
SERPINE2	2	Echolalia; Language disorder

**SERPINE3**	2	Echolalia; Unresponsiveness to spoken voice
**SERTAD4**	2	Echolalia; Motor delay
**SESTD1**	2	Echolalia; Language disorder
**SETBP1**	2	Echolalia; Language disorder
**SETD2**	2	Echolalia; Language disorder
**SETD5**	2	Echolalia; Unresponsiveness to spoken voice
**SETDB2**	2	Aggressive behaviour; Echolalia
**SETDB2-PHF11**	2	Echolalia; Unresponsiveness to spoken voice
**SETMAR**	2	Echolalia; Mutism
**SEZ6L2**	2	Echolalia; Language disorder
**SF3A1**	2	Echolalia; Motor delay
**SFI1**	2	Echolalia; Motor delay
**SFRP2**	2	Aggressive behaviour; Echolalia
**SFRP4**	2	Anxiety; Echolalia
**SFRP5**	2	Echolalia; Motor delay
**SFTA3**	2	Echolalia; Sleep disturbances
**SFTPA1**	2	Echolalia; Unresponsiveness to spoken voice
**SFTPA2**	2	Echolalia; Unresponsiveness to spoken voice
**SFTPB**	2	Aggressive behaviour; Echolalia
**SFTPD**	2	Echolalia; Mutism
**SFXN1**	2	Echolalia; Sleep disturbances
**SGCA**	2	Aggressive behaviour; Echolalia
**SGF29**	2	Echolalia; Unresponsiveness to spoken voice
**SGO2**	2	Echolalia; Language disorder
**SGPP1**	2	Echolalia; Unresponsiveness to spoken voice
**SGPP2**	2	Echolalia; Sleep disturbances
**SGSM1**	2	Echolalia; Motor delay
**SGSM3**	2	Anxiety; Echolalia
**SH2B3**	2	Anxiety; Echolalia
**SH2D5**	2	Echolalia; Unresponsiveness to spoken voice
**SH2D7**	2	Aggressive behaviour; Echolalia
**SH3BP5**	2	Echolalia; Language disorder
**SH3D19**	2	Echolalia; Motor delay
**SH3GL1**	2	Echolalia; Motor delay
**SH3GL3**	2	Echolalia; Motor delay
**SH3PXD2B**	2	Echolalia; Language disorder
**SH3TC2**	2	Echolalia; Unresponsiveness to spoken voice
**SH3YL1**	2	Aggressive behaviour; Echolalia
**SHANK1**	2	Echolalia; Language disorder
**SHANK3**	2	Echolalia; Sleep disturbances
**SHC1**	2	Echolalia; Motor delay
**SHC4**	2	Anxiety; Echolalia
**SHCBP1**	2	Echolalia; Unresponsiveness to spoken voice
**SHE**	2	Echolalia; Mutism
**SHISA2**	2	Echolalia; Unresponsiveness to spoken voice
**SHISA8**	2	Aggressive behaviour; Echolalia

SHLD1	2	Echolalia; Motor delay
SHLD3	2	Echolalia; Unresponsiveness to spoken voice
SHOC1	2	Echolalia; Motor delay
SHROOM1	2	Anxiety; Echolalia
SHROOM2	2	Echolalia; Motor delay
SHROOM3	2	Echolalia; Motor delay
SIAE	2	Echolalia; Unresponsiveness to spoken voice
SIDT2	2	Aggressive behaviour; Echolalia
SIGLEC10	2	Echolalia; Motor delay
SIGLEC10-AS1	2	Echolalia; Motor delay
SIGLEC11	2	Echolalia; Motor delay
SIGLEC14	2	Aggressive behaviour; Echolalia
SIGLEC5	2	Echolalia; Language disorder
SIGLEC8	2	Echolalia; Sleep disturbances
SIGLEC9	2	Echolalia; Motor delay
SIK1	2	Echolalia; Mutism
SIK1B	2	Echolalia; Mutism
SIK3	2	Anxiety; Echolalia
SIL1	2	Digestive disorder; Echolalia
SIM2	2	Anxiety; Echolalia
SIN3B	2	Echolalia; Language disorder
SIPA1	2	Echolalia; Unresponsiveness to spoken voice
SIPA1L2	2	Echolalia; Mutism
SIRPA	2	Aggressive behaviour; Echolalia
SIRPB2	2	Echolalia; Motor delay
SIRPG	2	Anxiety; Echolalia
SIRT3	2	Anxiety; Echolalia
SIRT4	2	Echolalia; Sleep disturbances
SIRT5	2	Anxiety; Echolalia
SIRT6	2	Echolalia; Sleep disturbances
SIX6	2	Echolalia; Unresponsiveness to spoken voice
SKAP1	2	Echolalia; Mutism
SKI	2	Echolalia; Sleep disturbances
SLAIN1	2	Aggressive behaviour; Echolalia
SLAMF9	2	Anxiety; Echolalia
SLC10A5	2	Echolalia; Mutism
SLC12A3	2	Echolalia; Unresponsiveness to spoken voice
SLC12A4	2	Echolalia; Sleep disturbances
SLC12A7	2	Echolalia; Sleep disturbances
SLC12A8	2	Echolalia; Motor delay
SLC12A9	2	Echolalia; Sleep disturbances
SLC13A1	2	Echolalia; Sleep disturbances
SLC13A5	2	Echolalia; Unresponsiveness to spoken voice
SLC15A1	2	Echolalia; Unresponsiveness to spoken voice
SLC16A5	2	Aggressive behaviour; Echolalia
SLC16A9	2	Aggressive behaviour; Echolalia

SLC17A3	2	Aggressive behaviour; Echolalia
SLC18A1	2	Echolalia; Motor delay
SLC1A4	2	Echolalia; Mutism
SLC1A5	2	Echolalia; Motor delay
SLC1A6	2	Echolalia; Sleep disturbances
SLC20A2	2	Echolalia; Motor delay
SLC22A10	2	Anxiety; Echolalia
SLC22A18	2	Echolalia; Sleep disturbances
SLC22A2	2	Echolalia; Sleep disturbances
SLC22A3	2	Echolalia; Motor delay
SLC22A5	2	Aggressive behaviour; Echolalia
SLC24A2	2	Anxiety; Echolalia
SLC24A3	2	Aggressive behaviour; Echolalia
SLC25A12	2	Echolalia; Motor delay
SLC25A26	2	Echolalia; Unresponsiveness to spoken voice
SLC25A32	2	Aggressive behaviour; Echolalia
SLC25A34	2	Anxiety; Echolalia
SLC25A37	2	Echolalia; Sleep disturbances
SLC25A39	2	Aggressive behaviour; Echolalia
SLC25A46	2	Echolalia; Mutism
SLC25A48	2	Anxiety; Echolalia
SLC25A52	2	Echolalia; Unresponsiveness to spoken voice
SLC26A11	2	Echolalia; Unresponsiveness to spoken voice
SLC26A2	2	Aggressive behaviour; Echolalia
SLC26A6	2	Anxiety; Echolalia
SLC28A2	2	Echolalia; Motor delay
SLC28A3	2	Echolalia; Mutism
SLC29A3	2	Aggressive behaviour; Echolalia
SLC2A10	2	Anxiety; Echolalia
SLC2A14	2	Echolalia; Mutism
SLC2A4	2	Aggressive behaviour; Echolalia
SLC2A4RG	2	Echolalia; Motor delay
SLC2A9	2	Echolalia; Unresponsiveness to spoken voice
SLC30A3	2	Echolalia; Unresponsiveness to spoken voice
SLC30A8	2	Anxiety; Echolalia
SLC34A1	2	Aggressive behaviour; Echolalia
SLC34A3	2	Aggressive behaviour; Echolalia
SLC35A2	2	Language disorder; Motor delay
SLC35A3	2	Echolalia; Motor delay
SLC35B2	2	Echolalia; Unresponsiveness to spoken voice
SLC35E2A	2	Echolalia; Language disorder
SLC35E2B	2	Echolalia; Motor delay
SLC35E4	2	Anxiety; Echolalia
SLC35F4	2	Aggressive behaviour; Echolalia
SLC35G6	2	Anxiety; Echolalia
SLC37A1	2	Echolalia; Unresponsiveness to spoken voice

SLC37A3	2	Echolalia; Motor delay
SLC37A4	2	Echolalia; Motor delay
SLC38A10	2	Aggressive behaviour; Echolalia
SLC38A5	2	Echolalia; Unresponsiveness to spoken voice
SLC38A6	2	Anxiety; Echolalia
SLC39A10	2	Aggressive behaviour; Echolalia
SLC39A11	2	Anxiety; Echolalia
SLC39A14	2	Echolalia; Unresponsiveness to spoken voice
SLC39A6	2	Echolalia; Sleep disturbances
SLC41A3	2	Echolalia; Motor delay
SLC43A1	2	Echolalia; Motor delay
SLC44A1	2	Echolalia; Sleep disturbances
SLC45A1	2	Echolalia; Motor delay
SLC45A3	2	Echolalia; Unresponsiveness to spoken voice
SLC4A10	2	Aggressive behaviour; Echolalia
SLC52A1	2	Echolalia; Motor delay
SLC52A3	2	Echolalia; Motor delay
SLC5A5	2	Echolalia; Mutism
SLC66A2	2	Echolalia; Motor delay
SLC6A1	2	Echolalia; Language disorder
SLC6A1-AS1	2	Echolalia; Motor delay
SLC6A13	2	Aggressive behaviour; Echolalia
SLC6A15	2	Echolalia; Sleep disturbances
SLC6A2	2	Echolalia; Unresponsiveness to spoken voice
SLC6A6	2	Echolalia; Unresponsiveness to spoken voice
SLC7A5	2	Echolalia; Unresponsiveness to spoken voice
SLC7A7	2	Echolalia; Language disorder
SLC8A1	2	Aggressive behaviour; Anxiety
SLC8A2	2	Echolalia; Unresponsiveness to spoken voice
SLC8A3	2	Echolalia; Motor delay
SLC9A3	2	Aggressive behaviour; Echolalia
SLC9A3R2	2	Anxiety; Echolalia
SLC9A8	2	Anxiety; Echolalia
SLC9A9	2	Echolalia; Motor delay
SLC9B2	2	Echolalia; Unresponsiveness to spoken voice
SLC9C1	2	Echolalia; Motor delay
SLC9C2	2	Echolalia; Sleep disturbances
SLCO1A2	2	Echolalia; Mutism
SLCO1B3	2	Echolalia; Sleep disturbances
SLCO2A1	2	Anxiety; Echolalia
SLCO2B1	2	Echolalia; Mutism
SLCO4A1	2	Echolalia; Language disorder
SLCO6A1	2	Echolalia; Unresponsiveness to spoken voice
SLMAP	2	Echolalia; Unresponsiveness to spoken voice
SLTM	2	Echolalia; Language disorder
SLX4	2	Echolalia; Unresponsiveness to spoken voice

SLX4IP	2	Echolalia; Motor delay
SMAD2	2	Aggressive behaviour; Echolalia
SMAP2	2	Echolalia; Unresponsiveness to spoken voice
SMARCA2	2	Echolalia; Motor delay
SMC1B	2	Echolalia; Motor delay
SMC2	2	Echolalia; Unresponsiveness to spoken voice
SMC3	2	Anxiety; Echolalia
SMC4	2	Echolalia; Unresponsiveness to spoken voice
SMG1	2	Digestive disorder; Echolalia
SMG7	2	Echolalia; Language disorder
SMIM24	2	Echolalia; Sleep disturbances
SMIM4	2	Echolalia; Motor delay
SMIM41	2	Anxiety; Echolalia
SMOC2	2	Echolalia; Mutism
SMPD1	2	Motor delay; Unresponsiveness to spoken voice
SMPD3	2	Digestive disorder; Echolalia
SMPD4BP	2	Echolalia; Language disorder
SMPD5	2	Anxiety; Echolalia
SNAPC1	2	Echolalia; Sleep disturbances
SNCA	2	Echolalia; Unresponsiveness to spoken voice
SNCG	2	Echolalia; Unresponsiveness to spoken voice
SND1	2	Digestive disorder; Echolalia
SNHG5	2	Aggressive behaviour; Echolalia
SNORC	2	Echolalia; Motor delay
SNRK	2	Echolalia; Unresponsiveness to spoken voice
SNRPA	2	Echolalia; Unresponsiveness to spoken voice
SNTG1	2	Echolalia; Unresponsiveness to spoken voice
SNTG2	2	Anxiety; Echolalia
SNUPN	2	Anxiety; Echolalia
SNW1	2	Anxiety; Echolalia
SNX13	2	Echolalia; Motor delay
SNX15	2	Anxiety; Echolalia
SNX18	2	Echolalia; Unresponsiveness to spoken voice
SNX21	2	Aggressive behaviour; Echolalia
SNX22	2	Echolalia; Motor delay
SNX29	2	Echolalia; Motor delay
SNX30	2	Echolalia; Unresponsiveness to spoken voice
SNX5	2	Echolalia; Unresponsiveness to spoken voice
SNX6	2	Echolalia; Motor delay
SOAT2	2	Echolalia; Language disorder
SOBP	2	Echolalia; Unresponsiveness to spoken voice
SOCS1	2	Echolalia; Motor delay
SOD3	2	Echolalia; Motor delay
SOGA1	2	Anxiety; Echolalia
SOGA3	2	Echolalia; Mutism
SOHLH2	2	Echolalia; Motor delay

SON	2	Echolalia; Motor delay
SORBS1	2	Echolalia; Unresponsiveness to spoken voice
SORBS3	2	Anxiety; Echolalia
SORCS2	2	Anxiety; Echolalia
SORL1	2	Anxiety; Echolalia
SORT1	2	Aggressive behaviour; Echolalia
1 SOS	2	Echolalia; Language disorder
SOX1-OT	2	Echolalia; Language disorder
SOX21	2	Echolalia; Motor delay
SOX8	2	Aggressive behaviour; Echolalia
SP110	2	Echolalia; Unresponsiveness to spoken voice
SP2-AS1	2	Aggressive behaviour; Echolalia
SP7	2	Anxiety; Echolalia
SP9	2	Aggressive behaviour; Echolalia
SPACA1	2	Language disorder; Motor delay
SPAG1	2	Echolalia; Motor delay
SPAG16	2	Echolalia; Motor delay
SPAG16-DT	2	Echolalia; Motor delay
SPAG5	2	Echolalia; Language disorder
SPAG6	2	Aggressive behaviour; Echolalia
SPAG7	2	Aggressive behaviour; Echolalia
SPAG8	2	Anxiety; Echolalia
SPAM1	2	Anxiety; Echolalia
SPANXC	2	Echolalia; Unresponsiveness to spoken voice
SPANXN3	2	Anxiety; Echolalia
SPART	2	Anxiety; Echolalia
SPATA13	2	Echolalia; Language disorder
SPATA16	2	Aggressive behaviour; Echolalia
SPATA17	2	Echolalia; Unresponsiveness to spoken voice
SPATA18	2	Language disorder; Motor delay
SPATA20	2	Echolalia; Motor delay
SPATA21	2	Echolalia; Unresponsiveness to spoken voice
SPATA22	2	Echolalia; Language disorder
SPATA31C2	2	Echolalia; Sleep disturbances
SPATA4	2	Aggressive behaviour; Echolalia
SPATA46	2	Echolalia; Language disorder
SPATA5	2	Echolalia; Motor delay
SPATA6L	2	Anxiety; Echolalia
SPATA9	2	Anxiety; Echolalia
SPDYE2	2	Echolalia; Mutism
SPDYE2B	2	Echolalia; Mutism
SPECC1L	2	Aggressive behaviour; Echolalia
SPEF2	2	Echolalia; Sleep disturbances
SPEG	2	Anxiety; Echolalia
SPEGNB	2	Aggressive behaviour; Motor delay
SPEM3	2	Anxiety; Echolalia

SPEN	2	Anxiety; Echolalia
SPESP1	2	Aggressive behaviour; Echolalia
SPHK1	2	Aggressive behaviour; Echolalia
SPHKAP	2	Echolalia; Unresponsiveness to spoken voice
SPIN2A	2	Aggressive behaviour; Echolalia
SPINK6	2	Echolalia; Motor delay
SPINT3	2	Aggressive behaviour; Echolalia
SPIRE1	2	Aggressive behaviour; Echolalia
SPNS1	2	Echolalia; Motor delay
SPNS2	2	Echolalia; Motor delay
SPOCD1	2	Echolalia; Unresponsiveness to spoken voice
SPOCK3	2	Aggressive behaviour; Echolalia
SPON2	2	Anxiety; Echolalia
SPOPL	2	Aggressive behaviour; Echolalia
SPP1	2	Echolalia; Sleep disturbances
SPP2	2	Echolalia; Unresponsiveness to spoken voice
SPPL2B	2	Aggressive behaviour; Echolalia
SPRED2	2	Echolalia; Unresponsiveness to spoken voice
SPRED3	2	Anxiety; Echolalia
SPRNP1	2	Echolalia; Motor delay
SPRR2F	2	Digestive disorder; Unresponsiveness to spoken voice
SPRY2	2	Aggressive behaviour; Echolalia
SPTBN2	2	Echolalia; Motor delay
SPTLC2	2	Echolalia; Motor delay
SRA1	2	Aggressive behaviour; Echolalia
SRBD1	2	Aggressive behaviour; Echolalia
SRD5A1	2	Aggressive behaviour; Echolalia
SRD5A2	2	Echolalia; Language disorder
SRFBP1	2	Aggressive behaviour; Echolalia
SRGAP2C	2	Anxiety; Echolalia
SRGAP3	2	Anxiety; Echolalia
SRL	2	Echolalia; Motor delay
SRP14	2	Echolalia; Unresponsiveness to spoken voice
SRP68	2	Echolalia; Unresponsiveness to spoken voice
SRP72	2	Echolalia; Sleep disturbances
SRPK3	2	Language disorder; Unresponsiveness to spoken voice
SRPX	2	Echolalia; Language disorder
SRRM1	2	Echolalia; Motor delay
SRRM2	2	Echolalia; Language disorder
SRRM5	2	Echolalia; Unresponsiveness to spoken voice
SSBP4	2	Anxiety; Echolalia
SSH2	2	Echolalia; Sleep disturbances
SSPN	2	Echolalia; Unresponsiveness to spoken voice
SSR3	2	Anxiety; Echolalia
SSR4P1	2	Aggressive behaviour; Echolalia
SSTR5	2	Echolalia; Motor delay

ST14	2	Echolalia; Language disorder
ST20-MTHFS	2	Aggressive behaviour; Echolalia
ST3GAL3	2	Echolalia; Language disorder
ST6GALNAC2	2	Echolalia; Unresponsiveness to spoken voice
ST6GALNAC4	2	Aggressive behaviour; Echolalia
ST6GALNAC5	2	Echolalia; Motor delay
ST6GALNAC6	2	Echolalia; Unresponsiveness to spoken voice
ST8SIA1	2	Echolalia; Motor delay
STAB2	2	Aggressive behaviour; Echolalia
STAG3	2	Anxiety; Echolalia
STAG3L3	2	Aggressive behaviour; Echolalia
STARD3	2	Aggressive behaviour; Echolalia
STARD5	2	Aggressive behaviour; Echolalia
STAT2	2	Aggressive behaviour; Echolalia
STAT5A	2	Anxiety; Echolalia
STAT5B	2	Echolalia; Sleep disturbances
STEAP1B	2	Echolalia; Unresponsiveness to spoken voice
STEAP2	2	Echolalia; Unresponsiveness to spoken voice
STIM2	2	Motor delay; Mutism
STING1	2	Aggressive behaviour; Echolalia
STK10	2	Echolalia; Unresponsiveness to spoken voice
STK24	2	Anxiety; Echolalia
STK26	2	Echolalia; Unresponsiveness to spoken voice
STK32B	2	Echolalia; Sleep disturbances
STK32C	2	Echolalia; Unresponsiveness to spoken voice
STK35	2	Echolalia; Language disorder
STK40	2	Echolalia; Sleep disturbances
STKLD1	2	Echolalia; Motor delay
STON1	2	Anxiety; Echolalia
STPG2	2	Echolalia; Unresponsiveness to spoken voice
STPG4	2	Echolalia; Unresponsiveness to spoken voice
STRA6	2	Aggressive behaviour; Echolalia
STRA8	2	Aggressive behaviour; Echolalia
STRADB	2	Echolalia; Motor delay
STRBP	2	Echolalia; Motor delay
STRC	2	Aggressive behaviour; Echolalia
STRN4	2	Echolalia; Mutism
STT3B	2	Echolalia; Language disorder
STX18	2	Echolalia; Motor delay
STX1A	2	Echolalia; Unresponsiveness to spoken voice
STX3	2	Echolalia; Unresponsiveness to spoken voice
STX5	2	Anxiety; Echolalia
STX8	2	Echolalia; Motor delay
STXBP4	2	Echolalia; Unresponsiveness to spoken voice
SUCLG2	2	Echolalia; Unresponsiveness to spoken voice
SULF2	2	Anxiety; Echolalia

SULT1A3	2	Echolalia; Language disorder
SULT1A4	2	Echolalia; Language disorder
SULT1C3	2	Aggressive behaviour; Echolalia
SUMO3	2	Anxiety; Echolalia
SUPT16H	2	Echolalia; Language disorder
SUPT20H	2	Echolalia; Motor delay
SUPT20HL1	2	Echolalia; Sleep disturbances
SUPT20HL2	2	Aggressive behaviour; Echolalia
SUPV3L1	2	Echolalia; Sleep disturbances
SURF6	2	Aggressive behaviour; Echolalia
SUSD3	2	Anxiety; Echolalia
SVOP	2	Echolalia; Sleep disturbances
SYBU	2	Echolalia; Mutism
SYCN	2	Echolalia; Unresponsiveness to spoken voice
SYCP2	2	Echolalia; Unresponsiveness to spoken voice
SYCP2L	2	Echolalia; Unresponsiveness to spoken voice
SYCP3	2	Echolalia; Unresponsiveness to spoken voice
SYDE1	2	Anxiety; Echolalia
SYN3	2	Digestive disorder; Echolalia
SYNE3	2	Aggressive behaviour; Echolalia
SYNGAP1	2	Echolalia; Unresponsiveness to spoken voice
SYNGR1	2	Aggressive behaviour; Echolalia
SYNJ1	2	Aggressive behaviour; Echolalia
SYNPO	2	Aggressive behaviour; Echolalia
SYNPO2L	2	Echolalia; Unresponsiveness to spoken voice
SYT1	2	Anxiety; Echolalia
SYT17	2	Echolalia; Unresponsiveness to spoken voice
SYT3	2	Echolalia; Motor delay
SYT6	2	Echolalia; Sleep disturbances
SYT7	2	Echolalia; Sleep disturbances
SYT8	2	Echolalia; Motor delay
SYTL1	2	Echolalia; Unresponsiveness to spoken voice
SYTL4	2	Anxiety; Echolalia
TAAR6	2	Echolalia; Sleep disturbances
TAB3	2	Echolalia; Unresponsiveness to spoken voice
TACC2	2	Echolalia; Motor delay
TACC3	2	Echolalia; Mutism
TACR2	2	Echolalia; Sleep disturbances
TAF11	2	Echolalia; Sleep disturbances
TAF1B	2	Echolalia; Unresponsiveness to spoken voice
TAF1C	2	Echolalia; Unresponsiveness to spoken voice
TAF1D	2	Echolalia; Mutism
TAF2	2	Anxiety; Echolalia
TAF5	2	Motor delay; Unresponsiveness to spoken voice
TAF5L	2	Echolalia; Motor delay
TAFA3	2	Echolalia; Motor delay

TAGLN3	2	Echolalia; Motor delay
TAL2	2	Echolalia; Motor delay
TAOK2	2	Echolalia; Language disorder
TAPBPL	2	Aggressive behaviour; Echolalia
TARBP1	2	Aggressive behaviour; Echolalia
TARP	2	Echolalia; Sleep disturbances
TARS1	2	Aggressive behaviour; Echolalia
TARS3	2	Echolalia; Motor delay
TAS1R3	2	Echolalia; Unresponsiveness to spoken voice
TAS2R16	2	Echolalia; Unresponsiveness to spoken voice
TAS2R38	2	Echolalia; Motor delay
TAS2R41	2	Echolalia; Language disorder
TASOR2	2	Echolalia; Sleep disturbances
TATDN2	2	Anxiety; Echolalia
TBC1D1	2	Echolalia; Unresponsiveness to spoken voice
TBC1D15	2	Echolalia; Motor delay
TBC1D16	2	Echolalia; Motor delay
TBC1D19	2	Echolalia; Sleep disturbances
TBC1D2	2	Aggressive behaviour; Echolalia
TBC1D21	2	Echolalia; Motor delay
TBC1D2B	2	Echolalia; Unresponsiveness to spoken voice
TBC1D30	2	Anxiety; Echolalia
TBC1D32	2	Echolalia; Language disorder
TBC1D5	2	Echolalia; Motor delay
TBC1D7	2	Echolalia; Unresponsiveness to spoken voice
TBCK	2	Echolalia; Language disorder
TBK1	2	Anxiety; Echolalia
TBRG4	2	Echolalia; Motor delay
TBX2	2	Echolalia; Unresponsiveness to spoken voice
TBX21	2	Echolalia; Language disorder
TBXA2R	2	Echolalia; Motor delay
TBXT	2	Echolalia; Language disorder
TCAF2	2	Echolalia; Language disorder
TCAP	2	Echolalia; Unresponsiveness to spoken voice
TCEA3	2	Echolalia; Motor delay
TCEAL3	2	Echolalia; Unresponsiveness to spoken voice
TCEAL4	2	Aggressive behaviour; Echolalia
TCERG1	2	Echolalia; Unresponsiveness to spoken voice
TCF21	2	Echolalia; Unresponsiveness to spoken voice
TCF25	2	Echolalia; Unresponsiveness to spoken voice
TCF3	2	Echolalia; Unresponsiveness to spoken voice
TCHH	2	Echolalia; Unresponsiveness to spoken voice
TDP1	2	Echolalia; Unresponsiveness to spoken voice
TDRD1	2	Anxiety; Mutism
TDRD5	2	Aggressive behaviour; Echolalia
TDRKH	2	Echolalia; Language disorder

TEAD3	2	Echolalia; Unresponsiveness to spoken voice
TEDC1	2	Echolalia; Unresponsiveness to spoken voice
TEKT3	2	Echolalia; Language disorder
TELO2	2	Aggressive behaviour; Echolalia
TEN1	2	Echolalia; Unresponsiveness to spoken voice
TENM2	2	Echolalia; Language disorder
TENM3	2	Anxiety; Echolalia
TEPSIN	2	Echolalia; Unresponsiveness to spoken voice
TESMIN	2	Echolalia; Motor delay
TET1	2	Echolalia; Unresponsiveness to spoken voice
TET2	2	Echolalia; Motor delay
TEX13B	2	Echolalia; Unresponsiveness to spoken voice
TEX13C	2	Echolalia; Unresponsiveness to spoken voice
TEX19	2	Echolalia; Language disorder
TEX2	2	Aggressive behaviour; Echolalia
TEX264	2	Anxiety; Echolalia
TEX29	2	Echolalia; Mutism
TEX38	2	Echolalia; Language disorder
TEX44	2	Echolalia; Unresponsiveness to spoken voice
TEX51	2	Echolalia; Language disorder
TEX55	2	Echolalia; Motor delay
TFAP2A	2	Echolalia; Sleep disturbances
TFAP2C	2	Echolalia; Unresponsiveness to spoken voice
TFAP4	2	Aggressive behaviour; Echolalia
TFG	2	Echolalia; Language disorder
TFPI	2	Aggressive behaviour; Echolalia
TFR2	2	Echolalia; Motor delay
TGFB1	2	Echolalia; Unresponsiveness to spoken voice
TGFBR1	2	Echolalia; Language disorder
TGM1	2	Echolalia; Motor delay
TGM4	2	Anxiety; Echolalia
TGM6	2	Echolalia; Unresponsiveness to spoken voice
TH	2	Echolalia; Unresponsiveness to spoken voice
THAP4	2	Motor delay; Unresponsiveness to spoken voice
THAP7	2	Aggressive behaviour; Echolalia
THBS4	2	Echolalia; Language disorder
THEM5	2	Echolalia; Unresponsiveness to spoken voice
THNSL2	2	Echolalia; Mutism
THOC1	2	Anxiety; Motor delay
THOC3	2	Aggressive behaviour; Echolalia
THRAP3	2	Echolalia; Motor delay
THSD7B	2	Echolalia; Unresponsiveness to spoken voice
TIAL1	2	Echolalia; Mutism
TIAM1	2	Echolalia; Language disorder
TIAM2	2	Echolalia; Language disorder
TICRR	2	Aggressive behaviour; Echolalia

TIE1	2	Echolalia; Unresponsiveness to spoken voice
TIGD6	2	Echolalia; Language disorder
TIMM21	2	Aggressive behaviour; Echolalia
TIMM23B	2	Echolalia; Motor delay
TINAG	2	Echolalia; Motor delay
TJP1	2	Aggressive behaviour; Echolalia
TKFC	2	Anxiety; Unresponsiveness to spoken voice
TKT	2	Aggressive behaviour; Echolalia
TKTL2	2	Echolalia; Unresponsiveness to spoken voice
TLCD5	2	Echolalia; Motor delay
TLE1	2	Anxiety; Echolalia
TLE2	2	Echolalia; Language disorder
TLL2	2	Echolalia; Unresponsiveness to spoken voice
TLN2	2	Echolalia; Language disorder
TLR2	2	Aggressive behaviour; Echolalia
TLR3	2	Anxiety; Echolalia
TLR4	2	Echolalia; Motor delay
TLR6	2	Echolalia; Unresponsiveness to spoken voice
TM4SF19	2	Echolalia; Motor delay
TM7SF2	2	Anxiety; Echolalia
TMBIM1	2	Aggressive behaviour; Echolalia
TMC1	2	Anxiety; Echolalia
TMC2	2	Anxiety; Echolalia
TMC7	2	Echolalia; Motor delay
TMCC1	2	Anxiety; Echolalia
TMCC3	2	Aggressive behaviour; Echolalia
TMCO4	2	Aggressive behaviour; Echolalia
TMED5	2	Aggressive behaviour; Echolalia
TMED8	2	Echolalia; Language disorder
TMEM102	2	Aggressive behaviour; Echolalia
TMEM104	2	Echolalia; Unresponsiveness to spoken voice
TMEM108	2	Echolalia; Unresponsiveness to spoken voice
TMEM120A	2	Aggressive behaviour; Echolalia
TMEM123	2	Echolalia; Motor delay
TMEM126B	2	Digestive disorder; Echolalia
TMEM127	2	Echolalia; Unresponsiveness to spoken voice
TMEM131	2	Echolalia; Unresponsiveness to spoken voice
TMEM132A	2	Echolalia; Motor delay
TMEM132B	2	Echolalia; Unresponsiveness to spoken voice
TMEM132D	2	Aggressive behaviour; Echolalia
TMEM132E	2	Aggressive behaviour; Echolalia
TMEM143	2	Anxiety; Echolalia
TMEM14B	2	Echolalia; Sleep disturbances
TMEM171	2	Echolalia; Motor delay
TMEM176A	2	Anxiety; Echolalia
TMEM176B	2	Echolalia; Language disorder

TMEM178B	2	Aggressive behaviour; Echolalia
TMEM18	2	Echolalia; Language disorder
TMEM184A	2	Aggressive behaviour; Echolalia
TMEM192	2	Echolalia; Motor delay
TMEM198	2	Aggressive behaviour; Echolalia
TMEM200C	2	Anxiety; Echolalia
TMEM207	2	Aggressive behaviour; Echolalia
TMEM209	2	Aggressive behaviour; Echolalia
TMEM213	2	Echolalia; Motor delay
TMEM215	2	Aggressive behaviour; Echolalia
TMEM230	2	Aggressive behaviour; Echolalia
TMEM235	2	Echolalia; Sleep disturbances
TMEM236	2	Echolalia; Unresponsiveness to spoken voice
TMEM237	2	Aggressive behaviour; Echolalia
TMEM245	2	Echolalia; Language disorder
TMEM247	2	Echolalia; Sleep disturbances
TMEM249	2	Echolalia; Unresponsiveness to spoken voice
TMEM255B	2	Echolalia; Language disorder
TMEM269	2	Echolalia; Unresponsiveness to spoken voice
TMEM30A	2	Echolalia; Sleep disturbances
TMEM52	2	Echolalia; Unresponsiveness to spoken voice
TMEM63A	2	Aggressive behaviour; Echolalia
TMEM63B	2	Anxiety; Echolalia
TMEM64	2	Echolalia; Sleep disturbances
TMEM71	2	Echolalia; Unresponsiveness to spoken voice
TMEM74B	2	Echolalia; Sleep disturbances
TMEM80	2	Echolalia; Unresponsiveness to spoken voice
TMEM81	2	Aggressive behaviour; Echolalia
TMEM86B	2	Echolalia; Unresponsiveness to spoken voice
TMEM87A	2	Echolalia; Sleep disturbances
TMEM8B	2	Echolalia; Sleep disturbances
TMEM9	2	Aggressive behaviour; Echolalia
TMEM94	2	Echolalia; Sleep disturbances
TMEM95	2	Anxiety; Echolalia
TMPRSS2	2	Echolalia; Sleep disturbances
TMPRSS9	2	Echolalia; Unresponsiveness to spoken voice
TMTC4	2	Echolalia; Unresponsiveness to spoken voice
TMUB2	2	Echolalia; Unresponsiveness to spoken voice
TNFAIP3	2	Echolalia; Motor delay
TNFAIP6	2	Echolalia; Sleep disturbances
TNFRSF10C	2	Echolalia; Unresponsiveness to spoken voice
TNFRSF10D	2	Echolalia; Language disorder
TNFRSF11B	2	Echolalia; Language disorder
TNFRSF19	2	Anxiety; Echolalia
TNFRSF1B	2	Echolalia; Unresponsiveness to spoken voice
TNFRSF25	2	Anxiety; Unresponsiveness to spoken voice

TNFRSF4	2	Echolalia; Sleep disturbances
TNIK	2	Echolalia; Language disorder
TNIP3	2	Echolalia; Language disorder
TNK1	2	Echolalia; Motor delay
TNK2	2	Aggressive behaviour; Echolalia
TNKS	2	Echolalia; Unresponsiveness to spoken voice
TNKS1BP1	2	Echolalia; Motor delay
TNNI2	2	Echolalia; Language disorder
TNNT1	2	Echolalia; Language disorder
TNNT3	2	Aggressive behaviour; Echolalia
TNPO1	2	Anxiety; Echolalia
TNPO2	2	Echolalia; Unresponsiveness to spoken voice
TNPO3	2	Echolalia; Unresponsiveness to spoken voice
TNRC6C	2	Echolalia; Sleep disturbances
TNS1	2	Aggressive behaviour; Echolalia
TNS3	2	Echolalia; Motor delay
TNS4	2	Echolalia; Motor delay
TOMM34	2	Anxiety; Echolalia
TOMM70	2	Echolalia; Unresponsiveness to spoken voice
TONSL	2	Aggressive behaviour; Echolalia
TOP1MT	2	Aggressive behaviour; Echolalia
TOX	2	Echolalia; Unresponsiveness to spoken voice
TP53	2	Echolalia; Motor delay
TP53AIP1	2	Aggressive behaviour; Echolalia
TP53BP1	2	Echolalia; Motor delay
TP53BP2	2	Echolalia; Language disorder
TP53I3	2	Aggressive behaviour; Echolalia
TP53TG5	2	Echolalia; Unresponsiveness to spoken voice
TPBGL	2	Anxiety; Echolalia
TPGS2	2	Echolalia; Sleep disturbances
TPH1	2	Motor delay; Unresponsiveness to spoken voice
TPH2	2	Aggressive behaviour; Echolalia
TPK1	2	Echolalia; Unresponsiveness to spoken voice
TPP1	2	Anxiety; Echolalia
TPPP2	2	Mutism; Unresponsiveness to spoken voice
TPR	2	Echolalia; Unresponsiveness to spoken voice
TPRG1	2	Digestive disorder; Echolalia
TPTE	2	Echolalia; Unresponsiveness to spoken voice
TPTE2P6	2	Echolalia; Language disorder
TRAF2	2	Aggressive behaviour; Echolalia
TRAF3	2	Aggressive behaviour; Echolalia
TRAF3IP1	2	Anxiety; Echolalia
TRANK1	2	Echolalia; Motor delay
TRAP1	2	Echolalia; Motor delay
TRAPPC10	2	Aggressive behaviour; Echolalia
TRAPPC11	2	Echolalia; Language disorder

TRAPPC2L	2	Echolalia; Unresponsiveness to spoken voice
TRAPPC6B	2	Echolalia; Motor delay
TRDMT1	2	Echolalia; Language disorder
TREH	2	Echolalia; Unresponsiveness to spoken voice
TREML4	2	Anxiety; Echolalia
TREX1	2	Anxiety; Echolalia
TRH	2	Aggressive behaviour; Echolalia
TRIB3	2	Echolalia; Unresponsiveness to spoken voice
TRIL	2	Anxiety; Echolalia
TRIM16	2	Echolalia; Sleep disturbances
TRIM17	2	Echolalia; Language disorder
TRIM24	2	Echolalia; Language disorder
TRIM29	2	Echolalia; Sleep disturbances
TRIM45	2	Echolalia; Unresponsiveness to spoken voice
TRIM47	2	Language disorder; Motor delay
TRIM49B	2	Echolalia; Unresponsiveness to spoken voice
TRIM49C	2	Aggressive behaviour; Echolalia
TRIM49D1	2	Echolalia; Motor delay
TRIM49D2	2	Echolalia; Motor delay
TRIM60	2	Echolalia; Motor delay
TRIM61	2	Aggressive behaviour; Echolalia
TRIM62	2	Echolalia; Sleep disturbances
TRIM64B	2	Aggressive behaviour; Echolalia
TRIM64C	2	Echolalia; Unresponsiveness to spoken voice
TRIM7	2	Echolalia; Motor delay
TRIML2	2	Echolalia; Motor delay
TRIOBP	2	Aggressive behaviour; Echolalia
TRIP11	2	Echolalia; Unresponsiveness to spoken voice
TRIP12	2	Echolalia; Unresponsiveness to spoken voice
TRIP4	2	Echolalia; Unresponsiveness to spoken voice
TRMO	2	Anxiety; Echolalia
TRMT2A	2	Echolalia; Unresponsiveness to spoken voice
TRMT61B	2	Echolalia; Unresponsiveness to spoken voice
TRMT9B	2	Anxiety; Echolalia
TRNP1	2	Echolalia; Sleep disturbances
TRNT1	2	Digestive disorder; Echolalia
TROAP	2	Echolalia; Sleep disturbances
TRPC1	2	Echolalia; Motor delay
TRPC7	2	Echolalia; Language disorder
TRPM1	2	Echolalia; Unresponsiveness to spoken voice
TRPM3	2	Anxiety; Echolalia
TRPM8	2	Anxiety; Echolalia
TRPS1	2	Echolalia; Motor delay
TRPT1	2	Anxiety; Echolalia
TRPV6	2	Aggressive behaviour; Echolalia
TSC1	2	Echolalia; Motor delay

TSC22D2	2	Aggressive behaviour; Echolalia
TSEN2	2	Anxiety; Language disorder
TSEN54	2	Echolalia; Unresponsiveness to spoken voice
TSFM	2	Echolalia; Unresponsiveness to spoken voice
TSGA10IP	2	Anxiety; Echolalia
TSHB	2	Anxiety; Echolalia
TSHR	2	Anxiety; Echolalia
TSNARE1	2	Echolalia; Motor delay
TSPAN10	2	Echolalia; Motor delay
TSPAN18	2	Echolalia; Unresponsiveness to spoken voice
TSPAN9	2	Echolalia; Sleep disturbances
TSPEAR	2	Echolalia; Language disorder
TSPOAP1	2	Echolalia; Motor delay
TSPY8	2	Echolalia; Motor delay
TSPYL6	2	Anxiety; Echolalia
TSTD2	2	Anxiety; Echolalia
TTC12	2	Aggressive behaviour; Echolalia
TTC13	2	Aggressive behaviour; Echolalia
TTC14	2	Echolalia; Motor delay
TTC17	2	Anxiety; Echolalia
TTC24	2	Anxiety; Echolalia
TTC25	2	Anxiety; Echolalia
TTC28	2	Aggressive behaviour; Echolalia
TTC29	2	Aggressive behaviour; Echolalia
TTC31	2	Aggressive behaviour; Echolalia
TTC38	2	Aggressive behaviour; Echolalia
TTC39A	2	Echolalia; Unresponsiveness to spoken voice
TTC5	2	Echolalia; Language disorder
TTC7A	2	Aggressive behaviour; Echolalia
TTC9	2	Aggressive behaviour; Echolalia
TTC9C	2	Aggressive behaviour; Echolalia
TTI2	2	Echolalia; Language disorder
TTLL13P	2	Aggressive behaviour; Echolalia
TTLL2	2	Aggressive behaviour; Echolalia
TTLL3	2	Echolalia; Motor delay
TTLL7	2	Anxiety; Language disorder
TTYH2	2	Echolalia; Unresponsiveness to spoken voice
TUB	2	Echolalia; Motor delay
TUBA1B	2	Aggressive behaviour; Echolalia
TUBA1C	2	Anxiety; Motor delay
TUBA3E	2	Echolalia; Motor delay
TUBA4B	2	Aggressive behaviour; Echolalia
TUBA8	2	Echolalia; Unresponsiveness to spoken voice
TUBAL3	2	Echolalia; Unresponsiveness to spoken voice
TUBB1	2	Echolalia; Unresponsiveness to spoken voice
TUBB2B	2	Echolalia; Motor delay

TUBB6	2	Echolalia; Motor delay
TULP2	2	Echolalia; Unresponsiveness to spoken voice
TULP3	2	Echolalia; Motor delay
TUT7	2	Echolalia; Sleep disturbances
TVP23B	2	Echolalia; Unresponsiveness to spoken voice
TVP23C	2	Echolalia; Motor delay
TXK	2	Echolalia; Language disorder
TXLNB	2	Anxiety; Echolalia
TXNDC11	2	Echolalia; Unresponsiveness to spoken voice
TXNDC17	2	Motor delay; Unresponsiveness to spoken voice
TXNRD1	2	Echolalia; Language disorder
TXNRD2	2	Anxiety; Echolalia
TYK2	2	Echolalia; Mutism
TYMS	2	Echolalia; Sleep disturbances
TYR	2	Echolalia; Unresponsiveness to spoken voice
TYRP1	2	Echolalia; Motor delay
TYSND1	2	Echolalia; Language disorder
TYW1	2	Echolalia; Unresponsiveness to spoken voice
TYW3	2	Aggressive behaviour; Echolalia
UBA1	2	Echolalia; Language disorder
UBAC1	2	Anxiety; Echolalia
UBASH3B	2	Echolalia; Language disorder
UBE2D4	2	Language disorder; Motor delay
UBE2F-SCLY	2	Anxiety; Echolalia
UBE2H	2	Echolalia; Motor delay
UBE2I	2	Echolalia; Motor delay
UBE2J1	2	Aggressive behaviour; Echolalia
UBE2Q2	2	Anxiety; Echolalia
UBE2U	2	Aggressive behaviour; Echolalia
UBE3D	2	Aggressive behaviour; Echolalia
UBE4A	2	Aggressive behaviour; Echolalia
UBOX5	2	Echolalia; Motor delay
UBR4	2	Anxiety; Echolalia
UBTFL1	2	Echolalia; Language disorder
UBXN10	2	Echolalia; Sleep disturbances
UCP3	2	Echolalia; Unresponsiveness to spoken voice
UEVLD	2	Aggressive behaviour; Echolalia
UGDH	2	Echolalia; Motor delay
UGT1A10	2	Echolalia; Motor delay
UGT1A4	2	Echolalia; Motor delay
UGT2B11	2	Echolalia; Motor delay
UGT2B17	2	Anxiety; Echolalia
UGT2B28	2	Aggressive behaviour; Echolalia
UGT2B4	2	Aggressive behaviour; Echolalia
UHRF1	2	Echolalia; Unresponsiveness to spoken voice
ULBP1	2	Echolalia; Motor delay

ULBP2	2	Anxiety; Echolalia
ULBP3	2	Echolalia; Unresponsiveness to spoken voice
ULK1	2	Echolalia; Language disorder
ULK3	2	Aggressive behaviour; Echolalia
ULK4	2	Echolalia; Unresponsiveness to spoken voice
UMPS	2	Echolalia; Language disorder
UNC13B	2	Echolalia; Unresponsiveness to spoken voice
UNC13D	2	Echolalia; Unresponsiveness to spoken voice
UNC45A	2	Echolalia; Sleep disturbances
UNC45B	2	Aggressive behaviour; Echolalia
UNC5C	2	Anxiety; Echolalia
UNC5D	2	Echolalia; Motor delay
UNCX	2	Echolalia; Unresponsiveness to spoken voice
UNG	2	Echolalia; Motor delay
UPF1	2	Echolalia; Language disorder
UPF2	2	Echolalia; Unresponsiveness to spoken voice
UPK2	2	Echolalia; Unresponsiveness to spoken voice
UPK3B	2	Aggressive behaviour; Echolalia
UPP2	2	Echolalia; Unresponsiveness to spoken voice
UQCC3	2	Echolalia; Motor delay
URAD	2	Echolalia; Mutism
URGCP	2	Anxiety; Echolalia
UROC1	2	Language disorder; Motor delay
USB1	2	Echolalia; Unresponsiveness to spoken voice
USF2	2	Echolalia; Unresponsiveness to spoken voice
USO1	2	Echolalia; Sleep disturbances
USP1	2	Echolalia; Motor delay
USP13	2	Echolalia; Language disorder
USP17L1	2	Echolalia; Motor delay
USP17L10	2	Echolalia; Sleep disturbances
USP17L20	2	Echolalia; Unresponsiveness to spoken voice
USP17L3	2	Echolalia; Motor delay
USP19	2	Echolalia; Unresponsiveness to spoken voice
USP2	2	Echolalia; Sleep disturbances
USP20	2	Aggressive behaviour; Echolalia
USP21	2	Anxiety; Echolalia
USP25	2	Echolalia; Motor delay
USP31	2	Echolalia; Language disorder
USP33	2	Echolalia; Unresponsiveness to spoken voice
USP38	2	Aggressive behaviour; Echolalia
USP4	2	Echolalia; Unresponsiveness to spoken voice
USP45	2	Aggressive behaviour; Echolalia
USP53	2	Aggressive behaviour; Echolalia
USP6NL	2	Echolalia; Unresponsiveness to spoken voice
UTP20	2	Echolalia; Unresponsiveness to spoken voice
UTP23	2	Anxiety; Echolalia

**UTP25**	2	Anxiety; Motor delay
**UTRN**	2	Echolalia; Unresponsiveness to spoken voice
**UTS2R**	2	Anxiety; Echolalia
**UTY**	2	Echolalia; Motor delay
**UVRAG**	2	Motor delay; Unresponsiveness to spoken voice
**VAC14**	2	Echolalia; Sleep disturbances
**VASH2**	2	Echolalia; Unresponsiveness to spoken voice
**VCAN**	2	Echolalia; Unresponsiveness to spoken voice
**VCL**	2	Digestive disorder; Echolalia
**VCPIP1**	2	Echolalia; Motor delay
**VCPKMT**	2	Echolalia; Unresponsiveness to spoken voice
**VCX2**	2	Echolalia; Motor delay
**VCX3A**	2	Echolalia; Motor delay
**VCX3B**	2	Echolalia; Unresponsiveness to spoken voice
**VENTX**	2	Digestive disorder; Echolalia
**VEPH1**	2	Anxiety; Echolalia
**VIL1**	2	Echolalia; Sleep disturbances
**VILL**	2	Anxiety; Echolalia
**VIPR1**	2	Echolalia; Sleep disturbances
**VIPR2**	2	Anxiety; Echolalia
**VKORC1L1**	2	Echolalia; Motor delay
**VLDLR**	2	Echolalia; Unresponsiveness to spoken voice
**VN1R2**	2	Anxiety; Echolalia
**1,00 VNN**	2	Aggressive behaviour; Echolalia
**2,00 VNN**	2	Echolalia; Language disorder
**VPS11**	2	Echolalia; Unresponsiveness to spoken voice
**VPS13B**	2	Echolalia; Unresponsiveness to spoken voice
**VPS13D**	2	Anxiety; Echolalia
**VPS18**	2	Echolalia; Sleep disturbances
**VPS26B**	2	Echolalia; Motor delay
**VPS33A**	2	Echolalia; Language disorder
**VPS33B**	2	Language disorder; Motor delay
**VPS37A**	2	Echolalia; Language disorder
**VPS54**	2	Aggressive behaviour; Echolalia
**VPS9D1**	2	Echolalia; Language disorder
**VSTM2L**	2	Echolalia; Motor delay
**VSTM4**	2	Echolalia; Motor delay
**VSX2**	2	Echolalia; Language disorder
**VTI1B**	2	Echolalia; Motor delay
**VTN**	2	Echolalia; Language disorder
**VWA1**	2	Motor delay; Unresponsiveness to spoken voice
**VWA2**	2	Aggressive behaviour; Echolalia
**VWA5B1**	2	Aggressive behaviour; Echolalia
**VWA8**	2	Echolalia; Unresponsiveness to spoken voice
**WASF2**	2	Echolalia; Unresponsiveness to spoken voice
**WASHC1**	2	Aggressive behaviour; Echolalia

WASHC2A	2	Echolalia; Motor delay
WASHC2C	2	Echolalia; Motor delay
WASHC4	2	Echolalia; Language disorder
WASHC5	2	Echolalia; Language disorder
WBP1L	2	Echolalia; Language disorder
WBP2NL	2	Echolalia; Motor delay
WDCP	2	Aggressive behaviour; Echolalia
WDHD1	2	Echolalia; Language disorder
WDR11	2	Echolalia; Unresponsiveness to spoken voice
WDR17	2	Echolalia; Unresponsiveness to spoken voice
WDR19	2	Echolalia; Language disorder
WDR24	2	Echolalia; Unresponsiveness to spoken voice
WDR31	2	Echolalia; Mutism
WDR33	2	Echolalia; Unresponsiveness to spoken voice
WDR35	2	Digestive disorder; Echolalia
WDR41	2	Echolalia; Motor delay
WDR43	2	Aggressive behaviour; Echolalia
WDR44	2	Aggressive behaviour; Echolalia
WDR5	2	Echolalia; Unresponsiveness to spoken voice
WDR55	2	Echolalia; Motor delay
WDR64	2	Anxiety; Echolalia
WDR7	2	Echolalia; Unresponsiveness to spoken voice
WDR70	2	Echolalia; Unresponsiveness to spoken voice
WDR73	2	Echolalia; Unresponsiveness to spoken voice
WDR74	2	Anxiety; Echolalia
WDR76	2	Echolalia; Unresponsiveness to spoken voice
WDR81	2	Aggressive behaviour; Echolalia
WDR90	2	Echolalia; Sleep disturbances
WDR91	2	Echolalia; Unresponsiveness to spoken voice
WDR92	2	Anxiety; Echolalia
WDR93	2	Echolalia; Unresponsiveness to spoken voice
WEE1	2	Aggressive behaviour; Echolalia
WFDC1	2	Anxiety; Echolalia
WFDC13	2	Digestive disorder; Echolalia
WFIKKN2	2	Anxiety; Echolalia
WHAMM	2	Aggressive behaviour; Echolalia
WHRN	2	Echolalia; Language disorder
WIPF1	2	Aggressive behaviour; Echolalia
WNK1	2	Aggressive behaviour; Echolalia
WNT10B	2	Echolalia; Unresponsiveness to spoken voice
WNT2B	2	Echolalia; Language disorder
WRAP53	2	Echolalia; Unresponsiveness to spoken voice
WRAP73	2	Anxiety; Echolalia
WRNIP1	2	Echolalia; Sleep disturbances
WSCD2	2	Aggressive behaviour; Echolalia
WWC3	2	Echolalia; Unresponsiveness to spoken voice

WWP1	2	Anxiety; Unresponsiveness to spoken voice
XG	2	Anxiety; Echolalia
XIRP2	2	Anxiety; Echolalia
XKR5	2	Echolalia; Language disorder
XPA	2	Echolalia; Language disorder
XPC	2	Echolalia; Unresponsiveness to spoken voice
XPO4	2	Echolalia; Sleep disturbances
XPO5	2	Aggressive behaviour; Echolalia
XPO6	2	Echolalia; Unresponsiveness to spoken voice
XRCC3	2	Echolalia; Unresponsiveness to spoken voice
XRN1	2	Aggressive behaviour; Echolalia
XRN2	2	Aggressive behaviour; Echolalia
XYLT2	2	Anxiety; Echolalia
YAF2	2	Aggressive behaviour; Echolalia
YEATS2	2	Echolalia; Motor delay
YEATS4	2	Echolalia; Motor delay
YIPF7	2	Echolalia; Language disorder
YME1L1	2	Aggressive behaviour; Echolalia
YWHAZ	2	Echolalia; Motor delay
YY1AP1	2	Anxiety; Echolalia
ZACN	2	Echolalia; Unresponsiveness to spoken voice
ZADH2	2	Echolalia; Unresponsiveness to spoken voice
ZASP	2	Echolalia; Unresponsiveness to spoken voice
ZBED5	2	Echolalia; Sleep disturbances
ZBED8	2	Anxiety; Echolalia
ZBED9	2	Echolalia; Motor delay
ZBP1	2	Anxiety; Echolalia
ZBTB14	2	Aggressive behaviour; Echolalia
ZBTB32	2	Echolalia; Language disorder
ZBTB39	2	Echolalia; Unresponsiveness to spoken voice
ZBTB4	2	Anxiety; Echolalia
ZBTB42	2	Echolalia; Motor delay
ZBTB49	2	Echolalia; Motor delay
ZBTB7B	2	Echolalia; Motor delay
ZC3H12D	2	Echolalia; Unresponsiveness to spoken voice
ZC3H14	2	Echolalia; Motor delay
ZCWPW2	2	Echolalia; Motor delay
ZDBF2	2	Echolalia; Motor delay
ZDHHC11	2	Anxiety; Echolalia
ZDHHC11B	2	Echolalia; Motor delay
ZDHHC13	2	Echolalia; Language disorder
ZDHHC14	2	Echolalia; Unresponsiveness to spoken voice
ZDHHC18	2	Echolalia; Language disorder
ZFAND2A	2	Echolalia; Motor delay
ZFAND4	2	Echolalia; Motor delay
ZFAT	2	Echolalia; Unresponsiveness to spoken voice

ZFC3H1	2	Echolalia; Motor delay
ZFP2	2	Echolalia; Motor delay
ZFP36L1	2	Echolalia; Language disorder
ZFP69	2	Echolalia; Motor delay
ZFPM2	2	Aggressive behaviour; Echolalia
ZFYVE1	2	Anxiety; Echolalia
ZFYVE19	2	Aggressive behaviour; Echolalia
ZGPAT	2	Anxiety; Unresponsiveness to spoken voice
ZHX2	2	Echolalia; Unresponsiveness to spoken voice
ZIC3	2	Aggressive behaviour; Echolalia
ZIM2	2	Echolalia; Unresponsiveness to spoken voice
ZIM3	2	Echolalia; Motor delay
ZMAT3	2	Motor delay; Unresponsiveness to spoken voice
ZMIZ1	2	Echolalia; Unresponsiveness to spoken voice
ZMIZ2	2	Echolalia; Language disorder
ZMYM1	2	Echolalia; Unresponsiveness to spoken voice
ZMYM2	2	Echolalia; Motor delay
ZMYM4	2	Echolalia; Mutism
ZMYM6	2	Aggressive behaviour; Echolalia
ZMYND10	2	Echolalia; Unresponsiveness to spoken voice
ZNF106	2	Echolalia; Sleep disturbances
ZNF107	2	Aggressive behaviour; Language disorder
ZNF117	2	Aggressive behaviour; Echolalia
ZNF14	2	Echolalia; Language disorder
ZNF140	2	Language disorder; Motor delay
ZNF142	2	Echolalia; Unresponsiveness to spoken voice
ZNF143	2	Echolalia; Unresponsiveness to spoken voice
ZNF175	2	Echolalia; Unresponsiveness to spoken voice
ZNF184	2	Echolalia; Unresponsiveness to spoken voice
ZNF185	2	Echolalia; Language disorder
ZNF189	2	Echolalia; Mutism
ZNF19	2	Echolalia; Unresponsiveness to spoken voice
ZNF213	2	Echolalia; Language disorder
ZNF22	2	Echolalia; Unresponsiveness to spoken voice
ZNF223	2	Echolalia; Unresponsiveness to spoken voice
ZNF235	2	Echolalia; Motor delay
ZNF239	2	Aggressive behaviour; Echolalia
ZNF25	2	Echolalia; Unresponsiveness to spoken voice
ZNF252P-AS1	2	Echolalia; Language disorder
ZNF26	2	Echolalia; Motor delay
ZNF280D	2	Aggressive behaviour; Echolalia
ZNF287	2	Aggressive behaviour; Echolalia
ZNF302	2	Digestive disorder; Echolalia
ZNF304	2	Echolalia; Language disorder
ZNF317	2	Echolalia; Mutism
ZNF320	2	Aggressive behaviour; Echolalia

ZNF322	2	Echolalia; Sleep disturbances
ZNF324	2	Echolalia; Mutism
ZNF324B	2	Language disorder; Motor delay
ZNF326	2	Echolalia; Language disorder
ZNF333	2	Anxiety; Echolalia
ZNF337	2	Echolalia; Unresponsiveness to spoken voice
ZNF33B	2	Echolalia; Unresponsiveness to spoken voice
ZNF34	2	Aggressive behaviour; Echolalia
ZNF343	2	Language disorder; Motor delay
ZNF35	2	Echolalia; Mutism
ZNF37A	2	Anxiety; Unresponsiveness to spoken voice
ZNF382	2	Echolalia; Sleep disturbances
ZNF385D	2	Echolalia; Motor delay
ZNF404	2	Anxiety; Echolalia
ZNF407	2	Echolalia; Motor delay
ZNF408	2	Echolalia; Motor delay
ZNF416	2	Echolalia; Unresponsiveness to spoken voice
ZNF419	2	Aggressive behaviour; Echolalia
ZNF426	2	Echolalia; Language disorder
ZNF429	2	Echolalia; Motor delay
ZNF43	2	Digestive disorder; Echolalia
ZNF432	2	Echolalia; Unresponsiveness to spoken voice
ZNF436	2	Echolalia; Mutism
ZNF438	2	Echolalia; Unresponsiveness to spoken voice
ZNF439	2	Anxiety; Echolalia
ZNF445	2	Echolalia; Unresponsiveness to spoken voice
ZNF461	2	Echolalia; Motor delay
ZNF467	2	Echolalia; Unresponsiveness to spoken voice
ZNF468	2	Echolalia; Motor delay
ZNF470	2	Echolalia; Language disorder
ZNF474	2	Aggressive behaviour; Echolalia
ZNF48	2	Echolalia; Unresponsiveness to spoken voice
ZNF486	2	Echolalia; Motor delay
ZNF487	2	Aggressive behaviour; Anxiety
ZNF490	2	Echolalia; Sleep disturbances
ZNF491	2	Aggressive behaviour; Echolalia
ZNF496	2	Anxiety; Echolalia
ZNF497	2	Aggressive behaviour; Echolalia
ZNF506	2	Echolalia; Language disorder
ZNF510	2	Echolalia; Language disorder
ZNF516	2	Echolalia; Motor delay
ZNF517	2	Aggressive behaviour; Echolalia
ZNF526	2	Anxiety; Mutism
ZNF527	2	Anxiety; Echolalia
ZNF536	2	Echolalia; Language disorder
ZNF541	2	Echolalia; Mutism

ZNF544	2	Echolalia; Unresponsiveness to spoken voice
ZNF550	2	Echolalia; Unresponsiveness to spoken voice
ZNF554	2	Anxiety; Echolalia
ZNF557	2	Echolalia; Unresponsiveness to spoken voice
ZNF559	2	Echolalia; Unresponsiveness to spoken voice
ZNF562	2	Echolalia; Unresponsiveness to spoken voice
ZNF567	2	Echolalia; Motor delay
ZNF570	2	Echolalia; Sleep disturbances
ZNF572	2	Echolalia; Unresponsiveness to spoken voice
ZNF580	2	Digestive disorder; Echolalia
ZNF583	2	Echolalia; Language disorder
ZNF584	2	Anxiety; Echolalia
ZNF585B	2	Echolalia; Unresponsiveness to spoken voice
ZNF589	2	Anxiety; Echolalia
ZNF594	2	Echolalia; Unresponsiveness to spoken voice
ZNF595	2	Echolalia; Unresponsiveness to spoken voice
ZNF597	2	Echolalia; Motor delay
ZNF599	2	Aggressive behaviour; Echolalia
ZNF600	2	Anxiety; Echolalia
ZNF611	2	Anxiety; Mutism
ZNF616	2	Aggressive behaviour; Echolalia
ZNF619	2	Language disorder; Motor delay
ZNF625	2	Echolalia; Motor delay
ZNF627	2	Aggressive behaviour; Echolalia
ZNF628	2	Echolalia; Motor delay
ZNF639	2	Echolalia; Sleep disturbances
ZNF646	2	Echolalia; Motor delay
ZNF652	2	Echolalia; Language disorder
ZNF654	2	Echolalia; Unresponsiveness to spoken voice
ZNF66	2	Echolalia; Language disorder
ZNF665	2	Echolalia; Unresponsiveness to spoken voice
ZNF672	2	Anxiety; Echolalia
ZNF682	2	Echolalia; Unresponsiveness to spoken voice
ZNF683	2	Anxiety; Echolalia
ZNF684	2	Echolalia; Language disorder
ZNF687	2	Echolalia; Unresponsiveness to spoken voice
ZNF689	2	Motor delay; Unresponsiveness to spoken voice
ZNF69	2	Echolalia; Mutism
ZNF692	2	Echolalia; Motor delay
ZNF695	2	Echolalia; Sleep disturbances
ZNF7	2	Aggressive behaviour; Echolalia
ZNF704	2	Echolalia; Motor delay
ZNF705A	2	Echolalia; Motor delay
ZNF716	2	Echolalia; Language disorder
ZNF730	2	Echolalia; Mutism
ZNF735	2	Echolalia; Language disorder

ZNF736	2	Echolalia; Language disorder
ZNF74	2	Echolalia; Motor delay
ZNF747	2	Echolalia; Unresponsiveness to spoken voice
ZNF749	2	Aggressive behaviour; Echolalia
ZNF750	2	Anxiety; Echolalia
ZNF75A	2	Echolalia; Motor delay
ZNF765	2	Echolalia; Unresponsiveness to spoken voice
ZNF774	2	Echolalia; Unresponsiveness to spoken voice
ZNF775	2	Echolalia; Unresponsiveness to spoken voice
ZNF778	2	Echolalia; Unresponsiveness to spoken voice
ZNF780B	2	Echolalia; Sleep disturbances
ZNF785	2	Echolalia; Sleep disturbances
ZNF788P	2	Echolalia; Motor delay
ZNF792	2	Echolalia; Unresponsiveness to spoken voice
ZNF799	2	Aggressive behaviour; Echolalia
ZNF8	2	Language disorder; Motor delay
ZNF8-ERVK3-1	2	Echolalia; Unresponsiveness to spoken voice
ZNF80	2	Echolalia; Language disorder
ZNF800	2	Echolalia; Motor delay
ZNF804A	2	Echolalia; Language disorder
ZNF806	2	Echolalia; Language disorder
ZNF814	2	Echolalia; Unresponsiveness to spoken voice
ZNF816-ZNF321P	2	Motor delay; Unresponsiveness to spoken voice
ZNF826P	2	Echolalia; Motor delay
ZNF830	2	Echolalia; Unresponsiveness to spoken voice
ZNF835	2	Echolalia; Language disorder
ZNF846	2	Aggressive behaviour; Echolalia
ZNF879	2	Echolalia; Unresponsiveness to spoken voice
ZNF891	2	Aggressive behaviour; Echolalia
ZNF90	2	Echolalia; Unresponsiveness to spoken voice
ZNF91	2	Echolalia; Motor delay
ZNF92	2	Echolalia; Unresponsiveness to spoken voice
ZNHIT6	2	Echolalia; Sleep disturbances
ZNRF1	2	Echolalia; Language disorder
ZP1	2	Echolalia; Motor delay
ZP4	2	Aggressive behaviour; Echolalia
ZRANB2	2	Echolalia; Sleep disturbances
ZRANB3	2	Echolalia; Sleep disturbances
ZRSR2	2	Aggressive behaviour; Echolalia
ZSCAN12	2	Echolalia; Language disorder
ZSCAN16	2	Echolalia; Unresponsiveness to spoken voice
ZSCAN22	2	Echolalia; Sleep disturbances
ZSCAN25	2	Echolalia; Unresponsiveness to spoken voice
ZSCAN26	2	Aggressive behaviour; Echolalia
ZSCAN29	2	Echolalia; Language disorder
ZSCAN31	2	Echolalia; Language disorder

ZSCAN32	2	Echolalia; Unresponsiveness to spoken voice
ZSCAN5B	2	Echolalia; Unresponsiveness to spoken voice
ZSWIM6	2	Echolalia; Unresponsiveness to spoken voice
ZSWIM8	2	Anxiety; Echolalia
ZSWIM9	2	Echolalia; Unresponsiveness to spoken voice
ZXDB	2	Echolalia; Unresponsiveness to spoken voice
A1BG	1	Echolalia
A1CF	1	Echolalia
A2M	1	Echolalia
A3GALT2	1	Anxiety
A4GALT	1	Echolalia
AAAS	1	Echolalia
AACS	1	Echolalia
AADACL4	1	Motor delay
AAGAB	1	Echolalia
AAK1	1	Echolalia
AAMDC	1	Echolalia
AARS1	1	Echolalia
AARS2	1	Echolalia
AASS	1	Echolalia
ABCA1	1	Echolalia
ABCA10	1	Echolalia
ABCA3	1	Echolalia
ABCA4	1	Echolalia
ABCA5	1	Echolalia
ABCA6	1	Sleep disturbances
ABCA7	1	Echolalia
ABCA8	1	Echolalia
ABCB1	1	Echolalia
ABCB11	1	Motor delay
ABCB4	1	Echolalia
ABCB5	1	Echolalia
ABCB8	1	Echolalia
ABCB9	1	Echolalia
ABCC10	1	Echolalia
ABCC3	1	Echolalia
ABCC4	1	Echolalia
ABCC5	1	Echolalia
ABCC8	1	Echolalia
ABCD4	1	Echolalia
ABCF2	1	Echolalia
ABCF2-H2BE1	1	Echolalia
ABCG2	1	Echolalia
ABCG4	1	Echolalia
ABCG8	1	Echolalia
ABHD1	1	Echolalia

ABHD12	1	Echolalia
ABHD18	1	Echolalia
ABHD4	1	Echolalia
ABHD8	1	Echolalia
ABI3BP	1	Echolalia
ABL1	1	Echolalia
ABLIM1	1	Echolalia
ABLIM3	1	Echolalia
ABRA	1	Motor delay
ABT1	1	Echolalia
ABTB2	1	Echolalia
ACAA1	1	Echolalia
ACACB	1	Echolalia
ACAD8	1	Echolalia
ACAD9	1	Echolalia
ACADL	1	Echolalia
ACADS	1	Echolalia
ACAP1	1	Echolalia
ACAP2	1	Echolalia
ACAP3	1	Echolalia
ACAT1	1	Echolalia
ACAT2	1	Echolalia
ACBD5	1	Echolalia
ACBD7-DCLRE1CP1	1	Echolalia
ACD	1	Echolalia
ACE	1	Echolalia
ACER1	1	Echolalia
ACER3	1	Echolalia
ACHE	1	Echolalia
ACIN1	1	Echolalia
ACKR1	1	Echolalia
ACKR3	1	Echolalia
ACKR4	1	Echolalia
ACO2	1	Echolalia
ACOD1	1	Echolalia
ACOT11	1	Echolalia
ACOT4	1	Echolalia
ACOX1	1	Echolalia
ACOX2	1	Echolalia
ACOX3	1	Echolalia
ACOXL	1	Echolalia
ACP2	1	Echolalia
ACP6	1	Echolalia
ACSBG2	1	Echolalia
ACSF2	1	Echolalia

ACSL1	1	Echolalia
ACSL5	1	Echolalia
ACSM2A	1	Echolalia
ACSM2B	1	Echolalia
ACSM3	1	Echolalia
ACSS1	1	Echolalia
ACSS3	1	Echolalia
ACTA2	1	Echolalia
ACTB	1	Echolalia
ACTL10	1	Motor delay
ACTL6A	1	Echolalia
ACTL7B	1	Echolalia
ACTN2	1	Echolalia
ACTN3	1	Echolalia
ACTN4	1	Echolalia
ACTR3B	1	Echolalia
ACTR3C	1	Echolalia
ACTR5	1	Echolalia
ACTRT3	1	Echolalia
ACVR1	1	Echolalia
ACVR1C	1	Unresponsiveness to spoken voice
ACVR2A	1	Echolalia
ACVRL1	1	Echolalia
ACYP2	1	Echolalia
ADA2	1	Echolalia
ADAD2	1	Echolalia
ADAM12	1	Echolalia
ADAM20	1	Motor delay
ADAM21	1	Echolalia
ADAM28	1	Echolalia
ADAM7	1	Echolalia
ADAM8	1	Echolalia
ADAM9	1	Echolalia
ADAMDEC1	1	Echolalia
ADAMTS10	1	Echolalia
ADAMTS13	1	Echolalia
ADAMTS14	1	Echolalia
ADAMTS15	1	Echolalia
ADAMTS16	1	Echolalia
ADAMTS18	1	Echolalia
ADAMTS2	1	Echolalia
ADAMTS7	1	Echolalia
ADAMTSL3	1	Echolalia
ADAMTSL4	1	Echolalia
ADAMTSL5	1	Echolalia
ADARB1	1	Echolalia

ADARB2	1	Echolalia
ADCK1	1	Echolalia
ADCY2	1	Echolalia
ADCY3	1	Echolalia
ADCY4	1	Echolalia
ADCY6	1	Echolalia
ADCY8	1	Echolalia
ADCY9	1	Echolalia
ADD1	1	Echolalia
ADD3	1	Echolalia
ADGRA1	1	Echolalia
ADGRA1-AS1	1	Echolalia
ADGRA3	1	Echolalia
ADGRB1	1	Echolalia
ADGRB2	1	Echolalia
ADGRD1	1	Echolalia
ADGRE2	1	Echolalia
ADGRE3	1	Echolalia
ADGRE5	1	Echolalia
ADGRF5	1	Echolalia
ADGRG1	1	Echolalia
ADGRG3	1	Echolalia
ADGRG4	1	Echolalia
ADGRG5	1	Echolalia
ADGRG6	1	Echolalia
ADGRG7	1	Echolalia
ADGRL1	1	Echolalia
ADGRL3	1	Echolalia
ADH1B	1	Echolalia
ADIPOR2	1	Echolalia
ADNP	1	Echolalia
ADO	1	Echolalia
ADORA1	1	Echolalia
ADORA2A-AS1	1	Echolalia
ADORA2B	1	Echolalia
ADORA3	1	Echolalia
ADPGK	1	Echolalia
ADRA1A	1	Echolalia
ADRA1D	1	Echolalia
ADRA2A	1	Echolalia
ADRA2B	1	Echolalia
ADRA2C	1	Echolalia
ADRB1	1	Echolalia
AEBP1	1	Echolalia
AFAP1L2	1	Echolalia
AFDN	1	Echolalia

AFF1	1	Echolalia
AFF3	1	Echolalia
AFF4	1	Echolalia
AFG1L	1	Echolalia
AFMID	1	Echolalia
AFTPH	1	Echolalia
AGAP1	1	Echolalia
AGAP13P	1	Echolalia
AGAP4	1	Echolalia
AGAP6	1	Echolalia
AGAP9	1	Echolalia
AGBL1	1	Echolalia
AGBL2	1	Echolalia
AGBL4	1	Echolalia
AGBL5	1	Echolalia
AGGF1	1	Echolalia
AGK	1	Echolalia
AGL	1	Echolalia
AGMAT	1	Echolalia
AGO2	1	Echolalia
AGPAT2	1	Echolalia
AGPAT3	1	Echolalia
AGPAT4	1	Echolalia
AGPAT5	1	Echolalia
AGPS	1	Echolalia
AGR2	1	Echolalia
AGRN	1	Echolalia
AGRP	1	Echolalia
AGT	1	Echolalia
AGTPBP1	1	Echolalia
AGTR1	1	Echolalia
AGXT	1	Echolalia
AHCTF1	1	Echolalia
AHCYL2	1	Echolalia
AHNAK2	1	Echolalia
AHSG	1	Echolalia
AIFM2	1	Echolalia
AIG1	1	Echolalia
AIM2	1	Motor delay
AIMP2	1	Echolalia
AIP	1	Echolalia
AIRE	1	Echolalia
AJM1	1	Echolalia
AK1	1	Echolalia
AK5	1	Echolalia
AK7	1	Echolalia

AK9	1	Echolalia
AKAP1	1	Motor delay
AKAP10	1	Echolalia
AKAP11	1	Echolalia
AKAP13	1	Echolalia
AKAP6	1	Echolalia
AKAP7	1	Echolalia
AKAP9	1	Echolalia
AKIP1	1	Language disorder
AKNA	1	Echolalia
AKR1C2	1	Echolalia
AKR1C3	1	Echolalia
AKR1C4	1	Echolalia
AKR1C8P	1	Echolalia
AKR7A2	1	Echolalia
AKT2	1	Echolalia
ALAS2	1	Echolalia
ALCAM	1	Echolalia
ALDH1B1	1	Echolalia
ALDH1L1	1	Echolalia
ALDH1L2	1	Unresponsiveness to spoken voice
ALDH3A2	1	Echolalia
ALDH4A1	1	Echolalia
ALDH5A1	1	Echolalia
ALDH6A1	1	Echolalia
ALDH7A1	1	Echolalia
ALDH9A1	1	Echolalia
ALG10	1	Echolalia
ALG10B	1	Echolalia
ALG12	1	Echolalia
ALG13	1	Echolalia
ALG1L	1	Echolalia
ALG1L9P	1	Echolalia
ALK	1	Echolalia
ALKAL2	1	Echolalia
ALKBH2	1	Echolalia
ALKBH3	1	Echolalia
ALKBH3-AS1	1	Echolalia
ALKBH5	1	Echolalia
ALKBH8	1	Echolalia
ALLC	1	Echolalia
ALOX12	1	Echolalia
ALOX15	1	Echolalia
ALOXE3	1	Echolalia
ALPG	1	Echolalia
ALPI	1	Echolalia

ALPK2	1	Echolalia
ALPK3	1	Echolalia
ALPP	1	Echolalia
ALS2	1	Echolalia
AMBP	1	Motor delay
AMBRA1	1	Echolalia
AMER1	1	Echolalia
AMER3	1	Echolalia
AMHR2	1	Echolalia
AMOTL1	1	Motor delay
AMOTL2	1	Echolalia
AMPD1	1	Echolalia
AMPD3	1	Echolalia
AMPH	1	Echolalia
AMTN	1	Echolalia
AMY2A	1	Echolalia
AMZ2	1	Echolalia
ANAPC1	1	Echolalia
ANAPC10	1	Echolalia
ANAPC11	1	Echolalia
ANAPC16	1	Unresponsiveness to spoken voice
ANAPC1P2	1	Echolalia
ANAPC1P4	1	Echolalia
ANAPC2	1	Echolalia
ANAPC4	1	Echolalia
ANG	1	Echolalia
ANGPT2	1	Echolalia
ANGPT4	1	Echolalia
ANGPTL1	1	Echolalia
ANGPTL4	1	Echolalia
ANGPTL6	1	Echolalia
ANGPTL8	1	Anxiety
ANHX	1	Echolalia
ANK2	1	Echolalia
ANK3	1	Echolalia
ANKFY1	1	Echolalia
ANKIB1	1	Echolalia
ANKK1	1	Echolalia
ANKLE1	1	Echolalia
ANKLE2	1	Echolalia
ANKMY2	1	Echolalia
ANKRD1	1	Language disorder
ANKRD12	1	Echolalia
ANKRD13C	1	Echolalia
ANKRD13D	1	Echolalia
ANKRD16	1	Echolalia

ANKRD18A	1	Echolalia
ANKRD2	1	Echolalia
ANKRD20A4-ANKRD20A20P	1	Echolalia
ANKRD22	1	Echolalia
ANKRD24	1	Echolalia
ANKRD26	1	Echolalia
ANKRD27	1	Echolalia
ANKRD29	1	Echolalia
ANKRD30A	1	Echolalia
ANKRD30B	1	Echolalia
ANKRD36	1	Echolalia
ANKRD36B	1	Echolalia
ANKRD36C	1	Echolalia
ANKRD44	1	Echolalia
ANKRD45	1	Echolalia
ANKRD53	1	Echolalia
ANKRD55	1	Echolalia
ANKRD6	1	Echolalia
ANKRD61	1	Echolalia
ANKRD62	1	Echolalia
ANKRD65	1	Echolalia
ANKS1B	1	Echolalia
ANKS4B	1	Echolalia
ANKZF1	1	Echolalia
ANO10	1	Echolalia
ANO5	1	Echolalia
ANO6	1	Echolalia
ANO7	1	Echolalia
ANO9	1	Echolalia
ANP32E	1	Echolalia
ANTXR1	1	Echolalia
ANXA10	1	Echolalia
ANXA11	1	Echolalia
ANXA13	1	Echolalia
ANXA2	1	Echolalia
ANXA6	1	Echolalia
ANXA8	1	Echolalia
AOAH	1	Echolalia
AOC1	1	Echolalia
AOPEP	1	Echolalia
AP1B1	1	Echolalia
AP1G2	1	Echolalia
AP1M1	1	Echolalia
AP1M2	1	Echolalia
AP2B1	1	Echolalia

AP2M1	1	Echolalia
AP3B1	1	Echolalia
AP3B2	1	Echolalia
AP4B1	1	Echolalia
AP4M1	1	Echolalia
AP5M1	1	Echolalia
AP5Z1	1	Echolalia
APBA1	1	Echolalia
APBB1	1	Echolalia
APC	1	Echolalia
APC2	1	Echolalia
APCDD1	1	Echolalia
APCDD1L	1	Echolalia
APH1B	1	Echolalia
APIP	1	Echolalia
APLF	1	Echolalia
APLP1	1	Echolalia
APOA2	1	Echolalia
APOA4	1	Echolalia
APOB	1	Echolalia
APOBEC1	1	Echolalia
APOBEC2	1	Echolalia
APOBEC3B	1	Echolalia
APOBEC3D	1	Unresponsiveness to spoken voice
APOBEC3F	1	Echolalia
APOBEC3G	1	Echolalia
APOBEC4	1	Echolalia
APOBR	1	Echolalia
APOC1	1	Echolalia
APOC3	1	Echolalia
APOH	1	Echolalia
APOL1	1	Echolalia
APOL2	1	Echolalia
APOL3	1	Echolalia
APOL5	1	Echolalia
APOLD1	1	Echolalia
APPBP2	1	Echolalia
APPL2	1	Echolalia
APRT	1	Echolalia
AQP2	1	Anxiety
AQP3	1	Echolalia
AQP4	1	Echolalia
AQP7	1	Echolalia
AQP7P3	1	Echolalia
AQP8	1	Echolalia
AQP9	1	Echolalia

AR	1	Echolalia
ARAP2	1	Echolalia
ARCN1	1	Echolalia
AREG	1	Echolalia
AREL1	1	Anxiety
ARF3	1	Echolalia
ARFGAP1	1	Echolalia
ARFGEF1	1	Echolalia
ARFGEF3	1	Echolalia
ARHGAP10	1	Motor delay
ARHGAP12	1	Echolalia
ARHGAP17	1	Echolalia
ARHGAP18	1	Echolalia
ARHGAP19	1	Echolalia
ARHGAP20	1	Echolalia
ARHGAP21	1	Echolalia
ARHGAP26	1	Echolalia
ARHGAP32	1	Echolalia
ARHGAP33	1	Echolalia
ARHGAP39	1	Echolalia
ARHGAP40	1	Echolalia
ARHGAP44	1	Echolalia
ARHGAP8	1	Echolalia
ARHGEF1	1	Echolalia
ARHGEF12	1	Echolalia
ARHGEF15	1	Echolalia
ARHGEF25	1	Echolalia
ARHGEF28	1	Echolalia
ARHGEF3	1	Echolalia
ARHGEF35	1	Echolalia
ARHGEF37	1	Echolalia
ARHGEF4	1	Echolalia
ARHGEF40	1	Echolalia
ARID1A	1	Echolalia
ARID1B	1	Echolalia
ARID3B	1	Echolalia
ARID5B	1	Echolalia
10,00 ARL	1	Echolalia
ARL6IP6	1	Echolalia
ARMC1	1	Echolalia
ARMC2	1	Echolalia
ARMC4	1	Echolalia
ARMC5	1	Echolalia
ARMC6	1	Echolalia
ARMC9	1	Echolalia
ARMCX2	1	Echolalia

ARMCX3	1	Echolalia
ARMCX4	1	Echolalia
ARMH1	1	Motor delay
ARMH2	1	Echolalia
ARMT1	1	Echolalia
ARNT2	1	Echolalia
ARNTL	1	Echolalia
ARPC4-TTLL3	1	Motor delay
ARPC5	1	Echolalia
ARRB2	1	Echolalia
ARRDC1	1	Echolalia
ARRDC2	1	Echolalia
ARRDC4	1	Echolalia
ARSA	1	Echolalia
ARSB	1	Echolalia
ARSD	1	Echolalia
ARSG	1	Echolalia
ARSI	1	Echolalia
ARSK	1	Echolalia
ART3	1	Echolalia
ART5	1	Echolalia
ARVCF	1	Echolalia
ASAP1	1	Echolalia
ASB1	1	Anxiety
ASB10	1	Echolalia
ASB12	1	Echolalia
ASB14	1	Unresponsiveness to spoken voice
ASB16	1	Echolalia
ASB18	1	Echolalia
ASB3	1	Echolalia
ASB6	1	Motor delay
ASB7	1	Echolalia
ASCC1	1	Echolalia
ASCL3	1	Echolalia
ASCL5	1	Echolalia
ASF1B	1	Echolalia
ASGR2	1	Echolalia
ASIC1	1	Unresponsiveness to spoken voice
ASIC2	1	Echolalia
ASNS	1	Echolalia
ASNSD1	1	Echolalia
ASPDH	1	Echolalia
ASPG	1	Echolalia
ASPH	1	Anxiety
ASTE1	1	Echolalia
ASTL	1	Echolalia

ASTN1	1	Echolalia
ASXL2	1	Echolalia
ATAD2	1	Echolalia
ATAD2B	1	Echolalia
ATAD3A	1	Echolalia
ATAD3B	1	Echolalia
ATE1	1	Echolalia
ATF1	1	Echolalia
ATF4	1	Echolalia
ATF5	1	Echolalia
ATF6	1	Echolalia
ATG10	1	Echolalia
ATG13	1	Motor delay
ATG14	1	Echolalia
ATG16L2	1	Unresponsiveness to spoken voice
ATG2B	1	Echolalia
ATG4C	1	Echolalia
ATG4D	1	Language disorder
ATIC	1	Echolalia
ATMIN	1	Echolalia
ATN1	1	Echolalia
ATOH8	1	Echolalia
ATP10A	1	Echolalia
ATP11B	1	Echolalia
ATP12A	1	Echolalia
ATP13A2	1	Echolalia
ATP13A5	1	Echolalia
ATP1A1	1	Echolalia
ATP1A3	1	Echolalia
ATP1A4	1	Echolalia
ATP2B4	1	Echolalia
ATP2C1	1	Echolalia
ATP2C2	1	Echolalia
ATP4A	1	Echolalia
ATP5MC3	1	Echolalia
ATP5MF-PTCD1	1	Echolalia
ATP5MPL	1	Echolalia
ATP5PB	1	Echolalia
ATP5PO	1	Echolalia
ATP6AP1L	1	Echolalia
ATP6V0A2	1	Echolalia
ATP6V0A4	1	Echolalia
ATP6V0B	1	Echolalia
ATP6V1B1	1	Echolalia
ATP6V1B1-AS1	1	Echolalia
ATP6V1C2	1	Echolalia

ATP7B	1	Echolalia
ATP8A2	1	Echolalia
ATP8B3	1	Echolalia
ATP9A	1	Echolalia
ATP9B	1	Echolalia
ATPSCKMT	1	Echolalia
ATR	1	Echolalia
ATRAID	1	Echolalia
ATRN	1	Echolalia
ATRNL1	1	Echolalia
ATRX	1	Unresponsiveness to spoken voice
ATXN1	1	Echolalia
ATXN10	1	Echolalia
ATXN1L	1	Echolalia
ATXN2	1	Echolalia
ATXN3	1	Echolalia
ATXN3L	1	Echolalia
AUNIP	1	Motor delay
AUP1	1	Echolalia
AURKA	1	Echolalia
AURKAIP1	1	Echolalia
AURKB	1	Echolalia
AVPR1A	1	Echolalia
AVPR1B	1	Echolalia
AWAT2	1	Echolalia
AXIN1	1	Echolalia
AXIN2	1	Echolalia
AXL	1	Aggressive behaviour
AZIN1	1	Echolalia
B3GALNT1	1	Echolalia
B3GALT2	1	Echolalia
B3GAT2	1	Echolalia
B3GLCT	1	Echolalia
B3GNT10	1	Echolalia
B3GNT6	1	Echolalia
B3GNT7	1	Echolalia
B4GALNT2	1	Echolalia
B4GALNT3	1	Echolalia
B4GALNT4	1	Echolalia
B4GALT4	1	Echolalia
B4GALT7	1	Echolalia
B9D1	1	Motor delay
BAALC	1	Echolalia
BABAM1	1	Echolalia
BAD	1	Echolalia
BAG1	1	Anxiety

BAG4	1	Echolalia
BAGE3	1	Echolalia
BAIAP2	1	Echolalia
BANK1	1	Echolalia
BAP1	1	Echolalia
BARD1	1	Echolalia
BAZ1A	1	Echolalia
BAZ2B	1	Echolalia
BBS1	1	Echolalia
BBS10	1	Echolalia
BBS9	1	Echolalia
BBX	1	Echolalia
BCAM	1	Echolalia
BCAR1	1	Echolalia
BCAS1	1	Echolalia
BCAT1	1	Echolalia
BCAT2	1	Motor delay
BCDIN3D	1	Echolalia
BCHE	1	Echolalia
BCKDHA	1	Echolalia
BCKDK	1	Motor delay
BCL10	1	Echolalia
BCL11B	1	Echolalia
BCL2A1	1	Echolalia
BCL2L11	1	Echolalia
BCL2L13	1	Echolalia
BCL2L14	1	Echolalia
BCL3	1	Echolalia
BCL7A	1	Echolalia
BCL9	1	Echolalia
BCL9L	1	Echolalia
BCLAF1	1	Echolalia
BCOR	1	Echolalia
BCORL1	1	Aggressive behaviour
BCS1L	1	Echolalia
BDKRB1	1	Echolalia
BDKRB2	1	Echolalia
BDNF	1	Echolalia
BEND3	1	Echolalia
BEND7	1	Echolalia
BEST2	1	Echolalia
BEST4	1	Echolalia
BGN	1	Unresponsiveness to spoken voice
BHLHA15	1	Language disorder
BHLHA9	1	Echolalia
BICC1	1	Echolalia

BICD1	1	Echolalia
BICDL1	1	Echolalia
BICRAL	1	Echolalia
BIN1	1	Echolalia
BIN3	1	Echolalia
BIRC7	1	Echolalia
BIRC8	1	Echolalia
BIVM-ERCC5	1	Echolalia
BLK	1	Echolalia
BLMH	1	Echolalia
BLNK	1	Echolalia
BLOC1S2	1	Echolalia
BLOC1S3	1	Echolalia
BLOC1S5	1	Echolalia
BLVRA	1	Echolalia
BLZF1	1	Echolalia
BMP1	1	Echolalia
BMP2K	1	Echolalia
BMP3	1	Echolalia
BMP4	1	Echolalia
BMP6	1	Echolalia
BMP7	1	Echolalia
BMP8A	1	Echolalia
BMP8B	1	Echolalia
BMS1P20	1	Echolalia
BMS1P22	1	Echolalia
BNIP1	1	Echolalia
BNIP3L	1	Sleep disturbances
BNIPL	1	Echolalia
BOC	1	Echolalia
BOLL	1	Echolalia
BORA	1	Echolalia
BORCS7-ASMT	1	Echolalia
BPHL	1	Echolalia
BPIFB1	1	Echolalia
BPIFB4	1	Echolalia
BPIFB6	1	Echolalia
BPTF	1	Echolalia
BRD1	1	Echolalia
BRD8	1	Echolalia
BRD9	1	Echolalia
BRDT	1	Echolalia
BRINP1	1	Echolalia
BRINP2	1	Echolalia
BRINP3	1	Echolalia
BRWD1	1	Echolalia

BSG	1	Echolalia
BSN	1	Echolalia
BST1	1	Echolalia
BTBD10	1	Echolalia
BTBD11	1	Echolalia
BTBD16	1	Echolalia
BTBD19	1	Echolalia
BTBD8	1	Echolalia
BTBD9	1	Anxiety
BTD	1	Echolalia
BTN1A1	1	Echolalia
BTN3A1	1	Echolalia
BTN3A3	1	Echolalia
BTNL3	1	Echolalia
BTNL8	1	Echolalia
BTNL9	1	Echolalia
BUB1B-PAK6	1	Echolalia
BVES	1	Echolalia
BYSL	1	Echolalia
BZW2	1	Unresponsiveness to spoken voice
C10orf105	1	Echolalia
C10orf143	1	Echolalia
C10orf53	1	Echolalia
C10orf62	1	Echolalia
C10orf67	1	Echolalia
C10orf71	1	Echolalia
C10orf99	1	Language disorder
C11orf21	1	Echolalia
C11orf24	1	Echolalia
C11orf42	1	Echolalia
C11orf45	1	Echolalia
C11orf52	1	Anxiety
C11orf68	1	Anxiety
C11orf91	1	Echolalia
C12orf43	1	Echolalia
C12orf45	1	Echolalia
C12orf50	1	Echolalia
C12orf56	1	Echolalia
C12orf60	1	Echolalia
C12orf76	1	Echolalia
C13orf42	1	Echolalia
C13orf46	1	Echolalia
C14orf178	1	Echolalia
C14orf39	1	Echolalia
C15orf32	1	Echolalia
C15orf40	1	Echolalia

C15orf54	1	Echolalia
C16orf46	1	Echolalia
C16orf71	1	Echolalia
C16orf72	1	Echolalia
C16orf95	1	Echolalia
C16orf96	1	Echolalia
C17orf102	1	Echolalia
C17orf50	1	Aggressive behaviour
C17orf64	1	Echolalia
C18orf12	1	Echolalia
C18orf63	1	Echolalia
C19orf12	1	Echolalia
C19orf44	1	Echolalia
C19orf48	1	Echolalia
C19orf54	1	Echolalia
C19orf57	1	Echolalia
C19orf71	1	Echolalia
C19orf73	1	Echolalia
C19orf81	1	Echolalia
C1QA	1	Anxiety
C1QL1	1	Echolalia
C1QL3	1	Echolalia
C1QTNF2	1	Echolalia
C1QTNF4	1	Language disorder
C1QTNF6	1	Echolalia
C1QTNF8	1	Echolalia
C1QTNF9	1	Echolalia
C1QTNF9B	1	Echolalia
C1R	1	Echolalia
C1orf105	1	Echolalia
C1orf112	1	Echolalia
C1orf116	1	Echolalia
C1orf131	1	Echolalia
C1orf141	1	Echolalia
C1orf159	1	Echolalia
C1orf167	1	Echolalia
C1orf174	1	Echolalia
C1orf195	1	Echolalia
C1orf198	1	Echolalia
C1orf229	1	Echolalia
C1orf35	1	Echolalia
C1orf54	1	Anxiety
C1orf68	1	Echolalia
C1orf87	1	Echolalia
C20orf203	1	Echolalia
C20orf96	1	Echolalia

C21orf58	1	Echolalia
C21orf91	1	Echolalia
C22orf24	1	Echolalia
C22orf31	1	Aggressive behaviour
C22orf34	1	Echolalia
C22orf42	1	Echolalia
C22orf46	1	Echolalia
C2CD2L	1	Language disorder
C2CD4B	1	Echolalia
C2orf16	1	Echolalia
C2orf42	1	Echolalia
C2orf49	1	Echolalia
C2orf72	1	Echolalia
C2orf74	1	Echolalia
C2orf78	1	Echolalia
C2orf91	1	Echolalia
C3AR1	1	Echolalia
C3orf20	1	Echolalia
C3orf22	1	Echolalia
C3orf33	1	Echolalia
C3orf38	1	Echolalia
C3orf52	1	Motor delay
C3orf80	1	Echolalia
C4BPA	1	Echolalia
C4orf17	1	Echolalia
C4orf33	1	Echolalia
C4orf36	1	Echolalia
C4orf47	1	Echolalia
C4orf50	1	Echolalia
C4orf54	1	Echolalia
C5	1	Echolalia
C5orf22	1	Echolalia
C5orf34	1	Aggressive behaviour
C5orf38	1	Echolalia
C5orf47	1	Echolalia
C5orf58	1	Sleep disturbances
C5orf64	1	Echolalia
C6	1	Echolalia
C6orf141	1	Echolalia
C6orf223	1	Echolalia
C6orf226	1	Echolalia
C6orf58	1	Echolalia
C6orf89	1	Echolalia
C7	1	Echolalia
C7orf25	1	Echolalia
C7orf31	1	Echolalia

C7orf33	1	Echolalia
C7orf50	1	Echolalia
C7orf57	1	Echolalia
C8A	1	Echolalia
C8G	1	Echolalia
C8orf31	1	Echolalia
C8orf37-AS1	1	Unresponsiveness to spoken voice
C8orf44	1	Echolalia
C8orf48	1	Echolalia
C8orf49	1	Echolalia
C8orf58	1	Echolalia
C8orf74	1	Echolalia
C8orf86	1	Echolalia
C8orf87	1	Echolalia
C9	1	Echolalia
C9orf106	1	Echolalia
C9orf131	1	Echolalia
C9orf135	1	Echolalia
C9orf147	1	Echolalia
C9orf152	1	Echolalia
C9orf153	1	Echolalia
C9orf43	1	Echolalia
C9orf47	1	Echolalia
C9orf62	1	Echolalia
CA1	1	Echolalia
CA10	1	Echolalia
CA12	1	Echolalia
CA6	1	Echolalia
CA9	1	Echolalia
CAAP1	1	Echolalia
CAB39L	1	Echolalia
CABIN1	1	Echolalia
CABLES1	1	Echolalia
CABLES2	1	Echolalia
CABP1	1	Echolalia
CACNA1B	1	Echolalia
CACNA1D	1	Echolalia
CACNA1E	1	Echolalia
CACNA1F	1	Echolalia
CACNA1H	1	Echolalia
CACNA1S	1	Echolalia
CACNA2D1	1	Echolalia
CACNA2D2	1	Echolalia
CACNA2D4	1	Echolalia
CACNB2	1	Echolalia
CACNG6	1	Echolalia

CACNG7	1	Anxiety
CACTIN	1	Motor delay
CADM1	1	Echolalia
CADPS	1	Echolalia
CALCA	1	Echolalia
CALCOCO1	1	Echolalia
CALD1	1	Echolalia
CALHM1	1	Echolalia
CALHM2	1	Echolalia
CALHM3	1	Motor delay
CALHM4	1	Echolalia
CALHM6	1	Echolalia
CALML5	1	Echolalia
CALML6	1	Echolalia
CALU	1	Echolalia
CALY	1	Echolalia
CAMK1G	1	Echolalia
CAMK2G	1	Echolalia
CAMKK2	1	Echolalia
CAMKV	1	Echolalia
CAMSAP1	1	Echolalia
CAMSAP2	1	Echolalia
CAMSAP3	1	Echolalia
CAMTA1	1	Echolalia
CAMTA2	1	Echolalia
CAND2	1	Echolalia
CAPG	1	Echolalia
CAPN10	1	Echolalia
CAPN13	1	Echolalia
CAPN14	1	Echolalia
CAPN15	1	Echolalia
CAPN2	1	Echolalia
CAPN3	1	Echolalia
CAPN5	1	Echolalia
CAPN9	1	Echolalia
CAPNS1	1	Echolalia
CAPZA1	1	Echolalia
CARD10	1	Echolalia
CARD11	1	Echolalia
CARD19	1	Echolalia
CARHSP1	1	Echolalia
CARMIL1	1	Echolalia
CARNS1	1	Echolalia
CARS2	1	Echolalia
CASC3	1	Echolalia
CASD1	1	Echolalia

CASKIN1	1	Echolalia
CASKIN2	1	Echolalia
CASP7	1	Echolalia
CASP8	1	Echolalia
CAT	1	Echolalia
CATSPER4	1	Echolalia
CATSPERD	1	Echolalia
CATSPERE	1	Echolalia
CAV1	1	Echolalia
CAV3	1	Echolalia
CAVIN1	1	Echolalia
CAVIN2	1	Echolalia
CBFA2T3	1	Echolalia
CBL	1	Echolalia
CBLC	1	Echolalia
CBLIF	1	Echolalia
CBLL2	1	Sleep disturbances
CBR3	1	Echolalia
CBWD1	1	Echolalia
CBWD6	1	Echolalia
CBX2	1	Echolalia
CBY3	1	Unresponsiveness to spoken voice
CC2D1A	1	Echolalia
CC2D1B	1	Echolalia
CC2D2B	1	Echolalia
CCAR1	1	Echolalia
CCAR2	1	Echolalia
CCDC105	1	Echolalia
CCDC106	1	Echolalia
CCDC110	1	Echolalia
CCDC114	1	Echolalia
CCDC116	1	Echolalia
CCDC127	1	Echolalia
CCDC13	1	Echolalia
CCDC142	1	Echolalia
CCDC144NL	1	Echolalia
CCDC146	1	Echolalia
CCDC149	1	Echolalia
CCDC150	1	Echolalia
CCDC151	1	Echolalia
CCDC154	1	Echolalia
CCDC157	1	Echolalia
CCDC158	1	Echolalia
CCDC168	1	Echolalia
CCDC169	1	Echolalia

CCDC169-SOHLH2	1	Echolalia
CCDC17	1	Echolalia
CCDC173	1	Echolalia
CCDC175	1	Echolalia
CCDC178	1	Echolalia
CCDC18	1	Echolalia
CCDC180	1	Echolalia
CCDC181	1	Echolalia
CCDC182	1	Language disorder
CCDC184	1	Echolalia
CCDC185	1	Echolalia
CCDC187	1	Echolalia
CCDC188	1	Echolalia
CCDC198	1	Echolalia
CCDC24	1	Echolalia
CCDC27	1	Echolalia
CCDC28B	1	Echolalia
CCDC30	1	Echolalia
CCDC32	1	Echolalia
CCDC38	1	Echolalia
CCDC50	1	Echolalia
CCDC51	1	Echolalia
CCDC57	1	Echolalia
CCDC6	1	Echolalia
CCDC60	1	Echolalia
CCDC62	1	Motor delay
CCDC63	1	Unresponsiveness to spoken voice
CCDC65	1	Unresponsiveness to spoken voice
CCDC66	1	Echolalia
CCDC69	1	Echolalia
CCDC7	1	Echolalia
CCDC73	1	Echolalia
CCDC74A	1	Echolalia
CCDC8	1	Echolalia
CCDC80	1	Echolalia
CCDC82	1	Echolalia
CCDC83	1	Echolalia
CCDC85C	1	Echolalia
CCDC88A	1	Echolalia
CCDC88B	1	Echolalia
CCDC9	1	Echolalia
CCDC90B	1	Echolalia
CCDC91	1	Echolalia
CCDC92B	1	Echolalia
CCDC93	1	Echolalia

CCIN	1	Echolalia
CCL13	1	Echolalia
CCL17	1	Echolalia
CCL2	1	Echolalia
CCL22	1	Echolalia
CCL23	1	Echolalia
CCL24	1	Echolalia
CCL25	1	Echolalia
CCL27	1	Echolalia
CCM2	1	Echolalia
CCN1	1	Echolalia
CCN3	1	Echolalia
CCN4	1	Echolalia
CCN6	1	Echolalia
CCNB1	1	Unresponsiveness to spoken voice
CCND1	1	Echolalia
CCND3	1	Echolalia
CCNE1	1	Echolalia
CCNG2	1	Echolalia
CCNJL	1	Echolalia
CCNO	1	Echolalia
CCNT2	1	Echolalia
CCNY	1	Echolalia
CCPG1	1	Echolalia
CCR3	1	Echolalia
CCR6	1	Echolalia
CCR7	1	Echolalia
CCR9	1	Echolalia
CCRL2	1	Echolalia
CCS	1	Echolalia
CCT6A	1	Echolalia
CCT8L2	1	Unresponsiveness to spoken voice
CCZ1	1	Echolalia
CD101	1	Echolalia
CD109	1	Echolalia
CD14	1	Echolalia
CD163	1	Echolalia
CD163L1	1	Echolalia
CD177	1	Echolalia
CD19	1	Echolalia
CD1A	1	Echolalia
CD200	1	Echolalia
CD200R1	1	Motor delay
CD200R1L	1	Echolalia
CD209	1	Echolalia
CD22	1	Echolalia

CD226	1	Aggressive behaviour
CD247	1	Echolalia
CD27-AS1	1	Echolalia
CD276	1	Echolalia
CD2AP	1	Echolalia
CD300C	1	Echolalia
CD300H	1	Echolalia
CD300LF	1	Echolalia
CD37	1	Echolalia
CD3G	1	Echolalia
CD48	1	Echolalia
CD5	1	Echolalia
CD52	1	Echolalia
CD6	1	Echolalia
CD68	1	Echolalia
CD83	1	Echolalia
CD86	1	Echolalia
CD8B2	1	Echolalia
CD93	1	Echolalia
CD96	1	Echolalia
CD99L2	1	Unresponsiveness to spoken voice
CDA	1	Echolalia
CDAN1	1	Echolalia
CDC14C	1	Echolalia
CDC16	1	Echolalia
CDC25A	1	Echolalia
CDC25B	1	Echolalia
CDC27	1	Echolalia
CDC42BPA	1	Echolalia
CDC42EP1	1	Echolalia
CDC7	1	Echolalia
CDC73	1	Echolalia
CDCA2	1	Echolalia
CDCA4	1	Echolalia
CDCA5	1	Language disorder
CDH13	1	Echolalia
CDH18	1	Echolalia
CDH23	1	Echolalia
CDH24	1	Echolalia
CDH3	1	Echolalia
CDH4	1	Echolalia
CDH6	1	Echolalia
CDH7	1	Echolalia
CDH9	1	Unresponsiveness to spoken voice
CDHR1	1	Echolalia
CDHR3	1	Echolalia

CDHR4	1	Echolalia
CDIN1	1	Echolalia
CDK10	1	Echolalia
CDK11A	1	Echolalia
CDK14	1	Echolalia
CDK15	1	Echolalia
CDK18	1	Echolalia
CDK2	1	Echolalia
CDK20	1	Echolalia
CDK4	1	Echolalia
CDK5R1	1	Unresponsiveness to spoken voice
CDK5R2	1	Echolalia
CDK5RAP1	1	Echolalia
CDK5RAP3	1	Echolalia
CDKL1	1	Echolalia
CDKL4	1	Echolalia
CDKL5	1	Echolalia
CDKN2A	1	Echolalia
CDNF	1	Echolalia
CDON	1	Echolalia
CDPF1	1	Echolalia
CDRT1	1	Echolalia
CDRT15	1	Echolalia
CDRT4	1	Echolalia
CDSN	1	Echolalia
CDT1	1	Echolalia
CDX2	1	Echolalia
CDYL2	1	Aggressive behaviour
CEACAM1	1	Language disorder
CEACAM16	1	Echolalia
CEACAM19	1	Echolalia
CEACAM20	1	Echolalia
CEACAM7	1	Anxiety
CEACAM8	1	Echolalia
CEBPD	1	Echolalia
CEBPZ	1	Echolalia
CELA2A	1	Echolalia
CELF2	1	Echolalia
CELSR1	1	Echolalia
CELSR3	1	Echolalia
CEMIP	1	Echolalia
CEMP1	1	Echolalia
CENPC	1	Echolalia
CENPM	1	Echolalia
CENPN	1	Echolalia
CEP104	1	Echolalia

CEP120	1	Echolalia
CEP126	1	Echolalia
CEP128	1	Echolalia
CEP135	1	Echolalia
CEP152	1	Echolalia
CEP162	1	Echolalia
CEP164	1	Echolalia
CEP170B	1	Echolalia
CEP250	1	Echolalia
CEP290	1	Echolalia
CEP295	1	Echolalia
CEP295NL	1	Echolalia
CEP350	1	Echolalia
CEP41	1	Echolalia
CEP43	1	Echolalia
CEP44	1	Echolalia
CEP55	1	Echolalia
CEP57	1	Echolalia
CEP63	1	Echolalia
CEP70	1	Echolalia
CEP72	1	Echolalia
CEP83	1	Echolalia
CEP85	1	Echolalia
CEP89	1	Echolalia
CEP97	1	Language disorder
CEPT1	1	Echolalia
CERKL	1	Echolalia
CERS3	1	Echolalia
CERS5	1	Echolalia
CERT1	1	Echolalia
CES1	1	Echolalia
CES1P1	1	Echolalia
CES3	1	Echolalia
CES4A	1	Echolalia
CES5A	1	Echolalia
CFAP157	1	Echolalia
CFAP161	1	Echolalia
CFAP206	1	Echolalia
CFAP20DC	1	Echolalia
CFAP221	1	Echolalia
CFAP251	1	Echolalia
CFAP298-TCP10L	1	Echolalia
CFAP299	1	Echolalia
CFAP410	1	Echolalia
CFAP43	1	Echolalia
CFAP45	1	Echolalia

CFAP46	1	Echolalia
CFAP47	1	Echolalia
CFAP52	1	Echolalia
CFAP54	1	Echolalia
CFAP57	1	Echolalia
CFAP61	1	Echolalia
CFAP70	1	Echolalia
CFAP73	1	Echolalia
CFAP74	1	Echolalia
CFAP77	1	Echolalia
CFAP99	1	Echolalia
CFHR1	1	Echolalia
CFI	1	Echolalia
CFL1	1	Unresponsiveness to spoken voice
CFTR	1	Echolalia
CGAS	1	Echolalia
CGB1	1	Echolalia
CGB3	1	Echolalia
CGB8	1	Echolalia
CGN	1	Echolalia
CH25H	1	Echolalia
CHAC1	1	Motor delay
CHADL	1	Echolalia
CHAF1A	1	Echolalia
CHAF1B	1	Echolalia
CHAMP1	1	Echolalia
CHD1	1	Unresponsiveness to spoken voice
CHD2	1	Echolalia
CHD5	1	Echolalia
CHD6	1	Echolalia
CHD7	1	Echolalia
CHD8	1	Echolalia
CHD9	1	Echolalia
CHDH	1	Echolalia
CHEK1	1	Echolalia
CHFR	1	Echolalia
CHGB	1	Echolalia
CHI3L2	1	Echolalia
CHIA	1	Echolalia
CHID1	1	Echolalia
CHIT1	1	Echolalia
CHML	1	Echolalia
CHMP2B	1	Echolalia
CHMP4C	1	Echolalia
CHPF	1	Echolalia
CHPF2	1	Echolalia

CHRM3	1	Echolalia
CHRM4	1	Echolalia
CHRNA1	1	Echolalia
CHRNA10	1	Echolalia
CHRNA2	1	Echolalia
CHRNA4	1	Echolalia
CHRNA9	1	Echolalia
CHRNB1	1	Echolalia
CHRNB4	1	Echolalia
CHRNE	1	Echolalia
CHRNG	1	Echolalia
CHST10	1	Aggressive behaviour
CHST12	1	Echolalia
CHST3	1	Echolalia
CHST7	1	Echolalia
CHST9	1	Language disorder
CHTF18	1	Echolalia
CIAO2A	1	Anxiety
CIAO3	1	Echolalia
CIAPIN1	1	Echolalia
CIB3	1	Echolalia
CIB4	1	Echolalia
CIBAR2	1	Echolalia
CIC	1	Echolalia
CIDEA	1	Echolalia
CIDEB	1	Echolalia
CIDEC	1	Echolalia
CILP	1	Echolalia
CILP2	1	Echolalia
CIP2A	1	Echolalia
CIPC	1	Mutism
CIRBP	1	Echolalia
CISD2	1	Echolalia
CIT	1	Echolalia
CIZ1	1	Echolalia
CKAP2L	1	Echolalia
CKAP4	1	Echolalia
CKAP5	1	Echolalia
CKB	1	Echolalia
CKMT1B	1	Echolalia
CLASP1	1	Echolalia
CLC	1	Echolalia
CLCA2	1	Echolalia
CLCA4	1	Echolalia
CLCC1	1	Echolalia
CLCN1	1	Echolalia

CLCN2	1	Echolalia
CLCN3	1	Echolalia
CLCN5	1	Unresponsiveness to spoken voice
CLCN6	1	Echolalia
CLCNKA	1	Echolalia
CLDN1	1	Echolalia
CLDN12	1	Echolalia
CLDN16	1	Language disorder
CLDN17	1	Echolalia
CLDN18	1	Motor delay
CLDN19	1	Echolalia
CLDN23	1	Echolalia
CLDN34	1	Echolalia
CLDN6	1	Echolalia
CLDN7	1	Echolalia
CLDN8	1	Echolalia
CLEC10A	1	Echolalia
CLEC12A	1	Echolalia
CLEC12B	1	Echolalia
CLEC16A	1	Echolalia
CLEC18A	1	Echolalia
CLEC18B	1	Echolalia
CLEC1A	1	Echolalia
CLEC1B	1	Echolalia
CLEC2D	1	Echolalia
CLEC3B	1	Echolalia
CLEC4F	1	Echolalia
CLEC4M	1	Echolalia
CLGN	1	Echolalia
CLHC1	1	Echolalia
CLIP1	1	Echolalia
CLIP2	1	Echolalia
CLIP3	1	Echolalia
CLK3	1	Echolalia
CLK4	1	Echolalia
CLNK	1	Echolalia
CLNS1A	1	Echolalia
CLOCK	1	Echolalia
CLPP	1	Language disorder
CLPSL1	1	Echolalia
CLPTM1L	1	Echolalia
CLRN3	1	Echolalia
CLSTN2	1	Echolalia
CLSTN3	1	Echolalia
CLTB	1	Echolalia
CLTCL1	1	Echolalia

CLU	1	Echolalia
CLUAP1	1	Echolalia
CMC2	1	Echolalia
CMKLR1	1	Language disorder
CMPK2	1	Echolalia
CMSS1	1	Echolalia
CMTM2	1	Echolalia
CMTM7	1	Echolalia
CMTR2	1	Echolalia
CNBD1	1	Echolalia
CNDP1	1	Echolalia
CNGA1	1	Echolalia
CNGA3	1	Echolalia
CNGA4	1	Echolalia
CNIH3	1	Echolalia
CNKSR3	1	Echolalia
CNN1	1	Echolalia
CNN2	1	Echolalia
CNNM1	1	Echolalia
CNNM2	1	Echolalia
CNNM4	1	Aggressive behaviour
CNOT1	1	Echolalia
CNOT10	1	Motor delay
CNOT4	1	Echolalia
CNP	1	Echolalia
CNPPD1	1	Echolalia
CNPY1	1	Echolalia
CNR2	1	Echolalia
CNTF	1	Unresponsiveness to spoken voice
CNTN1	1	Echolalia
CNTN3	1	Echolalia
CNTN4	1	Echolalia
CNTNAP3	1	Echolalia
COA6	1	Motor delay
COASY	1	Echolalia
COBLL1	1	Echolalia
COG5	1	Echolalia
COG8	1	Anxiety
COIL	1	Echolalia
COL10A1	1	Echolalia
COL12A1	1	Echolalia
COL13A1	1	Echolalia
COL14A1	1	Echolalia
COL15A1	1	Echolalia
COL17A1	1	Echolalia
COL18A1	1	Echolalia

COL1A2	1	Echolalia
COL20A1	1	Echolalia
COL22A1	1	Echolalia
COL23A1	1	Echolalia
COL25A1	1	Echolalia
COL26A1	1	Echolalia
COL27A1	1	Echolalia
COL28A1	1	Echolalia
COL2A1	1	Echolalia
COL4A1	1	Echolalia
COL4A2-AS2	1	Echolalia
COL4A4	1	Echolalia
COL4A5	1	Motor delay
COL4A6	1	Echolalia
COL5A1	1	Echolalia
COL5A3	1	Echolalia
COL6A1	1	Echolalia
COL6A2	1	Echolalia
COL6A3	1	Echolalia
COL7A1	1	Echolalia
COLCA1	1	Echolalia
COLCA2	1	Unresponsiveness to spoken voice
COLEC11	1	Echolalia
COLEC12	1	Echolalia
COLGALT2	1	Echolalia
COLQ	1	Motor delay
COMMD10	1	Echolalia
COMMD4P1	1	Echolalia
COMMD7	1	Echolalia
COMP	1	Anxiety
COPS9	1	Echolalia
COQ10A	1	Echolalia
COQ2	1	Echolalia
COQ3	1	Echolalia
COQ5	1	Echolalia
COQ7	1	Echolalia
COQ8A	1	Echolalia
COQ8B	1	Echolalia
COQ9	1	Language disorder
CORIN	1	Echolalia
CORO2A	1	Motor delay
COX10	1	Anxiety
COX15	1	Echolalia
COX17	1	Echolalia
COX20	1	Echolalia
COX4I1	1	Mutism

COX7A2L	1	Echolalia
CP	1	Echolalia
CPA3	1	Aggressive behaviour
CPA4	1	Echolalia
CPA5	1	Echolalia
CPA6	1	Echolalia
CPD	1	Echolalia
CPEB2	1	Echolalia
CPHXL	1	Echolalia
CPLANE1	1	Echolalia
CPM	1	Echolalia
CPN2	1	Echolalia
CPNE1	1	Echolalia
CPNE2	1	Echolalia
CPNE3	1	Echolalia
CPNE7	1	Echolalia
CPO	1	Echolalia
CPPED1	1	Echolalia
CPQ	1	Echolalia
CPSF1	1	Echolalia
CPT1B	1	Echolalia
CPT1C	1	Echolalia
CPTP	1	Echolalia
CPVL	1	Echolalia
CPXCR1	1	Echolalia
CPXM1	1	Echolalia
CPZ	1	Echolalia
CR1	1	Echolalia
CRACD	1	Echolalia
CRACDL	1	Echolalia
CRB1	1	Echolalia
CRB2	1	Echolalia
CRB3	1	Motor delay
CRCP	1	Echolalia
CREB3L2	1	Echolalia
CREB3L3	1	Echolalia
CREB3L4	1	Echolalia
CREB5	1	Echolalia
CREBRF	1	Echolalia
CREG2	1	Echolalia
CREM	1	Echolalia
CRHR2	1	Echolalia
CRIP1	1	Echolalia
CRLF3	1	Echolalia
CRLS1	1	Echolalia
CRNKL1	1	Echolalia

CRNN	1	Echolalia
CROCC	1	Echolalia
CRP	1	Echolalia
CRTAC1	1	Echolalia
CRTAP	1	Echolalia
CRTC2	1	Echolalia
CRTC3	1	Echolalia
CRX	1	Echolalia
CRY2	1	Echolalia
CRYBA4	1	Echolalia
CRYBB2	1	Language disorder
CRYBB3	1	Echolalia
CRYBG2	1	Echolalia
CRYBG3	1	Echolalia
CRYGD	1	Echolalia
CRYM	1	Echolalia
CSAD	1	Echolalia
CSF2RB	1	Echolalia
CSGALNACT2	1	Echolalia
CSK	1	Echolalia
CSMD1	1	Echolalia
CSMD2	1	Echolalia
CSMD3	1	Echolalia
CSN1S1	1	Echolalia
CSNK1D	1	Echolalia
CSNK1E	1	Echolalia
CSNK1G2	1	Echolalia
CSPP1	1	Echolalia
CST1	1	Echolalia
CST2	1	Echolalia
CST8	1	Echolalia
CSTA	1	Echolalia
CSTF2T	1	Echolalia
CTAG2	1	Echolalia
CTAGE1	1	Echolalia
CTAGE15	1	Echolalia
CTAGE9	1	Echolalia
CTBS	1	Echolalia
CTC1	1	Echolalia
CTDNEP1	1	Echolalia
CTIF	1	Echolalia
CTLA4	1	Echolalia
CTNNA1	1	Echolalia
CTNND2	1	Echolalia
CTPS2	1	Echolalia
CTR9	1	Echolalia

CTRB1	1	Echolalia
CTRL	1	Echolalia
CTSA	1	Echolalia
CTSD	1	Echolalia
CTSE	1	Echolalia
CTSH	1	Echolalia
CTSK	1	Echolalia
CTSL	1	Echolalia
CTTNBP2	1	Echolalia
CTTNBP2NL	1	Echolalia
CTU1	1	Echolalia
CTU2	1	Echolalia
CTXND1	1	Echolalia
CUBN	1	Echolalia
CUEDC1	1	Echolalia
CUL4A	1	Echolalia
CUL5	1	Motor delay
CUL7	1	Echolalia
CUL9	1	Echolalia
CWC22	1	Echolalia
CWC27	1	Echolalia
CWF19L1	1	Echolalia
CWF19L2	1	Echolalia
CWH43	1	Echolalia
CX3CR1	1	Echolalia
CXCL1	1	Language disorder
CXCL8	1	Echolalia
CXCR1	1	Echolalia
CXCR2	1	Echolalia
CXCR5	1	Echolalia
CXCR6	1	Echolalia
CXXC1	1	Echolalia
CXXC4	1	Echolalia
CXorf58	1	Echolalia
CYB5D2	1	Motor delay
CYB5R2	1	Echolalia
CYB5R4	1	Sleep disturbances
CYB5RL	1	Echolalia
CYC1	1	Echolalia
CYFIP1	1	Language disorder
CYGB	1	Echolalia
CYP11A1	1	Echolalia
CYP11B2	1	Echolalia
CYP17A1	1	Echolalia
CYP1A1	1	Echolalia
CYP20A1	1	Echolalia

CYP24A1	1	Echolalia
CYP26B1	1	Echolalia
CYP27B1	1	Echolalia
CYP2A13	1	Echolalia
CYP2A7	1	Echolalia
CYP2C19	1	Echolalia
CYP2C9	1	Echolalia
CYP2D7	1	Echolalia
CYP2J2	1	Echolalia
CYP2S1	1	Echolalia
CYP2U1	1	Echolalia
CYP2W1	1	Echolalia
CYP39A1	1	Echolalia
CYP3A43	1	Echolalia
CYP3A5	1	Echolalia
CYP3A7-CYP3A51P	1	Echolalia
CYP4B1	1	Echolalia
CYP4F11	1	Echolalia
CYP4F3	1	Echolalia
CYP4F8	1	Echolalia
CYP4X1	1	Echolalia
CYP4Z1	1	Echolalia
CYP7A1	1	Motor delay
CYP7B1	1	Echolalia
CYSTM1	1	Echolalia
CYTH3	1	Echolalia
CYTH4	1	Motor delay
CYTOR	1	Language disorder
D2HGDH	1	Echolalia
DAAM2	1	Echolalia
DAB1	1	Echolalia
DAB2IP	1	Echolalia
DACH2	1	Motor delay
DACT2	1	Echolalia
DAD1	1	Echolalia
DAG1	1	Echolalia
DALRD3	1	Echolalia
DAP	1	Echolalia
DAP3	1	Echolalia
DAPP1	1	Echolalia
DAZL	1	Echolalia
DBF4	1	Echolalia
DBF4B	1	Language disorder
DBNL	1	Echolalia
DBX2	1	Echolalia

DCAF12L2	1	Echolalia
DCAF15	1	Echolalia
DCAF17	1	Echolalia
DCAF4	1	Echolalia
DCAF6	1	Echolalia
DCAF8L2	1	Echolalia
DCANP1	1	Echolalia
DCBLD1	1	Echolalia
DCC	1	Echolalia
DCDC1	1	Echolalia
DCDC2	1	Echolalia
DCDC2C	1	Echolalia
DCK	1	Echolalia
DCLK3	1	Echolalia
DCLRE1A	1	Echolalia
DCLRE1CP1	1	Echolalia
DCP1A	1	Echolalia
DCST1	1	Echolalia
DCST2	1	Echolalia
DCSTAMP	1	Echolalia
DCT	1	Echolalia
DCTN5	1	Echolalia
DCUN1D2	1	Echolalia
DDI2	1	Echolalia
DDIAS	1	Echolalia
DDIT4L	1	Echolalia
DDO	1	Echolalia
DDX1	1	Echolalia
DDX11	1	Echolalia
DDX20	1	Echolalia
DDX25	1	Echolalia
DDX27	1	Echolalia
DDX31	1	Echolalia
DDX3X	1	Echolalia
DDX49	1	Echolalia
DDX50	1	Echolalia
DDX53	1	Aggressive behaviour
DDX54	1	Echolalia
DDX55	1	Echolalia
DDX60	1	Echolalia
DDX60L	1	Echolalia
DEAF1	1	Echolalia
DEDD2	1	Echolalia
DEF6	1	Echolalia
DEFB114	1	Echolalia
DEFB124	1	Echolalia

DEFB128	1	Echolalia
DEFB129	1	Echolalia
DEGS1	1	Language disorder
DEGS2	1	Echolalia
DEK	1	Echolalia
DELE1	1	Echolalia
DENND1A	1	Echolalia
DENND2B	1	Echolalia
DENND2C	1	Echolalia
DENND4C	1	Echolalia
DENND5A	1	Mutism
DENND6B	1	Echolalia
DEPDC1	1	Echolalia
DEPDC5	1	Echolalia
DEPDC7	1	Echolalia
DERL2	1	Echolalia
DESI2	1	Echolalia
DEXI	1	Unresponsiveness to spoken voice
DFFA	1	Echolalia
DGCR2	1	Echolalia
DGCR5	1	Echolalia
DGKA	1	Echolalia
DGKB	1	Echolalia
DGKD	1	Echolalia
DGKE	1	Echolalia
DGKH	1	Echolalia
DGKI	1	Echolalia
DGKQ	1	Echolalia
DGLUCY	1	Echolalia
DGUOK	1	Echolalia
DHCR7	1	Echolalia
DHDDS	1	Echolalia
DHDH	1	Echolalia
DHFR	1	Echolalia
DHFR2	1	Echolalia
DHODH	1	Echolalia
DHPS	1	Aggressive behaviour
DHRS1	1	Echolalia
DHRS12	1	Echolalia
DHRS13	1	Echolalia
DHRS3	1	Echolalia
DHRS4	1	Echolalia
DHRS4L2	1	Echolalia
DHTKD1	1	Echolalia
DHX15	1	Echolalia
DHX30	1	Echolalia

DHX33	1	Echolalia
DHX34	1	Echolalia
DHX35	1	Echolalia
DHX37	1	Echolalia
DHX57	1	Echolalia
DICER1	1	Echolalia
DIDO1	1	Echolalia
DIO2	1	Echolalia
DIP2A	1	Echolalia
DIP2B	1	Language disorder
DIP2C-AS1	1	Language disorder
DIPK1C	1	Unresponsiveness to spoken voice
DIPK2A	1	Echolalia
DIRAS3	1	Echolalia
DIS3	1	Echolalia
DIS3L2	1	Echolalia
DISP3	1	Echolalia
DLC1	1	Echolalia
DLD	1	Echolalia
DLG1	1	Echolalia
DLG2	1	Echolalia
DLG3	1	Echolalia
DLG4	1	Echolalia
DLGAP1	1	Echolalia
DLGAP3	1	Echolalia
DLGAP4	1	Echolalia
DLGAP5	1	Echolalia
DLK1	1	Unresponsiveness to spoken voice
DLK2	1	Echolalia
DLL3	1	Echolalia
DLL4	1	Echolalia
DLST	1	Echolalia
DLX2	1	Echolalia
DLX3	1	Echolalia
DMAC1	1	Echolalia
DMAC2L	1	Echolalia
DMBX1	1	Echolalia
DMGDH	1	Echolalia
DMKN	1	Echolalia
DMP1	1	Echolalia
DMRT1	1	Echolalia
DMRT3	1	Echolalia
DMRTA1	1	Echolalia
DMTN	1	Echolalia
DNA2	1	Echolalia
DNAAF2	1	Echolalia

DNAAF3	1	Echolalia
DNAAF4	1	Motor delay
DNAH1	1	Echolalia
DNAH12	1	Echolalia
DNAH17	1	Echolalia
DNAH2	1	Echolalia
DNAH3	1	Echolalia
DNAH6	1	Echolalia
DNAH8	1	Echolalia
DNAH9	1	Echolalia
DNAI1	1	Echolalia
DNAI3	1	Echolalia
DNAI4	1	Echolalia
DNAJA4	1	Echolalia
DNAJB13	1	Echolalia
DNAJC11	1	Echolalia
DNAJC13	1	Echolalia
DNAJC2	1	Echolalia
DNAJC27	1	Echolalia
DNAJC9	1	Echolalia
DNASE1L3	1	Echolalia
DNASE2	1	Echolalia
DNER	1	Echolalia
DNHD1	1	Echolalia
DNLZ	1	Motor delay
DNM1L	1	Motor delay
DNM3	1	Echolalia
DNMT3A	1	Echolalia
DNMT3B	1	Echolalia
DNPH1	1	Unresponsiveness to spoken voice
DNTTIP2	1	Echolalia
DOC2A	1	Echolalia
DOCK11	1	Echolalia
DOCK2	1	Echolalia
DOCK3	1	Echolalia
DOCK4	1	Echolalia
DOCK7	1	Echolalia
DOCK8	1	Echolalia
DOCK8-AS1	1	Echolalia
DOCK9	1	Echolalia
DOK2	1	Echolalia
DOLPP1	1	Echolalia
DOT1L	1	Echolalia
DPEP1	1	Echolalia
DPEP3	1	Echolalia
DPF2	1	Echolalia

DPF3	1	Echolalia
DPH1	1	Echolalia
DPM1	1	Mutism
DPM2	1	Echolalia
DPP4	1	Echolalia
DPP6	1	Echolalia
DPP8	1	Echolalia
DPP9	1	Echolalia
DPT	1	Echolalia
DPY19L2	1	Echolalia
DPYD	1	Echolalia
DPYSL2	1	Echolalia
DPYSL3	1	Echolalia
DPYSL4	1	Echolalia
DRC7	1	Echolalia
DRD1	1	Echolalia
DROSHA	1	Echolalia
DSC1	1	Echolalia
DSC2	1	Echolalia
DSC3	1	Echolalia
DSCAM	1	Echolalia
DSCAML1	1	Echolalia
DSE	1	Echolalia
DSG1	1	Echolalia
DSG2	1	Echolalia
DSG2-AS1	1	Mutism
DSP	1	Echolalia
DSPP	1	Echolalia
DSTYK	1	Echolalia
DTHD1	1	Echolalia
DTX1	1	Echolalia
DTX2	1	Echolalia
DTYMK	1	Echolalia
DUOX1	1	Echolalia
DUOX2	1	Echolalia
DUOXA1	1	Echolalia
DUS1L	1	Echolalia
DUS2	1	Echolalia
DUS3L	1	Echolalia
DUS4L	1	Echolalia
DUS4L-BCAP29	1	Echolalia
DUSP1	1	Echolalia
DUSP10	1	Echolalia
DUSP12	1	Echolalia
DUSP13	1	Echolalia
DUSP15	1	Echolalia

DUSP26	1	Unresponsiveness to spoken voice
DUSP29	1	Echolalia
DUSP4	1	Language disorder
DUSP5	1	Echolalia
DUSP6	1	Echolalia
DUXAP10	1	Echolalia
DUXAP8	1	Echolalia
DVL3	1	Echolalia
DYDC2	1	Echolalia
DYNAP	1	Echolalia
DYNC1H1	1	Echolalia
DYNC1I1	1	Echolalia
DYNC2H1	1	Echolalia
DYNC2I2	1	Echolalia
DYNLRB1	1	Echolalia
DYRK1A	1	Echolalia
DYSF	1	Echolalia
DZANK1	1	Echolalia
E2F1	1	Echolalia
E2F3	1	Echolalia
EAF1	1	Echolalia
EAPP	1	Echolalia
EARS2	1	Echolalia
EBF1	1	Echolalia
EBF3	1	Echolalia
EBI3	1	Echolalia
EBLN2	1	Echolalia
EBNA1BP2	1	Echolalia
ECD	1	Echolalia
ECH1	1	Echolalia
ECHDC2	1	Echolalia
ECHDC3	1	Echolalia
ECHS1	1	Echolalia
ECMXP	1	Echolalia
ECRG4	1	Echolalia
ECT2	1	Echolalia
EDA2R	1	Anxiety
EDDM3A	1	Echolalia
EDEM2	1	Echolalia
EDEM3	1	Echolalia
EDIL3	1	Echolalia
EDNRA	1	Echolalia
EDNRB-AS1	1	Echolalia
EEA1	1	Echolalia
EED	1	Echolalia
EEF1A1	1	Echolalia

EEF1AKMT1	1	Echolalia
EEF1G	1	Language disorder
EEF2	1	Echolalia
EFCAB12	1	Echolalia
EFCAB13	1	Echolalia
EFCAB2	1	Echolalia
EFCAB3	1	Echolalia
EFCAB7	1	Echolalia
EFCAB9	1	Echolalia
EFCC1	1	Echolalia
EFEMP2	1	Echolalia
EFHC1	1	Echolalia
EFHD1	1	Echolalia
EFL1	1	Echolalia
EFNB1	1	Echolalia
EFR3A	1	Echolalia
EFR3B	1	Echolalia
EFS	1	Echolalia
EGF	1	Echolalia
EGFLAM	1	Echolalia
EGLN2	1	Echolalia
EHBP1	1	Echolalia
EHD4	1	Echolalia
EHF	1	Echolalia
EID2B	1	Echolalia
EIF2AK3	1	Echolalia
EIF2AK4	1	Echolalia
EIF2B5	1	Echolalia
EIF2S3	1	Echolalia
EIF3A	1	Echolalia
EIF3C	1	Echolalia
EIF3D	1	Echolalia
EIF3H	1	Echolalia
EIF3L	1	Echolalia
EIF4B	1	Echolalia
EIF4E2	1	Echolalia
EIF4ENIF1	1	Echolalia
EIF4G1	1	Echolalia
EIF5A	1	Echolalia
EIF5B	1	Echolalia
EIPR1	1	Echolalia
ELAPOR1	1	Echolalia
ELAPOR2	1	Echolalia
ELAVL1	1	Echolalia
ELF1	1	Echolalia
ELF2	1	Language disorder

ELF5	1	Echolalia
ELK1	1	Motor delay
ELL	1	Echolalia
ELL2	1	Echolalia
ELMO1	1	Echolalia
ELMOD3	1	Echolalia
ELN	1	Echolalia
ELOA	1	Echolalia
ELOVL2	1	Echolalia
ELOVL5	1	Echolalia
ELP1	1	Echolalia
ELP2	1	Echolalia
ELP4	1	Echolalia
ELSPBP1	1	Echolalia
EMC10	1	Echolalia
EMILIN3	1	Echolalia
EML3	1	Echolalia
EML6	1	Echolalia
EMP1	1	Echolalia
EMX2	1	Echolalia
EN1	1	Echolalia
ENAH	1	Echolalia
ENAM	1	Echolalia
ENDOD1	1	Echolalia
ENDOV	1	Echolalia
ENG	1	Echolalia
ENGASE	1	Echolalia
ENKD1	1	Echolalia
ENKUR	1	Unresponsiveness to spoken voice
ENOSF1	1	Echolalia
ENOX1	1	Echolalia
ENPEP	1	Echolalia
ENPP1	1	Echolalia
ENPP2	1	Echolalia
ENPP5	1	Echolalia
ENPP7	1	Echolalia
ENTPD1	1	Echolalia
ENTPD1-AS1	1	Echolalia
ENTPD3	1	Echolalia
ENTPD4	1	Echolalia
ENTPD7	1	Echolalia
ENTPD8	1	Echolalia
ENTR1	1	Echolalia
EOMES	1	Mutism
EP300	1	Motor delay
EP400	1	Echolalia

EPB41	1	Echolalia
EPB41L1	1	Echolalia
EPB41L3	1	Echolalia
EPB41L5	1	Echolalia
EPC1	1	Echolalia
EPC2	1	Echolalia
EPG5	1	Echolalia
EPHA10	1	Echolalia
EPHA3	1	Echolalia
EPHA5	1	Echolalia
EPHA6	1	Echolalia
EPHA7	1	Echolalia
EPHA8	1	Echolalia
EPHB2	1	Echolalia
EPHB3	1	Echolalia
EPHB4	1	Echolalia
EPHX1	1	Echolalia
EPHX2	1	Echolalia
EPM2A	1	Echolalia
EPN1	1	Echolalia
EPS15	1	Echolalia
EPS8	1	Echolalia
EPS8L2	1	Echolalia
EPX	1	Echolalia
EPYC	1	Echolalia
ERAL1	1	Echolalia
ERAP1	1	Echolalia
ERAP2	1	Echolalia
ERBB3	1	Echolalia
ERBIN	1	Echolalia
ERCC2	1	Echolalia
ERCC3	1	Echolalia
ERCC6L2	1	Echolalia
ERCC8	1	Echolalia
ERFE	1	Echolalia
ERGIC1	1	Echolalia
ERGIC3	1	Echolalia
ERI2	1	Echolalia
ERICH6	1	Echolalia
ERICH6B	1	Echolalia
ERLIN2	1	Anxiety
ERMAP	1	Echolalia
1,00 ERN	1	Echolalia
2,00 ERN	1	Echolalia
ERRFI1	1	Echolalia
ERVFRD-1	1	Echolalia

ERVW-1	1	Echolalia
ESCO1	1	Echolalia
ESCO2	1	Echolalia
ESD	1	Echolalia
ESF1	1	Echolalia
ESPNL	1	Echolalia
ESRRA	1	Echolalia
ESRRB	1	Echolalia
ESRRG	1	Anxiety
ESYT2	1	Echolalia
ETAA1	1	Echolalia
ETNK2	1	Echolalia
ETS2	1	Echolalia
ETV2	1	Echolalia
ETV3	1	Echolalia
ETV3L	1	Echolalia
ETV7	1	Echolalia
EVA1A	1	Echolalia
EVC2	1	Echolalia
EVI5L	1	Echolalia
EVL	1	Echolalia
EVPL	1	Echolalia
EVX2	1	Echolalia
EXD3	1	Echolalia
EXO5	1	Echolalia
EXOC3	1	Echolalia
EXOC3L1	1	Echolalia
EXOC3L4	1	Echolalia
EXOC4	1	Echolalia
EXOC5	1	Echolalia
EXOC6	1	Echolalia
EXOC6B	1	Echolalia
EXOSC10	1	Echolalia
EXOSC3	1	Echolalia
EXOSC5	1	Echolalia
EXOSC7	1	Echolalia
EXOSC8	1	Echolalia
EXOSC9	1	Echolalia
EXPH5	1	Echolalia
EXT2	1	Unresponsiveness to spoken voice
EXTL1	1	Echolalia
EYA1	1	Echolalia
EYA2	1	Echolalia
EYS	1	Echolalia
F10	1	Echolalia
F12	1	Echolalia

F13A1	1	Echolalia
F8	1	Echolalia
FA2H	1	Echolalia
FAAH	1	Echolalia
FAAH2	1	Unresponsiveness to spoken voice
FAAP100	1	Echolalia
FAAP20	1	Echolalia
FAAP24	1	Echolalia
FABP2	1	Echolalia
FABP9	1	Motor delay
FADS2	1	Echolalia
FADS6	1	Echolalia
FAH	1	Echolalia
FAHD1	1	Echolalia
FAIM	1	Echolalia
FAM110A	1	Echolalia
FAM111A	1	Echolalia
FAM111B	1	Echolalia
FAM114A2	1	Echolalia
FAM117A	1	Echolalia
FAM118A	1	Echolalia
FAM118B	1	Echolalia
FAM120A	1	Echolalia
FAM124A	1	Echolalia
FAM131A	1	Echolalia
FAM131B	1	Echolalia
FAM131C	1	Echolalia
FAM133B	1	Echolalia
FAM135A	1	Echolalia
FAM136A	1	Echolalia
FAM13B	1	Echolalia
FAM149A	1	Echolalia
FAM149B1	1	Echolalia
FAM151A	1	Echolalia
FAM153A	1	Echolalia
FAM153B	1	Echolalia
FAM160A1	1	Echolalia
FAM160A2	1	Echolalia
FAM166A	1	Echolalia
FAM166C	1	Echolalia
FAM167A	1	Echolalia
FAM169B	1	Unresponsiveness to spoken voice
FAM171B	1	Echolalia
FAM174C	1	Echolalia
FAM177B	1	Echolalia
FAM181B	1	Echolalia

FAM182B	1	Echolalia
FAM184A	1	Echolalia
FAM185A	1	Echolalia
FAM186B	1	Echolalia
FAM187B	1	Echolalia
FAM200A	1	Language disorder
FAM205A	1	Echolalia
FAM207A	1	Echolalia
FAM20C	1	Echolalia
FAM210A	1	Echolalia
FAM210B	1	Echolalia
FAM214B	1	Echolalia
FAM216A	1	Echolalia
FAM218A	1	Echolalia
FAM221A	1	Echolalia
FAM221B	1	Echolalia
FAM222A	1	Echolalia
FAM227B	1	Echolalia
FAM228B	1	Echolalia
FAM230G	1	Echolalia
FAM230H	1	Echolalia
FAM234B	1	Echolalia
FAM241B	1	Echolalia
FAM25A	1	Echolalia
FAM25C	1	Echolalia
FAM25G	1	Echolalia
FAM32A	1	Echolalia
FAM3B	1	Echolalia
FAM3D	1	Echolalia
FAM47E	1	Echolalia
FAM71B	1	Echolalia
FAM71E1	1	Echolalia
FAM71F1	1	Echolalia
FAM72A	1	Echolalia
FAM78A	1	Echolalia
FAM83D	1	Unresponsiveness to spoken voice
FAM83E	1	Echolalia
FAM86B1	1	Echolalia
FAM90A1	1	Echolalia
FAM91A1	1	Echolalia
FAM95B1	1	Echolalia
FAM9C	1	Echolalia
FANCB	1	Echolalia
FANCD2	1	Echolalia
FANCE	1	Echolalia
FANCG	1	Unresponsiveness to spoken voice

FANCI	1	Echolalia
FANCL	1	Echolalia
FAP	1	Echolalia
FAR2	1	Echolalia
FARP1	1	Echolalia
FASN	1	Echolalia
FASTKD1	1	Echolalia
FASTKD2	1	Echolalia
FASTKD3	1	Echolalia
FAT1	1	Echolalia
FAT2	1	Echolalia
FAT3	1	Echolalia
FAT4	1	Echolalia
FBF1	1	Echolalia
FBH1	1	Echolalia
FBLIM1	1	Echolalia
FBLL1	1	Echolalia
FBLN2	1	Echolalia
FBN1	1	Echolalia
FBN2	1	Echolalia
FBP2	1	Echolalia
FBRSL1	1	Echolalia
FBXL13	1	Echolalia
FBXL16	1	Echolalia
FBXL18	1	Echolalia
FBXL19	1	Echolalia
FBXL21P	1	Echolalia
FBXL8	1	Aggressive behaviour
FBXO16	1	Echolalia
FBXO17	1	Echolalia
FBXO24	1	Echolalia
FBXO25	1	Echolalia
FBXO28	1	Anxiety
FBXO31	1	Sleep disturbances
FBXO33	1	Echolalia
FBXO34	1	Echolalia
FBXO38	1	Echolalia
FBXO39	1	Echolalia
FBXO40	1	Echolalia
FBXO41	1	Mutism
FBXO44	1	Echolalia
FBXO5	1	Echolalia
FBXW11	1	Unresponsiveness to spoken voice
FBXW4	1	Echolalia
FCAMR	1	Echolalia
FCER2	1	Echolalia

FCGR1B	1	Echolalia
FCGR2A	1	Echolalia
FCHO2	1	Echolalia
FCHSD1	1	Echolalia
FCMR	1	Echolalia
FCN1	1	Echolalia
FCN2	1	Echolalia
FCRL4	1	Echolalia
FCRLB	1	Echolalia
FDXACB1	1	Echolalia
FDXR	1	Echolalia
FECH	1	Echolalia
FEM1B	1	Echolalia
FEM1C	1	Echolalia
FER1L6	1	Echolalia
FERMT2	1	Echolalia
FERMT3	1	Echolalia
FES	1	Echolalia
FETUB	1	Echolalia
FEZ1	1	Echolalia
FFAR2	1	Echolalia
FFAR3	1	Echolalia
FGD1	1	Anxiety
FGD2	1	Echolalia
FGD3	1	Echolalia
FGD5	1	Echolalia
FGF11	1	Anxiety
FGF13	1	Echolalia
FGF20	1	Echolalia
FGF21	1	Echolalia
FGF6	1	Echolalia
FGFR2	1	Echolalia
FGFR3	1	Echolalia
FGFR4	1	Echolalia
FGG	1	Echolalia
FGGY	1	Echolalia
FGL1	1	Echolalia
FHAD1	1	Echolalia
FHIT	1	Echolalia
FHL5	1	Echolalia
FHOD3	1	Echolalia
FIG4	1	Echolalia
FIGLA	1	Motor delay
FIGNL1	1	Echolalia
FIGNL2	1	Echolalia
FILIP1	1	Echolalia

FITM1	1	Motor delay
FKBP10	1	Echolalia
FKBP5	1	Echolalia
FKBP6	1	Echolalia
FLACC1	1	Echolalia
FLG2	1	Echolalia
FLI1	1	Echolalia
FLJ44635	1	Echolalia
FLNA	1	Echolalia
FLNB	1	Echolalia
FLRT2	1	Echolalia
FLRT3	1	Echolalia
FLT1	1	Echolalia
FLT3	1	Echolalia
FLT3LG	1	Echolalia
FLT4	1	Echolalia
FLVCR1	1	Echolalia
FLVCR2	1	Echolalia
FLYWCH1	1	Echolalia
FMN1	1	Echolalia
FMN2	1	Echolalia
FMNL1	1	Echolalia
FMNL3	1	Echolalia
FMO1	1	Echolalia
FMO2	1	Echolalia
FMO3	1	Echolalia
FMO5	1	Echolalia
FMOD	1	Echolalia
FNBP1	1	Echolalia
FNDC1	1	Echolalia
FNDC11	1	Aggressive behaviour
FNDC3A	1	Echolalia
FNDC3B	1	Echolalia
FNDC7	1	Echolalia
FNIP2	1	Echolalia
FOLH1	1	Echolalia
FOLH1B	1	Echolalia
FOXA1	1	Language disorder
FOXA3	1	Echolalia
FOXC2	1	Echolalia
FOXD2	1	Echolalia
FOXD4	1	Echolalia
FOXD4L3	1	Echolalia
FOXD4L5	1	Echolalia
FOXD4L6	1	Echolalia
FOXE1	1	Echolalia

FOXE3	1	Echolalia
FOXJ3	1	Echolalia
FOXK1	1	Echolalia
FOXK2	1	Echolalia
FOXL3	1	Echolalia
FOXO3	1	Echolalia
FOXO6	1	Echolalia
FOXP4	1	Echolalia
FOXRED1	1	Motor delay
FPGS	1	Echolalia
FPR1	1	Echolalia
FREM2	1	Echolalia
FRG2B	1	Echolalia
FRG2C	1	Echolalia
FRK	1	Echolalia
FRMD3	1	Echolalia
FRMD4A	1	Echolalia
FRMD4B	1	Echolalia
FRMD6	1	Echolalia
FRMD7	1	Echolalia
FRMD8	1	Echolalia
FRMPD3	1	Echolalia
FRRS1	1	Echolalia
FRY	1	Echolalia
FSCN1	1	Echolalia
FSCN3	1	Echolalia
FSD1L	1	Echolalia
FSHB	1	Echolalia
FSHR	1	Echolalia
FSIP1	1	Echolalia
FTCD	1	Echolalia
FTL	1	Echolalia
FTSJ3	1	Echolalia
FUCA1	1	Echolalia
FUCA2	1	Echolalia
FURIN	1	Echolalia
FUT10	1	Echolalia
FUT3	1	Echolalia
FUT4	1	Echolalia
FUT8	1	Echolalia
FXYD3	1	Echolalia
FXYD5	1	Echolalia
FXYD7	1	Echolalia
FYCO1	1	Echolalia
FYN	1	Echolalia
FYTTD1	1	Echolalia

FZD10	1	Echolalia
G6PD	1	Echolalia
GAB2	1	Echolalia
GAB4	1	Echolalia
GABBR2	1	Echolalia
GABPB2	1	Echolalia
GABRA2	1	Echolalia
GABRA6	1	Echolalia
GABRB1	1	Echolalia
GABRB2	1	Echolalia
GABRD	1	Echolalia
GABRQ	1	Aggressive behaviour
GABRR3	1	Echolalia
GADL1	1	Echolalia
GAGE12C	1	Echolalia
GAGE12D	1	Echolalia
GAGE12E	1	Echolalia
GAGE12F	1	Echolalia
GAGE12G	1	Echolalia
GAGE12J	1	Echolalia
GAK	1	Echolalia
GAL	1	Echolalia
GAL3ST2	1	Language disorder
GALK2	1	Echolalia
GALM	1	Echolalia
GALNS	1	Echolalia
GALNT10	1	Aggressive behaviour
GALNT11	1	Echolalia
GALNT12	1	Echolalia
GALNT16	1	Echolalia
GALNT18	1	Echolalia
GALNT4	1	Echolalia
GALNT5	1	Echolalia
GALNT6	1	Echolalia
GALNT9	1	Echolalia
GALNTL5	1	Echolalia
GALP	1	Echolalia
GALR1	1	Echolalia
GALR2	1	Echolalia
GALT	1	Echolalia
GAMT	1	Language disorder
GAN	1	Echolalia
GANC	1	Echolalia
GAP43	1	Language disorder
GAPDH	1	Echolalia
GAPDHS	1	Echolalia

GAPVD1	1	Echolalia
GAREM1	1	Echolalia
GARRE1	1	Language disorder
GARS1	1	Echolalia
GAS2L1	1	Echolalia
GAS2L2	1	Echolalia
GAS6	1	Echolalia
GAS7	1	Echolalia
GATA2	1	Echolalia
GATA3	1	Echolalia
GATA4	1	Echolalia
GATA6	1	Echolalia
GATAD1	1	Echolalia
GATB	1	Echolalia
GATC	1	Echolalia
GATD1	1	Echolalia
GBA3	1	Echolalia
GBE1	1	Echolalia
GBP7	1	Echolalia
GBX2	1	Echolalia
GCAT	1	Echolalia
GCC1	1	Echolalia
GCDH	1	Echolalia
GCH1	1	Echolalia
GCKR	1	Echolalia
GCLC	1	Anxiety
GCM1	1	Echolalia
GCN1	1	Echolalia
GCNT1	1	Echolalia
GCNT2	1	Echolalia
GCNT3	1	Echolalia
GCNT4	1	Unresponsiveness to spoken voice
GCOM1	1	Echolalia
GDA	1	Echolalia
GDF2	1	Sleep disturbances
GDF5	1	Echolalia
GDF7	1	Echolalia
GDNF	1	Echolalia
GDNF-AS1	1	Unresponsiveness to spoken voice
GDPD4	1	Echolalia
GDPD5	1	Echolalia
GEMIN4	1	Echolalia
GEMIN5	1	Echolalia
GET1-SH3BGR	1	Echolalia
GET4	1	Echolalia
GFER	1	Echolalia

GFM1	1	Echolalia
GFRA1	1	Echolalia
GFRA2	1	Echolalia
GFRAL	1	Echolalia
GFY	1	Echolalia
GGCX	1	Echolalia
GGN	1	Echolalia
GGT1	1	Echolalia
GGTLC2	1	Echolalia
GHR	1	Echolalia
GHRL	1	Echolalia
GHSR	1	Echolalia
GIMAP5	1	Echolalia
GIMAP6	1	Echolalia
GIMAP7	1	Echolalia
GIMD1	1	Echolalia
GIN1	1	Echolalia
GINM1	1	Echolalia
GIPC2	1	Echolalia
GIPR	1	Echolalia
GIT2	1	Echolalia
GJA4	1	Echolalia
GJB2	1	Echolalia
GJB3	1	Echolalia
GJB7	1	Echolalia
GJD2	1	Echolalia
GJD4	1	Echolalia
GK2	1	Echolalia
GK3P	1	Echolalia
GKN1	1	Echolalia
GLB1	1	Echolalia
GLB1L2	1	Echolalia
GLCCI1	1	Echolalia
GLCE	1	Echolalia
GLDC	1	Echolalia
GLDN	1	Echolalia
GLI1	1	Echolalia
GLI2	1	Echolalia
GLI3	1	Echolalia
GLIPR2	1	Anxiety
GLIS1	1	Echolalia
GLMP	1	Echolalia
GLP1R	1	Echolalia
GLP2R	1	Echolalia
GLRA3	1	Echolalia
GLRA4	1	Unresponsiveness to spoken voice

GLRX2	1	Echolalia
GLT1D1	1	Echolalia
GLT6D1	1	Echolalia
GLT8D2	1	Echolalia
GLTPD2	1	Echolalia
GLUD2	1	Echolalia
GLYAT	1	Echolalia
GLYATL2	1	Echolalia
GLYATL3	1	Echolalia
GM2A	1	Echolalia
GMCL2	1	Echolalia
GMDS	1	Motor delay
GMEB2	1	Echolalia
GMIP	1	Unresponsiveness to spoken voice
GMNN	1	Echolalia
GMPS	1	Anxiety
GNA12	1	Echolalia
GNA14	1	Echolalia
GNA15	1	Echolalia
GNAI3	1	Echolalia
GNAO1	1	Echolalia
GNB4	1	Echolalia
GNG4	1	Unresponsiveness to spoken voice
GNG8	1	Echolalia
GNGT1	1	Echolalia
GNL2	1	Echolalia
GNLY	1	Echolalia
GNPTAB	1	Echolalia
GNRHR	1	Echolalia
GNS	1	Echolalia
GOLGA1	1	Echolalia
GOLGA2	1	Echolalia
GOLGA2P11	1	Echolalia
GOLGA3	1	Echolalia
GOLGA4	1	Echolalia
GOLGA6A	1	Echolalia
GOLGA6B	1	Echolalia
GOLGA6L10	1	Echolalia
GOLGA6L2	1	Echolalia
GOLGA6L3	1	Echolalia
GOLGA6L6	1	Echolalia
GOLGA6L7	1	Echolalia
GOLGA8H	1	Echolalia
GOLGA8N	1	Echolalia
GOLGA8Q	1	Echolalia
GOLGA8S	1	Echolalia

GOLGA8T	1	Echolalia
GOLM1	1	Echolalia
GOLM2	1	Echolalia
GOLT1A	1	Echolalia
GORASP2	1	Echolalia
GOSR2	1	Echolalia
GOT1L1	1	Echolalia
GP2	1	Echolalia
GPA33	1	Echolalia
GPAA1	1	Echolalia
GPAM	1	Echolalia
GPAT2	1	Echolalia
GPAT4	1	Anxiety
GPATCH2	1	Echolalia
GPATCH3	1	Language disorder
GPATCH4	1	Anxiety
GPATCH8	1	Echolalia
GPBP1L1	1	Echolalia
GPC5	1	Echolalia
GPCPD1	1	Echolalia
GPD2	1	Echolalia
GPER1	1	Echolalia
GPHN	1	Unresponsiveness to spoken voice
GPI	1	Echolalia
GPKOW	1	Unresponsiveness to spoken voice
GPLD1	1	Echolalia
GPN1	1	Motor delay
GPN2	1	Echolalia
GPR1	1	Echolalia
GPR101	1	Echolalia
GPR107	1	Language disorder
GPR137C	1	Echolalia
GPR141	1	Echolalia
GPR146	1	Echolalia
GPR150	1	Echolalia
GPR151	1	Echolalia
GPR157	1	Echolalia
GPR162	1	Echolalia
GPR17	1	Anxiety
GPR180	1	Echolalia
GPR182	1	Echolalia
GPR19	1	Echolalia
GPR25	1	Echolalia
GPR3	1	Echolalia
GPR33	1	Echolalia
GPR35	1	Echolalia

GPR37	1	Echolalia
GPR37L1	1	Echolalia
GPR4	1	Anxiety
GPR42	1	Echolalia
GPR50	1	Echolalia
GPR55	1	Echolalia
GPR65	1	Echolalia
GPR68	1	Echolalia
GPR75-ASB3	1	Echolalia
GPR87	1	Echolalia
GPR88	1	Mutism
GPRASP1	1	Echolalia
GPRC5A	1	Echolalia
GPRC5B	1	Echolalia
GPRC5D	1	Echolalia
GPRIN2	1	Echolalia
GPS1	1	Echolalia
GPS2	1	Echolalia
GPSM1	1	Echolalia
GPSM2	1	Echolalia
GPT2	1	Anxiety
GPX6	1	Unresponsiveness to spoken voice
GPX7	1	Echolalia
GRAMD4	1	Echolalia
GRB10	1	Echolalia
GRB14	1	Echolalia
GREB1L	1	Echolalia
GREM2	1	Echolalia
GRHL1	1	Echolalia
GRHPR	1	Echolalia
GRIA3	1	Echolalia
GRIA4	1	Echolalia
GRID2	1	Echolalia
GRIFIN	1	Echolalia
GRIK1	1	Echolalia
GRIK3	1	Echolalia
GRIN2A	1	Echolalia
GRIN2C	1	Echolalia
GRIN2D	1	Echolalia
GRIP2	1	Echolalia
GRIPAP1	1	Unresponsiveness to spoken voice
GRK3	1	Motor delay
GRK4	1	Echolalia
GRK7	1	Echolalia
GRM3	1	Echolalia
GRM4	1	Echolalia

GRM5	1	Echolalia
GRM6	1	Echolalia
GRM7	1	Echolalia
GRM7-AS3	1	Echolalia
GRPR	1	Echolalia
GRTP1	1	Echolalia
GSAP	1	Echolalia
GSDMC	1	Echolalia
GSG1L	1	Echolalia
GSG1L2	1	Echolalia
GSK3A	1	Echolalia
GSN	1	Echolalia
GSPT1	1	Echolalia
GSTM5	1	Echolalia
GSTO1	1	Echolalia
GSTO2	1	Echolalia
GSX1	1	Echolalia
GSX2	1	Anxiety
GTDC1	1	Echolalia
GTF2A1L	1	Echolalia
GTF2E1	1	Unresponsiveness to spoken voice
GTF2IRD1	1	Motor delay
GTF2IRD2	1	Echolalia
GTF2IRD2B	1	Echolalia
GTF3A	1	Echolalia
GTF3C2	1	Echolalia
GTF3C3	1	Echolalia
GTPBP10	1	Echolalia
GTPBP8	1	Echolalia
GTSE1	1	Echolalia
GUCA1C	1	Echolalia
GUCA2A	1	Echolalia
GUCA2B	1	Echolalia
GUCY1A1	1	Echolalia
GUCY1A2	1	Echolalia
GUCY2D	1	Echolalia
GUCY2F	1	Echolalia
GUF1	1	Echolalia
GUSB	1	Echolalia
GXYLT1	1	Echolalia
GYPA	1	Echolalia
GYPE	1	Echolalia
GYS1	1	Echolalia
GZF1	1	Echolalia
GZMH	1	Echolalia
GZMM	1	Echolalia

H1-1	1	Echolalia
H1-2	1	Echolalia
H1-5	1	Echolalia
H1-7	1	Unresponsiveness to spoken voice
H1-8	1	Echolalia
H2AC1	1	Echolalia
H2AC16	1	Echolalia
H2AC4	1	Echolalia
H2AC7	1	Echolalia
H2BC1	1	Echolalia
H2BC7	1	Echolalia
H2BC8	1	Echolalia
H2BP2	1	Echolalia
H3-3B	1	Echolalia
H3-5	1	Echolalia
H3C11	1	Echolalia
H4-16	1	Echolalia
H4C11	1	Echolalia
H4C12	1	Echolalia
H4C3	1	Echolalia
HAAO	1	Echolalia
HABP2	1	Echolalia
HACD1	1	Echolalia
HACD2	1	Echolalia
HACD3	1	Echolalia
HACL1	1	Echolalia
HAGH	1	Echolalia
HAGHL	1	Echolalia
HAL	1	Echolalia
HAND1	1	Echolalia
HAND2	1	Echolalia
HAO1	1	Echolalia
HAPLN1	1	Echolalia
HAPLN3	1	Echolalia
HAS1	1	Echolalia
HAS3	1	Echolalia
HAUS5	1	Echolalia
HAUS7	1	Echolalia
HAUS8	1	Echolalia
HBB	1	Echolalia
HBM	1	Echolalia
HCAR2	1	Echolalia
HCCAT5	1	Unresponsiveness to spoken voice
HCN1	1	Echolalia
HCN2	1	Echolalia
HCRTR1	1	Unresponsiveness to spoken voice

HCRTR2	1	Language disorder
HDAC4	1	Echolalia
HDAC4-AS1	1	Echolalia
HDAC7	1	Echolalia
HDAC9	1	Echolalia
HDGFL1	1	Echolalia
HDHD3	1	Echolalia
HDHD5	1	Echolalia
HEATR3	1	Echolalia
HEATR4	1	Echolalia
HEATR5A	1	Echolalia
HEATR6	1	Echolalia
HEBP2	1	Echolalia
HECTD1	1	Echolalia
HECTD4	1	Echolalia
HECW2	1	Echolalia
HEG1	1	Echolalia
HELB	1	Echolalia
HELLS	1	Echolalia
HELZ	1	Echolalia
HELZ2	1	Echolalia
HEMGN	1	Anxiety
HEPACAM	1	Echolalia
HEPACAM2	1	Echolalia
HEPN1	1	Echolalia
HERC1	1	Echolalia
HERC2P3	1	Echolalia
HERC4	1	Echolalia
HERC6	1	Echolalia
HERPUD1	1	Echolalia
HEXA	1	Echolalia
HEXB	1	Echolalia
HEXIM1	1	Echolalia
HEYL	1	Echolalia
HFE	1	Echolalia
HGC6.3	1	Echolalia
HGFAC	1	Echolalia
HGH1	1	Echolalia
HHAT	1	Echolalia
HHIP-AS1	1	Echolalia
HHIPL1	1	Echolalia
HHLA1	1	Echolalia
HHLA2	1	Echolalia
HHLA3	1	Echolalia
HIBCH	1	Echolalia
HIC1	1	Echolalia

HIC2	1	Motor delay
HID1	1	Echolalia
HIF1A	1	Echolalia
HINT1	1	Echolalia
HIP1	1	Echolalia
HIPK1	1	Echolalia
HIPK3	1	Anxiety
HIRA	1	Echolalia
HK3	1	Echolalia
HKDC1	1	Echolalia
HLA-C	1	Echolalia
HLA-DQA1	1	Echolalia
HLA-DQB1	1	Echolalia
HLA-DRB1	1	Echolalia
HLA-DRB1;HLA-DRB1	1	Echolalia
HLCS	1	Echolalia
HMCN1	1	Echolalia
HMCN2	1	Echolalia
HMGCR	1	Echolalia
HMGCS1	1	Echolalia
HMGCS2	1	Anxiety
HMX1	1	Echolalia
HNF1A	1	Echolalia
HNF4G	1	Echolalia
HNMT	1	Echolalia
HNRNPCL1	1	Echolalia
HNRNPCL2	1	Echolalia
HNRNPLL	1	Echolalia
HNRNPM	1	Echolalia
HNRNPU	1	Echolalia
HOMER2	1	Language disorder
HOPX	1	Echolalia
HORMAD2	1	Echolalia
HOXA1	1	Echolalia
HOXA13	1	Echolalia
HOXA3	1	Echolalia
HOXB-AS3	1	Echolalia
HOXB3	1	Mutism
HOXB7	1	Echolalia
HOXC10	1	Echolalia
HOXC4	1	Echolalia
HOXD12	1	Echolalia
HOXD13	1	Echolalia
HOXD3	1	Echolalia
HOXD9	1	Echolalia

HP	1	Language disorder
HPAT5	1	Echolalia
HPGD	1	Echolalia
HPN	1	Echolalia
HPS1	1	Echolalia
HPS3	1	Echolalia
HPS4	1	Echolalia
HPS5	1	Echolalia
HPS6	1	Echolalia
HPSE2	1	Echolalia
HR	1	Echolalia
HRG	1	Echolalia
HRH3	1	Echolalia
HRH4	1	Echolalia
HRNR	1	Echolalia
HS3ST1	1	Echolalia
HSD11B2	1	Echolalia
HSD17B13	1	Echolalia
HSD17B3	1	Echolalia
HSD17B4	1	Echolalia
HSD3B7	1	Echolalia
HSF4	1	Anxiety
HSF5	1	Echolalia
HSP90B1	1	Echolalia
HSPA12A	1	Echolalia
HSPA4	1	Echolalia
HSPB9	1	Echolalia
HSPBP1	1	Unresponsiveness to spoken voice
HSPD1	1	Echolalia
HSPE1-MOB4	1	Echolalia
HSPG2	1	Echolalia
HTATSF1	1	Echolalia
HTR1A	1	Echolalia
HTR1E	1	Echolalia
HTR2A	1	Echolalia
HTR3A	1	Echolalia
HTR3B	1	Echolalia
HTR3D	1	Echolalia
HTR3E	1	Echolalia
HTR5A	1	Echolalia
HTR6	1	Echolalia
HTR7	1	Echolalia
HTRA1	1	Echolalia
HTRA2	1	Echolalia
HTRA3	1	Echolalia
HTT	1	Echolalia

HUNK	1	Echolalia
HYAL1	1	Echolalia
HYAL2	1	Echolalia
HYAL3	1	Echolalia
HYDIN	1	Echolalia
HYOU1	1	Echolalia
HYPK	1	Echolalia
IARS2	1	Echolalia
IBSP	1	Echolalia
ICAM1	1	Echolalia
ICAM5	1	Echolalia
ICOSLG	1	Echolalia
IDH1	1	Echolalia
IDH3B	1	Echolalia
IDI1	1	Echolalia
IDI2	1	Echolalia
IDNK	1	Echolalia
IDO2	1	Echolalia
IDUA	1	Echolalia
IER2	1	Echolalia
IER5	1	Echolalia
IER5L	1	Echolalia
IFFO1	1	Echolalia
IFI16	1	Echolalia
IFI30	1	Echolalia
IFI44L	1	Echolalia
IFIT1	1	Echolalia
IFIT2	1	Echolalia
IFIT3	1	Echolalia
IFNA10	1	Echolalia
IFNA13	1	Echolalia
IFNA14	1	Echolalia
IFNA16	1	Echolalia
IFNA17	1	Echolalia
IFNA4	1	Echolalia
IFNA5	1	Echolalia
IFNA6	1	Echolalia
IFNAR1	1	Echolalia
IFNAR2	1	Echolalia
IFNL3	1	Echolalia
IFT122	1	Echolalia
IFT140	1	Echolalia
IFT172	1	Echolalia
IFT88	1	Echolalia
IGF1	1	Motor delay
IGF2BP2	1	Echolalia

IGF2R	1	Echolalia
IGFALS	1	Echolalia
IGFBP2	1	Echolalia
IGFBP3	1	Echolalia
IGFBP5	1	Echolalia
IGFL1	1	Echolalia
IGFL4	1	Echolalia
IGHMBP2	1	Echolalia
IGLL5	1	Echolalia
IGSF21	1	Anxiety
IGSF22	1	Echolalia
IGSF23	1	Echolalia
IGSF5	1	Echolalia
IGSF8	1	Echolalia
IGSF9	1	Echolalia
IGSF9B	1	Echolalia
IHO1	1	Echolalia
IKBIP	1	Echolalia
IKBKE	1	Echolalia
IKZF2	1	Echolalia
IKZF3	1	Echolalia
IL10RA	1	Echolalia
IL10RB	1	Echolalia
IL11	1	Echolalia
IL11RA	1	Echolalia
IL12A-AS1	1	Echolalia
IL12B	1	Echolalia
IL12RB1	1	Echolalia
IL13	1	Echolalia
IL16	1	Echolalia
IL17B	1	Anxiety
IL17F	1	Echolalia
IL17RA	1	Echolalia
IL17RC	1	Echolalia
IL17RD	1	Echolalia
IL17REL	1	Echolalia
IL18	1	Echolalia
IL19	1	Echolalia
IL1B	1	Echolalia
IL1F10	1	Echolalia
IL1R1	1	Echolalia
IL1R2	1	Echolalia
IL1RAP	1	Echolalia
IL1RL2	1	Echolalia
IL20RA	1	Echolalia
IL21	1	Echolalia

IL24	1	Aggressive behaviour
IL27	1	Echolalia
IL2RA	1	Echolalia
IL2RB	1	Echolalia
IL31RA	1	Echolalia
IL34	1	Echolalia
IL37	1	Echolalia
IL4R	1	Echolalia
IL6	1	Echolalia
IL6R	1	Echolalia
IL6ST	1	Echolalia
ILF3	1	Echolalia
ILK	1	Echolalia
IMPA2	1	Echolalia
IMPACT	1	Echolalia
IMPG1	1	Echolalia
IMPG2	1	Echolalia
INA	1	Echolalia
INAVA	1	Echolalia
INCENP	1	Echolalia
INF2	1	Echolalia
ING1	1	Echolalia
ING2	1	Echolalia
INHBB	1	Echolalia
INKA1	1	Echolalia
INO80	1	Echolalia
INO80C	1	Echolalia
INPP1	1	Echolalia
INPP4B	1	Echolalia
INPP5B	1	Echolalia
INPP5D	1	Echolalia
INPP5F	1	Echolalia
INPP5J	1	Echolalia
INPP5K	1	Echolalia
INSC	1	Echolalia
INSIG2	1	Echolalia
INSL3	1	Anxiety
INSL4	1	Echolalia
INSL6	1	Echolalia
INSRR	1	Echolalia
INSYN2B	1	Echolalia
INTS10	1	Echolalia
INTS11	1	Echolalia
INTS14	1	Echolalia
INTS6	1	Echolalia
INTS8	1	Echolalia

INTU	1	Echolalia
INVS	1	Echolalia
IP6K2	1	Echolalia
IP6K3	1	Echolalia
IPCEF1	1	Echolalia
IPO11	1	Echolalia
IPO13	1	Echolalia
IPO4	1	Echolalia
IPO5	1	Echolalia
IPPK	1	Echolalia
IQCA1	1	Echolalia
IQCC	1	Echolalia
IQCG	1	Echolalia
IQCJ-SCHIP1	1	Echolalia
IRAG1	1	Echolalia
IRAG2	1	Echolalia
IRAK1	1	Echolalia
IRAK1BP1	1	Echolalia
IRAK3	1	Echolalia
IRAK4	1	Echolalia
IRF2BP1	1	Echolalia
IRF2BPL	1	Echolalia
IRF3	1	Echolalia
IRF6	1	Echolalia
IRF8	1	Echolalia
IRGC	1	Echolalia
IRGQ	1	Unresponsiveness to spoken voice
IRS1	1	Echolalia
IRS2	1	Echolalia
IRX1	1	Echolalia
IRX2	1	Echolalia
IRX3	1	Echolalia
IRX4	1	Echolalia
ISG20	1	Echolalia
ISL2	1	Echolalia
ISM1	1	Echolalia
ISOC1	1	Echolalia
ISOC2	1	Echolalia
ITGA1	1	Echolalia
ITGA10	1	Echolalia
ITGA2B	1	Unresponsiveness to spoken voice
ITGA3	1	Echolalia
ITGA4	1	Echolalia
ITGA5	1	Echolalia
ITGA9	1	Echolalia
ITGAX	1	Echolalia

ITGB1	1	Echolalia
ITGB3	1	Echolalia
ITGB3BP	1	Echolalia
ITGB4	1	Echolalia
ITGB5	1	Echolalia
ITGB6	1	Echolalia
ITIH3	1	Echolalia
ITIH4	1	Echolalia
ITIH5	1	Echolalia
ITIH6	1	Echolalia
ITM2B	1	Echolalia
ITPKB	1	Echolalia
ITPKC	1	Echolalia
ITPR2	1	Echolalia
ITPR3	1	Echolalia
ITPRID2	1	Echolalia
ITPRIP	1	Language disorder
ITPRIPL1	1	Echolalia
ITPRIPL2	1	Echolalia
ITSN1	1	Echolalia
IWS1	1	Echolalia
IYD	1	Echolalia
IZUMO1	1	Unresponsiveness to spoken voice
JAG1	1	Echolalia
JAG2	1	Echolalia
JAK1	1	Echolalia
JAK3	1	Echolalia
JAKMIP2	1	Echolalia
JAKMIP3	1	Echolalia
JAM2	1	Echolalia
JARID2	1	Echolalia
JAZF1	1	Echolalia
JDP2	1	Echolalia
JHY	1	Echolalia
JMJD1C	1	Echolalia
JMJD4	1	Echolalia
JMY	1	Echolalia
JOSD2	1	Echolalia
JPH1	1	Echolalia
JPH2	1	Echolalia
JPH4	1	Echolalia
JUP	1	Echolalia
KALRN	1	Echolalia
KANK4	1	Echolalia
KANSL1L	1	Echolalia
KAT2A	1	Language disorder

KAT6B	1	Echolalia
KAT7	1	Echolalia
KATNAL2	1	Echolalia
KATNB1	1	Echolalia
KAZALD1	1	Echolalia
KAZN	1	Echolalia
KBTBD12	1	Echolalia
KBTBD2	1	Echolalia
KBTBD6	1	Echolalia
KBTBD8	1	Echolalia
KCNA1	1	Echolalia
KCNA5	1	Echolalia
KCNAB1	1	Echolalia
KCNC1	1	Echolalia
KCNC2	1	Echolalia
KCNC3	1	Echolalia
KCND1	1	Echolalia
KCND3	1	Echolalia
KCNE2	1	Language disorder
KCNE3	1	Echolalia
KCNE4	1	Echolalia
KCNF1	1	Echolalia
KCNH1	1	Echolalia
KCNH3	1	Echolalia
KCNH4	1	Echolalia
KCNH7	1	Motor delay
KCNH8	1	Echolalia
KCNIP1	1	Echolalia
KCNIP4	1	Echolalia
KCNJ1	1	Echolalia
KCNJ18	1	Echolalia
KCNJ2	1	Echolalia
KCNJ3	1	Echolalia
KCNJ4	1	Echolalia
KCNJ6	1	Echolalia
KCNJ9	1	Echolalia
KCNK1	1	Echolalia
KCNK10	1	Echolalia
KCNK12	1	Echolalia
KCNK15	1	Echolalia
KCNK15-AS1	1	Echolalia
KCNK16	1	Anxiety
KCNK17	1	Motor delay
KCNK2	1	Echolalia
KCNK3	1	Echolalia
KCNK6	1	Echolalia

KCNMA1	1	Echolalia
KCNMB1	1	Echolalia
KCNMB2	1	Echolalia
KCNN1	1	Echolalia
KCNN2	1	Echolalia
KCNQ1	1	Echolalia
KCNQ2	1	Echolalia
KCNQ3	1	Echolalia
KCNQ4	1	Echolalia
KCNQ5	1	Echolalia
KCNT1	1	Echolalia
KCTD1	1	Echolalia
KCTD10	1	Echolalia
KCTD12	1	Echolalia
KCTD14	1	Echolalia
KCTD17	1	Echolalia
KCTD19	1	Echolalia
KCTD20	1	Echolalia
KCTD3	1	Echolalia
KDM4B	1	Echolalia
KDM4C	1	Echolalia
KDM5B	1	Echolalia
KDM6A	1	Motor delay
KDM6B	1	Echolalia
KDM8	1	Echolalia
KERA	1	Echolalia
KHK	1	Language disorder
KHNYN	1	Echolalia
KHSRP	1	Anxiety
KIAA0232	1	Unresponsiveness to spoken voice
KIAA0754	1	Motor delay
KIAA0930	1	Echolalia
KIAA1109	1	Echolalia
KIAA1328	1	Echolalia
KIAA1522	1	Echolalia
KIAA1549L	1	Echolalia
KIAA1586	1	Echolalia
KIAA1614	1	Echolalia
KIAA1656	1	Echolalia
KIAA1671	1	Echolalia
KIAA1755	1	Echolalia
KIAA2012	1	Echolalia
KIAA2026	1	Echolalia
KIDINS220	1	Echolalia
KIF12	1	Echolalia
KIF13A	1	Echolalia

KIF14	1	Echolalia
KIF16B	1	Echolalia
KIF18B	1	Echolalia
KIF1B	1	Echolalia
KIF23	1	Echolalia
KIF24	1	Echolalia
KIF27	1	Echolalia
KIF2B	1	Echolalia
KIF2C	1	Echolalia
KIF3C	1	Echolalia
KIF5A	1	Echolalia
KIF6	1	Echolalia
KIF7	1	Echolalia
KIF9	1	Echolalia
KIFAP3	1	Echolalia
KIFBP	1	Echolalia
KIFC2	1	Echolalia
KIFC3	1	Echolalia
KIRREL1	1	Echolalia
KISS1	1	Echolalia
KISS1R	1	Echolalia
KIT	1	Echolalia
KITLG	1	Echolalia
KIZ	1	Echolalia
KL	1	Echolalia
KLB	1	Echolalia
KLC2	1	Echolalia
KLF1	1	Echolalia
KLF10	1	Echolalia
KLF14	1	Echolalia
KLF4	1	Echolalia
KLF6	1	Echolalia
KLF7	1	Motor delay
KLHDC3	1	Echolalia
KLHDC4	1	Echolalia
KLHDC8A	1	Echolalia
KLHL1	1	Echolalia
KLHL17	1	Echolalia
KLHL2	1	Echolalia
KLHL21	1	Echolalia
KLHL22	1	Echolalia
KLHL25	1	Echolalia
KLHL3	1	Echolalia
KLHL31	1	Echolalia
KLHL32	1	Echolalia
KLHL33	1	Echolalia

KLHL35	1	Echolalia
KLHL36	1	Echolalia
KLHL38	1	Echolalia
KLHL5	1	Echolalia
KLHL6	1	Echolalia
KLHL7	1	Language disorder
KLHL9	1	Mutism
KLK11	1	Echolalia
KLK12	1	Echolalia
KLK14	1	Anxiety
KLK15	1	Echolalia
KLK3	1	Echolalia
KLK6	1	Echolalia
KLK8	1	Echolalia
KLLN	1	Motor delay
KLRB1	1	Anxiety
KLRC3	1	Echolalia
KLRC4	1	Echolalia
KLRF2	1	Echolalia
KLRG1	1	Echolalia
KLRG2	1	Echolalia
KMO	1	Echolalia
KMT2C	1	Echolalia
KMT2E	1	Echolalia
KMT5C	1	Echolalia
KNDC1	1	Echolalia
KNG1	1	Echolalia
KNTC1	1	Echolalia
KPNA2	1	Echolalia
KPNA7	1	Echolalia
KPTN	1	Echolalia
KRBA1	1	Echolalia
KRCC1	1	Echolalia
KREMEN1	1	Echolalia
KREMEN2	1	Echolalia
KRR1	1	Echolalia
KRT13	1	Motor delay
KRT15	1	Echolalia
KRT16	1	Echolalia
KRT17	1	Anxiety
KRT19	1	Echolalia
KRT2	1	Echolalia
KRT222	1	Echolalia
KRT25	1	Echolalia
KRT32	1	Echolalia
KRT35	1	Echolalia

KRT38	1	Echolalia
KRT72	1	Echolalia
KRT73	1	Echolalia
KRT74	1	Echolalia
KRT75	1	Echolalia
KRT78	1	Echolalia
KRT79	1	Echolalia
KRT8	1	Echolalia
KRT80	1	Echolalia
KRT84	1	Echolalia
KRT85	1	Echolalia
KRT86	1	Echolalia
KRT9	1	Echolalia
KRTAP10-1	1	Echolalia
KRTAP10-10	1	Echolalia
KRTAP10-11	1	Echolalia
KRTAP10-3	1	Echolalia
KRTAP10-7	1	Echolalia
KRTAP11-1	1	Echolalia
KRTAP12-2	1	Echolalia
KRTAP12-4	1	Echolalia
KRTAP13-1	1	Echolalia
KRTAP13-2	1	Echolalia
KRTAP13-4	1	Echolalia
KRTAP19-6	1	Echolalia
KRTAP19-8	1	Echolalia
KRTAP25-1	1	Echolalia
KRTAP4-1	1	Echolalia
KRTAP4-9	1	Echolalia
KRTAP5-10	1	Echolalia
KRTAP7-1	1	Echolalia
KRTAP9-1	1	Echolalia
KRTAP9-2	1	Echolalia
KRTAP9-8	1	Echolalia
KSR1	1	Echolalia
KSR2	1	Echolalia
KTN1	1	Echolalia
KXD1	1	Echolalia
KYAT1	1	Echolalia
L1TD1	1	Echolalia
L2HGDH	1	Echolalia
L3HYPDH	1	Echolalia
L3MBTL1	1	Echolalia
L3MBTL3	1	Echolalia
LAMA1	1	Echolalia
LAMA2	1	Echolalia

LAMA5	1	Echolalia
LAMB2	1	Echolalia
LAMC3	1	Echolalia
LAMP2	1	Echolalia
LAMP5	1	Echolalia
LAMTOR4	1	Echolalia
LAMTOR5	1	Echolalia
LANCL1	1	Unresponsiveness to spoken voice
LANCL2	1	Echolalia
LAPTM5	1	Echolalia
LARGE1	1	Echolalia
LARP1	1	Echolalia
LARP1B	1	Echolalia
LARP4B	1	Echolalia
LARS1	1	Echolalia
LAT2	1	Echolalia
LATS2	1	Echolalia
LAX1	1	Motor delay
LAYN	1	Echolalia
LBHD1	1	Echolalia
LCA5	1	Echolalia
LCA5L	1	Echolalia
LCAT	1	Echolalia
LCE1D	1	Echolalia
LCE1E	1	Echolalia
LCE1F	1	Echolalia
LCE2A	1	Motor delay
LCE2D	1	Echolalia
LCE4A	1	Motor delay
LCE5A	1	Echolalia
LCN1	1	Echolalia
LCN10	1	Echolalia
LCN12	1	Echolalia
LCN15	1	Echolalia
LCOR	1	Echolalia
LCORL	1	Echolalia
LCP1	1	Echolalia
LCP2	1	Echolalia
LCT	1	Echolalia
LCTL	1	Echolalia
LDHA	1	Echolalia
LDHAL6B	1	Echolalia
LDLR	1	Echolalia
LDLRAD2	1	Echolalia
LDLRAD4	1	Echolalia
LDLRAP1	1	Echolalia

LEAP2	1	Echolalia
LEF1	1	Unresponsiveness to spoken voice
LEFTY2	1	Echolalia
LEMD1	1	Echolalia
LEO1	1	Language disorder
LEP	1	Echolalia
LEPR	1	Echolalia
LETM1	1	Echolalia
LETM2	1	Unresponsiveness to spoken voice
LGALS1	1	Echolalia
LGALS3	1	Echolalia
LGI2	1	Echolalia
LGI4	1	Echolalia
LGMN	1	Echolalia
LGR4	1	Unresponsiveness to spoken voice
LGR5	1	Echolalia
LGSN	1	Echolalia
LHCGR	1	Echolalia
LHFPL2	1	Echolalia
LHFPL6	1	Echolalia
LHX3	1	Echolalia
LHX8	1	Echolalia
LIAS	1	Echolalia
LIFR	1	Echolalia
LIG1	1	Mutism
LIG4	1	Echolalia
LILRA4	1	Language disorder
LILRB2	1	Echolalia
LILRB3	1	Echolalia
LIMD1	1	Echolalia
LIMK2	1	Echolalia
LIMS2	1	Echolalia
LIN7B	1	Echolalia
LINC00221	1	Echolalia
LINC00226	1	Echolalia
LINC00304	1	Echolalia
LINC00471	1	Echolalia
LINC00473	1	Echolalia
LINC00634	1	Echolalia
LINC00659	1	Echolalia
LINC00944	1	Echolalia
LINC01001	1	Echolalia
LINC01106	1	Echolalia
LINC01168	1	Echolalia
LINC01249	1	Echolalia
LINC01287	1	Echolalia

LINC01297-DUXAP10-NBEAP6	1	Echolalia
LINC01310	1	Echolalia
LINC01410	1	Echolalia
LINC01505	1	Echolalia
LINC01568	1	Unresponsiveness to spoken voice
LINC01570	1	Anxiety
LINC01622	1	Echolalia
LINC01678	1	Echolalia
LINC01750	1	Echolalia
LINC01783	1	Echolalia
LINC01989	1	Echolalia
LINC02028	1	Echolalia
LINC02139	1	Echolalia
LINC02154	1	Echolalia
LINC02182	1	Echolalia
LINC02203	1	Echolalia
LINC02247	1	Echolalia
LINC02259	1	Echolalia
LINC02332	1	Echolalia
LINC02337	1	Echolalia
LINC02350	1	Echolalia
LINC02388	1	Motor delay
LINC02403	1	Motor delay
LINC02414	1	Echolalia
LINC02458	1	Echolalia
LINC02484	1	Echolalia
LINC02537	1	Echolalia
LINC02645	1	Echolalia
LINC02692	1	Echolalia
LINC02694	1	Echolalia
LINC02749	1	Echolalia
LINC02767	1	Echolalia
LINC02868	1	Echolalia
LINC02872	1	Anxiety
LINC02875	1	Echolalia
LINC02876	1	Unresponsiveness to spoken voice
LINC02881	1	Echolalia
LINGO3	1	Echolalia
LINS1	1	Echolalia
LIPA	1	Echolalia
LIPC	1	Echolalia
LIPE	1	Echolalia
LIPE-AS1	1	Echolalia
LIPG	1	Echolalia
LIPJ	1	Echolalia

LIPM	1	Echolalia
LIPT1	1	Echolalia
LKAAEAR1	1	Unresponsiveness to spoken voice
LLPH	1	Echolalia
LMAN1	1	Echolalia
LMAN1L	1	Echolalia
LMBR1	1	Echolalia
LMBR1L	1	Echolalia
LMBRD1	1	Echolalia
LMBRD2	1	Echolalia
LMCD1	1	Echolalia
LMF1	1	Echolalia
LMF1-AS1	1	Echolalia
LMF2	1	Echolalia
LMLN2	1	Echolalia
LMNA	1	Echolalia
LMNTD1	1	Echolalia
LMO2	1	Echolalia
LMO7	1	Echolalia
LMTK3	1	Echolalia
LMX1B	1	Echolalia
LNPEP	1	Echolalia
LNPK	1	Echolalia
LNX1	1	Echolalia
LNX2	1	Echolalia
LOC100128276	1	Echolalia
LOC100128398	1	Echolalia
LOC100128988	1	Echolalia
LOC100129697	1	Echolalia
LOC100129878	1	Echolalia
LOC100130370	1	Echolalia
LOC100130849	1	Echolalia
LOC100130872	1	Echolalia
LOC100132287	1	Echolalia
LOC100132813	1	Echolalia
LOC100134391	1	Echolalia
LOC100287387	1	Echolalia
LOC100288203	1	Echolalia
LOC100288637	1	Echolalia
LOC100288728	1	Echolalia
LOC100289333	1	Echolalia
LOC100422781	1	Echolalia
LOC100505549	1	Echolalia
LOC100506258	1	Echolalia
LOC100507053	1	Echolalia
LOC100996413	1	Echolalia

LOC100996643	1	Echolalia
LOC100996671	1	Echolalia
LOC100996750	1	Echolalia
LOC101059915	1	Echolalia
LOC101926889	1	Echolalia
LOC101927066	1	Echolalia
LOC101927410	1	Echolalia
LOC101927691	1	Echolalia
LOC101927762	1	Echolalia
LOC101927789	1	Echolalia
LOC101927811	1	Echolalia
LOC101928068	1	Echolalia
LOC101928436	1	Echolalia
LOC101928626	1	Echolalia
LOC101928663	1	Echolalia
LOC101928764	1	Echolalia
LOC101929058	1	Echolalia
LOC101929122	1	Echolalia
LOC101929552	1	Echolalia
LOC101929613	1	Echolalia
LOC101929829	1	Echolalia
LOC102723753	1	Echolalia
LOC102724152	1	Echolalia
LOC102724156	1	Echolalia
LOC102724238	1	Echolalia
LOC102724488	1	Echolalia
LOC103171574	1	Echolalia
LOC105369199	1	Echolalia
LOC105369209	1	Unresponsiveness to spoken voice
LOC105369535	1	Echolalia
LOC105370362	1	Echolalia
LOC105370401	1	Echolalia
LOC105370687	1	Echolalia
LOC105370697	1	Echolalia
LOC105370705	1	Echolalia
LOC105370708	1	Echolalia
LOC105370980	1	Unresponsiveness to spoken voice
LOC105371910	1	Echolalia
LOC105373289	1	Echolalia
LOC105373394	1	Echolalia
LOC105375131	1	Echolalia
LOC105375303	1	Echolalia
LOC105375683	1	Echolalia
LOC105376353	1	Echolalia
LOC105376917	1	Echolalia
LOC105377752	1	Echolalia

LOC105377785	1	Echolalia
LOC105378123	1	Echolalia
LOC105378230	1	Echolalia
LOC105378479	1	Echolalia
LOC105378948	1	Echolalia
LOC105378979	1	Echolalia
LOC105379443	1	Echolalia
LOC107984265	1	Echolalia
LOC107984399	1	Echolalia
LOC107984974	1	Echolalia
LOC107985532	1	Echolalia
LOC107985856	1	Echolalia
LOC107985911	1	Echolalia
LOC107985939	1	Echolalia
LOC107986876	1	Echolalia
LOC107987142	1	Echolalia
LOC112577516	1	Echolalia
LOC112577592	1	Echolalia
LOC150935	1	Echolalia
LOC344967	1	Echolalia
LOC389199	1	Echolalia
LOC400682	1	Echolalia
LOC407835	1	Echolalia
LOC440895	1	Echolalia
LOC441155	1	Echolalia
LOC644090	1	Echolalia
LOC645188	1	Echolalia
LOC651337	1	Echolalia
LOC728392	1	Echolalia
LOC728715	1	Echolalia
LOC729254	1	Echolalia
LORICRIN	1	Echolalia
LOXHD1	1	Echolalia
LOXL2	1	Echolalia
LOXL4	1	Echolalia
LPA	1	Echolalia
LPAR1	1	Echolalia
LPAR6	1	Motor delay
LPCAT3	1	Echolalia
LPIN1	1	Echolalia
LPIN3	1	Echolalia
LPL	1	Echolalia
LPP	1	Echolalia
LPXN	1	Anxiety
LRBA	1	Echolalia
LRCH3	1	Echolalia

LRFN2	1	Echolalia
LRFN3	1	Echolalia
LRGUK	1	Echolalia
LRIG2	1	Echolalia
LRIG3	1	Echolalia
LRIT1	1	Echolalia
LRMDA	1	Echolalia
LRP1	1	Echolalia
LRP10	1	Echolalia
LRP11	1	Echolalia
LRP1B	1	Echolalia
LRP2	1	Echolalia
LRP2BP	1	Echolalia
LRP3	1	Echolalia
LRP4	1	Echolalia
LRP5L	1	Echolalia
LRPPRC	1	Echolalia
LRRC19	1	Echolalia
LRRC2	1	Echolalia
LRRC23	1	Echolalia
LRRC27	1	Echolalia
LRRC30	1	Mutism
LRRC34	1	Echolalia
LRRC37A3	1	Echolalia
LRRC37B	1	Echolalia
LRRC3B	1	Echolalia
LRRC40	1	Echolalia
LRRC41	1	Echolalia
LRRC42	1	Echolalia
LRRC43	1	Echolalia
LRRC45	1	Language disorder
LRRC52	1	Echolalia
LRRC53	1	Echolalia
LRRC56	1	Echolalia
LRRC59	1	Echolalia
LRRC61	1	Echolalia
LRRC63	1	Echolalia
LRRC7	1	Echolalia
LRRC70	1	Echolalia
LRRC71	1	Echolalia
LRRC73	1	Unresponsiveness to spoken voice
LRRC75A	1	Echolalia
LRRC8A	1	Echolalia
LRRC8B	1	Echolalia
LRRC8D	1	Echolalia
LRRCC1	1	Echolalia

LRRD1	1	Echolalia
LRRFIP1	1	Echolalia
LRRIQ1	1	Echolalia
LRRIQ3	1	Echolalia
LRRN1	1	Echolalia
LRRN2	1	Echolalia
LRRN3	1	Echolalia
LRTOMT	1	Echolalia
LRWD1	1	Echolalia
LSG1	1	Echolalia
11,00 LSM	1	Echolalia
12,00 LSM	1	Unresponsiveness to spoken voice
LSP1	1	Echolalia
LSR	1	Echolalia
LSS	1	Echolalia
LTB4R	1	Echolalia
LTBP1	1	Echolalia
LTBP2	1	Echolalia
LTBR	1	Echolalia
LTK	1	Echolalia
LTN1	1	Echolalia
LTO1	1	Unresponsiveness to spoken voice
LTV1	1	Echolalia
LURAP1	1	Echolalia
LUZP2	1	Echolalia
LY6D	1	Echolalia
LY6L	1	Echolalia
LY96	1	Echolalia
LYL1	1	Echolalia
LYPD2	1	Echolalia
LYPD4	1	Echolalia
LYPD8	1	Echolalia
LYPD9P	1	Echolalia
LYSMD4	1	Echolalia
LYST	1	Echolalia
LYZL1	1	Echolalia
LZTR1	1	Echolalia
LZTS2	1	Echolalia
MAB21L3	1	Echolalia
MACC1	1	Echolalia
MACF1	1	Echolalia
MACROH2A1	1	Echolalia
MAD1L1	1	Echolalia
MAD2L1BP	1	Echolalia
MADCAM1	1	Echolalia
MADD	1	Echolalia

MAEA	1	Echolalia
MAEL	1	Echolalia
MAG	1	Echolalia
MAGEA1	1	Echolalia
MAGEA4	1	Anxiety
MAGEA6	1	Echolalia
MAGEA8	1	Echolalia
MAGEB10	1	Echolalia
MAGEB16	1	Echolalia
MAGEB18	1	Unresponsiveness to spoken voice
MAGEB3	1	Echolalia
MAGEB4	1	Echolalia
MAGEB6	1	Echolalia
MAGEC1	1	Echolalia
MAGEC3	1	Motor delay
MAGEF1	1	Echolalia
MAGI3	1	Echolalia
MAGIX	1	Echolalia
MAGOHB	1	Echolalia
MAJIN	1	Unresponsiveness to spoken voice
MAK	1	Echolalia
MALRD1	1	Echolalia
MALT1	1	Echolalia
MAMSTR	1	Echolalia
MAN1A1	1	Echolalia
MAN2A1	1	Echolalia
MAN2A2	1	Echolalia
MAN2B2	1	Echolalia
MANBA	1	Echolalia
MAP10	1	Echolalia
MAP1B	1	Echolalia
MAP1LC3B	1	Echolalia
MAP1LC3B2	1	Echolalia
MAP2	1	Echolalia
MAP2K2	1	Echolalia
MAP2K3	1	Echolalia
MAP2K4	1	Language disorder
MAP2K5	1	Echolalia
MAP2K7	1	Echolalia
MAP3K11	1	Motor delay
MAP3K15	1	Echolalia
MAP3K20	1	Echolalia
MAP3K21	1	Echolalia
MAP3K4	1	Echolalia
MAP3K8	1	Echolalia
MAP4K1	1	Echolalia

**MAP4K2**	1	Anxiety
**MAP4K3**	1	Echolalia
**MAP4K5**	1	Echolalia
**MAP6**	1	Echolalia
**MAP6D1**	1	Anxiety
**MAP7**	1	Echolalia
**MAPK10**	1	Echolalia
**MAPK11**	1	Echolalia
**MAPK12**	1	Echolalia
**MAPK14**	1	Echolalia
**MAPK4**	1	Echolalia
**MAPK8IP2**	1	Echolalia
**MAPK8IP3**	1	Echolalia
**MAPK9**	1	Echolalia
**MAPKAPK2**	1	Echolalia
**MAPKAPK5**	1	Echolalia
**MAPKBP1**	1	Echolalia
**MARCHF1**	1	Echolalia
**MARCHF10**	1	Unresponsiveness to spoken voice
**MARCHF3**	1	Echolalia
**MARCHF7**	1	Echolalia
**MARCOL**	1	Echolalia
**MARK1**	1	Echolalia
**MARK2**	1	Unresponsiveness to spoken voice
**MARVELD1**	1	Echolalia
**MARVELD3**	1	Echolalia
**MASP2**	1	Echolalia
**MAST1**	1	Echolalia
**MAST2**	1	Echolalia
**MAST4**	1	Echolalia
**MASTL**	1	Echolalia
**MATK**	1	Echolalia
**MATN1**	1	Echolalia
**MATN3**	1	Echolalia
**MAVS**	1	Echolalia
**MB**	1	Echolalia
**MBD3L2B**	1	Echolalia
**MBD4**	1	Echolalia
**MBL2**	1	Echolalia
**MBLAC2**	1	Echolalia
**MBOAT1**	1	Echolalia
**MBOAT4**	1	Echolalia
**MBP**	1	Echolalia
**MC1R**	1	Echolalia
**MC5R**	1	Echolalia
**MCCC1**	1	Echolalia

MCEE	1	Echolalia
MCF2L2	1	Echolalia
MCM10	1	Echolalia
MCM3	1	Echolalia
MCM3AP	1	Echolalia
MCM4	1	Echolalia
MCM8	1	Echolalia
MCM9	1	Echolalia
MCMBP	1	Echolalia
MCOLN1	1	Echolalia
MCPH1	1	Echolalia
MCTP1	1	Echolalia
MCTP2	1	Echolalia
MCUB	1	Echolalia
MCUR1	1	Echolalia
MDFIC2	1	Aggressive behaviour
MDGA2	1	Echolalia
MDH2	1	Echolalia
MDM2	1	Echolalia
MDM4	1	Echolalia
MDN1	1	Echolalia
MDS2	1	Echolalia
ME2	1	Echolalia
ME3	1	Echolalia
MEA1	1	Echolalia
MEAK7	1	Echolalia
MEAT6	1	Echolalia
MECOM	1	Echolalia
MECP2	1	Unresponsiveness to spoken voice
MED12L	1	Echolalia
MED13	1	Echolalia
MED13L	1	Echolalia
MED14	1	Echolalia
MED16	1	Echolalia
MED23	1	Echolalia
MED29	1	Echolalia
MED31	1	Echolalia
MEDAG	1	Echolalia
MEF2A	1	Echolalia
MEGF10	1	Echolalia
MEGF6	1	Echolalia
MEGF8	1	Echolalia
MEGF9	1	Echolalia
MEI1	1	Echolalia
MEIOC	1	Echolalia
MEIS1	1	Echolalia

MELK	1	Echolalia
MELTF	1	Echolalia
MEN1	1	Anxiety
MEP1A	1	Echolalia
MEPCE	1	Echolalia
MEPE	1	Echolalia
MERTK	1	Echolalia
MESP1	1	Echolalia
MESP2	1	Echolalia
MET	1	Echolalia
METAP1D	1	Echolalia
METRN	1	Echolalia
METTL1	1	Echolalia
METTL14	1	Echolalia
METTL15	1	Echolalia
METTL21C	1	Echolalia
METTL22	1	Echolalia
METTL26	1	Aggressive behaviour
METTL2B	1	Echolalia
METTL4	1	Echolalia
METTL8	1	Echolalia
MEX3C	1	Echolalia
MEX3D	1	Echolalia
MFAP2	1	Echolalia
MFGE8	1	Echolalia
MFHAS1	1	Echolalia
MFSD1	1	Echolalia
MFSD11	1	Language disorder
MFSD12	1	Echolalia
MFSD13A	1	Echolalia
MFSD14A	1	Echolalia
MFSD3	1	Echolalia
MFSD4A	1	Echolalia
MFSD4B	1	Echolalia
MFSD5	1	Echolalia
MFSD6	1	Anxiety
MFSD6L	1	Echolalia
MFSD8	1	Echolalia
MFSD9	1	Echolalia
MGAM	1	Echolalia
MGAT3	1	Echolalia
MGAT5	1	Echolalia
MGLL	1	Echolalia
MGMT	1	Echolalia
MIA2	1	Echolalia
MIB2	1	Echolalia

MICAL1	1	Echolalia
MICAL3	1	Echolalia
MICALL1	1	Echolalia
MICALL2	1	Echolalia
MICOS13	1	Echolalia
MICU2	1	Echolalia
MICU3	1	Echolalia
MID1	1	Anxiety
MID2	1	Echolalia
MIDN	1	Echolalia
MIGA2	1	Echolalia
MIIP	1	Echolalia
MINAR1	1	Echolalia
MINDY2	1	Echolalia
MINDY4B	1	Echolalia
MINK1	1	Echolalia
MIOX	1	Echolalia
MIR12128	1	Echolalia
MIR1268A	1	Echolalia
MIR181A1HG	1	Echolalia
MIR3659HG	1	Language disorder
MIR3675	1	Echolalia
MIR3683	1	Echolalia
MIR3936HG	1	Echolalia
MIR4283-1	1	Echolalia
MIR4289	1	Echolalia
MIR4436A	1	Echolalia
MIR4457	1	Echolalia
MIR4535	1	Echolalia
MIR4686	1	Echolalia
MIR4713HG	1	Echolalia
MIR509-1	1	Echolalia
MIR510	1	Echolalia
MIR5571	1	Language disorder
MIR5703	1	Echolalia
MIR662	1	Echolalia
MIR662;MSLN	1	Sleep disturbances
MIR8071-1	1	Echolalia
MIS18BP1	1	Echolalia
MITD1	1	Echolalia
MITF	1	Echolalia
MKKS	1	Echolalia
MKNK2	1	Echolalia
MLC1	1	Echolalia
1,00 MLF	1	Echolalia
MLH1	1	Motor delay

MLH3	1	Echolalia
MLIP	1	Echolalia
MLKL	1	Echolalia
MLLT3	1	Echolalia
MLN	1	Language disorder
MLPH	1	Echolalia
MLST8	1	Echolalia
MLX	1	Echolalia
MLXIPL	1	Echolalia
MLYCD	1	Echolalia
MMAA	1	Echolalia
MMACHC	1	Echolalia
MMD2	1	Motor delay
MME	1	Echolalia
MMP1	1	Echolalia
MMP12	1	Echolalia
MMP14	1	Echolalia
MMP15	1	Echolalia
MMP17	1	Echolalia
MMP2	1	Echolalia
MMP20	1	Echolalia
MMP21	1	Echolalia
MMP24	1	Echolalia
MMP24-AS1-EDEM2	1	Echolalia
MMP25	1	Echolalia
MMP27	1	Echolalia
MMP3	1	Echolalia
MMP8	1	Echolalia
MMRN2	1	Echolalia
MMS19	1	Echolalia
MMS22L	1	Echolalia
MNT	1	Echolalia
MOB2	1	Echolalia
MOB3A	1	Echolalia
MOB3B	1	Echolalia
MOB3C	1	Echolalia
MOGAT2	1	Motor delay
MOGAT3	1	Echolalia
MOK	1	Echolalia
MORC1	1	Echolalia
MORC3	1	Echolalia
MORN5	1	Echolalia
MOS	1	Echolalia
MOSMO	1	Echolalia
MOSPD2	1	Anxiety

MOV10	1	Echolalia
MOV10L1	1	Echolalia
MPC1	1	Echolalia
MPC2	1	Echolalia
MPDU1	1	Echolalia
MPDZ	1	Echolalia
MPHOSPH6	1	Echolalia
MPHOSPH8	1	Mutism
MPHOSPH9	1	Echolalia
MPI	1	Echolalia
MPL	1	Echolalia
MPND	1	Echolalia
MPO	1	Echolalia
MPP3	1	Echolalia
MPP7	1	Echolalia
MPRIP	1	Echolalia
MPV17L2	1	Echolalia
MPZL2	1	Anxiety
MRC1	1	Echolalia
MRE11	1	Unresponsiveness to spoken voice
MRGPRD	1	Aggressive behaviour
MRGPRX1	1	Echolalia
MRGPRX2	1	Echolalia
MRGPRX3	1	Echolalia
MRM3	1	Echolalia
MROH2A	1	Echolalia
MROH5	1	Echolalia
MROH9	1	Echolalia
MRPL10	1	Echolalia
MRPL11	1	Unresponsiveness to spoken voice
MRPL19	1	Echolalia
MRPL20	1	Echolalia
MRPL22	1	Echolalia
MRPL30	1	Echolalia
MRPL35	1	Echolalia
MRPL38	1	Unresponsiveness to spoken voice
MRPL39	1	Echolalia
MRPL4	1	Echolalia
MRPL44	1	Echolalia
MRPL53	1	Echolalia
MRPL58	1	Echolalia
MRPL9	1	Echolalia
MRPS16	1	Echolalia
MRPS22	1	Unresponsiveness to spoken voice
MRPS30	1	Echolalia
MRPS34	1	Echolalia

MRPS35	1	Echolalia
MRPS5	1	Echolalia
MRS2	1	Echolalia
MS4A14	1	Echolalia
MS4A18	1	Echolalia
MS4A2	1	Echolalia
MS4A4E	1	Echolalia
MS4A6A	1	Echolalia
MS4A7	1	Echolalia
MSGN1	1	Echolalia
MSH2	1	Echolalia
MSH3	1	Echolalia
MSH4	1	Echolalia
MSH6	1	Echolalia
MSI1	1	Sleep disturbances
MSL1	1	Anxiety
MSLN	1	Echolalia
MSR1	1	Echolalia
MSS51	1	Echolalia
MSTN	1	Echolalia
MT1A	1	Echolalia
MT1G	1	Echolalia
MT4	1	Echolalia
MTA1	1	Echolalia
MTAP	1	Echolalia
MTBP	1	Echolalia
MTCH1	1	Echolalia
MTCL1	1	Echolalia
MTERF2	1	Echolalia
MTERF3	1	Unresponsiveness to spoken voice
MTERF4	1	Echolalia
MTF1	1	Echolalia
MTF2	1	Echolalia
MTFP1	1	Echolalia
MTHFD2L	1	Echolalia
MTHFR	1	Echolalia
MTIF3	1	Echolalia
MTMR1	1	Echolalia
MTMR11	1	Echolalia
MTMR2	1	Echolalia
MTMR4	1	Echolalia
MTNR1B	1	Echolalia
MTOR	1	Motor delay
MTR	1	Echolalia
MTREX	1	Echolalia
MTRF1	1	Echolalia

MTRF1L	1	Echolalia
MTRNR2L5	1	Echolalia
MTRR	1	Echolalia
MTSS2	1	Echolalia
MTTP	1	Echolalia
MTURN	1	Echolalia
MTUS1	1	Echolalia
MUC1	1	Echolalia
MUC12	1	Echolalia
MUC15	1	Echolalia
MUC16	1	Echolalia
MUC2	1	Echolalia
MUC5AC	1	Echolalia
MUC7	1	Echolalia
MUS81	1	Echolalia
MUSK	1	Echolalia
MUTYH	1	Echolalia
MVD	1	Echolalia
MVP	1	Motor delay
MX2	1	Echolalia
MXI1	1	Echolalia
MXRA7	1	Echolalia
MYADML2	1	Unresponsiveness to spoken voice
MYBBP1A	1	Echolalia
MYBL2	1	Echolalia
MYBPC1	1	Echolalia
MYBPC2	1	Echolalia
MYBPH	1	Echolalia
MYCBP2	1	Echolalia
MYCL	1	Aggressive behaviour
MYCT1	1	Aggressive behaviour
MYH1	1	Echolalia
MYH10	1	Echolalia
MYH13	1	Echolalia
MYH14	1	Echolalia
MYH15	1	Echolalia
MYH2	1	Echolalia
MYH6	1	Echolalia
MYH7	1	Echolalia
MYH8	1	Echolalia
MYH9	1	Echolalia
MYL10	1	Echolalia
MYL7	1	Echolalia
MYLK	1	Echolalia
MYO10	1	Echolalia
MYO15A	1	Echolalia

MYO15B	1	Echolalia
MYO16	1	Echolalia
MYO18A	1	Echolalia
MYO1C	1	Echolalia
MYO1E	1	Echolalia
MYO1F	1	Echolalia
MYO1G	1	Echolalia
MYO1H	1	Echolalia
MYO3A	1	Echolalia
MYO6	1	Echolalia
MYO9B	1	Echolalia
MYOM2	1	Echolalia
MYORG	1	Echolalia
MYOT	1	Echolalia
MYPN	1	Echolalia
MYPOP	1	Echolalia
MYRIP	1	Echolalia
MYT1L	1	Echolalia
MZB1	1	Echolalia
MZF1	1	Echolalia
MZT2A	1	Echolalia
N6AMT1	1	Echolalia
NAA15	1	Motor delay
NAA16	1	Echolalia
NAALADL2	1	Echolalia
NAB2	1	Echolalia
NACC1	1	Echolalia
NACC2	1	Echolalia
NADSYN1	1	Echolalia
NAF1	1	Echolalia
NAGA	1	Aggressive behaviour
NAGS	1	Echolalia
NALCN	1	Echolalia
NAMPT	1	Echolalia
NANOG	1	Echolalia
NANOGP8	1	Echolalia
NANS	1	Echolalia
NAP1L1	1	Echolalia
NAPA-AS1	1	Echolalia
NAPB	1	Language disorder
NAPG	1	Echolalia
NASP	1	Echolalia
NAT14	1	Echolalia
NAT16	1	Echolalia
NAT8B	1	Echolalia
NAXD	1	Echolalia

NAXE	1	Anxiety
NBAS	1	Echolalia
NBEA	1	Echolalia
NBEAL1	1	Echolalia
NBEAL2	1	Echolalia
NBN	1	Language disorder
NBPF1	1	Echolalia
NBPF10	1	Echolalia
NBPF14	1	Echolalia
NBPF19	1	Echolalia
NBPF20	1	Echolalia
NBPF25P	1	Echolalia
NBPF26	1	Echolalia
NBPF6	1	Echolalia
NBPF7	1	Echolalia
NBPF8	1	Echolalia
NBPF9	1	Echolalia
NBR1	1	Language disorder
NCAM1	1	Echolalia
NCAM1-AS1	1	Echolalia
NCAM2	1	Echolalia
NCAPG	1	Echolalia
NCAPH2	1	Echolalia
NCBP3	1	Unresponsiveness to spoken voice
NCF4	1	Echolalia
NCKAP1	1	Echolalia
NCKAP5L	1	Echolalia
NCKIPSD	1	Sleep disturbances
NCLN	1	Echolalia
NCOA1	1	Echolalia
NCOA2	1	Echolalia
NCOA7	1	Echolalia
NCOR2	1	Echolalia
NDOR1	1	Echolalia
NDRG1	1	Echolalia
NDRG4	1	Echolalia
NDST3	1	Echolalia
NDUFA13	1	Unresponsiveness to spoken voice
NDUFA7	1	Echolalia
NDUFAF1	1	Echolalia
NDUFAF4	1	Echolalia
NDUFAF5	1	Echolalia
NDUFAF6	1	Echolalia
NDUFC2	1	Echolalia
NDUFC2-KCTD14	1	Echolalia
NDUFS2	1	Echolalia

NDUFS4	1	Echolalia
NDUFS8	1	Echolalia
NEB	1	Echolalia
NEBL	1	Echolalia
NECAB2	1	Echolalia
NECAB3	1	Echolalia
NECAP2	1	Motor delay
NECTIN1	1	Echolalia
NECTIN2	1	Echolalia
NECTIN4	1	Echolalia
NEDD1	1	Echolalia
NEDD4	1	Echolalia
NEDD4L	1	Echolalia
NEGR1	1	Echolalia
NEIL3	1	Echolalia
NEK1	1	Echolalia
NEK10	1	Echolalia
NEK4	1	Echolalia
NEK5	1	Echolalia
NELFA	1	Echolalia
NELFCD	1	Echolalia
NELL1	1	Echolalia
NEMF	1	Echolalia
NEO1	1	Echolalia
NEPRO	1	Echolalia
NETO1	1	Echolalia
NEU2	1	Echolalia
NEURL1	1	Echolalia
NEUROD4	1	Echolalia
NEUROG2	1	Echolalia
NFAM1	1	Echolalia
NFASC	1	Echolalia
NFAT5	1	Echolalia
NFATC1	1	Echolalia
NFATC2	1	Echolalia
NFE2L3	1	Echolalia
NFIB	1	Echolalia
NFIC	1	Echolalia
NFILZ	1	Echolalia
NFKB2	1	Echolalia
NFKBIE	1	Echolalia
NFKBIZ	1	Anxiety
NFX1	1	Echolalia
NFXL1	1	Echolalia
NGF	1	Echolalia
NGFR	1	Echolalia

NGLY1	1	Motor delay
NGRN	1	Echolalia
NHEJ1	1	Echolalia
NHP2	1	Echolalia
NHSL2	1	Motor delay
NIBAN1	1	Echolalia
NICN1	1	Echolalia
NID1	1	Echolalia
NID2	1	Echolalia
NIM1K	1	Echolalia
NIN	1	Echolalia
NINJ2	1	Unresponsiveness to spoken voice
NIPA1	1	Echolalia
NIPA2	1	Echolalia
NIPSNAP2	1	Echolalia
NIPSNAP3B	1	Echolalia
NKTR	1	Echolalia
NKX1-1	1	Echolalia
NKX1-2	1	Echolalia
NKX2-5	1	Anxiety
NKX6-2	1	Echolalia
NLGN2	1	Echolalia
NLRC3	1	Echolalia
NLRC5	1	Echolalia
NLRP1	1	Echolalia
NLRP12	1	Echolalia
NLRP14	1	Echolalia
NLRP5	1	Echolalia
NLRP8	1	Echolalia
NLRX1	1	Echolalia
NMB	1	Echolalia
NMBR	1	Echolalia
NMD3	1	Unresponsiveness to spoken voice
NME4	1	Echolalia
NMRK2	1	Echolalia
NMS	1	Echolalia
NMT1	1	Echolalia
NNT	1	Echolalia
NOB1	1	Echolalia
NOBOX	1	Echolalia
NOC2L	1	Echolalia
NOC3L	1	Echolalia
NOD1	1	Echolalia
NOD2	1	Echolalia
NOL3	1	Echolalia
NOL4	1	Echolalia

NOL4L	1	Echolalia
NOL6	1	Echolalia
NOL9	1	Echolalia
NOMO2	1	Echolalia
NONE	1	Echolalia
NOP14	1	Echolalia
NOP56	1	Echolalia
NOP58	1	Echolalia
NOP9	1	Echolalia
NOS1	1	Echolalia
NOTCH2NLA	1	Echolalia
NOTCH2NLB	1	Echolalia
NOTCH2NLR	1	Echolalia
NOTCH3	1	Echolalia
NOVA2	1	Echolalia
NOX3	1	Echolalia
NOX5	1	Echolalia
NOXA1	1	Echolalia
NOXO1	1	Echolalia
NOXRED1	1	Echolalia
NPAS1	1	Aggressive behaviour
NPBWR1	1	Echolalia
NPC1L1	1	Echolalia
NPEPL1	1	Echolalia
NPFFR2	1	Language disorder
NPHP4	1	Echolalia
NPHS1	1	Echolalia
NPIPB11	1	Echolalia
NPIPB12	1	Echolalia
NPIPB15	1	Echolalia
NPIPB5	1	Echolalia
NPIPB6	1	Echolalia
NPIPB9	1	Echolalia
NPL	1	Echolalia
NPNT	1	Echolalia
NPPB	1	Echolalia
2,00 NPR	1	Echolalia
NPRL3	1	Echolalia
NPSR1	1	Echolalia
NPTX2	1	Anxiety
NPTXR	1	Echolalia
NPVF	1	Echolalia
NPY2R	1	Echolalia
NPY4R	1	Echolalia
NPY4R2	1	Echolalia
NPY5R	1	Language disorder

NQO1	1	Echolalia
NR1D2	1	Echolalia
NR1H2	1	Echolalia
NR1I3	1	Echolalia
NR2F6	1	Echolalia
NR3C1	1	Echolalia
NR4A1	1	Echolalia
NR4A3	1	Echolalia
NR5A1	1	Echolalia
NRAP	1	Echolalia
NRBF2	1	Echolalia
NRDC	1	Echolalia
NRDE2	1	Echolalia
NRF1	1	Echolalia
NRG1	1	Echolalia
NRG3	1	Echolalia
NRIP2	1	Echolalia
NRL	1	Echolalia
NRP1	1	Echolalia
NRROS	1	Anxiety
NRTN	1	Echolalia
NRXN1	1	Echolalia
NRXN3	1	Echolalia
NSA2	1	Unresponsiveness to spoken voice
NSD1	1	Echolalia
NSD3	1	Echolalia
NSDHL	1	Echolalia
NSG2	1	Echolalia
NSMAF	1	Echolalia
NSMCE1	1	Echolalia
NSMCE2	1	Echolalia
NSRP1	1	Echolalia
NSUN2	1	Echolalia
NSUN4	1	Echolalia
NSUN7	1	Echolalia
NT5C1B	1	Echolalia
NT5C1B-RDH14	1	Echolalia
NT5C2	1	Echolalia
NT5C3A	1	Echolalia
NT5C3B	1	Echolalia
NT5DC3	1	Echolalia
NT5DC4	1	Echolalia
NT5M	1	Echolalia
NTAQ1	1	Echolalia
NTF3	1	Echolalia
NTM	1	Echolalia

NTN3	1	Echolalia
NTSR2	1	Unresponsiveness to spoken voice
NUAK1	1	Echolalia
NUB1	1	Echolalia
NUBP2	1	Echolalia
NUBPL	1	Echolalia
NUCB1	1	Echolalia
NUCB2	1	Echolalia
NUDCD1	1	Echolalia
NUDT10	1	Echolalia
NUDT12	1	Echolalia
NUDT14	1	Echolalia
NUDT15	1	Echolalia
NUDT17	1	Echolalia
NUDT18	1	Aggressive behaviour
NUDT6	1	Echolalia
NUDT7	1	Echolalia
NUDT8	1	Echolalia
NUF2	1	Echolalia
NUFIP1	1	Echolalia
NUMBL	1	Echolalia
NUP133	1	Echolalia
NUP155	1	Echolalia
NUP188	1	Echolalia
NUP205	1	Echolalia
NUP58	1	Echolalia
NUP62	1	Echolalia
NUP62CL	1	Aggressive behaviour
NUP85	1	Echolalia
NUP88	1	Echolalia
NUP93	1	Echolalia
NUP98	1	Echolalia
NUS1	1	Unresponsiveness to spoken voice
NUTM2A	1	Echolalia
NUTM2B	1	Echolalia
NUTM2D	1	Echolalia
NUTM2E	1	Echolalia
NWD2	1	Echolalia
NXNL2	1	Echolalia
NXPE3	1	Echolalia
NXPH1	1	Echolalia
NXPH4	1	Echolalia
NYAP2	1	Echolalia
NYNRIN	1	Echolalia
NYX	1	Echolalia
OAF	1	Echolalia

OAS1	1	Echolalia
OASL	1	Echolalia
OAZ1	1	Echolalia
OBI1	1	Language disorder
OBSL1	1	Echolalia
OCEL1	1	Echolalia
ODAM	1	Echolalia
ODAPH	1	Echolalia
ODC1	1	Echolalia
ODF2L	1	Echolalia
ODF3	1	Echolalia
ODF3L2	1	Echolalia
OFD1	1	Echolalia
OGA	1	Echolalia
OGDH	1	Echolalia
OGFOD1	1	Echolalia
OGFOD3	1	Echolalia
OGFR	1	Echolalia
OGFRL1	1	Echolalia
OGG1	1	Echolalia
OLAH	1	Echolalia
OLFM2	1	Echolalia
OLFM3	1	Echolalia
OLFM4	1	Echolalia
OLFML1	1	Echolalia
OLFML2A	1	Echolalia
OLFML2B	1	Echolalia
OLIG1	1	Echolalia
OLR1	1	Echolalia
OMA1	1	Echolalia
ONECUT1	1	Echolalia
ONECUT3	1	Aggressive behaviour
OOEP	1	Echolalia
OPA3	1	Echolalia
OPHN1	1	Echolalia
OPN1LW	1	Echolalia
OPN1SW	1	Echolalia
OPN3	1	Echolalia
OPN4	1	Echolalia
OPRD1	1	Language disorder
OPRK1	1	Echolalia
OPRM1	1	Echolalia
OPRPN	1	Echolalia
OR10A2	1	Echolalia
OR10G3	1	Echolalia
OR10G7	1	Echolalia

OR10H1	1	Echolalia
OR10J1	1	Echolalia
OR10J5	1	Echolalia
OR10R2	1	Echolalia
OR10S1	1	Echolalia
OR10V1	1	Echolalia
OR10X1	1	Echolalia
OR10Z1	1	Echolalia
OR11G2	1	Echolalia
OR11H2	1	Echolalia
OR13C2	1	Echolalia
OR13D1	1	Echolalia
OR13F1	1	Echolalia
OR13G1	1	Echolalia
OR14A16	1	Echolalia
OR14C36	1	Echolalia
OR14I1	1	Echolalia
OR1C1	1	Echolalia
OR1D5	1	Echolalia
OR1E1	1	Echolalia
OR1E2	1	Echolalia
OR1F12	1	Echolalia
OR1J2	1	Echolalia
OR1L1	1	Echolalia
OR1L3	1	Echolalia
OR1L6	1	Echolalia
OR1L8	1	Echolalia
OR1N2	1	Echolalia
OR1S1	1	Echolalia
OR1S2	1	Echolalia
OR2A1	1	Echolalia
OR2A14	1	Echolalia
OR2A25	1	Echolalia
OR2A42	1	Echolalia
OR2A5	1	Echolalia
OR2AK2	1	Echolalia
OR2B2	1	Echolalia
OR2B6	1	Echolalia
OR2C1	1	Echolalia
OR2D2	1	Echolalia
OR2F2	1	Echolalia
OR2G2	1	Echolalia
OR2G3	1	Echolalia
OR2L2	1	Anxiety
OR2L3	1	Echolalia
OR2M2	1	Echolalia

OR2M7	1	Echolalia
OR2S2	1	Echolalia
OR2T12	1	Echolalia
OR2T27	1	Echolalia
OR2W5	1	Unresponsiveness to spoken voice
OR2Y1	1	Echolalia
OR3A1	1	Echolalia
OR3A3	1	Echolalia
OR4A5	1	Echolalia
OR4C13	1	Echolalia
OR4C16	1	Echolalia
OR4C45	1	Motor delay
OR4C46	1	Echolalia
OR4C5	1	Echolalia
OR4C6	1	Echolalia
OR4D1	1	Echolalia
OR4D6	1	Echolalia
OR4D9	1	Echolalia
OR4E1	1	Echolalia
OR4E2	1	Echolalia
OR4F15	1	Echolalia
OR4F21	1	Echolalia
OR4F4	1	Echolalia
OR4F5	1	Echolalia
OR4F6	1	Echolalia
OR4K1	1	Echolalia
OR4K13	1	Echolalia
OR4K15	1	Echolalia
OR4K17	1	Aggressive behaviour
OR4K3	1	Echolalia
OR4K5	1	Echolalia
OR4L1	1	Echolalia
OR4N2	1	Echolalia
OR4N4	1	Echolalia
OR4Q2	1	Echolalia
OR4S1	1	Echolalia
OR51A4	1	Echolalia
OR51A7	1	Echolalia
OR51D1	1	Echolalia
OR51E1	1	Echolalia
OR51F2	1	Echolalia
OR51G1	1	Echolalia
OR51L1	1	Echolalia
OR51M1	1	Echolalia
OR51Q1	1	Echolalia
OR51V1	1	Mutism

OR52B4	1	Echolalia
OR52D1	1	Echolalia
OR52E1	1	Echolalia
OR52E2	1	Echolalia
OR52E4	1	Echolalia
OR52I1	1	Echolalia
OR52J3	1	Echolalia
OR52K2	1	Echolalia
OR52L1	1	Echolalia
OR52W1	1	Echolalia
OR56A3	1	Echolalia
OR56A4	1	Echolalia
OR56A5	1	Echolalia
OR5AC2	1	Echolalia
OR5AK2	1	Echolalia
OR5AL1	1	Echolalia
OR5AP2	1	Echolalia
OR5AS1	1	Echolalia
OR5AU1	1	Echolalia
OR5B17	1	Unresponsiveness to spoken voice
OR5B3	1	Unresponsiveness to spoken voice
OR5F1	1	Echolalia
OR5H2	1	Echolalia
OR5K1	1	Echolalia
OR5K2	1	Echolalia
OR5K3	1	Echolalia
OR5K4	1	Echolalia
OR5L2	1	Echolalia
OR5M10	1	Echolalia
OR5M11	1	Echolalia
OR5M8	1	Language disorder
OR6A2	1	Echolalia
OR6B1	1	Echolalia
OR6B3	1	Echolalia
OR6C1	1	Echolalia
OR6C68	1	Echolalia
OR6C74	1	Echolalia
OR6K2	1	Echolalia
OR6M1	1	Echolalia
OR6N2	1	Echolalia
OR6Q1	1	Echolalia
OR6S1	1	Echolalia
OR6Y1	1	Echolalia
OR7A10	1	Echolalia
OR7A17	1	Echolalia
OR7C1	1	Echolalia

OR7C2	1	Echolalia
OR7D2	1	Echolalia
OR7G1	1	Echolalia
OR7G3	1	Echolalia
OR8A1	1	Echolalia
OR8B2	1	Echolalia
OR8D2	1	Echolalia
OR8G1	1	Echolalia
OR8G5	1	Echolalia
OR8H3	1	Echolalia
OR9K2	1	Echolalia
ORAI2	1	Echolalia
ORC1	1	Echolalia
ORC3	1	Echolalia
ORC4	1	Echolalia
ORC6	1	Echolalia
OSBP	1	Echolalia
OSBPL11	1	Echolalia
OSBPL3	1	Echolalia
OSBPL5	1	Echolalia
OSBPL7	1	Echolalia
OSBPL9	1	Echolalia
OSCP1	1	Echolalia
OSER1	1	Echolalia
OSGEPL1	1	Echolalia
OSGIN1	1	Echolalia
OTC	1	Language disorder
OTOA	1	Echolalia
OTOF	1	Echolalia
OTOP1	1	Echolalia
OTOP2	1	Echolalia
OTOP3	1	Echolalia
OTUD1	1	Echolalia
OTUD3	1	Echolalia
OTUD4	1	Echolalia
OTUD5	1	Echolalia
OTUD6B	1	Echolalia
OTULINL	1	Echolalia
OVCH1	1	Echolalia
OVCH1-AS1	1	Echolalia
OVCH2	1	Echolalia
OVOL3	1	Echolalia
OXA1L	1	Echolalia
OXGR1	1	Echolalia
OXLD1	1	Aggressive behaviour
OXNAD1	1	Echolalia

OXSR1	1	Echolalia
OXTR	1	Echolalia
P2RX1	1	Echolalia
P2RX3	1	Anxiety
P2RX4	1	Echolalia
P2RX5	1	Motor delay
P2RX6	1	Echolalia
P2RY1	1	Echolalia
P2RY11	1	Echolalia
P2RY12	1	Echolalia
P2RY13	1	Echolalia
P2RY4	1	Echolalia
P3H1	1	Echolalia
P3H2	1	Echolalia
P3H3	1	Echolalia
P3H4	1	Echolalia
PAAF1	1	Echolalia
PACC1	1	Echolalia
PACRG	1	Echolalia
PACS1	1	Echolalia
PACS2	1	Echolalia
PACSIN2	1	Echolalia
PACSIN3	1	Motor delay
PADI3	1	Echolalia
PADI4	1	Echolalia
PADI6	1	Echolalia
PAEP	1	Echolalia
PAFAH1B2	1	Echolalia
PAFAH2	1	Echolalia
PAG1	1	Echolalia
PAGE2	1	Echolalia
PAGE3	1	Echolalia
PAH	1	Echolalia
PAICS	1	Echolalia
PAIP1	1	Echolalia
PAK2	1	Echolalia
PAK5	1	Echolalia
PAK6	1	Echolalia
PALD1	1	Echolalia
PALM	1	Echolalia
PALM2AKAP2	1	Echolalia
PALM3	1	Echolalia
PAMR1	1	Echolalia
PAN2	1	Echolalia
PANX1	1	Echolalia
PAPPA	1	Echolalia

PAPPA2	1	Echolalia
PAPSS1	1	Echolalia
PAPSS2	1	Echolalia
PAQR4	1	Echolalia
PAQR6	1	Echolalia
PAQR8	1	Echolalia
PARD3	1	Echolalia
PARD3B	1	Echolalia
PARD6G	1	Echolalia
PARL	1	Anxiety
PARP1	1	Echolalia
PARP10	1	Echolalia
PARP12	1	Echolalia
PARP14	1	Echolalia
PARP15	1	Echolalia
PARP2	1	Echolalia
PARP4	1	Echolalia
PARP6	1	Echolalia
PARP9	1	Echolalia
PARVA	1	Echolalia
PASK	1	Echolalia
PATE1	1	Echolalia
PATE4	1	Anxiety
PATJ	1	Echolalia
PATL2	1	Echolalia
PAX2	1	Echolalia
PAX4	1	Echolalia
PAX6	1	Echolalia
PAX6-AS1	1	Echolalia
PAX7	1	Echolalia
PAX8	1	Echolalia
PAX9	1	Echolalia
PAXIP1	1	Echolalia
PBRM1	1	Echolalia
PBX1	1	Anxiety
PCARE	1	Echolalia
PCCA	1	Echolalia
PCDH1	1	Echolalia
PCDH10	1	Echolalia
PCDH11X	1	Echolalia
PCDH11Y	1	Echolalia
PCDH12	1	Echolalia
PCDH15	1	Echolalia
PCDH20	1	Echolalia
PCDH7	1	Echolalia
PCDH8	1	Echolalia

PCDHA1	1	Language disorder
PCDHA13	1	Echolalia
PCDHA3	1	Echolalia
PCDHA4	1	Echolalia
PCDHA7	1	Echolalia
PCDHA8	1	Echolalia
PCDHA9	1	Echolalia
PCDHAC1	1	Echolalia
PCDHB10	1	Echolalia
PCDHB13	1	Echolalia
PCDHB15	1	Echolalia
PCDHB16	1	Echolalia
PCDHB2	1	Echolalia
PCDHB3	1	Echolalia
PCDHB4	1	Echolalia
PCDHGA1	1	Echolalia
PCDHGA10	1	Echolalia
PCDHGA11	1	Echolalia
PCDHGA12	1	Echolalia
PCDHGA2	1	Echolalia
PCDHGA3	1	Echolalia
PCDHGA4	1	Echolalia
PCDHGA5	1	Echolalia
PCDHGA6	1	Echolalia
PCDHGA7	1	Echolalia
PCDHGA8	1	Echolalia
PCDHGA9	1	Echolalia
PCDHGB1	1	Echolalia
PCDHGB2	1	Echolalia
PCDHGB3	1	Echolalia
PCDHGB5	1	Echolalia
PCDHGB6	1	Echolalia
PCDHGC5	1	Echolalia
PCED1A	1	Echolalia
PCED1B	1	Echolalia
PCGF3	1	Echolalia
PCK1	1	Echolalia
PCLO	1	Echolalia
PCM1	1	Echolalia
PCMTD1	1	Echolalia
PCNT	1	Echolalia
PCNX1	1	Echolalia
PCNX2	1	Echolalia
PCNX3	1	Echolalia
PCNX4	1	Echolalia
PCOLCE2	1	Echolalia

PCSK6	1	Echolalia
PCSK7	1	Echolalia
PCSK9	1	Echolalia
PCYOX1L	1	Echolalia
PDCD1LG2	1	Echolalia
PDCD2	1	Echolalia
PDCD4	1	Echolalia
PDCD6	1	Echolalia
PDE10A	1	Echolalia
PDE11A	1	Echolalia
PDE12	1	Language disorder
PDE1B	1	Echolalia
PDE4B	1	Echolalia
PDE4D	1	Echolalia
PDE4DIP	1	Echolalia
PDE4DIPP4	1	Echolalia
PDE6H	1	Aggressive behaviour
PDE8A	1	Echolalia
PDF	1	Echolalia
PDGFB	1	Echolalia
PDGFD	1	Echolalia
PDGFRA	1	Echolalia
PDIA2	1	Echolalia
PDIA3	1	Motor delay
PDIA4	1	Echolalia
PDIK1L	1	Anxiety
PDILT	1	Echolalia
PDK4	1	Echolalia
PDLIM1	1	Echolalia
PDLIM2	1	Echolalia
PDLIM5	1	Echolalia
PDLIM7	1	Echolalia
PDPN	1	Echolalia
PDPR	1	Echolalia
PDS5A	1	Echolalia
PDS5B	1	Language disorder
PDX1	1	Anxiety
PDYN	1	Language disorder
PDZD4	1	Echolalia
PDZRN3	1	Echolalia
PEBP4	1	Echolalia
PECAM1	1	Echolalia
PEG3	1	Echolalia
PELI1	1	Echolalia
PELI2	1	Echolalia
PELO	1	Echolalia

PELP1	1	Echolalia
PEMT	1	Language disorder
PENK	1	Echolalia
PER1	1	Echolalia
PER2	1	Echolalia
PERCC1	1	Aggressive behaviour
PERM1	1	Echolalia
PERP	1	Echolalia
1,00 PES	1	Motor delay
PEX13	1	Language disorder
PEX14	1	Echolalia
PEX3	1	Echolalia
PEX5	1	Echolalia
PEX5L	1	Echolalia
PEX6	1	Echolalia
PFAS	1	Echolalia
PFKFB2	1	Echolalia
PFKL	1	Echolalia
PFN1	1	Echolalia
PFN4	1	Echolalia
PGAM1	1	Echolalia
PGAM5	1	Echolalia
PGAP2	1	Echolalia
PGAP4	1	Echolalia
PGBD3	1	Echolalia
PGBD5	1	Echolalia
PGGHG	1	Echolalia
PGLYRP2	1	Echolalia
PGM2	1	Echolalia
PGM3	1	Echolalia
PGPEP1	1	Language disorder
PGPEP1L	1	Echolalia
PGR	1	Echolalia
PGRMC2	1	Echolalia
PGS1	1	Echolalia
PHACTR1	1	Echolalia
PHACTR2	1	Echolalia
PHACTR3	1	Echolalia
PHACTR4	1	Echolalia
PHAX	1	Echolalia
PHB	1	Echolalia
PHC2	1	Echolalia
PHETA2	1	Echolalia
PHF12	1	Echolalia
PHF2	1	Echolalia
PHF20L1	1	Echolalia

PHGDH	1	Echolalia
PHIP	1	Echolalia
PHKA2	1	Echolalia
PHKB	1	Echolalia
PHLDB2	1	Echolalia
PHLPP1	1	Echolalia
PHLPP2	1	Echolalia
PHOSPHO2-KLHL23	1	Echolalia
PHOX2B	1	Echolalia
PHYHD1	1	Echolalia
PHYKPL	1	Echolalia
PI16	1	Echolalia
PI4K2B	1	Echolalia
PI4KA	1	Echolalia
PI4KB	1	Language disorder
PIAS3	1	Echolalia
PIAS4	1	Echolalia
PICALM	1	Echolalia
PIDD1	1	Echolalia
PIEZO1	1	Echolalia
PIEZO2	1	Echolalia
PIFO	1	Echolalia
PIGB	1	Echolalia
PIGC	1	Echolalia
PIGG	1	Echolalia
PIGL	1	Unresponsiveness to spoken voice
PIGM	1	Echolalia
PIGQ	1	Echolalia
PIGR	1	Echolalia
PIGS	1	Anxiety
PIGT	1	Echolalia
PIGZ	1	Echolalia
PIK3C2A	1	Echolalia
PIK3C2B	1	Echolalia
PIK3C2G	1	Echolalia
PIK3CG	1	Echolalia
PIK3R2	1	Echolalia
PIK3R5	1	Echolalia
PIKFYVE	1	Echolalia
PILRB	1	Echolalia
PIM1	1	Echolalia
PIM3	1	Echolalia
PIMREG	1	Unresponsiveness to spoken voice
PIN1	1	Echolalia
PIN4	1	Echolalia

PINX1	1	Echolalia
PIP4K2A	1	Echolalia
PIP5K1B	1	Echolalia
PITX1	1	Echolalia
PIWIL3	1	Echolalia
PJVK	1	Echolalia
PKD1	1	Echolalia
PKD1L2	1	Echolalia
PKD2	1	Echolalia
PKD2L1	1	Echolalia
PKDREJ	1	Echolalia
PKHD1	1	Echolalia
PKHD1L1	1	Echolalia
PKN1	1	Echolalia
PKP1	1	Echolalia
PKP2	1	Echolalia
PKP3	1	Echolalia
PKP4	1	Motor delay
PLA1A	1	Echolalia
PLA2G1B	1	Echolalia
PLA2G2A	1	Echolalia
PLA2G2C	1	Echolalia
PLA2G4B	1	Echolalia
PLA2G4D	1	Echolalia
PLA2G4F	1	Echolalia
PLA2R1	1	Echolalia
PLAAT1	1	Echolalia
PLAC8	1	Echolalia
PLAGL1	1	Unresponsiveness to spoken voice
PLAGL2	1	Echolalia
PLAUR	1	Echolalia
PLB1	1	Echolalia
PLBD1	1	Unresponsiveness to spoken voice
PLBD2	1	Echolalia
PLCD3	1	Echolalia
PLCD4	1	Anxiety
PLCE1	1	Echolalia
PLCG1	1	Echolalia
PLCG2	1	Echolalia
PLCH1	1	Echolalia
PLCH2	1	Echolalia
PLCL1	1	Echolalia
PLCL2	1	Echolalia
PLCXD3	1	Echolalia
PLD1	1	Echolalia
PLD2	1	Echolalia

PLD4	1	Echolalia
PLD5	1	Echolalia
PLEK	1	Echolalia
PLEK2	1	Echolalia
PLEKHA1	1	Echolalia
PLEKHA3	1	Echolalia
PLEKHA6	1	Echolalia
PLEKHA7	1	Echolalia
PLEKHA8	1	Echolalia
PLEKHG3	1	Echolalia
PLEKHG4B	1	Echolalia
PLEKHG6	1	Echolalia
PLEKHH1	1	Echolalia
PLEKHH3	1	Echolalia
PLEKHN1	1	Echolalia
PLEKHN1;KLHL17	1	Echolalia
PLEKHO2	1	Echolalia
PLEKHS1	1	Echolalia
PLET1	1	Echolalia
PLG	1	Echolalia
PLGLB1	1	Echolalia
PLGLB2	1	Echolalia
PLIN1	1	Echolalia
PLIN2	1	Echolalia
PLK1	1	Echolalia
PLK3	1	Echolalia
PLK4	1	Echolalia
PLK5	1	Echolalia
PLOD1	1	Echolalia
PLPP3	1	Echolalia
PLPP4	1	Echolalia
PLPP7	1	Echolalia
PLPPR5	1	Echolalia
PLS3	1	Echolalia
PLSCR1	1	Echolalia
PLSCR3	1	Echolalia
PLXDC1	1	Echolalia
PLXNA3	1	Echolalia
PLXNB1	1	Echolalia
PLXNB2	1	Echolalia
PLXND1	1	Echolalia
PMAIP1	1	Echolalia
PMEPA1	1	Echolalia
PMF1	1	Echolalia
PMF1-BGLAP	1	Echolalia
PML	1	Echolalia

PMP2	1	Echolalia
PMPCB	1	Anxiety
PMS2	1	Echolalia
PNLIPRP3	1	Echolalia
PNMA6F	1	Echolalia
PNN	1	Echolalia
PNO1	1	Echolalia
PNOC	1	Anxiety
PNPLA1	1	Echolalia
PNPLA2	1	Echolalia
PNPLA5	1	Echolalia
PNPLA6	1	Echolalia
PNPLA7	1	Echolalia
PNPO	1	Echolalia
PNPT1	1	Echolalia
PNRC1	1	Echolalia
POC1B-GALNT4	1	Echolalia
POC5	1	Echolalia
PODN	1	Echolalia
PODXL2	1	Echolalia
POGLUT2	1	Echolalia
POGLUT3	1	Echolalia
POGZ	1	Echolalia
POLA1	1	Echolalia
POLD1	1	Echolalia
POLE	1	Echolalia
POLG	1	Echolalia
POLG2	1	Echolalia
POLI	1	Echolalia
POLK	1	Echolalia
POLL	1	Echolalia
POLN	1	Echolalia
POLR1A	1	Echolalia
POLR1D	1	Echolalia
POLR1E	1	Echolalia
POLR2B	1	Echolalia
POLR2M	1	Echolalia
POLR3A	1	Echolalia
POLR3E	1	Echolalia
POLR3F	1	Echolalia
POLR3H	1	Echolalia
POM121	1	Echolalia
POM121L2	1	Echolalia
POMC	1	Echolalia
POMGNT1	1	Echolalia
POMK	1	Echolalia

POMT2	1	Echolalia
POP1	1	Echolalia
POP4	1	Echolalia
POP5	1	Echolalia
POPDC2	1	Echolalia
POSTN	1	Echolalia
POTEA	1	Anxiety
POTEB3	1	Echolalia
POTEC	1	Echolalia
POTEE	1	Echolalia
POTEG	1	Echolalia
POTEM	1	Echolalia
POU1F1	1	Language disorder
POU2F3	1	Echolalia
POU3F3	1	Echolalia
POU4F3	1	Anxiety
POU5F1B	1	Echolalia
POU6F1	1	Echolalia
POU6F2	1	Echolalia
PPA2	1	Echolalia
PPARA	1	Echolalia
PPARD	1	Echolalia
PPARG	1	Echolalia
PPAT	1	Motor delay
PPFIA2	1	Echolalia
PPFIA4	1	Echolalia
PPIE	1	Echolalia
PPIG	1	Echolalia
PPIP5K2	1	Echolalia
PPL	1	Echolalia
PPM1F	1	Echolalia
PPM1M	1	Echolalia
PPP1R12C	1	Echolalia
PPP1R15A	1	Echolalia
PPP1R16A	1	Echolalia
PPP1R17	1	Echolalia
PPP1R1A	1	Anxiety
PPP1R1B	1	Aggressive behaviour
PPP1R1C	1	Echolalia
PPP1R26	1	Echolalia
PPP1R36	1	Echolalia
PPP1R37	1	Echolalia
PPP1R3G	1	Echolalia
PPP1R9A	1	Echolalia
PPP1R9B	1	Echolalia
PPP2R1A	1	Language disorder

PPP2R1B	1	Echolalia
PPP2R2A	1	Anxiety
PPP2R2B	1	Echolalia
PPP2R2D	1	Echolalia
PPP4R2	1	Echolalia
PPP5C	1	Echolalia
PPP6R1	1	Echolalia
PPP6R3	1	Echolalia
PPY	1	Motor delay
PRADC1	1	Echolalia
PRAG1	1	Echolalia
PRAM1	1	Echolalia
PRAMEF1	1	Echolalia
PRAMEF10	1	Echolalia
PRAMEF11	1	Echolalia
PRAMEF12	1	Echolalia
PRAMEF14	1	Echolalia
PRAMEF15	1	Echolalia
PRAMEF17	1	Echolalia
PRAMEF19	1	Echolalia
PRAMEF2	1	Echolalia
PRAMEF20	1	Echolalia
PRAMEF27	1	Echolalia
PRAMEF4	1	Echolalia
PRAMEF6	1	Echolalia
PRAMEF7	1	Echolalia
PRAMEF9	1	Echolalia
PRCC	1	Echolalia
PRCP	1	Echolalia
PRDM1	1	Echolalia
PRDM10	1	Echolalia
PRDM12	1	Echolalia
PRDM13	1	Echolalia
PRDM15	1	Echolalia
PRDM2	1	Echolalia
PRDM5	1	Echolalia
PRDM6	1	Echolalia
PRDM7	1	Echolalia
PRDM8	1	Echolalia
PRDM9	1	Echolalia
PRDX4	1	Echolalia
PREX1	1	Echolalia
PREX2	1	Echolalia
PRG2	1	Echolalia
PRG3	1	Echolalia
PRG4	1	Echolalia

PRICKLE2	1	Echolalia
PRIM1	1	Echolalia
PRIM2	1	Echolalia
PRKAA2	1	Echolalia
PRKCE	1	Echolalia
PRKCH	1	Echolalia
PRKD2	1	Echolalia
PRKN	1	Echolalia
PRL	1	Anxiety
PRLHR	1	Echolalia
PRM3	1	Echolalia
PRMT2	1	Echolalia
PRMT3	1	Sleep disturbances
PRMT7	1	Echolalia
PROB1	1	Echolalia
PROC	1	Echolalia
PROCR	1	Echolalia
PROKR1	1	Echolalia
PROKR2	1	Echolalia
PROM1	1	Echolalia
PRORP	1	Echolalia
PROSER1	1	Echolalia
PROSER2	1	Echolalia
PROSER3	1	Echolalia
PROZ	1	Echolalia
PRPF18	1	Motor delay
PRPF38B	1	Unresponsiveness to spoken voice
PRPF4B	1	Echolalia
PRPF6	1	Echolalia
PRPH	1	Echolalia
PRPS1L1	1	Echolalia
PRR11	1	Echolalia
PRR12	1	Echolalia
PRR14L	1	Echolalia
PRR18	1	Echolalia
PRR20G	1	Echolalia
PRR22	1	Echolalia
PRR23C	1	Echolalia
PRR29	1	Echolalia
PRR30	1	Anxiety
PRR32	1	Echolalia
PRR34	1	Echolalia
PRR5	1	Echolalia
PRR5-ARHGAP8	1	Echolalia
PRR5L	1	Echolalia
PRRC2B	1	Echolalia

PRRG2	1	Echolalia
PRRG3	1	Language disorder
PRRG4	1	Echolalia
PRRT1B	1	Echolalia
PRSS12	1	Echolalia
PRSS16	1	Echolalia
PRSS21	1	Motor delay
PRSS33	1	Echolalia
PRSS48	1	Echolalia
PRSS50	1	Echolalia
PRSS53	1	Echolalia
PRUNE1	1	Echolalia
PRX	1	Echolalia
PSAPL1	1	Echolalia
PSCA	1	Echolalia
PSD3	1	Echolalia
PSD4	1	Echolalia
PSEN1	1	Echolalia
PSG10P	1	Language disorder
PSG11	1	Echolalia
PSG3	1	Echolalia
PSG6	1	Echolalia
PSG8	1	Echolalia
PSG9	1	Echolalia
PSMA6	1	Echolalia
PSMB10	1	Echolalia
PSMB6	1	Echolalia
PSMB7	1	Echolalia
PSMC3	1	Echolalia
PSMC4	1	Echolalia
PSMD13	1	Echolalia
PSMD2	1	Echolalia
PSMD3	1	Echolalia
PSMD5	1	Mutism
PSMD6	1	Echolalia
PSMD9	1	Echolalia
PSME2	1	Motor delay
PSMG1	1	Echolalia
PSMG2	1	Echolalia
PSMG4	1	Echolalia
PSPH	1	Echolalia
PSPN	1	Echolalia
PSRC1	1	Echolalia
PSTK	1	Echolalia
PSTPIP1	1	Echolalia
PSTPIP2	1	Echolalia

PTBP2	1	Echolalia
PTBP3	1	Echolalia
PTCD1	1	Echolalia
PTCH1	1	Echolalia
PTDSS1	1	Echolalia
PTGDR2	1	Echolalia
PTGER1	1	Anxiety
PTGES3L	1	Echolalia
PTGES3L-AARSD1	1	Echolalia
PTGFRN	1	Echolalia
PTGIR	1	Echolalia
PTGIS	1	Echolalia
PTGR1	1	Echolalia
PTGS1	1	Echolalia
PTGS2	1	Echolalia
PTK2B	1	Echolalia
PTK7	1	Echolalia
PTPN1	1	Echolalia
PTPN12	1	Echolalia
PTPN13	1	Echolalia
PTPN14	1	Echolalia
PTPN20	1	Echolalia
PTPN3	1	Echolalia
PTPN4	1	Echolalia
PTPN5	1	Echolalia
PTPN6	1	Echolalia
PTPRA	1	Echolalia
PTPRC	1	Echolalia
PTPRCAP	1	Echolalia
PTPRD	1	Echolalia
PTPRF	1	Echolalia
PTPRG	1	Echolalia
PTPRH	1	Echolalia
PTPRJ	1	Echolalia
PTPRM	1	Echolalia
PTPRN2	1	Echolalia
PTPRQ	1	Echolalia
PTPRS	1	Echolalia
PTPRU	1	Echolalia
PTPRZ1	1	Echolalia
PTRH1	1	Echolalia
PTTG1IP	1	Echolalia
PTX3	1	Anxiety
PUM3	1	Echolalia
PUS1	1	Echolalia

PUSL1	1	Echolalia
PVALEF	1	Echolalia
PWP1	1	Echolalia
PWRN1	1	Echolalia
PWRN2	1	Echolalia
PWRN3	1	Echolalia
PWWP2A	1	Echolalia
PWWP2B	1	Echolalia
PXK	1	Echolalia
PXMP2	1	Echolalia
PYCR2	1	Echolalia
PYDC5	1	Echolalia
PYGB	1	Echolalia
PYGL	1	Echolalia
PYGM	1	Echolalia
PYGO1	1	Echolalia
PYHIN1	1	Echolalia
PYROXD2	1	Echolalia
PYY2	1	Anxiety
QDPR	1	Echolalia
QPCT	1	Echolalia
QPCTL	1	Motor delay
QRFPR	1	Echolalia
QRICH2	1	Echolalia
QSOX1	1	Echolalia
QSOX2	1	Echolalia
R3HCC1L	1	Echolalia
R3HDM1	1	Echolalia
R3HDM2	1	Echolalia
RAB11FIP1	1	Echolalia
RAB11FIP2	1	Aggressive behaviour
RAB11FIP4	1	Echolalia
RAB11FIP5	1	Echolalia
RAB12	1	Sleep disturbances
RAB15	1	Echolalia
RAB17	1	Echolalia
RAB19	1	Echolalia
RAB1A	1	Echolalia
RAB20	1	Echolalia
RAB26	1	Echolalia
RAB27A	1	Echolalia
RAB36	1	Echolalia
RAB3A	1	Echolalia
RAB3D	1	Anxiety
RAB3IL1	1	Echolalia
RAB40B	1	Echolalia

RAB44	1	Echolalia
RAB5C	1	Echolalia
RAB6C	1	Echolalia
RAB7A	1	Echolalia
RABEP1	1	Echolalia
RABGGTA	1	Echolalia
RAC2	1	Echolalia
RACGAP1	1	Anxiety
RAD1	1	Aggressive behaviour
RAD21	1	Echolalia
RAD21L1	1	Echolalia
RAD23A	1	Echolalia
RAD50	1	Echolalia
RAD51AP2	1	Echolalia
RAD51D	1	Echolalia
RAD52	1	Echolalia
RAD54L	1	Echolalia
RAD9A	1	Echolalia
RADIL	1	Echolalia
RAE1	1	Echolalia
RAET1E	1	Echolalia
RAET1G	1	Echolalia
RAET1L	1	Echolalia
RAF1	1	Echolalia
RAG1	1	Echolalia
RAI1	1	Echolalia
RAI14	1	Echolalia
RALGAPA2	1	Echolalia
RALGAPB	1	Echolalia
RALGDS	1	Echolalia
RALGPS1	1	Echolalia
RALGPS2	1	Echolalia
RAN	1	Echolalia
RANBP17	1	Echolalia
RANBP3L	1	Echolalia
RANBP6	1	Echolalia
RANBP9	1	Echolalia
RAP1GDS1	1	Echolalia
RAPGEF2	1	Echolalia
RAPGEF4	1	Echolalia
RAPGEF6	1	Echolalia
RARG	1	Echolalia
RARRES1	1	Echolalia
RARRES2	1	Echolalia
RARS1	1	Echolalia
RARS2	1	Aggressive behaviour

RASA1	1	Echolalia
RASA2	1	Echolalia
RASA3	1	Echolalia
RASA4B	1	Echolalia
RASAL1	1	Echolalia
RASAL2	1	Echolalia
RASAL3	1	Echolalia
RASGEF1A	1	Echolalia
RASGEF1C	1	Echolalia
RASGRF1	1	Echolalia
RASGRF2	1	Echolalia
RASGRP1	1	Echolalia
RASGRP3	1	Echolalia
RASGRP4	1	Echolalia
RASIP1	1	Echolalia
RASL11A	1	Anxiety
RASSF1	1	Echolalia
RASSF3	1	Echolalia
RASSF5	1	Echolalia
RASSF9	1	Echolalia
RAX	1	Echolalia
RB1CC1	1	Echolalia
RBAKDN	1	Unresponsiveness to spoken voice
RBBP6	1	Echolalia
RBBP8NL	1	Echolalia
RBCK1	1	Echolalia
RBFA	1	Echolalia
RBFOX3	1	Echolalia
RBL2	1	Echolalia
RBM11	1	Echolalia
RBM12B	1	Echolalia
RBM14	1	Echolalia
RBM15	1	Echolalia
RBM17	1	Echolalia
RBM19	1	Echolalia
RBM22	1	Anxiety
RBM33	1	Echolalia
RBM34	1	Echolalia
RBM44	1	Echolalia
RBM47	1	Echolalia
RBM48	1	Echolalia
RBM6	1	Echolalia
RBMS3	1	Echolalia
RBMXL3	1	Echolalia
RBMY1A1	1	Echolalia
RBMY1B	1	Echolalia

RBMY1D	1	Echolalia
RBP3	1	Echolalia
RBP5	1	Echolalia
RBPJ	1	Echolalia
RC3H1	1	Echolalia
RC3H2	1	Echolalia
RCAN3	1	Echolalia
RCBTB1	1	Echolalia
RCBTB2	1	Unresponsiveness to spoken voice
RCC1	1	Echolalia
RCN1	1	Echolalia
RCVRN	1	Echolalia
RD3	1	Motor delay
RD3L	1	Echolalia
RDH5	1	Echolalia
RDX	1	Echolalia
RECK	1	Echolalia
REEP5	1	Sleep disturbances
REG3A	1	Echolalia
REG3G	1	Echolalia
RELL2	1	Echolalia
RELN	1	Echolalia
REM1	1	Echolalia
REP15	1	Echolalia
REPIN1	1	Echolalia
REPS1	1	Echolalia
REPS2	1	Motor delay
RERE	1	Echolalia
RERG	1	Echolalia
RESF1	1	Echolalia
RESP18	1	Aggressive behaviour
RETREG2	1	Echolalia
RETSAT	1	Echolalia
REXO1	1	Echolalia
REXO4	1	Aggressive behaviour
REXO5	1	Echolalia
RFC2	1	Echolalia
RFC4	1	Echolalia
RFC5	1	Echolalia
RFK	1	Echolalia
RFLNA	1	Echolalia
RFNG	1	Echolalia
RFPL3	1	Echolalia
RFPL4A	1	Echolalia
RFPL4AL1	1	Echolalia
RFPL4B	1	Echolalia

RFTN1	1	Echolalia
RFTN2	1	Echolalia
RFWD3	1	Echolalia
RFX1	1	Echolalia
RFX2	1	Echolalia
RFX5	1	Echolalia
RGL1	1	Echolalia
RGL3	1	Echolalia
RGMA	1	Echolalia
RGPD1	1	Echolalia
RGPD2	1	Echolalia
RGPD3	1	Echolalia
RGPD4	1	Echolalia
RGPD5	1	Echolalia
RGPD8	1	Echolalia
RGS11	1	Echolalia
RGS12	1	Echolalia
RGS13	1	Echolalia
RGS20	1	Echolalia
RGS9	1	Echolalia
RGS9BP	1	Echolalia
RHAG	1	Echolalia
RHBDD3	1	Echolalia
RHCE	1	Echolalia
RHD	1	Echolalia
RHEX	1	Echolalia
RHOF	1	Echolalia
RHOT2	1	Echolalia
RHOU	1	Language disorder
RHOXF2	1	Echolalia
RHOXF2B	1	Echolalia
RHPN1	1	Echolalia
RHPN2	1	Echolalia
RIBC2	1	Echolalia
RIC8A	1	Echolalia
RIC8B	1	Echolalia
RICTOR	1	Echolalia
RIF1	1	Echolalia
RIMBP2	1	Echolalia
RIMBP3C	1	Echolalia
RIMKLB	1	Echolalia
RIMS1	1	Echolalia
RIMS2	1	Echolalia
RIN1	1	Echolalia
RIN3	1	Echolalia
RINL	1	Echolalia

RIOK1	1	Echolalia
RIOX1	1	Echolalia
RIPK3	1	Echolalia
RIPOR2	1	Echolalia
RIPOR3	1	Echolalia
RITA1	1	Echolalia
RLIM	1	Echolalia
RLN2	1	Anxiety
RMND5A	1	Mutism
RMND5B	1	Echolalia
RMRP	1	Motor delay
RNASE10	1	Echolalia
RNASE4	1	Echolalia
RNASE6	1	Echolalia
RNASE9	1	Echolalia
RNASEH2A	1	Echolalia
RNASEL	1	Unresponsiveness to spoken voice
RNASET2	1	Echolalia
RND1	1	Echolalia
RNF111	1	Echolalia
RNF113B	1	Echolalia
RNF123	1	Echolalia
RNF130	1	Echolalia
RNF135	1	Echolalia
RNF144A	1	Echolalia
RNF151	1	Unresponsiveness to spoken voice
RNF152	1	Echolalia
RNF157	1	Echolalia
RNF169	1	Echolalia
RNF17	1	Echolalia
RNF180	1	Echolalia
RNF186	1	Motor delay
RNF19B	1	Echolalia
RNF20	1	Echolalia
RNF207-AS1	1	Digestive disorder
RNF212	1	Echolalia
RNF215	1	Echolalia
RNF216	1	Echolalia
RNF217	1	Echolalia
RNF224	1	Echolalia
RNF225	1	Echolalia
RNF25	1	Echolalia
RNF32	1	Echolalia
RNF43	1	Echolalia
RNF6	1	Echolalia
RNFT2	1	Echolalia

RNPEPL1	1	Echolalia
ROBO1	1	Echolalia
ROBO3	1	Echolalia
ROBO4	1	Echolalia
ROPN1	1	Echolalia
ROR2	1	Echolalia
RP1	1	Echolalia
RPAIN	1	Echolalia
RPE65	1	Echolalia
RPF1	1	Echolalia
RPF2	1	Echolalia
RPGR	1	Echolalia
RPGRIP1L	1	Echolalia
RPH3A	1	Echolalia
RPL10	1	Echolalia
RPL23AP53	1	Echolalia
RPL28	1	Echolalia
RPL3	1	Echolalia
RPL32	1	Motor delay
RPL39	1	Echolalia
RPL3L	1	Echolalia
RPL7A	1	Echolalia
RPN1	1	Echolalia
RPP30	1	Echolalia
RPP40	1	Echolalia
RPRD1A	1	Echolalia
RPRD1B	1	Echolalia
RPRD2	1	Echolalia
RPS14	1	Echolalia
RPS15	1	Echolalia
RPS16	1	Echolalia
RPS2	1	Echolalia
RPS6KA1	1	Echolalia
RPS6KA2	1	Echolalia
RPS6KA4	1	Echolalia
RPS6KA5	1	Echolalia
RPS6KC1	1	Echolalia
RPS6KL1	1	Anxiety
RPUSD1	1	Echolalia
RPUSD3	1	Echolalia
RPUSD4	1	Echolalia
RRAGC	1	Echolalia
RRAS	1	Echolalia
RRBP1	1	Echolalia
RREB1	1	Echolalia
RRM1	1	Echolalia

RRM2	1	Echolalia
RRM2B	1	Echolalia
RRN3P2	1	Echolalia
RRP1	1	Echolalia
RRP36	1	Echolalia
RRP7A	1	Echolalia
RRS1	1	Echolalia
RSAD1	1	Echolalia
RSAD2	1	Anxiety
RSBN1	1	Echolalia
RSF1	1	Echolalia
RSPH1	1	Echolalia
RSPH10B	1	Echolalia
RSPH10B2	1	Echolalia
RSPH14	1	Echolalia
RSPH4A	1	Echolalia
RSPH9	1	Echolalia
RSPO4	1	Echolalia
RSRP1	1	Echolalia
RSU1	1	Echolalia
RTCB	1	Echolalia
RTEL1	1	Echolalia
RTF1	1	Echolalia
RTF2	1	Echolalia
RTL1	1	Echolalia
RTL5	1	Echolalia
RTL9	1	Echolalia
RTN1	1	Echolalia
RTN2	1	Echolalia
RTN4	1	Echolalia
RTN4R	1	Echolalia
RTN4RL1	1	Echolalia
RUBCN	1	Echolalia
RUFY4	1	Echolalia
RUNDC3A	1	Echolalia
RUNX2	1	Echolalia
RUSC2	1	Echolalia
RXRG	1	Echolalia
RXYLT1	1	Echolalia
RYK	1	Echolalia
RYR1	1	Echolalia
RYR3	1	Echolalia
S100A13	1	Echolalia
S100A16	1	Echolalia
S100A3	1	Aggressive behaviour
S100A7A	1	Echolalia

S100P	1	Echolalia
S100PBP	1	Echolalia
S1PR5	1	Echolalia
SAA1	1	Echolalia
SAA2	1	Echolalia
SAC3D1	1	Echolalia
SACM1L	1	Echolalia
SACS	1	Echolalia
SAE1	1	Echolalia
SAFB2	1	Echolalia
SAGE1	1	Echolalia
SALL1	1	Echolalia
SALRNA1	1	Echolalia
SAMD12	1	Echolalia
SAMD13	1	Echolalia
SAMD5	1	Echolalia
SAMD9L	1	Echolalia
SAMM50	1	Echolalia
SAP130	1	Echolalia
SAP18	1	Echolalia
SARDH	1	Echolalia
SART3	1	Echolalia
SASH1	1	Echolalia
SAT2	1	Echolalia
SAXO2	1	Echolalia
SBDS	1	Echolalia
SBF1	1	Echolalia
SBNO2	1	Unresponsiveness to spoken voice
SBSN	1	Echolalia
SBSPON	1	Echolalia
SCAF1	1	Echolalia
SCAF11	1	Echolalia
SCAF4	1	Echolalia
SCAI	1	Echolalia
SCAMP4	1	Echolalia
SCAP	1	Echolalia
SCARA3	1	Echolalia
SCARA5	1	Echolalia
SCARB1	1	Echolalia
SCARF2	1	Echolalia
SCART1	1	Echolalia
SCD5	1	Echolalia
SCEL	1	Echolalia
SCFD2	1	Echolalia
SCHIP1	1	Echolalia
SCIN	1	Echolalia

SCLT1	1	Echolalia
SCML4	1	Echolalia
SCN10A	1	Echolalia
SCN11A	1	Echolalia
SCN1A	1	Echolalia
SCN1B	1	Echolalia
SCN2A	1	Echolalia
SCN4A	1	Echolalia
SCN4B	1	Echolalia
SCN7A	1	Echolalia
SCN8A	1	Echolalia
SCN9A	1	Echolalia
SCNM1	1	Echolalia
SCNN1A	1	Echolalia
SCNN1B	1	Echolalia
SCNN1G	1	Echolalia
SCO1	1	Echolalia
SCO2	1	Echolalia
SCP2D1	1	Echolalia
SCPEP1	1	Echolalia
SCRN1	1	Echolalia
SCRN2	1	Echolalia
SCTR	1	Echolalia
SCYL1	1	Language disorder
SCYL2	1	Echolalia
SCYL3	1	Echolalia
SDAD1	1	Echolalia
SDC2	1	Echolalia
SDC3	1	Echolalia
SDHA	1	Echolalia
SDHB	1	Echolalia
SDK1	1	Echolalia
SDK2	1	Echolalia
SDR16C5	1	Echolalia
SDR9C7	1	Unresponsiveness to spoken voice
SDSL	1	Echolalia
SEBOX	1	Echolalia
SEC14L1	1	Echolalia
SEC14L2	1	Echolalia
SEC14L3	1	Echolalia
SEC14L4	1	Echolalia
SEC14L6	1	Echolalia
SEC1P	1	Echolalia
SEC22B2P	1	Echolalia
SEC22C	1	Anxiety
SEC23A	1	Echolalia

SEC23B	1	Echolalia
SEC24B	1	Echolalia
SEC24D	1	Echolalia
SEC31B	1	Echolalia
SEC63	1	Echolalia
SECISBP2L	1	Echolalia
SEH1L	1	Echolalia
SEL1L2	1	Echolalia
SELE	1	Echolalia
SELENBP1	1	Echolalia
SELENON	1	Echolalia
SELENOO	1	Echolalia
SELP	1	Echolalia
SEMA3A	1	Echolalia
SEMA3C	1	Echolalia
SEMA3F-AS1	1	Echolalia
SEMA4A	1	Echolalia
SEMA4B	1	Echolalia
SEMA4C	1	Echolalia
SEMA4D	1	Echolalia
SEMA4G	1	Echolalia
SEMA5A	1	Echolalia
SEMA5B	1	Echolalia
SEMA6C	1	Echolalia
SEMA6D	1	Echolalia
SEMA7A	1	Echolalia
SENP2	1	Echolalia
SENP3	1	Echolalia
SENP6	1	Echolalia
SEPHS1	1	Language disorder
SEPTIN1	1	Echolalia
SEPTIN10	1	Echolalia
SEPTIN3	1	Echolalia
SEPTIN7P11	1	Motor delay
SEPTIN7P13	1	Echolalia
SEPTIN9	1	Echolalia
SERAC1	1	Echolalia
SERGEF	1	Echolalia
SERHL2	1	Echolalia
SERINC1	1	Echolalia
SERINC2	1	Echolalia
SERPINA1	1	Echolalia
SERPINA2	1	Echolalia
SERPINB12	1	Unresponsiveness to spoken voice
SERPINB13	1	Echolalia
SERPINB2	1	Echolalia

SERPINB4	1	Echolalia
SERPINB8	1	Echolalia
SERPIND1	1	Echolalia
SERPINE1	1	Echolalia
SERPINF1	1	Echolalia
SERPINF2	1	Echolalia
SERPING1	1	Mutism
SERPINH1	1	Echolalia
SERPINI1	1	Echolalia
SERPINI2	1	Echolalia
SERTAD1	1	Echolalia
SESN1	1	Echolalia
SETD1A	1	Language disorder
SETD4	1	Echolalia
SETD7	1	Echolalia
SETD9	1	Echolalia
SETDB1	1	Echolalia
SETX	1	Echolalia
SEZ6L	1	Echolalia
SF3B1	1	Echolalia
SF3B3	1	Motor delay
SFMBT2	1	Echolalia
SFN	1	Motor delay
SFSWAP	1	Echolalia
SFT2D1	1	Echolalia
SFT2D3	1	Echolalia
SFTPC	1	Echolalia
SFXN4	1	Echolalia
SGCD	1	Echolalia
SGCE	1	Language disorder
SGCG	1	Echolalia
SGO1	1	Echolalia
SGSM2	1	Echolalia
SGTA	1	Echolalia
SH2B2	1	Echolalia
SH2D1B	1	Echolalia
SH2D3C	1	Echolalia
SH2D4A	1	Echolalia
SH3BGR	1	Echolalia
SH3BP1	1	Echolalia
SH3BP2	1	Echolalia
SH3BP4	1	Echolalia
SH3BP5L	1	Language disorder
SH3GL2	1	Echolalia
SH3GLB2	1	Echolalia
SH3KBP1	1	Unresponsiveness to spoken voice

SH3PXD2A	1	Echolalia
SH3RF1	1	Echolalia
SH3RF2	1	Echolalia
SH3RF3	1	Anxiety
SH3TC1	1	Echolalia
SHANK2	1	Echolalia
SHARPIN	1	Echolalia
SHBG	1	Echolalia
SHC2	1	Echolalia
SHC3	1	Echolalia
SHCBP1L	1	Echolalia
SHH	1	Echolalia
SHISA6	1	Echolalia
SHISA7	1	Aggressive behaviour
SHISA9	1	Echolalia
SHISAL2A	1	Echolalia
SHISAL2B	1	Echolalia
SHKBP1	1	Echolalia
SHLD2	1	Echolalia
SHMT2	1	Echolalia
SHOC2	1	Echolalia
SHPK	1	Echolalia
SHPRH	1	Echolalia
SHQ1	1	Echolalia
SHROOM4	1	Echolalia
SI	1	Echolalia
SIAH2	1	Echolalia
SIDT1	1	Echolalia
SIGLEC1	1	Echolalia
SIGLEC12	1	Echolalia
SIGLEC15	1	Echolalia
SIGLEC16	1	Echolalia
SIGLEC6	1	Echolalia
SIGLEC7	1	Echolalia
SIM1	1	Echolalia
SIMC1	1	Echolalia
SIPA1L1	1	Echolalia
SIPA1L3	1	Echolalia
SIRPB1	1	Echolalia
SIRPD	1	Echolalia
SIRT1	1	Echolalia
SIRT7	1	Echolalia
SIX3	1	Echolalia
SIX4	1	Echolalia
SIX5	1	Echolalia
SKA3	1	Echolalia

SKIDA1	1	Echolalia
SKOR2	1	Echolalia
SLA	1	Echolalia
SLAMF1	1	Echolalia
SLAMF7	1	Echolalia
SLAMF8	1	Language disorder
SLBP	1	Echolalia
SLC10A2	1	Echolalia
SLC10A6	1	Echolalia
SLC11A2	1	Echolalia
SLC12A2	1	Echolalia
SLC12A5	1	Echolalia
SLC12A6	1	Echolalia
SLC13A3	1	Echolalia
SLC13A4	1	Echolalia
SLC14A2	1	Echolalia
SLC15A2	1	Anxiety
SLC15A4	1	Echolalia
SLC15A5	1	Echolalia
SLC16A10	1	Echolalia
SLC16A13	1	Echolalia
SLC16A14	1	Echolalia
SLC16A3	1	Sleep disturbances
SLC16A4	1	Echolalia
SLC16A6	1	Echolalia
SLC16A7	1	Echolalia
SLC16A8	1	Echolalia
SLC17A1	1	Motor delay
SLC17A2	1	Echolalia
SLC17A4	1	Echolalia
SLC17A5	1	Echolalia
SLC17A9	1	Echolalia
SLC18A2	1	Echolalia
SLC18B1	1	Echolalia
SLC19A1	1	Echolalia
SLC19A2	1	Echolalia
SLC19A3	1	Echolalia
SLC1A1	1	Echolalia
SLC1A2	1	Echolalia
SLC1A7	1	Echolalia
SLC22A1	1	Echolalia
SLC22A11	1	Echolalia
SLC22A12	1	Echolalia
SLC22A14	1	Echolalia
SLC22A15	1	Echolalia
SLC22A16	1	Echolalia

SLC22A20P	1	Echolalia
SLC22A23	1	Echolalia
SLC22A25	1	Echolalia
SLC22A4	1	Echolalia
SLC22A6	1	Echolalia
SLC22A7	1	Echolalia
SLC22A8	1	Echolalia
SLC22A9	1	Echolalia
SLC23A1	1	Unresponsiveness to spoken voice
SLC23A2	1	Unresponsiveness to spoken voice
SLC24A1	1	Unresponsiveness to spoken voice
SLC25A15	1	Unresponsiveness to spoken voice
SLC25A19	1	Echolalia
SLC25A22	1	Echolalia
SLC25A23	1	Echolalia
SLC25A24	1	Echolalia
SLC25A25	1	Unresponsiveness to spoken voice
SLC25A3	1	Echolalia
SLC25A31	1	Echolalia
SLC25A38	1	Echolalia
SLC25A41	1	Echolalia
SLC25A42	1	Echolalia
SLC25A45	1	Echolalia
SLC25A47	1	Echolalia
SLC25A5	1	Echolalia
SLC25A51	1	Echolalia
SLC26A1	1	Echolalia
SLC26A10	1	Motor delay
SLC26A3	1	Echolalia
SLC26A4	1	Echolalia
SLC26A7	1	Echolalia
SLC26A8	1	Echolalia
SLC26A9	1	Echolalia
SLC27A1	1	Echolalia
SLC27A2	1	Echolalia
SLC27A4	1	Echolalia
SLC27A5	1	Echolalia
SLC27A6	1	Echolalia
SLC28A1	1	Echolalia
SLC29A1	1	Echolalia
SLC29A4	1	Echolalia
SLC2A1	1	Echolalia
SLC2A2	1	Echolalia
SLC2A3	1	Echolalia
SLC2A6	1	Echolalia
SLC2A7	1	Echolalia

SLC2A8	1	Echolalia
SLC30A5	1	Echolalia
SLC30A9	1	Echolalia
SLC31A2	1	Unresponsiveness to spoken voice
SLC33A1	1	Anxiety
SLC35A4	1	Echolalia
SLC35A5	1	Echolalia
SLC35B1	1	Echolalia
SLC35B4	1	Echolalia
SLC35C1	1	Echolalia
SLC35D1	1	Echolalia
SLC35E1	1	Unresponsiveness to spoken voice
SLC35E3	1	Echolalia
SLC35F3	1	Echolalia
SLC35G2	1	Echolalia
SLC35G4	1	Echolalia
SLC35G5	1	Echolalia
SLC36A1	1	Echolalia
SLC36A2	1	Echolalia
SLC36A3	1	Echolalia
SLC36A4	1	Echolalia
SLC38A1	1	Echolalia
SLC38A11	1	Echolalia
SLC38A2	1	Echolalia
SLC38A4	1	Echolalia
SLC38A8	1	Echolalia
SLC38A9	1	Echolalia
SLC39A3	1	Anxiety
SLC39A4	1	Echolalia
SLC39A5	1	Echolalia
SLC39A8	1	Echolalia
SLC3A1	1	Echolalia
SLC3A2	1	Echolalia
SLC41A1	1	Echolalia
SLC44A2	1	Echolalia
SLC44A3	1	Echolalia
SLC44A5	1	Echolalia
SLC45A4	1	Echolalia
SLC46A2	1	Echolalia
SLC47A1	1	Echolalia
SLC47A2	1	Echolalia
SLC48A1	1	Echolalia
SLC49A3	1	Echolalia
SLC4A1	1	Echolalia
SLC4A11	1	Echolalia
SLC4A1AP	1	Echolalia

SLC4A2	1	Echolalia
SLC4A3	1	Echolalia
SLC4A4	1	Echolalia
SLC4A8	1	Echolalia
SLC51A	1	Echolalia
SLC5A1	1	Echolalia
SLC5A10	1	Echolalia
SLC5A4	1	Echolalia
SLC5A4-AS1	1	Echolalia
SLC5A6	1	Echolalia
SLC5A9	1	Echolalia
SLC6A11	1	Echolalia
SLC6A16	1	Echolalia
SLC6A17	1	Echolalia
SLC6A18	1	Echolalia
SLC6A19	1	Echolalia
SLC6A20	1	Echolalia
SLC6A4	1	Echolalia
SLC6A5	1	Echolalia
SLC6A7	1	Echolalia
SLC6A8	1	Motor delay
SLC6A9	1	Echolalia
SLC7A1	1	Echolalia
SLC7A10	1	Echolalia
SLC7A11	1	Echolalia
SLC7A13	1	Echolalia
SLC7A2	1	Echolalia
SLC7A3	1	Echolalia
SLC7A4	1	Echolalia
SLC7A6OS	1	Echolalia
SLC7A8	1	Echolalia
SLC7A9	1	Echolalia
SLC8B1	1	Echolalia
SLC9A1	1	Echolalia
SLC9A3-AS1	1	Echolalia
SLC9A3R1	1	Echolalia
SLC9A4	1	Echolalia
SLC9B1	1	Echolalia
SLCO1B1	1	Echolalia
SLCO1B3-SLCO1B7	1	Echolalia
SLCO1B7	1	Echolalia
SLCO1C1	1	Echolalia
SLCO3A1	1	Echolalia
SLCO4C1	1	Echolalia
SLCO5A1	1	Echolalia

**SLF1**	1	Echolalia
**SLF2**	1	Echolalia
**SLFN11**	1	Echolalia
**SLFN12**	1	Echolalia
**SLFN12L**	1	Echolalia
**SLFN13**	1	Echolalia
**SLFN14**	1	Echolalia
**SLFN5**	1	Echolalia
**SLFNL1**	1	Echolalia
**SLIT1**	1	Echolalia
**SLIT2**	1	Echolalia
**SLIT3**	1	Echolalia
**SLITRK2**	1	Echolalia
**SLITRK3**	1	Echolalia
**SLITRK4**	1	Echolalia
**SLK**	1	Echolalia
**SLU7**	1	Echolalia
**SLURP2**	1	Echolalia
**SLX1A**	1	Echolalia
**SLX1B**	1	Echolalia
**SMAD3**	1	Echolalia
**SMAD4**	1	Echolalia
**SMAD5**	1	Unresponsiveness to spoken voice
**SMAD6**	1	Echolalia
**SMAD9**	1	Echolalia
**SMAGP**	1	Echolalia
**SMAP1**	1	Echolalia
**SMARCA4**	1	Echolalia
**SMARCA5**	1	Echolalia
**SMARCAD1**	1	Echolalia
**SMARCD2**	1	Echolalia
**SMC6**	1	Echolalia
**SMCHD1**	1	Echolalia
**SMCO3**	1	Echolalia
**SMCO4**	1	Echolalia
**SMG5**	1	Echolalia
**SMG6**	1	Echolalia
**SMG8**	1	Echolalia
**SMIM20**	1	Echolalia
**SMIM22**	1	Echolalia
**SMIM23**	1	Anxiety
**SMIM28**	1	Echolalia
**SMIM29**	1	Echolalia
**SMIM5**	1	Unresponsiveness to spoken voice
**SMNDC1**	1	Echolalia
**SMO**	1	Echolalia

SMOC1	1	Echolalia
SMOX	1	Echolalia
SMPD2	1	Echolalia
SMPD4	1	Echolalia
SMPDL3A	1	Echolalia
SMPDL3B	1	Echolalia
SMTN	1	Echolalia
SMTNL2	1	Echolalia
SMURF1	1	Echolalia
SMURF2	1	Echolalia
SMYD1	1	Echolalia
SMYD3	1	Unresponsiveness to spoken voice
SMYD4	1	Echolalia
SNAI1	1	Echolalia
SNAP47	1	Echolalia
SNAP91	1	Echolalia
SNAPC2	1	Unresponsiveness to spoken voice
SNAPC4	1	Echolalia
SNCAIP	1	Echolalia
SNPH	1	Echolalia
SNRNP200	1	Echolalia
SNRNP48	1	Echolalia
SNTB1	1	Echolalia
SNX19	1	Echolalia
SNX20	1	Echolalia
SNX25	1	Echolalia
SNX29P2	1	Echolalia
SNX31	1	Echolalia
SNX33	1	Echolalia
SNX8	1	Echolalia
SNX9	1	Echolalia
SOAT1	1	Echolalia
SOD2	1	Echolalia
SOHLH1	1	Echolalia
SORCS1	1	Echolalia
SORCS3	1	Echolalia
SORD	1	Echolalia
2 SOS	1	Echolalia
SOWAHA	1	Echolalia
SOWAHB	1	Echolalia
SOX10	1	Echolalia
SOX18	1	Motor delay
SOX2	1	Echolalia
SOX30	1	Echolalia
SOX4	1	Echolalia
SOX9	1	Echolalia

SP100	1	Echolalia
SP140	1	Echolalia
SP140L	1	Echolalia
SP5	1	Echolalia
SP8	1	Language disorder
SPACA6	1	Echolalia
SPAG11A	1	Echolalia
SPAG17	1	Echolalia
SPAG9	1	Echolalia
SPANXB1	1	Echolalia
SPARC	1	Echolalia
SPARCL1	1	Echolalia
SPATA1	1	Echolalia
SPATA12	1	Anxiety
SPATA2	1	Echolalia
SPATA24	1	Echolalia
SPATA2L	1	Echolalia
SPATA3	1	Echolalia
SPATA31A1	1	Echolalia
SPATA31A3	1	Echolalia
SPATA31A5	1	Echolalia
SPATA31A6	1	Echolalia
SPATA31A7	1	Echolalia
SPATA31C1	1	Echolalia
SPATA31D3	1	Unresponsiveness to spoken voice
SPATA31E1	1	Echolalia
SPATA33	1	Echolalia
SPATA5L1	1	Echolalia
SPATA6	1	Motor delay
SPATC1	1	Echolalia
SPATC1L	1	Echolalia
SPATS2L	1	Echolalia
SPDEF	1	Echolalia
SPDL1	1	Echolalia
SPDYE1	1	Echolalia
SPDYE6	1	Echolalia
SPECC1	1	Echolalia
SPECC1L-ADORA2A	1	Echolalia
SPEF1	1	Motor delay
SPEM2	1	Echolalia
SPG11	1	Echolalia
SPG7	1	Echolalia
SPHK2	1	Echolalia
SPI1	1	Echolalia
SPIB	1	Echolalia

SPIC	1	Echolalia
SPICE1	1	Echolalia
SPINK5	1	Echolalia
SPINT1	1	Echolalia
SPINT2	1	Anxiety
SPINT4	1	Echolalia
SPIRE2	1	Echolalia
SPN	1	Echolalia
SPOCK2	1	Echolalia
SPON1	1	Echolalia
SPPL2A	1	Echolalia
SPRED1	1	Echolalia
SPRING1	1	Echolalia
SPRN	1	Echolalia
SPRR1A	1	Echolalia
SPRR1B	1	Echolalia
SPRR3	1	Echolalia
SPRR4	1	Echolalia
SPSB1	1	Echolalia
SPSB4	1	Echolalia
SPTA1	1	Echolalia
SPTAN1	1	Echolalia
SPTB	1	Echolalia
SPTBN1	1	Echolalia
SPTBN4	1	Echolalia
SPTBN5	1	Echolalia
SPTLC3	1	Echolalia
SPTY2D1	1	Echolalia
SPZ1	1	Echolalia
SQLE	1	Echolalia
SQOR	1	Echolalia
SRARP	1	Echolalia
SRCAP	1	Echolalia
SREBF1	1	Echolalia
SREBF2	1	Echolalia
SREK1IP1	1	Unresponsiveness to spoken voice
SRF	1	Echolalia
SRGAP1	1	Motor delay
SRGAP2	1	Echolalia
SRGAP2B	1	Motor delay
SRGN	1	Unresponsiveness to spoken voice
SRMS	1	Echolalia
SRPK1	1	Echolalia
SRPRB	1	Echolalia
SRRD	1	Language disorder
SRRM4	1	Echolalia

**SRRT**	1	Echolalia
**SRSF2**	1	Anxiety
**SRSF4**	1	Echolalia
**SRSF8**	1	Echolalia
**SRSF9**	1	Echolalia
**SS18L1**	1	Echolalia
**SSB**	1	Echolalia
**SSC5D**	1	Echolalia
**SSH1**	1	Echolalia
**SSH3**	1	Echolalia
**SSPOP**	1	Echolalia
**SST**	1	Echolalia
**SSTR3**	1	Echolalia
**SSU72**	1	Anxiety
**SSX2IP**	1	Echolalia
**ST13**	1	Echolalia
**ST18**	1	Echolalia
**ST3GAL1**	1	Echolalia
**ST3GAL4**	1	Echolalia
**ST3GAL5**	1	Echolalia
**ST3GAL6**	1	Echolalia
**ST6GAL2**	1	Echolalia
**ST6GALNAC1**	1	Echolalia
**ST6GALNAC3**	1	Echolalia
**ST7L**	1	Echolalia
**ST8SIA2**	1	Echolalia
**ST8SIA3**	1	Echolalia
**ST8SIA6**	1	Echolalia
**STAB1**	1	Echolalia
**STAG1**	1	Unresponsiveness to spoken voice
**STAG3L5P-PVRIG2P-PILRB**	1	Echolalia
**STAM**	1	Echolalia
**STAMBPL1**	1	Echolalia
**STAP1**	1	Echolalia
**STAP2**	1	Echolalia
**STARD13**	1	Echolalia
**STARD6**	1	Echolalia
**STARD7**	1	Echolalia
**STARD9**	1	Echolalia
**STAT1**	1	Echolalia
**STAT4**	1	Echolalia
**STAU1**	1	Echolalia
**STAU2**	1	Echolalia
**STC2**	1	Echolalia
**STEAP1**	1	Echolalia

STEAP3	1	Echolalia
STEAP4	1	Echolalia
STIM1	1	Unresponsiveness to spoken voice
STIMATE	1	Echolalia
STIMATE-MUSTN1	1	Echolalia
STK11	1	Echolalia
STK11IP	1	Echolalia
STK17B	1	Echolalia
STK3	1	Echolalia
STK31	1	Echolalia
STK32A	1	Echolalia
STK33	1	Echolalia
STK36	1	Echolalia
STK38	1	Language disorder
STK4	1	Echolalia
STMN4	1	Echolalia
1,00 STN	1	Echolalia
STON1-GTF2A1L	1	Echolalia
STON2	1	Echolalia
STOX1	1	Echolalia
STPG1	1	Echolalia
STRN	1	Echolalia
STRN3	1	Echolalia
STT3A	1	Echolalia
STX11	1	Echolalia
STX1B	1	Echolalia
STX2	1	Echolalia
STX6	1	Echolalia
STXBP2	1	Echolalia
STXBP5	1	Unresponsiveness to spoken voice
STXBP5L	1	Echolalia
STYXL1	1	Echolalia
STYXL2	1	Echolalia
SUCLA2	1	Echolalia
SUCNR1	1	Echolalia
SUCO	1	Echolalia
SUFU	1	Echolalia
SUGCT	1	Echolalia
SUGP1	1	Echolalia
SUGP2	1	Echolalia
SULF1	1	Echolalia
SULT1A1	1	Echolalia
SULT1A2	1	Echolalia
SULT2A1	1	Unresponsiveness to spoken voice
SULT2B1	1	Echolalia

SULT4A1	1	Echolalia
SUN1	1	Echolalia
SUN2	1	Echolalia
SUN5	1	Echolalia
SUPT3H	1	Echolalia
SUPT5H	1	Echolalia
SUPT6H	1	Echolalia
SURF2	1	Echolalia
SUSD1	1	Echolalia
SUSD2	1	Echolalia
SUSD4	1	Echolalia
SUSD5	1	Echolalia
SUZ12	1	Echolalia
SV2A	1	Echolalia
SV2B	1	Echolalia
SV2C	1	Echolalia
SVEP1	1	Echolalia
SVIL	1	Echolalia
SVOPL	1	Echolalia
SWAP70	1	Echolalia
SWI5	1	Motor delay
SWSAP1	1	Echolalia
SWT1	1	Language disorder
SYCE1L	1	Echolalia
SYCP1	1	Echolalia
SYF2	1	Echolalia
SYK	1	Echolalia
SYMPK	1	Echolalia
SYN2	1	Echolalia
SYNDIG1	1	Echolalia
SYNDIG1L	1	Echolalia
SYNE1	1	Echolalia
SYNE2	1	Echolalia
SYNE4	1	Echolalia
SYNGR2	1	Echolalia
SYNGR4	1	Echolalia
SYNJ2	1	Echolalia
SYNM	1	Echolalia
SYNPO2	1	Echolalia
SYNRG	1	Echolalia
SYPL1	1	Echolalia
SYT10	1	Echolalia
SYT13	1	Echolalia
SYT14	1	Echolalia
SYT15	1	Echolalia
SYT2	1	Anxiety

SYT5	1	Echolalia
SYT9	1	Echolalia
SYTL2	1	Echolalia
SYTL3	1	Echolalia
SYTL5	1	Echolalia
SZT2	1	Echolalia
TAAR1	1	Echolalia
TAAR2	1	Echolalia
TAAR5	1	Echolalia
TAAR9	1	Echolalia
TAB2	1	Echolalia
TACR1	1	Echolalia
TAF1A	1	Unresponsiveness to spoken voice
TAF3	1	Echolalia
TAF4B	1	Echolalia
TAF7L	1	Motor delay
TAGAP	1	Echolalia
TAL1	1	Echolalia
TAMALIN	1	Echolalia
TAMM41	1	Echolalia
TANC2	1	Echolalia
TANGO2	1	Echolalia
TANGO6	1	Echolalia
TANK	1	Motor delay
TAOK1	1	Echolalia
TAOK3	1	Echolalia
TARDBP	1	Echolalia
TAS1R1	1	Echolalia
TAS1R2	1	Echolalia
TAS2R1	1	Echolalia
TAS2R3	1	Motor delay
TAS2R4	1	Echolalia
TAS2R5	1	Aggressive behaviour
TASOR	1	Echolalia
TASP1	1	Echolalia
TAT	1	Echolalia
TAX1BP1	1	Echolalia
TBC1D10A	1	Echolalia
TBC1D10B	1	Echolalia
TBC1D10C	1	Echolalia
TBC1D14	1	Echolalia
TBC1D17	1	Echolalia
TBC1D22A	1	Echolalia
TBC1D22B	1	Echolalia
TBC1D25	1	Echolalia
TBC1D27P	1	Echolalia

TBC1D28	1	Echolalia
TBC1D31	1	Echolalia
TBC1D4	1	Echolalia
TBC1D7-LOC100130357	1	Echolalia
TBC1D8	1	Echolalia
TBC1D9	1	Echolalia
TBC1D9B	1	Echolalia
TBCA	1	Echolalia
TBCB	1	Echolalia
TBCD	1	Echolalia
TBCEL-TECTA	1	Echolalia
TBKBP1	1	Echolalia
TBL2	1	Echolalia
TBL3	1	Echolalia
TBP	1	Echolalia
TBPL2	1	Echolalia
TBX1	1	Echolalia
TBX10	1	Echolalia
TBX15	1	Echolalia
TBX19	1	Unresponsiveness to spoken voice
TBX20	1	Echolalia
TBX3	1	Anxiety
TBX4	1	Echolalia
TBX5	1	Echolalia
TCAIM	1	Anxiety
TCEAL2	1	Echolalia
TCEANC2	1	Echolalia
TCF4	1	Echolalia
TCF7	1	Echolalia
TCF7L2	1	Echolalia
TCHHL1	1	Unresponsiveness to spoken voice
TCHP	1	Echolalia
TCIRG1	1	Echolalia
TCN1	1	Echolalia
TCN2	1	Echolalia
TCOF1	1	Echolalia
TCP10L	1	Echolalia
TCP10L3	1	Echolalia
TCP11L1	1	Echolalia
TCP11L2	1	Echolalia
TCP11X1	1	Echolalia
TCP11X2	1	Echolalia
TCTE1	1	Echolalia
TCTEX1D1	1	Echolalia
TCTEX1D4	1	Echolalia

TCTN2	1	Echolalia
TCTN3	1	Echolalia
TDGF1	1	Echolalia
TDP2	1	Echolalia
TDRD12	1	Echolalia
TDRD15	1	Echolalia
TDRD3	1	Echolalia
TDRD6	1	Echolalia
TDRD9	1	Echolalia
TEC	1	Unresponsiveness to spoken voice
TECPR1	1	Echolalia
TECPR2	1	Echolalia
TECR	1	Echolalia
TECTA	1	Echolalia
TECTB	1	Motor delay
TEDC2	1	Echolalia
TEDDM1	1	Echolalia
TEK	1	Echolalia
TEKT1	1	Echolalia
TEKT5	1	Echolalia
TENM4	1	Echolalia
TENT2	1	Echolalia
TENT4A	1	Echolalia
TENT4B	1	Echolalia
TENT5A	1	Echolalia
TENT5B	1	Unresponsiveness to spoken voice
TENT5C	1	Echolalia
TEP1	1	Echolalia
TEPP	1	Echolalia
TERB2	1	Aggressive behaviour
TERT	1	Echolalia
TES	1	Echolalia
TESK2	1	Echolalia
TESPA1	1	Echolalia
TET3	1	Echolalia
TEX10	1	Echolalia
TEX101	1	Echolalia
TEX11	1	Anxiety
TEX13A	1	Echolalia
TEX13D	1	Echolalia
TEX14	1	Echolalia
TEX15	1	Echolalia
TEX26	1	Echolalia
TEX33	1	Echolalia
TEX35	1	Echolalia
TEX36	1	Echolalia

TEX45	1	Echolalia
TEX47	1	Echolalia
TEX48	1	Echolalia
TEX52	1	Echolalia
TF	1	Echolalia
TFAP2B	1	Echolalia
TFAP2D	1	Echolalia
TFB2M	1	Echolalia
TFDP1	1	Language disorder
TFDP2	1	Echolalia
TFEB	1	Echolalia
TFEC	1	Echolalia
TFF1	1	Echolalia
TFIP11	1	Echolalia
TFRC	1	Echolalia
TG	1	Echolalia
TGFA	1	Echolalia
TGFB2	1	Echolalia
TGFBI	1	Echolalia
TGFBR2	1	Echolalia
TGFBR3	1	Echolalia
TGFBR3L	1	Echolalia
TGFBRAP1	1	Echolalia
TGIF1	1	Echolalia
TGIF2LX	1	Echolalia
TGM2	1	Echolalia
TGM3	1	Echolalia
TGM5	1	Echolalia
TGM7	1	Echolalia
TGOLN2	1	Echolalia
TGS1	1	Echolalia
THADA	1	Echolalia
THAP10	1	Echolalia
THAP8	1	Echolalia
THAP9	1	Echolalia
THBD	1	Echolalia
THBS1	1	Echolalia
THBS2	1	Echolalia
THBS3	1	Echolalia
THEG	1	Echolalia
THEGL	1	Echolalia
THEM4	1	Echolalia
THEM6	1	Echolalia
THEMIS2	1	Echolalia
THG1L	1	Echolalia
THOC5	1	Echolalia

THOP1	1	Echolalia
THRB	1	Echolalia
THSD1	1	Echolalia
THSD7A	1	Echolalia
THUMPD1	1	Language disorder
THUMPD2	1	Echolalia
THUMPD3	1	Echolalia
TICAM1	1	Echolalia
TIGAR	1	Echolalia
TIGD1	1	Echolalia
TIGD2	1	Echolalia
TIGD4	1	Echolalia
TIMD4	1	Unresponsiveness to spoken voice
TIMELESS	1	Echolalia
TIMM10B	1	Anxiety
TIMM44	1	Echolalia
TIMM50	1	Echolalia
TIMMDC1	1	Echolalia
TIPIN	1	Echolalia
TIRAP	1	Echolalia
TJP2	1	Echolalia
TJP3	1	Echolalia
TK1	1	Echolalia
TK2	1	Echolalia
TLDC2	1	Echolalia
TLE3	1	Echolalia
TLE4	1	Echolalia
TLK1	1	Echolalia
TLN1	1	Echolalia
TLR1	1	Echolalia
TLR10	1	Echolalia
TLR5	1	Echolalia
TLR7	1	Echolalia
TLR8	1	Echolalia
TLR9	1	Echolalia
TLX1	1	Echolalia
TM2D3	1	Echolalia
TM4SF20	1	Echolalia
TM4SF5	1	Echolalia
TM6SF1	1	Echolalia
TM6SF2	1	Echolalia
TM7SF3	1	Echolalia
TM9SF1	1	Echolalia
TM9SF3	1	Echolalia
TMA16	1	Echolalia
TMBIM6	1	Unresponsiveness to spoken voice

TMBIM7P	1	Echolalia
TMC3	1	Echolalia
TMC5	1	Echolalia
TMC6	1	Echolalia
TMC8	1	Echolalia
TMCC2	1	Echolalia
TMCO1	1	Echolalia
TMCO3	1	Echolalia
TMCO6	1	Echolalia
TMED6	1	Echolalia
TMEM105	1	Echolalia
TMEM106C	1	Echolalia
TMEM116	1	Echolalia
TMEM119	1	Echolalia
TMEM120B	1	Echolalia
TMEM128	1	Echolalia
TMEM129	1	Echolalia
TMEM130	1	Language disorder
TMEM131L	1	Echolalia
TMEM132C	1	Echolalia
TMEM140	1	Echolalia
TMEM150B	1	Language disorder
TMEM151A	1	Echolalia
TMEM151B	1	Echolalia
TMEM154	1	Echolalia
TMEM156	1	Echolalia
TMEM159	1	Echolalia
TMEM160	1	Echolalia
TMEM161A	1	Echolalia
TMEM164	1	Echolalia
TMEM17	1	Echolalia
TMEM170B	1	Echolalia
TMEM175	1	Echolalia
TMEM179B	1	Motor delay
TMEM184B	1	Echolalia
TMEM185B	1	Echolalia
TMEM186	1	Echolalia
TMEM187	1	Echolalia
TMEM199	1	Echolalia
TMEM200A	1	Echolalia
TMEM200B	1	Echolalia
TMEM204	1	Echolalia
TMEM208	1	Echolalia
TMEM217	1	Echolalia
TMEM221	1	Echolalia
TMEM222	1	Echolalia

TMEM223	1	Echolalia
TMEM225	1	Echolalia
TMEM231	1	Echolalia
TMEM232	1	Echolalia
TMEM240	1	Echolalia
TMEM242	1	Echolalia
TMEM244	1	Echolalia
TMEM25	1	Echolalia
TMEM253	1	Language disorder
TMEM259	1	Echolalia
TMEM260	1	Echolalia
TMEM262	1	Echolalia
TMEM263	1	Echolalia
TMEM266	1	Echolalia
TMEM273	1	Motor delay
TMEM30B	1	Echolalia
TMEM39B	1	Echolalia
TMEM43	1	Echolalia
TMEM44	1	Echolalia
TMEM45B	1	Echolalia
TMEM51	1	Echolalia
TMEM51-AS1	1	Echolalia
TMEM70	1	Echolalia
TMEM79	1	Echolalia
TMEM82	1	Echolalia
TMEM87B	1	Echolalia
TMEM88	1	Echolalia
TMEM88B	1	Echolalia
TMEM98	1	Echolalia
TMEM99	1	Echolalia
TMF1	1	Echolalia
TMIGD2	1	Echolalia
TMPO	1	Echolalia
TMPPE	1	Anxiety
TMPRSS11A	1	Echolalia
TMPRSS11B	1	Echolalia
TMPRSS11F	1	Echolalia
TMPRSS12	1	Echolalia
TMPRSS13	1	Echolalia
TMPRSS3	1	Echolalia
TMPRSS4	1	Echolalia
TMPRSS5	1	Echolalia
TMPRSS6	1	Echolalia
TMPRSS7	1	Echolalia
TMTC3	1	Echolalia
TMUB1	1	Echolalia

TMX1	1	Echolalia
TMX4	1	Echolalia
TNC	1	Echolalia
TNFAIP2	1	Echolalia
TNFAIP8L1	1	Unresponsiveness to spoken voice
TNFAIP8L2-SCNM1	1	Echolalia
TNFRSF10B	1	Echolalia
TNFRSF13B	1	Echolalia
TNFRSF13C	1	Echolalia
TNFRSF18	1	Echolalia
TNFRSF1A	1	Echolalia
TNFRSF21	1	Anxiety
TNFRSF6B	1	Echolalia
TNFRSF8	1	Echolalia
TNFRSF9	1	Echolalia
TNFSF11	1	Echolalia
TNFSF12-TNFSF13	1	Echolalia
TNFSF13	1	Echolalia
TNFSF14	1	Echolalia
TNFSF15	1	Echolalia
TNFSF18	1	Echolalia
TNFSF4	1	Echolalia
TNFSF9	1	Motor delay
TNIP1	1	Echolalia
TNIP2	1	Echolalia
TNKS2	1	Echolalia
TNN	1	Echolalia
TNNI3	1	Echolalia
TNNT2	1	Echolalia
TNP2	1	Echolalia
TNR	1	Echolalia
TNRC18	1	Echolalia
TNRC6A	1	Echolalia
TNRC6B	1	Echolalia
TNS2	1	Echolalia
TOB2	1	Echolalia
TOE1	1	Echolalia
TOGARAM1	1	Echolalia
TOGARAM2	1	Echolalia
TOLLIP	1	Anxiety
TOM1	1	Echolalia
TOMM40	1	Echolalia
TOMM5	1	Echolalia
TOP2B	1	Echolalia
TOP3A	1	Unresponsiveness to spoken voice

TOP3B	1	Echolalia
TOPAZ1	1	Anxiety
TOPBP1	1	Echolalia
TOPORS	1	Echolalia
TOR1A	1	Echolalia
TOR1B	1	Motor delay
TOX3	1	Echolalia
TOX4	1	Echolalia
TP53I11	1	Echolalia
TP53INP1	1	Echolalia
TP53RK	1	Echolalia
TP63	1	Echolalia
TPBG	1	Echolalia
TPCN1	1	Echolalia
TPCN2	1	Echolalia
TPI1	1	Echolalia
TPM4	1	Echolalia
TPP2	1	Echolalia
TPPP	1	Echolalia
TPPP3	1	Echolalia
TPRX1	1	Echolalia
TPSAB1	1	Echolalia
TPSB2	1	Echolalia
TPSD1	1	Echolalia
TPSG1	1	Echolalia
TPST2	1	Echolalia
TPTE2	1	Echolalia
TPTEP2-CSNK1E	1	Echolalia
TPX2	1	Echolalia
TRABD	1	Echolalia
TRABD2A	1	Echolalia
TRABD2B	1	Echolalia
TRAF1	1	Echolalia
TRAF3IP2	1	Echolalia
TRAF7	1	Aggressive behaviour
TRAIP	1	Language disorder
TRAK1	1	Echolalia
TRAK2	1	Echolalia
TRAM2	1	Echolalia
TRAPPC12	1	Echolalia
TRAPPC13	1	Unresponsiveness to spoken voice
TRAPPC3L	1	Echolalia
TRAPPC5	1	Echolalia
TRAPPC8	1	Echolalia
TRAPPC9	1	Echolalia
TRARG1	1	Echolalia

TRDN	1	Echolalia
TREM1	1	Echolalia
TRERF1	1	Echolalia
TRHDE	1	Echolalia
TRHR	1	Echolalia
TRIM11	1	Echolalia
TRIM21	1	Echolalia
TRIM22	1	Echolalia
TRIM28	1	Echolalia
TRIM3	1	Echolalia
TRIM33	1	Echolalia
TRIM35	1	Echolalia
TRIM38	1	Echolalia
TRIM4	1	Echolalia
TRIM41	1	Echolalia
TRIM48	1	Echolalia
TRIM49	1	Echolalia
TRIM5	1	Echolalia
TRIM51	1	Echolalia
TRIM54	1	Echolalia
TRIM56	1	Echolalia
TRIM58	1	Echolalia
TRIM59	1	Echolalia
TRIM6	1	Echolalia
TRIM6-TRIM34	1	Echolalia
TRIM63	1	Echolalia
TRIM64	1	Echolalia
TRIM65	1	Echolalia
TRIM66	1	Echolalia
TRIM67	1	Echolalia
TRIM68	1	Echolalia
TRIM69	1	Echolalia
TRIM77	1	Echolalia
TRIML1	1	Echolalia
TRIO	1	Echolalia
TRIP10	1	Echolalia
TRIP6	1	Echolalia
TRMT1	1	Echolalia
TRMT10C	1	Echolalia
TRMT13	1	Echolalia
TRMT44	1	Echolalia
TRMT5	1	Anxiety
TRMT6	1	Echolalia
TRMU	1	Echolalia
TRNAU1AP	1	Echolalia
TRO	1	Anxiety

TRPC3	1	Echolalia
TRPC6	1	Echolalia
TRPM2	1	Echolalia
TRPM4	1	Echolalia
TRPM5	1	Echolalia
TRPM6	1	Echolalia
TRPV1	1	Echolalia
TRPV3	1	Echolalia
TRPV4	1	Echolalia
TRRAP	1	Echolalia
TRUB1	1	Echolalia
TRUB2	1	Echolalia
TRY2P	1	Echolalia
TSC2	1	Echolalia
TSC22D1	1	Echolalia
TSG101	1	Echolalia
TSGA10	1	Echolalia
TSHZ1	1	Echolalia
TSKS	1	Echolalia
TSNAXIP1	1	Echolalia
TSPAN11	1	Echolalia
TSPAN14	1	Echolalia
TSPAN17	1	Echolalia
TSPAN2	1	Echolalia
TSPAN31	1	Echolalia
TSPAN32	1	Echolalia
TSPAN7	1	Motor delay
TSPAN8	1	Echolalia
TSPO	1	Echolalia
TSPY1	1	Echolalia
TSPY10	1	Echolalia
TSPY3	1	Echolalia
TSPY4	1	Echolalia
TSPYL1	1	Echolalia
TSPYL4	1	Echolalia
TSPYL5	1	Echolalia
TSR1	1	Echolalia
TSSC4	1	Echolalia
TSSK1B	1	Echolalia
TSSK2	1	Echolalia
TTBK1	1	Echolalia
TTBK2	1	Echolalia
TTC16	1	Echolalia
TTC19	1	Echolalia
TTC21A	1	Echolalia
TTC21B	1	Echolalia

TTC22	1	Echolalia
TTC23	1	Echolalia
TTC27	1	Echolalia
TTC30A	1	Echolalia
TTC30B	1	Echolalia
TTC37	1	Echolalia
TTC39B	1	Echolalia
TTC4	1	Echolalia
TTC7B	1	Echolalia
TTC8	1	Echolalia
TTF1	1	Echolalia
TTF2	1	Echolalia
TTI1	1	Echolalia
TTL	1	Echolalia
TTLL1	1	Echolalia
TTLL11	1	Echolalia
TTLL12	1	Echolalia
TTLL4	1	Echolalia
TTLL5	1	Echolalia
TTLL6	1	Echolalia
TTLL8	1	Echolalia
TTLL9	1	Echolalia
TTN	1	Echolalia
TTR	1	Echolalia
TUBA1A	1	Echolalia
TUBA3C	1	Echolalia
TUBA3D	1	Echolalia
TUBB8	1	Echolalia
TUBB8B	1	Echolalia
TUBD1	1	Echolalia
TUBG1	1	Echolalia
TUBG2	1	Echolalia
TUBGCP2	1	Echolalia
TUBGCP3	1	Echolalia
TUBGCP5	1	Echolalia
TUBGCP6	1	Echolalia
TULP1	1	Echolalia
TULP4	1	Echolalia
TUSC1	1	Echolalia
TUSC3	1	Echolalia
TUT4	1	Echolalia
TWF2	1	Echolalia
TWSG1	1	Echolalia
TXNDC12	1	Anxiety
TXNDC15	1	Echolalia
TXNDC16	1	Echolalia

TXNDC5	1	Echolalia
TXNDC8	1	Echolalia
TXNL1	1	Echolalia
TXNRD3	1	Echolalia
TYRO3	1	Echolalia
TYROBP	1	Echolalia
TYW1B	1	Echolalia
UACA	1	Echolalia
UAP1	1	Echolalia
UAP1L1	1	Echolalia
UBA2	1	Echolalia
UBA52	1	Unresponsiveness to spoken voice
UBA6	1	Echolalia
UBA7	1	Echolalia
UBAC2	1	Echolalia
UBAC2-AS1	1	Echolalia
UBAP1	1	Language disorder
UBAP2	1	Echolalia
UBASH3A	1	Echolalia
UBC	1	Echolalia
UBE2E3	1	Language disorder
UBE2F	1	Motor delay
UBE2M	1	Echolalia
UBE2O	1	Echolalia
UBE3B	1	Echolalia
UBE3C	1	Echolalia
UBE4B	1	Echolalia
UBN1	1	Echolalia
UBN2	1	Echolalia
UBQLN1	1	Echolalia
UBQLN3	1	Echolalia
UBQLNL	1	Echolalia
UBR2	1	Echolalia
UBR3	1	Echolalia
UBR5	1	Echolalia
UBTF	1	Echolalia
UBXN11	1	Echolalia
UBXN2B	1	Echolalia
UBXN6	1	Echolalia
UCK1	1	Echolalia
UCK2	1	Echolalia
UCKL1	1	Echolalia
UCMA	1	Echolalia
UCN3	1	Echolalia
UFL1	1	Echolalia
UFSP1	1	Echolalia

UFSP2	1	Echolalia
UGCG	1	Echolalia
UGGT1	1	Echolalia
UGGT2	1	Echolalia
UGT1A1	1	Echolalia
UGT1A3	1	Echolalia
UGT1A5	1	Echolalia
UGT1A8	1	Echolalia
UGT2A1	1	Echolalia
UGT2A2	1	Echolalia
UGT3A1	1	Echolalia
UGT8	1	Echolalia
UHRF1BP1L	1	Echolalia
UIMC1	1	Echolalia
ULK2	1	Echolalia
UMAD1	1	Echolalia
UMOD	1	Echolalia
UMODL1	1	Echolalia
UNC119B	1	Echolalia
UNC13A	1	Echolalia
UNC13C	1	Echolalia
UNC50	1	Echolalia
UNC5A	1	Echolalia
UNC5B	1	Echolalia
UNC5CL	1	Echolalia
UNC93A	1	Echolalia
UNC93B1	1	Echolalia
UNK	1	Unresponsiveness to spoken voice
UNKL	1	Echolalia
UPB1	1	Echolalia
UPF3A	1	Echolalia
UPF3B	1	Unresponsiveness to spoken voice
UPK1A	1	Echolalia
UPK3A	1	Echolalia
UPK3BL1	1	Echolalia
UPK3BL2	1	Echolalia
UPP1	1	Unresponsiveness to spoken voice
UQCRB	1	Echolalia
UQCRC2	1	Echolalia
UQCRFS1	1	Echolalia
URB1	1	Echolalia
URB2	1	Aggressive behaviour
USF3	1	Echolalia
USH1C	1	Echolalia
USH2A	1	Echolalia
USHBP1	1	Echolalia

USP10	1	Echolalia
USP15	1	Echolalia
USP16	1	Echolalia
USP17L11	1	Echolalia
USP17L15	1	Echolalia
USP17L18	1	Echolalia
USP17L2	1	Echolalia
USP17L22	1	Echolalia
USP17L7	1	Echolalia
USP17L8	1	Echolalia
USP18	1	Echolalia
USP22	1	Echolalia
USP24	1	Echolalia
USP28	1	Echolalia
USP29	1	Echolalia
USP32	1	Echolalia
USP34	1	Echolalia
USP35	1	Echolalia
USP36	1	Echolalia
USP39	1	Echolalia
USP40	1	Echolalia
USP42	1	Echolalia
USP43	1	Echolalia
USP44	1	Echolalia
USP47	1	Echolalia
USP48	1	Echolalia
USP49	1	Echolalia
USP54	1	Echolalia
USP7	1	Echolalia
USP8	1	Echolalia
USP9Y	1	Echolalia
USPL1	1	Motor delay
UST	1	Echolalia
UTP14C	1	Echolalia
UTP15	1	Echolalia
UTP18	1	Echolalia
UTP6	1	Motor delay
UTS2	1	Echolalia
UVSSA	1	Echolalia
VAMP8	1	Echolalia
VANGL1	1	Echolalia
VANGL2	1	Echolalia
VAPA	1	Echolalia
VAPB	1	Echolalia
VASH1	1	Echolalia
VAT1L	1	Echolalia

VAV1	1	Echolalia
VAV2	1	Echolalia
VAV3	1	Echolalia
VAX2	1	Echolalia
VCAM1	1	Echolalia
VCP	1	Echolalia
VCX	1	Echolalia
VDR	1	Echolalia
VEGFA	1	Echolalia
VEGFB	1	Echolalia
VEZF1	1	Echolalia
VEZT	1	Echolalia
VGLL4	1	Echolalia
VIM	1	Echolalia
VIT	1	Echolalia
VKORC1	1	Echolalia
VN1R4	1	Echolalia
VN1R5	1	Echolalia
3,00 VNN	1	Echolalia
VOPP1	1	Echolalia
VPREB1	1	Echolalia
VPS13A	1	Echolalia
VPS13C	1	Echolalia
VPS16	1	Echolalia
VPS25	1	Echolalia
VPS26C	1	Aggressive behaviour
VPS35L	1	Echolalia
VPS37B	1	Motor delay
VPS37C	1	Echolalia
VPS37D	1	Echolalia
VPS39	1	Echolalia
VPS41	1	Echolalia
VPS45	1	Echolalia
VPS4A	1	Echolalia
VPS53	1	Echolalia
VPS8	1	Echolalia
VRK1	1	Echolalia
VRTN	1	Echolalia
VSIG10L2	1	Echolalia
VSIG8	1	Echolalia
VSIR	1	Echolalia
VSTM2A	1	Echolalia
VSX1	1	Echolalia
VTI1A	1	Echolalia
VWA3A	1	Echolalia
VWA3B	1	Echolalia

VWC2	1	Echolalia
VWCE	1	Echolalia
VWDE	1	Echolalia
VXN	1	Echolalia
WAPL	1	Echolalia
WARS2	1	Echolalia
WASF3	1	Echolalia
WBP11	1	Echolalia
WDFY2	1	Echolalia
WDFY3	1	Echolalia
WDFY4	1	Echolalia
WDPCP	1	Echolalia
WDR1	1	Echolalia
WDR20	1	Echolalia
WDR25	1	Echolalia
WDR36	1	Echolalia
WDR48	1	Echolalia
WDR49	1	Echolalia
WDR59	1	Echolalia
WDR5B	1	Echolalia
WDR62	1	Echolalia
WDR75	1	Echolalia
WDR87	1	Echolalia
WDR88	1	Echolalia
WDR97	1	Echolalia
WDTC1	1	Sleep disturbances
WFDC3	1	Echolalia
WFIKKN1	1	Echolalia
WFS1	1	Echolalia
WIPF3	1	Echolalia
WIPI2	1	Echolalia
WIZ	1	Echolalia
WLS	1	Echolalia
WNK2	1	Echolalia
WNK3	1	Unresponsiveness to spoken voice
WNK4	1	Echolalia
WNT11	1	Echolalia
WNT16	1	Echolalia
WNT2	1	Language disorder
WNT5B	1	Echolalia
WNT7A	1	Echolalia
WNT7B	1	Echolalia
WNT8A	1	Echolalia
WNT8B	1	Echolalia
WNT9A	1	Echolalia
WRN	1	Echolalia

WSB1	1	Echolalia
WSB2	1	Echolalia
WSCD1	1	Echolalia
WTAP	1	Echolalia
WTIP	1	Echolalia
WWC2	1	Echolalia
WWOX	1	Echolalia
WWP2	1	Echolalia
XAF1	1	Echolalia
XCL1	1	Echolalia
XCL2	1	Echolalia
XDH	1	Echolalia
XIRP1	1	Echolalia
XPNPEP1	1	Unresponsiveness to spoken voice
XPO1	1	Echolalia
XPO7	1	Echolalia
XPR1	1	Anxiety
XRCC1	1	Echolalia
XRCC5	1	Echolalia
XRRA1	1	Echolalia
XXYLT1	1	Echolalia
XYLB	1	Echolalia
XYLT1	1	Echolalia
YAE1	1	Echolalia
YARS1	1	Echolalia
YARS2	1	Echolalia
YBEY	1	Echolalia
YBX2	1	Echolalia
YBX3	1	Echolalia
YDJC	1	Echolalia
YIF1B	1	Echolalia
YIPF2	1	Motor delay
YIPF3	1	Echolalia
YJU2	1	Echolalia
YLPM1	1	Echolalia
YTHDC2	1	Echolalia
ZAN	1	Echolalia
ZAP70	1	Echolalia
ZAR1L	1	Echolalia
ZBBX	1	Echolalia
ZBED2	1	Echolalia
ZBED4	1	Echolalia
ZBED6	1	Echolalia
ZBED6CL	1	Echolalia
ZBTB10	1	Echolalia
ZBTB17	1	Echolalia

ZBTB18	1	Echolalia
ZBTB21	1	Echolalia
ZBTB25	1	Echolalia
ZBTB37	1	Echolalia
ZBTB40	1	Echolalia
ZBTB43	1	Echolalia
ZBTB45	1	Echolalia
ZBTB46	1	Echolalia
ZBTB47	1	Echolalia
ZBTB48	1	Echolalia
ZBTB7A	1	Echolalia
ZBTB7C	1	Echolalia
ZC2HC1A	1	Digestive disorder
ZC2HC1C	1	Echolalia
ZC3H11A	1	Echolalia
ZC3H11B	1	Echolalia
ZC3H12A	1	Echolalia
ZC3H12C	1	Echolalia
ZC3H13	1	Echolalia
ZC3H3	1	Echolalia
ZC3H4	1	Echolalia
ZC3H6	1	Echolalia
ZC3H7B	1	Echolalia
ZC3HAV1	1	Echolalia
ZC3HAV1L	1	Echolalia
ZC3HC1	1	Echolalia
ZCCHC14	1	Echolalia
ZCCHC24	1	Echolalia
ZCCHC3	1	Echolalia
ZCCHC4	1	Echolalia
ZCCHC7	1	Echolalia
ZCCHC8	1	Echolalia
ZCCHC9	1	Echolalia
ZCWPW1	1	Echolalia
ZDHHC1	1	Echolalia
ZDHHC12	1	Echolalia
ZDHHC17	1	Echolalia
ZDHHC19	1	Echolalia
ZDHHC21	1	Echolalia
ZDHHC23	1	Echolalia
ZDHHC24	1	Echolalia
ZDHHC3	1	Echolalia
ZDHHC7	1	Echolalia
ZDHHC8	1	Echolalia
ZEB2	1	Echolalia
ZER1	1	Echolalia

ZFAND3	1	Echolalia
ZFHX2	1	Echolalia
ZFHX3	1	Echolalia
ZFHX4	1	Echolalia
ZFP28	1	Echolalia
ZFP36	1	Echolalia
ZFP36L2	1	Echolalia
ZFP41	1	Echolalia
ZFP62	1	Echolalia
ZFP64	1	Echolalia
ZFP90	1	Echolalia
ZFPM1	1	Echolalia
ZFR	1	Echolalia
ZFR2	1	Echolalia
ZFYVE16	1	Echolalia
ZFYVE21	1	Echolalia
ZFYVE26	1	Echolalia
ZFYVE27	1	Echolalia
ZFYVE9	1	Echolalia
ZG16B	1	Echolalia
ZGLP1	1	Echolalia
ZGRF1	1	Echolalia
ZHX3	1	Echolalia
ZIC2	1	Echolalia
ZIC4	1	Echolalia
ZKSCAN2	1	Echolalia
ZKSCAN3	1	Echolalia
ZKSCAN4	1	Echolalia
ZKSCAN7	1	Echolalia
ZKSCAN7-AS1	1	Mutism
ZKSCAN8	1	Echolalia
ZMAT4	1	Echolalia
ZMYM5	1	Echolalia
ZMYND12	1	Echolalia
ZMYND15	1	Echolalia
ZMYND8	1	Echolalia
ZNF101	1	Echolalia
ZNF112	1	Echolalia
ZNF12	1	Echolalia
ZNF131	1	Echolalia
ZNF132	1	Echolalia
ZNF133	1	Echolalia
ZNF134	1	Echolalia
ZNF138	1	Echolalia
ZNF154	1	Echolalia
ZNF169	1	Echolalia

ZNF17	1	Echolalia
ZNF177	1	Echolalia
ZNF18	1	Motor delay
ZNF180	1	Echolalia
ZNF181	1	Echolalia
ZNF2	1	Motor delay
ZNF202	1	Echolalia
ZNF205	1	Echolalia
ZNF208	1	Echolalia
ZNF211	1	Echolalia
ZNF212	1	Echolalia
ZNF214	1	Echolalia
ZNF22-AS1	1	Motor delay
ZNF221	1	Echolalia
ZNF222	1	Mutism
ZNF224	1	Echolalia
ZNF225	1	Echolalia
ZNF232	1	Echolalia
ZNF233	1	Echolalia
ZNF234	1	Echolalia
ZNF236	1	Echolalia
ZNF248	1	Echolalia
ZNF260	1	Motor delay
ZNF267	1	Echolalia
ZNF268	1	Echolalia
ZNF273	1	Echolalia
ZNF275	1	Echolalia
ZNF28	1	Echolalia
ZNF280B	1	Echolalia
ZNF283	1	Echolalia
ZNF284	1	Echolalia
ZNF286A	1	Echolalia
ZNF292	1	Echolalia
ZNF30	1	Echolalia
ZNF300	1	Echolalia
ZNF316	1	Echolalia
ZNF318	1	Echolalia
ZNF319	1	Echolalia
ZNF331	1	Echolalia
ZNF334	1	Echolalia
ZNF335	1	Echolalia
ZNF33A	1	Echolalia
ZNF341	1	Echolalia
ZNF346	1	Echolalia
ZNF347	1	Echolalia
ZNF350	1	Echolalia

ZNF354B	1	Motor delay
ZNF354C	1	Echolalia
ZNF358	1	Echolalia
ZNF365	1	Echolalia
ZNF366	1	Echolalia
ZNF385B	1	Echolalia
ZNF385C	1	Echolalia
ZNF396	1	Echolalia
ZNF397	1	Echolalia
ZNF398	1	Echolalia
ZNF41	1	Language disorder
ZNF414	1	Echolalia
ZNF415	1	Echolalia
ZNF418	1	Echolalia
ZNF420	1	Echolalia
ZNF44	1	Echolalia
ZNF440	1	Echolalia
ZNF441	1	Echolalia
ZNF442	1	Echolalia
ZNF443	1	Echolalia
ZNF446	1	Echolalia
ZNF45	1	Echolalia
ZNF451	1	Echolalia
ZNF454	1	Echolalia
ZNF460	1	Echolalia
ZNF462	1	Echolalia
ZNF469	1	Echolalia
ZNF471	1	Echolalia
ZNF479	1	Echolalia
ZNF483	1	Echolalia
ZNF484	1	Echolalia
ZNF485	1	Echolalia
ZNF488	1	Echolalia
ZNF492	1	Echolalia
ZNF493	1	Echolalia
ZNF500	1	Echolalia
ZNF503	1	Echolalia
ZNF507	1	Echolalia
ZNF512	1	Echolalia
ZNF512B	1	Echolalia
ZNF516-DT	1	Echolalia
ZNF518A	1	Echolalia
ZNF518B	1	Echolalia
ZNF519	1	Echolalia
ZNF521	1	Echolalia
ZNF524	1	Echolalia

ZNF525	1	Echolalia
ZNF528	1	Echolalia
ZNF529	1	Echolalia
ZNF532	1	Echolalia
ZNF534	1	Echolalia
ZNF540	1	Echolalia
ZNF546	1	Echolalia
ZNF548	1	Echolalia
ZNF552	1	Echolalia
ZNF555	1	Echolalia
ZNF559-ZNF177	1	Echolalia
ZNF56	1	Echolalia
ZNF561	1	Echolalia
ZNF565	1	Echolalia
ZNF566	1	Echolalia
ZNF568	1	Echolalia
ZNF57	1	Echolalia
ZNF571-AS1	1	Echolalia
ZNF573	1	Echolalia
ZNF577	1	Echolalia
ZNF578	1	Echolalia
ZNF579	1	Echolalia
ZNF582	1	Echolalia
ZNF585A	1	Echolalia
ZNF587	1	Echolalia
ZNF587B	1	Echolalia
ZNF592	1	Echolalia
ZNF596	1	Echolalia
ZNF598	1	Echolalia
ZNF607	1	Echolalia
ZNF608	1	Echolalia
ZNF609	1	Echolalia
ZNF610	1	Echolalia
ZNF614	1	Echolalia
ZNF615	1	Echolalia
ZNF618	1	Echolalia
ZNF620	1	Echolalia
ZNF621	1	Echolalia
ZNF630	1	Echolalia
ZNF638	1	Echolalia
ZNF641	1	Echolalia
ZNF648	1	Echolalia
ZNF649	1	Echolalia
ZNF653	1	Echolalia
ZNF658	1	Echolalia
ZNF662	1	Echolalia

ZNF664	1	Language disorder
ZNF664-RFLNA	1	Echolalia
ZNF667	1	Echolalia
ZNF668	1	Echolalia
ZNF669	1	Echolalia
ZNF677	1	Echolalia
ZNF679	1	Echolalia
ZNF680	1	Echolalia
ZNF681	1	Echolalia
ZNF697	1	Echolalia
ZNF70	1	Echolalia
ZNF700	1	Echolalia
ZNF701	1	Echolalia
ZNF705E	1	Echolalia
ZNF705G	1	Echolalia
ZNF708	1	Echolalia
ZNF71	1	Echolalia
ZNF710	1	Echolalia
ZNF714	1	Echolalia
ZNF717	1	Echolalia
ZNF718	1	Echolalia
ZNF721	1	Echolalia
ZNF723	1	Echolalia
ZNF724	1	Echolalia
ZNF726	1	Echolalia
ZNF727	1	Echolalia
ZNF728	1	Echolalia
ZNF729	1	Unresponsiveness to spoken voice
ZNF732	1	Echolalia
ZNF737	1	Echolalia
ZNF746	1	Echolalia
ZNF763	1	Echolalia
ZNF764	1	Echolalia
ZNF766	1	Echolalia
ZNF768	1	Echolalia
ZNF77	1	Echolalia
ZNF770	1	Unresponsiveness to spoken voice
ZNF772	1	Echolalia
ZNF776	1	Echolalia
ZNF777	1	Echolalia
ZNF780A	1	Echolalia
ZNF781	1	Echolalia
ZNF782	1	Echolalia
ZNF79	1	Echolalia
ZNF791	1	Echolalia
ZNF793	1	Echolalia

ZNF804B	1	Echolalia
ZNF805	1	Echolalia
ZNF808	1	Echolalia
ZNF81	1	Echolalia
ZNF813	1	Echolalia
ZNF816	1	Echolalia
ZNF821	1	Echolalia
ZNF827	1	Echolalia
ZNF83	1	Echolalia
ZNF831	1	Echolalia
ZNF836	1	Echolalia
ZNF837	1	Echolalia
ZNF839	1	Echolalia
ZNF84	1	Echolalia
ZNF841	1	Echolalia
ZNF843	1	Motor delay
ZNF844	1	Echolalia
ZNF845	1	Echolalia
ZNF850	1	Echolalia
ZNF853	1	Echolalia
ZNF860	1	Echolalia
ZNF875	1	Echolalia
ZNF878	1	Motor delay
ZNF880	1	Echolalia
ZNF883	1	Language disorder
ZNF888	1	Echolalia
ZNF93	1	Echolalia
ZNF98	1	Echolalia
ZNF99	1	Echolalia
ZNFX1	1	Echolalia
ZNRF3	1	Echolalia
ZP2	1	Echolalia
ZP3	1	Echolalia
ZPBP2	1	Echolalia
ZPR1	1	Echolalia
ZSCAN1	1	Echolalia
ZSCAN10	1	Echolalia
ZSCAN18	1	Echolalia
ZSCAN20	1	Echolalia
ZSCAN30	1	Echolalia
ZSCAN5C	1	Echolalia
ZSCAN9	1	Echolalia
ZSWIM1	1	Echolalia
ZSWIM2	1	Echolalia
ZSWIM3	1	Echolalia
ZSWIM4	1	Echolalia

ZWILCH	1	Echolalia
ZWINT	1	Echolalia
ZXDC	1	Echolalia
ZYG11A	1	Echolalia
ZYX	1	Echolalia
ZZEF1	1	Echolalia
