# Supplementary material for: Genetic Heterogeneity in Autism Spectrum Disorder: Diagnostic Yield, Recurrent Genes, and Rare Variant-Phenotype Associations from Whole-Exome Sequencing
Source: Int J Mol Sci. 2026 Aug 1;27(15):6901. doi: 10.3390/ijms27156901 (PMC13467236; doi:10.3390/ijms27156901)
Supplement: Supplementary file 1 [file ijms-27-06901-s001.zip › Tables S1 and S2.pdf]

Table S1: Recurrent Rare Variants Within the top 5% recurrent genes.

variant_id	gene	sample_count	gnomad_af	max_af	coding_change	protein_change	diseases	sfari_presence
**chr8:100706805:G>DEL**	PABPC1	18	0	0	Unknown	Unknown	Developmental delay, Autosomal dominant de-novo	Present
**chr7:74753919:A>G**	GTF2I	14	0	0	c.2715A>G	p.Val905=	Williams syndrome,	Present
**chr6:32034963:G>A**	C4B	7	0	0	c.5064G>A	p.Leu1688=	C4B deficiency, | Complement component 4B deficiency, AR | Immunodeficiency due to a classical component pathway complement deficiency, | Systemic lupus erythematosus,	Present
**chr11:96092219:C>INS**	MAML2	6	0	0	Unknown	Unknown	Mucoepidermoid salivary gland carcinoma,	Not present
**chr17:77380262:C>INS**	SEPTIN9	5	0	0	Unknown	Unknown	Amyotrophy, hereditary neuralgic, Autosomal dominant | Neuralgic amyotrophy,	Not present
**chr4:103146038:T>C**	CENPE	4	0,004835843	0,0451974	c.4204A>G	p.Ile1402Val	Microcephaly 13, primary, autosomal recessive, Autosomal recessive | Autosomal recessive primary microcephaly, | Seckel syndrome,	Not present
**chr15:88856875:T>C**	ACAN	3	0,003629524	0,00666667	c.4290T>C	p.Ser1430=	Short stature and advanced bone age, with or without early-onset osteoarthritis and/or osteochondritis dissecans, Autosomal dominant | Spondyloepimetaphyseal dysplasia, aggrecan type, Autosomal recessive | Spondyloepiphyseal dysplasia, Kimberley type, Autosomal dominant | Familial osteochondritis dissecans, Autosomal dominant | Short stature-advanced bone age-early-onset osteoarthritis syndrome,	Not present
**chr2:169128828:C>T**	LRP2	3	0,007229619	0,0255545	c.13803G>A	p.Met4601Ile	Donnai-Barrow syndrome, Autosomal recessive | Faciooculoacousticorenal syndrome, AR	Present
**chr17:77380255:G>INS**	SEPTIN9	3	0	0	Unknown	Unknown	Amyotrophy, hereditary neuralgic, Autosomal dominant | Neuralgic amyotrophy,	Not present
**chr7:152435727:C>T**	KMT2C	3	0,009546793	0,0483368	c.60G>A	p.Glu20=	Kleefstra syndrome 2, Autosomal dominant | Neurodevelopmental disorder distinct from Kleefstra and Kabuki syndromes, Autosomal dominant,Autosomal dominant de-novo | Kleefstra syndrome due to a point mutation,	Present
**chr11:1234256:C>A**	MUC5B	3	0,001886285	0,0108565	c.2429C>A	p.Thr810Asn	Interstitial lung disease 2, Autosomal dominant | Diffuse panbronchiolitis, | Hypersensitivity pneumonitis, | Idiopathic pulmonary fibrosis,	Not present
**chr15:88855993:C>T**	ACAN	3	0	0	c.3408C>T	p.Thr1136=	Short stature and advanced bone age, with or without early-onset osteoarthritis and/or osteochondritis dissecans, Autosomal dominant | Spondyloepimetaphyseal dysplasia, aggrecan type, Autosomal recessive | Spondyloepiphyseal dysplasia, Kimberley type, Autosomal dominant | Familial osteochondritis dissecans, Autosomal dominant | Short stature-advanced bone age-early-onset osteoarthritis syndrome,	Not present
**chr15:88856164:C>T**	ACAN	3	0	0	c.3579C>T	p.Thr1193=	Short stature and advanced bone age, with or without early-onset osteoarthritis and/or osteochondritis dissecans, Autosomal dominant | Spondyloepimetaphyseal dysplasia, aggrecan type, Autosomal recessive | Spondyloepiphyseal dysplasia, Kimberley type, Autosomal dominant | Familial osteochondritis dissecans, Autosomal dominant | Short stature-advanced bone age-early-onset osteoarthritis syndrome,	Not present
**chr7:83134242:T>TAA**	PCLO	3	0,004572405	0,0167708	c.3300+6_3300+7dup	Unknown	Pontocerebellar hypoplasia, type 3, Autosomal recessive	Present

**chr4:125449554:C>T**	FAT4	2	0,008171652	0,0125864	c.8544C>T	p.Ser2848=	Hennekam lymphangiectasia-lymphedema syndrome 2, Autosomal recessive | Van Maldergem syndrome 2, Autosomal recessive | Cerebrofacioarticular syndrome, | Hennekam syndrome,	Not present
**chr22:50462490:C>T**	SBF1	2	0,001323861	0,0139551	c.2128-17G>A	Unknown	Charcot-Marie-Tooth disease, type 4B3, Autosomal recessive	Present
**chr7:152145254:G>A**	KMT2C	2	0,000549256	0,00431542	c.14073C>T	p.Tyr4691=	Kleefstra syndrome 2, Autosomal dominant | Neurodevelopmental disorder distinct from Kleefstra and Kabuki syndromes, Autosomal dominant,Autosomal dominant de-novo | Kleefstra syndrome due to a point mutation,	Present
**chr2:169173147:C>T**	LRP2	2	0,006574433	0,0142237	c.11092G>A	p.Val3698Met	Donnai-Barrow syndrome, Autosomal recessive | Faciooculoacousticorenal syndrome, AR	Present
**chr6:56617082:G>C**	DST	2	1,6648E-05	6,04376E-05	c.6385C>G	p.Leu2129Val	Epidermolysis bullosa simplex 3, localized or generalized intermediate, with bp230 deficiency, Autosomal recessive | Neuropathy, hereditary sensory and autonomic, type VI, Autosomal recessive | Congenital myopathy, Autosomal recessive | Epidermolysis bullosa simplex due to BP230 deficiency, | Hereditary sensory and autonomic neuropathy type 6,	Present
**chr11:1251385:G>A**	MUC5B	2	0,000736545	0,0156146	c.14505G>A	p.Thr4835=	Interstitial lung disease 2, Autosomal dominant | Diffuse panbronchiolitis, | Hypersensitivity pneumonitis, | Idiopathic pulmonary fibrosis,	Not present
**chr12:6936368:A>G**	ATN1	2	0,004371826	0,0164014	c.1101A>G	p.Pro367=	Congenital hypotonia, epilepsy, developmental delay, and digital anomalies, Autosomal dominant | Dentatorubral-pallidoluysian atrophy, Autosomal dominant | Dentatorubro-pallidoluysian atrophy, AD	Not present
**chr22:50459579:G>A**	SBF1	2	0,000284621	0,00283822	c.3579C>T	p.Pro1193=	Charcot-Marie-Tooth disease, type 4B3, Autosomal recessive	Present
**chr4:103139790:C>G**	CENPE	2	0,000817216	0,00464094	c.6203G>C	p.Arg2068Thr	Microcephaly 13, primary, autosomal recessive, Autosomal recessive | Autosomal recessive primary microcephaly, | Seckel syndrome,	Not present
**chr4:103196188:G>A**	CENPE	2	0,000221585	0,000980777	c.213C>T	p.Ile71=	Microcephaly 13, primary, autosomal recessive, Autosomal recessive | Autosomal recessive primary microcephaly, | Seckel syndrome,	Not present
**chr16:2111096:C>A**	PKD1	2	0,007171736	0,0107499	c.4071G>T	p.Leu1357=	Polycystic kidney disease 1, Autosomal dominant | Polycystic kidney disease 1 with or without polycystic liver disease, AD | Autosomal dominant polycystic kidney disease, | Autosomal dominant polycystic kidney disease type 1 with tuberous sclerosis,	Present
**chr15:88838778:C>T**	ACAN	2	0,000941275	0,010512	c.186C>T	p.Thr62=	Short stature and advanced bone age, with or without early-onset osteoarthritis and/or osteochondritis dissecans, Autosomal dominant | Spondyloepimetaphyseal dysplasia, aggrecan type, Autosomal recessive | Spondyloepiphyseal dysplasia, Kimberley type, Autosomal dominant | Familial osteochondritis dissecans, Autosomal dominant | Short stature-advanced bone age-early-onset osteoarthritis syndrome,	Not present
**chr15:88840109:G>A**	ACAN	2	0,001448761	0,0162889	c.552G>A	p.Thr184=	Short stature and advanced bone age, with or without early-onset osteoarthritis and/or osteochondritis dissecans, Autosomal dominant | Spondyloepimetaphyseal dysplasia, aggrecan type, Autosomal recessive | Spondyloepiphyseal dysplasia, Kimberley type, Autosomal dominant | Familial osteochondritis dissecans, Autosomal dominant | Short stature-advanced bone age-early-onset osteoarthritis syndrome,	Not present

**chr15:88856050:C>T**	ACAN	2	0	0	c.3465C>T	p.Thr1155=	Short stature and advanced bone age, with or without early-onset osteoarthritis and/or osteochondritis dissecans, Autosomal dominant | Spondyloepimetaphyseal dysplasia, aggrecan type, Autosomal recessive | Spondyloepiphyseal dysplasia, Kimberley type, Autosomal dominant | Familial osteochondritis dissecans, Autosomal dominant | Short stature-advanced bone age-early-onset osteoarthritis syndrome,	Not present
**chr16:72794366:G>A**	ZFHX3	2	0,006866793	0,0119093	c.8316C>T	p.His2772=	Atrial fibrillation 8, susceptibility to, Autosomal dominant | Prostate cancer, somatic, Autosomal dominant,Somatic mutation | Spinocerebellar ataxia 4, Autosomal dominant | Childhood partial epilepsy and infantile spasms, Autosomal recessive | Syndromic intellectual disability (ZFHX3), Autosomal dominant de-novo	Present
**chr4:76770867:G>A**	SHROOM3	2	0,00082572	0,0204918	c.5591G>A	p.Gly1864Asp	Heterotaxy, Autosomal recessive | Neural Tube Defects, Autosomal dominant	Not present
**chr4:113355252:G>A**	ANK2	2	0,004421821	0,0499904	c.6634G>A	p.Gly2212Ser	Cardiac arrhythmia, ankyrin-B-related, Autosomal dominant | Long QT syndrome 4, Autosomal dominant | Epilepsy, Autosomal dominant de-novo | Romano-Ward syndrome,	Present
**chr4:113264911:A>G**	ANK2	2	0,002854825	0,0136919	c.1401A>G	p.Ala467=	Cardiac arrhythmia, ankyrin-B-related, Autosomal dominant | Long QT syndrome 4, Autosomal dominant | Epilepsy, Autosomal dominant de-novo | Romano-Ward syndrome,	Present
**chr11:1241629:C>T**	MUC5B	2	0,002538899	0,0085027	c.4749C>T	p.Gly1583=	Interstitial lung disease 2, Autosomal dominant | Diffuse panbronchiolitis, | Hypersensitivity pneumonitis, | Idiopathic pulmonary fibrosis,	Not present
**chr16:2100209:C>T**	PKD1	2	0,006308536	0,0389375	c.9669G>A	p.Thr3223=	Polycystic kidney disease 1, Autosomal dominant | Polycystic kidney disease 1 with or without polycystic liver disease, AD | Autosomal dominant polycystic kidney disease, | Autosomal dominant polycystic kidney disease type 1 with tuberous sclerosis,	Present
**chr9:128577336:C>T**	SPTAN1	2	0,002997263	0,0072324	c.931-16C>T	Unknown	Developmental and epileptic encephalopathy 5, Autosomal dominant | Developmental delay with or without epilepsy, Autosomal dominant | Neuronopathy, distal hereditary motor, autosomal dominant 11, Autosomal dominant | Spastic paraplegia 91, autosomal dominant, with or without cerebellar ataxia, Autosomal dominant | Distal myopathy, Autosomal dominant de-novo,Autosomal dominant | Hereditary spastic paraplegia, Autosomal recessive | Spastic Paraplegia and Cerebellar Ataxia, Autosomal dominant,Autosomal dominant de-novo | Neuronopathy, distal hereditary motor, 11, autosomal dominant, AD | Infantile epileptic spasms syndrome,	Not present
**chr4:113358472:T>C**	ANK2	2	0,009577955	0,0340251	c.9854T>C	p.Ile3285Thr	Cardiac arrhythmia, ankyrin-B-related, Autosomal dominant | Long QT syndrome 4, Autosomal dominant | Epilepsy, Autosomal dominant de-novo | Romano-Ward syndrome,	Present
**chr4:76756493:T>A**	SHROOM3	2	0,002405165	0,0181556	c.4754T>A	p.Met1585Lys	Heterotaxy, Autosomal recessive | Neural Tube Defects, Autosomal dominant	Not present
**chr22:50447442:G>A**	SBF1	2	0,001950707	0,00705687	c.5463C>T	p.Tyr1821=	Charcot-Marie-Tooth disease, type 4B3, Autosomal recessive	Present
**chr11:1254776:G>A**	MUC5B	2	0,002744364	0,0190092	c.15560G>A	p.Arg5187His	Interstitial lung disease 2, Autosomal dominant | Diffuse panbronchiolitis, | Hypersensitivity pneumonitis, | Idiopathic pulmonary fibrosis,	Not present

**chr12:132018266:C>T**	EP400	2	0,000613565	0,00368481	c.4167C>T	p.His1389=	Epilepsy with neurodevelopmental disorders, Autosomal recessive	Present
**chr5:150396504:A>G**	TCOF1	2	0,009253132	0,0208098	c.4007A>G	p.Lys1336Arg	Treacher Collins syndrome 1, Autosomal dominant | Treacher-Collins syndrome,	Not present
**chr4:103182849:G>A**	CENPE	2	0,003430321	0,0363492	c.876C>T	p.Leu292=	Microcephaly 13, primary, autosomal recessive, Autosomal recessive | Autosomal recessive primary microcephaly, | Seckel syndrome,	Not present
**chr12:6936728:A>ACAGCAGCAGCAGCAGCAGCAGCAG**	ATN1	2	0,001859111	0,00470655	c.1485_1508dup	p.Gln495_Gln502dup	Congenital hypotonia, epilepsy, developmental delay, and digital anomalies, Autosomal dominant | Dentatorubral-pallidoluysian atrophy, Autosomal dominant | Dentatorubro-pallidoluysian atrophy, AD	Not present
**chr1:237648466:C>T**	RYR2	2	0,004496039	0,0112615	c.7365C>T	p.Asp2455=	Ventricular arrhythmias due to cardiac ryanodine receptor calcium release deficiency syndrome, Autosomal dominant | Ventricular tachycardia, catecholaminergic polymorphic, 1, Autosomal dominant | Primary Generalised Epilepsy (RYR2), Autosomal dominant | Arrhythmogenic right ventricular dysplasia, familial, 2, AD | Ventricular tachycardia, catecholaminergic polymorphic, 1, with or without atrial dysfunction and/or dilated cardomyopathy, AD | Catecholaminergic polymorphic ventricular tachycardia, | Inherited isolated arrhythmogenic cardiomyopathy, dominant-left variant, | Inherited isolated arrhythmogenic cardiomyopathy, dominant-right variant, | Inherited isolated arrhythmogenic ventricular dysplasia, biventricular variant,	Not present
**chr17:77380254:G>INS**	SEPTIN9	2	0	0	Unknown	Unknown	Amyotrophy, hereditary neuralgic, Autosomal dominant | Neuralgic amyotrophy,	Not present
**chr8:100709222:G>DEL**	PABPC1	2	0	0	Unknown	Unknown	Developmental delay, Autosomal dominant de-novo	Present
**chr10:77796543:A>G**	DLG5	2	0,003152191	0,0354617	c.5216T>C	p.Leu1739Pro	Yuksel-Vogel-Bauser syndrome, Autosomal recessive | Congenital Heart Disease, Congenital Anomalies including Ciliopathy Phenotypes, Autosomal dominant,Autosomal recessive	Not present
**chr10:77796547:C>T**	DLG5	2	0,003399436	0,0385689	c.5212G>A	p.Ala1738Thr	Yuksel-Vogel-Bauser syndrome, Autosomal recessive | Congenital Heart Disease, Congenital Anomalies including Ciliopathy Phenotypes, Autosomal dominant,Autosomal recessive	Not present
**chr15:88858594:T>C**	ACAN	2	0,007223823	0,0397612	c.6009T>C	p.Tyr2003=	Short stature and advanced bone age, with or without early-onset osteoarthritis and/or osteochondritis dissecans, Autosomal dominant | Spondyloepimetaphyseal dysplasia, aggrecan type, Autosomal recessive | Spondyloepiphyseal dysplasia, Kimberley type, Autosomal dominant | Familial osteochondritis dissecans, Autosomal dominant | Short stature-advanced bone age-early-onset osteoarthritis syndrome,	Not present
**chr10:77824421:C>T**	DLG5	1	0,002465981	0,0129846	c.2345G>A	p.Arg782His	Yuksel-Vogel-Bauser syndrome, Autosomal recessive | Congenital Heart Disease, Congenital Anomalies including Ciliopathy Phenotypes, Autosomal dominant,Autosomal recessive	Not present
**chr15:63654327:ACAG>A**	HERC1	1	8,48613E-05	0,00141777	c.10085-6_10085-4del	Unknown	Macrocephaly, dysmorphic facies, and psychomotor retardation, Autosomal recessive | Megalencephaly-severe kyphoscoliosis-overgrowth syndrome,	Present
**chr12:132044179:C>T**	EP400	1	0,006797198	0,0266386	c.6453C>T	p.Asp2151=	Epilepsy with neurodevelopmental disorders, Autosomal recessive	Present
**chr16:2109302:G>T**	PKD1	1	3,19202E-05	0,000115827	c.5865C>A	p.His1955Gln	Polycystic kidney disease 1, Autosomal dominant | Polycystic kidney disease 1 with or without polycystic liver disease, AD | Autosomal dominant polycystic	Present

							kidney disease, | Autosomal dominant polycystic kidney disease type 1 with tuberous sclerosis,	
**chr1:109250126:C>CGCT**	CELSR2	1	0,00023322	0,000646128	c.58_60dup	p.Leu25dup	Joubert Syndrome, Autosomal recessive	Not present
**chr4:3074923:AGCAG>A**	HTT	1	0,003254982	0,0102328	c.99_102del	p.Gln33HisfsTer67	Huntington disease, Autosomal dominant | Lopes-Maciel-Rodan syndrome, Autosomal recessive | Lopes-Maciel-Rodan syndrome (LOMARS), AD/AR | Juvenile Huntington disease, | Non-specific syndromic intellectual disability,	Not present
**chr4:3105413:C>T**	HTT	1	0,000518521	0,0018109	c.585C>T	p.His195=	Huntington disease, Autosomal dominant | Lopes-Maciel-Rodan syndrome, Autosomal recessive | Lopes-Maciel-Rodan syndrome (LOMARS), AD/AR | Juvenile Huntington disease, | Non-specific syndromic intellectual disability,	Not present
**chr7:82956442:G>C**	PCLO	1	0,002414487	0,025994	c.4511C>G	p.Thr1504Ser	Pontocerebellar hypoplasia, type 3, Autosomal recessive	Present
**chr7:82952241:C>T**	PCLO	1	0,003542593	0,0380654	c.8712G>A	p.Lys2904=	Pontocerebellar hypoplasia, type 3, Autosomal recessive	Present
**chr22:50454820:G>A**	SBF1	1	8,1976E-05	0,000891918	c.4806C>T	p.Asp1602=	Charcot-Marie-Tooth disease, type 4B3, Autosomal recessive	Present
**chr4:125316935:G>T**	FAT4	1	0,003108573	0,0204918	c.524G>T	p.Arg175Leu	Hennekam lymphangiectasia-lymphedema syndrome 2, Autosomal recessive | Van Maldergem syndrome 2, Autosomal recessive | Cerebrofacioarticular syndrome, | Hennekam syndrome,	Not present
**chr16:2097398:C>T**	PKD1	1	0,000416786	0,00281399	c.10326G>A	p.Ala3442=	Polycystic kidney disease 1, Autosomal dominant | Polycystic kidney disease 1 with or without polycystic liver disease, AD | Autosomal dominant polycystic kidney disease, | Autosomal dominant polycystic kidney disease type 1 with tuberous sclerosis,	Present
**chr7:82902696:C>T**	PCLO	1	3,59991E-05	7,25689E-05	c.13483G>A	p.Ala4495Thr	Pontocerebellar hypoplasia, type 3, Autosomal recessive	Present
**chr4:103145116:T>G**	CENPE	1	0,004905022	0,00851466	c.4791A>C	p.Arg1597Ser	Microcephaly 13, primary, autosomal recessive, Autosomal recessive | Autosomal recessive primary microcephaly, | Seckel syndrome,	Not present
**chr4:113358818:G>C**	ANK2	1	1,02456E-05	3,76336E-05	c.10200G>C	p.Lys3400Asn	Cardiac arrhythmia, ankyrin-B-related, Autosomal dominant | Long QT syndrome 4, Autosomal dominant | Epilepsy, Autosomal dominant de-novo | Romano-Ward syndrome,	Present
**chr6:56529740:T>C**	DST	1	7,85599E-06	2,93953E-05	c.17303A>G	p.His5768Arg	Epidermolysis bullosa simplex 3, localized or generalized intermediate, with bp230 deficiency, Autosomal recessive | Neuropathy, hereditary sensory and autonomic, type VI, Autosomal recessive | Congenital myopathy, Autosomal recessive | Epidermolysis bullosa simplex due to BP230 deficiency, | Hereditary sensory and autonomic neuropathy type 6,	Present
**chr4:103181448:A>G**	CENPE	1	1,74116E-06	3,02312E-06	c.972T>C	p.Ser324=	Microcephaly 13, primary, autosomal recessive, Autosomal recessive | Autosomal recessive primary microcephaly, | Seckel syndrome,	Not present
**chr1:39387189:G>A**	MACF1	1	0,000207553	0,00193776	c.14347G>A	p.Asp4783Asn	Lissencephaly 9 with complex brainstem malformation, Autosomal dominant | Generalised epilepsy, Autosomal recessive,Autosomal dominant de-novo | Posterior-predominant lissencephaly-broad flat pons and medulla-midline crossing defects syndrome,	Present
**chr16:72959452:G>A**	ZFHX3	1	5,8897E-05	9,06399E-05	c.694C>T	p.Arg232Cys	Atrial fibrillation 8, susceptibility to, Autosomal dominant | Prostate cancer, somatic, Autosomal dominant,Somatic mutation | Spinocerebellar ataxia 4, Autosomal dominant | Childhood partial epilepsy and infantile spasms, Autosomal recessive | Syndromic intellectual disability (ZFHX3), Autosomal dominant de-novo	Present
**chr10:77821493:A>C**	DLG5	1	0,004437517	0,00590698	c.2991T>G	p.Pro997=	Yuksel-Vogel-Bauser syndrome, Autosomal recessive | Congenital Heart Disease, Congenital Anomalies	Not present

							including Ciliopathy Phenotypes, Autosomal dominant,Autosomal recessive	
**chr5:150375440:G>A**	TCOF1	1	0,001847098	0,0134144	c.1590G>A	p.Gly530=	Treacher Collins syndrome 1, Autosomal dominant | Treacher-Collins syndrome,	Not present
**chr6:56608118:G>T**	DST	1	0	0	c.6510C>A	p.Pro2170=	Epidermolysis bullosa simplex 3, localized or generalized intermediate, with bp230 deficiency, Autosomal recessive | Neuropathy, hereditary sensory and autonomic, type VI, Autosomal recessive | Congenital myopathy, Autosomal recessive | Epidermolysis bullosa simplex due to BP230 deficiency, | Hereditary sensory and autonomic neuropathy type 6,	Present
**chr7:74700401:C>G**	GTF2I	1	0	0	c.528C>G	p.Asn176Lys	Williams syndrome,	Present
**chr7:82954779:T>C**	PCLO	1	0,006383834	0,00986417	c.6174A>G	p.Leu2058=	Pontocerebellar hypoplasia, type 3, Autosomal recessive	Present
**chr4:3199863:C>T**	HTT	1	0,000760482	0,00696295	c.5500C>T	p.Leu1834=	Huntington disease, Autosomal dominant | Lopes-Maciel-Rodan syndrome, Autosomal recessive | Lopes-Maciel-Rodan syndrome (LOMARS), AD/AR | Juvenile Huntington disease, | Non-specific syndromic intellectual disability,	Not present
**chr1:109252359:C>T**	CELSR2	1	1,11532E-05	3,02261E-05	c.2280C>T	p.Ile760=	Joubert Syndrome, Autosomal recessive	Not present
**chr11:96092210:TTGCTGCTGC TGCTGCTGCTGCTGCTGC>T**	MAML2	1	0,004739377	0,015497	c.1794_1820del	p.Gln613_Gln621del	Mucoepidermoid salivary gland carcinoma,	Not present
**chr4:3211988:T>C**	HTT	1	0,001874299	0,0320275	c.6474T>C	p.Gly2158=	Huntington disease, Autosomal dominant | Lopes-Maciel-Rodan syndrome, Autosomal recessive | Lopes-Maciel-Rodan syndrome (LOMARS), AD/AR | Juvenile Huntington disease, | Non-specific syndromic intellectual disability,	Not present
**chr4:3212138:G>A**	HTT	1	0,003159136	0,032187	c.6624G>A	p.Leu2208=	Huntington disease, Autosomal dominant | Lopes-Maciel-Rodan syndrome, Autosomal recessive | Lopes-Maciel-Rodan syndrome (LOMARS), AD/AR | Juvenile Huntington disease, | Non-specific syndromic intellectual disability,	Not present
**chr4:3224037:G>A**	HTT	1	0,001878068	0,0320721	c.7671G>A	p.Glu2557=	Huntington disease, Autosomal dominant | Lopes-Maciel-Rodan syndrome, Autosomal recessive | Lopes-Maciel-Rodan syndrome (LOMARS), AD/AR | Juvenile Huntington disease, | Non-specific syndromic intellectual disability,	Not present
**chr4:103108863:A>INS**	CENPE	1	0	0	Unknown	Unknown	Microcephaly 13, primary, autosomal recessive, Autosomal recessive | Autosomal recessive primary microcephaly, | Seckel syndrome,	Not present
**chr12:132062551:G>A**	EP400	1	0,00251412	0,0387306	c.8184G>A	p.Gln2728=	Epilepsy with neurodevelopmental disorders, Autosomal recessive	Present
**chr15:63696138:A>INS**	HERC1	1	0	0	Unknown	Unknown	Macrocephaly, dysmorphic facies, and psychomotor retardation, Autosomal recessive | Megalencephaly-severe kyphoscoliosis-overgrowth syndrome,	Present
**chr15:63727727:A>INS**	HERC1	1	0	0	Unknown	Unknown	Macrocephaly, dysmorphic facies, and psychomotor retardation, Autosomal recessive | Megalencephaly-severe kyphoscoliosis-overgrowth syndrome,	Present
**chr12:6935675:C>T**	ATN1	1	0,005120988	0,00828014	c.408C>T	p.Tyr136=	Congenital hypotonia, epilepsy, developmental delay, and digital anomalies, Autosomal dominant | Dentatorubral-pallidoluysian atrophy, Autosomal dominant | Dentatorubro-pallidoluysian atrophy, AD	Not present
**chr1:39453773:A>INS**	MACF1	1	0	0	Unknown	Unknown	Lissencephaly 9 with complex brainstem malformation, Autosomal dominant | Generalised epilepsy, Autosomal recessive,Autosomal dominant de-novo | Posterior-predominant lissencephaly-broad flat pons and medulla-midline crossing defects syndrome,	Present
**chr9:128582730:A>INS**	SPTAN1	1	0	0	Unknown	Unknown	Developmental and epileptic encephalopathy 5, Autosomal dominant | Developmental delay with or without epilepsy, Autosomal dominant | Neuronopathy,	Not present

							distal hereditary motor, autosomal dominant 11, Autosomal dominant | Spastic paraplegia 91, autosomal dominant, with or without cerebellar ataxia, Autosomal dominant | Distal myopathy, Autosomal dominant de-novo,Autosomal dominant | Hereditary spastic paraplegia, Autosomal recessive | Spastic Paraplegia and Cerebellar Ataxia, Autosomal dominant,Autosomal dominant de-novo | Neuronopathy, distal hereditary motor, 11, autosomal dominant, AD | Infantile epileptic spasms syndrome,	
**chr2:169257160:G>C**	LRP2	1	0,00099633	0,00612245	c.2603C>G	p.Thr868Ser	Donnai-Barrow syndrome, Autosomal recessive | Faciooculoacousticorenal syndrome, AR	Present
**chr1:237718469:C>INS**	RYR2	1	0	0	Unknown	Unknown	Ventricular arrhythmias due to cardiac ryanodine receptor calcium release deficiency syndrome, Autosomal dominant | Ventricular tachycardia, catecholaminergic polymorphic, 1, Autosomal dominant | Primary Generalised Epilepsy (RYR2), Autosomal dominant | Arrhythmogenic right ventricular dysplasia, familial, 2, AD | Ventricular tachycardia, catecholaminergic polymorphic, 1, with or without atrial dysfunction and/or dilated cardomyopathy, AD | Catecholaminergic polymorphic ventricular tachycardia, | Inherited isolated arrhythmogenic cardiomyopathy, dominant-left variant, | Inherited isolated arrhythmogenic cardiomyopathy, dominant-right variant, | Inherited isolated arrhythmogenic ventricular dysplasia, biventricular variant,	Not present
**chr1:237778725:C>T**	RYR2	1	0,002961365	0,0243022	c.11835C>T	p.Val3945=	Ventricular arrhythmias due to cardiac ryanodine receptor calcium release deficiency syndrome, Autosomal dominant | Ventricular tachycardia, catecholaminergic polymorphic, 1, Autosomal dominant | Primary Generalised Epilepsy (RYR2), Autosomal dominant | Arrhythmogenic right ventricular dysplasia, familial, 2, AD | Ventricular tachycardia, catecholaminergic polymorphic, 1, with or without atrial dysfunction and/or dilated cardomyopathy, AD | Catecholaminergic polymorphic ventricular tachycardia, | Inherited isolated arrhythmogenic cardiomyopathy, dominant-left variant, | Inherited isolated arrhythmogenic cardiomyopathy, dominant-right variant, | Inherited isolated arrhythmogenic ventricular dysplasia, biventricular variant,	Not present
**chr6:56648591:A>C**	DST	1	4,51842E-05	8,68851E-05	c.1533T>G	p.Pro511=	Epidermolysis bullosa simplex 3, localized or generalized intermediate, with bp230 deficiency, Autosomal recessive | Neuropathy, hereditary sensory and autonomic, type VI, Autosomal recessive | Congenital myopathy, Autosomal recessive | Epidermolysis bullosa simplex due to BP230 deficiency, | Hereditary sensory and autonomic neuropathy type 6,	Present
**chr15:88845689:G>A**	ACAN	1	0,007807338	0,013245	c.1236G>A	p.Leu412=	Short stature and advanced bone age, with or without early-onset osteoarthritis and/or osteochondritis dissecans, Autosomal dominant | Spondyloepimetaphyseal dysplasia, aggrecan type, Autosomal recessive | Spondyloepiphyseal dysplasia, Kimberley type, Autosomal dominant | Familial osteochondritis dissecans, Autosomal dominant | Short stature-advanced bone age-early-onset osteoarthritis syndrome,	Not present
**chr10:77817068:C>T**	DLG5	1	0,003595179	0,0196854	c.3813G>A	p.Ala1271=	Yuksel-Vogel-Bauser syndrome, Autosomal recessive | Congenital Heart Disease, Congenital Anomalies	Not present

							including Ciliopathy Phenotypes, Autosomal dominant,Autosomal recessive	
**chr10:77856873:C>T**	DLG5	1	0,004876927	0,0294355	c.393G>A	p.Pro131=	Yuksel-Vogel-Bauser syndrome, Autosomal recessive | Congenital Heart Disease, Congenital Anomalies including Ciliopathy Phenotypes, Autosomal dominant,Autosomal recessive	Not present
**chr4:125490370:G>A**	FAT4	1	0,002906441	0,0113308	c.13554G>A	p.Leu4518=	Hennekam lymphangiectasia-lymphedema syndrome 2, Autosomal recessive | Van Maldergem syndrome 2, Autosomal recessive | Cerebrofacioarticular syndrome, | Hennekam syndrome,	Not present
**chr7:152177496:G>C**	KMT2C	1	0,007676491	0,0205103	c.7957C>G	p.Leu2653Val	Kleefstra syndrome 2, Autosomal dominant | Neurodevelopmental disorder distinct from Kleefstra and Kabuki syndromes, Autosomal dominant,Autosomal dominant de-novo | Kleefstra syndrome due to a point mutation,	Present
**chr7:152177962:G>A**	KMT2C	1	0,00779102	0,0239288	c.7491C>T	p.Arg2497=	Kleefstra syndrome 2, Autosomal dominant | Neurodevelopmental disorder distinct from Kleefstra and Kabuki syndromes, Autosomal dominant,Autosomal dominant de-novo | Kleefstra syndrome due to a point mutation,	Present
**chr10:77796591:G>A**	DLG5	1	1,40914E-05	7,16456E-05	c.5168C>T	p.Ser1723Leu	Yuksel-Vogel-Bauser syndrome, Autosomal recessive | Congenital Heart Disease, Congenital Anomalies including Ciliopathy Phenotypes, Autosomal dominant,Autosomal recessive	Not present
**chr4:125318213:A>G**	FAT4	1	8,32164E-05	0,00047259	c.1802A>G	p.Glu601Gly	Hennekam lymphangiectasia-lymphedema syndrome 2, Autosomal recessive | Van Maldergem syndrome 2, Autosomal recessive | Cerebrofacioarticular syndrome, | Hennekam syndrome,	Not present
**chr1:237441447:C>T**	RYR2	1	0,000790777	0,00129412	c.1134C>T	p.Asp378=	Ventricular arrhythmias due to cardiac ryanodine receptor calcium release deficiency syndrome, Autosomal dominant | Ventricular tachycardia, catecholaminergic polymorphic, 1, Autosomal dominant | Primary Generalised Epilepsy (RYR2), Autosomal dominant | Arrhythmogenic right ventricular dysplasia, familial, 2, AD | Ventricular tachycardia, catecholaminergic polymorphic, 1, with or without atrial dysfunction and/or dilated cardomyopathy, AD | Catecholaminergic polymorphic ventricular tachycardia, | Inherited isolated arrhythmogenic cardiomyopathy, dominant-left variant, | Inherited isolated arrhythmogenic cardiomyopathy, dominant-right variant, | Inherited isolated arrhythmogenic ventricular dysplasia, biventricular variant,	Not present
**chr1:237723192:A>G**	RYR2	1	0	0	c.10619A>G	p.Asp3540Gly	Ventricular arrhythmias due to cardiac ryanodine receptor calcium release deficiency syndrome, Autosomal dominant | Ventricular tachycardia, catecholaminergic polymorphic, 1, Autosomal dominant | Primary Generalised Epilepsy (RYR2), Autosomal dominant | Arrhythmogenic right ventricular dysplasia, familial, 2, AD | Ventricular tachycardia, catecholaminergic polymorphic, 1, with or without atrial dysfunction and/or dilated cardomyopathy, AD | Catecholaminergic polymorphic ventricular tachycardia, | Inherited isolated arrhythmogenic cardiomyopathy, dominant-left variant, | Inherited isolated arrhythmogenic cardiomyopathy, dominant-right variant, | Inherited isolated arrhythmogenic ventricular dysplasia, biventricular variant,	Not present
**chr22:50454681:G>A**	SBF1	1	8,63884E-05	0,000613155	c.4874C>T	p.Thr1625Met	Charcot-Marie-Tooth disease, type 4B3, Autosomal recessive	Present

**chr4:3115406:A>G**	HTT	1	4,86493E-05	0,00192864	c.850A>G	p.Thr284Ala	Huntington disease, Autosomal dominant | Lopes-Maciel-Rodan syndrome, Autosomal recessive | Lopes-Maciel-Rodan syndrome (LOMARS), AD/AR | Juvenile Huntington disease, | Non-specific syndromic intellectual disability,	Not present
**chr1:109270029:C>G**	CELSR2	1	7,68327E-06	2,93988E-05	c.7204C>G	p.Arg2402Gly	Joubert Syndrome, Autosomal recessive	Not present
**chr4:3130311:A>G**	HTT	1	0,005279031	0,0183358	c.1874A>G	p.Gln625Arg	Huntington disease, Autosomal dominant | Lopes-Maciel-Rodan syndrome, Autosomal recessive | Lopes-Maciel-Rodan syndrome (LOMARS), AD/AR | Juvenile Huntington disease, | Non-specific syndromic intellectual disability,	Not present
**chr4:3235649:C>T**	HTT	1	0,002095346	0,0123427	c.8656C>T	p.Leu2886=	Huntington disease, Autosomal dominant | Lopes-Maciel-Rodan syndrome, Autosomal recessive | Lopes-Maciel-Rodan syndrome (LOMARS), AD/AR | Juvenile Huntington disease, | Non-specific syndromic intellectual disability,	Not present
**chr4:3238940:C>T**	HTT	1	0,006243099	0,0182565	c.9177C>T	p.Phe3059=	Huntington disease, Autosomal dominant | Lopes-Maciel-Rodan syndrome, Autosomal recessive | Lopes-Maciel-Rodan syndrome (LOMARS), AD/AR | Juvenile Huntington disease, | Non-specific syndromic intellectual disability,	Not present
**chr4:76740408:G>A**	SHROOM3	1	1,66708E-05	4,41176E-05	c.2235G>A	p.Pro745=	Heterotaxy, Autosomal recessive | Neural Tube Defects, Autosomal dominant	Not present
**chr4:103151254:T>C**	CENPE	1	1,62205E-06	2,87778E-06	c.3361A>G	p.Arg1121Gly	Microcephaly 13, primary, autosomal recessive, Autosomal recessive | Autosomal recessive primary microcephaly, | Seckel syndrome,	Not present
**chr1:237798063:C>T**	RYR2	1	0,002611043	0,00391345	c.13983C>T	p.Tyr4661=	Ventricular arrhythmias due to cardiac ryanodine receptor calcium release deficiency syndrome, Autosomal dominant | Ventricular tachycardia, catecholaminergic polymorphic, 1, Autosomal dominant | Primary Generalised Epilepsy (RYR2), Autosomal dominant | Arrhythmogenic right ventricular dysplasia, familial, 2, AD | Ventricular tachycardia, catecholaminergic polymorphic, 1, with or without atrial dysfunction and/or dilated cardomyopathy, AD | Catecholaminergic polymorphic ventricular tachycardia, | Inherited isolated arrhythmogenic cardiomyopathy, dominant-left variant, | Inherited isolated arrhythmogenic cardiomyopathy, dominant-right variant, | Inherited isolated arrhythmogenic ventricular dysplasia, biventricular variant,	Not present
**chr1:39412111:T>C**	MACF1	1	0,000731337	0,00930031	c.1846T>C	p.Leu616=	Lissencephaly 9 with complex brainstem malformation, Autosomal dominant | Generalised epilepsy, Autosomal recessive,Autosomal dominant de-novo | Posterior-predominant lissencephaly-broad flat pons and medulla-midline crossing defects syndrome,	Present
**chr12:6936728:A>ACAGCAGCAGCAGCAGCAGCAGCAGCAGCAGCAG**	ATN1	1	0,00020555	0,000677201	c.1476_1508dup	p.Gln492_Gln502dup	Congenital hypotonia, epilepsy, developmental delay, and digital anomalies, Autosomal dominant | Dentatorubral-pallidoluysian atrophy, Autosomal dominant | Dentatorubro-pallidoluysian atrophy, AD	Not present
**chr16:2093545:C>T**	PKD1	1	0,000545589	0,00125188	c.11015G>A	p.Arg3672Gln	Polycystic kidney disease 1, Autosomal dominant | Polycystic kidney disease 1 with or without polycystic liver disease, AD | Autosomal dominant polycystic kidney disease, | Autosomal dominant polycystic kidney disease type 1 with tuberous sclerosis,	Present
**chr15:63625971:C>T**	HERC1	1	4,82725E-05	0,000196284	c.13275+14G>A	Unknown	Macrocephaly, dysmorphic facies, and psychomotor retardation, Autosomal recessive | Megalencephaly-severe kyphoscoliosis-overgrowth syndrome,	Present

**chr1:39410749:A>T**	MACF1	1	0,00327585	0,0396745	c.484A>T	p.Asn162Tyr	Lissencephaly 9 with complex brainstem malformation, Autosomal dominant | Generalised epilepsy, Autosomal recessive,Autosomal dominant de-novo | Posterior-predominant lissencephaly-broad flat pons and medulla-midline crossing defects syndrome,	Present
**chr5:83540422:C>T**	VCAN	1	0,00785162	0,0340008	c.7419C>T	p.Ser2473=	Wagner syndrome 1, Autosomal dominant | Wagner vitreoretinopathy, AD | Wagner disease,	Not present
**chr6:7230496:C>T**	RREB1	1	0,00167624	0,0101471	c.2397C>T	p.Phe799=	Noonan-like RASopathy (RREB1), Autosomal dominant | RASopathy Syndrome of Congenital Heart Disease, Genitourinary Malformations, and Developmental Delay, Autosomal dominant de-novo,Autosomal dominant | 22q11.2 deletion syndrome,	Not present
**chr11:1251567:G>T**	MUC5B	1	0,007020043	0,0187102	c.14687G>T	p.Ser4896Ile	Interstitial lung disease 2, Autosomal dominant | Diffuse panbronchiolitis, | Hypersensitivity pneumonitis, | Idiopathic pulmonary fibrosis,	Not present
**chr12:132044823:G>A**	EP400	1	0,004828747	0,0193586	c.6654G>A	p.Pro2218=	Epilepsy with neurodevelopmental disorders, Autosomal recessive	Present
**chr12:132066893:C>T**	EP400	1	0,000147378	0,000654108	c.8673C>T	p.Ser2891=	Epilepsy with neurodevelopmental disorders, Autosomal recessive	Present
**chr7:82951497:T>C**	PCLO	1	0,002938303	0,0305037	c.9098-7A>G	Unknown	Pontocerebellar hypoplasia, type 3, Autosomal recessive	Present
**chr7:83135608:C>T**	PCLO	1	0,001887515	0,019254	c.1942G>A	p.Gly648Arg	Pontocerebellar hypoplasia, type 3, Autosomal recessive	Present
**chr16:2089874:G>A**	PKD1	1	0,004412302	0,00783455	c.12765C>T	p.Pro4255=	Polycystic kidney disease 1, Autosomal dominant | Polycystic kidney disease 1 with or without polycystic liver disease, AD | Autosomal dominant polycystic kidney disease, | Autosomal dominant polycystic kidney disease type 1 with tuberous sclerosis,	Present
**chr22:50466657:G>A**	SBF1	1	0,002229046	0,0118418	c.603C>T	p.Ala201=	Charcot-Marie-Tooth disease, type 4B3, Autosomal recessive	Present
**chr16:72787694:AGCCGCCGCCGCCGCCGCCGCCGCCACCGCCGCCGCCGCCGCCACTGCCACC>A**	ZFHX3	1	0,000138715	0,000806614	c.10531_10581del	p.Gly3511_Gly3527del	Atrial fibrillation 8, susceptibility to, Autosomal dominant | Prostate cancer, somatic, Autosomal dominant,Somatic mutation | Spinocerebellar ataxia 4, Autosomal dominant | Childhood partial epilepsy and infantile spasms, Autosomal recessive | Syndromic intellectual disability (ZFHX3), Autosomal dominant de-novo	Present
**chr12:131982328:G>A**	EP400	1	0,002573663	0,0300929	c.1779G>A	p.Pro593=	Epilepsy with neurodevelopmental disorders, Autosomal recessive	Present
**chr12:132038100:AGAATACATT>A**	EP400	1	0,002460727	0,029324	c.6207+9_6207+17del	Unknown	Epilepsy with neurodevelopmental disorders, Autosomal recessive	Present
**chr12:132055000:T>G**	EP400	1	0,004220868	0,0479277	c.7755T>G	p.Thr2585=	Epilepsy with neurodevelopmental disorders, Autosomal recessive	Present
**chr6:32024331:C>T**	C4B	1	0	0	c.1341+7C>T	Unknown	C4B deficiency, | Complement component 4B deficiency, AR | Immunodeficiency due to a classical component pathway complement deficiency, | Systemic lupus erythematosus,	Present
**chr6:56594038:A>C**	DST	1	0,004074976	0,0431415	c.12351T>G	p.Thr4117=	Epidermolysis bullosa simplex 3, localized or generalized intermediate, with bp230 deficiency, Autosomal recessive | Neuropathy, hereditary sensory and autonomic, type VI, Autosomal recessive | Congenital myopathy, Autosomal recessive | Epidermolysis bullosa simplex due to BP230 deficiency, | Hereditary sensory and autonomic neuropathy type 6,	Present
**chr6:56608121:T>C**	DST	1	0,004108441	0,0436185	c.6507A>G	p.Gln2169=	Epidermolysis bullosa simplex 3, localized or generalized intermediate, with bp230 deficiency, Autosomal recessive | Neuropathy, hereditary sensory and autonomic, type VI, Autosomal recessive | Congenital myopathy, Autosomal recessive | Epidermolysis bullosa simplex due to BP230 deficiency, | Hereditary sensory and autonomic neuropathy type 6,	Present

**chr9:128632799:C>T**	SPTAN1	1	0,000367495	0,00189215	c.7161-9C>T	Unknown	Developmental and epileptic encephalopathy 5, Autosomal dominant | Developmental delay with or without epilepsy, Autosomal dominant | Neuronopathy, distal hereditary motor, autosomal dominant 11, Autosomal dominant | Spastic paraplegia 91, autosomal dominant, with or without cerebellar ataxia, Autosomal dominant | Distal myopathy, Autosomal dominant de-novo,Autosomal dominant | Hereditary spastic paraplegia, Autosomal recessive | Spastic Paraplegia and Cerebellar Ataxia, Autosomal dominant,Autosomal dominant de-novo | Neuronopathy, distal hereditary motor, 11, autosomal dominant, AD | Infantile epileptic spasms syndrome,	Not present
**chr22:50455597:G>A**	SBF1	1	0,002138911	0,02231	c.4267-15C>T	Unknown	Charcot-Marie-Tooth disease, type 4B3, Autosomal recessive	Present
**chr5:150393480:G>A**	TCOF1	1	0,001115225	0,00672544	c.3712G>A	p.Ala1238Thr	Treacher Collins syndrome 1, Autosomal dominant | Treacher-Collins syndrome,	Not present
**chr1:39387313:C>T**	MACF1	1	0,000203572	0,00144648	c.14471C>T	p.Ala4824Val	Lissencephaly 9 with complex brainstem malformation, Autosomal dominant | Generalised epilepsy, Autosomal recessive,Autosomal dominant de-novo | Posterior-predominant lissencephaly-broad flat pons and medulla-midline crossing defects syndrome,	Present
**chr16:72794126:G>A**	ZFHX3	1	1,66459E-05	3,02133E-05	c.8556C>T	p.Asn2852=	Atrial fibrillation 8, susceptibility to, Autosomal dominant | Prostate cancer, somatic, Autosomal dominant,Somatic mutation | Spinocerebellar ataxia 4, Autosomal dominant | Childhood partial epilepsy and infantile spasms, Autosomal recessive | Syndromic intellectual disability (ZFHX3), Autosomal dominant de-novo	Present
**chr16:72959699:C>T**	ZFHX3	1	0	0	c.447G>A	p.Gln149=	Atrial fibrillation 8, susceptibility to, Autosomal dominant | Prostate cancer, somatic, Autosomal dominant,Somatic mutation | Spinocerebellar ataxia 4, Autosomal dominant | Childhood partial epilepsy and infantile spasms, Autosomal recessive | Syndromic intellectual disability (ZFHX3), Autosomal dominant de-novo	Present
**chr4:3230031:G>A**	HTT	1	1,66469E-05	0,000144732	c.8254G>A	p.Val2752Ile	Huntington disease, Autosomal dominant | Lopes-Maciel-Rodan syndrome, Autosomal recessive | Lopes-Maciel-Rodan syndrome (LOMARS), AD/AR | Juvenile Huntington disease, | Non-specific syndromic intellectual disability,	Not present
**chr15:63669656:T>C**	HERC1	1	0,00622125	0,0104503	c.8088A>G	p.Thr2696=	Macrocephaly, dysmorphic facies, and psychomotor retardation, Autosomal recessive | Megalencephaly-severe kyphoscoliosis-overgrowth syndrome,	Present
**chr16:2109590:A>G**	PKD1	1	0,000295526	0,00236518	c.5577T>C	p.Ala1859=	Polycystic kidney disease 1, Autosomal dominant | Polycystic kidney disease 1 with or without polycystic liver disease, AD | Autosomal dominant polycystic kidney disease, | Autosomal dominant polycystic kidney disease type 1 with tuberous sclerosis,	Present
**chr16:2111232:C>T**	PKD1	1	0,00257997	0,0298278	c.3935G>A	p.Arg1312Gln	Polycystic kidney disease 1, Autosomal dominant | Polycystic kidney disease 1 with or without polycystic liver disease, AD | Autosomal dominant polycystic kidney disease, | Autosomal dominant polycystic kidney disease type 1 with tuberous sclerosis,	Present
**chr1:237792281:G>A**	RYR2	1	0,00103318	0,011262	c.13740G>A	p.Thr4580=	Ventricular arrhythmias due to cardiac ryanodine receptor calcium release deficiency syndrome, Autosomal dominant | Ventricular tachycardia, catecholaminergic polymorphic, 1, Autosomal dominant | Primary Generalised Epilepsy (RYR2), Autosomal dominant |	Not present

							Arrhythmogenic right ventricular dysplasia, familial, 2, AD | Ventricular tachycardia, catecholaminergic polymorphic, 1, with or without atrial dysfunction and/or dilated cardomyopathy, AD | Catecholaminergic polymorphic ventricular tachycardia, | Inherited isolated arrhythmogenic cardiomyopathy, dominant-left variant, | Inherited isolated arrhythmogenic cardiomyopathy, dominant-right variant, | Inherited isolated arrhythmogenic ventricular dysplasia, biventricular variant,	
**chr4:125317150:C>A**	FAT4	1	0,004548951	0,0088854	c.739C>A	p.Pro247Thr	Hennekam lymphangiectasia-lymphedema syndrome 2, Autosomal recessive | Van Maldergem syndrome 2, Autosomal recessive | Cerebrofacioarticular syndrome, | Hennekam syndrome,	Not present
**chr1:237798057:G>A**	RYR2	1	0,002838331	0,0324241	c.13977G>A	p.Glu4659=	Ventricular arrhythmias due to cardiac ryanodine receptor calcium release deficiency syndrome, Autosomal dominant | Ventricular tachycardia, catecholaminergic polymorphic, 1, Autosomal dominant | Primary Generalised Epilepsy (RYR2), Autosomal dominant | Arrhythmogenic right ventricular dysplasia, familial, 2, AD | Ventricular tachycardia, catecholaminergic polymorphic, 1, with or without atrial dysfunction and/or dilated cardomyopathy, AD | Catecholaminergic polymorphic ventricular tachycardia, | Inherited isolated arrhythmogenic cardiomyopathy, dominant-left variant, | Inherited isolated arrhythmogenic cardiomyopathy, dominant-right variant, | Inherited isolated arrhythmogenic ventricular dysplasia, biventricular variant,	Not present
**chr1:237503283:C>T**	RYR2	1	0,004002232	0,0322977	c.2397-6C>T	Unknown	Ventricular arrhythmias due to cardiac ryanodine receptor calcium release deficiency syndrome, Autosomal dominant | Ventricular tachycardia, catecholaminergic polymorphic, 1, Autosomal dominant | Primary Generalised Epilepsy (RYR2), Autosomal dominant | Arrhythmogenic right ventricular dysplasia, familial, 2, AD | Ventricular tachycardia, catecholaminergic polymorphic, 1, with or without atrial dysfunction and/or dilated cardomyopathy, AD | Catecholaminergic polymorphic ventricular tachycardia, | Inherited isolated arrhythmogenic cardiomyopathy, dominant-left variant, | Inherited isolated arrhythmogenic cardiomyopathy, dominant-right variant, | Inherited isolated arrhythmogenic ventricular dysplasia, biventricular variant,	Not present
**chr1:237792141:C>T**	RYR2	1	3,05577E-05	9,36944E-05	c.13600C>T	p.Pro4534Ser	Ventricular arrhythmias due to cardiac ryanodine receptor calcium release deficiency syndrome, Autosomal dominant | Ventricular tachycardia, catecholaminergic polymorphic, 1, Autosomal dominant | Primary Generalised Epilepsy (RYR2), Autosomal dominant | Arrhythmogenic right ventricular dysplasia, familial, 2, AD | Ventricular tachycardia, catecholaminergic polymorphic, 1, with or without atrial dysfunction and/or dilated cardomyopathy, AD | Catecholaminergic polymorphic ventricular tachycardia, | Inherited isolated arrhythmogenic cardiomyopathy, dominant-left variant, | Inherited isolated arrhythmogenic cardiomyopathy, dominant-right variant, | Inherited isolated arrhythmogenic ventricular dysplasia, biventricular variant,	Not present

**chr4:113360843:C>T**	ANK2	1	0,00013982	0,000724638	c.10702C>T	p.Arg3568Trp	Cardiac arrhythmia, ankyrin-B-related, Autosomal dominant | Long QT syndrome 4, Autosomal dominant | Epilepsy, Autosomal dominant de-novo | Romano-Ward syndrome,	Present
**chr1:109271497:C>G**	CELSR2	1	0,000106278	0,00144648	c.7788C>G	p.Thr2596=	Joubert Syndrome, Autosomal recessive	Not present
**chr1:109251694:C>T**	CELSR2	1	0,000467359	0,00094697	c.1615C>T	p.Arg539Cys	Joubert Syndrome, Autosomal recessive	Not present
**chr6:56487154:C>T**	DST	1	0,008196406	0,0123765	c.20997G>A	p.Leu6999=	Epidermolysis bullosa simplex 3, localized or generalized intermediate, with bp230 deficiency, Autosomal recessive | Neuropathy, hereditary sensory and autonomic, type VI, Autosomal recessive | Congenital myopathy, Autosomal recessive | Epidermolysis bullosa simplex due to BP230 deficiency, | Hereditary sensory and autonomic neuropathy type 6,	Present
**chr7:83162337:G>T**	PCLO	1	0,000589143	0,00278674	c.248+8C>A	Unknown	Pontocerebellar hypoplasia, type 3, Autosomal recessive	Present
**chr7:152181521:T>G**	KMT2C	1	0,007174705	0,0116922	c.6339A>C	p.Ser2113=	Kleefstra syndrome 2, Autosomal dominant | Neurodevelopmental disorder distinct from Kleefstra and Kabuki syndromes, Autosomal dominant,Autosomal dominant de-novo | Kleefstra syndrome due to a point mutation,	Present
**chr5:83520356:A>G**	VCAN	1	2,5657E-05	0,00094697	c.2050A>G	p.Arg684Gly	Wagner syndrome 1, Autosomal dominant | Wagner vitreoretinopathy, AD | Wagner disease,	Not present
**chr10:77828904:G>A**	DLG5	1	6,01817E-05	0,000621118	c.2267C>T	p.Ala756Val	Yuksel-Vogel-Bauser syndrome, Autosomal recessive | Congenital Heart Disease, Congenital Anomalies including Ciliopathy Phenotypes, Autosomal dominant,Autosomal recessive	Not present
**chr11:1250017:G>A**	MUC5B	1	0,00210536	0,0111875	c.13137G>A	p.Thr4379=	Interstitial lung disease 2, Autosomal dominant | Diffuse panbronchiolitis, | Hypersensitivity pneumonitis, | Idiopathic pulmonary fibrosis,	Not present
**chr11:1250169:C>T**	MUC5B	1	0,000110391	0,000728509	c.13289C>T	p.Thr4430Met	Interstitial lung disease 2, Autosomal dominant | Diffuse panbronchiolitis, | Hypersensitivity pneumonitis, | Idiopathic pulmonary fibrosis,	Not present
**chr15:63686389:T>C**	HERC1	1	0,000996113	0,00165284	c.6195A>G	p.Thr2065=	Macrocephaly, dysmorphic facies, and psychomotor retardation, Autosomal recessive | Megalencephaly-severe kyphoscoliosis-overgrowth syndrome,	Present
**chr16:2111149:G>A**	PKD1	1	0,003012861	0,00442395	c.4018C>T	p.Arg1340Trp	Polycystic kidney disease 1, Autosomal dominant | Polycystic kidney disease 1 with or without polycystic liver disease, AD | Autosomal dominant polycystic kidney disease, | Autosomal dominant polycystic kidney disease type 1 with tuberous sclerosis,	Present
**chr17:77402183:C>T**	SEPTIN9	1	0,002341548	0,00336745	c.201C>T	p.Gly67=	Amyotrophy, hereditary neuralgic, Autosomal dominant | Neuralgic amyotrophy,	Not present
**chr4:113369717:C>T**	ANK2	1	4,22627E-05	0,000343849	c.11522C>T	p.Pro3841Leu	Cardiac arrhythmia, ankyrin-B-related, Autosomal dominant | Long QT syndrome 4, Autosomal dominant | Epilepsy, Autosomal dominant de-novo | Romano-Ward syndrome,	Present
**chr1:39317341:G>T**	MACF1	1	0,006198427	0,0379245	c.3716G>T	p.Arg1239Leu	Lissencephaly 9 with complex brainstem malformation, Autosomal dominant | Generalised epilepsy, Autosomal recessive,Autosomal dominant de-novo | Posterior-predominant lissencephaly-broad flat pons and medulla-midline crossing defects syndrome,	Present
**chr1:39333159:C>T**	MACF1	1	0,005728706	0,0377572	c.6571C>T	p.His2191Tyr	Lissencephaly 9 with complex brainstem malformation, Autosomal dominant | Generalised epilepsy, Autosomal recessive,Autosomal dominant de-novo | Posterior-predominant lissencephaly-broad flat pons and medulla-midline crossing defects syndrome,	Present
**chr1:39411381:G>A**	MACF1	1	0,001505939	0,00461361	c.1116G>A	p.Val372=	Lissencephaly 9 with complex brainstem malformation, Autosomal dominant | Generalised epilepsy, Autosomal recessive,Autosomal dominant de-novo | Posterior-	Present

							predominant lissencephaly-broad flat pons and medulla-midline crossing defects syndrome,	
**chr15:63652489:T>G**	HERC1	1	4,80454E-06	8,62843E-06	c.10343A>C	p.Asn3448Thr	Macrocephaly, dysmorphic facies, and psychomotor retardation, Autosomal recessive | Megalencephaly-severe kyphoscoliosis-overgrowth syndrome,	Present
**chr15:88858209:G>A**	ACAN	1	6,40415E-06	9,07057E-05	c.5624G>A	p.Ser1875Asn	Short stature and advanced bone age, with or without early-onset osteoarthritis and/or osteochondritis dissecans, Autosomal dominant | Spondyloepimetaphyseal dysplasia, aggrecan type, Autosomal recessive | Spondyloepiphyseal dysplasia, Kimberley type, Autosomal dominant | Familial osteochondritis dissecans, Autosomal dominant | Short stature-advanced bone age-early-onset osteoarthritis syndrome,	Not present
**chr4:76741336:G>A**	SHROOM3	1	0,000113206	0,00100978	c.3163G>A	p.Ala1055Thr	Heterotaxy, Autosomal recessive | Neural Tube Defects, Autosomal dominant	Not present
**chr4:125491128:G>A**	FAT4	1	5,76184E-05	0,00183007	c.14312G>A	p.Arg4771His	Hennekam lymphangiectasia-lymphedema syndrome 2, Autosomal recessive | Van Maldergem syndrome 2, Autosomal recessive | Cerebrofacioarticular syndrome, | Hennekam syndrome,	Not present
**chr4:3125535:A>G**	HTT	1	0,000402398	0,00345622	c.1322-14A>G	Unknown	Huntington disease, Autosomal dominant | Lopes-Maciel-Rodan syndrome, Autosomal recessive | Lopes-Maciel-Rodan syndrome (LOMARS), AD/AR | Juvenile Huntington disease, | Non-specific syndromic intellectual disability,	Not present
**chr4:3121242:G>C**	HTT	1	0,002367447	0,0239629	c.1083G>C	p.Thr361=	Huntington disease, Autosomal dominant | Lopes-Maciel-Rodan syndrome, Autosomal recessive | Lopes-Maciel-Rodan syndrome (LOMARS), AD/AR | Juvenile Huntington disease, | Non-specific syndromic intellectual disability,	Not present
**chr2:169294717:TAAAAAA>T**	LRP2	1	0,008421704	0,0132367	c.428-13_428-8del	Unknown	Donnai-Barrow syndrome, Autosomal recessive | Faciooculoacousticorenal syndrome, AR	Present
**chr1:237496595:A>G**	RYR2	1	0,002029735	0,0226065	c.2046A>G	p.Thr682=	Ventricular arrhythmias due to cardiac ryanodine receptor calcium release deficiency syndrome, Autosomal dominant | Ventricular tachycardia, catecholaminergic polymorphic, 1, Autosomal dominant | Primary Generalised Epilepsy (RYR2), Autosomal dominant | Arrhythmogenic right ventricular dysplasia, familial, 2, AD | Ventricular tachycardia, catecholaminergic polymorphic, 1, with or without atrial dysfunction and/or dilated cardomyopathy, AD | Catecholaminergic polymorphic ventricular tachycardia, | Inherited isolated arrhythmogenic cardiomyopathy, dominant-left variant, | Inherited isolated arrhythmogenic cardiomyopathy, dominant-right variant, | Inherited isolated arrhythmogenic ventricular dysplasia, biventricular variant,	Not present
**chr16:72797656:T>C**	ZFHX3	1	0,002162424	0,0147724	c.5026A>G	p.Ser1676Gly	Atrial fibrillation 8, susceptibility to, Autosomal dominant | Prostate cancer, somatic, Autosomal dominant,Somatic mutation | Spinocerebellar ataxia 4, Autosomal dominant | Childhood partial epilepsy and infantile spasms, Autosomal recessive | Syndromic intellectual disability (ZFHX3), Autosomal dominant de-novo	Present
**chr16:72950840:C>T**	ZFHX3	1	0,001855223	0,0185374	c.2845G>A	p.Val949Ile	Atrial fibrillation 8, susceptibility to, Autosomal dominant | Prostate cancer, somatic, Autosomal dominant,Somatic mutation | Spinocerebellar ataxia 4, Autosomal dominant | Childhood partial epilepsy and infantile spasms, Autosomal recessive | Syndromic	Present

							intellectual disability (ZFHX3), Autosomal dominant de-novo	
**chr11:1246353:C>A**	MUC5B	1	0,000329201	0,00376454	c.9473C>A	p.Pro3158Gln	Interstitial lung disease 2, Autosomal dominant | Diffuse panbronchiolitis, | Hypersensitivity pneumonitis, | Idiopathic pulmonary fibrosis,	Not present
**chr10:77792448:G>A**	DLG5	1	0,00034694	0,00313404	c.5752C>T	p.Pro1918Ser	Yuksel-Vogel-Bauser syndrome, Autosomal recessive | Congenital Heart Disease, Congenital Anomalies including Ciliopathy Phenotypes, Autosomal dominant,Autosomal recessive	Not present
**chr11:1248395:A>G**	MUC5B	1	0,000722753	0,00231803	c.11515A>G	p.Thr3839Ala	Interstitial lung disease 2, Autosomal dominant | Diffuse panbronchiolitis, | Hypersensitivity pneumonitis, | Idiopathic pulmonary fibrosis,	Not present
**chr4:125414950:A>G**	FAT4	1	0,001419123	0,00804658	c.5987A>G	p.Lys1996Arg	Hennekam lymphangiectasia-lymphedema syndrome 2, Autosomal recessive | Van Maldergem syndrome 2, Autosomal recessive | Cerebrofacioarticular syndrome, | Hennekam syndrome,	Not present
**chr4:125446451:G>T**	FAT4	1	0,001652876	0,00851566	c.7358G>T	p.Ser2453Ile	Hennekam lymphangiectasia-lymphedema syndrome 2, Autosomal recessive | Van Maldergem syndrome 2, Autosomal recessive | Cerebrofacioarticular syndrome, | Hennekam syndrome,	Not present
**chr4:125448945:C>T**	FAT4	1	0,001429875	0,00812902	c.7935C>T	p.Asp2645=	Hennekam lymphangiectasia-lymphedema syndrome 2, Autosomal recessive | Van Maldergem syndrome 2, Autosomal recessive | Cerebrofacioarticular syndrome, | Hennekam syndrome,	Not present
**chr4:125479767:C>T**	FAT4	1	0,002798712	0,019741	c.12506C>T	p.Thr4169Ile	Hennekam lymphangiectasia-lymphedema syndrome 2, Autosomal recessive | Van Maldergem syndrome 2, Autosomal recessive | Cerebrofacioarticular syndrome, | Hennekam syndrome,	Not present
**chr6:7229123:G>A**	RREB1	1	0,003140001	0,0264415	c.1024G>A	p.Asp342Asn	Noonan-like RASopathy (RREB1), Autosomal dominant | RASopathy Syndrome of Congenital Heart Disease, Genitourinary Malformations, and Developmental Delay, Autosomal dominant de-novo,Autosomal dominant | 22q11.2 deletion syndrome,	Not present
**chr6:7231288:C>T**	RREB1	1	0,004476297	0,0257282	c.3189C>T	p.Pro1063=	Noonan-like RASopathy (RREB1), Autosomal dominant | RASopathy Syndrome of Congenital Heart Disease, Genitourinary Malformations, and Developmental Delay, Autosomal dominant de-novo,Autosomal dominant | 22q11.2 deletion syndrome,	Not present
**chr5:150374302:G>A**	TCOF1	1	0,001525917	0,0109756	c.999G>A	p.Gly333=	Treacher Collins syndrome 1, Autosomal dominant | Treacher-Collins syndrome,	Not present
**chr4:125317707:T>C**	FAT4	1	0,000222798	0,00144648	c.1296T>C	p.Pro432=	Hennekam lymphangiectasia-lymphedema syndrome 2, Autosomal recessive | Van Maldergem syndrome 2, Autosomal recessive | Cerebrofacioarticular syndrome, | Hennekam syndrome,	Not present
**chr1:237456642:G>A**	RYR2	1	0,004254018	0,0468266	c.1519G>A	p.Val507Ile	Ventricular arrhythmias due to cardiac ryanodine receptor calcium release deficiency syndrome, Autosomal dominant | Ventricular tachycardia, catecholaminergic polymorphic, 1, Autosomal dominant | Primary Generalised Epilepsy (RYR2), Autosomal dominant | Arrhythmogenic right ventricular dysplasia, familial, 2, AD | Ventricular tachycardia, catecholaminergic polymorphic, 1, with or without atrial dysfunction and/or dilated cardomyopathy, AD | Catecholaminergic polymorphic ventricular tachycardia, | Inherited isolated arrhythmogenic cardiomyopathy, dominant-left variant, | Inherited isolated arrhythmogenic cardiomyopathy, dominant-right variant, | Inherited isolated arrhythmogenic ventricular dysplasia, biventricular variant,	Not present

**chr16:72794380:T>A**	ZFHX3	1	0,000532739	0,00659502	c.8302A>T	p.Thr2768Ser	Atrial fibrillation 8, susceptibility to, Autosomal dominant | Prostate cancer, somatic, Autosomal dominant,Somatic mutation | Spinocerebellar ataxia 4, Autosomal dominant | Childhood partial epilepsy and infantile spasms, Autosomal recessive | Syndromic intellectual disability (ZFHX3), Autosomal dominant de-novo	Present
**chr12:131981504:C>T**	EP400	1	0,000308753	0,00121832	c.1451C>T	p.Pro484Leu	Epilepsy with neurodevelopmental disorders, Autosomal recessive	Present
**chr12:132027442:G>A**	EP400	1	0,000619739	0,0036162	c.5020G>A	p.Val1674Ile	Epilepsy with neurodevelopmental disorders, Autosomal recessive	Present
**chr15:63689685:T>C**	HERC1	1	0,003348223	0,0376476	c.5952A>G	p.Leu1984=	Macrocephaly, dysmorphic facies, and psychomotor retardation, Autosomal recessive | Megalencephaly-severe kyphoscoliosis-overgrowth syndrome,	Present
**chr15:63746981:T>C**	HERC1	1	0,00345613	0,0391288	c.2457A>G	p.Ala819=	Macrocephaly, dysmorphic facies, and psychomotor retardation, Autosomal recessive | Megalencephaly-severe kyphoscoliosis-overgrowth syndrome,	Present
**chr16:2109315:C>T**	PKD1	1	0,000478699	0,00463689	c.5852G>A	p.Arg1951Gln	Polycystic kidney disease 1, Autosomal dominant | Polycystic kidney disease 1 with or without polycystic liver disease, AD | Autosomal dominant polycystic kidney disease, | Autosomal dominant polycystic kidney disease type 1 with tuberous sclerosis,	Present
**chr16:72950556:G>A**	ZFHX3	1	6,2739E-05	0,000478469	c.3129C>T	p.Asn1043=	Atrial fibrillation 8, susceptibility to, Autosomal dominant | Prostate cancer, somatic, Autosomal dominant,Somatic mutation | Spinocerebellar ataxia 4, Autosomal dominant | Childhood partial epilepsy and infantile spasms, Autosomal recessive | Syndromic intellectual disability (ZFHX3), Autosomal dominant de-novo	Present
**chr12:132066948:G>A**	EP400	1	0,000126119	0,000477555	c.8728G>A	p.Gly2910Ser	Epilepsy with neurodevelopmental disorders, Autosomal recessive	Present
**chr4:113358297:A>C**	ANK2	1	0,000119189	0,00022017	c.9679A>C	p.Thr3227Pro	Cardiac arrhythmia, ankyrin-B-related, Autosomal dominant | Long QT syndrome 4, Autosomal dominant | Epilepsy, Autosomal dominant de-novo | Romano-Ward syndrome,	Present
**chr16:72797249:G>A**	ZFHX3	1	0,000137109	0,000242945	c.5433C>T	p.Pro1811=	Atrial fibrillation 8, susceptibility to, Autosomal dominant | Prostate cancer, somatic, Autosomal dominant,Somatic mutation | Spinocerebellar ataxia 4, Autosomal dominant | Childhood partial epilepsy and infantile spasms, Autosomal recessive | Syndromic intellectual disability (ZFHX3), Autosomal dominant de-novo	Present
**chr1:39385472:T>C**	MACF1	1	4,77131E-06	8,56908E-06	c.13887T>C	p.His4629=	Lissencephaly 9 with complex brainstem malformation, Autosomal dominant | Generalised epilepsy, Autosomal recessive,Autosomal dominant de-novo | Posterior-predominant lissencephaly-broad flat pons and medulla-midline crossing defects syndrome,	Present
**chr9:128625846:C>T**	SPTAN1	1	1,92075E-05	9,42685E-05	c.6147C>T	p.Leu2049=	Developmental and epileptic encephalopathy 5, Autosomal dominant | Developmental delay with or without epilepsy, Autosomal dominant | Neuronopathy, distal hereditary motor, autosomal dominant 11, Autosomal dominant | Spastic paraplegia 91, autosomal dominant, with or without cerebellar ataxia, Autosomal dominant | Distal myopathy, Autosomal dominant de-novo,Autosomal dominant | Hereditary spastic paraplegia, Autosomal recessive | Spastic Paraplegia and Cerebellar Ataxia, Autosomal dominant,Autosomal dominant de-novo | Neuronopathy, distal hereditary	Not present

							motor, 11, autosomal dominant, AD | Infantile epileptic spasms syndrome,	
**chr11:96092210:T>TTGC**	MAML2	1	0,000437131	0,00130626	c.1818_1820dup	p.Gln621dup	Mucoepidermoid salivary gland carcinoma,	Not present
**chr15:88845851:C>T**	ACAN	1	0,004609382	0,0165906	c.1398C>T	p.Thr466=	Short stature and advanced bone age, with or without early-onset osteoarthritis and/or osteochondritis dissecans, Autosomal dominant | Spondyloepimetaphyseal dysplasia, aggrecan type, Autosomal recessive | Spondyloepiphyseal dysplasia, Kimberley type, Autosomal dominant | Familial osteochondritis dissecans, Autosomal dominant | Short stature-advanced bone age-early-onset osteoarthritis syndrome,	Not present
**chr4:125415182:A>G**	FAT4	1	0,005823378	0,00922787	c.6219A>G	p.Gln2073=	Hennekam lymphangiectasia-lymphedema syndrome 2, Autosomal recessive | Van Maldergem syndrome 2, Autosomal recessive | Cerebrofacioarticular syndrome, | Hennekam syndrome,	Not present
**chr7:83155272:C>T**	PCLO	1	0,004210356	0,0195853	c.1369G>A	p.Gly457Arg	Pontocerebellar hypoplasia, type 3, Autosomal recessive	Present
**chr6:56900580:T>C**	DST	1	0,00147779	0,00282398	c.258A>G	p.Ala86=	Epidermolysis bullosa simplex 3, localized or generalized intermediate, with bp230 deficiency, Autosomal recessive | Neuropathy, hereditary sensory and autonomic, type VI, Autosomal recessive | Congenital myopathy, Autosomal recessive | Epidermolysis bullosa simplex due to BP230 deficiency, | Hereditary sensory and autonomic neuropathy type 6,	Present
**chr15:63696158:C>INS**	HERC1	1	0	0	Unknown	Unknown	Macrocephaly, dysmorphic facies, and psychomotor retardation, Autosomal recessive | Megalencephaly-severe kyphoscoliosis-overgrowth syndrome,	Present
**chr5:83572449:A>INS**	VCAN	1	0	0	Unknown	Unknown	Wagner syndrome 1, Autosomal dominant | Wagner vitreoretinopathy, AD | Wagner disease,	Not present
**chr4:103181365:A>INS**	CENPE	1	0	0	Unknown	Unknown	Microcephaly 13, primary, autosomal recessive, Autosomal recessive | Autosomal recessive primary microcephaly, | Seckel syndrome,	Not present
**chr6:7246473:C>A**	RREB1	1	8,52687E-06	3,32182E-05	c.4023C>A	p.Asp1341Glu	Noonan-like RASopathy (RREB1), Autosomal dominant | RASopathy Syndrome of Congenital Heart Disease, Genitourinary Malformations, and Developmental Delay, Autosomal dominant de-novo,Autosomal dominant | 22q11.2 deletion syndrome,	Not present
**chr2:169212200:G>A**	LRP2	1	0,002044068	0,0153304	c.6048C>T	p.Ala2016=	Donnai-Barrow syndrome, Autosomal recessive | Faciooculoacousticorenal syndrome, AR	Present
**chr10:77843632:G>A**	DLG5	1	7,94147E-05	0,000131303	c.939C>T	p.Val313=	Yuksel-Vogel-Bauser syndrome, Autosomal recessive | Congenital Heart Disease, Congenital Anomalies including Ciliopathy Phenotypes, Autosomal dominant,Autosomal recessive	Not present
**chr12:132011621:A>G**	EP400	1	0,002547366	0,00409671	c.3428A>G	p.Lys1143Arg	Epilepsy with neurodevelopmental disorders, Autosomal recessive	Present
**chr15:88847896:C>G**	ACAN	1	0	0	c.1605-15C>G	Unknown	Short stature and advanced bone age, with or without early-onset osteoarthritis and/or osteochondritis dissecans, Autosomal dominant | Spondyloepimetaphyseal dysplasia, aggrecan type, Autosomal recessive | Spondyloepiphyseal dysplasia, Kimberley type, Autosomal dominant | Familial osteochondritis dissecans, Autosomal dominant | Short stature-advanced bone age-early-onset osteoarthritis syndrome,	Not present
**chr16:2099781:G>A**	PKD1	1	0,001377072	0,00210193	c.9924-11C>T	Unknown	Polycystic kidney disease 1, Autosomal dominant | Polycystic kidney disease 1 with or without polycystic liver disease, AD | Autosomal dominant polycystic kidney disease, | Autosomal dominant polycystic kidney disease type 1 with tuberous sclerosis,	Present

**chr4:125319813:A>T**	FAT4	1	0,001702731	0,0144009	c.3402A>T	p.Glu1134Asp	Hennekam lymphangiectasia-lymphedema syndrome 2, Autosomal recessive | Van Maldergem syndrome 2, Autosomal recessive | Cerebrofacioarticular syndrome, | Hennekam syndrome,	Not present
**chr4:125491756:A>G**	FAT4	1	0,001203168	0,00315344	c.14940A>G	p.Glu4980=	Hennekam lymphangiectasia-lymphedema syndrome 2, Autosomal recessive | Van Maldergem syndrome 2, Autosomal recessive | Cerebrofacioarticular syndrome, | Hennekam syndrome,	Not present
**chr6:56618881:T>G**	DST	1	0,002776988	0,00389599	c.5153A>C	p.Lys1718Thr	Epidermolysis bullosa simplex 3, localized or generalized intermediate, with bp230 deficiency, Autosomal recessive | Neuropathy, hereditary sensory and autonomic, type VI, Autosomal recessive | Congenital myopathy, Autosomal recessive | Epidermolysis bullosa simplex due to BP230 deficiency, | Hereditary sensory and autonomic neuropathy type 6,	Present
**chr6:56629343:T>C**	DST	1	6,40574E-06	0,000014705	c.4382A>G	p.Tyr1461Cys	Epidermolysis bullosa simplex 3, localized or generalized intermediate, with bp230 deficiency, Autosomal recessive | Neuropathy, hereditary sensory and autonomic, type VI, Autosomal recessive | Congenital myopathy, Autosomal recessive | Epidermolysis bullosa simplex due to BP230 deficiency, | Hereditary sensory and autonomic neuropathy type 6,	Present
**chr15:63725336:G>A**	HERC1	1	2,3057E-05	0,000113033	c.3524C>T	p.Thr1175Met	Macrocephaly, dysmorphic facies, and psychomotor retardation, Autosomal recessive | Megalencephaly-severe kyphoscoliosis-overgrowth syndrome,	Present
**chr2:169202833:C>T**	LRP2	1	0,000215083	0,00891996	c.8132G>A	p.Arg2711His	Donnai-Barrow syndrome, Autosomal recessive | Faciooculoacousticorenal syndrome, AR	Present
**chr1:39322623:C>A**	MACF1	1	0,001206127	0,00331439	c.4045C>A	p.Leu1349Met	Lissencephaly 9 with complex brainstem malformation, Autosomal dominant | Generalised epilepsy, Autosomal recessive,Autosomal dominant de-novo | Posterior-predominant lissencephaly-broad flat pons and medulla-midline crossing defects syndrome,	Present
**chr6:7230707:C>T**	RREB1	1	1,83602E-05	5,96036E-05	c.2608C>T	p.Leu870Phe	Noonan-like RASopathy (RREB1), Autosomal dominant | RASopathy Syndrome of Congenital Heart Disease, Genitourinary Malformations, and Developmental Delay, Autosomal dominant de-novo,Autosomal dominant | 22q11.2 deletion syndrome,	Not present
**chr9:128566850:G>A**	SPTAN1	1	3,18083E-06	3,02133E-05	c.110G>A	p.Arg37His	Developmental and epileptic encephalopathy 5, Autosomal dominant | Developmental delay with or without epilepsy, Autosomal dominant | Neuronopathy, distal hereditary motor, autosomal dominant 11, Autosomal dominant | Spastic paraplegia 91, autosomal dominant, with or without cerebellar ataxia, Autosomal dominant | Distal myopathy, Autosomal dominant de-novo,Autosomal dominant | Hereditary spastic paraplegia, Autosomal recessive | Spastic Paraplegia and Cerebellar Ataxia, Autosomal dominant,Autosomal dominant de-novo | Neuronopathy, distal hereditary motor, 11, autosomal dominant, AD | Infantile epileptic spasms syndrome,	Not present
**chr1:109265801:T>G**	CELSR2	1	0,002431648	0,00602991	c.5794T>G	p.Leu1932Val	Joubert Syndrome, Autosomal recessive	Not present
**chr4:3074969:T>G**	HTT	1	0,001043317	0,00331762	c.144T>G	p.Pro48=	Huntington disease, Autosomal dominant | Lopes-Maciel-Rodan syndrome, Autosomal recessive | Lopes-Maciel-Rodan syndrome (LOMARS), AD/AR | Juvenile Huntington disease, | Non-specific syndromic intellectual disability,	Not present
**chr4:76741208:C>A**	SHROOM3	1	0,007690161	0,0170815	c.3035C>A	p.Thr1012Asn	Heterotaxy, Autosomal recessive | Neural Tube Defects, Autosomal dominant	Not present

**chr6:32017141:C>A**	C4B	1	0	0	c.1040C>A	p.Ser347Tyr	C4B deficiency, | Complement component 4B deficiency, AR | Immunodeficiency due to a classical component pathway complement deficiency, | Systemic lupus erythematosus,	Present
**chr2:169206753:G>A**	LRP2	1	0,004249517	0,0144396	c.6967C>T	p.Leu2323=	Donnai-Barrow syndrome, Autosomal recessive | Faciooculoacousticorenal syndrome, AR	Present
**chr5:83542237:G>A**	VCAN	1	0,003157617	0,00844187	c.9234G>A	p.Glu3078=	Wagner syndrome 1, Autosomal dominant | Wagner vitreoretinopathy, AD | Wagner disease,	Not present
**chr7:83154752:G>A**	PCLO	1	4,22706E-05	0,000723589	c.1889C>T	p.Thr630Met	Pontocerebellar hypoplasia, type 3, Autosomal recessive	Present
**chr16:2108418:G>A**	PKD1	1	0,002617478	0,00463689	c.6749C>T	p.Thr2250Met	Polycystic kidney disease 1, Autosomal dominant | Polycystic kidney disease 1 with or without polycystic liver disease, AD | Autosomal dominant polycystic kidney disease, | Autosomal dominant polycystic kidney disease type 1 with tuberous sclerosis,	Present
**chr1:109250529:G>A**	CELSR2	1	0,000152421	0,00161896	c.450G>A	p.Leu150=	Joubert Syndrome, Autosomal recessive	Not present
**chr12:131986551:C>T**	EP400	1	1,41072E-05	0,00094697	c.1967C>T	p.Pro656Leu	Epilepsy with neurodevelopmental disorders, Autosomal recessive	Present
**chr12:132066974:C>T**	EP400	1	1,4889E-05	2,48262E-05	c.8749+5C>T	Unknown	Epilepsy with neurodevelopmental disorders, Autosomal recessive	Present
**chr7:152162335:C>T**	KMT2C	1	0,001213651	0,013756	c.11242G>A	p.Ala3748Thr	Kleefstra syndrome 2, Autosomal dominant | Neurodevelopmental disorder distinct from Kleefstra and Kabuki syndromes, Autosomal dominant,Autosomal dominant de-novo | Kleefstra syndrome due to a point mutation,	Present
**chr11:1227704:A>G**	MUC5B	1	5,24993E-06	3,22331E-05	c.697A>G	p.Asn233Asp	Interstitial lung disease 2, Autosomal dominant | Diffuse panbronchiolitis, | Hypersensitivity pneumonitis, | Idiopathic pulmonary fibrosis,	Not present
**chr11:1251158:A>T**	MUC5B	1	2,18313E-05	3,71909E-05	c.14278A>T	p.Thr4760Ser	Interstitial lung disease 2, Autosomal dominant | Diffuse panbronchiolitis, | Hypersensitivity pneumonitis, | Idiopathic pulmonary fibrosis,	Not present
**chr12:6936728:A>ACAGCAGCAGCAGCAGCAGCAGCAGCAG**	ATN1	1	0,000953142	0,00266588	c.1482_1508dup	p.Gln494_Gln502dup	Congenital hypotonia, epilepsy, developmental delay, and digital anomalies, Autosomal dominant | Dentatorubral-pallidoluysian atrophy, Autosomal dominant | Dentatorubro-pallidoluysian atrophy, AD	Not present
**chr15:63637592:A>G**	HERC1	1	9,6998E-06	5,74152E-05	c.12145T>C	p.Leu4049=	Macrocephaly, dysmorphic facies, and psychomotor retardation, Autosomal recessive | Megalencephaly-severe kyphoscoliosis-overgrowth syndrome,	Present
**chr4:3074876:C>CCAGCAGCAGCAGCAGCAGCAG**	HTT	1	0,009067625	0,0118734	c.90_110dup	p.Gln32_Gln38dup	Huntington disease, Autosomal dominant | Lopes-Maciel-Rodan syndrome, Autosomal recessive | Lopes-Maciel-Rodan syndrome (LOMARS), AD/AR | Juvenile Huntington disease, | Non-specific syndromic intellectual disability,	Not present
**chr4:125450461:G>A**	FAT4	1	0,000946339	0,00772228	c.9451G>A	p.Ala3151Thr	Hennekam lymphangiectasia-lymphedema syndrome 2, Autosomal recessive | Van Maldergem syndrome 2, Autosomal recessive | Cerebrofacioarticular syndrome, | Hennekam syndrome,	Not present
**chr5:83539768:A>G**	VCAN	1	9,54548E-06	2,28781E-05	c.6765A>G	p.Gly2255=	Wagner syndrome 1, Autosomal dominant | Wagner vitreoretinopathy, AD | Wagner disease,	Not present
**chr6:7189230:C>G**	RREB1	1	2,30815E-05	0,000206782	c.333C>G	p.Ser111=	Noonan-like RASopathy (RREB1), Autosomal dominant | RASopathy Syndrome of Congenital Heart Disease, Genitourinary Malformations, and Developmental Delay, Autosomal dominant de-novo,Autosomal dominant | 22q11.2 deletion syndrome,	Not present
**chr7:82953722:G>T**	PCLO	1	5,01748E-05	9,17883E-05	c.7231C>A	p.Pro2411Thr	Pontocerebellar hypoplasia, type 3, Autosomal recessive	Present
**chr2:169152880:C>T**	LRP2	1	0,002339339	0,0255958	c.12380G>A	p.Arg4127His	Donnai-Barrow syndrome, Autosomal recessive | Faciooculoacousticorenal syndrome, AR	Present
**chr2:169243501:G>A**	LRP2	1	0,009127064	0,0342198	c.3452C>T	p.Pro1151Leu	Donnai-Barrow syndrome, Autosomal recessive | Faciooculoacousticorenal syndrome, AR	Present

**chr7:152221999:A>INS**	KMT2C	1	0	0	Unknown	Unknown	Kleefstra syndrome 2, Autosomal dominant | Neurodevelopmental disorder distinct from Kleefstra and Kabuki syndromes, Autosomal dominant,Autosomal dominant de-novo | Kleefstra syndrome due to a point mutation,	Present
**chr4:103123040:G>C**	CENPE	1	0,00734665	0,0244957	c.6974C>G	p.Ser2325Cys	Microcephaly 13, primary, autosomal recessive, Autosomal recessive | Autosomal recessive primary microcephaly, | Seckel syndrome,	Not present
**chr4:113357679:G>A**	ANK2	1	0,001553093	0,0190525	c.9061G>A	p.Ala3021Thr	Cardiac arrhythmia, ankyrin-B-related, Autosomal dominant | Long QT syndrome 4, Autosomal dominant | Epilepsy, Autosomal dominant de-novo | Romano-Ward syndrome,	Present
**chr5:83539126:C>T**	VCAN	1	0,003552857	0,0412451	c.6123C>T	p.Ile2041=	Wagner syndrome 1, Autosomal dominant | Wagner vitreoretinopathy, AD | Wagner disease,	Not present
**chr4:113373380:C>T**	ANK2	1	0,003964138	0,0336479	c.11790C>T	p.Ile3930=	Cardiac arrhythmia, ankyrin-B-related, Autosomal dominant | Long QT syndrome 4, Autosomal dominant | Epilepsy, Autosomal dominant de-novo | Romano-Ward syndrome,	Present
**chr4:125317512:A>G**	FAT4	1	0,004211249	0,047281	c.1101A>G	p.Val367=	Hennekam lymphangiectasia-lymphedema syndrome 2, Autosomal recessive | Van Maldergem syndrome 2, Autosomal recessive | Cerebrofacioarticular syndrome, | Hennekam syndrome,	Not present
**chr4:125320565:T>C**	FAT4	1	0,001709286	0,019783	c.4154T>C	p.Leu1385Ser	Hennekam lymphangiectasia-lymphedema syndrome 2, Autosomal recessive | Van Maldergem syndrome 2, Autosomal recessive | Cerebrofacioarticular syndrome, | Hennekam syndrome,	Not present
**chr5:83538158:A>T**	VCAN	1	0,003643661	0,0425911	c.5155A>T	p.Thr1719Ser	Wagner syndrome 1, Autosomal dominant | Wagner vitreoretinopathy, AD | Wagner disease,	Not present
**chr5:83538579:C>A**	VCAN	1	0,00364083	0,0425681	c.5576C>A	p.Ala1859Glu	Wagner syndrome 1, Autosomal dominant | Wagner vitreoretinopathy, AD | Wagner disease,	Not present
**chr5:83539240:G>A**	VCAN	1	0,003530415	0,0411532	c.6237G>A	p.Lys2079=	Wagner syndrome 1, Autosomal dominant | Wagner vitreoretinopathy, AD | Wagner disease,	Not present
**chr6:7230718:C>T**	RREB1	1	3,66484E-05	0,000154693	c.2619C>T	p.Gly873=	Noonan-like RASopathy (RREB1), Autosomal dominant | RASopathy Syndrome of Congenital Heart Disease, Genitourinary Malformations, and Developmental Delay, Autosomal dominant de-novo,Autosomal dominant | 22q11.2 deletion syndrome,	Not present
**chr5:150368840:C>T**	TCOF1	1	0,000555729	0,00691244	c.503C>T	p.Thr168Met	Treacher Collins syndrome 1, Autosomal dominant | Treacher-Collins syndrome,	Not present
**chr6:7230973:G>A**	RREB1	1	3,8417E-06	3,02188E-05	c.2874G>A	p.Lys958=	Noonan-like RASopathy (RREB1), Autosomal dominant | RASopathy Syndrome of Congenital Heart Disease, Genitourinary Malformations, and Developmental Delay, Autosomal dominant de-novo,Autosomal dominant | 22q11.2 deletion syndrome,	Not present
**chr7:74691092:A>G**	GTF2I	1	0,00051771	0,00236295	c.219A>G	p.Gln73=	Williams syndrome,	Present
**chr1:109250113:A>ACGCCGCCGC**	CELSR2	1	2,95433E-05	0,000481696	c.41_49dup	p.Pro14_Pro16dup	Joubert Syndrome, Autosomal recessive	Not present
**chr12:131961952:G>A**	EP400	1	5,15322E-06	3,90427E-05	c.1333G>A	p.Glu445Lys	Epilepsy with neurodevelopmental disorders, Autosomal recessive	Present
**chr16:72787715:CGCCACCGCCGCCGCCGCCGCCACT>C**	ZFHX3	1	0,001551022	0,00988142	c.10537_10560del	p.Ser3513_Gly3520del	Atrial fibrillation 8, susceptibility to, Autosomal dominant | Prostate cancer, somatic, Autosomal dominant,Somatic mutation | Spinocerebellar ataxia 4, Autosomal dominant | Childhood partial epilepsy and infantile spasms, Autosomal recessive | Syndromic intellectual disability (ZFHX3), Autosomal dominant de-novo	Present
**chr12:6935973:A>C**	ATN1	1	0,003989958	0,00638667	c.706A>C	p.Lys236Gln	Congenital hypotonia, epilepsy, developmental delay, and digital anomalies, Autosomal dominant |	Not present

							Dentatorubral-pallidoluysian atrophy, Autosomal dominant | Dentatorubro-pallidoluysian atrophy, AD	
**chr11:1247532:C>G**	MUC5B	1	0,000168448	0,000730105	c.10652C>G	p.Ser3551Trp	Interstitial lung disease 2, Autosomal dominant | Diffuse panbronchiolitis, | Hypersensitivity pneumonitis, | Idiopathic pulmonary fibrosis,	Not present
**chr11:1249717:C>T**	MUC5B	1	0,002593242	0,0076555	c.12837C>T	p.Pro4279=	Interstitial lung disease 2, Autosomal dominant | Diffuse panbronchiolitis, | Hypersensitivity pneumonitis, | Idiopathic pulmonary fibrosis,	Not present
**chr9:128625810:C>T**	SPTAN1	1	0,002358321	0,00713487	c.6111C>T	p.Gly2037=	Developmental and epileptic encephalopathy 5, Autosomal dominant | Developmental delay with or without epilepsy, Autosomal dominant | Neuronopathy, distal hereditary motor, autosomal dominant 11, Autosomal dominant | Spastic paraplegia 91, autosomal dominant, with or without cerebellar ataxia, Autosomal dominant | Distal myopathy, Autosomal dominant de-novo,Autosomal dominant | Hereditary spastic paraplegia, Autosomal recessive | Spastic Paraplegia and Cerebellar Ataxia, Autosomal dominant,Autosomal dominant de-novo | Neuronopathy, distal hereditary motor, 11, autosomal dominant, AD | Infantile epileptic spasms syndrome,	Not present
**chr7:83156131:T>C**	PCLO	1	1,59086E-06	3,02334E-05	c.510A>G	p.Val170=	Pontocerebellar hypoplasia, type 3, Autosomal recessive	Present
**chr7:152180043:T>C**	KMT2C	1	0,000834784	0,0036162	c.7233A>G	p.Ser2411=	Kleefstra syndrome 2, Autosomal dominant | Neurodevelopmental disorder distinct from Kleefstra and Kabuki syndromes, Autosomal dominant,Autosomal dominant de-novo | Kleefstra syndrome due to a point mutation,	Present
**chr4:103136208:A>G**	CENPE	1	9,73578E-05	0,000176465	c.6455T>C	p.Ile2152Thr	Microcephaly 13, primary, autosomal recessive, Autosomal recessive | Autosomal recessive primary microcephaly, | Seckel syndrome,	Not present
**chr17:77497305:C>T**	SEPTIN9	1	0,004733798	0,00709984	c.1574-10C>T	Unknown	Amyotrophy, hereditary neuralgic, Autosomal dominant | Neuralgic amyotrophy,	Not present
**chr7:83156330:C>T**	PCLO	1	0,000361631	0,00844187	c.311G>A	p.Arg104His	Pontocerebellar hypoplasia, type 3, Autosomal recessive	Present
**chr1:109269247:G>A**	CELSR2	1	0,00022953	0,00144788	c.6769G>A	p.Gly2257Arg	Joubert Syndrome, Autosomal recessive	Not present
**chr5:150361209:A>G**	TCOF1	1	0,002962838	0,0308377	c.162A>G	p.Gln54=	Treacher Collins syndrome 1, Autosomal dominant | Treacher-Collins syndrome,	Not present
**chr5:150376525:C>T**	TCOF1	1	0,002844479	0,0303701	c.2245C>T	p.Pro749Ser	Treacher Collins syndrome 1, Autosomal dominant | Treacher-Collins syndrome,	Not present
**chr7:74689179:G>A**	GTF2I	1	0,000464944	0,00581677	c.51G>A	p.Ser17=	Williams syndrome,	Present
**chr7:83134242:T>TATAA**	PCLO	1	9,26586E-05	0,000473485	c.3300+7_3300+8insTTAT	Unknown	Pontocerebellar hypoplasia, type 3, Autosomal recessive	Present
**chr1:39387318:T>C**	MACF1	1	0,002011544	0,0164171	c.14476T>C	p.Leu4826=	Lissencephaly 9 with complex brainstem malformation, Autosomal dominant | Generalised epilepsy, Autosomal recessive,Autosomal dominant de-novo | Posterior-predominant lissencephaly-broad flat pons and medulla-midline crossing defects syndrome,	Present
**chr4:76739340:T>A**	SHROOM3	1	0,000206258	0,00474383	c.1167T>A	p.Ser389Arg	Heterotaxy, Autosomal recessive | Neural Tube Defects, Autosomal dominant	Not present
**chr5:83519829:T>A**	VCAN	1	0,002737446	0,0049403	c.1523T>A	p.Ile508Asn	Wagner syndrome 1, Autosomal dominant | Wagner vitreoretinopathy, AD | Wagner disease,	Not present
**chr16:2102618:C>T**	PKD1	1	0,007820876	0,0206059	c.8964G>A	p.Ala2988=	Polycystic kidney disease 1, Autosomal dominant | Polycystic kidney disease 1 with or without polycystic liver disease, AD | Autosomal dominant polycystic kidney disease, | Autosomal dominant polycystic kidney disease type 1 with tuberous sclerosis,	Present
**chr5:150372090:C>T**	TCOF1	1	4,73738E-05	0,000434385	c.724C>T	p.Pro242Ser	Treacher Collins syndrome 1, Autosomal dominant | Treacher-Collins syndrome,	Not present
**chr22:50462625:A>G**	SBF1	1	1,02617E-05	6,54365E-05	c.2061T>C	p.Tyr687=	Charcot-Marie-Tooth disease, type 4B3, Autosomal recessive	Present

**chr7:82914969:C>T**	PCLO	1	6,36604E-06	6,04961E-05	c.13017G>A	p.Pro4339=	Pontocerebellar hypoplasia, type 3, Autosomal recessive	Present
**chr12:131960979:C>T**	EP400	1	0,000710313	0,00483779	c.360C>T	p.Tyr120=	Epilepsy with neurodevelopmental disorders, Autosomal recessive	Present
**chr6:7246415:G>A**	RREB1	1	0,004954504	0,0238498	c.3974-9G>A	Unknown	Noonan-like RASopathy (RREB1), Autosomal dominant | RASopathy Syndrome of Congenital Heart Disease, Genitourinary Malformations, and Developmental Delay, Autosomal dominant de-novo,Autosomal dominant | 22q11.2 deletion syndrome,	Not present
**chr5:83520371:A>G**	VCAN	1	0,009871942	0,0273933	c.2065A>G	p.Thr689Ala	Wagner syndrome 1, Autosomal dominant | Wagner vitreoretinopathy, AD | Wagner disease,	Not present
**chr4:3074876:C>CCAGCAGCAGCAGCAGCAGCAGCAGCAGCAG**	HTT	1	0,003762194	0,00618941	c.81_110dup	p.Gln29_Gln38dup	Huntington disease, Autosomal dominant | Lopes-Maciel-Rodan syndrome, Autosomal recessive | Lopes-Maciel-Rodan syndrome (LOMARS), AD/AR | Juvenile Huntington disease, | Non-specific syndromic intellectual disability,	Not present
**chr4:125451863:C>T**	FAT4	1	0,004314286	0,00619172	c.10853C>T	p.Thr3618Met	Hennekam lymphangiectasia-lymphedema syndrome 2, Autosomal recessive | Van Maldergem syndrome 2, Autosomal recessive | Cerebrofacioarticular syndrome, | Hennekam syndrome,	Not present
**chr16:2097408:C>T**	PKD1	1	8,11003E-05	0,000413736	c.10316G>A	p.Arg3439Gln	Polycystic kidney disease 1, Autosomal dominant | Polycystic kidney disease 1 with or without polycystic liver disease, AD | Autosomal dominant polycystic kidney disease, | Autosomal dominant polycystic kidney disease type 1 with tuberous sclerosis,	Present
**chr16:2108375:T>G**	PKD1	1	0,000114494	0,000477555	c.6792A>C	p.Ser2264=	Polycystic kidney disease 1, Autosomal dominant | Polycystic kidney disease 1 with or without polycystic liver disease, AD | Autosomal dominant polycystic kidney disease, | Autosomal dominant polycystic kidney disease type 1 with tuberous sclerosis,	Present
**chr17:77492713:C>T**	SEPTIN9	1	0,000147253	0,00236518	c.1473C>T	p.Phe491=	Amyotrophy, hereditary neuralgic, Autosomal dominant | Neuralgic amyotrophy,	Not present
**chr16:72795569:G>A**	ZFHX3	1	2,56152E-05	0,00072324	c.7113C>T	p.Ala2371=	Atrial fibrillation 8, susceptibility to, Autosomal dominant | Prostate cancer, somatic, Autosomal dominant,Somatic mutation | Spinocerebellar ataxia 4, Autosomal dominant | Childhood partial epilepsy and infantile spasms, Autosomal recessive | Syndromic intellectual disability (ZFHX3), Autosomal dominant de-novo	Present
**chr16:72797921:G>C**	ZFHX3	1	0,000259935	0,0024108	c.4761C>G	p.Pro1587=	Atrial fibrillation 8, susceptibility to, Autosomal dominant | Prostate cancer, somatic, Autosomal dominant,Somatic mutation | Spinocerebellar ataxia 4, Autosomal dominant | Childhood partial epilepsy and infantile spasms, Autosomal recessive | Syndromic intellectual disability (ZFHX3), Autosomal dominant de-novo	Present
**chr1:109268900:G>A**	CELSR2	1	0,004572928	0,0327629	c.6523G>A	p.Asp2175Asn	Joubert Syndrome, Autosomal recessive	Not present
**chr16:72794085:T>C**	ZFHX3	1	3,07314E-05	0,00072324	c.8597A>G	p.Lys2866Arg	Atrial fibrillation 8, susceptibility to, Autosomal dominant | Prostate cancer, somatic, Autosomal dominant,Somatic mutation | Spinocerebellar ataxia 4, Autosomal dominant | Childhood partial epilepsy and infantile spasms, Autosomal recessive | Syndromic intellectual disability (ZFHX3), Autosomal dominant de-novo	Present
**chr4:3211942:G>A**	HTT	1	0	0	c.6428G>A	p.Ser2143Asn	Huntington disease, Autosomal dominant | Lopes-Maciel-Rodan syndrome, Autosomal recessive | Lopes-Maciel-Rodan syndrome (LOMARS), AD/AR | Juvenile Huntington disease, | Non-specific syndromic intellectual disability,	Not present

**chr9:128576886:C>T**	SPTAN1	1	2,30507E-05	0,000228634	c.715C>T	p.Arg239Trp	Developmental and epileptic encephalopathy 5, Autosomal dominant | Developmental delay with or without epilepsy, Autosomal dominant | Neuronopathy, distal hereditary motor, autosomal dominant 11, Autosomal dominant | Spastic paraplegia 91, autosomal dominant, with or without cerebellar ataxia, Autosomal dominant | Distal myopathy, Autosomal dominant de-novo,Autosomal dominant | Hereditary spastic paraplegia, Autosomal recessive | Spastic Paraplegia and Cerebellar Ataxia, Autosomal dominant,Autosomal dominant de-novo | Neuronopathy, distal hereditary motor, 11, autosomal dominant, AD | Infantile epileptic spasms syndrome,	Not present
**chr12:132062553:AGCAG>A**	EP400	1	0,000236334	0,00292914	c.8187_8190del	p.Gln2729HisfsTer47	Epilepsy with neurodevelopmental disorders, Autosomal recessive	Present

Table S2: Shared Variants Summary.

variant_id	gene	sample_count	gnomad_af	max_af	coding_change	protein_change	sfari_gene_presence
**chr8:100706805:G>DEL**	PABPC1	18	0	0	Unknown	Unknown	Present
**chr7:74753919:A>G**	GTF2I	14	0	0	c.2715A>G	p.Val905=	Present
**chr6:32034963:G>A**	C4B	7	0	0	c.5064G>A	p.Leu1688=	Present
**chr11:96092219:C>INS**	MAML2	6	0	0	Unknown	Unknown	Not present
**chr9:128683961:G>T**	SET	6	0,003145583	0,017336	c.66G>T	p.Leu22=	Present
**chr5:41850114:C>A**	OXCT1	6	0,004932096	0,0382967	c.480G>T	p.Gly160=	Not present
**chr17:77380262:C>INS**	SEPTIN9	5	0	0	Unknown	Unknown	Not present
**chr6:81752068:C>T**	TENT5A	4	0,003646283	0,0213604	c.74G>A	p.Gly25Asp	Not present
**chr16:8903412:T>C**	USP7	4	0,003694787	0,0171332	c.1705-10A>G	Unknown	Present
**chr4:103146038:T>C**	CENPE	4	0,004835843	0,0451974	c.4204A>G	p.Ile1402Val	Not present
**chr6:3225759:G>A**	TUBB2B	4	0,007417465	0,0212818	c.330C>T	p.Ala110=	Not present
**chr19:17656395:G>C**	UNC13A	4	0,0094288	0,0178777	c.771C>G	p.Pro257=	Present
**chr3:169128065:G>C**	MECOM	4	0,001551065	0,00752092	c.614-5C>G	Unknown	Not present
**chr1:23313619:G>A**	HNRNPR	4	0,008242843	0,0266516	c.1101C>T	p.Leu367=	Present
**chr11:10802029:C>INS**	EIF4G2	3	0	0	Unknown	Unknown	Not present
**chr3:151190791:G>T**	MED12L	3	0,0040752	0,015553	c.1828G>T	p.Asp610Tyr	Present
**chr1:16564176:T>C**	NBPF1	3	0,002041372	0,00255805	c.3031A>G	p.Met1011Val	Not present
**chr8:67291967:C>A**	ARFGEF1	3	0,005064257	0,0108486	c.796G>T	p.Asp266Tyr	Not present
**chr1:196677462:T>C**	CFH	3	0,001801192	0,0166747	c.428-14T>C	Unknown	Not present
**chr15:88856875:T>C**	ACAN	3	0,003629524	0,00666667	c.4290T>C	p.Ser1430=	Not present
**chr2:169128828:C>T**	LRP2	3	0,007229619	0,0255545	c.13803G>A	p.Met4601Ile	Present
**chr2:74100904:C>T**	TET3	3	0,002528691	0,0354941	c.4116C>T	p.Ala1372=	Present

**chr7:88114575:C>T**	ADAM22	3	0,002490764	0,00555556	c.474-9C>T	Unknown	Not present
**chr1:16564103:T>A**	NBPF1	3	0,000671772	0,000795229	c.3104A>T	p.Tyr1035Phe	Not present
**chr17:77380255:G>INS**	SEPTIN9	3	0	0	Unknown	Unknown	Not present
**chr19:41905184:T>G**	ARHGEF1	3	0,006546611	0,00937914	c.2259T>G	p.Ala753=	Not present
**chr9:67862963:A>G**	ANKRD20A1	3	4,10752E-05	0,000162681	c.226A>G	p.Thr76Ala	Not present
**chr1:16564149:C>T**	NBPF1	3	0,000417362	0,000601685	c.3058G>A	p.Val1020Ile	Not present
**chr7:152435727:C>T**	KMT2C	3	0,009546793	0,0483368	c.60G>A	p.Glu20=	Present
**chr22:29292471:C>G**	EWSR1	3	0,007610953	0,0132042	c.1046-17C>G	Unknown	Not present
**chr7:103556999:T>C**	RELN	3	0,001503309	0,0163388	c.5775A>G	p.Arg1925=	Present
**chr1:16564177:G>A**	NBPF1	3	0,002040222	0,00253165	c.3030C>T	p.Gly1010=	Not present
**chr8:103918435:T>C**	RIMS2	3	0,004146737	0,0469119	c.2310-6T>C	Unknown	Present
**chr2:112575321:G>A**	POLR1B	3	0,005486682	0,0488301	c.3000G>A	p.Gln1000=	Not present
**chr13:101827878:A>G**	FGF14	3	0,002883385	0,0190421	c.468T>C	p.Pro156=	Present
**chr2:159350063:C>T**	BAZ2B	3	0,006500174	0,0468065	c.4508G>A	p.Ser1503Asn	Present
**chr11:1234256:C>A**	MUC5B	3	0,001886285	0,0108565	c.2429C>A	p.Thr810Asn	Not present
**chr1:173970591:T>C**	RC3H1	3	0,004338828	0,0451439	c.1248A>G	p.Thr416=	Not present
**chr10:69382736:C>T**	HK1	3	0,005339355	0,0420493	c.1515C>T	p.Asn505=	Not present
**chr15:88855993:C>T**	ACAN	3	0	0	c.3408C>T	p.Thr1136=	Not present
**chr17:7849004:C>G**	KDM6B	3	0,000672339	0,0208438	c.2716C>G	p.Pro906Ala	Present
**chr15:88856164:C>T**	ACAN	3	0	0	c.3579C>T	p.Thr1193=	Not present
**chr14:102974082:C>T**	CDC42BPB	3	0,001850367	0,0289296	c.1575G>A	p.Thr525=	Present
**chr3:4836911:C>A**	ITPR1	3	0,005597596	0,0366456	c.8166C>A	p.Gly2722=	Present
**chr1:204549371:C>G**	MDM4	3	0,000728592	0,0048216	c.1162C>G	p.Pro388Ala	Not present
**chr1:10375007:A>G**	KIF1B	3	0,005134272	0,00807179	c.5250A>G	p.Ala1750=	Not present
**chr7:83134242:T>TAA**	PCLO	3	0,004572405	0,0167708	c.3300+6_3300+7dup	Unknown	Present
**chr10:13971769:G>T**	FRMD4A	3	0,006867339	0,0405599	c.31C>A	p.Pro11Thr	Not present
**chr14:18977471:T>C**	POTEM	2	0,00382006	0,00934453	c.923T>C	p.Val308Ala	Not present
**chr19:42273451:G>A**	CIC	2	0,007849041	0,0186349	c.1668G>A	p.Thr556=	Present
**chr18:21452083:GCGATGACCTAGA>G**	GREB1L	2	0,003068637	0,0308571	c.1852_1863del	p.Asp618_Asp621del	Not present
**chr1:57010704:G>A**	DAB1	2	0,005524556	0,0296659	c.1659C>T	p.Ala553=	Not present
**chr6:149379043:G>A**	TAB2	2	0,003060117	0,0198675	c.1128G>A	p.Thr376=	Not present

**chr4:125449554:C>T**	FAT4	2	0,008171652	0,0125864	c.8544C>T	p.Ser2848=	Not present
**chr22:50462490:C>T**	SBF1	2	0,001323861	0,0139551	c.2128-17G>A	Unknown	Present
**chr6:72097141:C>T**	RIMS1	2	0,004616027	0,0179803	c.438C>T	p.Arg146=	Present
**chr13:110169729:G>C**	COL4A1	2	0,001183296	0,0108511	c.3776C>G	p.Pro1259Arg	Not present
**chr3:62372830:C>G**	FEZF2	2	0,003367038	0,00700216	c.39G>C	p.Pro13=	Present
**chr9:136523752:G>A**	NOTCH1	2	0,000923242	0,0161383	c.368C>T	p.Thr123Met	Present
**chr9:132326843:A>C**	SETX	2	0,006380712	0,016681	c.4755T>G	p.Pro1585=	Not present
**chr3:47847329:C>T**	DHX30	2	0,002441423	0,0238277	c.1986C>T	p.Ile662=	Present
**chr4:54736746:G>A**	KIT	2	0,001977064	0,00285244	c.2622G>A	p.Pro874=	Not present
**chr14:102000093:G>A**	DYNC1H1	2	0,009836506	0,0222674	c.3909G>A	p.Ala1303=	Present
**chr21:33553353:T>C**	SON	2	0,008798625	0,0331412	c.4122T>C	p.Thr1374=	Present
**chr7:103561880:C>T**	RELN	2	0,005555904	0,0311888	c.5284G>A	p.Val1762Ile	Present
**chr2:27222276:T>G**	CAD	2	0,000234354	0,0018315	c.435T>G	p.Pro145=	Not present
**chr7:152145254:G>A**	KMT2C	2	0,000549256	0,00431542	c.14073C>T	p.Tyr4691=	Present
**chr7:70790590:G>GCCA**	AUTS2	2	0,008427222	0,0264595	c.3398_3400dup	p.His1133dup	Present
**chr1:239908603:C>T**	CHRM3	2	0,000241124	0,00145631	c.1152C>T	p.Pro384=	Present
**chr2:39013966:G>A**	1 SOS	2	0,007847695	0,0133321	c.1964C>T	p.Pro655Leu	Not present
**chr14:100337086:G>A**	WARS1	2	0,005570339	0,0165819	c.1230C>T	p.Asp410=	Not present
**chr2:46376581:T>C**	EPAS1	2	0,00029575	0,000587993	c.1077T>C	p.Thr359=	Not present
**chr8:60856859:A>C**	CHD7	2	0,00252889	0,00458716	c.7579A>C	p.Met2527Leu	Present
**chr2:169173147:C>T**	LRP2	2	0,006574433	0,0142237	c.11092G>A	p.Val3698Met	Present
**chr12:6957776:G>A**	PTPN6	2	0,004078032	0,0387614	c.1197G>A	p.Pro399=	Not present
**chr6:56617082:G>C**	DST	2	1,6648E-05	6,04376E-05	c.6385C>G	p.Leu2129Val	Present
**chr11:1251385:G>A**	MUC5B	2	0,000736545	0,0156146	c.14505G>A	p.Thr4835=	Not present
**chr2:219060145:G>A**	IHH	2	0,004026989	0,0237498	c.315+8C>T	Unknown	Not present
**chr3:4663188:A>G**	ITPR1	2	0,00234026	0,0161783	c.1536A>G	p.Glu512=	Present
**chr12:6936368:A>G**	ATN1	2	0,004371826	0,0164014	c.1101A>G	p.Pro367=	Not present
**chr1:114405772:G>A**	TRIM33	2	0,004792828	0,0253238	c.2419-13C>T	Unknown	Present
**chr1:154737064:A>C**	KCNN3	2	0,009339993	0,0356967	c.1473T>G	p.Ser491=	Not present
**chr12:118043202:C>T**	WSB2	2	0,003861103	0,0188042	c.358G>A	p.Asp120Asn	Not present

**chr10:96223879:C>A**	BLNK	2	0,00589159	0,0380623	c.472G>T	p.Ala158Ser	Not present
**chr22:50459579:G>A**	SBF1	2	0,000284621	0,00283822	c.3579C>T	p.Pro1193=	Present
**chr18:33743965:A>G**	ASXL3	2	0,000101162	0,00094697	c.4117A>G	p.Lys1373Glu	Present
**chr4:103139790:C>G**	CENPE	2	0,000817216	0,00464094	c.6203G>C	p.Arg2068Thr	Not present
**chr4:103196188:G>A**	CENPE	2	0,000221585	0,000980777	c.213C>T	p.Ile71=	Not present
**chr8:52668141:A>G**	RB1CC1	2	0,004311184	0,007109	c.1053T>C	p.Ile351=	Not present
**chr3:196885322:A>G**	SENP5	2	0,004651121	0,0319882	c.141A>G	p.Pro47=	Not present
**chr15:28257189:T>C**	HERC2	2	0,002708712	0,0350072	c.2389A>G	p.Met797Val	Present
**chr8:76833372:T>C**	ZFHX4	2	4,62887E-05	0,000184374	c.3360T>C	p.Asp1120=	Present
**chr14:35742494:T>C**	RALGAPA1	2	0,004874059	0,00851466	c.1323A>G	p.Gln441=	Not present
**chr12:114683140:G>A**	TBX3	2	0,006857222	0,0105931	c.61C>T	p.Leu21=	Not present
**chr12:57581428:G>A**	KIF5A	2	0,004646873	0,00737491	c.2769G>A	p.Arg923=	Not present
**chr14:73211830:A>G**	PSEN1	2	0,00050322	0,00692441	c.1017A>G	p.Glu339=	Not present
**chr16:2111096:C>A**	PKD1	2	0,007171736	0,0107499	c.4071G>T	p.Leu1357=	Present
**chr9:123010434:G>A**	RABGAP1	2	0,001166755	0,010224	c.1455G>A	p.Arg485=	Not present
**chr15:42752061:G>A**	TTBK2	2	0,002446475	0,0104014	c.3185C>T	p.Thr1062Ile	Not present
**chr12:124486476:G>A**	NCOR2	2	0,006049779	0,0131923	c.198C>T	p.Pro66=	Not present
**chr15:88838778:C>T**	ACAN	2	0,000941275	0,010512	c.186C>T	p.Thr62=	Not present
**chr15:88840109:G>A**	ACAN	2	0,001448761	0,0162889	c.552G>A	p.Thr184=	Not present
**chr15:88856050:C>T**	ACAN	2	0	0	c.3465C>T	p.Thr1155=	Not present
**chr10:87360639:T>C**	NUTM2D	2	0,008087028	0,0192308	c.325T>C	p.Phe109Leu	Not present
**chr19:18853257:C>A**	UPF1	2	0,000941406	0,0113746	c.1063C>A	p.Arg355=	Not present
**chr10:87360768:T>C**	NUTM2D	2	0,00067317	0,00501584	c.454T>C	p.Leu152=	Not present
**chr10:87360777:A>G**	NUTM2D	2	0,000603566	0,00598802	c.463A>G	p.Thr155Ala	Not present
**chr11:4107509:T>C**	RRM1	2	0,004015632	0,0339817	c.361T>C	p.Leu121=	Not present
**chr7:102348423:T>C**	SPDYE6	2	0,000672175	0,00143914	c.1010A>G	p.His337Arg	Not present
**chr16:72794366:G>A**	ZFHX3	2	0,006866793	0,0119093	c.8316C>T	p.His2772=	Present
**chr17:17794090:C>T**	RAI1	2	0,004124379	0,0138329	c.1142C>T	p.Ala381Val	Present
**chr9:131198511:G>C**	NUP214	2	0,002985518	0,019159	c.5017G>C	p.Val1673Leu	Not present
**chr11:112087953:A>G**	SDHD	2	0,006879888	0,0184834	c.149A>G	p.His50Arg	Not present
**chr12:887217:G>A**	WNK1	2	0,002916624	0,00448463	c.5281-4G>A	Unknown	Not present

**chr4:76770867:G>A**	SHROOM3	2	0,00082572	0,0204918	c.5591G>A	p.Gly1864Asp	Not present
**chr5:72198381:G>A**	MAP1B	2	0,005723182	0,00828049	c.5026G>A	p.Val1676Met	Present
**chr4:113355252:G>A**	ANK2	2	0,004421821	0,0499904	c.6634G>A	p.Gly2212Ser	Present
**chr1:182881348:C>T**	DHX9	2	0,003783301	0,0115718	c.2709C>T	p.Ile903=	Present
**chr1:204530752:A>T**	MDM4	2	0,007676413	0,0173519	c.222A>T	p.Val74=	Not present
**chr4:157363418:C>G**	GRIA2	2	0,004784597	0,0441909	c.*4-17C>G	Unknown	Present
**chr2:60921756:C>A**	REL	2	0,004806413	0,0385983	c.992-7C>A	Unknown	Not present
**chr2:74046356:C>A**	TET3	2	8,33235E-05	0,00130702	c.439C>A	p.Arg147=	Present
**chr4:113264911:A>G**	ANK2	2	0,002854825	0,0136919	c.1401A>G	p.Ala467=	Present
**chr11:1241629:C>T**	MUC5B	2	0,002538899	0,0085027	c.4749C>T	p.Gly1583=	Not present
**chr16:15715019:C>G**	MYH11	2	0,00530558	0,0175988	c.5676G>C	p.Glu1892Asp	Not present
**chr12:2679502:C>G**	CACNA1C	2	0,000798802	0,0089545	c.5150C>G	p.Ala1717Gly	Present
**chr7:45622728:C>T**	ADCY1	2	0,000234183	0,00285714	c.1005C>T	p.Phe335=	Present
**chr16:4875083:A>C**	UBN1	2	0,002062682	0,00954832	c.2673A>C	p.Ser891=	Not present
**chr16:2100209:C>T**	PKD1	2	0,006308536	0,0389375	c.9669G>A	p.Thr3223=	Present
**chr10:349428:G>A**	DIP2C	2	0,004924293	0,0378879	c.3012C>T	p.Cys1004=	Present
**chr7:138580646:A>G**	TRIM24	2	0,00153421	0,0139894	c.2670A>G	p.Glu890=	Not present
**chr10:29495017:C>T**	SVIL	2	0,000884416	0,00637121	c.3755-17G>A	Unknown	Not present
**chr9:128577336:C>T**	SPTAN1	2	0,002997263	0,0072324	c.931-16C>T	Unknown	Not present
**chr17:68522799:G>A**	PRKAR1A	2	0,000358509	0,00425733	c.221G>A	p.Arg74His	Not present
**chr2:121425215:C>T**	CLASP1	2	0,002684528	0,00506268	c.2136G>A	p.Pro712=	Present
**chr17:17165623:C>T**	MPRIP	2	0,004486881	0,0161406	c.4032C>T	p.Ser1344=	Not present
**chr13:35583901:A>G**	NBEA	2	0,004807494	0,0130543	c.7039A>G	p.Ile2347Val	Present
**chr4:26430875:A>G**	RBPJ	2	0,001250954	0,0221794	c.1332A>G	p.Ala444=	Not present
**chr21:33553305:TGTCCTGGAGTCTTCGGCTGTGACC>T**	SON	2	0,007845733	0,0326212	c.4090_4113del	p.Ala1364_Ser1371del	Present
**chr8:94668229:C>T**	ESRP1	2	0,00387369	0,0261988	c.1212C>T	p.Ser404=	Not present
**chr19:3977291:G>A**	EEF2	2	0,009952225	0,0151515	c.2307C>T	p.His769=	Not present
**chr10:277559:T>G**	DIP2C	2	0,003338302	0,0254003	c.4437A>C	p.Thr1479=	Present
**chr19:13255218:G>A**	CACNA1A	2	0,001812247	0,0041489	c.4632C>T	p.Thr1544=	Present
**chr1:15937958:G>A**	SPEN	2	0,00127202	0,00797872	c.10656G>A	p.Arg3552=	Present
**chr4:113358472:T>C**	ANK2	2	0,009577955	0,0340251	c.9854T>C	p.Ile3285Thr	Present

**chr1:15931430:T>C**	SPEN	2	0,001859069	0,00940212	c.5190T>C	p.Tyr1730=	Present
**chr11:70485536:G>A**	SHANK2	2	0,000686103	0,00650916	c.4757C>T	p.Pro1586Leu	Present
**chr19:18865675:C>T**	UPF1	2	0,009457258	0,0141129	c.3134C>T	p.Ala1045Val	Not present
**chr10:399211:C>G**	DIP2C	2	0,001523754	0,015429	c.1158G>C	p.Leu386=	Present
**chr1:15930329:T>G**	SPEN	2	0,004039229	0,0296778	c.4089T>G	p.Asp1363Glu	Present
**chr4:76756493:T>A**	SHROOM3	2	0,002405165	0,0181556	c.4754T>A	p.Met1585Lys	Not present
**chr8:107284736:C>T**	ANGPT1	2	0,001403037	0,0410393	c.1151G>A	p.Arg384Gln	Not present
**chr7:103610771:T>C**	RELN	2	0,004267669	0,0473178	c.2932A>G	p.Thr978Ala	Present
**chr16:89281033:G>A**	ANKRD11	2	0,004614669	0,0338567	c.5509C>T	p.Pro1837Ser	Present
**chr9:14307535:G>A**	NFIB	2	0,004408874	0,0481298	c.31-15C>T	Unknown	Present
**chr22:50447442:G>A**	SBF1	2	0,001950707	0,00705687	c.5463C>T	p.Tyr1821=	Present
**chr19:2210737:C>T**	DOT1L	2	0,001257589	0,00672544	c.1233C>T	p.Arg411=	Present
**chr3:101343827:C>A**	SENP7	2	0,000178027	0,00236295	c.1965G>T	p.Leu655Phe	Not present
**chr11:1254776:G>A**	MUC5B	2	0,002744364	0,0190092	c.15560G>A	p.Arg5187His	Not present
**chr10:362527:G>A**	DIP2C	2	0,002759785	0,0253575	c.2757C>T	p.Cys919=	Present
**chr2:25743945:T>C**	ASXL2	2	0,000791415	0,00596785	c.2392A>G	p.Ile798Val	Not present
**chr12:132018266:C>T**	EP400	2	0,000613565	0,00368481	c.4167C>T	p.His1389=	Present
**chr20:63419612:G>A**	KCNQ2	2	0,000154739	0,00178026	c.1301+7C>T	Unknown	Present
**chr2:8731309:G>A**	KIDINS220	2	0,000124206	0,0011583	c.4727C>T	p.Ala1576Val	Not present
**chr10:74095684:C>T**	VCL	2	0,000274042	0,00245558	c.1572C>T	p.Ala524=	Not present
**chr13:20067282:A>G**	ZMYM2	2	0,004341781	0,0498615	c.3345A>G	p.Thr1115=	Present
**chr3:12901261:C>G**	IQSEC1	2	0,006436276	0,0416887	c.3067G>C	p.Ala1023Pro	Not present
**chr17:82083521:C>T**	FASN	2	0,002252697	0,0197807	c.5337G>A	p.Pro1779=	Not present
**chr5:150396504:A>G**	TCOF1	2	0,009253132	0,0208098	c.4007A>G	p.Lys1336Arg	Not present
**chr1:108995640:CGAG>C**	WDR47	2	0,008915881	0,012477	c.1628_1630del	p.Pro543del	Not present
**chr7:151076168:C>T**	SLC4A2	2	0,009901919	0,0267525	c.3627C>T	p.Thr1209=	Not present
**chr19:12706298:G>T**	TNPO2	2	0,000418736	0,00348742	c.1566C>A	p.Thr522=	Not present
**chr14:31173249:A>G**	HECTD1	2	8,36999E-05	0,000482859	c.1063T>C	p.Leu355=	Present
**chr15:33854782:C>T**	RYR3	2	0,000132667	0,000485201	c.13877C>T	p.Ala4626Val	Not present

**chr1:26775584:G>A**	ARID1A	2	0,001158839	0,0115759	c.5001G>A	p.Pro1667=	Present
**chr11:63898288:C>G**	MARK2	2	0,000686303	0,006027	c.337+8C>G	Unknown	Present
**chr1:205159570:G>A**	DSTYK	2	0,003092103	0,0443001	c.2215C>T	p.Arg739Trp	Not present
**chr10:390795:G>A**	DIP2C	2	0,004695515	0,0253551	c.1329C>T	p.Asp443=	Present
**chr10:384040:G>A**	DIP2C	2	0,004608606	0,0253585	c.1863C>T	p.Asp621=	Present
**chr19:46921642:C>T**	ARHGAP35	2	0,000879833	0,0103425	c.2967C>T	p.Ile989=	Not present
**chr6:169241770:T>C**	THBS2	2	0,002451233	0,0225777	c.883A>G	p.Lys295Glu	Not present
**chr4:103182849:G>A**	CENPE	2	0,003430321	0,0363492	c.876C>T	p.Leu292=	Not present
**chr6:137875680:C>G**	TNFAIP3	2	0,005649985	0,0467105	c.487-8C>G	Unknown	Not present
**chr6:39879377:C>A**	DAAM2	2	0,00161666	0,0113308	c.1745C>A	p.Pro582His	Not present
**chr14:77570417:C>T**	SPTLC2	2	0,002459949	0,0273341	c.723G>A	p.Thr241=	Not present
**chr1:149906883:G>T**	SV2A	2	0,000236003	0,00202614	c.8C>A	p.Ala3Asp	Not present
**chr17:31352383:A>G**	NF1	2	0,001187028	0,00820859	c.7584A>G	p.Gln2528=	Present
**chr1:155963149:T>C**	ARHGEF2	2	0,00066688	0,0036162	c.759A>G	p.Thr253=	Present
**chr3:52609327:T>G**	PBRM1	2	0,002411372	0,0217142	c.2598A>C	p.Ala866=	Not present
**chr1:175324434:C>T**	TNR	2	0,002859126	0,0269018	c.3879G>A	p.Ser1293=	Not present
**chr12:6936728:A>ACAGCAGCAGCAGCAGCAGCAGCAG**	ATN1	2	0,001859111	0,00470655	c.1485_1508dup	p.Gln495_Gln502dup	Not present
**chr1:175355581:G>A**	TNR	2	0,003200006	0,0306988	c.3171C>T	p.Ala1057=	Not present
**chr1:237648466:C>T**	RYR2	2	0,004496039	0,0112615	c.7365C>T	p.Asp2455=	Not present
**chr17:31326219:G>A**	NF1	2	0,003818999	0,00650916	c.5235G>A	p.Lys1745=	Present
**chr17:77380254:G>INS**	SEPTIN9	2	0	0	Unknown	Unknown	Not present
**chr1:186360792:C>T**	TPR	2	5,51545E-05	0,000967118	c.1072G>A	p.Asp358Asn	Not present
**chr3:49532782:C>T**	DAG1	2	0,002499513	0,0250933	c.2271C>T	p.Ala757=	Not present
**chr2:3575820:C>A**	RPS7	2	5,64501E-05	0,000657462	c.79C>A	p.Leu27Ile	Not present
**chr3:77644825:C>T**	ROBO2	2	0,000532672	0,00331126	c.4341C>T	p.Gly1447=	Present
**chr1:116579666:G>GTCCTCC**	IGSF3	2	0,000745096	0,00335893	c.3054_3059dup	p.Glu1018_Glu1019dup	Not present
**chr16:30964180:C>T**	SETD1A	2	0,004950951	0,0113308	c.726C>T	p.Pro242=	Present
**chr5:139293962:C>INS**	MATR3	2	0	0	Unknown	Unknown	Not present
**chr7:50604057:G>A**	GRB10	2	0,008703044	0,0183221	c.1485C>T	p.His495=	Present
**chr3:49030617:G>A**	QRICH1	2	0,0015969	0,0307427	c.2166C>T	p.Asp722=	Present
**chr8:47778507:G>A**	PRKDC	2	0,006353945	0,0347323	c.11805C>T	p.Gly3935=	Present
**chr8:100709222:G>DEL**	PABPC1	2	0	0	Unknown	Unknown	Present

**chr11:118379543:C>T**	UBE4A	2	0,000368696	0,00355906	c.1669C>T	p.Leu557Phe	Not present
**chr18:51065553:T>C**	SMAD4	2	0,001774452	0,0167147	c.1086T>C	p.Phe362=	Present
**chr14:102944355:TCCG>T**	CDC42BPB	2	0,000211515	0,00144648	c.3941_3943del	p.Ala1314del	Present
**chr8:13499807:C>T**	DLC1	2	0,000460969	0,00176448	c.265G>A	p.Asp89Asn	Not present
**chr7:128854456:A>G**	FLNC	2	0,002626389	0,0264044	c.6771A>G	p.Pro2257=	Not present
**chr7:103489775:C>T**	RELN	2	0,003557115	0,0420999	c.9730G>A	p.Gly3244Ser	Present
**chr7:103486277:G>A**	RELN	2	0,003832041	0,0437566	c.9903C>T	p.Tyr3301=	Present
**chr10:77796543:A>G**	DLG5	2	0,003152191	0,0354617	c.5216T>C	p.Leu1739Pro	Not present
**chr10:77796547:C>T**	DLG5	2	0,003399436	0,0385689	c.5212G>A	p.Ala1738Thr	Not present
**chr9:106929340:G>A**	ZNF462	2	0,000139575	0,000640146	c.5428G>A	p.Val1810Ile	Present
**chr14:36663408:G>A**	PAX9	2	0,007749891	0,0182589	c.516G>A	p.Lys172=	Not present
**chr4:41746200:G>A**	PHOX2B	2	0,009333176	0,022203	c.552C>T	p.Ser184=	Not present
**chr15:88858594:T>C**	ACAN	2	0,007223823	0,0397612	c.6009T>C	p.Tyr2003=	Not present
**chr15:41095913:A>G**	INO80	2	0,00023176	0,00168756	c.159T>C	p.Asp53=	Not present
**chr9:20414300:A>G**	MLLT3	1	0,001801077	0,00409281	c.546T>C	p.Ser182=	Not present
**chr8:47881511:A>G**	PRKDC	1	1,69435E-06	2,98622E-06	c.4972T>C	p.Ser1658Pro	Present
**chr19:5137668:C>T**	KDM4B	1	5,01198E-05	0,000473934	c.2433C>T	p.Thr811=	Present
**chr9:27197590:C>T**	TEK	1	0,000918376	0,00289575	c.1900C>T	p.Leu634Phe	Present
**chr8:47930755:G>A**	PRKDC	1	0,000112501	0,00103434	c.1809C>T	p.Ile603=	Present
**chr1:42931063:G>A**	SLC2A1	1	0,000101165	0,000257894	c.258C>T	p.Phe86=	Not present
**chr9:99828310:C>A**	NR4A3	1	2,0526E-05	0,000114411	c.268C>A	p.Arg90=	Not present
**chr19:5111513:A>C**	KDM4B	1	8,88763E-05	0,00120715	c.1254A>C	p.Leu418=	Present
**chr17:37097916:G>A**	ACACA	1	0,001241927	0,00214791	c.6634C>T	p.Leu2212=	Not present
**chr10:60076010:G>C**	ANK3	1	0	0	c.4871C>G	p.Thr1624Ser	Present
**chr16:28826366:G>A**	ATXN2L	1	0,009845166	0,0262332	c.592G>A	p.Val198Ile	Not present
**chr16:2056724:C>G**	TSC2	1	0,003543514	0,013936	c.729C>G	p.Leu243=	Present
**chr10:77824421:C>T**	DLG5	1	0,002465981	0,0129846	c.2345G>A	p.Arg782His	Not present
**chr10:92632569:C>A**	KIF11	1	0,00073372	0,00802623	c.1578C>A	p.His526Gln	Not present
**chr15:84782807:A>G**	ZNF592	1	1,59037E-06	2,85626E-06	c.132A>G	p.Pro44=	Not present
**chr10:102599470:C>G**	SUFU	1	2,56108E-06	1,46977E-05	c.948C>G	p.Leu316=	Not present

**chr15:63654327:ACAG>A**	HERC1	1	8,48613E-05	0,00141777	c.10085-6_10085-4del	Unknown	Present
**chr14:105167784:G>A**	JAG2	1	0,004465448	0,00676285	c.390C>T	p.Val130=	Not present
**chr13:28322931:T>C**	FLT1	1	0,003070399	0,00935162	c.2812A>G	p.Met938Val	Not present
**chr12:132044179:C>T**	EP400	1	0,006797198	0,0266386	c.6453C>T	p.Asp2151=	Present
**chr10:110116304:C>T**	ADD3	1	2,56108E-06	0,000030217	c.380C>T	p.Thr127Ile	Not present
**chr8:28717909:C>T**	EXTL3	1	4,7712E-06	8,56903E-06	c.1850C>T	p.Thr617Ile	Not present
**chr5:168700650:G>A**	SLIT3	1	0,001793666	0,0120432	c.2874C>T	p.Thr958=	Not present
**chr19:13298582:C>G**	CACNA1A	1	6,5722E-06	1,46998E-05	c.3051G>C	p.Ala1017=	Present
**chr19:58547421:T>C**	TRIM28	1	0	0	c.632T>C	p.Val211Ala	Not present
**chr1:154737064:AG>A**	KCNN3	1	0,000116745	0,000473485	c.1472del	p.Ser491LeufsTer15	Not present
**chr3:194405946:T>C**	ATP13A3	1	0,000343142	0,00156965	c.3744A>G	p.Gly1248=	Not present
**chr3:47123222:G>A**	SETD2	1	3,82247E-05	0,000305488	c.1414C>T	p.Arg472Cys	Present
**chr17:17797971:G>C**	RAI1	1	0,000577456	0,00724938	c.5023G>C	p.Val1675Leu	Present
**chr3:189864351:A>G**	TP63	1	0,000162607	0,000326797	c.699A>G	p.Lys233=	Not present
**chr19:49601605:C>T**	PRR12	1	0,000161663	0,000956938	c.4460C>T	p.Pro1487Leu	Present
**chr3:132499777:G>A**	DNAJC13	1	0,009448603	0,0172804	c.4385G>A	p.Arg1462His	Not present
**chr3:31622289:A>C**	STT3B	1	1,59084E-06	2,85714E-06	c.1520A>C	p.Gln507Pro	Not present
**chr16:2109302:G>T**	PKD1	1	3,19202E-05	0,000115827	c.5865C>A	p.His1955Gln	Present
**chr6:24848117:C>T**	RIPOR2	1	6,14853E-05	0,000143034	c.1072G>A	p.Ala358Thr	Not present
**chr3:23917934:C>G**	RPL15	1	0,005870298	0,0216014	c.75C>G	p.Val25=	Not present
**chr2:232335787:G>T**	DIS3L2	1	0,000154718	0,00101266	c.2409G>T	p.Arg803=	Not present
**chr4:10088333:G>A**	WDR1	1	1,23936E-05	6,21968E-05	c.677C>T	p.Ala226Val	Not present
**chr10:60076055:G>A**	ANK3	1	0,000273979	0,00177962	c.4826C>T	p.Thr1609Met	Present
**chr12:100326625:C>T**	SCYL2	1	8,24069E-05	0,000191289	c.1513C>T	p.Arg505Cys	Not present
**chr1:239908754:G>A**	CHRM3	1	0,000187002	0,00144718	c.1303G>A	p.Val435Met	Present
**chr2:232334670:A>G**	DIS3L2	1	0,000330317	0,00149328	c.2329A>G	p.Ile777Val	Not present
**chr15:41085517:C>T**	INO80	1	5,7618E-05	0,000964506	c.725G>A	p.Arg242His	Not present
**chr1:7661873:C>T**	CAMTA1	1	5,48366E-05	0,000485673	c.805+7C>T	Unknown	Not present
**chr1:21728683:T>C**	USP48	1	0,000891136	0,00376577	c.1337A>G	p.Lys446Arg	Not present

**chr1:186334538:T>TA**	TPR	1	0,000502677	0,00268424	c.4974-6dup	Unknown	Not present
**chr1:109250126:C>CGCT**	CELSR2	1	0,00023322	0,000646128	c.58_60dup	p.Leu25dup	Not present
**chr1:94480580:T>C**	ABCD3	1	2,04979E-05	0,000724288	c.801T>C	p.Tyr267=	Not present
**chr4:3074923:AGCAG>A**	HTT	1	0,003254982	0,0102328	c.99_102del	p.Gln33HisfsTer67	Not present
**chr4:3105413:C>T**	HTT	1	0,000518521	0,0018109	c.585C>T	p.His195=	Not present
**chr4:39321353:C>T**	RFC1	1	0,000148586	0,000457645	c.742G>A	p.Gly248Arg	Not present
**chr6:160061757:C>T**	IGF2R	1	0,000165175	0,00216972	c.3411C>T	p.Ser1137=	Not present
**chr7:82956442:G>C**	PCLO	1	0,002414487	0,025994	c.4511C>G	p.Thr1504Ser	Present
**chr7:82952241:C>T**	PCLO	1	0,003542593	0,0380654	c.8712G>A	p.Lys2904=	Present
**chr20:13534052:C>T**	TASP1	1	0,004890414	0,0195751	c.765G>A	p.Leu255=	Not present
**chr20:13587326:C>A**	TASP1	1	0,004858094	0,0190606	c.327G>T	p.Leu109=	Not present
**chr21:33797505:C>T**	ITSN1	1	0,004188746	0,0124622	c.2079C>T	p.Gly693=	Present
**chr22:39225814:G>A**	PDGFB	1	0,00580387	0,0148909	c.635C>T	p.Thr212Met	Not present
**chr22:50454820:G>A**	SBF1	1	8,1976E-05	0,000891918	c.4806C>T	p.Asp1602=	Present
**chr19:16869506:C>T**	SIN3B	1	1,59222E-06	2,85879E-06	c.1853C>T	p.Pro618Leu	Present
**chr7:73465443:T>G**	BAZ1B	1	0	0	c.3067A>C	p.Lys1023Gln	Not present
**chr7:50368238:C>T**	IKZF1	1	0,000870466	0,00268958	c.393C>T	p.Asn131=	Present
**chr6:71968976:G>A**	RIMS1	1	6,27725E-05	0,000105735	c.165-7G>A	Unknown	Present
**chr4:125316935:G>T**	FAT4	1	0,003108573	0,0204918	c.524G>T	p.Arg175Leu	Not present
**chr17:31358497:C>T**	NF1	1	4,77269E-06	8,57094E-06	c.7988C>T	p.Ser2663Phe	Present
**chr19:2434473:T>C**	LMNB2	1	3,84617E-06	1,47054E-05	c.1024A>G	p.Met342Val	Not present
**chr19:2444522:G>C**	LMNB2	1	0	0	c.283C>G	p.Leu95Val	Not present
**chr9:98290633:C>T**	GABBR2	1	1,47953E-05	2,58818E-05	c.2777G>A	p.Arg926His	Present
**chr4:159352800:A>G**	RAPGEF2	1	0	0	c.3981A>G	p.Ser1327=	Not present
**chr4:73078731:G>A**	ANKRD17	1	6,36155E-06	6,04266E-05	c.7319C>T	p.Thr2440Met	Present
**chr17:7191901:C>T**	DLG4	1	4,14453E-06	3,82585E-05	c.1968G>A	p.Glu656=	Present
**chr20:58854540:C>A**	GNAS	1	3,36791E-06	3,11993E-05	c.1088C>A	p.Ser363Ter	Present
**chr4:148435156:T>C**	NR3C2	1	0,000597849	0,00342417	c.1705A>G	p.Arg569Gly	Present
**chr4:139071944:T>C**	ELF2	1	0,001339518	0,00627413	c.448A>G	p.Thr150Ala	Not present
**chr2:128318353:G>A**	HS6ST1	1	3,18856E-06	2,28707E-05	c.211C>T	p.Pro71Ser	Not present
**chr4:84690563:C>T**	WDFY3	1	6,01819E-05	0,000477555	c.9306G>A	p.Val3102=	Present

**chr11:108172808:TAGA>T**	NPAT	1	0,00959041	0,0166345	c.2173_2175del	p.Ser726del	Not present
**chr17:57979247:TGC>T**	VEZF1	1	0,00529446 2	0,0282293	c.1041_1042del	p.Gln348AlafsTer27	Present
**chr17:72121467:A>G**	SOX9	1	0,00047847 4	0,00332655	c.76A>G	p.Met26Val	Not present
**chr18:75288548:G>A**	TSHZ1	1	0,00388172 5	0,00816736	c.3141G>A	p.Ala1047=	Present
**chr19:2222326:G>A**	DOT1L	1	0,00166058	0,00964785	c.3157G>A	p.Ala1053Thr	Present
**chr19:3113320:G>A**	GNA11	1	0,00046165 3	0,00518242	c.322-10G>A	Unknown	Not present
**chr19:38726952:C>T**	ACTN4	1	0,00238094 6	0,026686	c.2191-5C>T	Unknown	Present
**chr19:46850659:G>A**	AP2S1	1	8,08638E-06	1,52124E-05	c.6C>T	p.Val2=	Present
**chr19:49909531:C>T**	NUP62	1	0,00141996	0,0159751	c.277G>A	p.Gly93Ser	Not present
**chr20:59900784:T>C**	SYCP2	1	0,00023594 6	0,0024855	c.1217A>G	p.Lys406Arg	Not present
**chr20:63406719:C>T**	KCNQ2	1	0,00304388 4	0,00584737	c.2544G>A	p.Pro848=	Present
**chr22:41125999:A>G**	EP300	1	0,00240179	0,00382174	c.865A>G	p.Met289Val	Present
**chr1:225422231:G>A**	LBR	1	2,56177E-06	1,46985E-05	c.212C>T	p.Ser71Phe	Not present
**chr5:139319028:A>DEL**	MATR3	1	0	0	Unknown	Unknown	Not present
**chr22:29678185:C>T**	NF2	1	7,30052E-05	0,00072324	c.1447-11C>T	Unknown	Not present
**chr14:95117713:T>C**	DICER1	1	0	0	c.1418A>G	p.Tyr473Cys	Not present
**chr1:15934254:G>A**	SPEN	1	6,6581E-05	0,00054442 8	c.8014G>A	p.Val2672Met	Present
**chr5:139322505:C>DEL**	MATR3	1	0	0	Unknown	Unknown	Not present
**chr5:139326282:A>DEL**	MATR3	1	0	0,001188	Unknown	Unknown	Not present
**chr17:61482988:C>G**	TBX4	1	0,00028809 8	0,00343151	c.1113C>G	p.Pro371=	Not present
**chr17:57979245:GC>G**	VEZF1	1	0,00582196 2	0,0288172	c.1044del	p.Gln348HisfsTer9	Present
**chr11:1449797:C>G**	BRSK2	1	0,00069694 2	0,00265572	c.1248C>G	p.Ala416=	Present
**chr17:12763374:C>A**	MYOCD	1	0,00124507 3	0,00206163	c.2691C>A	p.Asp897Glu	Present
**chr11:57801985:C>T**	CTNND1	1	0,00011653 3	0,00033256 7	c.1209C>T	p.Asp403=	Present
**chr11:61784292:C>T**	MYRF	1	0,00357324 9	0,035445	c.3207C>T	p.Pro1069=	Not present
**chr11:102223693:C>T**	YAP1	1	0,00613464 7	0,00955629	c.1104C>T	p.Pro368=	Not present
**chr11:108161949:G>A**	NPAT	1	0,00521510 5	0,0116401	c.3137C>T	p.Pro1046Leu	Not present
**chr11:128485068:T>C**	ETS1	1	0,00265853 3	0,00381545	c.617A>G	p.Tyr206Cys	Not present
**chr12:754011:C>T**	WNK1	1	0,00255588 8	0,0345196	c.446C>T	p.Ala149Val	Not present
**chr12:813797:G>A**	WNK1	1	0,00074293 4	0,00414766	c.915G>A	p.Thr305=	Not present
**chr12:15658097:C>T**	EPS8	1	0,00112832 6	0,0100865	c.1083G>A	p.Leu361=	Not present
**chr12:48832108:G>A**	DDX23	1	0,00156497 3	0,0159471	c.2034C>T	p.Asp678=	Present
**chr13:101726751:G>A**	FGF14	1	0,00038204 3	0,00518027	c.468C>T	p.Ile156=	Present

**chr14:58257912:C>T**	PSMA3	1	0,001196595	0,00736453	c.331-13C>T	Unknown	Not present
**chr14:100353743:G>A**	WARS1	1	0,000177985	0,000392773	c.669C>T	p.Ile223=	Not present
**chr15:87940635:A>G**	NTRK3	1	0,000873537	0,0132047	c.1704T>C	p.Leu568=	Present
**chr16:2097398:C>T**	PKD1	1	0,000416786	0,00281399	c.10326G>A	p.Ala3442=	Present
**chr16:8963226:G>A**	USP7	1	0,006801741	0,0483218	c.60C>T	p.Pro20=	Present
**chr16:51141987:C>G**	SALL1	1	0,003921227	0,0392044	c.235G>C	p.Ala79Pro	Not present
**chr17:1361226:G>A**	YWHAE	1	0,000175501	0,00145409	c.444C>T	p.Ser148=	Present
**chr5:139325661:T>DEL**	MATR3	1	0	0,001956	Unknown	Unknown	Not present
**chr22:27797470:A>G**	MN1	1	2,08503E-05	6,16257E-05	c.3074T>C	p.Ile1025Thr	Not present
**chr15:56096466:C>G**	RFX7	1	9,57598E-06	3,02865E-05	c.1262G>C	p.Gly421Ala	Present
**chr20:41404853:G>C**	CHD6	1	4,60953E-05	8,82094E-05	c.7888C>G	p.Pro2630Ala	Not present
**chr7:82902696:C>T**	PCLO	1	3,59991E-05	7,25689E-05	c.13483G>A	p.Ala4495Thr	Present
**chr1:118885330:C>T**	TBX15	1	3,32946E-05	0,000386847	c.1211G>A	p.Arg404Gln	Not present
**chr1:75143917:C>G**	LHX8	1	0	0	c.653C>G	p.Ala218Gly	Not present
**chr1:181796865:G>A**	CACNA1E	1	6,31059E-05	0,000770713	c.6399+7G>A	Unknown	Present
**chr4:103145116:T>G**	CENPE	1	0,004905022	0,00851466	c.4791A>C	p.Arg1597Ser	Not present
**chr4:166014510:G>A**	TLL1	1	2,8236E-05	0,000443927	c.992G>A	p.Arg331His	Not present
**chr6:16327687:C>CTGA**	ATXN1	1	0,00219797	0,00839798	c.623_624insTCA	p.Gln207_Gln208insHis	Not present
**chr7:98955087:C>T**	TRRAP	1	0,009430597	0,026751	c.5731-11C>T	Unknown	Present
**chr7:128849200:C>T**	FLNC	1	0,001331906	0,0193644	c.4947C>T	p.Gly1649=	Not present
**chr7:135638603:G>T**	NUP205	1	0,00264429	0,00411728	c.5312G>T	p.Ser1771Ile	Not present
**chr8:41694695:G>A**	ANK1	1	0,003910171	0,0450826	c.3224C>T	p.Thr1075Ile	Not present
**chr8:60801516:C>CT**	CHD7	1	0,001600957	0,0129585	c.2377-3dup	Unknown	Present
**chr9:35547592:T>G**	RUSC2	1	0,003374737	0,0127716	c.1071T>G	p.Pro357=	Not present
**chr9:130884330:C>T**	ABL1	1	0,002445594	0,00949427	c.2040C>T	p.Gly680=	Not present
**chr9:130884406:G>A**	ABL1	1	0,002482957	0,00949609	c.2116G>A	p.Gly706Ser	Not present
**chr9:130885515:G>A**	ABL1	1	4,60977E-05	8,28321E-05	c.3225G>A	p.Gln1075=	Not present
**chr9:138023668:G>C**	CACNA1B	1	0,006437119	0,0303644	c.2925G>C	p.Pro975=	Present
**chr5:132934888:G>A**	AFF4	1	0,000192207	0,00106166	c.177C>T	p.Tyr59=	Not present
**chr15:43082645:C>T**	UBR1	1	0,00022417	0,00531144	c.410G>A	p.Arg137His	Present
**chr7:97006076:A>ACAGCAG**	DLX6	1	0,000229289	0,0010553	c.117_122dup	p.Gln43_Gln44dup	Present
**chr11:4091302:C>T**	STIM1	1	1,7926E-05	0,00192864	c.1655C>T	p.Ser552Leu	Not present

**chr6:79002113:C>T**	PHIP	1	0	0	c.1665G>A	p.Gln555=	Present
**chr16:15724919:G>A**	MYH11	1	0,000113956	0,00117662	c.3932C>T	p.Ala1311Val	Not present
**chr10:100806541:G>A**	PAX2	1	0,000553153	0,00387684	c.728G>A	p.Arg243Gln	Not present
**chr4:113358818:G>C**	ANK2	1	1,02456E-05	3,76336E-05	c.10200G>C	p.Lys3400Asn	Present
**chr12:57177502:C>T**	LRP1	1	0	0	c.4272C>T	p.Arg1424=	Present
**chr20:46021797:C>G**	SLC12A5	1	4,1407E-05	0,00135777	c.32C>G	p.Pro11Arg	Present
**chr9:968008:G>C**	DMRT1	1	0,000244639	0,000434157	c.991G>C	p.Asp331His	Not present
**chr5:139314672:C>INS**	MATR3	1	0	0	Unknown	Unknown	Not present
**chr15:69421649:T>C**	KIF23	1	0,000695913	0,00144858	c.20T>C	p.Leu7Pro	Not present
**chr8:76851678:G>A**	ZFHX4	1	2,54573E-05	4,76554E-05	c.4757G>A	p.Ser1586Asn	Present
**chr6:56529740:T>C**	DST	1	7,85599E-06	2,93953E-05	c.17303A>G	p.His5768Arg	Present
**chr15:40645023:A>T**	KNL1	1	3,08028E-05	0,000478011	c.5825A>T	p.Asp1942Val	Not present
**chr6:87258959:C>T**	ZNF292	1	0,000483406	0,0020272	c.5330C>T	p.Ser1777Phe	Present
**chr20:36188376:C>T**	EPB41L1	1	0,00034711	0,00199501	c.903C>T	p.Gly301=	Not present
**chr15:69417401:C>A**	KIF23	1	0,000686467	0,00144998	c.100C>A	p.Pro34Thr	Not present
**chr4:103181448:A>G**	CENPE	1	1,74116E-06	3,02312E-06	c.972T>C	p.Ser324=	Not present
**chr11:27658307:G>A**	BDNF	1	3,58575E-05	0,000130976	c.258C>T	p.Asp86=	Not present
**chr10:129841045:C>CCCG**	EBF3	1	0,004767006	0,00726501	c.1373-14_1373-13insCGG	Unknown	Present
**chr12:31424702:T>C**	DENND5B	1	6,28034E-05	0,000482625	c.2239-15A>G	Unknown	Not present
**chr2:60460775:G>A**	BCL11A	1	6,37115E-06	1,91476E-05	c.2137C>T	p.Arg713Cys	Present
**chr6:157201351:C>T**	ARID1B	1	5,38341E-05	0,000102947	c.5126C>T	p.Thr1709Met	Present
**chr12:121810434:G>C**	SETD1B	1	2,23474E-05	4,41543E-05	c.1489G>C	p.Asp497His	Present
**chr11:31793654:G>A**	PAX6	1	2,04879E-05	0,000207211	c.956C>T	p.Pro319Leu	Present
**chr1:39387189:G>A**	MACF1	1	0,000207553	0,00193776	c.14347G>A	p.Asp4783Asn	Present
**chr12:79788730:C>T**	PPP1R12A	1	0,000125668	0,000631241	c.2720G>A	p.Arg907His	Not present
**chr14:95096431:C>T**	DICER1	1	2,06752E-05	6,04266E-05	c.4489G>A	p.Asp1497Asn	Not present
**chr3:50434412:C>G**	CACNA2D2	1	0,000234282	0,000988595	c.306G>C	p.Arg102=	Not present
**chr12:52517897:C>T**	KRT5	1	2,43288E-05	0,000207211	c.927G>A	p.Ala309=	Not present
**chr5:177211685:C>T**	NSD1	1	0,000206175	0,00120347	c.3286C>T	p.His1096Tyr	Present
**chr1:151441049:G>A**	POGZ	1	0,000235642	0,000477555	c.162C>T	p.Pro54=	Present
**chr16:72959452:G>A**	ZFHX3	1	5,8897E-05	9,06399E-05	c.694C>T	p.Arg232Cys	Present

**chr9:106925492:G>A**	ZNF462	1	4,73837E-05	0,00048216	c.1580G>A	p.Gly527Asp	Present
**chr4:182799723:C>T**	TENM3	1	5,27974E-06	9,31388E-05	c.7472C>T	p.Thr2491Met	Not present
**chr7:74515390:G>A**	GTF2IRD1	1	0,000100504	0,000207211	c.311G>A	p.Arg104Gln	Not present
**chr1:12322671:A>C**	VPS13D	1	5,1214E-06	6,54108E-05	c.7840A>C	p.Thr2614Pro	Not present
**chr20:41420527:G>A**	CHD6	1	0,000258243	0,00269151	c.6108C>T	p.Asp2036=	Not present
**chr1:12277610:C>T**	VPS13D	1	0,003174989	0,00485589	c.4022C>T	p.Ser1341Leu	Not present
**chr8:11749037:C>T**	GATA4	1	0	0	c.738C>T	p.His246=	Not present
**chr21:33549888:A>G**	SON	1	0,000806501	0,00993789	c.657A>G	p.Thr219=	Present
**chr10:77821493:A>C**	DLG5	1	0,004437517	0,00590698	c.2991T>G	p.Pro997=	Not present
**chr20:47651116:GCAGCAACAGCAA>G**	NCOA3	1	7,55828E-05	0,000566251	c.3804_3815del	p.Gln1273_Gln1276del	Not present
**chr12:47982950:C>T**	COL2A1	1	0,001507946	0,0267849	c.2095-4G>A	Unknown	Not present
**chr13:28034268:G>A**	FLT3	1	0	0	c.1704+33C>T	Unknown	Not present
**chr14:77512309:G>A**	SPTLC2	1	0,005033097	0,0275316	c.1664C>T	p.Thr555Met	Not present
**chr15:41092112:T>C**	INO80	1	0,001005244	0,0102364	c.452A>G	p.Asn151Ser	Not present
**chr15:72046381:A>G**	MYO9A	1	0,000139549	0,00094518	c.183T>C	p.Tyr61=	Not present
**chr16:3729328:C>T**	CREBBP	1	0,001722907	0,00686324	c.5719G>A	p.Ala1907Thr	Present
**chr16:24797969:AAG>A**	TNRC6A	1	3,98728E-05	7,45973E-05	c.3694+7_3694+8del	Unknown	Not present
**chr16:28834169:C>T**	ATXN2L	1	1,53657E-05	8,31716E-05	c.2130C>T	p.Tyr710=	Not present
**chr16:51141832:C>T**	SALL1	1	0,009777073	0,012739	c.390G>A	p.Pro130=	Not present
**chr17:17793335:A>G**	RAI1	1	4,74747E-05	0,000967586	c.387A>G	p.Pro129=	Present
**chr17:50192029:G>T**	COL1A1	1	0,003409622	0,0051875	c.1984-5C>A	Unknown	Not present
**chr18:21452171:T>C**	GREB1L	1	5,29568E-06	9,46122E-06	c.1938T>C	p.Cys646=	Not present
**chr18:22181476:G>A**	GATA6	1	3,18082E-06	5,71252E-06	c.1326G>A	p.Leu442=	Not present
**chr18:75286130:G>A**	TSHZ1	1	2,82101E-05	7,16435E-05	c.723G>A	p.Thr241=	Present
**chr19:35721614:C>CACAGCCACAGCT**	KMT2B	1	0,00129304	0,00236295	c.2268_2279dup	p.Gln759_Pro762dup	Present
**chr20:8740329:A>C**	PLCB1	1	0,005566244	0,0113997	c.2309-15A>C	Unknown	Present
**chr20:41512842:A>G**	CHD6	1	0,008593024	0,0237889	c.852+4T>C	Unknown	Not present
**chr1:21706810:T>C**	USP48	1	0,000180595	0,000604266	c.2022A>G	p.Lys674=	Not present
**chr1:35565516:C>T**	NCDN	1	0,009597437	0,0136711	c.2043C>T	p.Pro681=	Not present
**chr1:43966994:G>A**	IPO13	1	2,3058E-05	0,00024108	c.2588G>A	p.Arg863His	Not present
**chr1:81990481:G>T**	ADGRL2	1	0,001901467	0,00912106	c.3746G>T	p.Gly1249Val	Not present

**chr4:102566989:C>A**	NFKB1	1	0,000120407	0,00060184	c.261C>A	p.Ile87=	Not present
**chr4:182681938:C>T**	TENM3	1	0,001737859	0,019917	c.1959C>T	p.Ser653=	Not present
**chr5:150375440:G>A**	TCOF1	1	0,001847098	0,0134144	c.1590G>A	p.Gly530=	Not present
**chr5:180630744:C>T**	FLT4	1	0,000167334	0,00197874	c.211G>A	p.Gly71Arg	Not present
**chr6:7727550:C>G**	BMP6	1	0,000541022	0,00682947	c.595C>G	p.Pro199Ala	Not present
**chr6:56608118:G>T**	DST	1	0	0	c.6510C>A	p.Pro2170=	Present
**chr7:18656915:G>A**	HDAC9	1	1,97436E-05	0,000193648	c.1467G>A	p.Thr489=	Not present
**chr7:41964333:T>C**	GLI3	1	6,53019E-05	0,000731596	c.4740A>G	p.Gln1580=	Present
**chr7:74700401:C>G**	GTF2I	1	0	0	c.528C>G	p.Asn176Lys	Present
**chr7:78167992:A>G**	MAGI2	1	8,45181E-05	0,00048216	c.2520T>C	p.Tyr840=	Not present
**chr7:82954779:T>C**	PCLO	1	0,006383834	0,00986417	c.6174A>G	p.Leu2058=	Present
**chr7:98910604:T>C**	TRRAP	1	0,007047692	0,011194	c.1809T>C	p.Tyr603=	Present
**chr8:25886897:G>T**	EBF2	1	0,000171269	0,000529508	c.883-16C>A	Unknown	Not present
**chr8:140541215:G>A**	AGO2	1	0,003994005	0,0404284	c.1983C>T	p.Pro661=	Present
**chr9:106928151:G>A**	ZNF462	1	0,000119087	0,0031682	c.4239G>A	p.Val1413=	Present
**chr10:283269:C>T**	DIP2C	1	6,66462E-05	0,00024197	c.4294+3G>A	Unknown	Present
**chr10:21713803:G>A**	MLLT10	1	0,006117196	0,0172056	c.1731G>A	p.Ser577=	Not present
**chr4:81105941:TAGAG>T**	PRKG2	1	0,000640858	0,00435143	c.1941-10_1941-7del	Unknown	Not present
**chr4:10077753:C>T**	WDR1	1	0,006774995	0,0116804	c.1569G>A	p.Ser523=	Not present
**chr4:3199863:C>T**	HTT	1	0,000760482	0,00696295	c.5500C>T	p.Leu1834=	Not present
**chr1:224434204:C>CGGAGGAGGA**	WDR26	1	0,002139089	0,00469083	c.193_201dup	p.Ser65_Ser67dup	Present
**chr1:81990915:C>G**	ADGRL2	1	0,001698119	0,00771456	c.4180C>G	p.Pro1394Ala	Not present
**chr1:109252359:C>T**	CELSR2	1	1,11532E-05	3,02261E-05	c.2280C>T	p.Ile760=	Not present
**chr1:119987048:A>G**	NOTCH2	1	0,00017678	0,00083022	c.786T>C	p.Asp262=	Not present
**chr1:156791748:C>A**	PRCC	1	0,009037117	0,0254765	c.1135C>A	p.Pro379Thr	Not present
**chr1:175323363:C>A**	TNR	1	0,000389296	0,00167646	c.4071G>T	p.Gln1357His	Not present
**chr1:198702513:T>A**	PTPRC	1	0,000892345	0,00872518	c.566T>A	p.Ile189Asn	Present
**chr1:218405323:G>A**	TGFB2	1	0,000541785	0,00582363	c.501G>A	p.Glu167=	Not present
**chr2:36547007:T>G**	CRIM1	1	2,05397E-05	6,05364E-05	c.2770T>G	p.Tyr924Asp	Not present
**chr3:151190730:T>C**	MED12L	1	0,003806834	0,0223569	c.1767T>C	p.Ser589=	Present

**chr2:86089825:T>G**	POLR1A	1	0,003098957	0,00504278	c.537A>C	p.Ala179=	Not present
**chr2:108771695:T>G**	RANBP2	1	0,003214736	0,00800226	c.7850-6T>G	Unknown	Not present
**chr2:197392477:G>A**	SF3B1	1	0,003218047	0,0307527	c.3757-16C>T	Unknown	Present
**chr2:197402104:G>T**	SF3B1	1	0,004831118	0,0298755	c.2104C>A	p.Arg702=	Present
**chr2:197416735:A>T**	SF3B1	1	0,003069342	0,029826	c.666+6T>A	Unknown	Present
**chr3:105702188:C>G**	CBLB	1	0,006453017	0,0113308	c.1865G>C	p.Ser622Thr	Not present
**chr3:128481046:C>T**	GATA2	1	0,000238117	0,00111329	c.1416G>A	p.Pro472=	Not present
**chr12:117253693:C>T**	NOS1	1	5,24995E-05	0,000483092	c.2593G>A	p.Asp865Asn	Not present
**chr1:44636072:G>A**	RNF220	1	1,15237E-05	9,65111E-05	c.1036G>A	p.Glu346Lys	Not present
**chr11:96092210:TTGCTGCTGCTGCTGCTGCTGCTGCTGC>T**	MAML2	1	0,004739377	0,015497	c.1794_1820del	p.Gln613_Gln621del	Not present
**chr9:128694106:G>INS**	SET	1	0	0	Unknown	Unknown	Present
**chr6:24839089:A>INS**	RIPOR2	1	0	0	Unknown	Unknown	Not present
**chr6:31165628:C>A**	POU5F1	1	0,001152301	0,012264	c.600G>T	p.Leu200=	Not present
**chr6:87256319:C>T**	ZNF292	1	0,003447241	0,0362393	c.2690C>T	p.Ser897Leu	Present
**chr7:18458868:G>C**	HDAC9	1	0,004655178	0,0489413	c.52G>C	p.Val18Leu	Not present
**chr7:44701534:G>C**	OGDH	1	0	0	c.2560-9G>C	Unknown	Not present
**chr7:74084064:G>A**	LIMK1	1	0,000695757	0,00686119	c.55+19G>A	Unknown	Not present
**chr7:88153242:A>INS**	ADAM22	1	0	0	Unknown	Unknown	Not present
**chr7:97006051:G>GGCA**	DLX6	1	0,001153771	0,00543028	c.96_98dup	p.Gln44dup	Present
**chr7:103749506:T>INS**	RELN	1	0	0	Unknown	Unknown	Present
**chr7:105062285:G>T**	KMT2E	1	0	0	c.186+7G>T	Unknown	Present
**chr7:128845186:C>T**	FLNC	1	0,007257439	0,0132768	c.3721C>T	p.Arg1241Cys	Not present
**chr7:157253938:C>T**	UBE3C	1	0,001014555	0,0114945	c.2695-16C>T	Unknown	Present
**chr7:157253998:C>T**	UBE3C	1	0,001014546	0,0118993	c.2739C>T	p.Ser913=	Present
**chr8:86470216:A>INS**	WWP1, RMDN1	1	0	0	Unknown	Unknown	Not present
**chr8:94668132:A>INS**	ESRP1	1	0	0	Unknown	Unknown	Not present
**chr8:103766499:C>T**	RIMS2	1	2,54627E-05	0,000241196	c.792C>T	p.Gly264=	Present
**chr8:104251031:T>C**	RIMS2	1	0,005773139	0,0161989	c.4473T>C	p.Tyr1491=	Present
**chr9:35062235:G>A**	VCP	1	0,000758028	0,00904261	c.927C>T	p.Ile309=	Present
**chr9:35560263:TGGCCCG>T**	RUSC2	1	0,001698404	0,0211292	c.3632_3637del	p.Ala1211_Arg1212del	Not present
**chr6:13227984:C>T**	PHACTR1	1	0,000139605	0,00072324	c.1155C>T	p.Tyr385=	Not present
**chr5:180613079:A>G**	FLT4	1	0,000325451	0,00390095	c.3363T>C	p.Asn1121=	Not present

**chr5:180608994:G>A**	FLT4	1	0,002561883	0,0297276	c.3867C>T	p.Ser1289=	Not present
**chr4:147485814:C>G**	EDNRA	1	0	0	c.133C>G	p.Leu45Val	Not present
**chr4:2904964:C>T**	ADD1	1	0,00098462	0,0048216	c.1362C>T	p.Asp454=	Not present
**chr4:3211988:T>C**	HTT	1	0,001874299	0,0320275	c.6474T>C	p.Gly2158=	Not present
**chr4:3212138:G>A**	HTT	1	0,003159136	0,032187	c.6624G>A	p.Leu2208=	Not present
**chr4:3224037:G>A**	HTT	1	0,001878068	0,0320721	c.7671G>A	p.Glu2557=	Not present
**chr4:93238448:T>C**	GRID2	1	0,008588853	0,0138176	c.1203T>C	p.Leu401=	Present
**chr4:103108863:A>INS**	CENPE	1	0	0	Unknown	Unknown	Not present
**chr4:108165098:G>A**	LEF1	1	0,001402077	0,0166723	c.279C>T	p.Asp93=	Not present
**chr4:143514044:C>T**	SMARCA5	1	0,003032122	0,0337727	c.120C>T	p.Val40=	Not present
**chr5:45695854:G>T**	HCN1	1	0,000131954	0,00142586	c.240C>A	p.Gly80=	Present
**chr5:177248203:C>T**	NSD1	1	0,000581293	0,00656945	c.4520C>T	p.Thr1507Met	Present
**chr5:58990797:A>INS**	PDE4D	1	0	0	Unknown	Unknown	Not present
**chr5:59215980:G>A**	PDE4D	1	0,003241506	0,0366886	c.456-12C>T	Unknown	Not present
**chr5:59768348:G>C**	PDE4D	1	0,001071554	0,0126366	c.162C>G	p.Leu54=	Not present
**chr5:72195983:C>T**	MAP1B	1	0,000889798	0,0100573	c.2628C>T	p.Tyr876=	Present
**chr5:98882137:CAT>C**	CHD1	1	0,001517541	0,0159435	c.2719-16_2719-15del	Unknown	Present
**chr5:140114502:C>T**	PURA	1	0,000571929	0,00611482	c.321C>T	p.Ala107=	Not present
**chr5:150223135:G>A**	CAMK2A	1	0,001321319	0,00216068	c.1320C>T	p.Ile440=	Present
**chr5:169671094:A>INS**	DOCK2	1	0	0	Unknown	Unknown	Not present
**chr9:127816001:A>G**	ENG	1	0,008144253	0,044091	c.1794T>C	p.Gly598=	Not present
**chr9:130880663:C>T**	ABL1	1	0,002889113	0,0331407	c.1677C>T	p.Ser559=	Not present
**chr11:70807018:G>A**	SHANK2	1	0,000225729	0,00812408	c.1647C>T	p.Arg549=	Present
**chr9:137009984:C>A**	ABCA2	1	4,45608E-05	0,000384837	c.6494G>T	p.Arg2165Leu	Present
**chr12:57210294:G>A**	LRP1	1	1,77335E-06	3,18451E-06	c.12581-13G>A	Unknown	Present
**chr12:68813544:C>A**	MDM2	1	0,001694536	0,0207521	c.100-10C>A	Unknown	Not present
**chr12:114671931:T>C**	TBX3	1	0,003794894	0,0429906	c.2082A>G	p.Lys694=	Not present
**chr12:121810286:G>A**	SETD1B	1	0,000495502	0,0203831	c.1341G>A	p.Lys447=	Present
**chr12:132062551:G>A**	EP400	1	0,00251412	0,0387306	c.8184G>A	p.Gln2728=	Present
**chr13:35349144:T>C**	NBEA	1	0,001954494	0,0229583	c.5940T>C	p.Arg1980=	Present
**chr14:24151122:G>A**	RNF31	1	4,23115E-05	0,000477555	c.1489-9G>A	Unknown	Not present
**chr14:88594774:T>A**	ZC3H14	1	0,001075588	0,00814019	c.48T>A	p.Leu16=	Not present

**chr14:100734869:C>T**	DLK1	1	0,002419717	0,0257179	c.1125C>T	p.Ser375=	Not present
**chr15:51545684:A>INS**	DMXL2	1	0	0	Unknown	Unknown	Present
**chr15:63696138:A>INS**	HERC1	1	0	0	Unknown	Unknown	Present
**chr15:63727727:A>INS**	HERC1	1	0	0	Unknown	Unknown	Present
**chr15:69423294:A>G**	KIF23	1	0,002769178	0,00692384	c.699A>G	p.Leu233=	Not present
**chr15:71898763:T>C**	MYO9A	1	0,000985822	0,0138329	c.3740A>G	p.Gln1247Arg	Not present
**chr15:74410761:G>A**	SEMA7A	1	0,000568476	0,00305303	c.1864C>T	p.Arg622Cys	Not present
**chr15:90085111:G>A**	IDH2	1	0,00028195	0,0031084	c.1081-13C>T	Unknown	Not present
**chr16:1767854:C>T**	MAPK8IP3	1	0,005246049	0,0287976	c.3459C>T	p.Val1153=	Present
**chr16:2759621:TCTG>T**	SRRM2	1	0,000779869	0,00652666	c.797_799del	p.Ala266del	Present
**chr16:2769050:C>T**	SRRM2	1	0,000937226	0,00796477	c.7787C>T	p.Pro2596Leu	Present
**chr12:57194361:C>T**	LRP1	1	0,000747373	0,00711167	c.7926C>T	p.Thr2642=	Present
**chr12:57183854:C>T**	LRP1	1	0,005504242	0,0132594	c.5874C>T	p.Asp1958=	Present
**chr12:47976075:AAG>A**	COL2A1	1	6,40251E-05	0,00024108	c.3490-7_3490-6del	Unknown	Not present
**chr11:61776847:C>T**	MYRF	1	0,001010255	0,0158593	c.1560C>T	p.Ala520=	Not present
**chr9:137716856:C>G**	EHMT1	1	0,001385585	0,0161655	c.316C>G	p.Gln106Glu	Present
**chr9:137986833:C>T**	CACNA1B	1	0,001039791	0,0120995	c.1953C>T	p.Ala651=	Present
**chr10:60139028:A>G**	ANK3	1	0,000786476	0,00866168	c.2674T>C	p.Leu892=	Present
**chr10:69394956:G>A**	HK1	1	0,000321354	0,00298263	c.2226G>A	p.Glu742=	Not present
**chr10:74103843:A>T**	VCL	1	6,5293E-05	0,0012054	c.2046A>T	p.Leu682Phe	Not present
**chr10:100747179:C>T**	PAX2	1	0,001083402	0,00380155	c.301C>T	p.Leu101Phe	Not present
**chr10:117318884:A>INS**	PDZD8	1	0	0	Unknown	Unknown	Present
**chr11:8712713:G>A**	DENND2B	1	0,00488685	0,0248279	c.2010C>T	p.His670=	Present
**chr11:68158626:C>T**	KMT5B	1	0,000533906	0,00477178	c.1720G>A	p.Asp574Asn	Present
**chr12:6935675:C>T**	ATN1	1	0,005120988	0,00828014	c.408C>T	p.Tyr136=	Not present
**chr11:78732560:T>C**	TENM4	1	0,005644389	0,0236448	c.2894A>G	p.Asn965Ser	Not present
**chr11:84166954:A>INS**	DLG2	1	0	0	Unknown	Unknown	Present
**chr11:105974300:TC>T**	GRIA4	1	5,76997E-05	0,000946074	c.2410-4del	Unknown	Not present
**chr11:108185248:T>G**	NPAT	1	6,29015E-05	0,00120656	c.890A>C	p.Glu297Ala	Not present
**chr11:114250528:G>A**	ZBTB16	1	0,000235581	0,0022619	c.1995G>A	p.Lys665=	Present
**chr12:754062:T>A**	WNK1	1	0	0	c.497T>A	p.Val166Glu	Not present
**chr12:894638:A>G**	WNK1	1	0,000855393	0,00944143	c.5583+3A>G	Unknown	Not present
**chr12:896478:G>A**	WNK1	1	0,0023566	0,0276365	c.5991G>A	p.Glu1997=	Not present
**chr4:2904796:T>A**	ADD1	1	0,000979363	0,0048216	c.1194T>A	p.Pro398=	Not present

**chr4:2898279:T>C**	ADD1	1	0,000978155	0,0048216	c.837T>C	p.Asp279=	Not present
**chr4:2875960:G>T**	ADD1	1	0,001039354	0,00507001	c.45G>T	p.Pro15=	Not present
**chr4:1978711:C>T**	NSD2	1	0,002870554	0,0343054	c.3900C>T	p.Pro1300=	Present
**chr5:138381576:G>INS**	KDM3B	1	0	0	Unknown	Unknown	Present
**chr3:193657175:T>INS**	OPA1	1	0	0	Unknown	Unknown	Not present
**chr5:83545554:A>INS**	VCAN, VCAN-AS1	1	0	0	Unknown	Unknown	Not present
**chr9:81652231:A>INS**	TLE1	1	0	0	Unknown	Unknown	Not present
**chr14:101762935:A>INS**	PPP2R5C	1	0	0	Unknown	Unknown	Present
**chr6:7576294:G>INS**	DSP	1	0	0	Unknown	Unknown	Not present
**chr9:131174301:G>A**	NUP214	1	0,000219392	0,00197315	c.3140G>A	p.Gly1047Asp	Not present
**chr3:25624301:A>INS**	TOP2B	1	0	0	Unknown	Unknown	Present
**chr2:61496920:A>INS**	XPO1	1	0	0	Unknown	Unknown	Present
**chr15:92980818:A>INS**	CHD2	1	0	0	Unknown	Unknown	Present
**chr6:73194609:A>INS**	KCNQ5	1	0	0	Unknown	Unknown	Not present
**chr1:81986928:A>INS**	ADGRL2	1	0	0	Unknown	Unknown	Not present
**chr19:17632876:C>T**	UNC13A	1	3,33017E-05	0,000114312	c.3334G>A	p.Asp1112Asn	Present
**chr1:243505261:A>INS**	AKT3	1	0	0	Unknown	Unknown	Not present
**chr4:48556988:A>INS**	FRYL	1	0	0	Unknown	Unknown	Present
**chr10:10920008:G>INS**	CELF2	1	0	0	Unknown	Unknown	Present
**chr1:39453773:A>INS**	MACF1	1	0	0	Unknown	Unknown	Present
**chr2:39007036:A>INS**	1 SOS	1	0	0	Unknown	Unknown	Not present
**chr15:48610731:A>INS**	FBN1	1	0	0	Unknown	Unknown	Present
**chr9:128582730:A>INS**	SPTAN1	1	0	0	Unknown	Unknown	Not present
**chr16:24092827:A>INS**	PRKCB	1	0	0	Unknown	Unknown	Present
**chr14:35011523:A>INS**	SRP54	1	0	0	Unknown	Unknown	Not present
**chr5:139326196:A>INS**	MATR3	1	0	0	Unknown	Unknown	Not present
**chr20:45947608:G>A**	PCIF1	1	0,001718072	0,0189736	c.1968G>A	p.Thr656=	Not present
**chr20:47636698:G>A**	NCOA3	1	0	0	c.2312G>A	p.Ser771Asn	Not present
**chr20:51562586:A>G**	NFATC2	1	1,53172E-05	0,000201636	c.44T>C	p.Leu15Pro	Not present
**chr21:39210928:A>T**	BRWD1	1	5,4099E-05	0,000523903	c.3902T>A	p.Val1301Asp	Not present
**chr7:155803525:A>T**	SHH	1	0	0	c.764T>A	p.Ile255Asn	Not present
**chr12:119834087:G>A**	CIT	1	3,23305E-05	0,000335366	c.658C>T	p.Arg220Ter	Not present
**chr5:139317605:A>INS**	MATR3	1	0	0	Unknown	Unknown	Not present
**chr5:139326188:A>INS**	MATR3	1	0	0	Unknown	Unknown	Not present
**chr6:43547632:A>INS**	XPO5, POLR1C	1	0	0	Unknown	Unknown	Not present
**chr16:30724253:C>T**	SRCAP	1	1,79278E-05	0,000288018	c.4829C>T	p.Pro1610Leu	Present
**chr6:44446591:A>INS**	CDC5L, POLR1C	1	0	0	Unknown	Unknown	Not present
**chr4:110621310:T>A**	PITX2	1	0	0	c.265A>T	p.Lys89Ter	Not present
**chr18:22200707:A>G**	GATA6	1	1,6643E-05	0,000168342	c.1672A>G	p.Ser558Gly	Not present
**chr3:129171101:A>INS**	CNBP	1	0	0	Unknown	Unknown	Not present
**chr5:138887605:C>G**	CTNNA1	1	0	0	c.1259C>G	p.Ala420Gly	Not present
**chr19:46919924:G>A**	ARHGAP35	1	3,18171E-06	2,28624E-05	c.1249G>A	p.Glu417Lys	Not present
**chr15:36895155:A>INS**	MEIS2	1	0	0	Unknown	Unknown	Present

**chr15:38322324:G>INS**	SPRED1	1	0	0	Unknown	Unknown	Not present
**chr6:163563584:A>INS**	QKI	1	0	0	Unknown	Unknown	Not present
**chr1:9723152:C>T**	PIK3CD	1	0,000140909	0,00047259	c.2454C>T	p.Thr818=	Not present
**chr1:10106409:C>T**	UBE4B	1	0,00020243	0,0016226	c.1022C>T	p.Ser341Phe	Not present
**chr3:51275189:G>A**	DOCK3	1	2,43366E-05	0,000130787	c.2659G>A	p.Val887Ile	Not present
**chr2:169257160:G>C**	LRP2	1	0,00099633	0,00612245	c.2603C>G	p.Thr868Ser	Present
**chr2:207081194:A>INS**	KLF7	1	0	0	Unknown	Unknown	Present
**chr2:215433311:A>INS**	FN1	1	0	0	Unknown	Unknown	Not present
**chr3:25624775:T>C**	TOP2B	1	0,003943001	0,0448749	c.2253A>G	p.Leu751=	Present
**chr3:38605987:G>A**	SCN5A	1	0,003660578	0,0434009	c.1302C>T	p.Phe434=	Not present
**chr3:48662252:C>A**	CELSR3	1	0,00242037	0,0289576	c.383G>T	p.Gly128Val	Not present
**chr3:49510793:A>G**	DAG1	1	0,008137316	0,0193063	c.259A>G	p.Ile87Val	Not present
**chr3:50366615:T>C**	CACNA2D2	1	2,17879E-05	0,000723589	c.2600A>G	p.Asn867Ser	Not present
**chr3:52402800:T>G**	BAP1	1	0,000281687	0,00432526	c.1962A>C	p.Val654=	Not present
**chr2:135722542:C>T**	R3HDM1	1	0,001566345	0,024192	c.3038C>T	p.Ala1013Val	Not present
**chr3:69794486:G>A**	MITF	1	2,62978E-05	0,000096567	c.24G>A	p.Val8=	Not present
**chr3:77565128:C>G**	ROBO2	1	0,001696766	0,02	c.1861+8C>G	Unknown	Present
**chr3:77574601:C>T**	ROBO2	1	0,000857374	0,00900554	c.2086C>T	p.Pro696Ser	Present
**chr3:101326049:T>C**	SENP7	1	0,002171858	0,024781	c.3047A>G	p.His1016Arg	Not present
**chr3:128629938:A>T**	RPN1	1	0	0	c.1036+13T>A	Unknown	Not present
**chr3:132505290:A>INS**	DNAJC13	1	0	0	Unknown	Unknown	Not present
**chr3:197065792:C>A**	DLG1	1	0,001147352	0,00242483	c.2116G>T	p.Val706Leu	Present
**chr3:197081057:A>C**	DLG1	1	0,00858669	0,0129845	c.1899T>G	p.Asp633Glu	Present
**chr2:149011616:C>T**	KIF5C	1	0,000274025	0,00662824	c.2814C>T	p.Ala938=	Present
**chr2:120989430:G>A**	GLI2	1	7,43603E-05	0,00094697	c.3465G>A	p.Ala1155=	Not present
**chr1:18634523:C>T**	PAX7	1	6,27652E-05	0,000282582	c.306C>T	p.Gly102=	Not present
**chr1:198728362:A>INS**	PTPRC	1	0	0	Unknown	Unknown	Present
**chr1:22864978:G>A**	EPHB2	1	0,000109571	0,00124378	c.1069G>A	p.Val357Ile	Present
**chr1:103001923:A>INS**	COL11A1	1	0	0	Unknown	Unknown	Not present
**chr1:103005903:T>INS**	COL11A1	1	0	0	Unknown	Unknown	Not present
**chr1:103005906:T>INS**	COL11A1	1	0	0	Unknown	Unknown	Not present
**chr1:155951145:G>C**	ARHGEF2	1	3,23171E-06	3,05474E-05	c.2387C>G	p.Ala796Gly	Present
**chr1:156134501:G>A**	LMNA	1	0,009559987	0,0275476	c.612G>A	p.Leu204=	Not present
**chr1:160293170:A>INS**	COPA	1	0	0	Unknown	Unknown	Not present
**chr1:169127296:A>INS**	ATP1B1	1	0	0	Unknown	Unknown	Not present
**chr1:204549224:C>T**	MDM4	1	5,12197E-06	0,000477555	c.1015C>T	p.His339Tyr	Not present
**chr2:120989263:G>T**	GLI2	1	0,000608973	0,00726346	c.3298G>T	p.Val1100Leu	Not present

**chr1:231336773:C>T**	EXOC8	1	0,002340361	0,0274783	c.973G>A	p.Val325Ile	Not present
**chr1:237718469:C>INS**	RYR2	1	0	0	Unknown	Unknown	Not present
**chr1:237778725:C>T**	RYR2	1	0,002961365	0,0243022	c.11835C>T	p.Val3945=	Not present
**chr1:244055301:C>T**	ZBTB18	1	6,15243E-05	0,000508647	c.1527C>T	p.Ser509=	Present
**chr2:27242660:A>INS**	CAD	1	0	0	Unknown	Unknown	Not present
**chr2:51027773:G>C**	NRXN1	1	0,000558145	0,00430741	c.501C>G	p.Leu167=	Present
**chr2:55881745:C>T**	EFEMP1	1	0,003181344	0,0351093	c.518-11G>A	Unknown	Not present
**chr2:111825043:G>A**	ANAPC1	1	0,000872263	0,010518	c.2742-7C>T	Unknown	Not present
**chr16:49638864:G>A**	ZNF423	1	0,002927496	0,0181607	c.312C>T	p.Asp104=	Not present
**chr16:69691794:T>G**	NFAT5	1	0,002220265	0,00857796	c.1969T>G	p.Ser657Ala	Not present
**chr16:81921184:C>INS**	PLCG2	1	0	0	Unknown	Unknown	Not present
**chr6:56648591:A>C**	DST	1	4,51842E-05	8,68851E-05	c.1533T>G	p.Pro511=	Present
**chr16:3729593:C>T**	CREBBP	1	0,008472732	0,0172609	c.5454G>A	p.Val1818=	Present
**chr16:30739152:T>C**	SRCAP	1	0,000746513	0,00785577	c.9112T>C	p.Leu3038=	Present
**chr17:6478625:G>A**	PITPNM3	1	0,007961245	0,0146641	c.699C>T	p.Val233=	Not present
**chr17:17797460:G>T**	RAI1	1	0,005980119	0,013828	c.4512G>T	p.Leu1504=	Present
**chr19:2228330:G>T**	DOT1L	1	0	0	c.5200G>T	p.Gly1734Trp	Present
**chr19:8586554:T>C**	ADAMTS10	1	0,006380591	0,0177295	c.2403+4A>G	Unknown	Not present
**chr19:8595891:C>A**	ADAMTS10	1	0,003538853	0,0391093	c.1350G>T	p.Gly450=	Not present
**chr19:42286904:C>T**	CIC	1	8,34855E-05	0,000385004	c.2928C>T	p.Ala976=	Present
**chr20:8717804:A>G**	PLCB1	1	0,004123001	0,00643834	c.1469A>G	p.Tyr490Cys	Present
**chr20:48941880:C>T**	ARFGEF2	1	0,006107787	0,00954568	c.169C>T	p.Pro57Ser	Not present
**chr20:51601223:C>T**	ATP9A	1	1,59445E-06	2,86311E-06	c.3132G>A	p.Lys1044=	Present
**chr17:63420415:C>G**	TANC2	1	0	0	c.4685C>G	p.Thr1562Ser	Present
**chr16:56354872:A>G**	GNAO1	1	7,69314E-06	2,28749E-05	c.884A>G	p.Asn295Ser	Not present
**chr19:10987779:G>A**	SMARCA4	1	1,17482E-05	6,56944E-05	c.973G>A	p.Val325Met	Present
**chr17:61982727:A>G**	MED13	1	0	0	c.3276T>C	p.Ser1092=	Present
**chr1:6146865:G>C**	CHD5	1	2,51204E-05	5,88183E-05	c.1390C>G	p.Pro464Ala	Not present
**chr1:8360377:G>T**	RERE	1	4,87654E-05	0,000106107	c.3130C>A	p.Pro1044Thr	Present
**chr2:112542424:T>TTCCGGCGTGTACCGAGAGACTGGCG**	POLR1B	1	0,000248828	0,00168705	c.75_99dup	p.Cys34ArgfsTer28	Not present
**chr3:167789351:T>G**	SERPINI1	1	7,95277E-06	1,42828E-05	c.223T>G	p.Ser75Ala	Not present
**chr15:88845689:G>A**	ACAN	1	0,007807338	0,013245	c.1236G>A	p.Leu412=	Not present

**chr15:66490486:G>A**	MAP2K1	1	0,000229216	0,00250494	c.1069-16G>A	Unknown	Not present
**chr15:66387416:C>T**	MAP2K1	1	0,000162521	0,00180488	c.69C>T	p.Thr23=	Not present
**chr9:134811500:C>T**	COL5A1	1	0,000310365	0,000602604	c.3591C>T	p.Asp1197=	Not present
**chr6:156778889:CGGAGGA>C**	ARID1B	1	0,003172226	0,0186946	c.1229_1234del	p.Gly410_Gly411del	Present
**chr6:170572229:C>T**	TBP	1	0,000896388	0,00899765	c.984C>T	p.Ile328=	Not present
**chr7:128835339:C>T**	FLNC	1	5,63702E-05	0,000142966	c.366C>T	p.Ile122=	Not present
**chr8:41661508:C>T**	ANK1	1	0,000245853	0,00144648	c.5601G>A	p.Ala1867=	Not present
**chr8:132134369:G>A**	KCNQ3	1	0,002426675	0,00530035	c.1720C>T	p.Pro574Ser	Present
**chr9:92715277:C>T**	BICD2	1	0,003392583	0,00582267	c.2445G>A	p.Pro815=	Not present
**chr9:125326495:C>T**	GAPVD1	1	0,000951266	0,0015567	c.1938C>T	p.Asp646=	Not present
**chr9:127826641:G>A**	ENG	1	0,003905339	0,0277778	c.392C>T	p.Pro131Leu	Not present
**chr10:8058636:C>T**	GATA3	1	0,008429625	0,0356798	c.573C>T	p.Pro191=	Not present
**chr15:41749162:C>T**	MGA	1	2,30545E-05	0,000207211	c.5702C>T	p.Pro1901Leu	Not present
**chr10:29533429:T>C**	SVIL	1	0,004434919	0,0261411	c.938A>G	p.Glu313Gly	Not present
**chr10:77817068:C>T**	DLG5	1	0,003595179	0,0196854	c.3813G>A	p.Ala1271=	Not present
**chr10:77856873:C>T**	DLG5	1	0,004876927	0,0294355	c.393G>A	p.Pro131=	Not present
**chr11:27658292:C>T**	BDNF	1	0,000181825	0,00072324	c.273G>A	p.Thr91=	Not present
**chr11:66705772:G>A**	SPTBN2	1	0,00054787	0,0286885	c.1719C>T	p.His573=	Not present
**chr11:105903797:C>T**	GRIA4	1	6,43524E-06	1,46985E-05	c.886-17C>T	Unknown	Not present
**chr14:22845338:C>A**	MMP14	1	6,42134E-06	2,77469E-05	c.1389C>A	p.Pro463=	Not present
**chr15:40985353:T>C**	INO80	1	0,0075595	0,0118659	c.3906A>G	p.Glu1302=	Not present
**chr19:40265243:C>T**	AKT2	1	7,96191E-06	1,42921E-05	c.25G>A	p.Glu9Lys	Not present
**chr12:57175500:C>T**	LRP1	1	0,000161351	0,000294023	c.3588C>T	p.Cys1196=	Present
**chr12:57209167:C>G**	LRP1	1	0,000490494	0,000859813	c.12230C>G	p.Thr4077Arg	Present
**chr9:89405376:T>C**	SEMA4D	1	0,001009071	0,00156562	c.81A>G	p.Ile27Met	Not present
**chr4:87047363:G>T**	AFF1	1	0,00382347	0,0167988	c.828G>T	p.Pro276=	Not present
**chr4:87114755:C>T**	AFF1	1	0,004181382	0,0219489	c.1922C>T	p.Ser641Phe	Not present
**chr4:125490370:G>A**	FAT4	1	0,002906441	0,0113308	c.13554G>A	p.Leu4518=	Not present
**chr5:38957718:A>T**	RICTOR	1	0,008314637	0,0114671	c.2433T>A	p.Ile811=	Present
**chr7:116771936:C>T**	MET	1	0,009890135	0,0215997	c.2975C>T	p.Thr992Ile	Present

**chr7:152177496:G>C**	KMT2C	1	0,007676491	0,0205103	c.7957C>G	p.Leu2653Val	Present
**chr7:152177962:G>A**	KMT2C	1	0,00779102	0,0239288	c.7491C>T	p.Arg2497=	Present
**chr9:81634161:C>A**	TLE1	1	5,97679E-05	0,00144009	c.513G>T	p.Leu171=	Not present
**chr9:95516676:T>TTTC**	PTCH1	1	0,000935174	0,00963402	c.142_144dup	p.Glu48dup	Not present
**chr2:190983687:A>G**	STAT1	1	1,59043E-06	2,85636E-06	c.1401T>C	p.Gly467=	Not present
**chr9:126695879:C>T**	LMX1B	1	3,1844E-06	5,7152E-06	c.927C>T	p.Tyr309=	Present
**chr9:137017119:G>A**	ABCA2	1	0,002695636	0,0154366	c.2559C>T	p.Leu853=	Present
**chr10:63214130:A>G**	JMJD1C	1	0,001028066	0,0096432	c.2037T>C	p.His679=	Present
**chr10:102398285:T>C**	NFKB2	1	4,48184E-05	0,00024108	c.840T>C	p.Asp280=	Not present
**chr10:121565655:C>T**	FGFR2	1	0,003666699	0,0105461	c.159G>A	p.Ala53=	Not present
**chr11:57809347:G>A**	CTNND1	1	0,000112688	0,000702026	c.2316G>A	p.Glu772=	Present
**chr11:64266310:T>C**	PLCB3	1	0,000166855	0,000285636	c.3267-5T>C	Unknown	Not present
**chr11:65344611:G>A**	DPF2	1	2,4196E-05	0,000207211	c.669G>A	p.Ala223=	Not present
**chr2:215431981:G>A**	FN1	1	0,0028362	0,0167636	c.416-17C>T	Unknown	Not present
**chr2:159349268:C>G**	BAZ2B	1	0,001515124	0,0358076	c.4876G>C	p.Val1626Leu	Present
**chr12:112162497:C>A**	HECTD4	1	0	0	c.13147G>T	p.Val4383Leu	Present
**chr16:85911630:G>A**	IRF8	1	3,8421E-05	0,000482859	c.419G>A	p.Arg140His	Not present
**chr12:54283136:G>T**	HNRNPA1	1	7,95851E-06	3,76492E-05	c.809G>T	p.Gly270Val	Not present
**chr11:45902701:G>A**	MAPK8IP1	1	3,21793E-05	6,86373E-05	c.934G>A	p.Asp312Asn	Present
**chr3:123447728:A>G**	ADCY5	1	0,000340201	0,00334608	c.818T>C	p.Val273Ala	Present
**chr10:77796591:G>A**	DLG5	1	1,40914E-05	7,16456E-05	c.5168C>T	p.Ser1723Leu	Not present
**chr4:54727271:G>A**	KIT	1	0,000172846	0,000723589	c.1594G>A	p.Val532Ile	Not present
**chr3:50377486:C>T**	CACNA2D2	1	0,000111546	0,000191397	c.1607G>A	p.Arg536Lys	Not present
**chr2:215375319:G>A**	FN1	1	5,12215E-05	0,00024108	c.6052C>T	p.Arg2018Cys	Not present
**chr22:40418873:T>A**	MRTFA	1	7,97214E-06	1,42882E-05	c.1865A>T	p.Asp622Val	Not present
**chr4:125318213:A>G**	FAT4	1	8,32164E-05	0,00047259	c.1802A>G	p.Glu601Gly	Not present
**chr2:144399046:G>A**	ZEB2	1	0,002974506	0,00998869	c.2141C>T	p.Pro714Leu	Not present
**chr12:102958393:C>CGCAGCAGCAGCAGCAGCAGCA**	ASCL1	1	0,000322854	0,00258574	c.166_186dup	p.Gln56_Gln62dup	Not present
**chr1:26697417:GGCTGCGGCGGCGGCA>G**	ARID1A	1	0,000853532	0,002079	c.1029_1043del	p.Ala345_Ala349del	Present
**chr1:38852437:C>T**	RRAGC	1	0	0	c.693G>A	p.Lys231=	Not present
**chr1:42929946:G>A**	SLC2A1	1	2,81705E-05	0,00072324	c.606C>T	p.Ile202=	Not present

**chr1:204974186:G>A**	NFASC	1	0,000163997	0,00168837	c.1287G>A	p.Pro429=	Not present
**chr1:237441447:C>T**	RYR2	1	0,000790777	0,00129412	c.1134C>T	p.Asp378=	Not present
**chr1:237723192:A>G**	RYR2	1	0	0	c.10619A>G	p.Asp3540Gly	Not present
**chr2:27241897:T>C**	CAD	1	3,86192E-06	1,47089E-05	c.5884-14T>C	Unknown	Not present
**chr6:146159604:T>TTCTCTCTC**	GRM1	1	0,000391534	0,0012087	c.950+38_950+45dup	Unknown	Not present
**chr5:147453561:G>T**	DPYSL3	1	0,007914754	0,0147419	c.27C>A	p.Ile9=	Present
**chr17:7899154:A>G**	CHD3	1	0,003487675	0,0404657	c.2295A>G	p.Leu765=	Present
**chr5:141527699:T>TAAAAA**	DIAPH1	1	0,000760961	0,00344116	c.3149-7_3149-3dup	Unknown	Not present
**chr20:44429627:A>G**	HNF4A	1	0,000466247	0,0122951	c.1321A>G	p.Ile441Val	Not present
**chr20:46367406:A>T**	ELMO2	1	0,000193694	0,000734035	c.2117T>A	p.Ile706Asn	Not present
**chr20:47651113:GCAGCAGCAA>G**	NCOA3	1	0,00277136	0,0119197	c.3789_3797del	p.Gln1274_Gln1276del	Not present
**chr20:49374378:C>T**	KCNB1	1	0,002088724	0,0153159	c.1182G>A	p.Gly394=	Present
**chr20:51516892:G>A**	NFATC2	1	0,003558058	0,00757576	c.1224C>T	p.Tyr408=	Not present
**chr20:59892092:G>A**	SYCP2	1	0,002630997	0,00388899	c.2262C>T	p.Cys754=	Not present
**chr21:39255651:A>INS**	BRWD1	1	0	0	Unknown	Unknown	Not present
**chr22:19353405:G>A**	HIRA	1	0,003098734	0,026432	c.2799C>T	p.His933=	Not present
**chr22:30932383:T>C**	MORC2	1	0	0	c.2817A>G	p.Ser939=	Not present
**chr22:36282736:C>T**	MYH9	1	0,001593479	0,0159941	c.5815G>A	p.Ala1939Thr	Present
**chr22:50454681:G>A**	SBF1	1	8,63884E-05	0,000613155	c.4874C>T	p.Thr1625Met	Present
**chr22:50694818:G>A**	SHANK3	1	0,00153331	0,0135661	c.1299G>A	p.Leu433=	Present
**chr22:50694989:C>T**	SHANK3	1	0,001524224	0,0135044	c.1470C>T	p.Ala490=	Present
**chr10:79208421:T>C**	ZMIZ1	1	0	0	c.146T>C	p.Phe49Ser	Present
**chr16:30729134:C>T**	SRCAP	1	7,68285E-06	0,000113033	c.5827C>T	p.Pro1943Ser	Present
**chr22:30932406:T>G**	MORC2	1	1,59043E-06	2,28655E-05	c.2794A>C	p.Lys932Gln	Not present
**chr8:67259842:G>T**	ARFGEF1	1	1,79571E-05	6,05583E-05	c.2208C>A	p.Phe736Leu	Not present
**chr2:135649966:C>T**	R3HDM1	1	1,51455E-05	7,52615E-05	c.1688C>T	p.Pro563Leu	Not present
**chr5:98858344:CCTG>C**	CHD1	1	5,3855E-05	0,0011534	c.4620_4622del	p.Ser1540del	Present
**chr20:8697790:T>C**	PLCB1	1	0,001421641	0,0157169	c.1167+7T>C	Unknown	Present
**chr20:8646175:A>T**	PLCB1	1	0,001949979	0,0230466	c.458A>T	p.Glu153Val	Present
**chr19:50662661:G>A**	SHANK1	1	0,000962938	0,00419132	c.5790C>T	p.Ser1930=	Present
**chr17:82249444:A>T**	CSNK1D	1	0	0	c.1044T>A	p.Pro348=	Not present
**chr17:7900582:G>A**	CHD3	1	0,003472133	0,0400837	c.2829G>A	p.Glu943=	Present
**chr17:17793673:C>T**	RAI1	1	0,00300119	0,0294118	c.725C>T	p.Pro242Leu	Present

**chr17:36942855:G>A**	LHX1	1	0,000451693	0,00488043	c.945G>A	p.Pro315=	Not present
**chr17:37181363:G>GA**	ACACA	1	0,003749938	0,0403049	c.4777-8dup	Unknown	Not present
**chr17:37248681:G>GAAC**	ACACA	1	0,003647016	0,0391042	c.2082-10_2082-8dup	Unknown	Not present
**chr17:42130455:C>T**	RAB5C	1	0,007728236	0,0441958	c.48G>A	p.Gly16=	Not present
**chr17:46066559:C>T**	KANSL1	1	0,00053576	0,00435536	c.1826G>A	p.Ser609Asn	Present
**chr17:63693594:G>A**	MAP3K3	1	0,003054843	0,0307848	c.1698G>A	p.Pro566=	Not present
**chr18:53066062:G>A**	DCC	1	0,000276948	0,00313858	c.1157G>A	p.Arg386Gln	Present
**chr19:41905040:A>AG**	ARHGEF1	1	0,000445626	0,00394705	c.2249+11dup	Unknown	Not present
**chr18:75288176:G>A**	TSHZ1	1	0,004009997	0,0462304	c.2769G>A	p.Pro923=	Present
**chr19:3976601:G>A**	EEF2	1	0,00115725	0,0133878	c.2530C>T	p.Leu844=	Not present
**chr19:4941920:C>T**	UHRF1	1	0,003549114	0,0378475	c.1062C>T	p.Ser354=	Not present
**chr19:4945855:C>T**	UHRF1	1	0,004357007	0,0494944	c.1306-6C>T	Unknown	Not present
**chr19:17656305:C>A**	UNC13A	1	0,000492208	0,00220696	c.861G>T	p.Leu287=	Present
**chr19:18765482:G>A**	CRTC1	1	0,000809792	0,00590584	c.965G>A	p.Arg322His	Not present
**chr19:41898460:G>T**	ARHGEF1	1	0,00038653	0,00365437	c.1140G>T	p.Glu380Asp	Not present
**chr19:41902293:G>A**	ARHGEF1	1	0,000457043	0,00461805	c.1434G>A	p.Leu478=	Not present
**chr4:3115406:A>G**	HTT	1	4,86493E-05	0,00192864	c.850A>G	p.Thr284Ala	Not present
**chr12:861179:C>T**	WNK1	1	3,33016E-05	5,65227E-05	c.1787C>T	p.Ser596Phe	Not present
**chr2:113235441:T>C**	PAX8	1	2,83652E-05	0,00094697	c.1040A>G	p.His347Arg	Not present
**chr3:181712603:T>G**	SOX2	1	0,000240735	0,00192864	c.243T>G	p.Leu81=	Not present
**chr2:25743113:C>T**	ASXL2	1	0,00901442	0,020974	c.3224G>A	p.Arg1075Gln	Not present
**chr2:27238500:T>C**	CAD	1	0,005817428	0,0088787	c.4930T>C	p.Phe1644Leu	Not present
**chr2:96289939:T>C**	SNRNP200	1	0,002051537	0,0166182	c.2800A>G	p.Thr934Ala	Not present
**chr2:96297656:T>G**	SNRNP200	1	4,77113E-06	8,56883E-06	c.1184A>C	p.Asp395Ala	Not present
**chr2:111831398:G>A**	ANAPC1	1	0,009641264	0,0255701	c.2513C>T	p.Thr838Met	Not present
**chr2:159385274:T>C**	BAZ2B	1	0,001055736	0,00288018	c.3567A>G	p.Gly1189=	Present
**chr2:240739686:G>A**	KIF1A	1	0,002173628	0,00372015	c.3599-17C>T	Unknown	Present
**chr3:129563087:G>A**	PLXND1	1	0,000371655	0,00367564	c.4668+7C>T	Unknown	Not present
**chr4:2908581:G>A**	ADD1	1	0,003470498	0,0113033	c.1675G>A	p.Val559Met	Not present
**chr1:109270029:C>G**	CELSR2	1	7,68327E-06	2,93988E-05	c.7204C>G	p.Arg2402Gly	Not present
**chr4:3130311:A>G**	HTT	1	0,005279031	0,0183358	c.1874A>G	p.Gln625Arg	Not present

**chr4:3235649:C>T**	HTT	1	0,002095346	0,0123427	c.8656C>T	p.Leu2886=	Not present
**chr4:3238940:C>T**	HTT	1	0,006243099	0,0182565	c.9177C>T	p.Phe3059=	Not present
**chr4:76740408:G>A**	SHROOM3	1	1,66708E-05	4,41176E-05	c.2235G>A	p.Pro745=	Not present
**chr4:103151254:T>C**	CENPE	1	1,62205E-06	2,87778E-06	c.3361A>G	p.Arg1121Gly	Not present
**chr5:122077510:G>T**	LOX	1	0,006686102	0,0130183	c.476C>A	p.Pro159Gln	Not present
**chr5:132883385:T>C**	AFF4	1	0,00238805	0,0147059	c.3319A>G	p.Thr1107Ala	Not present
**chr5:138191951:A>G**	CDC23	1	0,001124139	0,00236295	c.1287-14T>C	Unknown	Not present
**chr1:237798063:C>T**	RYR2	1	0,002611043	0,00391345	c.13983C>T	p.Tyr4661=	Not present
**chr1:39412111:T>C**	MACF1	1	0,000731337	0,00930031	c.1846T>C	p.Leu616=	Present
**chr16:28832289:C>T**	ATXN2L	1	0,000485243	0,000822603	c.1406C>T	p.Ser469Leu	Not present
**chr4:84695988:G>A**	WDFY3	1	0,000536485	0,00265188	c.8883C>T	p.Phe2961=	Present
**chr3:9434421:G>T**	SETD5	1	2,30549E-05	0,000850897	c.265G>T	p.Val89Leu	Present
**chr8:47831912:T>G**	PRKDC	1	3,59236E-05	0,00121595	c.8167A>C	p.Thr2723Pro	Present
**chr3:129574411:C>T**	PLXND1	1	0,000687276	0,00797151	c.2610G>A	p.Met870Ile	Not present
**chr2:15942774:C>T**	MYCN	1	0,000363572	0,000958773	c.710C>T	p.Pro237Leu	Not present
**chr6:87256819:C>T**	ZNF292	1	0,000659805	0,00113396	c.3190C>T	p.Pro1064Ser	Present
**chr12:6936728:A>ACAGCAGCAGCAGCAGCAGCAGCAGCAGCAGCAG**	ATN1	1	0,00020555	0,000677201	c.1476_1508dup	p.Gln492_Gln502dup	Not present
**chr16:2093545:C>T**	PKD1	1	0,000545589	0,00125188	c.11015G>A	p.Arg3672Gln	Present
**chr7:40094562:T>C**	CDK13	1	0,000354425	0,000946074	c.4121T>C	p.Ile1374Thr	Present
**chr15:63625971:C>T**	HERC1	1	4,82725E-05	0,000196284	c.13275+14G>A	Unknown	Present
**chr1:39410749:A>T**	MACF1	1	0,00327585	0,0396745	c.484A>T	p.Asn162Tyr	Present
**chr1:43605602:G>A**	PTPRF	1	0,000244582	0,00138201	c.3463G>A	p.Glu1155Lys	Not present
**chr1:3414651:C>T**	PRDM16	1	6,93006E-05	0,000121102	c.2691+4C>T	Unknown	Not present
**chr1:6124673:G>C**	CHD5	1	7,56147E-05	0,00123811	c.4395-12C>G	Unknown	Not present
**chr1:8359866:G>A**	RERE	1	0,000137336	0,00143403	c.3516C>T	p.Arg1172=	Present
**chr1:8361203:G>C**	RERE	1	3,90961E-05	0,000956938	c.2304C>G	p.Pro768=	Present
**chr1:12276447:G>A**	VPS13D	1	0,006483198	0,0096432	c.2859G>A	p.Gln953=	Not present
**chr1:26447637:G>A**	DHDDS	1	6,65863E-05	0,00120598	c.519G>A	p.Val173=	Not present
**chr1:30950256:G>A**	PUM1	1	0,00452797	0,00731835	c.2727C>T	p.Gly909=	Not present
**chr10:77637505:A>AGAG**	KCNMA1	1	0,006051724	0,0364343	c.135_137dup	p.Ser60dup	Present
**chr2:224569728:A>INS**	CUL3	1	0	0	Unknown	Unknown	Present

**chr10:78054624:C>T**	RPS24	1	0,000684802	0,00685019	c.484C>T	p.Arg162Trp	Not present
**chr14:18980113:A>G**	POTEM	1	0	0	c.1095A>G	p.Ile365Met	Not present
**chr14:64541356:T>C**	HSPA2	1	0,000419856	0,00536905	c.507T>C	p.Asn169=	Not present
**chr14:71033500:C>T**	PCNX1	1	0,009047663	0,028779	c.3630C>T	p.Tyr1210=	Not present
**chr14:74357075:G>A**	VRTN	1	0,000295833	0,00141911	c.292G>A	p.Ala98Thr	Not present
**chr14:90890663:C>T**	RPS6KA5	1	0,001866747	0,01477	c.1660G>A	p.Asp554Asn	Not present
**chr15:42450074:T>C**	ZNF106	1	0	0	c.2198A>G	p.Gln733Arg	Not present
**chr15:64680795:A>ACAC**	ZNF609	1	6,02386E-05	0,000524453	c.4111_4113dup	p.His1371dup	Not present
**chr15:65463858:G>T**	DPP8	1	0,001798974	0,0181452	c.1874C>A	p.Thr625Asn	Not present
**chr16:70182399:G>A**	CLEC18C	1	0	0	c.586G>A	p.Val196Ile	Not present
**chr16:81699679:T>C**	CMIP	1	6,67579E-05	0,000482625	c.1639-6T>C	Unknown	Present
**chr16:82639403:C>T**	CDH13	1	0,000391445	0,00196271	c.124C>T	p.His42Tyr	Present
**chr17:7417181:G>A**	NLGN2	1	0,003119079	0,0336363	c.1890G>A	p.Pro630=	Present
**chr17:7463501:C>T**	ZBTB4	1	0,001857415	0,0182317	c.1481G>A	p.Ser494Asn	Not present
**chr17:7466484:GGAA>G**	ZBTB4	1	0,002937352	0,0195922	c.315_317del	p.Ser111del	Not present
**chr17:29636533:G>T**	SSH2	1	0,004822982	0,0179211	c.1697C>A	p.Ala566Asp	Not present
**chr17:45238938:A>T**	FMNL1	1	0,004079103	0,0426698	c.970-17A>T	Unknown	Not present
**chr17:45243187:G>A**	FMNL1	1	0,002052224	0,0197691	c.2080G>A	p.Ala694Thr	Not present
**chr17:63817805:A>G**	DDX42	1	0,004663419	0,0136294	c.2224A>G	p.Thr742Ala	Not present
**chr14:52720959:A>G**	PSMC6	1	0,004155497	0,00626798	c.876A>G	p.Pro292=	Not present
**chr13:107210684:A>T**	NALF1	1	0,003006056	0,0350401	c.987T>A	p.Pro329=	Not present
**chr19:3382202:A>G**	NFIC	1	1,41833E-05	0,000226142	c.521A>G	p.Lys174Arg	Not present
**chr13:46130952:C>A**	LCP1	1	0,004537908	0,0467704	c.1627-14G>T	Unknown	Not present
**chr11:62596808:G>A**	MTA2	1	0,002045138	0,0161844	c.711C>T	p.Thr237=	Not present
**chr11:70433166:C>T**	CTTN	1	0,000555961	0,00683119	c.1332C>T	p.Pro444=	Not present
**chr12:6384579:C>T**	LTBR	1	0,002506734	0,0273422	c.97-9C>T	Unknown	Not present
**chr12:6679532:A>G**	ZNF384	1	0,002949996	0,035338	c.-5-7T>C	Unknown	Not present
**chr12:44913793:A>G**	NELL2	1	0,002916912	0,0313132	c.38+6T>C	Unknown	Not present
**chr12:50058782:G>A**	ASIC1	1	1,99987E-05	0,00022057	c.16G>A	p.Glu6Lys	Not present
**chr12:53537486:G>T**	ATF7	1	0,000389438	0,00625626	c.331C>A	p.Pro111Thr	Not present

**chr12:54404227:C>T**	ITGA5	1	0,004174439	0,0468966	c.1483G>A	p.Ala495Thr	Not present
**chr12:57829586:G>A**	CTDSP2	1	1,02443E-05	2,94014E-05	c.75C>T	p.Ser25=	Not present
**chr12:69258118:G>C**	CPSF6	1	0	0	c.778G>C	p.Glu260Gln	Not present
**chr12:70566590:G>A**	PTPRB	1	0,001234539	0,0126597	c.3749C>T	p.Thr1250Ile	Present
**chr12:118150061:G>T**	TAOK3	1	0,005439924	0,0186531	c.2112C>A	p.His704Gln	Not present
**chr12:124913634:A>G**	UBC	1	0,000209572	0,00105693	c.138T>C	p.Ala46=	Not present
**chr12:129700067:C>T**	TMEM132D	1	0,007151842	0,0112457	c.711G>A	p.Gly237=	Not present
**chr13:45968021:C>T**	ZC3H13	1	0,001491997	0,0171758	c.3804G>A	p.Gln1268=	Not present
**chr13:45969002:C>T**	ZC3H13	1	0,000297041	0,00333484	c.3542G>A	p.Arg1181His	Not present
**chr13:45969207:T>C**	ZC3H13	1	0,004213735	0,0486365	c.3337A>G	p.Thr1113Ala	Not present
**chr17:78855792:C>G**	TIMP2	1	0	0	c.538G>C	p.Glu180Gln	Not present
**chr19:4037643:C>T**	PIAS4	1	0,000398211	0,00537391	c.1301C>T	p.Pro434Leu	Not present
**chr10:114220663:G>C**	TDRD1	1	0,003014077	0,0137482	c.2590G>C	p.Val864Leu	Not present
**chr12:111598949:GGCTGCTGCTGCTGCTGCTGCTGCTGCTGTT>G**	ATXN2	1	0,000172417	0,0026705	c.56_85del	p.Gln19_Gln28del	Present
**chr1:7664353:G>A**	CAMTA1	1	0,003402947	0,0378653	c.1806G>A	p.Thr602=	Not present
**chr1:10639215:TGCGGCG>T**	CASZ1	1	0,000151695	0,000523039	c.5001_5006del	p.Ala1669_Ala1670del	Present
**chr1:160354187:A>T**	NCSTN	1	0,003051271	0,0102459	c.1249A>T	p.Asn417Tyr	Not present
**chr2:207767503:G>A**	FZD5	1	0,004638307	0,0230415	c.1237C>T	p.Leu413=	Not present
**chr2:240720970:G>T**	KIF1A	1	0,003897432	0,0251165	c.4812C>A	p.Thr1604=	Present
**chr3:8990718:T>C**	SRGAP3	1	0,001473112	0,0165855	c.2680A>G	p.Ser894Gly	Present
**chr3:9060332:G>A**	SRGAP3	1	0,00392186	0,0447196	c.700C>T	p.Leu234=	Present
**chr3:48659977:G>A**	CELSR3	1	0,002643972	0,0291596	c.2658C>T	p.Asp886=	Not present
**chr3:48660055:A>C**	CELSR3	1	0,001546541	0,0167307	c.2580T>G	p.Ser860Arg	Not present
**chr3:53747413:G>A**	CACNA1D	1	0,002461818	0,00519849	c.3279G>A	p.Ala1093=	Present
**chr3:132484650:C>T**	DNAJC13	1	0,000439195	0,000735121	c.3245C>T	p.Thr1082Ile	Not present
**chr3:181712900:C>T**	SOX2	1	6,02893E-05	0,00120715	c.540C>T	p.Tyr180=	Not present
**chr3:184183586:C>G**	AP2M1	1	0	0	c.1278C>G	p.Gly426=	Present
**chr4:169660441:T>C**	CLCN3	1	0,002255093	0,0216963	c.72T>C	p.His24=	Not present
**chr5:35049386:G>A**	PRLR	1	0,000954649	0,00417076	c.1032C>T	p.Ala344=	Not present
**chr5:83540422:C>T**	VCAN	1	0,00785162	0,0340008	c.7419C>T	p.Ser2473=	Not present
**chr5:169708210:G>A**	DOCK2	1	1,27277E-05	0,000241196	c.1425G>A	p.Glu475=	Not present

**chr7:157181668:C>T**	UBE3C	1	7,82583E-05	0,000484161	c.767C>T	p.Ala256Val	Present
**chr2:108736169:C>G**	RANBP2	1	0,000177186	0,000795545	c.702C>G	p.Asp234Glu	Not present
**chr19:43627796:C>CGATTAGACA**	CADM4	1	0,002913224	0,0323947	c.65-15_65-7dup	Unknown	Not present
**chr2:47809222:T>C**	FBXO11	1	0,000349369	0,00726392	c.2491A>G	p.Ser831Gly	Present
**chr19:48729442:C>T**	RASIP1	1	0	0	c.1328G>A	p.Gly443Asp	Not present
**chr20:33677203:C>T**	E2F1	1	0,000238142	0,00220414	c.968G>A	p.Arg323Lys	Not present
**chr20:43702560:A>G**	MYBL2	1	0,002309569	0,0257841	c.1022A>G	p.Asn341Ser	Not present
**chr20:62882074:CGCTGCTGTTGTG>C**	DIDO1	1	0,004229481	0,0165906	c.3870_3881del	p.Thr1291_Ala1294del	Not present
**chr22:17791046:G>A**	MICAL3	1	6,57661E-06	6,54536E-05	c.5776C>T	p.Arg1926Trp	Not present
**chr22:17818804:G>A**	MICAL3	1	0,00495344	0,0310543	c.3857C>T	p.Pro1286Leu	Not present
**chr22:45577113:G>A**	FBLN1	1	0	0	c.1972+5G>A	Unknown	Not present
**chr22:40273575:G>C**	TNRC6B	1	0	0	c.3116G>C	p.Gly1039Ala	Present
**chr14:77027487:C>DEL**	IRF2BPL	1	0	0,00003	Unknown	Unknown	Present
**chr14:93653990:C>A**	UNC79	1	0	0	c.6580C>A	p.Leu2194Met	Present
**chr11:68416400:C>T**	LRP5	1	9,54271E-06	5,65163E-05	c.2900C>T	p.Pro967Leu	Not present
**chr8:41695184:C>T**	ANK1	1	5,1219E-06	1,46977E-05	c.3108G>A	p.Met1036Ile	Not present
**chr1:155357628:C>T**	ASH1L	1	4,22667E-05	0,000282582	c.6917G>A	p.Arg2306Gln	Present
**chr11:83633320:C>T**	DLG2	1	8,58948E-05	0,000262398	c.1831G>A	p.Ala611Thr	Present
**chr7:41965138:A>C**	GLI3	1	0,000298424	0,0024108	c.3935T>G	p.Met1312Arg	Present
**chr3:179576700:G>A**	ACTL6A	1	0,000537878	0,0092219	c.652G>A	p.Val218Ile	Not present
**chr10:117285036:G>A**	PDZD8	1	0,000537784	0,00457834	c.1697C>T	p.Pro566Leu	Present
**chr11:61863792:C>T**	FADS2	1	0,000882473	0,0038552	c.1157+6C>T	Unknown	Not present
**chr10:91982215:A>G**	BTAF1	1	0,000439257	0,0197781	c.2038A>G	p.Met680Val	Present
**chr6:7230496:C>T**	RREB1	1	0,00167624	0,0101471	c.2397C>T	p.Phe799=	Not present
**chr2:140536716:G>GAA**	LRP1B	1	0,001287785	0,00295469	c.7514-9_7514-8dup	Unknown	Not present
**chr2:159748862:G>A**	MARCHF7	1	2,9519E-05	0,00021693	c.1572G>A	p.Ala524=	Not present
**chr2:173964516:G>A**	SP3	1	0,001977526	0,0230088	c.45C>T	p.Ala15=	Not present
**chr2:177216504:T>A**	HNRNPA3	1	4,11193E-05	0,000483793	c.555T>A	p.Val185=	Not present
**chr2:233195411:C>T**	INPP5D	1	6,79913E-05	0,000243588	c.2609C>T	p.Thr870Met	Not present
**chr2:241249964:G>A**	HDLBP	1	0,003410424	0,0385503	c.1389C>T	p.Asp463=	Present
**chr3:17011743:A>G**	PLCL2	1	4,99288E-05	0,0012054	c.2397A>G	p.Thr799=	Not present
**chr3:33559356:T>C**	CLASP2	1	2,86862E-05	0,000238949	c.2960A>G	p.Asn987Ser	Not present
**chr3:49112414:C>T**	USP19	1	0,000362458	0,00506268	c.2647-12G>A	Unknown	Not present

**chr3:49968120:A>G**	RBM6	1	3,84131E-06	0,000477555	c.695A>G	p.Gln232Arg	Not present
**chr3:56633623:C>T**	TASOR	1	0,00283535	0,00400245	c.3168G>A	p.Glu1056=	Not present
**chr3:154293784:C>T**	DHX36	1	0,000283513	0,00277389	c.1634G>A	p.Arg545Gln	Not present
**chr3:195297290:C>CA**	ACAP2	1	0,005395437	0,0497649	c.1396-10dup	Unknown	Present
**chr3:195302190:ATTAC>A**	ACAP2	1	0,004655811	0,0430071	c.1117-20_1117-17del	Unknown	Present
**chr4:30723650:C>T**	PCDH7	1	0,005129032	0,0139826	c.2228C>T	p.Thr743Ile	Not present
**chr4:88664143:C>T**	HERC3	1	0,000716951	0,00746772	c.1272-10C>T	Unknown	Not present
**chr4:145761282:C>T**	ZNF827	1	3,28465E-05	0,000217155	c.3547G>A	p.Ala1183Thr	Present
**chr4:153604294:A>C**	TMEM131L	1	0,003139211	0,0353982	c.3282A>C	p.Ser1094=	Not present
**chr2:151465131:A>C**	RIF1	1	1,28326E-05	0,00048216	c.5611A>C	p.Asn1871His	Not present
**chr2:97760842:G>A**	TMEM131	1	2,17718E-05	0,0012054	c.4962C>T	p.Asn1654=	Not present
**chr5:72883099:T>C**	TNPO1	1	0,000230674	0,00207792	c.1017T>C	p.Ser339=	Not present
**chr2:64636110:G>A**	SERTAD2	1	0,009330011	0,0265283	c.762C>T	p.Ser254=	Not present
**chr1:27305190:A>G**	WDTC1	1	1,15353E-05	0,000114422	c.1775A>G	p.Gln592Arg	Not present
**chr1:33600966:C>T**	CSMD2	1	0,003065348	0,00502422	c.6755G>A	p.Arg2252Gln	Present
**chr1:36009495:G>A**	AGO3	1	0	0	c.1050G>A	p.Gly350=	Present
**chr1:36301003:G>A**	THRAP3	1	0,00718251	0,0174217	c.2421G>A	p.Thr807=	Not present
**chr1:41581789:G>A**	HIVEP3	1	2,78138E-05	9,40498E-05	c.3009C>T	p.Ala1003=	Present
**chr1:77297617:C>T**	AK5	1	1,59127E-06	3,02371E-05	c.474C>T	p.Phe158=	Not present
**chr1:85582896:C>T**	CCN1	1	0,002465899	0,00604839	c.1000C>T	p.Arg334Trp	Not present
**chr1:109490010:C>T**	ATXN7L2	1	1,92224E-05	0,000208247	c.1214C>T	p.Pro405Leu	Not present
**chr1:153767809:G>A**	INTS3	1	0,003517716	0,0447344	c.2226G>A	p.Thr742=	Not present
**chr1:167389572:A>T**	POU2F1	1	0,000370258	0,00124378	c.814-16A>T	Unknown	Not present
**chr1:171540275:C>G**	PRRC2C	1	0,004661369	0,0310993	c.2809C>G	p.Pro937Ala	Not present
**chr1:171540532:A>G**	PRRC2C	1	0,004664265	0,0310993	c.3066A>G	p.Val1022=	Not present
**chr1:197927733:G>A**	LHX9	1	3,84099E-06	2,86541E-05	c.876G>A	p.Pro292=	Not present
**chr1:200407966:T>C**	ZNF281	1	0,004868712	0,0373674	c.1740A>G	p.Pro580=	Not present
**chr2:23757828:T>C**	ATAD2B	1	0,007751676	0,0192163	c.3668A>G	p.Asn1223Ser	Not present
**chr2:32529828:T>A**	BIRC6	1	0,000118663	0,0149363	c.12094+4T>A	Unknown	Present
**chr2:40428770:G>T**	SLC8A1	1	1,59118E-06	0,000241313	c.1511C>A	p.Ala504Asp	Not present

**chr5:43297039:A>G**	HMGCS1	1	6,36515E-06	0,000241196	c.702T>C	p.Ser234=	Not present
**chr5:119177989:G>A**	DMXL1	1	0,007285076	0,0112067	c.6887-7G>A	Unknown	Not present
**chr10:73792540:T>C**	ZSWIM8	1	0,000674859	0,0072324	c.2001T>C	p.Tyr667=	Not present
**chr7:98193678:G>A**	LMTK2	1	0,004200271	0,0488866	c.3213G>A	p.Pro1071=	Not present
**chr7:149455581:C>T**	ZNF777	1	5,17613E-06	7,19528E-05	c.442G>A	p.Glu148Lys	Not present
**chr8:73673178:G>A**	STAU2	1	1,5512E-05	0,00243784	c.339C>T	p.Tyr113=	Not present
**chr8:130180885:A>C**	ASAP1	1	0,003931081	0,0118765	c.531-5T>G	Unknown	Not present
**chr9:67899325:T>C**	ANKRD20A1	1	0	0	c.1495T>C	p.Trp499Arg	Not present
**chr9:113988351:C>G**	ZNF618	1	0,004296198	0,0444562	c.108C>G	p.Thr36=	Not present
**chr9:116220050:G>C**	PAPPA	1	0,003123327	0,0341008	c.2032G>C	p.Ala678Pro	Not present
**chr9:123179143:C>T**	STRBP	1	0	0	c.88G>A	p.Ala30Thr	Not present
**chr9:125672553:T>C**	MAPKAP1	1	0,000308543	0,0036162	c.22A>G	p.Thr8Ala	Not present
**chr9:131464805:G>A**	PRRC2B	1	0,000150276	0,001213	c.1447G>A	p.Asp483Asn	Not present
**chr9:131475019:C>CAGG**	PRRC2B	1	0,000130654	0,00144858	c.2893_2895dup	p.Glu965dup	Not present
**chr9:136050060:G>C**	NACC2	1	0,000143517	0,00133215	c.462C>G	p.Ala154=	Not present
**chr10:1010417:T>C**	GTPBP4	1	6,57022E-06	2,41324E-05	c.1244-3T>C	Unknown	Not present
**chr10:1015825:C>G**	GTPBP4	1	0,007224649	0,0491155	c.1681C>G	p.Pro561Ala	Not present
**chr10:5937259:C>G**	FBH1	1	0,005440113	0,036515	c.3111C>G	p.Ala1037=	Not present
**chr10:7220434:C>T**	SFMBT2	1	1,66472E-05	0,00019216	c.1307G>A	p.Arg436Gln	Not present
**chr10:30026747:G>A**	JCAD	1	0,001838489	0,0197686	c.3401C>T	p.Pro1134Leu	Not present
**chr10:33213449:G>A**	NRP1	1	0,000290742	0,00230814	c.1551C>T	p.Ile517=	Not present
**chr7:139777618:G>A**	HIPK2	1	0,008362879	0,0156319	c.6C>T	p.Ala2=	Not present
**chr7:98190776:G>A**	LMTK2	1	0,000349535	0,00313404	c.1047G>A	p.Pro349=	Not present
**chr5:157814291:C>T**	CLINT1	1	7,63683E-05	0,00126199	c.246G>A	p.Ser82=	Not present
**chr7:28804494:C>T**	CREB5	1	2,56151E-05	0,00024108	c.998C>T	p.Pro333Leu	Not present
**chr6:12122277:G>A**	HIVEP1	1	0,002687112	0,0288798	c.2482G>A	p.Val828Ile	Not present
**chr6:12122959:C>T**	HIVEP1	1	0,002612571	0,0284419	c.3164C>T	p.Ser1055Phe	Not present
**chr6:17794569:T>C**	KIF13A	1	0,000104033	0,00141777	c.3075+3A>G	Unknown	Not present
**chr6:30554818:C>T**	GNL1	1	0,007710632	0,0291707	c.474G>A	p.Lys158=	Not present
**chr6:30602901:AGGACCCCCT>A**	PPP1R10	1	0,007699476	0,0281725	c.1893_1901del	p.Gly635_Pro637del	Not present

**chr6:30609070:C>T**	PPP1R10	1	0,0089858	0,0294118	c.194+7G>A	Unknown	Not present
**chr6:31631385:C>A**	PRRC2A	1	0,00256947	0,0134979	c.2712C>A	p.Arg904=	Not present
**chr6:39048899:G>A**	GLP1R	1	0,004104257	0,021902	c.59G>A	p.Arg20Lys	Not present
**chr6:42236244:G>A**	TRERF1	1	0,001028337	0,00675871	c.3027C>T	p.Pro1009=	Not present
**chr6:83135669:G>T**	DOP1A	1	0,000219111	0,00891996	c.2921G>T	p.Ser974Ile	Not present
**chr6:89862362:T>G**	CASP8AP2	1	0,001893287	0,0184516	c.653T>G	p.Val218Gly	Not present
**chr6:106107320:G>A**	PRDM1	1	0,002896443	0,0269018	c.2312G>A	p.Gly771Asp	Not present
**chr6:154824221:C>G**	SCAF8	1	0,000349399	0,00236967	c.1927-13C>G	Unknown	Not present
**chr6:158481108:C>T**	TULP4	1	0,000423695	0,00418434	c.1305C>T	p.Ala435=	Not present
**chr7:6147840:A>G**	USP42	1	0,000939238	0,0107959	c.1334A>G	p.Lys445Arg	Not present
**chr7:21429399:A>G**	SP4	1	0,002984222	0,0348141	c.234A>G	p.Gln78=	Not present
**chr7:26211755:T>DEL**	CBX3	1	0	0,009532	Unknown	Unknown	Not present
**chr5:171910812:C>CA**	FBXW11	1	0,003281732	0,00989209	c.211-16dup	Unknown	Not present
**chr6:7727292:C>G**	BMP6	1	0,004126011	0,0302658	c.337C>G	p.Gln113Glu	Not present
**chr1:19185180:G>A**	UBR4	1	0,001545344	0,0121763	c.1857C>T	p.Ser619=	Not present
**chr6:43186943:T>C**	CUL9	1	1,9231E-05	9,15751E-05	c.1252-17T>C	Unknown	Not present
**chr6:151351337:A>G**	AKAP12	1	6,40195E-06	4,76554E-05	c.2946A>G	p.Ala982=	Not present
**chr7:104736660:CT>C**	LHFPL3	1	0,000383612	0,00353137	c.446-14del	Unknown	Not present
**chr7:132507563:G>A**	PLXNA4	1	0,000431641	0,0011889	c.1131C>T	p.Gly377=	Present
**chr7:154955573:G>C**	PAXIP1	1	0	0	c.2608C>G	p.Leu870Val	Not present
**chr8:35748605:C>T**	UNC5D	1	2,30507E-05	0,00024108	c.1845C>T	p.Pro615=	Present
**chr8:61499445:C>T**	CLVS1	1	3,18447E-06	3,02572E-05	c.978-10C>T	Unknown	Not present
**chr8:112234404:C>G**	CSMD3	1	9,09911E-05	0,000161698	c.10701G>C	p.Val3567=	Present
**chr8:112234405:A>G**	CSMD3	1	9,09965E-05	0,000161774	c.10700T>C	p.Val3567Ala	Present
**chr8:138148521:A>G**	FAM135B	1	0,006169535	0,0260492	c.3447T>C	p.Asp1149=	Not present
**chr9:8507308:C>T**	PTPRD	1	4,77404E-06	4,57394E-05	c.1670G>A	p.Gly557Glu	Present
**chr9:86309535:C>T**	TUT7	1	2,94803E-05	0,000120779	c.3510G>A	p.Ser1170=	Not present
**chr9:86346305:T>C**	TUT7	1	0,001884835	0,00696667	c.696A>G	p.Leu232=	Not present
**chr9:134045323:G>A**	BRD3	1	0,000522767	0,00819672	c.1185C>T	p.His395=	Not present
**chr10:5763019:G>C**	TASOR2	1	0,000271097	0,0011534	c.7290-10G>C	Unknown	Not present
**chr10:67992660:T>C**	HERC4	1	0,000828776	0,00969496	c.1092A>G	p.Val364=	Not present

**chr10:68572579:C>T**	TET1	1	2,68913E-05	6,04303E-05	c.241C>T	p.Arg81Cys	Not present
**chr10:87361016:C>T**	NUTM2D	1	0,00028651	0,0030303	c.702C>T	p.His234=	Not present
**chr6:136272009:C>T**	BCLAF1	1	4,81931E-06	8,63598E-06	c.2029G>A	p.Glu677Lys	Not present
**chr6:31631535:ACCT>A**	PRRC2A	1	1,05512E-05	0,000248262	c.2865_2867del	p.Pro958del	Not present
**chr10:103379720:G>A**	TAF5	1	0,005731333	0,0130597	c.1226G>A	p.Ser409Asn	Not present
**chr6:17629053:T>C**	NUP153	1	0,002775495	0,00498645	c.3146A>G	p.Asn1049Ser	Not present
**chr1:232514418:T>C**	SIPA1L2	1	0,000876946	0,00148442	c.922A>G	p.Thr308Ala	Not present
**chr2:140234871:C>T**	LRP1B	1	0,000540662	0,000861393	c.13574G>A	p.Gly4525Glu	Not present
**chr2:140700613:C>A**	LRP1B	1	0,00357314	0,0138869	c.6436G>T	p.Val2146Phe	Not present
**chr2:209692919:T>C**	MAP2	1	3,81728E-05	0,00024108	c.749T>C	p.Ile250Thr	Not present
**chr2:217880994:C>G**	TNS1	1	0,004397242	0,0158593	c.1333G>C	p.Gly445Arg	Not present
**chr3:20140317:C>T**	KAT2B	1	0,003361506	0,0178571	c.1957C>T	p.Arg653Trp	Present
**chr3:26710245:C>T**	LRRC3B	1	0,006985616	0,0175201	c.573C>T	p.Asp191=	Not present
**chr3:48164392:A>G**	CDC25A	1	0,002582702	0,00463992	c.1237T>C	p.Leu413=	Not present
**chr3:48967119:C>T**	ARIH2	1	0,003195416	0,0342341	c.388-6C>T	Unknown	Not present
**chr3:49108462:G>T**	USP19	1	0,003208481	0,0357263	c.4105C>A	p.Pro1369Thr	Not present
**chr3:50176800:C>T**	SEMA3F	1	0,003442321	0,00675024	c.582C>T	p.Leu194=	Not present
**chr3:183988580:G>A**	ABCC5	1	0,001299424	0,00258649	c.435C>T	p.Asn145=	Not present
**chr4:18021686:A>AGCG**	LCORL	1	0,001985111	0,00803043	c.63_65dup	p.Ala22dup	Not present
**chr4:144734742:C>T**	HHIP	1	0,000309859	0,0044784	c.1762C>T	p.Pro588Ser	Not present
**chr5:119133866:G>A**	DMXL1	1	0,001752944	0,010964	c.1942G>A	p.Val648Ile	Not present
**chr5:173944977:A>C**	CPEB4	1	0,000802337	0,00507492	c.1293A>C	p.Ser431=	Present
**chr5:181234688:C>T**	TRIM41	1	6,40203E-06	1,46977E-05	c.1806C>T	p.His602=	Not present
**chr10:87361023:G>A**	NUTM2D	1	0,000308995	0,00225904	c.709G>A	p.Gly237Ser	Not present
**chr11:6403776:C>T**	APBB1	1	2,52378E-05	0,000265431	c.768G>A	p.Pro256=	Present
**chr1:113590573:T>C**	MAGI3	1	0,00615897	0,0127119	c.853T>C	p.Tyr285His	Not present
**chr16:85634077:C>T**	GSE1	1	0,007248598	0,0440668	c.171C>T	p.Ser57=	Not present
**chr17:3883929:G>A**	CAMKK1	1	0,008922554	0,0132576	c.417C>T	p.Tyr139=	Not present
**chr17:38779484:C>T**	PIP4K2B	1	0,003216208	0,0118156	c.553G>A	p.Gly185Ser	Not present
**chr17:42724401:C>T**	EZH1	1	1,53717E-05	6,04412E-05	c.270G>A	p.Pro90=	Not present

**chr17:44398302:T>C**	GPATCH8	1	0,005122229	0,0180348	c.3775A>G	p.Ser1259Gly	Not present
**chr17:59914627:T>A**	RPS6KB1	1	0,00759493	0,0115168	c.313-8T>A	Unknown	Not present
**chr18:25062752:CA>C**	ZNF521	1	0,008077447	0,0174026	c.3907-12del	Unknown	Not present
**chr19:5243911:C>T**	PTPRS	1	2,18593E-05	0,000250344	c.1560G>A	p.Thr520=	Present
**chr19:5262968:G>C**	PTPRS	1	0,006082071	0,01509	c.573C>G	p.Thr191=	Present
**chr19:11777597:G>T**	ZNF441	1	0,001293796	0,00236695	c.4-14G>T	Unknown	Not present
**chr19:18574964:C>A**	UBA52	1	0	0	c.285C>A	p.Ile95=	Not present
**chr19:46347098:T>C**	PPP5C	1	0,001979976	0,0443804	c.2T>C	p.Met1?	Present
**chr19:46678442:G>A**	PRKD2	1	0,000221527	0,00120446	c.2292C>T	p.Ala764=	Present
**chr19:49802561:C>T**	AP2A1	1	0,001689111	0,0276657	c.2142C>T	p.Ser714=	Not present
**chr20:63746978:C>T**	ZBTB46	1	2,98697E-05	0,000193424	c.1722G>A	p.Pro574=	Not present
**chr20:63962387:C>T**	ZNF512B	1	0,00029904	0,000504726	c.2164-13G>A	Unknown	Not present
**chr22:17790868:T>C**	MICAL3	1	9,54745E-06	3,02389E-05	c.5873A>G	p.Asn1958Ser	Not present
**chr22:20564628:C>CCAG**	MED15	1	0,005179013	0,00954198	c.654_656dup	p.Gln218dup	Not present
**chr16:87433197:A>G**	ZCCHC14	1	0,000929704	0,00235294	c.699T>C	p.Ser233=	Not present
**chr16:69367182:G>T**	TERF2	1	0,001572197	0,0192374	c.965C>A	p.Pro322Gln	Present
**chr11:32932818:G>A**	QSER1	1	0,002275719	0,00360789	c.1560G>A	p.Thr520=	Not present
**chr16:23069629:A>G**	USP31	1	6,66081E-06	7,30745E-05	c.2489-13T>C	Unknown	Not present
**chr11:63997467:C>T**	OTUB1	1	4,77158E-06	5,65163E-05	c.737C>T	p.Pro246Leu	Not present
**chr11:64315801:C>T**	ESRRA	1	0,009288724	0,0161383	c.1107C>T	p.Pro369=	Not present
**chr12:14424331:T>G**	ATF7IP	1	0,000590195	0,00444139	c.416T>G	p.Leu139Arg	Not present
**chr12:45948508:T>C**	SCAF11	1	0,008260005	0,0474802	c.327A>G	p.Thr109=	Not present
**chr12:50073893:C>A**	ASIC1	1	0	0	c.309C>A	p.Phe103Leu	Not present
**chr12:70559409:C>T**	PTPRB	1	0,006145646	0,0245994	c.4648G>A	p.Val1550Ile	Present
**chr12:122997507:G>A**	PITPNM2	1	0,003189831	0,0168634	c.1290C>T	p.His430=	Not present
**chr13:20827145:C>T**	XPO4	1	0,000373981	0,000647021	c.762G>A	p.Ser254=	Not present
**chr13:20989313:G>GA**	LATS2	1	0,000372999	0,000720376	c.476-10dup	Unknown	Not present
**chr13:25340029:T>C**	NUP58	1	1,59161E-05	4,58106E-05	c.1695T>C	p.Gly565=	Not present
**chr13:97985555:A>G**	IPO5	1	0,000556948	0,00471833	c.306A>G	p.Gln102=	Not present
**chr13:98883901:TA>T**	DOCK9	1	0,000186062	0,000684731	c.2383-3del	Unknown	Not present

**chr14:90695594:A>T**	TTC7B	1	0,000436433	0,0131514	c.699-16T>A	Unknown	Not present
**chr15:34884640:T>C**	AQR	1	0,001590513	0,0022739	c.2912A>G	p.His971Arg	Not present
**chr15:34910223:G>C**	AQR	1	0,003132922	0,00485052	c.1575C>G	p.Pro525=	Not present
**chr15:72209769:G>A**	PKM	1	5,25067E-05	0,000117657	c.469C>T	p.Leu157=	Not present
**chr15:85580395:A>G**	AKAP13	1	4,77119E-06	3,02133E-05	c.2327A>G	p.Asp776Gly	Not present
**chr1:167415742:T>C**	POU2F1	1	0,001215152	0,00795564	c.2233T>C	p.Ser745Pro	Not present
**chr1:111183476:G>A**	CEPT1	1	0,002099909	0,0139894	c.1020G>A	p.Thr340=	Not present
**chr6:15517262:C>T**	JARID2	1	0,000722194	0,00842284	c.3552C>T	p.Tyr1184=	Present
**chr9:138057795:C>T**	CACNA1B	1	0,003241499	0,0375127	c.4032C>T	p.Tyr1344=	Present
**chr10:103678148:G>A**	SH3PXD2A	1	0,000820028	0,00616698	c.48C>T	p.Asn16=	Not present
**chr11:1251567:G>T**	MUC5B	1	0,007020043	0,0187102	c.14687G>T	p.Ser4896Ile	Not present
**chr11:1450685:C>T**	BRSK2	1	1,93237E-05	6,53937E-05	c.1386C>T	p.Pro462=	Present
**chr11:46397642:C>A**	AMBRA1	1	4,87536E-05	0,000483325	c.3705G>T	p.Gln1235His	Not present
**chr11:68411562:C>T**	LRP5	1	0,000320236	0,00219499	c.2445C>T	p.Asp815=	Not present
**chr11:70798550:C>T**	SHANK2	1	0,002915172	0,0123848	c.1670G>A	p.Ser557Asn	Present
**chr11:102110929:G>A**	YAP1	1	4,61045E-05	0,000480307	c.81G>A	p.Gln27=	Not present
**chr11:113410754:C>T**	DRD2	1	0,00043279	0,00552802	c.1305G>A	p.Lys435=	Present
**chr11:115240380:G>A**	CADM1	1	3,45955E-05	0,00024108	c.165C>T	p.Ile55=	Present
**chr12:106720770:G>A**	RFX4	1	0,003637793	0,0409872	c.1245G>A	p.Lys415=	Present
**chr12:122333029:G>C**	CLIP1	1	1,9218E-05	0,000131113	c.2825C>G	p.Ser942Cys	Not present
**chr12:132044823:G>A**	EP400	1	0,004828747	0,0193586	c.6654G>A	p.Pro2218=	Present
**chr12:132066893:C>T**	EP400	1	0,000147378	0,000654108	c.8673C>T	p.Ser2891=	Present
**chr14:50138716:C>T**	2 SOS	1	0,000308608	0,00157749	c.2854G>A	p.Asp952Asn	Present
**chr14:64800825:A>G**	SPTB	1	0,000440385	0,00514299	c.807T>C	p.Tyr269=	Not present
**chr15:50481593:G>A**	USP8	1	3,02208E-05	0,000241196	c.1331G>A	p.Arg444His	Not present
**chr15:51576183:T>TAAAAAAAAAAA**	DMXL2	1	0,005034217	0,00751526	c.88-13_88-3dup	Unknown	Present
**chr10:77636494:C>G**	KCNMA1	1	0,006895296	0,039777	c.400G>C	p.Glu134Gln	Present
**chr9:137754162:G>T**	EHMT1	1	0,002066682	0,0168438	c.1249-9G>T	Unknown	Present
**chr15:101178100:T>C**	CHSY1	1	1,43138E-05	4,29812E-05	c.1697A>G	p.Lys566Arg	Not present

**chr9:128258143:C>T**	GOLGA2	1	0,001782683	0,00902589	c.2345G>A	p.Arg782His	Not present
**chr6:24842946:T>G**	RIPOR2	1	0,001473857	0,0152651	c.1773A>C	p.Leu591Phe	Not present
**chr6:24843003:A>G**	RIPOR2	1	0,001423422	0,0151135	c.1716T>C	p.Ser572=	Not present
**chr6:24843106:G>A**	RIPOR2	1	0,001439439	0,0149768	c.1613C>T	p.Ser538Leu	Not present
**chr6:24843267:T>C**	RIPOR2	1	0,001217813	0,0148655	c.1452A>G	p.Pro484=	Not present
**chr6:24843322:C>T**	RIPOR2	1	0,00121795	0,0148638	c.1397G>A	p.Arg466Lys	Not present
**chr6:28921964:G>A**	TRIM27	1	0,003682202	0,0260366	c.444C>T	p.Asp148=	Not present
**chr6:69324862:G>T**	ADGRB3	1	0,001097441	0,0171663	c.2815-10G>T	Unknown	Not present
**chr6:75123971:C>T**	COL12A1	1	0,003287652	0,0366858	c.6848G>A	p.Gly2283Glu	Present
**chr6:157200956:G>A**	ARID1B	1	0,000295801	0,00271309	c.4731G>A	p.Ser1577=	Present
**chr7:82951497:T>C**	PCLO	1	0,002938303	0,0305037	c.9098-7A>G	Unknown	Present
**chr7:83135608:C>T**	PCLO	1	0,001887515	0,019254	c.1942G>A	p.Gly648Arg	Present
**chr7:98917658:G>A**	TRRAP	1	0,000499359	0,00514993	c.2601G>A	p.Pro867=	Present
**chr8:32763857:A>G**	NRG1	1	1,28075E-05	0,000241196	c.1378A>G	p.Thr460Ala	Not present
**chr8:32763934:G>A**	NRG1	1	1,28087E-05	0,000241196	c.1455G>A	p.Arg485=	Not present
**chr8:47913952:G>A**	PRKDC	1	7,70258E-05	0,000333434	c.2730C>T	p.Phe910=	Present
**chr9:2170426:G>A**	SMARCA2	1	0,000747771	0,00538805	c.4207G>A	p.Val1403Met	Present
**chr9:3275625:C>G**	RFX3	1	0,005085712	0,0294118	c.974-13G>C	Unknown	Present
**chr15:84798771:G>A**	ZNF592	1	1,61273E-06	2,85879E-06	c.2920G>A	p.Ala974Thr	Not present
**chr16:2089874:G>A**	PKD1	1	0,004412302	0,00783455	c.12765C>T	p.Pro4255=	Present
**chr1:108699434:G>A**	PRPF38B	1	0,007464693	0,0116069	c.1055G>A	p.Arg352Lys	Not present
**chr22:41527419:C>T**	ACO2	1	6,95554E-05	0,000207211	c.2085C>T	p.His695=	Not present
**chr6:36210481:C>T**	BRPF3	1	4,15194E-05	7,15164E-05	c.2132C>T	p.Pro711Leu	Not present
**chr1:52488919:T>C**	TUT4	1	2,44067E-05	7,34927E-05	c.1505A>G	p.Tyr502Cys	Not present
**chr17:29576384:G>A**	GIT1	1	8,84293E-05	0,00143403	c.1447C>T	p.Leu483Phe	Not present
**chr10:5742222:G>A**	TASOR2	1	0,000267607	0,00115274	c.2453G>A	p.Ser818Asn	Not present
**chr10:5746620:G>C**	TASOR2	1	0,000268867	0,0011534	c.3199G>C	p.Glu1067Gln	Not present
**chr10:5748748:T>C**	TASOR2	1	0,000270146	0,00115407	c.5327T>C	p.Val1776Ala	Not present
**chr1:6680967:T>A**	DNAJC11	1	0,001436546	0,0230674	c.143A>T	p.Asp48Val	Not present

**chr1:16563946:C>T**	NBPF1	1	0,000142491	0,000142491	c.3261G>A	p.Gln1087=	Not present
**chr1:16563989:G>C**	NBPF1	1	0,001572327	0	c.3218C>G	p.Thr1073Arg	Not present
**chr1:16564107:T>G**	NBPF1	1	0	0	c.3100A>C	p.Met1034Leu	Not present
**chr1:16564144:C>T**	NBPF1	1	0,000979624	0,00113475	c.3063G>A	p.Leu1021=	Not present
**chr1:19173594:A>G**	UBR4	1	0,005487485	0,0087905	c.3010T>C	p.Leu1004=	Not present
**chr1:27306365:G>A**	WDTC1	1	1,15432E-05	0,000473037	c.2016G>A	p.Val672=	Not present
**chr1:31728043:G>C**	ADGRB2	1	0,001051125	0,0101543	c.4554C>G	p.Ala1518=	Not present
**chr1:34868707:C>T**	DLGAP3	1	2,05641E-05	9,14537E-05	c.2383G>A	p.Gly795Ser	Present
**chr1:40161511:C>T**	RLF	1	0,001965768	0,0028463	c.112C>T	p.Arg38Cys	Not present
**chr1:55178049:C>T**	USP24	1	0,001228946	0,0115823	c.408G>A	p.Leu136=	Present
**chr22:50466657:G>A**	SBF1	1	0,002229046	0,0118418	c.603C>T	p.Ala201=	Present
**chr22:29694806:G>A**	NF2	1	0,000713615	0,00505269	c.1629G>A	p.Arg543=	Not present
**chr16:30724027:C>G**	SRCAP	1	0,008030683	0,0274831	c.4603C>G	p.Pro1535Ala	Present
**chr22:27798945:TTGC>T**	MN1	1	0,005243081	0,0394758	c.1596_1598del	p.Gln550del	Not present
**chr16:69692351:G>A**	NFAT5	1	0,009074522	0,0198847	c.2526G>A	p.Glu842=	Not present
**chr16:72787694:A>DEL**	ZFHX3, ZFHX3-AS1	1	0	0	Unknown	Unknown	Not present
**chr16:72787694:AGCCGCCGCCGCCGCCGCCGCCGCCACCGCCGCCGCCGCCACTGCCACC>A**	ZFHX3	1	0,000138715	0,000806614	c.10531_10581del	p.Gly3511_Gly3527del	Present
**chr16:81895790:C>T**	PLCG2	1	7,30028E-05	0,000243574	c.1073-17C>T	Unknown	Not present
**chr17:46094686:T>C**	KANSL1	1	5,12101E-06	0,000207125	c.1305A>G	p.Glu435=	Present
**chr17:61479873:C>T**	TBX4	1	0,001898794	0,00319043	c.703-8C>T	Unknown	Not present
**chr18:55234615:C>G**	TCF4	1	0,002216514	0,0227535	c.1419G>C	p.Pro473=	Present
**chr18:74632152:A>G**	ZNF407	1	0,000966803	0,010341	c.1133A>G	p.Gln378Arg	Not present
**chr18:75064204:C>T**	ZNF407	1	0,000243725	0,00216327	c.6483C>T	p.Gly2161=	Not present
**chr18:75287069:C>T**	TSHZ1	1	0	0	c.1662C>T	p.Ser554=	Present
**chr19:4409009:C>G**	CHAF1A	1	0,002034384	0,0227221	c.210C>G	p.Ala70=	Not present
**chr19:8584904:C>T**	ADAMTS10	1	0,001933361	0,0210319	c.3193G>A	p.Gly1065Ser	Not present
**chr20:9435543:G>T**	PLCB4	1	0,002002718	0,0230318	c.2525-17G>T	Unknown	Not present
**chr20:45945820:C>T**	PCIF1	1	0,003262382	0,0383004	c.1278C>T	p.Asn426=	Not present
**chr20:63488394:C>T**	EEF1A2	1	0,003935422	0,00603476	c.1296G>A	p.Thr432=	Present
**chr21:39197093:A>G**	BRWD1	1	0,000203576	0,00180873	c.5976T>C	p.Tyr1992=	Not present

**chr22:21769312:G>A**	MAPK1	1	0,001248933	0,0139912	c.975C>T	p.Ala325=	Not present
**chr1:20942220:T>C**	EIF4G3	1	8,45086E-05	0,000543872	c.934A>G	p.Ile312Val	Not present
**chr1:19176687:C>T**	UBR4	1	0,002390586	0,012512	c.2678G>A	p.Ser893Asn	Not present
**chr10:103735733:C>T**	SH3PXD2A	1	0,000827484	0,00328822	c.305G>A	p.Arg102Gln	Not present
**chr10:103678112:C>T**	SH3PXD2A	1	0,000140333	0,00111082	c.84G>A	p.Ala28=	Not present
**chr10:117285289:C>T**	PDZD8	1	0,00450154	0,00843358	c.1444G>A	p.Ala482Thr	Present
**chr11:34082808:A>AT**	CAPRIN1	1	3,8548E-05	0,000328515	c.827-11dup	Unknown	Present
**chr11:62628776:C>T**	GANAB	1	0,000463724	0,00266473	c.2173G>A	p.Val725Ile	Not present
**chr11:64805088:C>T**	MEN1	1	0,00127262	0,00434783	c.1296G>A	p.Leu432=	Not present
**chr11:78658157:G>A**	TENM4	1	0,000128058	0,000473934	c.8211C>T	p.Tyr2737=	Not present
**chr12:323210:C>CAAAAAAAAAA**	KDM5A	1	0,004949955	0,00634016	c.2151-14_2151-5dup	Unknown	Present
**chr12:64455879:C>T**	TBK1	1	0,000689485	0,00895307	c.9C>T	p.Ser3=	Not present
**chr12:101685607:C>T**	MYBPC1	1	0,003793819	0,0414714	c.3497C>T	p.Ser1166Phe	Not present
**chr12:112212598:C>T**	HECTD4	1	0,003875919	0,0449381	c.7518G>A	p.Pro2506=	Present
**chr12:112244013:G>GA**	HECTD4	1	0,000488743	0,0038059	c.4514-5dup	Unknown	Present
**chr12:131982328:G>A**	EP400	1	0,002573663	0,0300929	c.1779G>A	p.Pro593=	Present
**chr12:132038100:AGAATACATT>A**	EP400	1	0,002460727	0,029324	c.6207+9_6207+17del	Unknown	Present
**chr12:132055000:T>G**	EP400	1	0,004220868	0,0479277	c.7755T>G	p.Thr2585=	Present
**chr14:35651890:A>T**	RALGAPA1	1	0,002186124	0,0181087	c.5608-17T>A	Unknown	Not present
**chr15:68289730:C>G**	FEM1B	1	0,003360688	0,0394015	c.372C>G	p.Pro124=	Not present
**chr15:68290021:G>A**	FEM1B	1	0,003468239	0,0406582	c.663G>A	p.Thr221=	Not present
**chr17:4195452:T>C**	ANKFY1	1	0,003823264	0,00573912	c.1123A>G	p.Ile375Val	Not present
**chr10:110577477:A>T**	SMC3	1	1,53766E-05	0,000120992	c.255A>T	p.Ser85=	Present
**chr10:103613013:G>T**	SH3PXD2A	1	0,000166428	0,00124102	c.1098C>A	p.Gly366=	Not present
**chr17:61483092:C>G**	TBX4	1	0,00092447	0,00650916	c.1217C>G	p.Ser406Cys	Not present
**chr10:60073656:A>G**	ANK3	1	0,005108028	0,0106126	c.7225T>C	p.Ser2409Pro	Present
**chr6:32024331:C>T**	C4B	1	0	0	c.1341+7C>T	Unknown	Present
**chr6:35241664:A>G**	SCUBE3	1	0,000637577	0,00704767	c.1312+5A>G	Unknown	Not present
**chr6:39902007:G>T**	DAAM2	1	0,003803167	0,0384282	c.3177G>T	p.Arg1059=	Not present
**chr6:56594038:A>C**	DST	1	0,004074976	0,0431415	c.12351T>G	p.Thr4117=	Present
**chr6:56608121:T>C**	DST	1	0,004108441	0,0436185	c.6507A>G	p.Gln2169=	Present

**chr6:75133360:G>A**	COL12A1	1	0,000606146	0,00559891	c.5727C>T	p.Tyr1909=	Present
**chr6:111344045:T>TA**	REV3L	1	0,003127173	0,0241063	c.7420-3dup	Unknown	Not present
**chr6:137877060:T>G**	TNFAIP3	1	6,76056E-06	3,20225E-05	c.806-16T>G	Unknown	Not present
**chr6:157207045:T>C**	ARID1B	1	0	0	c.6273T>C	p.His2091=	Present
**chr6:160024563:C>A**	IGF2R	1	0,000233567	0,00159763	c.514-9C>A	Unknown	Not present
**chr6:160027230:G>A**	IGF2R	1	0,000839833	0,00921216	c.692G>A	p.Gly231Asp	Not present
**chr6:160061560:C>T**	IGF2R	1	0,004327752	0,0495617	c.3320C>T	p.Thr1107Met	Not present
**chr6:160073896:A>G**	IGF2R	1	0,001560576	0,0128292	c.5087A>G	p.Gln1696Arg	Not present
**chr6:169232998:G>C**	THBS2	1	0,001166884	0,0132041	c.1671C>G	p.Pro557=	Not present
**chr9:128632799:C>T**	SPTAN1	1	0,000367495	0,00189215	c.7161-9C>T	Unknown	Not present
**chr9:129120497:A>AT**	PTPA	1	0,007738847	0,0114862	c.32-7dup	Unknown	Not present
**chr9:134700063:G>T**	COL5A1	1	5,77309E-05	0,000718579	c.432G>T	p.Thr144=	Not present
**chr17:7903922:C>T**	CHD3	1	0,008933875	0,012879	c.3825C>T	p.Asp1275=	Present
**chr17:75225787:C>G**	NUP85	1	4,80923E-06	4,58716E-05	c.945C>G	p.Ser315=	Not present
**chr5:153492252:G>T**	GRIA1	1	1,27257E-05	0,0002457	c.54G>T	p.Glu18Asp	Present
**chr1:85582843:C>G**	CCN1	1	0,005152079	0,00724744	c.947C>G	p.Ser316Cys	Not present
**chr1:156938433:C>T**	ARHGEF11	1	0,000782735	0,00262204	c.4177G>A	p.Gly1393Arg	Not present
**chr1:200849852:C>G**	CAMSAP2	1	0,008748095	0,0207329	c.3083C>G	p.Pro1028Arg	Not present
**chr1:201783702:T>C**	NAV1	1	7,68305E-06	9,14495E-05	c.3515T>C	p.Met1172Thr	Not present
**chr1:225519206:G>A**	ENAH	1	0,000407318	0,00384833	c.794C>T	p.Ser265Leu	Not present
**chr1:235175303:G>A**	ARID4B	1	0,002152556	0,00374856	c.3545C>T	p.Thr1182Ile	Not present
**chr2:53898342:A>G**	PSME4	1	0,002710999	0,0314503	c.3435T>C	p.Asn1145=	Not present
**chr2:71426555:G>A**	ZNF638	1	0,00083627	0,0102631	c.4686G>A	p.Lys1562=	Not present
**chr2:95941204:TAA>T**	ANKRD36C	1	0,000414331	0,000776888	c.1492-7_1492-6del	Unknown	Not present
**chr2:128016539:C>T**	SAP130	1	0,000701436	0,00439109	c.357G>A	p.Pro119=	Not present
**chr2:140994107:T>C**	LRP1B	1	0,005401258	0,0371061	c.2532A>G	p.Ile844Met	Not present
**chr2:186762529:C>T**	FAM171B	1	0,000439496	0,00229538	c.2187C>T	p.Ser729=	Not present
**chr2:203439476:G>T**	RAPH1	1	0,000589108	0,00729061	c.3714C>A	p.Pro1238=	Not present
**chr2:238182057:T>C**	ILKAP	1	0,000293669	0,00242014	c.836+8A>G	Unknown	Not present
**chr3:43343455:G>A**	SNRK	1	0,004307958	0,0499468	c.1056G>A	p.Pro352=	Not present

**chr3:127821802:G>A**	MGLL	1	0,001163851	0,0220813	c.47C>T	p.Ser16Phe	Not present
**chr3:130728699:A>AAAG**	PIK3R4	1	0,000879716	0,00154056	c.1586-16_1586-15insCTT	Unknown	Not present
**chr3:150409944:C>T**	TSC22D2	1	0,003088965	0,0339663	c.594C>T	p.Ser198=	Not present
**chr1:147620865:T>A**	BCL9	1	0,000370033	0,00835253	c.2710T>A	p.Ser904Thr	Not present
**chr12:120164457:C>T**	GCN1	1	8,96372E-06	0,000241196	c.1727G>A	p.Arg576His	Not present
**chr18:33739597:G>A**	ASXL3	1	0,001036066	0,0135369	c.2193G>A	p.Val731=	Present
**chr3:125576191:C>T**	OSBPL11	1	2,5921E-06	2,43096E-05	c.664G>A	p.Glu222Lys	Not present
**chr18:53397376:G>A**	DCC	1	9,736E-05	0,00072324	c.2757G>A	p.Ser919=	Present
**chr19:4442247:T>C**	CHAF1A	1	2,30498E-05	0,000723589	c.2676T>C	p.Ile892=	Not present
**chr19:18134901:G>A**	MAST3	1	0,000787519	0,00412569	c.1789G>A	p.Val597Ile	Present
**chr19:18759583:C>T**	CRTC1	1	0,003952488	0,0242718	c.657C>T	p.Pro219=	Not present
**chr19:29708294:G>A**	C19orf12	1	3,18326E-06	5,71259E-06	c.120C>T	p.Phe40=	Not present
**chr19:50697969:C>T**	SHANK1	1	0,000349715	0,00461894	c.1748-13G>A	Unknown	Present
**chr19:56781983:G>T**	ZIM2	1	0,00068403	0,00993219	c.709C>A	p.Leu237Met	Not present
**chr20:9444221:C>A**	PLCB4	1	0,003502869	0,0212458	c.2858C>A	p.Ser953Tyr	Not present
**chr20:10648732:G>A**	JAG1	1	4,77146E-06	8,56923E-06	c.1396-10C>T	Unknown	Not present
**chr20:51689156:G>A**	ATP9A	1	0,001614308	0,0188221	c.724-17C>T	Unknown	Present
**chr21:33550209:G>A**	SON	1	0,001198246	0,00424781	c.978G>A	p.Met326Ile	Present
**chr21:33575636:G>A**	SON	1	0,001794536	0,00320429	c.7074G>A	p.Gly2358=	Present
**chr22:36587493:G>A**	CACNG2	1	3,58472E-05	0,00024108	c.267C>T	p.Tyr89=	Present
**chr22:45533749:G>T**	FBLN1	1	6,56467E-05	0,00142045	c.647-12G>T	Unknown	Not present
**chr22:45547131:C>T**	FBLN1	1	0,001466032	0,010755	c.1368C>T	p.Asn456=	Not present
**chr22:50455597:G>A**	SBF1	1	0,002138911	0,02231	c.4267-15C>T	Unknown	Present
**chr8:123380659:C>T**	ATAD2	1	0	0	c.190G>A	p.Glu64Lys	Not present
**chr5:170082840:G>A**	DOCK2	1	0,002415948	0,0204024	c.5475G>A	p.Ser1825=	Not present
**chr5:150393480:G>A**	TCOF1	1	0,001115225	0,00672544	c.3712G>A	p.Ala1238Thr	Not present
**chr4:104490822:C>T**	CXXC4	1	0,000253492	0,000785593	c.981G>A	p.Arg327=	Not present
**chr17:42130468:C>T**	RAB5C	1	0,000998717	0,00216972	c.35G>A	p.Gly12Glu	Not present
**chr19:11060124:G>A**	SMARCA4	1	5,85071E-05	0,00039968	c.4848G>A	p.Pro1616=	Present
**chr19:17646041:G>A**	UNC13A	1	5,63607E-05	0,000113033	c.2115C>T	p.Val705=	Present

**chr19:35721513:C>T**	KMT2B	1	2,69594E-05	8,82119E-05	c.2166C>T	p.His722=	Present
**chr19:49597419:C>T**	PRR12	1	0,004065386	0,0261614	c.3084C>T	p.Gly1028=	Present
**chr19:56775370:G>A**	ZIM2	1	0,002536453	0,0176862	c.995C>T	p.Pro332Leu	Not present
**chr20:41445729:C>T**	CHD6	1	0,002462386	0,0132658	c.3813G>A	p.Glu1271=	Not present
**chr20:41533045:C>T**	CHD6	1	0,004170703	0,0213379	c.554+5G>A	Unknown	Not present
**chr20:41533340:T>A**	CHD6	1	0,004270604	0,0213626	c.264A>T	p.Gly88=	Not present
**chr20:64032975:C>T**	PRPF6	1	4,77783E-06	8,56903E-06	c.2808C>T	p.Arg936=	Not present
**chr22:36566439:A>G**	CACNG2	1	2,56097E-06	1,46972E-05	c.350T>C	p.Met117Thr	Present
**chr22:41146749:A>G**	EP300	1	0,003605412	0,0115718	c.2064A>G	p.Leu688=	Present
**chr1:39387313:C>T**	MACF1	1	0,000203572	0,00144648	c.14471C>T	p.Ala4824Val	Present
**chr2:240746156:C>T**	KIF1A	1	0,00164475	0,012433	c.3085G>A	p.Val1029Met	Present
**chr16:72794126:G>A**	ZFHX3	1	1,66459E-05	3,02133E-05	c.8556C>T	p.Asn2852=	Present
**chr16:72959699:C>T**	ZFHX3	1	0	0	c.447G>A	p.Gln149=	Present
**chr3:171190692:C>G**	TNIK	1	0,000110884	0,00189036	c.508+5G>C	Unknown	Not present
**chr4:3230031:G>A**	HTT	1	1,66469E-05	0,000144732	c.8254G>A	p.Val2752Ile	Not present
**chr18:21500047:C>T**	GREB1L	1	2,50528E-05	0,000130702	c.3710C>T	p.Ala1237Val	Not present
**chr17:40354331:C>T**	RARA	1	0,005190555	0,0321362	c.837C>T	p.Pro279=	Not present
**chr7:105077065:C>G**	KMT2E	1	2,17785E-05	0,00024108	c.871C>G	p.Gln291Glu	Present
**chr17:30211356:T>C**	SLC6A4	1	0,000780009	0,00374856	c.1273A>G	p.Ile425Val	Present
**chr11:61785884:G>A**	MYRF	1	0,002373752	0,00363107	c.3375+10G>A	Unknown	Not present
**chr11:78786899:G>A**	TENM4	1	1,12538E-05	5,57818E-05	c.2364C>T	p.Ile788=	Not present
**chr11:118504542:T>C**	KMT2A	1	0,001034523	0,00886845	c.8650T>C	p.Leu2884=	Present
**chr12:112261314:C>T**	HECTD4	1	0,001557898	0,00241704	c.2864G>A	p.Arg955His	Present
**chr14:22843694:G>A**	MMP14	1	3,59583E-05	0,000249501	c.851-16G>A	Unknown	Not present
**chr14:64822999:A>G**	SPTB	1	0,001056408	0,007643	c.96T>C	p.Asn32=	Not present
**chr14:64823069:T>G**	SPTB	1	0,001062686	0,00727802	c.26A>C	p.Asn9Thr	Not present
**chr15:40930657:C>T**	DLL4	1	0,003911653	0,0162548	c.369C>T	p.His123=	Not present
**chr15:41044982:G>A**	INO80	1	0,003320383	0,0128364	c.2829C>T	p.Tyr943=	Not present
**chr15:51576183:T>TAAAAAAAAAAAA**	DMXL2	1	0,009210188	0,0143051	c.88-14_88-3dup	Unknown	Present
**chr15:63669656:T>C**	HERC1	1	0,00622125	0,0104503	c.8088A>G	p.Thr2696=	Present

**chr15:82536888:C>T**	RPS17	1	0,002581848	0,0060216	c.328-7G>A	Unknown	Not present
**chr16:2109590:A>G**	PKD1	1	0,000295526	0,00236518	c.5577T>C	p.Ala1859=	Present
**chr16:2111232:C>T**	PKD1	1	0,00257997	0,0298278	c.3935G>A	p.Arg1312Gln	Present
**chr16:3729044:A>G**	CREBBP	1	0,003251418	0,0216014	c.6003T>C	p.Asn2001=	Present
**chr16:7510300:G>C**	RBFOX1	1	0,003821889	0,0273618	c.21G>C	p.Arg7Ser	Present
**chr16:89281454:G>C**	ANKRD11	1	0,000774737	0,0036162	c.5088C>G	p.Asp1696Glu	Present
**chr22:39225793:G>A**	PDGFB	1	3,58549E-05	0,000478011	c.656C>T	p.Pro219Leu	Not present
**chr5:177211313:A>G**	NSD1	1	4,99434E-05	0,000478011	c.2914A>G	p.Thr972Ala	Present
**chr4:182673090:T>C**	TENM3	1	0,006676529	0,0103664	c.1197T>C	p.Pro399=	Not present
**chr1:237792281:G>A**	RYR2	1	0,00103318	0,011262	c.13740G>A	p.Thr4580=	Not present
**chr2:28404262:G>A**	FOSL2	1	0,002410882	0,0264792	c.258G>A	p.Pro86=	Not present
**chr2:50053331:C>T**	NRXN1	1	0,004000789	0,0461198	c.4068G>A	p.Thr1356=	Present
**chr2:182942061:G>A**	NCKAP1	1	0,001803766	0,0184043	c.2695+9C>T	Unknown	Present
**chr2:189043275:G>A**	COL5A2	1	0,000849467	0,0130751	c.3364-17C>T	Unknown	Not present
**chr2:215435682:A>C**	FN1	1	0,001587733	0,0199525	c.121T>G	p.Ser41Ala	Not present
**chr3:49510792:G>C**	DAG1	1	0,003501544	0,0072324	c.258G>C	p.Leu86Phe	Not present
**chr3:50378998:G>T**	CACNA2D2	1	0,001896724	0,00293341	c.1261-5C>A	Unknown	Not present
**chr3:53740333:G>C**	CACNA1D	1	0,00065172	0,00462257	c.2805G>C	p.Leu935=	Present
**chr3:113658303:C>A**	USF3	1	0,00105496	0,0048216	c.3379G>T	p.Val1127Leu	Not present
**chr3:122254236:A>G**	CASR	1	0	0	c.47A>G	p.His16Arg	Not present
**chr3:128650754:G>C**	RPN1	1	0,002923928	0,0306687	c.47C>G	p.Thr16Ser	Not present
**chr3:194431036:T>C**	ATP13A3	1	1,1535E-05	0,000226219	c.2545-14A>G	Unknown	Not present
**chr4:1976477:C>T**	NSD2	1	1,27466E-05	2,28769E-05	c.3624C>T	p.Thr1208=	Present
**chr4:102613421:A>G**	NFKB1	1	0,001742719	0,017179	c.2593-4A>G	Unknown	Not present
**chr4:110618482:A>C**	PITX2	1	0,003332326	0,0371451	c.618T>G	p.Ser206=	Not present
**chr4:125317150:C>A**	FAT4	1	0,004548951	0,0088854	c.739C>A	p.Pro247Thr	Not present
**chr4:143521452:C>T**	SMARCA5	1	0,005579116	0,0218114	c.276C>T	p.Phe92=	Not present
**chr1:237798057:G>A**	RYR2	1	0,002838331	0,0324241	c.13977G>A	p.Glu4659=	Not present
**chr1:237503283:C>T**	RYR2	1	0,004002232	0,0322977	c.2397-6C>T	Unknown	Not present
**chr1:237792141:C>T**	RYR2	1	3,05577E-05	9,36944E-05	c.13600C>T	p.Pro4534Ser	Not present
**chr19:46608866:C>T**	CALM3	1	0,00068827	0,00680812	c.306C>T	p.Ser102=	Not present
**chr5:141526422:G>A**	DIAPH1	1	7,55434E-05	0,000473037	c.3313C>T	p.Arg1105Trp	Not present

**chr9:95506477:T>C**	PTCH1	1	0,000129462	0,000393177	c.324A>G	p.Ile108Met	Not present
**chr8:67200293:C>T**	ARFGEF1	1	0,0002485	0,00146987	c.4870G>A	p.Ala1624Thr	Not present
**chr2:211428453:T>C**	ERBB4	1	8,61874E-05	0,00094697	c.2674A>G	p.Ile892Val	Not present
**chr1:27551646:G>A**	AHDC1	1	0,000700081	0,00840114	c.470C>T	p.Ala157Val	Present
**chr3:174279420:G>A**	NLGN1	1	3,33289E-05	9,63995E-05	c.1479G>A	p.Ala493=	Present
**chr6:75119174:G>A**	COL12A1	1	0,000645505	0,001191	c.7223C>T	p.Thr2408Met	Present
**chr4:113360843:C>T**	ANK2	1	0,00013982	0,000724638	c.10702C>T	p.Arg3568Trp	Present
**chr9:134700062:C>A**	COL5A1	1	5,64347E-05	0,000718297	c.431C>A	p.Thr144Lys	Not present
**chr16:24797570:G>A**	TNRC6A	1	9,65897E-05	0,00242014	c.3642G>A	p.Ser1214=	Not present
**chr6:142774499:T>A**	HIVEP2	1	0,000330322	0,0034475	c.240A>T	p.Ala80=	Present
**chr16:14217187:A>G**	MRTFB	1	7,6836E-05	0,000723589	c.414A>G	p.Ala138=	Present
**chr5:143054436:T>C**	ARHGAP26	1	0,000331076	0,0038961	c.1286-3T>C	Unknown	Not present
**chr15:80404543:C>T**	ARNT2	1	9,67717E-05	0,000966184	c.28C>T	p.Pro10Ser	Present
**chr5:131672519:C>T**	FNIP1	1	0,000294634	0,00409836	c.1925G>A	p.Gly642Glu	Not present
**chr1:27550706:G>A**	AHDC1	1	0,002004478	0,0228857	c.1410C>T	p.Gly470=	Present
**chr1:109271497:C>G**	CELSR2	1	0,000106278	0,00144648	c.7788C>G	p.Thr2596=	Not present
**chr3:196885429:A>G**	SENP5	1	0,002474709	0,0112468	c.248A>G	p.Asn83Ser	Not present
**chr4:114623498:G>T**	UGT8	1	0,003190994	0,0161524	c.618G>T	p.Gly206=	Not present
**chr1:19176684:C>A**	UBR4	1	0,000676079	0,00344828	c.2681G>T	p.Gly894Val	Not present
**chr1:109251694:C>T**	CELSR2	1	0,000467359	0,00094697	c.1615C>T	p.Arg539Cys	Not present
**chr1:183133497:G>A**	LAMC1	1	0,003669962	0,00662879	c.3796G>A	p.Glu1266Lys	Not present
**chr1:244855607:G>A**	HNRNPU	1	0,006610836	0,011396	c.2169C>T	p.Ala723=	Present
**chr2:112550919:C>T**	POLR1B	1	9,54268E-06	3,02151E-05	c.679C>T	p.His227Tyr	Not present
**chr2:189081035:A>C**	COL5A2	1	3,1836E-06	5,71883E-06	c.861T>G	p.Arg287=	Not present
**chr2:215397236:A>G**	FN1	1	6,57263E-06	0,000065445	c.3518-13T>C	Unknown	Not present
**chr2:239108191:G>A**	HDAC4	1	0,000242245	0,000430293	c.1979-8C>T	Unknown	Present
**chr3:12901409:G>A**	IQSEC1	1	0,000168188	0,000290245	c.2919C>T	p.Phe973=	Not present
**chr3:42201046:C>T**	TRAK1	1	0,004852542	0,00693934	c.1419C>T	p.Ala473=	Not present
**chr3:47084394:T>TA**	SETD2	1	0,003261488	0,0234342	c.5398-13dup	Unknown	Present

**chr3:129265744:C>T**	COPG1	1	3,32936E-05	0,000385654	c.1420C>T	p.Arg474Cys	Not present
**chr3:173800298:C>T**	NLGN1	1	0,003444021	0,00787454	c.499C>T	p.Leu167Phe	Present
**chr6:24842955:A>G**	RIPOR2	1	0,003993363	0,00642398	c.1764T>C	p.Asn588=	Not present
**chr6:33451876:C>T**	SYNGAP1	1	0,000149716	0,00102824	c.4002C>T	p.Asn1334=	Present
**chr6:56487154:C>T**	DST	1	0,008196406	0,0123765	c.20997G>A	p.Leu6999=	Present
**chr6:157110528:G>A**	ARID1B	1	0,000779857	0,0158684	c.2548G>A	p.Ala850Thr	Present
**chr7:18762285:C>T**	HDAC9	1	2,69311E-05	0,00014346	c.2164+8C>T	Unknown	Not present
**chr7:83162337:G>T**	PCLO	1	0,000589143	0,00278674	c.248+8C>A	Unknown	Present
**chr1:181733455:G>A**	CACNA1E	1	0,000787634	0,00565078	c.2967G>A	p.Val989=	Present
**chr1:1535425:G>A**	TMEM240	1	8,79355E-05	0,000526064	c.456C>T	p.Ala152=	Not present
**chr9:35547151:G>A**	RUSC2	1	0	0	c.630G>A	p.Gly210=	Not present
**chr19:49480427:G>A**	FLT3LG	1	8,18154E-05	0,00117925	c.611G>A	p.Cys204Tyr	Not present
**chr7:152181521:T>G**	KMT2C	1	0,007174705	0,0116922	c.6339A>C	p.Ser2113=	Present
**chr5:38959841:A>C**	RICTOR	1	1,79344E-05	4,40995E-05	c.1989T>G	p.Ile663Met	Present
**chr22:41522970:A>G**	ACO2	1	4,48161E-05	0,00072324	c.1279A>G	p.Ile427Val	Not present
**chr12:49033255:G>T**	KMT2D	1	9,66576E-06	1,87216E-05	c.11450C>A	p.Ala3817Asp	Not present
**chr3:38587455:A>G**	SCN5A	1	4,77286E-06	0,00024108	c.2381T>C	p.Met794Thr	Not present
**chr15:33663730:A>T**	RYR3	1	1,61407E-06	2,88554E-06	c.5612A>T	p.Gln1871Leu	Not present
**chr3:48641932:C>G**	CELSR3	1	8,31558E-05	0,000673066	c.8743G>C	p.Asp2915His	Not present
**chr1:236749146:C>A**	ACTN2	1	1,79286E-05	3,42763E-05	c.1538C>A	p.Thr513Asn	Not present
**chr17:56594566:G>A**	NOG	1	3,27533E-06	2,44224E-05	c.343G>A	p.Ala115Thr	Not present
**chr6:50836129:G>A**	TFAP2B	1	0,00027272	0,00371712	c.670G>A	p.Val224Ile	Not present
**chr17:28534952:G>A**	FOXN1	1	6,36286E-06	1,14264E-05	c.1381G>A	p.Gly461Ser	Not present
**chr3:129605802:C>G**	PLXND1	1	0,000139086	0,00121124	c.838G>C	p.Val280Leu	Not present
**chr12:908664:G>A**	WNK1	1	0,000559483	0,000999559	c.7021G>A	p.Gly2341Ser	Not present
**chr4:182793277:C>T**	TENM3	1	0,000120407	0,000288184	c.6605C>T	p.Thr2202Met	Not present
**chr3:51381524:C>T**	DOCK3	1	0,000326093	0,000485381	c.6058C>T	p.Arg2020Cys	Not present
**chr4:48535666:A>G**	FRYL	1	0,000349924	0,00237643	c.6555T>C	p.Tyr2185=	Present
**chr5:83520356:A>G**	VCAN	1	2,5657E-05	0,00094697	c.2050A>G	p.Arg684Gly	Not present
**chr8:13393608:G>A**	DLC1	1	2,56073E-05	9,06399E-05	c.1259C>T	p.Thr420Met	Not present

**chr10:60073432:C>T**	ANK3	1	4,22668E-05	0,000216659	c.7449G>A	p.Ser2483=	Present
**chr15:38349488:T>G**	SPRED1	1	3,18825E-06	5,72938E-06	c.649T>G	p.Ser217Ala	Not present
**chr19:2127198:C>T**	AP3D1	1	0,001475648	0,00223958	c.810G>A	p.Thr270=	Not present
**chr19:35654555:A>G**	COX6B1	1	0,000850765	0,0173578	c.107-16A>G	Unknown	Not present
**chr19:35728970:A>C**	KMT2B	1	0,000853026	0,0168756	c.4688-15A>C	Unknown	Present
**chr19:35732798:G>T**	KMT2B	1	0,000902873	0,0169328	c.6249G>T	p.Thr2083=	Present
**chr20:2463081:C>A**	SNRPB	1	3,20176E-05	0,000261097	c.559+8G>T	Unknown	Not present
**chr20:10642618:T>G**	JAG1	1	0,00717522	0,0113422	c.2459-17A>C	Unknown	Not present
**chr20:47637636:C>A**	NCOA3	1	0,000143576	0,00189573	c.2377-12C>A	Unknown	Not present
**chr21:45175779:C>T**	ADARB1	1	1,4315E-05	0,000137199	c.78C>T	p.Ser26=	Not present
**chr22:40419116:A>C**	MRTFA	1	0,001833175	0,0147181	c.1622T>G	p.Val541Gly	Not present
**chr1:203703795:G>A**	ATP2B4	1	0	0	c.1081G>A	p.Val361Ile	Not present
**chr17:3018095:C>T**	RAP1GAP2	1	2,37208E-05	0,000487805	c.1529C>T	p.Thr510Ile	Not present
**chr20:34741210:T>G**	NCOA6	1	3,84092E-05	0,00048216	c.5046A>C	p.Pro1682=	Not present
**chr20:34754842:A>G**	NCOA6	1	0,000119067	0,00241779	c.1555T>C	p.Phe519Leu	Not present
**chr2:221426074:A>G**	EPHA4	1	1,79255E-05	0,000339098	c.2915T>C	p.Met972Thr	Not present
**chr2:217831460:G>A**	TNS1	1	3,19466E-05	0,00244858	c.3368C>T	p.Thr1123Ile	Not present
**chr1:19126617:T>C**	UBR4	1	0,000696648	0,00344828	c.9267A>G	p.Leu3089=	Not present
**chr1:19146980:T>C**	UBR4	1	0,00067912	0,0034087	c.7650A>G	p.Lys2550=	Not present
**chr1:19157944:G>A**	UBR4	1	0,000629892	0,00339136	c.5631C>T	p.Tyr1877=	Not present
**chr19:16869857:C>T**	SIN3B	1	0,00460367	0,00800582	c.2204C>T	p.Pro735Leu	Present
**chr18:26035040:C>A**	SS18	1	0,001844257	0,00600916	c.1061G>T	p.Gly354Val	Not present
**chr10:77828904:G>A**	DLG5	1	6,01817E-05	0,000621118	c.2267C>T	p.Ala756Val	Not present
**chr18:3456460:C>T**	TGIF1	1	0,001652717	0,00340584	c.123C>T	p.Asn41=	Not present
**chr11:1250017:G>A**	MUC5B	1	0,00210536	0,0111875	c.13137G>A	p.Thr4379=	Not present
**chr11:1250169:C>T**	MUC5B	1	0,000110391	0,000728509	c.13289C>T	p.Thr4430Met	Not present
**chr11:46542503:C>T**	AMBRA1	1	0,001491505	0,00300868	c.1514G>A	p.Arg505His	Not present
**chr11:68439859:C>T**	LRP5	1	0,002539148	0,00397234	c.4431C>T	p.His1477=	Not present
**chr11:78672224:C>T**	TENM4	1	0,00182213	0,0210923	c.5602G>A	p.Ala1868Thr	Not present
**chr12:49053016:C>T**	KMT2D	1	3,07332E-05	0,000395614	c.1011G>A	p.Ser337=	Not present
**chr13:28311673:G>A**	FLT1	1	0,002093588	0,00378788	c.3552C>T	p.Tyr1184=	Not present
**chr15:48487206:G>A**	FBN1	1	7,68321E-05	0,000395659	c.3464-6C>T	Unknown	Present

**chr15:63686389:T>C**	HERC1	1	0,000996113	0,00165284	c.6195A>G	p.Thr2065=	Present
**chr16:2111149:G>A**	PKD1	1	0,003012861	0,00442395	c.4018C>T	p.Arg1340Trp	Present
**chr16:9764849:G>T**	GRIN2A	1	4,77124E-06	8,56908E-06	c.2695C>A	p.Arg899=	Present
**chr16:30723987:T>A**	SRCAP	1	0,000618417	0,00119104	c.4563T>A	p.Pro1521=	Present
**chr17:1354236:T>C**	YWHAE	1	0,000165194	0,00096432	c.690A>G	p.Leu230=	Present
**chr17:35844679:CGGCGGCTATGGTGGGGACAGAGGA>C**	TAF15	1	0,001749578	0,0122736	c.1392_1415del	p.Gly478_Gly485del	Not present
**chr17:37687346:C>T**	HNF1B	1	2,4335E-05	0,000037132	c.1308G>A	p.Ala436=	Not present
**chr17:76045352:T>A**	SRP68	1	0,004306403	0,00803629	c.1334A>T	p.Glu445Val	Not present
**chr17:77402183:C>T**	SEPTIN9	1	0,002341548	0,00336745	c.201C>T	p.Gly67=	Not present
**chr1:12319613:AGTTT>A**	VPS13D	1	0	0	c.7537_7540del	p.Phe2513AlafsTer9	Not present
**chr3:185937850:C>A**	TRA2B	1	0	0	c.11G>T	p.Ser4Ile	Not present
**chr4:145150795:C>G**	OTUD4	1	0,004379762	0,00603866	c.1072+7G>C	Unknown	Not present
**chr9:93658132:G>T**	PHF2	1	0,005220979	0,048946	c.1148-13G>T	Unknown	Present
**chr9:128751227:G>A**	ZER1	1	0,001673935	0,0145068	c.1080C>T	p.Ile360=	Not present
**chr9:135486076:C>T**	PPP1R26	1	0,002012882	0,0246467	c.1566C>T	p.Asp522=	Not present
**chr9:135488116:C>T**	PPP1R26	1	0,000654405	0,00730509	c.3606C>T	p.Gly1202=	Not present
**chr9:136921160:C>T**	TRAF2	1	0,003972848	0,0459427	c.1083C>T	p.Phe361=	Not present
**chr10:43387774:A>G**	HNRNPF	1	0,000272787	0,00160037	c.111T>C	p.His37=	Present
**chr10:68959922:C>A**	DDX21	1	0,000141677	0,00102564	c.204C>A	p.Asp68Glu	Not present
**chr10:91940024:A>G**	BTAF1	1	2,69463E-05	0,000720084	c.211A>G	p.Lys71Glu	Present
**chr11:46784521:G>A**	CKAP5	1	0,006462609	0,00940389	c.2121C>T	p.Ala707=	Not present
**chr11:59657549:A>G**	PATL1	1	0,000454477	0,00484	c.602T>C	p.Val201Ala	Not present
**chr11:76463865:C>A**	EMSY	1	0,004147279	0,0407215	c.661C>A	p.Arg221=	Present
**chr11:119133135:A>G**	HINFP	1	0,004627911	0,0073203	c.1055A>G	p.Lys352Arg	Not present
**chr11:133921085:G>A**	IGSF9B	1	0,000918381	0,0108947	c.2640C>T	p.Ala880=	Not present
**chr12:19375517:C>T**	PLEKHA5	1	0,001156373	0,00305936	c.*12-14C>T	Unknown	Not present
**chr12:44532735:A>G**	NELL2	1	0,003452889	0,0372685	c.1664-14T>C	Unknown	Not present
**chr12:44876669:G>A**	NELL2	1	0,00388631	0,0432224	c.57C>T	p.Ala19=	Not present
**chr12:53019941:G>C**	EIF4B	1	0,000323287	0,000509136	c.392G>C	p.Ser131Thr	Not present
**chr12:62303422:G>A**	USP15	1	0,001068137	0,00382702	c.349-10G>A	Unknown	Present
**chr9:109320221:A>C**	EPB41L4B	1	8,9952E-05	0,00105116	c.226T>G	p.Ser76Ala	Not present

**chr9:68781004:A>G**	PABIR1	1	0,000660099	0,00104712	c.840A>G	p.Pro280=	Not present
**chr12:107649269:A>G**	ABTB3	1	0,000145956	0,00189215	c.2986A>G	p.Met996Val	Not present
**chr9:32630387:T>G**	TAF1L	1	0,003959831	0,0072324	c.5193A>C	p.Lys1731Asn	Not present
**chr4:158868403:G>A**	FNIP2	1	0,002753205	0,020974	c.1767G>A	p.Glu589=	Not present
**chr5:133980821:G>A**	VDAC1	1	0,000219195	0,00186609	c.459C>T	p.Tyr153=	Not present
**chr6:43130257:G>A**	PTK7	1	0,001707355	0,018621	c.498G>A	p.Gly166=	Present
**chr6:43222516:C>G**	CUL9	1	0,001280587	0,0096432	c.6922-15C>G	Unknown	Not present
**chr6:43572565:C>T**	XPO5	1	0,00356345	0,0363168	c.241G>A	p.Gly81Ser	Not present
**chr6:63684220:A>G**	PHF3	1	0,002375306	0,0261043	c.498A>G	p.Val166=	Present
**chr6:63684419:G>A**	PHF3	1	0,002047611	0,0222875	c.697G>A	p.Gly233Arg	Present
**chr6:63713333:G>A**	PHF3	1	0,001872247	0,020527	c.5745G>A	p.Arg1915=	Present
**chr6:89668083:A>G**	MDN1	1	0,000731151	0,0185632	c.14025T>C	p.Tyr4675=	Not present
**chr6:152975337:T>C**	FBXO5	1	0,007379545	0,011231	c.388A>G	p.Thr130Ala	Not present
**chr7:5593257:G>T**	FSCN1	1	0,000474311	0,00456819	c.321G>T	p.Ala107=	Not present
**chr7:32875170:T>C**	KBTBD2	1	1,11863E-05	0,000151644	c.171-13A>G	Unknown	Not present
**chr7:56058098:G>A**	CCT6A	1	1,59295E-06	2,8613E-06	c.720G>A	p.Glu240=	Not present
**chr7:103316937:T>C**	DNAJC2	1	0,008442741	0,0291923	c.1320A>G	p.Thr440=	Not present
**chr7:117322976:A>C**	WNT2	1	0,001206828	0,0121392	c.14T>G	p.Leu5Arg	Not present
**chr7:149436652:G>A**	ZNF777	1	0,000472529	0,00531254	c.1262C>T	p.Thr421Met	Not present
**chr8:38289486:T>C**	NSD3	1	2,3067E-05	0,000340909	c.3138A>G	p.Lys1046=	Not present
**chr12:70569818:C>T**	PTPRB	1	0,001675063	0,00520833	c.3491G>A	p.Ser1164Asn	Present
**chr12:117524956:C>T**	KSR2	1	0,000964408	0,00196104	c.2115G>A	p.Lys705=	Not present
**chr22:18606829:T>G**	RIMBP3	1	0	0	c.4606A>C	p.Asn1536His	Not present
**chr16:50153888:C>T**	TENT4B	1	0	0	c.267C>T	p.Arg89=	Not present
**chr17:1125231:G>A**	ABR	1	0,001726815	0,00993457	c.198C>T	p.Gly66=	Not present
**chr17:36175062:A>G**	TBC1D3B	1	0,000133423	0,000579912	c.-1-11T>C	Unknown	Not present
**chr17:38715771:G>A**	MLLT6	1	0,004545291	0,00696977	c.979G>A	p.Ala327Thr	Not present
**chr17:38716669:G>T**	MLLT6	1	0,004656167	0,00715945	c.1339G>T	p.Ala447Ser	Not present
**chr17:64505739:G>A**	DDX5	1	0,004168326	0,0459449	c.44+7C>T	Unknown	Not present
**chr18:76899174:C>T**	ZNF236	1	0,004293115	0,0370789	c.1846C>T	p.Pro616Ser	Not present

**chr18:76925217:C>T**	ZNF236	1	0,004010356	0,00835253	c.3690C>T	p.Cys1230=	Not present
**chr18:76955994:G>A**	ZNF236	1	2,06254E-05	8,81731E-05	c.4924G>A	p.Val1642Ile	Not present
**chr19:808631:C>T**	PTBP1	1	0,004315907	0,0449693	c.1332C>T	p.Arg444=	Not present
**chr19:3282449:G>C**	CELF5	1	0,003728169	0,0438841	c.990G>C	p.Gly330=	Not present
**chr19:10450585:A>ATT**	PDE4A	1	0,000568815	0,00136388	c.621-8_621-7dup	Unknown	Not present
**chr19:32353350:G>T**	ZNF507	1	0,000230447	0,00162028	c.520G>T	p.Val174Leu	Not present
**chr19:36341148:T>C**	ZFP14	1	0,003960003	0,0439697	c.678A>G	p.Lys226=	Not present
**chr19:46388681:G>A**	PPP5C	1	0	0	c.1305G>A	p.Glu435=	Present
**chr19:54456053:C>T**	LENG8	1	6,16586E-05	0,000774593	c.1112C>T	p.Ser371Leu	Not present
**chr21:25751975:T>A**	GABPA	1	0,000359545	0,00359524	c.308-14T>A	Unknown	Not present
**chr21:28986890:C>T**	LTN1	1	1,15245E-05	0,00013089	c.87G>A	p.Gln29=	Not present
**chr16:85170606:C>T**	GSE1	1	0,006727311	0,0199144	c.749C>T	p.Ala250Val	Not present
**chr16:24571551:A>G**	RBBP6	1	0,002134496	0,00542129	c.4485A>G	p.Thr1495=	Not present
**chr12:124344922:C>T**	NCOR2	1	0,00100586	0,013712	c.4389G>A	p.Ala1463=	Not present
**chr16:18870687:T>C**	SMG1	1	0,001145754	0,00236295	c.2415A>G	p.Gln805=	Not present
**chr12:124912839:G>A**	UBC	1	0,002420722	0,0271349	c.933C>T	p.Thr311=	Not present
**chr12:129700424:T>C**	TMEM132D	1	0,003797861	0,00543942	c.354A>G	p.Gly118=	Not present
**chr12:131725440:C>A**	SFSWAP	1	2,94462E-05	0,00024108	c.642C>A	p.Arg214=	Not present
**chr13:46741762:A>G**	LRCH1	1	0,00109719	0,0126275	c.2206A>G	p.Arg736Gly	Not present
**chr13:77753279:G>A**	SLAIN1	1	0,000757803	0,00793381	c.1335G>A	p.Pro445=	Not present
**chr14:22924045:C>G**	PRMT5	1	5,92568E-05	0,00050226	c.1338G>C	p.Ser446=	Not present
**chr14:24375980:G>A**	NFATC4	1	0,001849305	0,0222674	c.1935G>A	p.Thr645=	Not present
**chr14:32093527:A>G**	ARHGAP5	1	0,003663022	0,0230566	c.2858A>G	p.Asn953Ser	Present
**chr14:57208758:G>C**	EXOC5	1	3,86198E-05	0,000244978	c.1978C>G	p.Leu660Val	Present
**chr14:90872124:C>T**	RPS6KA5	1	0,001599537	0,00576701	c.2359G>A	p.Asp787Asn	Not present
**chr15:22927986:C>T**	CYFIP1	1	0,003955082	0,0455836	c.1153G>A	p.Ala385Thr	Present
**chr15:29718809:A>C**	TJP1	1	0,000466076	0,00559512	c.3333T>G	p.Ser1111=	Not present
**chr15:59081139:G>A**	RNF111	1	0,001563532	0,019253	c.2152G>A	p.Ala718Thr	Not present
**chr15:66501517:C>A**	RPL4	1	0,000156261	0,00214884	c.547-13G>T	Unknown	Not present
**chr15:72208850:A>G**	PKM	1	0,001856613	0,0213591	c.607T>C	p.Leu203=	Not present

**chr15:72266787:C>T**	PARP6	1	0,001425056	0,0167734	c.39G>A	p.Ser13=	Not present
**chr15:90492595:G>A**	IQGAP1	1	0,004639711	0,0137482	c.4512G>A	p.Val1504=	Not present
**chr20:40688398:G>A**	MAFB	1	1,70649E-06	2,99009E-06	c.453C>T	p.Ala151=	Not present
**chr1:12333301:C>G**	VPS13D	1	2,17661E-05	0,00313707	c.8363C>G	p.Pro2788Arg	Not present
**chr19:5693403:G>A**	LONP1	1	0,000193615	0,00168756	c.2598C>T	p.Ser866=	Not present
**chr1:181733553:C>T**	CACNA1E	1	0,002982605	0,0164364	c.3065C>T	p.Thr1022Met	Present
**chr4:113369717:C>T**	ANK2	1	4,22627E-05	0,000343849	c.11522C>T	p.Pro3841Leu	Present
**chr1:3405533:C>T**	PRDM16	1	0,002762094	0,028891	c.1071C>T	p.Arg357=	Not present
**chr1:11257178:G>C**	MTOR	1	0,001115884	0,0089545	c.272-13C>G	Unknown	Present
**chr1:15919484:A>T**	SPEN	1	0,001319565	0,00969402	c.1602A>T	p.Thr534=	Present
**chr1:15931150:T>C**	SPEN	1	0,003436215	0,0378659	c.4910T>C	p.Val1637Ala	Present
**chr1:15933897:A>G**	SPEN	1	0,002023301	0,024302	c.7657A>G	p.Ser2553Gly	Present
**chr1:21721081:C>A**	USP48	1	0	0	c.1849G>T	p.Ala617Ser	Not present
**chr1:24342974:C>T**	GRHL3	1	0,00799496	0,0195275	c.1368C>T	p.Pro456=	Not present
**chr1:24344916:C>A**	GRHL3	1	0,008268286	0,0193373	c.1439C>A	p.Pro480His	Not present
**chr1:27548852:G>A**	AHDC1	1	0,008439522	0,03197	c.3264C>T	p.Ser1088=	Present
**chr1:39317341:G>T**	MACF1	1	0,006198427	0,0379245	c.3716G>T	p.Arg1239Leu	Present
**chr1:39333159:C>T**	MACF1	1	0,005728706	0,0377572	c.6571C>T	p.His2191Tyr	Present
**chr1:39411381:G>A**	MACF1	1	0,001505939	0,00461361	c.1116G>A	p.Val372=	Present
**chr1:65392454:T>A**	DNAJC6	1	0,001199911	0,00575043	c.1492T>A	p.Cys498Ser	Not present
**chr1:81952012:A>G**	ADGRL2	1	0,005585633	0,0101058	c.1664A>G	p.Lys555Arg	Not present
**chr1:109004733:C>CA**	WDR47	1	3,32078E-05	0,000215544	c.1152-16dup	Unknown	Not present
**chr1:159193412:A>G**	CADM3	1	0,000247583	0,0019973	c.383-11A>G	Unknown	Not present
**chr1:15876350:G>A**	SPEN	1	0,000131917	0,00183607	c.553G>A	p.Ala185Thr	Present
**chr3:49654863:G>T**	BSN	1	4,7766E-06	3,02297E-05	c.5307G>T	p.Val1769=	Present
**chr15:63652489:T>G**	HERC1	1	4,80454E-06	8,62843E-06	c.10343A>C	p.Asn3448Thr	Present
**chr1:27548881:T>C**	AHDC1	1	2,57428E-06	2,77716E-05	c.3235A>G	p.Thr1079Ala	Present
**chr19:31278387:T>C**	TSHZ3	1	0,009301438	0,0318226	c.1406A>G	p.Glu469Gly	Present
**chr19:40257008:G>A**	AKT2	1	0,003665466	0,0112041	c.93C>T	p.Ser31=	Not present
**chr20:8657216:A>G**	PLCB1	1	0,001592536	0,026916	c.627A>G	p.Pro209=	Present

**chr21:33554082:A>G**	SON	1	0,001071612	0,0074928	c.4851A>G	p.Ala1617=	Present
**chr21:39196968:T>C**	BRWD1	1	3,07327E-05	0,000206868	c.6101A>G	p.Asn2034Ser	Not present
**chr8:132137928:C>T**	KCNQ3	1	0	0	c.1657G>A	p.Gly553Arg	Present
**chr22:31809618:C>T**	DEPDC5	1	0	0	c.1295C>T	p.Ser432Phe	Present
**chr1:149926033:G>A**	SF3B4	1	2,93987E-06	1,46981E-05	c.716C>T	p.Pro239Leu	Not present
**chr13:110187278:G>A**	COL4A1	1	0,000392261	0,000660045	c.1588C>T	p.Pro530Ser	Not present
**chr12:101685647:C>T**	MYBPC1	1	6,5489E-05	0,000161722	c.3385C>T	p.Arg1129Ter	Not present
**chr1:26775020:G>A**	ARID1A	1	3,25451E-06	1,46666E-05	c.4793G>A	p.Arg1598His	Present
**chr22:27750986:C>T**	MN1	1	2,86849E-05	0,000160868	c.3892G>A	p.Val1298Met	Not present
**chr15:88858209:G>A**	ACAN	1	6,40415E-06	9,07057E-05	c.5624G>A	p.Ser1875Asn	Not present
**chr12:79786420:T>C**	PPP1R12A	1	0,000194124	0,00123274	c.2861A>G	p.Asn954Ser	Not present
**chr1:22913757:A>T**	EPHB2	1	0,003461214	0,0405236	c.3055A>T	p.Lys1019Ter	Present
**chr5:143399990:T>C**	NR3C1	1	1,53631E-05	6,53424E-05	c.850A>G	p.Ile284Val	Not present
**chr1:180824808:G>T**	XPR1	1	0,00746498	0,0484429	c.819G>T	p.Arg273=	Not present
**chr1:196677525:T>C**	CFH	1	0,004330692	0,0498924	c.477T>C	p.Ser159=	Not present
**chr6:142770599:G>A**	HIVEP2	1	0,002140571	0,0260366	c.4140C>T	p.Thr1380=	Present
**chr1:196713826:A>G**	CFH	1	0,004459133	0,0498582	c.1428A>G	p.Gln476=	Not present
**chr4:55118758:A>G**	KDR	1	0,00228186	0,0225204	c.204T>C	p.Ser68=	Not present
**chr4:76741336:G>A**	SHROOM3	1	0,000113206	0,00100978	c.3163G>A	p.Ala1055Thr	Not present
**chr4:125491128:G>A**	FAT4	1	5,76184E-05	0,00183007	c.14312G>A	p.Arg4771His	Not present
**chr4:182754558:G>A**	TENM3	1	0,001516316	0,0162239	c.4191G>A	p.Ala1397=	Not present
**chr4:182755113:C>T**	TENM3	1	0,001305065	0,0141856	c.4746C>T	p.Asn1582=	Not present
**chr5:38953532:G>A**	RICTOR	1	0,002670748	0,00753799	c.2719C>T	p.Arg907Cys	Present
**chr5:128345408:C>A**	FBN2	1	0,00219319	0,0248003	c.3166G>T	p.Ala1056Ser	Not present
**chr5:128392160:G>A**	FBN2	1	0,002532392	0,0282837	c.1466-5C>T	Unknown	Not present
**chr5:138429848:G>A**	KDM3B	1	0,001600627	0,0084358	c.4776G>A	p.Glu1592=	Present
**chr5:161336785:TAC>T**	GABRB2	1	0,007886398	0,0207792	c.542-18_542-17del	Unknown	Present
**chr5:161459644:G>C**	GABRB2	1	0,001796346	0,0179456	c.438C>G	p.Gly146=	Present
**chr5:170076012:C>T**	DOCK2	1	4,22591E-05	0,00024108	c.4794C>T	p.Phe1598=	Not present
**chr5:180629830:C>T**	FLT4	1	2,82308E-05	0,00016976	c.682G>A	p.Glu228Lys	Not present
**chr6:35242718:G>C**	SCUBE3	1	2,30496E-05	0,000339136	c.1631G>C	p.Gly544Ala	Not present

**chr6:75130250:C>A**	COL12A1	1	0,000238117	0,00236518	c.6068-17G>T	Unknown	Present
**chr6:75184132:G>A**	COL12A1	1	1,59206E-06	0,000242014	c.1010C>T	p.Pro337Leu	Present
**chr6:87256606:G>A**	ZNF292	1	0,003363418	0,0401945	c.2977G>A	p.Asp993Asn	Present
**chr4:46928493:C>T**	GABRA4	1	0,001524664	0,00530888	c.1397G>A	p.Arg466Gln	Present
**chr4:3125535:A>G**	HTT	1	0,000402398	0,00345622	c.1322-14A>G	Unknown	Not present
**chr4:3121242:G>C**	HTT	1	0,002367447	0,0239629	c.1083G>C	p.Thr361=	Not present
**chr2:169294717:TAAAAAA>T**	LRP2	1	0,008421704	0,0132367	c.428-13_428-8del	Unknown	Present
**chr1:196715725:T>C**	CFH	1	0,004375414	0,0496896	c.1652T>C	p.Ile551Thr	Not present
**chr1:198709705:A>G**	PTPRC	1	0,002799927	0,0351173	c.1052A>G	p.Asn351Ser	Present
**chr1:201900588:C>T**	LMOD1	1	0,004738912	0,0485549	c.425G>A	p.Gly142Asp	Not present
**chr1:209795422:C>T**	IRF6	1	0,000175472	0,00123031	c.380-4G>A	Unknown	Not present
**chr1:237496595:A>G**	RYR2	1	0,002029735	0,0226065	c.2046A>G	p.Thr682=	Not present
**chr2:46376608:G>A**	EPAS1	1	0,000934654	0,00144075	c.1104G>A	p.Met368Ile	Not present
**chr2:80393245:T>C**	CTNNA2	1	0,00438423	0,0479307	c.1091T>C	p.Ile364Thr	Present
**chr3:4693723:C>G**	ITPR1	1	0,002314744	0,0153454	c.4263C>G	p.His1421Gln	Present
**chr4:2926652:C>T**	ADD1	1	2,30597E-05	0,000207555	c.1965C>T	p.Cys655=	Not present
**chr3:4702916:C>T**	ITPR1	1	0,004404434	0,0478693	c.4623C>T	p.Ser1541=	Present
**chr3:9058299:G>C**	SRGAP3	1	0,004067153	0,0438588	c.975C>G	p.Val325=	Present
**chr3:63996301:A>G**	ATXN7	1	0,000781444	0,00457782	c.2479A>G	p.Thr827Ala	Not present
**chr3:123296132:G>T**	ADCY5	1	0,003970988	0,0444846	c.3015C>A	p.Ala1005=	Present
**chr3:139358239:C>T**	COPB2	1	0,00093478	0,00727606	c.2586G>A	p.Pro862=	Not present
**chr3:149526142:G>T**	WWTR1	1	0,000310741	0,00432202	c.906-17C>A	Unknown	Not present
**chr4:1212975:G>A**	CTBP1	1	5,6351E-05	0,000297292	c.1044C>T	p.Ala348=	Not present
**chr19:4429462:G>A**	CHAF1A	1	7,17198E-05	0,00144009	c.1629G>A	p.Gly543=	Not present
**chr16:72797656:T>C**	ZFHX3	1	0,002162424	0,0147724	c.5026A>G	p.Ser1676Gly	Present
**chr16:24777228:G>A**	TNRC6A	1	0,009842908	0,0196732	c.459G>A	p.Gln153=	Not present
**chr16:17117977:G>A**	XYLT1	1	0,001482824	0,0100806	c.2226C>T	p.Val742=	Not present
**chr1:9720005:C>T**	PIK3CD	1	6,57004E-06	0,000014699	c.1327C>T	p.Pro443Ser	Not present
**chr2:165162674:G>C**	SCN3A	1	2,56113E-05	0,00048216	c.849C>G	p.Ser283Arg	Present
**chr2:99594041:A>T**	AFF3	1	0	0	c.1620T>A	p.Pro540=	Not present
**chr11:64667525:C>T**	NRXN2	1	0,00079134	0,00637479	c.1523G>A	p.Arg508His	Present

**chr8:47823996:C>T**	PRKDC	1	0,000394749	0,00249252	c.8784G>A	p.Lys2928=	Present
**chr18:33739137:C>T**	ASXL3	1	8,07227E-05	0,00096432	c.1733C>T	p.Ser578Phe	Present
**chr22:27798529:G>A**	MN1	1	0,000929845	0,00130913	c.2015C>T	p.Ser672Leu	Not present
**chr16:72950840:C>T**	ZFHX3	1	0,001855223	0,0185374	c.2845G>A	p.Val949Ile	Present
**chr19:10180853:G>A**	DNMT1	1	0,001669727	0,00330813	c.150C>T	p.His50=	Not present
**chr8:105802755:C>G**	ZFPM2	1	2,94766E-05	0,00024108	c.2673C>G	p.Asp891Glu	Not present
**chr14:88594797:G>A**	ZC3H14	1	6,27568E-05	0,000640146	c.71G>A	p.Gly24Asp	Not present
**chr19:8603814:C>T**	ADAMTS10	1	0,000407202	0,00189394	c.506G>A	p.Arg169Gln	Not present
**chr2:98564750:G>A**	INPP4A	1	3,81047E-05	0,000184957	c.2139G>A	p.Leu713=	Not present
**chr21:33553630:A>G**	SON	1	0,001084524	0,00750144	c.4399A>G	p.Ile1467Val	Present
**chr22:19361747:G>A**	HIRA	1	0,000111635	0,000726216	c.1960C>T	p.Pro654Ser	Not present
**chr21:33552243:T>C**	SON	1	0,001070324	0,00749712	c.3012T>C	p.Ala1004=	Present
**chr11:1246353:C>A**	MUC5B	1	0,000329201	0,00376454	c.9473C>A	p.Pro3158Gln	Not present
**chr19:14151403:C>T**	ADGRL1	1	2,31697E-05	3,72702E-05	c.3880G>A	p.Ala1294Thr	Present
**chr2:99594016:A>T**	AFF3	1	0	0	c.1645T>A	p.Ser549Thr	Not present
**chr22:40270278:C>T**	TNRC6B	1	3,09969E-05	0,000483092	c.2963C>T	p.Pro988Leu	Present
**chr17:67911387:A>G**	BPTF	1	0,001368667	0,0164463	c.3503A>G	p.Asp1168Gly	Not present
**chr12:119782578:T>C**	CIT	1	0,000244567	0,00048216	c.1605A>G	p.Ala535=	Not present
**chr12:121827508:G>C**	SETD1B	1	5,8849E-05	0,000126503	c.5338-11G>C	Unknown	Present
**chr13:40559754:G>A**	FOXO1	1	0,000219129	0,00217391	c.1737C>T	p.Ser579=	Not present
**chr14:64795481:C>T**	SPTB	1	1,40836E-05	0,00024108	c.1500G>A	p.Thr500=	Not present
**chr15:101235286:C>T**	CHSY1	1	0,001177853	0,00195941	c.612G>A	p.Thr204=	Not present
**chr17:31182623:G>A**	NF1	1	0,004896398	0,0327761	c.846G>A	p.Gln282=	Present
**chr17:44260744:G>A**	SLC4A1	1	0	0	c.240C>T	p.Arg80=	Not present
**chr18:45038613:G>C**	SETBP1	1	0,009503484	0,014389	c.4129G>C	p.Val1377Leu	Present
**chr22:24322309:T>G**	SPECC1L	1	0,009321589	0,019412	c.1329T>G	p.Val443=	Not present
**chr19:42271989:G>A**	CIC	1	0,002825821	0,0244275	c.206G>A	p.Arg69Gln	Present
**chr19:45037441:C>T**	RELB	1	0	0	c.1391C>T	p.Pro464Leu	Not present
**chr19:58549189:G>A**	TRIM28	1	0,000952871	0,0147059	c.1611G>A	p.Ala537=	Not present
**chr20:41417364:A>C**	CHD6	1	0,000921305	0,00631374	c.6128-15T>G	Unknown	Not present

**chr20:47249374:C>G**	ZMYND8	1	0,009792248	0,0457535	c.1687G>C	p.Val563Leu	Present
**chr20:64208099:AGAG>A**	MYT1	1	0,002996644	0,0312774	c.917_919del	p.Glu306del	Not present
**chr21:39274427:A>G**	BRWD1	1	0,000107565	0,000723589	c.1191T>C	p.Phe397=	Not present
**chr1:10107231:C>T**	UBE4B	1	4,38222E-06	7,02938E-05	c.1206C>T	p.Asp402=	Not present
**chr1:154869723:G>GGCTGCTGCTGCTGCTGCTGCTGCT**	KCNN3	1	0,00763273	0,0189686	c.218_241dup	p.Gln73_Gln80dup	Not present
**chr1:155478624:A>G**	ASH1L	1	0,00306296	0,0353326	c.4246T>C	p.Ser1416Pro	Present
**chr11:64256673:C>T**	PLCB3	1	6,40185E-06	6,85902E-05	c.921C>T	p.Ile307=	Not present
**chr9:34241241:C>T**	UBAP1	1	4,20427E-05	0,000392773	c.216C>T	p.Ile72=	Not present
**chr9:105389035:T>G**	SLC44A1	1	3,84487E-05	0,00024108	c.1953T>G	p.Ala651=	Not present
**chr9:131198269:G>C**	NUP214	1	0,002797645	0,0300102	c.4775G>C	p.Gly1592Ala	Not present
**chr9:132896448:C>T**	TSC1	1	0,001812805	0,0131683	c.3282G>A	p.Glu1094=	Present
**chr10:77792448:G>A**	DLG5	1	0,00034694	0,00313404	c.5752C>T	p.Pro1918Ser	Not present
**chr10:102400725:G>A**	NFKB2	1	0,001039797	0,0113614	c.1869G>A	p.Gly623=	Not present
**chr11:1248395:A>G**	MUC5B	1	0,000722753	0,00231803	c.11515A>G	p.Thr3839Ala	Not present
**chr11:64265326:G>A**	PLCB3	1	0,000396225	0,0045655	c.2859G>A	p.Pro953=	Not present
**chr9:20414342:GCTA>G**	MLLT3	1	0,003112735	0,00811124	c.501_503del	p.Ser190del	Not present
**chr11:65655898:T>C**	RELA	1	1,66459E-05	3,14189E-05	c.915A>G	p.Thr305=	Not present
**chr11:66061986:G>A**	SF3B2	1	0,004921449	0,0176201	c.1965G>A	p.Ser655=	Not present
**chr12:2115387:G>A**	CACNA1C	1	0,00428748	0,0445943	c.213G>A	p.Ala71=	Present
**chr12:79292034:T>C**	SYT1	1	0,005020235	0,0301402	c.378T>C	p.Thr126=	Present
**chr12:122336719:C>G**	CLIP1	1	9,57393E-06	6,05473E-05	c.2481G>C	p.Gly827=	Not present
**chr12:122361055:C>T**	CLIP1	1	6,14616E-05	9,99709E-05	c.909G>A	p.Thr303=	Not present
**chr14:102940284:G>A**	CDC42BPB	1	7,00045E-05	0,00047259	c.4449C>T	p.Gly1483=	Present
**chr9:20414363:GCTGCTGCTGCTGCTA>G**	MLLT3	1	0,00013219	0,00144342	c.468_482del	p.Ser186_Ser190del	Not present
**chr9:3271018:G>A**	RFX3	1	0,007290127	0,0138408	c.1187C>T	p.Thr396Ile	Present
**chr1:155951747:C>A**	ARHGEF2	1	0,003694332	0,0441035	c.2202G>T	p.Pro734=	Present
**chr4:56930866:G>A**	REST	1	0,000770078	0,00384039	c.2008G>A	p.Val670Met	Not present
**chr2:15942271:G>A**	MYCN	1	0,002571833	0,00380882	c.207G>A	p.Glu69=	Not present
**chr2:86078184:C>T**	POLR1A	1	0,002229127	0,025	c.1187G>A	p.Ser396Asn	Not present
**chr2:197405293:T>C**	SF3B1	1	0,006664669	0,0381969	c.1419A>G	p.Gln473=	Present
**chr3:14481788:G>A**	SLC6A6	1	0,007212363	0,0328434	c.1669G>A	p.Val557Ile	Not present

**chr3:27721859:G>A**	EOMES	1	0,000892491	0,00633641	c.436C>T	p.Leu146Phe	Not present
**chr3:41235762:C>T**	CTNNB1	1	1,79265E-05	0,000415282	c.1722C>T	p.Thr574=	Present
**chr4:56911256:A>C**	REST	1	0,000769487	0,00389963	c.618A>C	p.Ala206=	Not present
**chr4:108165124:C>T**	LEF1	1	0,008289436	0,0167512	c.253G>A	p.Asp85Asn	Not present
**chr8:118110930:T>C**	EXT1	1	0,001912078	0,0196677	c.117A>G	p.Glu39=	Present
**chr4:125414950:A>G**	FAT4	1	0,001419123	0,00804658	c.5987A>G	p.Lys1996Arg	Not present
**chr4:125446451:G>T**	FAT4	1	0,001652876	0,00851566	c.7358G>T	p.Ser2453Ile	Not present
**chr4:125448945:C>T**	FAT4	1	0,001429875	0,00812902	c.7935C>T	p.Asp2645=	Not present
**chr4:125479767:C>T**	FAT4	1	0,002798712	0,019741	c.12506C>T	p.Thr4169Ile	Not present
**chr6:7229123:G>A**	RREB1	1	0,003140001	0,0264415	c.1024G>A	p.Asp342Asn	Not present
**chr6:7231288:C>T**	RREB1	1	0,004476297	0,0257282	c.3189C>T	p.Pro1063=	Not present
**chr6:121447605:C>T**	GJA1	1	0,009773953	0,0160844	c.758C>T	p.Ala253Val	Not present
**chr6:111307568:G>T**	REV3L	1	0,001553161	0,0176929	c.9045C>A	p.Ile3015=	Not present
**chr7:98976144:C>G**	TRRAP	1	6,56927E-06	1,46985E-05	c.7840-5C>G	Unknown	Present
**chr18:33740062:T>C**	ASXL3	1	0,000102449	0,000946074	c.2658T>C	p.Phe886=	Present
**chr5:150374302:G>A**	TCOF1	1	0,001525917	0,0109756	c.999G>A	p.Gly333=	Not present
**chr2:215376669:T>C**	FN1	1	0	0	c.5716A>G	p.Ser1906Gly	Not present
**chr2:215393163:G>A**	FN1	1	0,001905699	0,0220979	c.3837C>T	p.Thr1279=	Not present
**chr2:215430809:G>T**	FN1	1	0,000874574	0,00926868	c.591C>A	p.Val197=	Not present
**chr2:232806567:G>T**	GIGYF2	1	0,00498831	0,0417068	c.1716G>T	p.Ala572=	Present
**chr2:240740107:A>G**	KIF1A	1	0,00218159	0,0218397	c.3852T>C	p.His1284=	Present
**chr3:4673157:G>A**	ITPR1	1	0,004968949	0,00771456	c.2226G>A	p.Ala742=	Present
**chr3:38477353:G>A**	ACVR2B	1	0,001253438	0,0148638	c.119G>A	p.Arg40His	Not present
**chr3:62371211:C>T**	FEZF2	1	7,68216E-06	0,000130787	c.1120+6G>A	Unknown	Present
**chr3:197115960:A>G**	DLG1	1	0,002420128	0,0271975	c.1410T>C	p.Ser470=	Present
**chr3:197116049:T>C**	DLG1	1	0,000110822	0,000689338	c.1321A>G	p.Thr441Ala	Present
**chr4:1961055:C>T**	NSD2	1	0,000542408	0,00432028	c.3276C>T	p.Tyr1092=	Present
**chr4:39288842:T>C**	RFC1	1	0,001836165	0,0213291	c.3363A>G	p.Lys1121=	Not present
**chr4:48510068:G>A**	FRYL	1	6,57384E-06	1,47029E-05	c.8385C>T	p.Ala2795=	Present
**chr4:56931414:G>A**	REST	1	0,002615509	0,0194899	c.2556G>A	p.Lys852=	Not present

**chr4:125317707:T>C**	FAT4	1	0,000222798	0,00144648	c.1296T>C	p.Pro432=	Not present
**chr5:39202142:T>G**	FYB1	1	0,000576252	0,00707377	c.819A>C	p.Pro273=	Not present
**chr5:150129943:G>A**	PDGFRB	1	0,002207788	0,0362903	c.1393C>T	p.Leu465=	Not present
**chr2:161839775:C>A**	SLC4A10	1	0,002421894	0,0098986	c.278-14C>A	Unknown	Present
**chr2:46159645:G>A**	PRKCE	1	0,003614137	0,0404595	c.1960G>A	p.Ala654Thr	Not present
**chr2:27232076:G>A**	CAD	1	0,001565793	0,0188199	c.2497G>A	p.Asp833Asn	Not present
**chr1:68146026:G>A**	WLS	1	0,002614835	0,00574938	c.1135-14C>T	Unknown	Not present
**chr3:53178490:C>T**	PRKCD	1	0,000239733	0,00350972	c.68C>T	p.Ala23Val	Not present
**chr19:10159732:C>G**	DNMT1	1	0,00043015	0,0055386	c.1206G>C	p.Leu402=	Not present
**chr5:131698933:C>A**	FNIP1	1	3,1987E-06	3,03564E-05	c.1186G>T	p.Ala396Ser	Not present
**chr20:49018994:C>T**	ARFGEF2	1	2,05072E-05	0,000114656	c.4620C>T	p.Tyr1540=	Not present
**chr1:1790455:C>T**	GNB1	1	0	0	c.639G>A	p.Val213=	Not present
**chr1:6105431:C>A**	CHD5	1	0,003409197	0,0147588	c.*47-4G>T	Unknown	Not present
**chr1:64178539:C>T**	ROR1	1	0	0	c.2498C>T	p.Ala833Val	Not present
**chr1:119925505:G>A**	NOTCH2	1	0,002391854	0,0232474	c.4311C>T	p.Gly1437=	Not present
**chr2:27217917:T>C**	CAD	1	0,001419426	0,017318	c.123T>C	p.Thr41=	Not present
**chr1:151286943:T>C**	ZNF687	1	0,003215009	0,0304643	c.652T>C	p.Leu218=	Not present
**chr1:160834058:G>A**	CD244	1	0,002144791	0,0234198	c.953C>T	p.Ser318Phe	Not present
**chr1:175337622:G>A**	TNR	1	0,003075174	0,0330831	c.3440C>T	p.Thr1147Ile	Not present
**chr1:181758811:C>T**	CACNA1E	1	0,004633519	0,0280741	c.4548C>T	p.Ala1516=	Present
**chr1:186344065:C>T**	TPR	1	0,00336806	0,0183394	c.3443G>A	p.Arg1148His	Not present
**chr1:186346301:C>A**	TPR	1	0,005915295	0,00973587	c.2944-14G>T	Unknown	Not present
**chr1:237456642:G>A**	RYR2	1	0,004254018	0,0468266	c.1519G>A	p.Val507Ile	Not present
**chr5:150129960:C>T**	PDGFRB	1	0,002213704	0,0363112	c.1376G>A	p.Arg459His	Not present
**chr5:177209914:T>C**	NSD1	1	0,001899135	0,0217328	c.1515T>C	p.Asn505=	Present
**chr7:157253971:C>T**	UBE3C	1	0,002785693	0,0331672	c.2712C>T	p.Phe904=	Present
**chr6:90008858:T>TGAAAGAAA**	BACH2	1	0,000663877	0,0037037	c.-12-10_-12-3dup	Unknown	Not present
**chr12:52515010:C>T**	KRT5	1	0,000721682	0,00811555	c.1705G>A	p.Gly569Arg	Not present
**chr12:57202470:C>T**	LRP1	1	4,86809E-05	0,0011583	c.10644C>T	p.Cys3548=	Present
**chr12:57211530:G>A**	LRP1	1	0,000777288	0,00172911	c.13135G>A	p.Gly4379Ser	Present
**chr12:66804130:C>G**	GRIP1	1	0,004931533	0,0398601	c.56G>C	p.Gly19Ala	Present

**chr12:112235670:G>A**	HECTD4	1	0,001082118	0,0128026	c.5559C>T	p.Cys1853=	Present
**chr13:110167151:G>A**	COL4A1	1	8,96358E-06	0,000241429	c.3949+7C>T	Unknown	Not present
**chr14:67122363:T>C**	GPHN	1	0,003507118	0,0391358	c.1734T>C	p.Gly578=	Present
**chr14:88572575:T>C**	ZC3H14	1	0,004015303	0,0433067	c.432-3T>C	Unknown	Not present
**chr14:100734677:C>T**	DLK1	1	1,79289E-05	0,000130873	c.933C>T	p.Gly311=	Not present
**chr14:102870390:G>A**	TRAF3	1	0,006436191	0,037201	c.189G>A	p.Pro63=	Not present
**chr15:56098294:T>C**	RFX7	1	0,002086413	0,00351336	c.894A>G	p.Pro298=	Present
**chr16:1767197:C>T**	MAPK8IP3	1	1,7946E-05	4,82462E-05	c.3137C>T	p.Pro1046Leu	Present
**chr16:9840813:CAAAAA>C**	GRIN2A	1	0,001326663	0,00179008	c.1498-18_1498-14del	Unknown	Present
**chr16:71756153:T>C**	AP1G1	1	0	0	c.1095A>G	p.Ala365=	Not present
**chr16:72794380:T>A**	ZFHX3	1	0,000532739	0,00659502	c.8302A>T	p.Thr2768Ser	Present
**chr17:1675997:G>C**	PRPF8	1	0	0	c.2610C>G	p.Ala870=	Present
**chr17:42337874:A>AACTCCTTGACCTGAGGGAAT**	STAT3	1	0,000887163	0,0132685	c.551-37_551-18dup	Unknown	Not present
**chr12:47989761:C>T**	COL2A1	1	0,00023574	0,00271278	c.1068G>A	p.Pro356=	Not present
**chr12:8934335:G>A**	PHC1	1	0,002321561	0,00660995	c.2110G>A	p.Ala704Thr	Not present
**chr11:108185277:G>A**	NPAT	1	0,001213667	0,0143531	c.861C>T	p.Asn287=	Not present
**chr7:151071608:G>A**	SLC4A2	1	0,005426819	0,0458913	c.2191+3G>A	Unknown	Not present
**chr7:55160157:A>G**	EGFR	1	0,002500384	0,0289929	c.1317A>G	p.Ala439=	Not present
**chr7:55163733:T>C**	EGFR	1	0,002536219	0,029445	c.1632T>C	p.Gly544=	Not present
**chr7:55165345:G>A**	EGFR	1	0,002542848	0,0296744	c.1788G>A	p.Pro596=	Not present
**chr7:74338482:C>T**	CLIP2	1	0,002574364	0,0181098	c.156C>T	p.Ser52=	Present
**chr7:74338695:G>A**	CLIP2	1	0,002630381	0,018007	c.369G>A	p.Ala123=	Present
**chr7:74338803:C>T**	CLIP2	1	0,002277055	0,0145862	c.477C>T	p.His159=	Present
**chr7:151064634:G>T**	SLC4A2	1	0,000135816	0,000948767	c.326G>T	p.Arg109Leu	Not present
**chr7:155806528:G>A**	SHH	1	3,72386E-05	0,000261301	c.330C>T	p.Ala110=	Not present
**chr10:101136859:TGAC>T**	TLX1	1	0,000219041	0,00230858	c.943_945del	p.Asp315del	Not present
**chr7:158656652:C>T**	NCAPG2	1	0,001729741	0,020911	c.2114G>A	p.Ser705Asn	Not present
**chr8:60742284:T>C**	CHD7	1	1,59088E-06	0,00024108	c.852T>C	p.Ala284=	Present
**chr8:91970796:G>A**	RUNX1T1	1	0,000152554	0,0012515	c.1401C>T	p.Ala467=	Present
**chr8:107303371:C>CAAA**	ANGPT1	1	0,002585037	0,0331899	c.809-7_809-5dup	Unknown	Not present

**chr9:2039593:G>T**	SMARCA2	1	0,004390424	0,0185632	c.483G>T	p.Pro161=	Present
**chr9:106926861:G>A**	ZNF462	1	1,53655E-05	6,53851E-05	c.2949G>A	p.Ser983=	Present
**chr9:137754160:T>C**	EHMT1	1	0,000177973	0,00236295	c.1249-11T>C	Unknown	Present
**chr20:41416690:C>T**	CHD6	1	0,000476337	0,00327096	c.6384G>A	p.Pro2128=	Not present
**chr9:128689571:C>INS**	SET	1	0	0	Unknown	Unknown	Present
**chr6:13205901:G>T**	PHACTR1	1	3,84412E-05	0,000297279	c.751G>T	p.Val251Leu	Not present
**chr9:16436212:T>C**	BNC2	1	2,56141E-05	0,00048216	c.1982A>G	p.Asn661Ser	Not present
**chr12:112235243:T>A**	HECTD4	1	0,003186851	0,00535074	c.5749A>T	p.Thr1917Ser	Present
**chr12:117259124:A>T**	NOS1	1	1,59071E-06	2,28697E-05	c.2374T>A	p.Ser792Thr	Not present
**chr12:121443746:C>T**	KDM2B	1	8,35951E-05	0,000143028	c.2499G>A	p.Pro833=	Present
**chr12:121509888:G>A**	KDM2B	1	0,000322842	0,00378131	c.1326C>T	p.Gly442=	Present
**chr12:121809958:C>T**	SETD1B	1	0,000846908	0,00649726	c.1013C>T	p.Ala338Val	Present
**chr12:121814693:C>T**	SETD1B	1	0,001778764	0,0197727	c.2478C>T	p.Ser826=	Present
**chr12:121817849:C>A**	SETD1B	1	0,001483179	0,0171606	c.3363C>A	p.Asp1121Glu	Present
**chr12:121822522:A>G**	SETD1B	1	0,001912337	0,0210333	c.3943A>G	p.Met1315Val	Present
**chr12:121823562:A>G**	SETD1B	1	0,000586609	0,00708003	c.4983A>G	p.Glu1661=	Present
**chr12:131981504:C>T**	EP400	1	0,000308753	0,00121832	c.1451C>T	p.Pro484Leu	Present
**chr12:132027442:G>A**	EP400	1	0,000619739	0,0036162	c.5020G>A	p.Val1674Ile	Present
**chr13:20005113:G>A**	ZMYM2	1	0,004118374	0,0466673	c.1173G>A	p.Val391=	Present
**chr14:66776450:T>C**	GPHN	1	5,13435E-06	1,47436E-05	c.144-14T>C	Unknown	Present
**chr14:77026113:C>A**	IRF2BPL	1	0	0	c.1680G>T	p.Leu560=	Present
**chr14:77239430:T>C**	TMEM63C	1	0,004330391	0,049716	c.744T>C	p.Ser248=	Not present
**chr14:77248515:G>A**	TMEM63C	1	0,002873919	0,0331121	c.1764+6G>A	Unknown	Not present
**chr15:34791307:TCACACACA>T**	ACTC1	1	0,005647747	0,0227836	c.809-20_809-13del	Unknown	Not present
**chr12:64115808:C>G**	SRGAP1	1	6,80244E-05	0,000946074	c.2145-6C>G	Unknown	Not present
**chr12:49034911:T>C**	KMT2D	1	0,001877316	0,00275124	c.10256A>G	p.Asp3419Gly	Not present
**chr12:45836727:A>C**	ARID2	1	0,003003619	0,0315554	c.773-14A>C	Unknown	Present
**chr11:4129149:A>C**	RRM1	1	0,000786392	0,00939075	c.1768A>C	p.Lys590Gln	Not present
**chr8:94849449:C>T**	INTS8	1	0	0	c.1261-13C>T	Unknown	Not present
**chr9:81593205:G>A**	TLE1	1	0,000133149	0,000946074	c.1401C>T	p.Ile467=	Not present
**chr9:89399350:G>A**	SEMA4D	1	0,00165281	0,0196112	c.253-12C>T	Unknown	Not present

**chr10:10798823:C>T**	CELF2	1	0,002369276	0,00398589	c.53+6C>T	Unknown	Present
**chr10:29550922:G>A**	SVIL	1	4,77114E-06	0,000113033	c.502C>T	p.Pro168Ser	Not present
**chr10:60082679:G>C**	ANK3	1	7,17061E-05	0,00020578	c.4259C>G	p.Thr1420Arg	Present
**chr10:119672400:G>A**	BAG3	1	0,000252272	0,00172811	c.653G>A	p.Arg218Gln	Not present
**chr11:27658118:C>T**	BDNF	1	0,000145966	0,000588697	c.447G>A	p.Thr149=	Not present
**chr12:15623230:A>C**	EPS8	1	0,00773565	0,0297173	c.2283T>G	p.Asp761Glu	Not present
**chr11:64807573:C>T**	MEN1	1	0,000250932	0,00072324	c.762G>A	p.Leu254=	Not present
**chr11:66687088:C>T**	SPTBN2	1	7,04281E-05	0,00747348	c.6802G>A	p.Val2268Met	Not present
**chr11:78669283:A>G**	TENM4	1	0,003097763	0,0352097	c.7062T>C	p.Asp2354=	Not present
**chr11:78676266:G>A**	TENM4	1	0,003105375	0,0351205	c.5382C>T	p.Thr1794=	Not present
**chr11:118368641:A>G**	UBE4A	1	0,000473719	0,00429573	c.132A>G	p.Pro44=	Not present
**chr11:118583173:G>A**	ARCN1	1	0,004102234	0,0378981	c.268-6G>A	Unknown	Not present
**chr12:2605744:G>C**	CACNA1C	1	0,001758034	0,0183113	c.3114G>C	p.Leu1038=	Present
**chr15:63689685:T>C**	HERC1	1	0,003348223	0,0376476	c.5952A>G	p.Leu1984=	Present
**chr15:63746981:T>C**	HERC1	1	0,00345613	0,0391288	c.2457A>G	p.Ala819=	Present
**chr15:66490570:C>T**	MAP2K1	1	0,000410957	0,0037843	c.1137C>T	p.Ile379=	Not present
**chr11:74634694:A>C**	POLD3	1	0,000487046	0,00313555	c.1118A>C	p.Lys373Thr	Not present
**chr20:37396157:C>T**	SRC	1	0,004501773	0,0493946	c.554-5C>T	Unknown	Not present
**chr20:58854720:C>A**	GNAS	1	0,003491957	0,0123422	c.1268C>A	p.Pro423His	Present
**chr21:37506324:A>G**	DYRK1A	1	0,000182284	0,00197315	c.1644+101A>G	Unknown	Present
**chr22:19354025:C>T**	HIRA	1	0,000531904	0,00576037	c.2655G>A	p.Pro885=	Not present
**chr22:19396832:C>T**	HIRA	1	0,001536299	0,00995719	c.609G>A	p.Thr203=	Not present
**chr22:41527276:C>T**	ACO2	1	0,001956276	0,0178485	c.1954-12C>T	Unknown	Not present
**chr2:165377645:C>T**	SCN2A	1	0	0	c.4303C>T	p.Arg1435Ter	Present
**chr16:3728690:A>AGGCTGGGGCTGCATGCCG**	CREBBP	1	2,56195E-06	1,47042E-05	c.6339_6356dup	p.Met2115_Gly2120dup	Present
**chr20:10652243:A>G**	JAG1	1	0,000495525	0,00281167	c.894T>C	p.Asn298=	Not present
**chr9:128693807:T>DEL**	SET	1	0	0	Unknown	Unknown	Present
**chr16:8905280:T>C**	USP7	1	2,22653E-05	0,000128944	c.1480A>G	p.Ile494Val	Present
**chr5:128336069:T>C**	FBN2	1	0	0	c.3643A>G	p.Lys1215Glu	Not present
**chr9:138058633:A>C**	CACNA1B	1	0,000140872	0,000810296	c.4373A>C	p.Gln1458Pro	Present
**chr8:41684576:C>T**	ANK1	1	3,84336E-05	0,000477555	c.4505G>A	p.Arg1502His	Not present

**chr5:153811057:C>T**	GRIA1	1	2,17675E-05	0,000274348	c.2553C>T	p.Asn851=	Present
**chr2:165295923:G>A**	SCN2A	1	0,000665827	0,00432277	c.100G>A	p.Ala34Thr	Present
**chr20:13435081:G>A**	TASP1	1	0,000461468	0,00193611	c.1059C>T	p.Ala353=	Not present
**chr20:10644993:G>A**	JAG1	1	2,56131E-06	6,54879E-05	c.2228-14C>T	Unknown	Not present
**chr15:69421729:A>G**	KIF23	1	0,003815924	0,0293948	c.293A>G	p.Asn98Ser	Not present
**chr17:44862920:C>A**	EFTUD2	1	0,004163187	0,02561	c.1414-14G>T	Unknown	Not present
**chr15:69422008:T>C**	KIF23	1	0,004789027	0,0437069	c.333T>C	p.Tyr111=	Not present
**chr15:69446265:A>G**	KIF23	1	0,003778499	0,0357345	c.2757-18A>G	Unknown	Not present
**chr16:2109315:C>T**	PKD1	1	0,000478699	0,00463689	c.5852G>A	p.Arg1951Gln	Present
**chr16:3793453:C>T**	CREBBP	1	0,001872045	0,0200648	c.1149G>A	p.Pro383=	Present
**chr16:72950556:G>A**	ZFHX3	1	6,2739E-05	0,000478469	c.3129C>T	p.Asn1043=	Present
**chr17:8888641:G>T**	PIK3R5	1	0,000887927	0,00136037	c.1146C>A	p.Gly382=	Not present
**chr17:44255219:A>G**	SLC4A1	1	0,004001416	0,0425701	c.1878T>C	p.Asp626=	Not present
**chr17:50191443:G>A**	COL1A1	1	0,003159094	0,0348988	c.2175C>T	p.Gly725=	Not present
**chr19:54129298:A>G**	PRPF31	1	0,004197149	0,0468991	c.1302A>G	p.Val434=	Not present
**chr17:50618996:G>A**	CACNA1G	1	7,17354E-06	0,0002936	c.5769G>A	p.Leu1923=	Present
**chr17:55315435:T>C**	HLF	1	0,003667056	0,0432176	c.660T>C	p.Pro220=	Not present
**chr18:33734389:A>G**	ASXL3	1	0,000480539	0,00293542	c.1056A>G	p.Lys352=	Present
**chr18:33746194:G>A**	ASXL3	1	0,002277537	0,00360331	c.6346G>A	p.Ala2116Thr	Present
**chr19:15238407:T>C**	BRD4	1	3,71311E-05	0,000452131	c.4059A>G	p.Leu1353=	Present
**chr19:18145157:C>T**	MAST3	1	0,004225229	0,0473042	c.2967C>T	p.Tyr989=	Present
**chr19:54129140:A>G**	PRPF31	1	0,004656867	0,0469555	c.1230A>G	p.Thr410=	Not present
**chr12:78200543:C>T**	NAV3	1	0,001599894	0,0291066	c.6786C>T	p.Asn2262=	Present
**chr12:32730998:C>T**	DNM1L	1	0,006978824	0,0143062	c.1080-16C>T	Unknown	Not present
**chr12:6577854:A>G**	CHD4	1	0,005871117	0,0138249	c.5292T>C	p.Pro1764=	Present
**chr2:25743994:T>A**	ASXL2	1	0,001281927	0,00419757	c.2343A>T	p.Pro781=	Not present
**chr1:209790561:C>T**	IRF6	1	0,000259885	0,00269151	c.994G>A	p.Val332Ile	Not present
**chr12:132066948:G>A**	EP400	1	0,000126119	0,000477555	c.8728G>A	p.Gly2910Ser	Present
**chr4:113358297:A>C**	ANK2	1	0,000119189	0,00022017	c.9679A>C	p.Thr3227Pro	Present

**chr9:134812672:C>T**	COL5A1	1	4,20934E-05	0,000154169	c.3812C>T	p.Pro1271Leu	Not present
**chr19:13207857:G>GCCT**	CACNA1A	1	0,000572458	0,00313339	c.6974_6976dup	p.Gln2325_Ala2326insGlu	Present
**chr11:47422672:T>C**	PSMC3	1	1,59039E-06	2,85629E-06	c.786A>G	p.Gly262=	Not present
**chr16:72797249:G>A**	ZFHX3	1	0,000137109	0,000242945	c.5433C>T	p.Pro1811=	Present
**chr10:8058543:C>G**	GATA3	1	0,000290046	0,00289296	c.480C>G	p.Asp160Glu	Not present
**chr1:3425743:C>T**	PRDM16	1	0,001936147	0,00662314	c.3102C>T	p.Asn1034=	Not present
**chr1:7663346:TC>T**	CAMTA1	1	0,009561922	0,0265439	c.806-5del	Unknown	Not present
**chr1:22910504:A>G**	EPHB2	1	0,006672692	0,0109667	c.2625A>G	p.Gln875=	Present
**chr1:27105963:C>T**	SLC9A1	1	7,30189E-05	0,000392054	c.1407G>A	p.Leu469=	Present
**chr1:39385472:T>C**	MACF1	1	4,77131E-06	8,56908E-06	c.13887T>C	p.His4629=	Present
**chr1:43545149:C>T**	PTPRF	1	0	0	c.74C>T	p.Ala25Val	Not present
**chr1:43591099:G>A**	PTPRF	1	3,97071E-05	0,000221791	c.1077G>A	p.Thr359=	Not present
**chr1:160419034:A>G**	VANGL2	1	2,18849E-05	0,000130993	c.225A>G	p.Val75=	Not present
**chr1:181511357:C>T**	CACNA1E	1	0,002789818	0,0325042	c.373-14C>T	Unknown	Present
**chr21:33552244:G>T**	SON	1	0,000161347	0,000576369	c.3013G>T	p.Ala1005Ser	Present
**chr12:112269801:T>G**	HECTD4	1	7,81118E-05	0,00216972	c.2224A>C	p.Ile742Leu	Present
**chr14:99874916:G>T**	EML1	1	5,43668E-05	0,000102923	c.384-15G>T	Unknown	Not present
**chr2:60461949:C>T**	BCL11A	1	0	0	c.963G>A	p.Glu321=	Present
**chr20:32798526:G>A**	DNMT3B	1	0,001357036	0,0127891	c.1557G>A	p.Gln519=	Not present
**chr20:47349878:C>T**	ZMYND8	1	1,42387E-05	0,000196412	c.12G>A	p.Ser4=	Present
**chr20:63493299:G>A**	EEF1A2	1	4,48173E-06	1,47067E-05	c.622-12C>T	Unknown	Present
**chr20:64208081:GGAGGAA>G**	MYT1	1	0,002288634	0,0102731	c.903_908del	p.Glu305_Glu306del	Not present
**chr22:36926059:C>T**	CSF2RB	1	0,008471256	0,0192626	c.273C>T	p.Cys91=	Not present
**chr22:27799076:C>T**	MN1	1	0	0	c.1468G>A	p.Ala490Thr	Not present
**chr12:45850744:C>A**	ARID2	1	0	0	c.2621C>A	p.Thr874Lys	Present
**chr12:14790529:A>G**	WBP11	1	0	0	c.1236T>C	p.Pro412=	Not present
**chr12:119701851:C>A**	CIT	1	1,02438E-05	1,99942E-05	c.5412G>T	p.Glu1804Asp	Not present
**chr6:13184946:T>A**	PHACTR1	1	5,24703E-06	1,04207E-05	c.812T>A	p.Leu271His	Not present
**chr7:44624459:G>A**	OGDH	1	5,12538E-06	0,000209205	c.116G>A	p.Cys39Tyr	Not present
**chr5:161326400:G>A**	GABRB2	1	9,0985E-05	0,000482393	c.1159C>T	p.Arg387Trp	Present
**chr5:128289136:G>A**	FBN2	1	4,09789E-05	9,40982E-05	c.6628C>T	p.Arg2210Cys	Not present
**chr1:186362850:T>G**	TPR	1	0	0	c.683A>C	p.Asn228Thr	Not present

**chr15:33857817:A>C**	RYR3	1	1,59051E-06	2,85664E-06	c.14045A>C	p.Tyr4682Ser	Not present
**chr1:160292478:C>A**	COPA	1	3,58988E-05	0,000229535	c.2960+6G>T	Unknown	Not present
**chr1:244858744:C>T**	HNRNPU	1	0,002067886	0,0121107	c.1215G>A	p.Val405=	Present
**chr2:27233482:C>T**	CAD	1	0,001060014	0,00310734	c.3162C>T	p.Leu1054=	Not present
**chr12:867856:G>A**	WNK1	1	0	0	c.2140-10G>A	Unknown	Not present
**chr2:50538274:G>T**	NRXN1	1	0,004425437	0,00699804	c.2122C>A	p.Leu708Ile	Present
**chr8:76863212:ACCT>A**	ZFHX4	1	0,000851048	0,00861057	c.9517_9519del	p.Pro3173del	Present
**chr9:20414345:ACTGCTGCTGCTGCTG>A**	MLLT3	1	6,78997E-06	2,57666E-05	c.486_500del	p.Ser186_Ser190del	Not present
**chr9:128625846:C>T**	SPTAN1	1	1,92075E-05	9,42685E-05	c.6147C>T	p.Leu2049=	Not present
**chr9:134817038:C>T**	COL5A1	1	0,007963863	0,030888	c.4135C>T	p.Pro1379Ser	Not present
**chr9:138046931:G>T**	CACNA1B	1	0,001228319	0,0095462	c.3441G>T	p.Val1147=	Present
**chr11:31801889:T>C**	PAX6	1	0	0	c.165A>G	p.Gln55=	Present
**chr11:96092210:T>TTGC**	MAML2	1	0,000437131	0,00130626	c.1818_1820dup	p.Gln621dup	Not present
**chr12:41020410:T>C**	CNTN1	1	0,006643014	0,044406	c.2493T>C	p.His831=	Not present
**chr12:98594096:C>T**	SLC25A3	1	0	0	c.118C>T	p.Pro40Ser	Not present
**chr12:117286213:T>G**	NOS1	1	0,005478088	0,0147059	c.1181A>C	p.Asp394Ala	Not present
**chr14:102975681:T>C**	CDC42BPB	1	0,000217678	0,00148002	c.1507+3A>G	Unknown	Present
**chr15:88845851:C>T**	ACAN	1	0,004609382	0,0165906	c.1398C>T	p.Thr466=	Not present
**chr16:24791553:C>G**	TNRC6A	1	0,001057809	0,00431598	c.2911C>G	p.Leu971Val	Not present
**chr17:6464718:G>A**	PITPNM3	1	0,000241978	0,00231717	c.1944C>T	p.Pro648=	Not present
**chr17:39792890:C>T**	IKZF3	1	7,68409E-06	9,06618E-05	c.207G>A	p.Glu69=	Not present
**chr17:45478097:G>A**	PLEKHM1	1	7,68285E-05	0,000206993	c.99C>T	p.Tyr33=	Not present
**chr19:13138142:C>T**	NACC1	1	6,37387E-06	1,14553E-05	c.1325-5C>T	Unknown	Present
**chr8:69586397:C>T**	SULF1	1	8,67521E-05	0,00314313	c.453C>T	p.Tyr151=	Not present
**chr8:52645747:A>T**	RB1CC1	1	0,007507303	0,0102935	c.3942T>A	p.Asn1314Lys	Not present
**chr7:135593180:C>T**	NUP205	1	0,003849138	0,0458047	c.1818C>T	p.Thr606=	Not present
**chr4:125415182:A>G**	FAT4	1	0,005823378	0,00922787	c.6219A>G	p.Gln2073=	Not present
**chr2:189068227:G>A**	COL5A2	1	0,001729807	0,0286885	c.1301C>T	p.Thr434Met	Not present
**chr2:189110286:C>T**	COL5A2	1	0,001595378	0,0286885	c.261G>A	p.Thr87=	Not present
**chr2:197398544:C>T**	SF3B1	1	3,18206E-06	5,65803E-05	c.3051G>A	p.Leu1017=	Present

**chr3:39088149:G>A**	WDR48	1	7,68393E-06	0,00024108	c.1496G>A	p.Arg499Gln	Not present
**chr3:105737189:G>A**	CBLB	1	0,00170302	0,0200751	c.1053C>T	p.Asp351=	Not present
**chr3:129574473:C>T**	PLXND1	1	0,001154044	0,00858634	c.2548G>A	p.Ala850Thr	Not present
**chr3:169143748:C>A**	MECOM	1	0,004206942	0,0217602	c.460G>T	p.Ala154Ser	Not present
**chr5:112843456:C>G**	APC	1	0,003556272	0,00551308	c.7862C>G	p.Ser2621Cys	Not present
**chr7:83155272:C>T**	PCLO	1	0,004210356	0,0195853	c.1369G>A	p.Gly457Arg	Present
**chr5:168748403:C>T**	SLIT3	1	0,000384292	0,0030545	c.2169G>A	p.Pro723=	Not present
**chr5:168806545:G>C**	SLIT3	1	0,000195871	0,0012054	c.836C>G	p.Ser279Cys	Not present
**chr6:12749741:C>T**	PHACTR1	1	0,001569024	0,00567537	c.201C>T	p.Ser67=	Not present
**chr6:56900580:T>C**	DST	1	0,00147779	0,00282398	c.258A>G	p.Ala86=	Present
**chr6:165711136:T>C**	PDE10A	1	0,003998897	0,00623364	c.76A>G	p.Met26Val	Not present
**chr7:5527853:G>A**	ACTB	1	0,003084143	0,0366906	c.1023C>T	p.Ile341=	Present
**chr7:77610785:T>C**	PTPN12	1	0,002187579	0,00517539	c.783T>C	p.Asn261=	Not present
**chr19:18854868:C>T**	UPF1	1	2,17743E-05	0,000072317	c.1266-11C>T	Unknown	Not present
**chr19:16417645:T>C**	EPS15L1	1	0,001565045	0,00867888	c.1108-8A>G	Unknown	Not present
**chr19:8605230:C>T**	ADAMTS10	1	0,004485816	0,00732052	c.217G>A	p.Glu73Lys	Not present
**chr17:45475112:C>T**	PLEKHM1	1	0,000427747	0,00580431	c.911G>A	p.Arg304Gln	Not present
**chr15:63696158:C>INS**	HERC1	1	0	0	Unknown	Unknown	Present
**chr17:31940362:A>INS**	SUZ12	1	0	0	Unknown	Unknown	Not present
**chr7:55019302:G>A**	EGFR	1	1,8788E-06	3,34979E-06	c.25G>A	p.Ala9Thr	Not present
**chr2:108775923:T>A**	RANBP2	1	0,000162158	0,000478011	c.8484T>A	p.Asp2828Glu	Not present
**chr5:83572449:A>INS**	VCAN	1	0	0	Unknown	Unknown	Not present
**chr8:104013512:C>T**	RIMS2	1	2,43381E-05	0,000113109	c.3340C>T	p.Arg1114Trp	Present
**chr12:110282633:A>INS**	ATP2A2	1	0	0	Unknown	Unknown	Not present
**chr6:69062925:C>INS**	ADGRB3	1	0	0	Unknown	Unknown	Not present
**chr9:92718656:G>A**	BICD2	1	5,25113E-05	0,000385356	c.1989C>T	p.Pro663=	Not present
**chr12:117242635:A>INS**	NOS1	1	0	0	Unknown	Unknown	Not present
**chr4:39457644:A>INS**	RPL9	1	0	0	Unknown	Unknown	Not present
**chr12:111555933:A>INS**	ATXN2	1	0	0	Unknown	Unknown	Present
**chr1:21238227:C>G**	ECE1	1	0	0	c.1296G>C	p.Trp432Cys	Not present
**chr1:43364659:G>A**	ELOVL1	1	5,12215E-06	1,47016E-05	c.376-12C>T	Unknown	Not present
**chr1:160841879:A>G**	CD244	1	0,003133355	0,011222	c.84T>C	p.His28=	Not present
**chr1:183125421:C>T**	LAMC1	1	0,000170315	0,00650916	c.2672C>T	p.Thr891Ile	Not present
**chr2:69152195:C>T**	ANTXR1	1	0,000183087	0,00265188	c.978C>T	p.Ala326=	Not present

**chr10:63186247:T>G**	JMJD1C	1	5,52144E-05	0,000483793	c.6707A>C	p.Lys2236Thr	Present
**chr1:102997083:A>INS**	COL11A1	1	0	0	Unknown	Unknown	Not present
**chr4:103181365:A>INS**	CENPE	1	0	0	Unknown	Unknown	Not present
**chr2:108751611:A>INS**	RANBP2	1	0	0	Unknown	Unknown	Not present
**chr12:57581136:A>G**	KIF5A	1	0	0	c.2719A>G	p.Lys907Glu	Not present
**chr7:148828741:A>INS**	EZH2	1	0	0	Unknown	Unknown	Not present
**chr15:38351192:A>C**	SPRED1	1	1,59054E-06	2,85642E-06	c.863A>C	p.Lys288Thr	Not present
**chr2:224495902:A>INS**	CUL3	1	0	0	Unknown	Unknown	Present
**chr3:136568792:A>INS**	STAG1	1	0	0	Unknown	Unknown	Present
**chr11:66193530:T>C**	PACS1	1	3,2017E-05	5,87907E-05	c.401T>C	p.Met134Thr	Present
**chr17:42483090:A>INS**	ATP6V0A1	1	0	0	Unknown	Unknown	Not present
**chr18:2666190:A>G**	SMCHD1	1	3,90382E-06	6,54707E-05	c.220A>G	p.Ile74Val	Not present
**chr17:29477710:A>INS**	TAOK1	1	0	0	Unknown	Unknown	Present
**chr10:11321320:G>A**	CELF2	1	2,95054E-05	0,000160037	c.1228G>A	p.Ala410Thr	Present
**chr10:71820243:A>INS**	PSAP	1	0	0	Unknown	Unknown	Not present
**chr14:67317412:A>INS**	PALS1, GPHN	1	0	0	Unknown	Unknown	Not present
**chr11:74640601:T>G**	POLD3	1	9,75029E-06	2,42907E-05	c.1236T>G	p.Asp412Glu	Not present
**chr6:111313373:A>INS**	REV3L	1	0	0	Unknown	Unknown	Not present
**chr6:7246473:C>A**	RREB1	1	8,52687E-06	3,32182E-05	c.4023C>A	p.Asp1341Glu	Not present
**chr4:84690520:C>T**	WDFY3	1	1,02436E-05	0,000386548	c.9349G>A	p.Val3117Ile	Present
**chr2:86070277:G>A**	POLR1A	1	0,001084085	0,00621118	c.1612-5C>T	Unknown	Not present
**chr2:169212200:G>A**	LRP2	1	0,002044068	0,0153304	c.6048C>T	p.Ala2016=	Present
**chr2:215386815:G>A**	FN1	1	0,00544688	0,00845912	c.4486C>T	p.Arg1496Trp	Not present
**chr12:122341310:C>T**	CLIP1	1	6,14858E-05	0,000434385	c.1894G>A	p.Ala632Thr	Not present
**chr9:137975914:A>G**	CACNA1B	1	0,00032805	0,000566073	c.1551A>G	p.Ala517=	Present
**chr10:75028901:GGAAGAGGAA>G**	KAT6B	1	0,008770759	0,012536	c.4097_4105del	p.Glu1366_Glu1368del	Present
**chr10:77843632:G>A**	DLG5	1	7,94147E-05	0,000131303	c.939C>T	p.Val313=	Not present
**chr11:72014588:C>T**	NUMA1	1	0,008750825	0,0135254	c.2915G>A	p.Arg972Gln	Not present
**chr12:52950324:C>G**	KRT18	1	0,001240466	0,00328767	c.418-4C>G	Unknown	Not present
**chr12:57206498:C>T**	LRP1	1	0,006914089	0,0108139	c.11616C>T	p.Ile3872=	Present
**chr12:112382087:GGCC>G**	HECTD4	1	0,00183291	0,00430395	c.39_41del	p.Ala15del	Present
**chr12:132011621:A>G**	EP400	1	0,002547366	0,00409671	c.3428A>G	p.Lys1143Arg	Present
**chr9:126693840:G>A**	LMX1B	1	0,004678153	0,0258014	c.914G>A	p.Gly305Asp	Present
**chr15:40982948:C>A**	INO80	1	0,000154935	0,000759334	c.4367G>T	p.Ser1456Ile	Not present
**chr15:88847896:C>G**	ACAN	1	0	0	c.1605-15C>G	Unknown	Not present
**chr16:2099781:G>A**	PKD1	1	0,001377072	0,00210193	c.9924-11C>T	Unknown	Present

**chr16:69670219:C>INS**	NFAT5	1	0	0	Unknown	Unknown	Not present
**chr16:89279945:G>C**	ANKRD11	1	0,000253599	0,000470898	c.6597C>G	p.Leu2199=	Present
**chr17:17803779:C>G**	RAI1	1	4,74234E-05	0,00041425	c.5589C>G	p.Ala1863=	Present
**chr17:43000031:G>A**	RPL27	1	0,003008171	0,0060471	c.180G>A	p.Lys60=	Not present
**chr9:130885206:G>A**	ABL1	1	0,003109617	0,0108536	c.2916G>A	p.Ser972=	Not present
**chr9:125302818:C>T**	GAPVD1	1	0,000504154	0,00442796	c.1021C>T	p.Leu341=	Not present
**chr3:9013751:G>A**	SRGAP3	1	0,001606733	0,0037843	c.1905C>T	p.Phe635=	Present
**chr5:14183969:T>INS**	TRIO	1	0	0	Unknown	Unknown	Present
**chr4:53458645:A>C**	FIP1L1	1	0,004613321	0,0109573	c.1500-8A>C	Unknown	Not present
**chr4:54274918:G>A**	PDGFRA	1	0,003244384	0,0231884	c.1731G>A	p.Pro577=	Not present
**chr4:71534257:C>T**	SLC4A4	1	0,001670608	0,0100865	c.2311C>T	p.Pro771Ser	Not present
**chr4:87114964:G>A**	AFF1	1	0,007258656	0,03861	c.2131G>A	p.Val711Ile	Not present
**chr4:125319813:A>T**	FAT4	1	0,001702731	0,0144009	c.3402A>T	p.Glu1134Asp	Not present
**chr4:125491756:A>G**	FAT4	1	0,001203168	0,00315344	c.14940A>G	p.Glu4980=	Not present
**chr4:152329708:G>A**	FBXW7	1	0,001388386	0,0085027	c.1200C>T	p.Asp400=	Not present
**chr5:39108220:A>INS**	FYB1	1	0	0	Unknown	Unknown	Not present
**chr9:81585518:T>C**	TLE1	1	0,000169149	0,00101678	c.2115A>G	p.Lys705=	Not present
**chr5:56888283:A>G**	MAP3K1	1	1,92086E-05	6,85997E-05	c.4315A>G	p.Ile1439Val	Not present
**chr5:87395042:A>INS**	RASA1, CCNH	1	0	0	Unknown	Unknown	Not present
**chr5:112839543:G>C**	APC	1	0,005122237	0,0139826	c.3949G>C	p.Glu1317Gln	Not present
**chr5:112840859:G>A**	APC	1	0,005420044	0,0144648	c.5265G>A	p.Ala1755=	Not present
**chr6:34418381:G>A**	RPS10	1	0,000112674	0,00118684	c.444C>T	p.Thr148=	Not present
**chr6:56618881:T>G**	DST	1	0,002776988	0,00389599	c.5153A>C	p.Lys1718Thr	Present
**chr9:27185596:A>T**	TEK	1	5,63754E-05	0,00094697	c.1294A>T	p.Met432Leu	Present
**chr22:36319565:G>A**	MYH9	1	0,004321139	0,031682	c.1083C>T	p.Asp361=	Present
**chr16:28866626:C>T**	SH2B1	1	1,02496E-05	0,000193349	c.532C>T	p.Pro178Ser	Not present
**chr16:30964202:T>C**	SETD1A	1	1,59037E-06	2,85626E-06	c.748T>C	p.Ser250Pro	Present
**chr19:17652660:CTCTCCT>C**	UNC13A	1	1,53733E-05	2,93988E-05	c.1404_1409del	p.Gly469_Glu470del	Present
**chr6:16327687:C>CTGCTGA**	ATXN1	1	0,000171609	0,00124069	c.623_624insTCAGCA	p.Gln207_Gln208insHisGln	Not present
**chr15:26547877:A>G**	GABRB3	1	6,27338E-05	0,000161703	c.1338T>C	p.Asn446=	Present

**chr6:56629343:T>C**	DST	1	6,40574E-06	0,000014705	c.4382A>G	p.Tyr1461Cys	Present
**chr4:39308660:T>C**	RFC1	1	3,1929E-06	5,73529E-06	c.1861A>G	p.Ser621Gly	Not present
**chr17:62010657:G>A**	MED13	1	0,00044952	0,000838161	c.1860C>T	p.Phe620=	Present
**chr15:43059113:T>G**	UBR1	1	0,000161321	0,000441034	c.1065A>C	p.Leu355Phe	Present
**chr15:63725336:G>A**	HERC1	1	2,3057E-05	0,000113033	c.3524C>T	p.Thr1175Met	Present
**chr15:77615268:G>A**	LINGO1	1	6,14779E-05	0,000621633	c.639C>T	p.His213=	Not present
**chr12:77831465:C>A**	NAV3	1	0,000297085	0,00150543	c.4C>A	p.Pro2Thr	Present
**chr4:84733466:C>T**	WDFY3	1	0,000152391	0,00189753	c.7137G>A	p.Arg2379=	Present
**chr1:3412384:C>G**	PRDM16	1	0,000477993	0,00150517	c.2187C>G	p.Phe729Leu	Not present
**chr5:87376917:T>G**	RASA1	1	7,68413E-06	0,000288018	c.2221T>G	p.Leu741Val	Not present
**chr5:170253133:G>A**	LCP2	1	1,02822E-05	4,82859E-05	c.1231C>T	p.Pro411Ser	Not present
**chr2:169202833:C>T**	LRP2	1	0,000215083	0,00891996	c.8132G>A	p.Arg2711His	Present
**chr1:11022387:C>G**	TARDBP	1	1,59093E-06	2,85765E-06	c.978C>G	p.Ala326=	Not present
**chr1:30950129:T>C**	PUM1	1	0,002005365	0,00468682	c.2854A>G	p.Ile952Val	Not present
**chr1:39322623:C>A**	MACF1	1	0,001206127	0,00331439	c.4045C>A	p.Leu1349Met	Present
**chr1:8361136:G>A**	RERE	1	0,000133687	0,0014218	c.2371C>T	p.Pro791Ser	Present
**chr17:7906929:A>G**	CHD3	1	0,00034572	0,00276401	c.4564A>G	p.Ser1522Gly	Present
**chr6:7230707:C>T**	RREB1	1	1,83602E-05	5,96036E-05	c.2608C>T	p.Leu870Phe	Not present
**chr22:36304126:C>G**	MYH9	1	0	0	c.2259G>C	p.Leu753=	Present
**chr19:31277168:G>A**	TSHZ3	1	2,68976E-05	0,000114322	c.2625C>T	p.Asp875=	Present
**chr19:31277843:G>A**	TSHZ3	1	3,99092E-05	0,000386518	c.1950C>T	p.Val650=	Present
**chr19:34452150:T>G**	UBA2	1	1,47742E-05	0,000249252	c.1038+3T>G	Unknown	Not present
**chr19:54148530:C>T**	CNOT3	1	0,004173982	0,0394813	c.1277C>T	p.Ala426Val	Present
**chr20:8774575:G>A**	PLCB1	1	0,00685876	0,0262904	c.2967G>A	p.Thr989=	Present
**chr20:51694014:C>T**	ATP9A	1	3,07744E-05	9,07606E-05	c.636G>A	p.Thr212=	Present
**chr21:14966122:G>A**	NRIP1	1	0,001963008	0,0032056	c.2071C>T	p.Pro691Ser	Not present
**chr12:45850450:A>G**	ARID2	1	0	0	c.2327A>G	p.His776Arg	Present
**chr19:13209377:C>T**	CACNA1A	1	2,33717E-05	0,000206782	c.6461G>A	p.Arg2154Gln	Present
**chr9:128566850:G>A**	SPTAN1	1	3,18083E-06	3,02133E-05	c.110G>A	p.Arg37His	Not present
**chr9:106927601:C>T**	ZNF462	1	1,59051E-06	2,85639E-06	c.3689C>T	p.Thr1230Met	Present
**chr17:67959799:A>T**	BPTF	1	5,1222E-06	3,02133E-05	c.8185A>T	p.Ile2729Phe	Not present

**chr21:33552629:C>G**	SON	1	6,40315E-06	1,47007E-05	c.3398C>G	p.Thr1133Ser	Present
**chr17:32464051:C>T**	PSMD11	1	5,12295E-06	0,00028818 4	c.321C>T	p.Val107=	Present
**chr8:101632228:T>A**	GRHL2	1	5,89142E-05	9,71479E-05	c.1348T>A	p.Ser450Thr	Not present
**chr3:49661726:A>G**	BSN	1	9,74127E-05	0,00047892 7	c.9881A>G	p.Asp3294Gly	Present
**chr1:81970306:C>T**	ADGRL2	1	3,87203E-06	6,58935E-05	c.2734-8C>T	Unknown	Not present
**chr1:94205663:C>G**	ARHGAP29	1	4,78303E-06	8,58487E-06	c.531G>C	p.Val177=	Not present
**chr1:109265801:T>G**	CELSR2	1	0,00243164 8	0,00602991	c.5794T>G	p.Leu1932Val	Not present
**chr9:131228332:G>GTAAGCCCCCTGGGGAGGGCCCTT**	NUP214	1	0,00607789 1	0,036266	c.6074+2_6074+24dup	Unknown	Not present
**chr7:116740941:C>G**	MET	1	0	0	c.1617C>G	p.Gly539=	Present
**chr7:135601362:C>G**	NUP205	1	6,57471E-06	2,41499E-05	c.2375-8C>G	Unknown	Not present
**chr8:41932226:G>A**	KAT6A	1	0,00080180 8	0,00252866	c.5994C>T	p.Asn1998=	Present
**chr8:102311413:A>G**	UBR5	1	5,63421E-05	0,00086405 5	c.2502T>C	p.Asp834=	Present
**chr9:35547811:ACCAGGCCCTGGC>A**	RUSC2	1	0,00102699 6	0,00795564	c.1295_1306del	p.Gly432_Pro435del	Not present
**chr9:124858014:G>A**	RPL35	1	0,00036957 7	0,00162044	c.276C>T	p.Arg92=	Not present
**chr9:128257207:T>C**	GOLGA2	1	0,00301435 5	0,0332429	c.2950A>G	p.Asn984Asp	Not present
**chr9:136496869:G>A**	NOTCH1	1	0,00289053 7	0,00700882	c.6870C>T	p.Ser2290=	Present
**chr6:170283021:G>A**	DLL1	1	0,00430620 5	0,0084378	c.2133C>T	p.Ser711=	Present
**chr10:63168077:C>T**	JMJD1C	1	0,00485732	0,00822131	c.7591G>A	p.Glu2531Lys	Present
**chr10:79311067:G>A**	ZMIZ1	1	0,00141607 8	0,00425733	c.2979G>A	p.Pro993=	Present
**chr10:103613166:C>T**	SH3PXD2A	1	0,00759703 2	0,0151351	c.945G>A	p.Ala315=	Not present
**chr10:103678147:C>G**	SH3PXD2A	1	0	0	c.49G>C	p.Gly17Arg	Not present
**chr10:121565644:G>A**	FGFR2	1	0,00457582 1	0,0217921	c.170C>T	p.Ser57Leu	Not present
**chr11:17772627:C>T**	KCNC1	1	0,00028143 6	0,00346722	c.1533C>T	p.Ile511=	Present
**chr11:45934201:T>C**	PHF21A	1	0,00163865 1	0,00282262	c.1813A>G	p.Ile605Val	Present
**chr7:84134924:G>T**	SEMA3A	1	1,40967E-05	0,00020712 5	c.140C>A	p.Thr47Asn	Not present
**chr6:87259603:T>C**	ZNF292	1	0,00078359 8	0,00268162	c.5974T>C	p.Ser1992Pro	Present
**chr1:110341408:C>T**	RBM15	1	0,00671007 8	0,0202507	c.2003C>T	p.Ala668Val	Not present
**chr4:3074969:T>G**	HTT	1	0,00104331 7	0,00331762	c.144T>G	p.Pro48=	Not present
**chr1:151814975:C>T**	RORC	1	0,00377928 6	0,00771343	c.749G>A	p.Ser250Asn	Not present
**chr1:183127303:G>A**	LAMC1	1	8,96296E-06	4,40878E-05	c.3022G>A	p.Val1008Met	Not present

**chr1:244054113:C>T**	ZBTB18	1	0,002914422	0,00915392	c.339C>T	p.Leu113=	Present
**chr2:211712052:A>C**	ERBB4	1	0,002528367	0,0249642	c.1122T>G	p.His374Gln	Not present
**chr2:239095043:G>A**	HDAC4	1	0,000257454	0,00604839	c.2247C>T	p.Ser749=	Present
**chr3:122261728:G>A**	CASR	1	6,56901E-06	0,000014699	c.693G>A	p.Glu231=	Not present
**chr3:174279309:T>C**	NLGN1	1	0,001933792	0,00307638	c.1368T>C	p.Ile456=	Present
**chr4:76741208:C>A**	SHROOM3	1	0,007690161	0,0170815	c.3035C>A	p.Thr1012Asn	Not present
**chr6:32017141:C>A**	C4B	1	0	0	c.1040C>A	p.Ser347Tyr	Present
**chr5:10411523:A>C**	MARCHF6	1	0,005882489	0,0161078	c.1882A>C	p.Asn628His	Not present
**chr5:72198257:C>T**	MAP1B	1	0,00441951	0,00635233	c.4902C>T	p.Pro1634=	Present
**chr5:112842515:G>A**	APC	1	0,001548364	0,00235329	c.6921G>A	p.Ser2307=	Not present
**chr5:138391480:G>T**	KDM3B	1	0,003591531	0,0214926	c.1848G>T	p.Glu616Asp	Present
**chr5:138418969:C>G**	KDM3B	1	0,001528692	0,00995997	c.3452C>G	p.Pro1151Arg	Present
**chr5:151065073:C>T**	TNIP1	1	1,66506E-05	0,00003142	c.23G>A	p.Arg8Gln	Not present
**chr5:168687070:T>C**	SLIT3	1	5,12139E-06	1,46951E-05	c.3223A>G	p.Asn1075Asp	Not present
**chr19:18857376:G>A**	UPF1	1	0,000185851	0,000338027	c.2025G>A	p.Ala675=	Not present
**chr19:14170770:G>A**	ADGRL1	1	0,001431522	0,00456432	c.306C>T	p.Cys102=	Present
**chr12:31479919:T>C**	DENND5B	1	3,18199E-06	3,02334E-05	c.574A>G	p.Ile192Val	Not present
**chr18:48950310:C>T**	SMAD7	1	0,008011482	0,0122351	c.115G>A	p.Gly39Arg	Not present
**chr2:108755041:C>T**	RANBP2	1	0,007778568	0,0360413	c.2339C>T	p.Pro780Leu	Not present
**chr2:108763494:G>A**	RANBP2	1	0,007757715	0,0359876	c.2955G>A	p.Pro985=	Not present
**chr2:108768392:A>G**	RANBP2	1	0,006876441	0,0322427	c.7849+4A>G	Unknown	Not present
**chr2:120968790:C>T**	GLI2	1	0,006635247	0,0233478	c.720C>T	p.Asp240=	Not present
**chr2:144400257:G>A**	ZEB2	1	0,003968417	0,0313718	c.930C>T	p.Tyr310=	Not present
**chr2:169206753:G>A**	LRP2	1	0,004249517	0,0144396	c.6967C>T	p.Leu2323=	Present
**chr2:224505962:G>C**	CUL3	1	6,62572E-06	3,12285E-05	c.1200C>G	p.Val400=	Present
**chr3:38551545:C>T**	SCN5A	1	2,62509E-05	0,000228311	c.4824G>A	p.Ser1608=	Not present
**chr3:38598997:C>T**	SCN5A	1	3,84484E-05	0,000473934	c.1944G>A	p.Pro648=	Not present
**chr3:76311363:C>T**	ROBO2	1	0,00021015	0,000622665	c.76C>T	p.Pro26Ser	Present
**chr3:119393521:T>C**	ARHGAP31	1	0,001149705	0,00634371	c.936T>C	p.Arg312=	Not present
**chr4:84691741:T>C**	WDFY3	1	0,008291721	0,0132594	c.9094A>G	p.Ile3032Val	Present

**chr4:98909689:G>A**	EIF4E	1	0,002911555	0,031119	c.91C>T	p.Arg31Ter	Present
**chr4:153270426:G>A**	TRIM2	1	0,001627634	0,00230855	c.122G>A	p.Arg41His	Not present
**chr4:182773473:T>C**	TENM3	1	1,28495E-05	0,000478927	c.4894T>C	p.Tyr1632His	Not present
**chr5:83542237:G>A**	VCAN	1	0,003157617	0,00844187	c.9234G>A	p.Glu3078=	Not present
**chr5:98894690:T>G**	CHD1	1	0,000169327	0,000283507	c.1711-4A>C	Unknown	Present
**chr2:39012145:G>T**	1 SOS	1	0,000596952	0,000965251	c.2371C>A	p.Leu791Ile	Not present
**chr1:205016506:G>A**	NFASC	1	0,006702567	0,0194399	c.3690G>A	p.Thr1230=	Not present
**chr1:162780159:G>T**	DDR2	1	0,00194418	0,0233597	c.2481G>T	p.Leu827=	Not present
**chr7:83154752:G>A**	PCLO	1	4,22706E-05	0,000723589	c.1889C>T	p.Thr630Met	Present
**chr2:219055673:A>T**	IHH	1	7,95393E-06	3,02206E-05	c.770T>A	p.Phe257Tyr	Not present
**chr12:57177038:C>T**	LRP1	1	1,79352E-05	0,000058808	c.3992-3C>T	Unknown	Present
**chr8:96145470:A>G**	GDF6	1	2,87673E-05	0,000409994	c.461T>C	p.Met154Thr	Not present
**chr16:2108418:G>A**	PKD1	1	0,002617478	0,00463689	c.6749C>T	p.Thr2250Met	Present
**chr7:135587937:C>T**	NUP205	1	8,96546E-06	2,77254E-05	c.1418C>T	p.Pro473Leu	Not present
**chr5:143400648:A>C**	NR3C1	1	1,66498E-05	0,00024108	c.192T>G	p.Asp64Glu	Not present
**chr12:52517207:C>G**	KRT5	1	5,25014E-05	0,000785032	c.1118G>C	p.Gly373Ala	Not present
**chr19:41896424:A>G**	ARHGEF1	1	7,8726E-06	4,53474E-05	c.1063A>G	p.Met355Val	Not present
**chr1:162755248:C>T**	DDR2	1	0,0011154	0,0123977	c.510C>T	p.Thr170=	Not present
**chr1:8360503:C>T**	RERE	1	0,006750739	0,011971	c.3004G>A	p.Ala1002Thr	Present
**chr1:8656247:T>TCGGTCC**	RERE	1	0,00739778	0,0190279	c.45_50dup	p.Asp20_Arg21dup	Present
**chr1:35189370:G>A**	SFPQ	1	0,000435599	0,00189394	c.1428C>T	p.Thr476=	Not present
**chr1:109250529:G>A**	CELSR2	1	0,000152421	0,00161896	c.450G>A	p.Leu150=	Not present
**chr1:116584796:G>A**	IGSF3	1	0,000221469	0,00242882	c.2697C>T	p.Gly899=	Not present
**chr1:116585022:C>T**	IGSF3	1	0,004204985	0,0252069	c.2471G>A	p.Gly824Glu	Not present
**chr1:162367163:C>T**	NOS1AP	1	0,001474235	0,0159398	c.1217C>T	p.Ala406Val	Not present
**chr5:168671245:G>A**	SLIT3	1	0,002956371	0,0126728	c.4080C>T	p.Thr1360=	Not present
**chr6:7585563:C>G**	DSP	1	0,00058256	0,00471788	c.8301C>G	p.Thr2767=	Not present
**chr6:28920186:G>A**	TRIM27	1	0,000119111	0,00124327	c.573C>T	p.His191=	Not present
**chr15:43056412:T>C**	UBR1	1	0,002876552	0,0257169	c.1213A>G	p.Ser405Gly	Present
**chr12:131986551:C>T**	EP400	1	1,41072E-05	0,00094697	c.1967C>T	p.Pro656Leu	Present
**chr12:132066974:C>T**	EP400	1	1,4889E-05	2,48262E-05	c.8749+5C>T	Unknown	Present

**chr14:35689773:C>A**	RALGAPA1	1	0	0	c.2638G>T	p.Ala880Ser	Not present
**chr14:102949854:G>A**	CDC42BPB	1	0,002188251	0,00681472	c.3360C>T	p.Val1120=	Present
**chr15:41699102:A>T**	MGA	1	0,001075765	0,00458494	c.2131A>T	p.Ile711Leu	Not present
**chr15:42990087:C>T**	UBR1	1	0,002224788	0,0166811	c.3791G>A	p.Gly1264Glu	Present
**chr15:43022718:A>G**	UBR1	1	0,000787878	0,0116448	c.2823T>C	p.Phe941=	Present
**chr15:43059796:T>C**	UBR1	1	0,000235593	0,0062711	c.891A>G	p.Pro297=	Present
**chr12:100326615:T>C**	SCYL2	1	0,008223305	0,0322061	c.1510-7T>C	Unknown	Not present
**chr17:8480398:C>T**	MYH10	1	0,000355005	0,00168756	c.5388+4G>A	Unknown	Present
**chr17:28535043:C>T**	FOXN1	1	0,000437585	0,00393596	c.1472C>T	p.Ser491Leu	Not present
**chr17:40396426:G>A**	TOP2A	1	1,59102E-06	2,85775E-06	c.3577C>T	p.Leu1193Phe	Not present
**chr17:65558351:G>A**	AXIN2	1	0,000969345	0,00230415	c.270C>T	p.Asp90=	Not present
**chr18:21490109:G>A**	GREB1L	1	3,47905E-05	0,00027089	c.2788G>A	p.Asp930Asn	Not present
**chr18:22181524:C>T**	GATA6	1	0,00256061	0,00380681	c.1374C>T	p.Asn458=	Not present
**chr18:44952958:T>C**	SETBP1	1	0,007738891	0,0190202	c.3618T>C	p.His1206=	Present
**chr12:119803216:C>T**	CIT	1	0,000768165	0,0154099	c.1285G>A	p.Gly429Ser	Not present
**chr12:64126022:G>A**	SRGAP1	1	1,02449E-05	2,93997E-05	c.2270G>A	p.Arg757Gln	Not present
**chr6:160048510:A>G**	IGF2R	1	0,002294776	0,0263803	c.2481A>G	p.Ala827=	Not present
**chr10:96216688:T>C**	BLNK	1	3,18116E-06	2,86558E-05	c.572A>G	p.His191Arg	Not present
**chr6:160060530:G>A**	IGF2R	1	0,00121672	0,0135303	c.3092-17G>A	Unknown	Not present
**chr7:102249027:C>T**	CUX1	1	0,001317892	0,00846906	c.4503C>T	p.Ile1501=	Present
**chr7:152162335:C>T**	KMT2C	1	0,001213651	0,013756	c.11242G>A	p.Ala3748Thr	Present
**chr8:20216465:C>A**	ATP6V1B2	1	0,003789665	0,0128205	c.1131C>A	p.Ile377=	Not present
**chr8:47774381:GA>G**	PRKDC	1	0,000227048	0,0011534	c.12183-5del	Unknown	Present
**chr8:105801037:GTC>G**	ZFPM2	1	0,002462435	0,0265553	c.965-4_965-3del	Unknown	Not present
**chr9:131198061:T>C**	NUP214	1	0	0	c.4567T>C	p.Ser1523Pro	Not present
**chr11:1227704:A>G**	MUC5B	1	5,24993E-06	3,22331E-05	c.697A>G	p.Asn233Asp	Not present
**chr12:56189381:C>T**	SMARCC2	1	0,002908278	0,00448113	c.81G>A	p.Val27=	Present
**chr11:1251158:A>T**	MUC5B	1	2,18313E-05	3,71909E-05	c.14278A>T	p.Thr4760Ser	Not present
**chr11:14483035:T>C**	COPB1	1	0,000111135	0,000776247	c.954A>G	p.Leu318=	Not present
**chr11:66700670:T>C**	SPTBN2	1	0,001423997	0,0118292	c.3429A>G	p.Leu1143=	Not present
**chr11:72010849:T>C**	NUMA1	1	4,77266E-06	3,02206E-05	c.4656A>G	p.Glu1552=	Not present

**chr11:78708489:T>C**	TENM4	1	0,001926136	0,0129712	c.4081A>G	p.Ile1361Val	Not present
**chr12:6936728:A>ACAGCAGCAGCAGCAGCAGCAGCAGCAG**	ATN1	1	0,000953142	0,00266588	c.1482_1508dup	p.Gln494_Gln502dup	Not present
**chr12:51994050:G>A**	ACVR1B	1	1,40865E-05	0,000192678	c.1458G>A	p.Thr486=	Not present
**chr17:50572665:C>A**	CACNA1G	1	0,000349198	0,00352874	c.858C>A	p.Pro286=	Present
**chr17:50603023:A>C**	CACNA1G	1	0,003956107	0,0453603	c.3993A>C	p.Ala1331=	Present
**chr17:50626697:A>G**	CACNA1G	1	0,001762572	0,0218718	c.7080A>G	p.Lys2360=	Present
**chr7:128846393:G>A**	FLNC	1	1,28569E-05	2,77239E-05	c.4057G>A	p.Val1353Ile	Not present
**chr19:42291303:C>T**	CIC	1	6,02756E-05	0,000392054	c.5262C>T	p.Ala1754=	Present
**chr20:8881805:C>T**	PLCB1	1	9,21848E-05	0,00094697	c.3607C>T	p.Leu1203=	Present
**chr20:41493535:T>C**	CHD6	1	0,003957811	0,00772947	c.1314+3A>G	Unknown	Not present
**chr22:30932942:C>G**	MORC2	1	0,006588314	0,0216972	c.2469G>C	p.Arg823=	Not present
**chr22:31766673:GAT>G**	DEPDC5	1	0,000497965	0,0246258	c.363+6_363+7del	Unknown	Present
**chr22:40323096:A>T**	TNRC6B	1	0	0	c.5357A>T	p.Asp1786Val	Present
**chr14:21392697:C>T**	CHD8	1	0	0	c.6581G>A	p.Gly2194Asp	Present
**chr14:77027445:CTGCTGT>C**	IRF2BPL	1	0,004554247	0,0195109	c.342_347del	p.Gln126_Gln127del	Present
**chr14:77027481:C>DEL**	IRF2BPL	1	0	0,00003	Unknown	Unknown	Present
**chr4:84808355:G>A**	WDFY3	1	0	0	c.2408C>T	p.Thr803Ile	Present
**chr12:2115295:G>A**	CACNA1C	1	6,56953E-06	0,000014699	c.121G>A	p.Glu41Lys	Present
**chr16:2764142:C>A**	SRRM2	1	3,18087E-06	5,71252E-06	c.3614C>A	p.Thr1205Lys	Present
**chr9:2083380:A>INS**	SMARCA2	1	0	0	Unknown	Unknown	Present
**chr12:23536474:A>INS**	SOX5	1	0	0	Unknown	Unknown	Present
**chr4:139344283:A>INS**	NAA15	1	0	0	Unknown	Unknown	Present
**chr3:136477324:A>INS**	STAG1	1	0	0	Unknown	Unknown	Present
**chr19:42288987:T>C**	CIC	1	1,40906E-05	0,000137174	c.3758T>C	p.Val1253Ala	Present
**chr19:8592859:G>A**	ADAMTS10	1	6,42201E-06	0,000413907	c.1491C>T	p.Ser497=	Not present
**chr19:2129177:A>DEL**	AP3D1	1	0	0	Unknown	Unknown	Not present
**chr18:75286016:A>G**	TSHZ1	1	0,000967036	0,00966184	c.609A>G	p.Ala203=	Present
**chr16:81908570:A>G**	PLCG2	1	0,007082462	0,0334459	c.1712A>G	p.Asn571Ser	Not present
**chr12:111320388:C>T**	CUX2	1	0,002914221	0,0325162	c.2379C>T	p.Arg793=	Present
**chr12:122272905:G>A**	CLIP1	1	0	0	c.4287C>T	p.Ala1429=	Not present
**chr13:49733025:A>G**	KPNA3	1	4,35701E-05	0,000241429	c.136T>C	p.Leu46=	Not present
**chr14:66924264:A>G**	GPHN	1	0,006918647	0,0207329	c.800A>G	p.Asn267Ser	Present
**chr14:102050150:G>A**	DYNC1H1	1	0,001192604	0,0142237	c.13764G>A	p.Thr4588=	Present
**chr14:105146720:G>T**	JAG2	1	4,23591E-05	0,000241196	c.2484C>A	p.Ile828=	Not present

**chr15:63637592:A>G**	HERC1	1	9,6998E-06	5,74152E-05	c.12145T>C	p.Leu4049=	Present
**chr17:6471273:C>T**	PITPNM3	1	7,17919E-05	0,00047259	c.1512G>A	p.Ser504=	Not present
**chr18:21384325:G>A**	GREB1L	1	0,003306827	0,00501249	c.277G>A	p.Glu93Lys	Not present
**chr17:7514164:T>A**	POLR2A	1	0	0	c.5898T>A	p.Ser1966Arg	Present
**chr17:44194677:G>A**	ATXN7L3	1	0,003178359	0,013245	c.738-3C>T	Unknown	Not present
**chr17:63405140:G>C**	TANC2	1	0,001075754	0,0108585	c.3350G>C	p.Gly1117Ala	Present
**chr17:65558057:C>G**	AXIN2	1	8,96383E-06	0,000478469	c.564G>C	p.Gln188His	Not present
**chr17:66645465:G>A**	PRKCA	1	1,92071E-05	0,000288018	c.483G>A	p.Arg161=	Present
**chr17:76043895:A>G**	SRP68	1	0,001471189	0,0268116	c.1458T>C	p.Tyr486=	Not present
**chr18:2769782:C>T**	SMCHD1	1	0,00096901	0,00912374	c.4808C>T	p.Thr1603Ile	Not present
**chr17:61982645:A>INS**	MED13	1	0	0	Unknown	Unknown	Present
**chr19:53891785:C>T**	PRKCG	1	2,43293E-05	4,40982E-05	c.641C>T	p.Thr214Met	Not present
**chr2:148469129:A>INS**	MBD5	1	0	0	Unknown	Unknown	Present
**chr17:17797198:A>G**	RAI1	1	1,92153E-05	0,00096432	c.4250A>G	p.Lys1417Arg	Present
**chr2:213148597:C>T**	IKZF2	1	0,000839909	0,0021275	c.33G>A	p.Thr11=	Not present
**chr3:4766606:T>G**	ITPR1	1	0,002951235	0,00850622	c.5621T>G	p.Val1874Gly	Present
**chr3:47088119:G>A**	SETD2	1	0,003883356	0,00652692	c.5271C>T	p.Leu1757=	Present
**chr3:48657086:C>T**	CELSR3	1	1,02476E-05	1,99989E-05	c.4011G>A	p.Gly1337=	Not present
**chr3:49655271:C>T**	BSN	1	0,00641024	0,0268012	c.5715C>T	p.Tyr1905=	Present
**chr3:113658810:C>T**	USF3	1	0,00017543	0,000762486	c.2872G>A	p.Val958Ile	Not present
**chr3:130940688:A>G**	ATP2C1	1	1,9103E-05	0,000157625	c.419A>G	p.His140Arg	Not present
**chr3:179199700:C>T**	PIK3CA	1	0,00787241	0,0211577	c.363C>T	p.Ile121=	Present
**chr3:193615742:G>T**	OPA1	1	0,008900416	0,0176243	c.420G>T	p.Val140=	Not present
**chr4:3074876:C>CCAGCAGCAGCAGCAGCAGCAG**	HTT	1	0,009067625	0,0118734	c.90_110dup	p.Gln32_Gln38dup	Not present
**chr4:125450461:G>A**	FAT4	1	0,000946339	0,00772228	c.9451G>A	p.Ala3151Thr	Not present
**chr5:14712901:G>A**	ANKH	1	0,000153956	0,00102468	c.1338C>T	p.Ile446=	Not present
**chr5:36985474:G>A**	NIPBL	1	0,000772471	0,00345821	c.2294G>A	p.Arg765Lys	Present
**chr5:56859791:A>G**	MAP3K1	1	0,001350822	0,0224421	c.710A>G	p.Gln237Arg	Not present
**chr5:72195314:A>G**	MAP1B	1	4,50627E-05	7,74309E-05	c.1959A>G	p.Lys653=	Present
**chr5:83539768:A>G**	VCAN	1	9,54548E-06	2,28781E-05	c.6765A>G	p.Gly2255=	Not present
**chr5:138930475:G>A**	CTNNA1	1	0,002601617	0,00653424	c.2013G>A	p.Ala671=	Not present
**chr1:198708210:A>G**	PTPRC	1	0,000603796	0,00126437	c.982A>G	p.Ile328Val	Present

**chr1:151302265:C>T**	PI4KB	1	0,003643859	0,00756859	c.1554G>A	p.Pro518=	Not present
**chr1:151289154:C>T**	ZNF687	1	1,66449E-05	0,000206697	c.2354C>T	p.Thr785Met	Not present
**chr2:108772543:G>A**	RANBP2	1	0,000567405	0,00110255	c.8075G>A	p.Gly2692Asp	Not present
**chr2:159432800:ATCT>A**	BAZ2B	1	5,50799E-05	0,000482393	c.1854_1856del	p.Glu618del	Present
**chr4:139137654:G>A**	ELF2	1	4,77286E-06	1,43287E-05	c.48C>T	p.Ser16=	Not present
**chr14:58270470:A>INS**	PSMA3, PSMA3-AS1	1	0	0	Unknown	Unknown	Not present
**chr8:60816369:A>INS**	CHD7	1	0	0	Unknown	Unknown	Present
**chr7:42026219:C>T**	GLI3	1	0,000164088	0,00119173	c.1222G>A	p.Gly408Ser	Present
**chr20:41115408:A>INS**	TOP1, PLCG1-AS1	1	0	0	Unknown	Unknown	Not present
**chr14:101760688:G>T**	PPP2R5C	1	0,00013478	0,00159405	c.4G>T	p.Gly2Cys	Present
**chr10:102398304:A>INS**	NFKB2	1	0	0	Unknown	Unknown	Not present
**chr1:35560382:C>T**	NCDN	1	1,40876E-05	2,77239E-05	c.231C>T	p.Phe77=	Not present
**chr8:106750812:A>INS**	OXR1	1	0	0	Unknown	Unknown	Not present
**chr6:7189230:C>G**	RREB1	1	2,30815E-05	0,000206782	c.333C>G	p.Ser111=	Not present
**chr19:1226556:C>T**	STK11	1	0,000602842	0,00173784	c.1211C>T	p.Ser404Phe	Not present
**chr8:47918371:T>C**	PRKDC	1	4,82181E-05	0,000207039	c.2432A>G	p.Asn811Ser	Present
**chr10:21735130:A>INS**	MLLT10	1	0	0	Unknown	Unknown	Not present
**chr7:82953722:G>T**	PCLO	1	5,01748E-05	9,17883E-05	c.7231C>A	p.Pro2411Thr	Present
**chr1:35559102:C>G**	NCDN	1	0	0	c.34-5C>G	Unknown	Not present
**chr12:51806598:A>T**	SCN8A	1	3,18081E-06	6,04266E-05	c.5112A>T	p.Ser1704=	Present
**chr12:49044470:A>G**	KMT2D	1	0,002329853	0,0274669	c.5016T>C	p.Pro1672=	Not present
**chr12:49042222:C>T**	KMT2D	1	0,003278004	0,0363334	c.5976G>A	p.Glu1992=	Not present
**chr11:128480393:G>A**	ETS1	1	0,008461034	0,0118981	c.921C>T	p.Arg307=	Not present
**chr1:53793240:A>G**	NDC1	1	0,000806722	0,00876697	c.1624T>C	p.Phe542Leu	Not present
**chr1:65384271:A>G**	DNAJC6	1	0,001431392	0,0109447	c.745A>G	p.Ile249Val	Not present
**chr1:119937285:G>A**	NOTCH2	1	0,001406345	0,020649	c.3519C>T	p.Cys1173=	Not present
**chr1:151287884:C>T**	ZNF687	1	0,000462343	0,00094697	c.1593C>T	p.Thr531=	Not present
**chr1:151404946:A>C**	POGZ	1	0,001437823	0,00578592	c.4089T>G	p.His1363Gln	Present
**chr2:25767743:C>T**	ASXL2	1	0,000171762	0,000572659	c.632-17G>A	Unknown	Not present
**chr2:50053466:T>C**	NRXN1	1	0,004940441	0,0137416	c.3933A>G	p.Ala1311=	Present
**chr2:169152880:C>T**	LRP2	1	0,002339339	0,0255958	c.12380G>A	p.Arg4127His	Present

**chr2:169243501:G>A**	LRP2	1	0,009127064	0,0342198	c.3452C>T	p.Pro1151Leu	Present
**chr2:232343413:A>AGGG**	DIS3L2	1	0,002934031	0,004285	c.1651_1652insGGG	p.Glu550_Ala551insGly	Not present
**chr3:9473449:A>G**	SETD5	1	0,002426015	0,0244085	c.3409A>G	p.Met1137Val	Present
**chr3:14443662:C>T**	SLC6A6	1	0,003019078	0,0353952	c.28C>T	p.Leu10=	Not present
**chr3:169131499:C>T**	MECOM	1	0,001265589	0,0139109	c.543G>A	p.Pro181=	Not present
**chr3:193614733:C>A**	OPA1	1	0,006089457	0,0245383	c.43C>A	p.Gln15Lys	Not present
**chr3:193615011:G>A**	OPA1	1	0,005806316	0,0245383	c.321G>A	p.Ser107=	Not present
**chr3:193643409:A>C**	OPA1	1	0,006097889	0,0245792	c.1342A>C	p.Arg448=	Not present
**chr4:71486986:G>A**	SLC4A4	1	0,000751216	0,00776647	c.1942G>A	p.Glu648Lys	Not present
**chr1:10642324:T>A**	CASZ1	1	3,61788E-05	0,000235405	c.4162+535A>T	Unknown	Present
**chr4:143543584:A>INS**	SMARCA5	1	0	0	Unknown	Unknown	Not present
**chr3:101347899:A>INS**	SENP7	1	0	0	Unknown	Unknown	Not present
**chr19:15272807:C>T**	BRD4	1	2,57083E-06	1,46977E-05	c.285+8G>A	Unknown	Present
**chr1:185091648:A>INS**	RNF2	1	0	0	Unknown	Unknown	Not present
**chr4:152411301:A>INS**	FBXW7	1	0	0	Unknown	Unknown	Not present
**chr5:138379623:A>INS**	KDM3B	1	0	0	Unknown	Unknown	Present
**chr2:189010813:A>INS**	COL3A1	1	0	0	Unknown	Unknown	Not present
**chr5:61543457:G>A**	ZSWIM6	1	0,000356443	0,0019802	c.2788G>A	p.Val930Ile	Present
**chr1:186346155:T>C**	TPR	1	0	0	c.3076A>G	p.Ile1026Val	Not present
**chr17:44260432:G>T**	SLC4A1	1	0,000540556	0,0109447	c.457C>A	p.Leu153Met	Not present
**chr7:152221999:A>INS**	KMT2C	1	0	0	Unknown	Unknown	Present
**chr19:49596821:G>A**	PRR12	1	0,000626976	0,00315259	c.2486G>A	p.Arg829His	Present
**chr4:95131511:A>G**	BMPR1B	1	0,000185678	0,00192957	c.1075A>G	p.Ser359Gly	Not present
**chr12:119757408:T>C**	CIT	1	0	0	c.2669A>G	p.Lys890Arg	Not present
**chr3:173605569:G>A**	NLGN1	1	0,000216928	0,000949668	c.529G>A	p.Gly177Ser	Present
**chr2:189061568:A>INS**	COL5A2	1	0	0	Unknown	Unknown	Not present
**chr11:7992082:A>T**	EIF3F	1	0	0	c.436-2A>T	Unknown	Present
**chr6:72313543:A>INS**	RIMS1	1	0	0	Unknown	Unknown	Present
**chr15:52372320:C>T**	MYO5A	1	1,29351E-05	5,55031E-05	c.2621G>A	p.Arg874Gln	Present
**chr4:81092465:AAAGGAAGGAAGGAAGG>A**	PRKG2	1	0,002759636	0,018972	c.2127-29_2127-14del	Unknown	Not present
**chr4:81140592:C>G**	PRKG2	1	0	0	c.1485G>C	p.Gln495His	Not present
**chr4:103123040:G>C**	CENPE	1	0,00734665	0,0244957	c.6974C>G	p.Ser2325Cys	Not present
**chr10:1084471:C>T**	WDR37	1	4,2254E-05	0,00024108	c.465C>T	p.His155=	Present
**chr8:72937617:C>A**	KCNB2	1	0,000242	0,00265188	c.2262C>A	p.Thr754=	Not present
**chr8:100137571:A>INS**	FBXO43	1	0	0	Unknown	Unknown	Not present
**chr8:127738361:G>A**	MYC	1	0,003399818	0,0379878	c.144G>A	p.Gln48=	Not present
**chr9:916825:G>A**	DMRT1	1	1,79244E-05	0,000473485	c.885G>A	p.Pro295=	Not present

**chr9:126696286:C>T**	LMX1B	1	2,3054E-05	0,000285987	c.1052-8C>T	Unknown	Present
**chr9:132300812:CAAG>C**	SETX	1	0,001758921	0,0184525	c.5375-12_5375-10del	Unknown	Not present
**chr9:137028726:A>C**	ABCA2	1	2,08455E-05	0,000244618	c.147T>G	p.Pro49=	Present
**chr10:29532007:C>T**	SVIL	1	6,14645E-05	0,000102834	c.2004G>A	p.Ser668=	Not present
**chr7:98882032:C>T**	TRRAP	1	0,002532708	0,0286457	c.150+8C>T	Unknown	Present
**chr10:60145969:T>C**	ANK3	1	0,008591765	0,0224274	c.2616A>G	p.Lys872=	Present
**chr11:7995959:G>T**	EIF3F	1	0	0	c.1011G>T	p.Val337=	Present
**chr11:8731213:G>T**	DENND2B	1	0,005402396	0,0154534	c.81-4C>A	Unknown	Present
**chr11:30011791:G>A**	KCNA4	1	1,59045E-06	2,85636E-06	c.888C>T	p.Leu296=	Not present
**chr11:61314089:A>G**	DDB1	1	2,56075E-05	0,000241196	c.1711T>C	p.Leu571=	Not present
**chr11:74636253:C>T**	POLD3	1	0,004957901	0,0140383	c.1176C>T	p.Tyr392=	Not present
**chr11:74636254:C>G**	POLD3	1	0,004954198	0,0140351	c.1177C>G	p.Leu393Val	Not present
**chr7:148829738:G>A**	EZH2	1	7,73557E-05	0,000474834	c.474C>T	p.His158=	Not present
**chr7:97024372:G>C**	DLX5	1	0,001056151	0,00259217	c.252C>G	p.Ala84=	Not present
**chr4:113357679:G>A**	ANK2	1	0,001553093	0,0190525	c.9061G>A	p.Ala3021Thr	Present
**chr5:83539126:C>T**	VCAN	1	0,003552857	0,0412451	c.6123C>T	p.Ile2041=	Not present
**chr4:113373380:C>T**	ANK2	1	0,003964138	0,0336479	c.11790C>T	p.Ile3930=	Present
**chr4:125317512:A>G**	FAT4	1	0,004211249	0,047281	c.1101A>G	p.Val367=	Not present
**chr4:125320565:T>C**	FAT4	1	0,001709286	0,019783	c.4154T>C	p.Leu1385Ser	Not present
**chr5:51389734:C>T**	ISL1	1	0,005791234	0,0115894	c.567C>T	p.Asn189=	Not present
**chr5:56882021:T>TCAA**	MAP3K1	1	0,000587967	0,00191041	c.2845_2847dup	p.Thr949dup	Not present
**chr5:83538158:A>T**	VCAN	1	0,003643661	0,0425911	c.5155A>T	p.Thr1719Ser	Not present
**chr5:83538579:C>A**	VCAN	1	0,00364083	0,0425681	c.5576C>A	p.Ala1859Glu	Not present
**chr5:83539240:G>A**	VCAN	1	0,003530415	0,0411532	c.6237G>A	p.Lys2079=	Not present
**chr7:41700030:T>C**	INHBA	1	0,003185971	0,0437318	c.345A>G	p.Glu115=	Not present
**chr5:98897315:T>C**	CHD1	1	0,001078781	0,0127084	c.1371A>G	p.Leu457=	Present
**chr5:131671658:G>C**	FNIP1	1	9,09186E-05	0,00048216	c.2786C>G	p.Thr929Ser	Not present
**chr5:132934659:T>G**	AFF4	1	0,002916654	0,0343619	c.406A>C	p.Thr136Pro	Not present
**chr6:7230718:C>T**	RREB1	1	3,66484E-05	0,000154693	c.2619C>T	p.Gly873=	Not present
**chr6:35239871:C>G**	SCUBE3	1	0,00195512	0,0154414	c.949C>G	p.Gln317Glu	Not present

**chr6:68639296:C>T**	ADGRB3	1	0,000121677	0,000663009	c.621C>T	p.Ile207=	Not present
**chr6:75184025:C>A**	COL12A1	1	3,71347E-05	0,000182932	c.1117G>T	p.Ala373Ser	Present
**chr5:143400622:G>A**	NR3C1	1	1,27234E-05	0,00024108	c.218C>T	p.Ala73Val	Not present
**chr5:150447645:A>G**	RPS14	1	0,000194626	0,00146153	c.89T>C	p.Val30Ala	Not present
**chr5:168817394:T>C**	SLIT3	1	0,00056972	0,00457033	c.699A>G	p.Arg233=	Not present
**chr15:40625036:A>G**	KNL1	1	0,000386743	0,00459355	c.4772A>G	p.Asn1591Ser	Not present
**chr3:63982349:A>T**	ATXN7	1	0,001319998	0,00215743	c.916A>T	p.Ile306Phe	Not present
**chr3:180951420:C>T**	FXR1	1	0,009570506	0,0491105	c.753C>T	p.Thr251=	Not present
**chr5:11397049:C>T**	CTNND2	1	0,000426691	0,00429816	c.594G>A	p.Thr198=	Present
**chr5:128393315:T>C**	FBN2	1	0,001462134	0,0174687	c.1285A>G	p.Ser429Gly	Not present
**chr5:150368840:C>T**	TCOF1	1	0,000555729	0,00691244	c.503C>T	p.Thr168Met	Not present
**chr6:7230973:G>A**	RREB1	1	3,8417E-06	3,02188E-05	c.2874G>A	p.Lys958=	Not present
**chr6:10402555:A>G**	TFAP2A	1	0,001823107	0,0232282	c.826T>C	p.Leu276=	Not present
**chr6:44149842:C>T**	TMEM63B	1	0,002453786	0,0258953	c.1414-17C>T	Unknown	Not present
**chr6:75095135:T>C**	COL12A1	1	0,000418707	0,000731663	c.8622A>G	p.Pro2874=	Present
**chr7:50755879:G>A**	GRB10	1	0,000867672	0,00557103	c.-47+8C>T	Unknown	Present
**chr7:74691092:A>G**	GTF2I	1	0,00051771	0,00236295	c.219A>G	p.Gln73=	Present
**chr7:103565502:G>C**	RELN	1	0,001178599	0,0143633	c.4986C>G	p.Thr1662=	Present
**chr7:103565554:GA>G**	RELN	1	0,009747916	0,0249119	c.4937-4del	Unknown	Present
**chr7:103661529:T>TA**	RELN	1	0,004389992	0,0487954	c.1290-3dup	Unknown	Present
**chr7:116699474:C>T**	MET	1	0,002008027	0,0237144	c.390C>T	p.Leu130=	Present
**chr7:116700169:T>C**	MET	1	0,001995793	0,0237179	c.1085T>C	p.Met362Thr	Present
**chr7:155803671:G>A**	SHH	1	0	0	c.618C>T	p.His206=	Not present
**chr3:12936356:G>A**	IQSEC1	1	0,000434665	0,00682451	c.660C>T	p.Asp220=	Not present
**chr2:215407248:G>A**	FN1	1	0,002994702	0,0337939	c.2592C>T	p.Ser864=	Not present
**chr2:190985665:A>G**	STAT1	1	0,001643966	0,00299497	c.1222-5T>C	Unknown	Not present
**chr1:61406555:C>A**	NFIA	1	0,003677251	0,0459614	c.1255-7C>A	Unknown	Present
**chr12:117234566:T>C**	NOS1	1	0,000266808	0,0026751	c.3234A>G	p.Leu1078=	Not present
**chr5:154814020:C>T**	LARP1	1	0,000628634	0,0048216	c.3215C>T	p.Pro1072Leu	Present
**chr22:19353502:G>A**	HIRA	1	4,89126E-05	0,000475299	c.2702C>T	p.Ala901Val	Not present

**chr1:3417945:C>G**	PRDM16	1	8,97307E-05	0,000988857	c.2809C>G	p.Pro937Ala	Not present
**chr1:3186110:C>T**	PRDM16	1	0,001899926	0,0210759	c.38-15C>T	Unknown	Not present
**chr1:7664637:C>T**	CAMTA1	1	0,001095222	0,0110934	c.2090C>T	p.Ala697Val	Not present
**chr1:10475010:G>A**	PEX14	1	0,00148892	0,0221902	c.36+8G>A	Unknown	Not present
**chr1:109250113:A>ACGCCGCCGC**	CELSR2	1	2,95433E-05	0,000481696	c.41_49dup	p.Pro14_Pro16dup	Not present
**chr2:165389725:C>T**	SCN2A	1	0,002593693	0,0278752	c.5919C>T	p.Pro1973=	Present
**chr1:158094745:C>T**	KIRREL1	1	0,000244722	0,000458355	c.1899C>T	p.Phe633=	Not present
**chr1:169130042:A>C**	ATP1B1	1	0,006849245	0,0101058	c.600A>C	p.Pro200=	Not present
**chr1:173910823:G>A**	SERPINC1	1	0,000231738	0,0026962	c.693C>T	p.Thr231=	Not present
**chr1:181733588:C>T**	CACNA1E	1	0	0	c.3100C>T	p.Leu1034=	Present
**chr1:218434067:C>T**	TGFB2	1	0	0	c.511-15C>T	Unknown	Not present
**chr2:55232876:C>CGGGCCGGGTGGTCATGAGG**	RPS27A	1	0,001839584	0,0217827	c.48+5_48+23dup	Unknown	Not present
**chr2:120955409:G>A**	GLI2	1	1,93248E-05	6,53339E-05	c.622G>A	p.Asp208Asn	Not present
**chr8:72937230:C>T**	KCNB2	1	0,000806716	0,00616028	c.1875C>T	p.Ser625=	Not present
**chr8:144790355:A>G**	RPL8	1	1,53711E-05	0,000959693	c.615T>C	p.Asn205=	Not present
**chr9:35548052:C>T**	RUSC2	1	0,000668816	0,00129332	c.1531C>T	p.Leu511=	Not present
**chr15:28274306:C>T**	HERC2	1	7,68136E-06	0,000226065	c.785G>A	p.Arg262Lys	Present
**chr12:78006615:C>A**	NAV3	1	0,000354671	0,00433944	c.1077C>A	p.Pro359=	Present
**chr12:102958393:CGCAGCAGCA>C**	ASCL1	1	0,003388389	0,0142857	c.178_186del	p.Gln60_Gln62del	Not present
**chr12:117278051:G>T**	NOS1	1	0,000122927	0,00130753	c.1572C>A	p.Val524=	Not present
**chr12:131961952:G>A**	EP400	1	5,15322E-06	3,90427E-05	c.1333G>A	p.Glu445Lys	Present
**chr13:28057405:T>C**	FLT3	1	5,89043E-05	0,00062168	c.426A>G	p.Leu142=	Not present
**chr14:21386156:C>T**	CHD8	1	0,00030744	0,00224064	c.7203G>A	p.Val2401=	Present
**chr14:95117758:A>C**	DICER1	1	0,003306645	0,0168837	c.1377-4T>G	Unknown	Not present
**chr15:41727223:A>G**	MGA	1	0,003946094	0,0458347	c.3474A>G	p.Pro1158=	Not present
**chr12:15669817:A>G**	EPS8	1	0,002958479	0,0328105	c.213T>C	p.Thr71=	Not present
**chr15:42983903:G>A**	UBR1	1	0,001324011	0,0173918	c.4144C>T	p.Leu1382=	Present
**chr15:88135132:G>A**	NTRK3	1	0,000294435	0,0012054	c.1173C>T	p.Ile391=	Present
**chr16:28834672:C>T**	ATXN2L	1	0,003784161	0,0443854	c.2412C>T	p.Pro804=	Not present
**chr16:69692043:T>C**	NFAT5	1	6,40262E-06	0,00072324	c.2218T>C	p.Leu740=	Not present
**chr16:72787715:CGCCACCGCCGCCGCCGCCGCCACT>C**	ZFHX3	1	0,001551022	0,00988142	c.10537_10560del	p.Ser3513_Gly3520del	Present

**chr16:87689758:T>C**	JPH3	1	0,000577467	0,00656257	c.1398T>C	p.Thr466=	Not present
**chr17:8493900:T>A**	MYH10	1	0,007135657	0,0231101	c.4057-15A>T	Unknown	Present
**chr12:57180149:C>T**	LRP1	1	1,59186E-06	0,00024108	c.5236+8C>T	Unknown	Present
**chr12:6935973:A>C**	ATN1	1	0,003989958	0,00638667	c.706A>C	p.Lys236Gln	Not present
**chr9:81593279:T>G**	TLE1	1	0,003480919	0,0401108	c.1332-5A>C	Unknown	Not present
**chr10:96224006:A>G**	BLNK	1	0,006926711	0,0171158	c.362-17T>C	Unknown	Not present
**chr9:105362874:C>A**	SLC44A1	1	0,000911251	0,0107143	c.954C>A	p.Ile318=	Not present
**chr9:127818296:C>T**	ENG	1	0,001208707	0,0087082	c.1510G>A	p.Val504Met	Not present
**chr9:127818354:G>A**	ENG	1	0,001173327	0,00873394	c.1452C>T	p.Ser484=	Not present
**chr9:128259034:A>G**	GOLGA2	1	2,18499E-05	0,000120581	c.2146T>C	p.Leu716=	Not present
**chr9:137013490:G>A**	ABCA2	1	3,99159E-05	0,00193237	c.4521C>T	p.Pro1507=	Present
**chr10:60084779:C>T**	ANK3	1	0,001249812	0,00295276	c.3897G>A	p.Thr1299=	Present
**chr10:96216739:C>G**	BLNK	1	0,007938561	0,0240331	c.526-5G>C	Unknown	Not present
**chr10:110132308:C>G**	ADD3	1	0,001110117	0,0130938	c.1736C>G	p.Ala579Gly	Not present
**chr12:333666:C>T**	KDM5A	1	0,007099654	0,0100039	c.1491-17G>A	Unknown	Present
**chr11:1247532:C>G**	MUC5B	1	0,000168448	0,000730105	c.10652C>G	p.Ser3551Trp	Not present
**chr11:1249717:C>T**	MUC5B	1	0,002593242	0,0076555	c.12837C>T	p.Pro4279=	Not present
**chr11:62626411:G>A**	GANAB	1	0,002802537	0,0291059	c.2548C>T	p.His850Tyr	Not present
**chr11:66234199:T>A**	PACS1	1	0,004089116	0,0474737	c.2061T>A	p.Ser687=	Present
**chr11:72022422:A>G**	NUMA1	1	0,001828676	0,0135363	c.292-3T>C	Unknown	Not present
**chr11:118503581:A>G**	KMT2A	1	0,002452982	0,0258845	c.7689A>G	p.Ser2563=	Present
**chr11:118506101:G>A**	KMT2A	1	0,002568541	0,0266757	c.10209G>A	p.Pro3403=	Present
**chr11:108161676:C>T**	NPAT	1	1,40909E-05	0,00024108	c.3410G>A	p.Arg1137Gln	Not present
**chr15:40621076:C>G**	KNL1	1	0,000344889	0,00404546	c.812C>G	p.Thr271Ser	Not present
**chr5:180603376:C>G**	FLT4	1	0,003334853	0,0149903	c.3908G>C	p.Gly1303Ala	Not present
**chr6:159785898:A>INS**	MRPL18, TCP1	1	0	0	Unknown	Unknown	Not present
**chr11:8715824:G>A**	DENND2B	1	6,31913E-05	0,00173544	c.1630-6C>T	Unknown	Present
**chr11:8717750:C>T**	DENND2B	1	0,000324877	0,00255336	c.1620G>A	p.Pro540=	Present
**chr11:17771925:G>T**	KCNC1	1	5,24981E-05	0,000477555	c.831G>T	p.Ser277=	Present

**chr11:72012930:G>A**	NUMA1	1	2,17713E-05	0,000339098	c.4573C>T	p.Arg1525Trp	Not present
**chr11:113412736:C>T**	DRD2	1	3,0739E-05	5,73115E-05	c.958G>A	p.Asp320Asn	Present
**chr11:116846420:G>A**	SIK3	1	0,003342799	0,00839229	c.4086C>T	p.Phe1362=	Not present
**chr12:6601978:A>C**	CHD4	1	0,002979248	0,0384615	c.420T>G	p.Asp140Glu	Present
**chr12:54512025:C>A**	NCKAP1L	1	1,40849E-05	0,000288351	c.861C>A	p.Gly287=	Not present
**chr12:122364081:G>T**	CLIP1	1	4,7713E-06	8,56913E-06	c.684C>A	p.Val228=	Not present
**chr13:110150453:C>T**	COL4A1	1	3,58665E-05	6,86059E-05	c.4929-9G>A	Unknown	Not present
**chr14:35686600:T>A**	RALGAPA1	1	0,000153021	0,0046729	c.4019A>T	p.Asn1340Ile	Not present
**chr14:35686612:C>G**	RALGAPA1	1	0,00015049	0,00451322	c.4007G>C	p.Gly1336Ala	Not present
**chr15:33742372:C>G**	RYR3	1	0,001143863	0,00536579	c.7827C>G	p.Ser2609=	Not present
**chr15:38349514:C>T**	SPRED1	1	0,000986358	0,00968054	c.675C>T	p.Ser225=	Not present
**chr15:48492594:GA>G**	FBN1	1	0,000129937	0,000840689	c.2729-9del	Unknown	Present
**chr15:52405409:G>A**	MYO5A	1	0,001033771	0,0116595	c.947-16C>T	Unknown	Present
**chr16:30723090:T>C**	SRCAP	1	3,84203E-06	3,02133E-05	c.4020T>C	p.Ala1340=	Present
**chr10:63215676:G>A**	JMJD1C	1	0,001467784	0,0136957	c.699C>T	p.Val233=	Present
**chr9:136508263:G>A**	NOTCH1	1	0,003313714	0,00521327	c.3294C>T	p.Ser1098=	Present
**chr9:128625810:C>T**	SPTAN1	1	0,002358321	0,00713487	c.6111C>T	p.Gly2037=	Not present
**chr7:128843792:A>T**	FLNC	1	2,30514E-05	4,40982E-05	c.2812-4A>T	Unknown	Not present
**chr6:7581408:G>A**	DSP	1	0,001489018	0,0197686	c.5218G>A	p.Glu1740Lys	Not present
**chr6:7727435:C>T**	BMP6	1	0,003057632	0,0100923	c.480C>T	p.Ser160=	Not present
**chr6:29612631:G>A**	GABBR1	1	0,00124338	0,00988428	c.1567-17C>T	Unknown	Not present
**chr6:163535133:A>G**	QKI	1	0,000722118	0,00902907	c.546+8A>G	Unknown	Not present
**chr7:39951707:C>G**	CDK13	1	0,002896652	0,00442816	c.1066C>G	p.Pro356Ala	Present
**chr7:83156131:T>C**	PCLO	1	1,59086E-06	3,02334E-05	c.510A>G	p.Val170=	Present
**chr7:116771626:G>C**	MET	1	6,36483E-06	1,14327E-05	c.2859G>C	p.Leu953=	Present
**chr7:138554892:C>T**	TRIM24	1	1,66475E-05	2,94031E-05	c.1456C>T	p.Arg486Trp	Not present
**chr9:34251508:C>T**	UBAP1	1	0,000172842	0,00144648	c.1485C>T	p.Leu495=	Not present
**chr7:152180043:T>C**	KMT2C	1	0,000834784	0,0036162	c.7233A>G	p.Ser2411=	Present
**chr8:28717004:C>T**	EXTL3	1	4,09687E-05	6,04266E-05	c.945C>T	p.Val315=	Not present
**chr8:67232956:TA>T**	ARFGEF1	1	0,002540631	0,0052381	c.3290-12del	Unknown	Not present

**chr8:76863078:C>G**	ZFHX4	1	0,002438758	0,00493763	c.9380-16C>G	Unknown	Present
**chr8:106692820:G>A**	OXR1	1	0,004075093	0,0171663	c.618G>A	p.Glu206=	Not present
**chr8:116847703:C>T**	RAD21	1	0,000219401	0,00285171	c.1705-12G>A	Unknown	Present
**chr9:34250741:C>T**	UBAP1	1	8,97229E-06	2,93971E-05	c.1350C>T	p.His450=	Not present
**chr17:39766369:G>A**	IKZF3	1	0,003868972	0,012724	c.951C>T	p.Ala317=	Not present
**chr17:47743077:G>A**	TBX21	1	0,000234347	0,00115274	c.653G>A	p.Arg218His	Not present
**chr17:67886231:C>A**	BPTF	1	2,56148E-06	1,46977E-05	c.2157C>A	p.Ser719=	Not present
**chr19:49597063:G>A**	PRR12	1	1,11144E-05	0,000245459	c.2728G>A	p.Ala910Thr	Present
**chr20:50894322:G>A**	ADNP	1	3,34257E-05	0,000482859	c.392C>T	p.Pro131Leu	Present
**chr6:159785385:C>INS**	TCP1	1	0	0	Unknown	Unknown	Not present
**chr2:28412007:G>A**	FOSL2	1	6,57151E-06	1,46994E-05	c.540G>A	p.Leu180=	Not present
**chr6:159780566:G>DEL**	TCP1	1	0	0,005777	Unknown	Unknown	Not present
**chr11:68413909:C>G**	LRP5	1	8,46842E-05	0,00072324	c.2724C>G	p.His908Gln	Not present
**chr5:150131999:G>C**	PDGFRB	1	0,000616036	0,00388508	c.1223C>G	p.Ser408Cys	Not present
**chr17:8478436:C>T**	MYH10	1	4,99334E-05	0,00192864	c.5608G>A	p.Ala1870Thr	Present
**chr19:2114213:T>C**	AP3D1	1	5,25347E-05	0,0065123	c.2513A>G	p.Glu838Gly	Not present
**chr11:4133572:C>G**	RRM1	1	6,57237E-06	1,47003E-05	c.1915C>G	p.Pro639Ala	Not present
**chr4:48547728:G>A**	FRYL	1	0,000158834	0,00094697	c.4930C>T	p.Arg1644Cys	Present
**chr18:22171206:C>G**	GATA6	1	0,000173468	0,00152784	c.62C>G	p.Ala21Gly	Not present
**chr15:51536693:G>C**	DMXL2	1	0,000353415	0,00723589	c.1787C>G	p.Ser596Cys	Present
**chr4:103136208:A>G**	CENPE	1	9,73578E-05	0,000176465	c.6455T>C	p.Ile2152Thr	Not present
**chr4:182679766:G>A**	TENM3	1	0,000174167	0,00189215	c.1427G>A	p.Ser476Asn	Not present
**chr1:151668631:A>G**	SNX27	1	5,13937E-06	0,000206782	c.1145A>G	p.His382Arg	Not present
**chr12:47975545:T>A**	COL2A1	1	0	0	c.3658A>T	p.Met1220Leu	Not present
**chr6:159779789:C>DEL**	TCP1	1	0	0,000237	Unknown	Unknown	Not present
**chr9:131144592:C>T**	NUP214	1	0,000101161	0,000482393	c.1607C>T	p.Ala536Val	Not present
**chr17:77497305:C>T**	SEPTIN9	1	0,004733798	0,00709984	c.1574-10C>T	Unknown	Not present
**chr19:55655139:A>ACGAGAATAAACAAGGTGAGGGCACCGGGGTCG**	U2AF2	1	0,0001412	0,000245122	c.38_49+20dup	Unknown	Present
**chr17:82079272:G>A**	FASN	1	0,000307713	0,0019595	c.7407C>T	p.Asp2469=	Not present
**chr19:18122662:C>T**	MAST3	1	6,5697E-06	2,41266E-05	c.321-11C>T	Unknown	Present
**chr19:18135783:C>T**	MAST3	1	0,001862511	0,0208444	c.1914C>T	p.Asp638=	Present

**chr19:45037613:C>T**	RELB	1	0,001780887	0,00264692	c.1563C>T	p.Ala521=	Not present
**chr19:49139651:G>A**	PPFIA3	1	0,009245188	0,0160471	c.2077-17G>A	Unknown	Present
**chr19:53906388:C>T**	PRKCG	1	0,000822427	0,00176767	c.1836C>T	p.Gly612=	Not present
**chr20:63407157:C>T**	KCNQ2	1	0,001818407	0,0206988	c.2106G>A	p.Ser702=	Present
**chr6:159781110:T>DEL**	TCP1	1	0	0	Unknown	Unknown	Not present
**chr22:42209870:G>A**	TCF20	1	0	0	c.5436C>T	p.Ala1812=	Present
**chr6:159779260:T>DEL**	TCP1	1	0	0	Unknown	Unknown	Not present
**chr6:159780087:A>DEL**	TCP1, SNORA20	1	0	0	Unknown	Unknown	Not present
**chr6:159787870:T>DEL**	TCP1	1	0	0	Unknown	Unknown	Not present
**chr6:159788143:A>DEL**	TCP1	1	0	0	Unknown	Unknown	Not present
**chr9:131197281:CCTT>C**	NUP214	1	0,0003252	0,00094518	c.3791_3793del	p.Ser1264del	Not present
**chr10:110964942:C>T**	SHOC2	1	1,59136E-06	3,02151E-05	c.584C>T	p.Thr195Ile	Not present
**chr13:110169746:C>T**	COL4A1	1	0	0	c.3759G>A	p.Met1253Ile	Not present
**chr3:47678213:A>INS**	SMARCC1	1	0	0	Unknown	Unknown	Not present
**chr3:179219590:A>INS**	PIK3CA	1	0	0	Unknown	Unknown	Present
**chr18:47845375:T>C**	SMAD2	1	5,63523E-05	0,00048216	c.1245A>G	p.Arg415=	Not present
**chr5:14741851:G>A**	ANKH	1	0,000573597	0,00625963	c.987C>T	p.Phe329=	Not present
**chr11:118382653:G>A**	UBE4A	1	0	0	c.2074G>A	p.Asp692Asn	Not present
**chr6:137878706:C>A**	TNFAIP3	1	1,28053E-05	0,000137174	c.1261C>A	p.Leu421Met	Not present
**chr19:49597030:G>A**	PRR12	1	3,17457E-05	0,000494315	c.2695G>A	p.Val899Ile	Present
**chr15:69440058:T>C**	KIF23	1	1,97246E-05	0,000029404	c.1910T>C	p.Met637Thr	Not present
**chr10:76953887:C>A**	KCNMA1	1	0,000249683	0,0054755	c.2398G>T	p.Asp800Tyr	Present
**chr3:14416410:A>G**	SLC6A6	1	0	0	c.-53-2A>G	Unknown	Not present
**chr13:114324287:ACTC>A**	CHAMP1	1	0,000102463	0,000982704	c.449_451del	p.Pro150del	Present
**chr7:44226560:G>A**	CAMK2B	1	0,000538867	0,00237192	c.1553C>T	p.Pro518Leu	Present
**chr7:40088190:G>A**	CDK13	1	9,60637E-05	0,000576369	c.3094G>A	p.Gly1032Ser	Present
**chr2:69145363:C>A**	ANTXR1	1	3,39294E-05	0,000241546	c.989C>A	p.Ala330Asp	Not present
**chr12:49041333:G>A**	KMT2D	1	0,000239321	0,000784519	c.6437C>T	p.Pro2146Leu	Not present
**chr17:31993273:A>C**	SUZ12	1	0,000309757	0,00418591	c.1233A>C	p.Gln411His	Not present
**chr1:12342930:G>A**	VPS13D	1	1,59203E-06	2,86033E-06	c.8764G>A	p.Val2922Ile	Not present
**chr7:98895799:C>T**	TRRAP	1	1,12712E-05	0,000030499	c.486C>T	p.Tyr162=	Present
**chr5:36985627:G>A**	NIPBL	1	0,000395739	0,00806916	c.2447G>A	p.Arg816His	Present
**chr17:50576048:G>C**	CACNA1G	1	0,000484388	0,00365141	c.1646G>C	p.Gly549Ala	Present
**chr7:83156330:C>T**	PCLO	1	0,000361631	0,00844187	c.311G>A	p.Arg104His	Present

**chr2:108782681:A>G**	RANBP2	1	3,18078E-06	5,71259E-06	c.9188A>G	p.Asn3063Ser	Not present
**chr3:187726893:C>T**	BCL6	1	8,96468E-06	3,02224E-05	c.1546G>A	p.Gly516Arg	Not present
**chr10:13747765:G>A**	FRMD4A	1	6,40526E-05	0,00072324	c.519C>T	p.Ala173=	Not present
**chr19:41987936:G>A**	ATP1A3	1	0,002302476	0,00914302	c.357C>T	p.Asn119=	Present
**chr19:4930717:T>C**	UHRF1	1	0,002096345	0,0249663	c.410T>C	p.Val137Ala	Not present
**chr19:4932780:C>T**	UHRF1	1	0,002193994	0,0251419	c.609C>T	p.Asp203=	Not present
**chr19:5144265:C>T**	KDM4B	1	0,000815398	0,00345267	c.2754C>T	p.Ala918=	Present
**chr19:10797398:C>T**	DNM2	1	7,69223E-05	0,000582266	c.1215C>T	p.Pro405=	Not present
**chr19:12865070:C>T**	MAST1	1	0,00471558	0,0105634	c.1530C>T	p.His510=	Present
**chr19:18850874:G>C**	UPF1	1	0	0	c.810+6G>C	Unknown	Not present
**chr19:29705402:CAAAAA>C**	C19orf12	1	0,008930192	0,0302548	c.-153-13_-153-9del	Unknown	Not present
**chr19:49595961:G>A**	PRR12	1	0,008779908	0,0129864	c.1626G>A	p.Ser542=	Present
**chr1:15933262:A>G**	SPEN	1	2,86291E-05	6,04266E-05	c.7022A>G	p.Lys2341Arg	Present
**chr19:50666540:G>A**	SHANK1	1	0,002533872	0,0102654	c.5420C>T	p.Pro1807Leu	Present
**chr21:31147008:A>AT**	TIAM1	1	4,77969E-06	3,02773E-05	c.3367-6dup	Unknown	Not present
**chr21:33797364:C>T**	ITSN1	1	5,12857E-05	0,000100341	c.1953-15C>T	Unknown	Present
**chr22:27798945:T>TTGCTGC**	MN1	1	0,003993368	0,00588235	c.1593_1598dup	p.Gln549_Gln550dup	Not present
**chr22:41150023:C>T**	EP300	1	4,77187E-06	8,56878E-06	c.2642C>T	p.Pro881Leu	Present
**chr18:33739673:A>G**	ASXL3	1	0	0	c.2269A>G	p.Asn757Asp	Present
**chr22:31846904:C>T**	DEPDC5	1	3,84133E-06	1,46964E-05	c.3092C>T	p.Pro1031Leu	Present
**chr1:109269247:G>A**	CELSR2	1	0,00022953	0,00144788	c.6769G>A	p.Gly2257Arg	Not present
**chr13:110719922:A>C**	ING1	1	6,38312E-05	0,000851343	c.830A>C	p.Tyr277Ser	Not present
**chr2:211424195:A>INS**	ERBB4	1	0	0	Unknown	Unknown	Not present
**chr19:46920082:G>A**	ARHGAP35	1	8,32778E-05	0,00107673	c.1407G>A	p.Glu469=	Not present
**chr5:150361209:A>G**	TCOF1	1	0,002962838	0,0308377	c.162A>G	p.Gln54=	Not present
**chr5:150376525:C>T**	TCOF1	1	0,002844479	0,0303701	c.2245C>T	p.Pro749Ser	Not present
**chr6:33443876:C>T**	SYNGAP1	1	0,001609215	0,0116405	c.3324C>T	p.Ser1108=	Present
**chr6:75907674:T>C**	MYO6	1	0,001360412	0,0151362	c.3246T>C	p.Tyr1082=	Not present
**chr6:107876670:CAAAA>C**	SEC63	1	0,005371438	0,0320827	c.1936-12_1936-9del	Unknown	Not present
**chr7:45575005:G>A**	ADCY1	1	5,78125E-05	0,00105173	c.462G>A	p.Gly154=	Present
**chr7:74689179:G>A**	GTF2I	1	0,000464944	0,00581677	c.51G>A	p.Ser17=	Present

**chr7:83134242:T>TATAA**	PCLO	1	9,26586E-05	0,000473485	c.3300+7_3300+8insTTAT	Unknown	Present
**chr7:98897818:G>A**	TRRAP	1	1,9212E-05	3,14192E-05	c.585G>A	p.Thr195=	Present
**chr7:135557955:C>T**	NUP205	1	0,006051799	0,0107705	c.11C>T	p.Pro4Leu	Not present
**chr7:151075514:T>C**	SLC4A2	1	1,31005E-05	0,000261883	c.3301+6T>C	Unknown	Not present
**chr8:105788814:G>C**	ZFPM2	1	0,00261882	0,00869054	c.629G>C	p.Ser210Thr	Not present
**chr9:89363849:G>A**	SEMA4D	1	0,000177971	0,00819672	c.1984C>T	p.Leu662Phe	Not present
**chr9:134818644:G>A**	COL5A1	1	0,003179576	0,0125057	c.4231-12G>A	Unknown	Not present
**chr9:134818983:C>T**	COL5A1	1	0,003039607	0,0118259	c.4393-17C>T	Unknown	Not present
**chr9:136496224:A>C**	NOTCH1	1	0,002874216	0,0297788	c.7515T>G	p.Pro2505=	Present
**chr9:136500807:G>A**	NOTCH1	1	0,003696945	0,0371943	c.5679C>T	p.Gly1893=	Present
**chr5:141485275:G>C**	PCDHGC4	1	4,77111E-06	3,02133E-05	c.102G>C	p.Pro34=	Not present
**chr5:138201415:G>A**	CDC23	1	2,17716E-05	9,06399E-05	c.449C>T	p.Ala150Val	Not present
**chr5:112839105:C>T**	APC	1	0,000364947	0,0024108	c.3511C>T	p.Arg1171Cys	Not present
**chr3:105868219:T>G**	CBLB	1	0,001775613	0,0128137	c.20A>C	p.Asn7Thr	Not present
**chr1:10095522:T>C**	UBE4B	1	1,28051E-05	0,000956023	c.273T>C	p.Asn91=	Not present
**chr1:11238574:G>A**	MTOR	1	0,001719467	0,0031682	c.1830C>T	p.Phe610=	Present
**chr1:39387318:T>C**	MACF1	1	0,002011544	0,0164171	c.14476T>C	p.Leu4826=	Present
**chr2:74362989:C>T**	DCTN1	1	0,00665493	0,00908497	c.3529+5G>A	Unknown	Not present
**chr2:188997207:C>A**	COL3A1	1	0,005242696	0,0217077	c.1804C>A	p.Pro602Thr	Not present
**chr2:207088517:G>A**	KLF7	1	0,003137221	0,0357401	c.596C>T	p.Ser199Leu	Present
**chr3:77563300:A>G**	ROBO2	1	3,18367E-06	0,000241896	c.1665A>G	p.Pro555=	Present
**chr4:48586692:A>G**	FRYL	1	0,00083846	0,00710227	c.1677T>C	p.Thr559=	Present
**chr5:87390789:C>T**	RASA1	1	1,28082E-05	3,02261E-05	c.3061-11C>T	Unknown	Not present
**chr4:76739340:T>A**	SHROOM3	1	0,000206258	0,00474383	c.1167T>A	p.Ser389Arg	Not present
**chr4:95129981:C>T**	BMPR1B	1	0,002624225	0,0291323	c.705C>T	p.Thr235=	Not present
**chr4:139370401:C>T**	NAA15	1	0,002434867	0,0283519	c.1944C>T	p.Ala648=	Present
**chr4:166057293:C>T**	TLL1	1	0,001474257	0,0180378	c.1830C>T	p.Asp610=	Not present
**chr5:56875262:G>A**	MAP3K1	1	0,002491607	0,0256584	c.1917G>A	p.Leu639=	Not present
**chr5:56882287:C>T**	MAP3K1	1	0,001070395	0,0128261	c.3087C>T	p.Phe1029=	Not present
**chr5:83519829:T>A**	VCAN	1	0,002737446	0,0049403	c.1523T>A	p.Ile508Asn	Not present

**chr17:63340097:G>T**	TANC2	1	1,15416E-05	0,00024108	c.1576-4G>T	Unknown	Present
**chr16:14140633:C>T**	MRTFB	1	0,003900098	0,00933258	c.27C>T	p.Thr9=	Present
**chr16:2102618:C>T**	PKD1	1	0,007820876	0,0206059	c.8964G>A	p.Ala2988=	Present
**chr15:87929287:G>A**	NTRK3	1	3,18076E-06	2,77239E-05	c.2037C>T	p.Asp679=	Present
**chr7:74601100:G>A**	GTF2IRD1	1	1,40876E-05	4,29984E-05	c.2686G>A	p.Val896Ile	Not present
**chr12:49040934:C>T**	KMT2D	1	0,000233175	0,000483092	c.6836G>A	p.Gly2279Glu	Not present
**chr21:31160494:A>G**	TIAM1	1	3,76353E-05	0,000649351	c.41T>C	p.Ile14Thr	Not present
**chr7:18590357:A>G**	HDAC9	1	0,00012188	0,00144928	c.286A>G	p.Ile96Val	Not present
**chr17:7846859:TACCACCACCACCACC>T**	KDM6B	1	8,81289E-05	0,00275157	c.777_791del	p.Pro260_Pro264del	Present
**chr5:150372090:C>T**	TCOF1	1	4,73738E-05	0,000434385	c.724C>T	p.Pro242Ser	Not present
**chr2:236580695:A>G**	ACKR3	1	3,20098E-05	0,000100292	c.230A>G	p.Tyr77Cys	Not present
**chr19:3977978:G>C**	EEF2	1	0,000665446	0,0100981	c.1908C>G	p.Ala636=	Not present
**chr16:28985377:G>T**	LAT	1	1,75696E-05	0,000091638	c.68G>T	p.Gly23Val	Not present
**chr20:47246409:T>G**	ZMYND8	1	0,00038921	0,00244867	c.1883A>C	p.Gln628Pro	Present
**chr9:136500810:C>T**	NOTCH1	1	0,000130562	0,000269397	c.5676G>A	p.Gly1892=	Present
**chr17:82083978:C>T**	FASN	1	0,000106001	0,000747492	c.5095G>A	p.Val1699Met	Not present
**chr22:50462625:A>G**	SBF1	1	1,02617E-05	6,54365E-05	c.2061T>C	p.Tyr687=	Present
**chr4:122620929:C>T**	IL21	1	5,25105E-05	0,000810296	c.83G>A	p.Ser28Asn	Not present
**chr1:6121545:C>A**	CHD5	1	0,002284563	0,00727342	c.4728G>T	p.Met1576Ile	Not present
**chr1:9721495:C>T**	PIK3CD	1	0,001246378	0,00217609	c.1863C>T	p.Tyr621=	Not present
**chr1:11212922:C>A**	MTOR	1	1,15333E-05	3,02407E-05	c.3286-14G>T	Unknown	Present
**chr9:98293870:C>T**	GABBR2	1	0,000134451	0,000653509	c.2575G>A	p.Asp859Asn	Present
**chr14:102005114:G>A**	DYNC1H1	1	4,48173E-05	0,000478011	c.5311G>A	p.Gly1771Arg	Present
**chr11:66700725:C>T**	SPTBN2	1	0,000105095	0,000339251	c.3374G>A	p.Arg1125Gln	Not present
**chr22:41127755:G>C**	EP300	1	0,008402156	0,0130391	c.1168+7G>C	Unknown	Present
**chr19:10987736:C>A**	SMARCA4	1	0,002303737	0,0273926	c.930C>A	p.Arg310=	Present
**chr19:14161397:G>A**	ADGRL1	1	0,002569674	0,0291624	c.1425C>T	p.Cys475=	Present
**chr19:49908325:G>A**	NUP62	1	0,001517332	0,0176156	c.1483C>T	p.Leu495=	Not present
**chr19:50688073:C>A**	SHANK1	1	0,00299527	0,0067535	c.2173-15G>T	Unknown	Present
**chr19:53904700:C>T**	PRKCG	1	0,0021428	0,0249802	c.1722C>T	p.Tyr574=	Not present
**chr19:54152924:C>T**	CNOT3	1	0,000360092	0,00288018	c.1962C>T	p.Pro654=	Present

**chr21:44082828:G>A**	TRAPPC10	1	0,001083507	0,00652489	c.1764G>A	p.Leu588=	Not present
**chr9:137744082:G>C**	EHMT1	1	4,77245E-06	8,56883E-06	c.1162G>C	p.Ala388Pro	Present
**chr15:77614704:A>T**	LINGO1	1	0,000106814	0,000207125	c.1203T>A	p.Phe401Leu	Not present
**chr3:49652571:C>G**	BSN	1	0	0	c.3015C>G	p.Asp1005Glu	Present
**chr19:31277631:G>A**	TSHZ3	1	0	0	c.2162C>T	p.Pro721Leu	Present
**chr5:150080910:G>A**	CSF1R	1	0	0	c.164C>T	p.Ser55Leu	Not present
**chr16:1766564:G>C**	MAPK8IP3	1	6,38097E-06	3,02444E-05	c.2855G>C	p.Arg952Pro	Present
**chr17:61982614:A>G**	MED13	1	2,561E-05	5,64525E-05	c.3389T>C	p.Met1130Thr	Present
**chr17:58005788:G>A**	SRSF1	1	1,66475E-05	0,000477555	c.565C>T	p.Arg189Cys	Present
**chr22:41523889:C>T**	ACO2	1	1,41135E-05	9,10194E-05	c.1430C>T	p.Thr477Met	Not present
**chr1:18631624:G>C**	PAX7	1	1,59388E-06	2,85796E-06	c.21G>C	p.Thr7=	Not present
**chr2:26934674:C>T**	DPYSL5	1	0,002789827	0,00856209	c.887C>T	p.Thr296Met	Not present
**chr2:36476946:G>A**	CRIM1	1	0,000670926	0,00109121	c.1049G>A	p.Arg350Gln	Not present
**chr10:110133375:C>T**	ADD3	1	0,000317509	0,000946074	c.1878C>T	p.Val626=	Not present
**chr6:142774545:C>A**	HIVEP2	1	0,002543907	0,0042632	c.194G>T	p.Gly65Val	Present
**chr7:45713918:G>A**	ADCY1	1	1,66568E-05	4,57247E-05	c.3283G>A	p.Val1095Ile	Present
**chr7:82914969:C>T**	PCLO	1	6,36604E-06	6,04961E-05	c.13017G>A	p.Pro4339=	Present
**chr9:128272809:C>T**	GOLGA2	1	8,82702E-06	7,24953E-05	c.264G>A	p.Ala88=	Not present
**chr9:132264633:A>G**	SETX	1	0,00431061	0,0121569	c.7640T>C	p.Ile2547Thr	Not present
**chr9:136516015:G>A**	NOTCH1	1	0,00870139	0,0181556	c.1635C>T	p.Asp545=	Present
**chr10:29493408:A>G**	SVIL	1	0,001628447	0,0164455	c.3842-17T>C	Unknown	Not present
**chr10:116065641:G>GA**	GFRA1	1	0,002824424	0,00484864	c.1198-16dup	Unknown	Not present
**chr6:89950862:C>T**	BACH2	1	0,002741087	0,0042282	c.1244G>A	p.Gly415Glu	Not present
**chr11:3771851:C>T**	NUP98	1	0,000582724	0,00556093	c.681G>A	p.Leu227=	Not present
**chr11:70798514:C>T**	SHANK2	1	0,008479176	0,0123949	c.1706G>A	p.Arg569His	Present
**chr12:111598975:CTGT>C**	ATXN2	1	0,002179641	0,013516	c.57_59del	p.Gln28del	Present
**chr12:131960979:C>T**	EP400	1	0,000710313	0,00483779	c.360C>T	p.Tyr120=	Present
**chr15:28182522:C>T**	HERC2	1	5,77611E-05	0,0015528	c.8826-10G>A	Unknown	Present
**chr15:33706969:G>A**	RYR3	1	3,18659E-06	5,72043E-06	c.6534G>A	p.Leu2178=	Not present
**chr15:71898076:G>C**	MYO9A	1	0,003949026	0,0271986	c.4427C>G	p.Pro1476Arg	Not present
**chr6:117703645:C>T**	NUS1	1	0,000103739	0,00201613	c.732C>T	p.Phe244=	Not present

**chr6:7246415:G>A**	RREB1	1	0,00495450 4	0,0238498	c.3974-9G>A	Unknown	Not present
**chr2:96297681:T>C**	SNRNP200	1	0,00373177 3	0,00614367	c.1159A>G	p.Met387Val	Not present
**chr2:240783086:C>T**	KIF1A	1	7,68549E-05	0,0008285	c.822G>A	p.Ser274=	Present
**chr2:108763701:G>T**	RANBP2	1	0,00326916 2	0,00564778	c.3162G>T	p.Gln1054His	Not present
**chr2:108763702:C>T**	RANBP2	1	0,00326903 7	0,00564726	c.3163C>T	p.Pro1055Ser	Not present
**chr2:148469973:G>A**	MBD5	1	0,00255109 9	0,00989382	c.2030G>A	p.Ser677Asn	Present
**chr2:154698894:A>G**	KCNJ3	1	0,00650000 8	0,0156756	c.119A>G	p.Lys40Arg	Not present
**chr2:189098775:G>A**	COL5A2	1	0,00741260 3	0,0394813	c.370-16C>T	Unknown	Not present
**chr2:189572975:G>A**	SLC40A1	1	4,74016E-05	9,14662E-05	c.272-14C>T	Unknown	Not present
**chr2:240745853:G>A**	KIF1A	1	0,00432466	0,00796332	c.3259C>T	p.Pro1087Ser	Present
**chr3:47849994:G>A**	DHX30	1	0	0	c.3459G>A	p.Leu1153=	Present
**chr5:83520371:A>G**	VCAN	1	0,00987194 2	0,0273933	c.2065A>G	p.Thr689Ala	Not present
**chr3:53183152:C>T**	PRKCD	1	0,00172449 1	0,00438446	c.603C>T	p.Ile201=	Not present
**chr3:101417673:T>C**	SENP7	1	0,00308337 7	0,0344985	c.402A>G	p.Ser134=	Not present
**chr3:105751591:T>C**	CBLB	1	0,00051990 2	0,0036162	c.594A>G	p.Arg198=	Not present
**chr3:132475009:C>A**	DNAJC13	1	0,00570062 5	0,012168	c.2369C>A	p.Ser790Tyr	Not present
**chr3:197194606:C>T**	DLG1	1	0,00076087 6	0,0150543	c.319-17G>A	Unknown	Present
**chr4:3074876:C>CCAGCAGCAGCAGCAGCAGCAGCAGCAGCAG**	HTT	1	0,00376219 4	0,00618941	c.81_110dup	p.Gln29_Gln38dup	Not present
**chr5:56878986:T>C**	MAP3K1	1	1,02479E-05	2,00038E-05	c.1972T>C	p.Leu658=	Not present
**chr9:136504680:C>T**	NOTCH1	1	0,00301339 7	0,0286153	c.5011G>A	p.Val1671Ile	Present
**chr9:136515524:C>T**	NOTCH1	1	0,00117100 8	0,00554484	c.1862G>A	p.Arg621His	Present
**chr9:136544100:G>A**	NOTCH1	1	0,00190232 6	0,0177558	c.64C>T	p.Pro22Ser	Present
**chr2:189179572:G>A**	COL5A2	1	0,0006568	0,00341691	c.33C>T	p.Leu11=	Not present
**chr7:74518164:G>T**	GTF2IRD1	1	0,00772093 2	0,0112587	c.447G>T	p.Val149=	Not present
**chr7:82066541:A>G**	CACNA2D1	1	0,00393750 4	0,0160604	c.659-17T>C	Unknown	Present
**chr7:103545304:C>T**	RELN	1	0,00221385 7	0,0258845	c.6343G>A	p.Gly2115Ser	Present
**chr7:103551177:G>A**	RELN	1	1,3134E-05	2,40581E-05	c.6192C>T	p.His2064=	Present
**chr7:103561959:C>CAAAAAAAAAAA**	RELN	1	0	0	c.5211-17_5211-7dup	Unknown	Present
**chr7:103565566:T>A**	RELN	1	0,00161596 1	0,0171758	c.4937-15A>T	Unknown	Present
**chr7:135600959:G>A**	NUP205	1	0,00291309 4	0,00544659	c.2364G>A	p.Val788=	Not present
**chr8:103885391:C>T**	RIMS2	1	0,00295423 8	0,0314169	c.924C>T	p.Thr308=	Present

**chr8:132129575:G>T**	KCNQ3	1	0,002627185	0,0213632	c.2306C>A	p.Pro769His	Present
**chr9:842060:C>A**	DMRT1	1	2,5284E-05	0,000280112	c.222C>A	p.Pro74=	Not present
**chr9:20413815:G>A**	MLLT3	1	0,003141215	0,028208	c.1031C>T	p.Thr344Ile	Not present
**chr9:128693631:C>G**	SET	1	2,59853E-06	3,06335E-05	c.493-7C>G	Unknown	Present
**chr10:21673528:G>C**	MLLT10	1	0,0002781	0,00124688	c.1230G>C	p.Gly410=	Not present
**chr10:29488708:C>G**	SVIL	1	0,001626083	0,0122324	c.4241G>C	p.Ser1414Thr	Not present
**chr10:60072347:C>T**	ANK3	1	0,001606238	0,0024855	c.8534G>A	p.Gly2845Glu	Present
**chr10:102398453:G>A**	NFKB2	1	0,000713119	0,00113396	c.921G>A	p.Leu307=	Not present
**chr10:102599540:G>T**	SUFU	1	0,006991413	0,0101761	c.1018G>T	p.Ala340Ser	Not present
**chr6:170561966:AGCAGCAGCAGCAGCAGCAGCAG>A**	TBP	1	0,002530698	0,0266138	c.231_252del	p.Gln77HisfsTer60	Not present
**chr6:148543929:G>A**	SASH1	1	0,001691304	0,00768272	c.2459G>A	p.Arg820Gln	Present
**chr6:146434425:C>G**	GRM1	1	0,002824461	0,016855	c.3214C>G	p.Pro1072Ala	Not present
**chr4:87090078:C>T**	AFF1	1	0,005552666	0,00817137	c.1191+8C>T	Unknown	Not present
**chr3:128069629:C>T**	SEC61A1	1	0,008848912	0,0135397	c.1398C>T	p.Ser466=	Not present
**chr3:129570950:G>A**	PLXND1	1	0,000116507	0,0024108	c.3601-15C>T	Unknown	Not present
**chr3:129584163:G>A**	PLXND1	1	0,000140474	0,00121832	c.2100C>T	p.Tyr700=	Not present
**chr3:129586219:G>A**	PLXND1	1	8,19163E-05	0,00105042	c.1674C>T	p.Cys558=	Not present
**chr3:196068142:C>A**	TFRC	1	0,001384892	0,0161309	c.802-12G>T	Unknown	Not present
**chr4:2884609:G>A**	ADD1	1	1,15398E-05	0,000192678	c.453G>A	p.Ala151=	Not present
**chr4:84794752:A>T**	WDFY3	1	0,001833954	0,0197422	c.3269-15T>A	Unknown	Present
**chr4:108167733:C>G**	LEF1	1	0,00013327	0,00137309	c.35G>C	p.Gly12Ala	Not present
**chr6:107883071:C>G**	SEC63	1	0	0	c.1750G>C	p.Asp584His	Not present
**chr4:125451863:C>T**	FAT4	1	0,004314286	0,00619172	c.10853C>T	p.Thr3618Met	Not present
**chr5:35065235:A>G**	PRLR	1	0	0	c.1723T>C	p.Ser575Pro	Not present
**chr5:131671533:C>A**	FNIP1	1	0,001034104	0,00620861	c.2911G>T	p.Ala971Ser	Not present
**chr5:131677716:A>G**	FNIP1	1	0,001026063	0,00621375	c.1506T>C	p.Leu502=	Not present
**chr5:136177321:C>T**	SMAD5	1	4,35709E-05	0,000151231	c.1255-16C>T	Unknown	Not present
**chr6:35227686:C>T**	SCUBE3	1	0,000345725	0,00146861	c.192C>T	p.Asp64=	Not present
**chr6:107634459:A>G**	SOBP	1	0,006568081	0,0284612	c.1615A>G	p.Ile539Val	Not present
**chr11:68363739:G>A**	LRP5	1	0,002296778	0,0231518	c.687-8G>A	Unknown	Not present
**chr11:68410117:G>A**	LRP5	1	0,000787726	0,00447503	c.2295G>A	p.Ser765=	Not present

**chr11:78812231:G>A**	TENM4	1	0,004319483	0,00693651	c.1869C>T	p.Cys623=	Not present
**chr18:27990163:T>C**	CDH2	1	0	0	c.1532A>G	p.His511Arg	Present
**chr20:6778459:C>T**	BMP2	1	0,00037514	0,00374424	c.561C>T	p.Pro187=	Not present
**chr20:51523446:G>A**	NFATC2	1	6,41334E-06	1,15003E-05	c.795C>T	p.Pro265=	Not present
**chr22:36292132:G>A**	MYH9	1	0,001179236	0,00195508	c.4198C>T	p.Arg1400Trp	Present
**chr22:36293875:G>A**	MYH9	1	0,004884489	0,0474034	c.3838-12C>T	Unknown	Present
**chr22:36306056:A>G**	MYH9	1	0,002953268	0,0267414	c.2038-5T>C	Unknown	Present
**chr22:36309402:G>A**	MYH9	1	0,002517202	0,0221805	c.1729-6C>T	Unknown	Present
**chr5:177259987:A>T**	NSD1	1	0	0	c.4967-2A>T	Unknown	Present
**chr2:229858830:C>A**	TRIP12	1	0	0	c.882G>T	p.Arg294Ser	Present
**chr19:41902839:C>T**	ARHGEF1	1	2,051E-05	0,00024108	c.1679C>T	p.Thr560Met	Not present
**chr12:111034221:T>C**	CUX2	1	4,77081E-06	9,10481E-06	c.44T>C	p.Phe15Ser	Present
**chr15:48534175:A>G**	FBN1	1	0	0	c.767T>C	p.Leu256Pro	Present
**chr2:54618118:G>A**	SPTBN1	1	3,32973E-05	0,000207469	c.688G>A	p.Ala230Thr	Present
**chr1:224114316:A>T**	FBXO28	1	0	0	c.187A>T	p.Thr63Ser	Not present
**chr18:45038540:A>INS**	SETBP1	1	0	0	Unknown	Unknown	Present
**chr11:16014936:A>G**	SOX6	1	0,000227076	0,00169328	c.1732+6T>C	Unknown	Present
**chr17:59685669:G>A**	CLTC	1	1,11338E-05	0,000114312	c.4688G>A	p.Arg1563Lys	Present
**chr19:50714240:C>T**	SHANK1	1	0,000731043	0,00692907	c.582G>A	p.Lys194=	Present
**chr19:18121763:G>A**	MAST3	1	0,000179285	0,00144718	c.240G>A	p.Ser80=	Present
**chr12:8922668:C>G**	PHC1	1	0,001373972	0,0149175	c.492C>G	p.Thr164=	Not present
**chr16:2097408:C>T**	PKD1	1	8,11003E-05	0,000413736	c.10316G>A	p.Arg3439Gln	Present
**chr12:50695254:C>T**	DIP2B	1	2,57437E-06	0,000477555	c.1720-13C>T	Unknown	Not present
**chr12:79449113:G>A**	SYT1	1	0,00252137	0,00864553	c.1258G>A	p.Val420Ile	Present
**chr12:114399544:C>A**	TBX5	1	0,003592756	0,0106647	c.331G>T	p.Asp111Tyr	Not present
**chr13:49732392:T>C**	KPNA3	1	0,009651741	0,0398338	c.362A>G	p.Lys121Arg	Not present
**chr14:24159851:C>T**	RNF31	1	0,002725505	0,0251022	c.2900-13C>T	Unknown	Not present
**chr14:24160348:G>T**	RNF31	1	0,002715638	0,0250481	c.3106G>T	p.Val1036Leu	Not present
**chr15:48411296:G>A**	FBN1	1	0,00021127	0,00168756	c.8310C>T	p.His2770=	Present
**chr16:2108375:T>G**	PKD1	1	0,000114494	0,000477555	c.6792A>C	p.Ser2264=	Present
**chr19:13207858:CCTGCTGCTG>C**	CACNA1A	1	0,00429934	0,0148026	c.6967_6975del	p.Gln2323_Gln2325del	Present
**chr16:3729114:T>C**	CREBBP	1	0,005056376	0,015	c.5933A>G	p.Asn1978Ser	Present
**chr16:15721419:T>C**	MYH11	1	0,001525015	0,00235183	c.4578+3A>G	Unknown	Not present
**chr17:7193071:G>A**	DLG4	1	0,004367976	0,00662824	c.1740C>T	p.Tyr580=	Present

**chr17:7509640:G>A**	POLR2A	1	0,003899304	0,0353372	c.3813+8G>A	Unknown	Present
**chr17:77492713:C>T**	SEPTIN9	1	0,000147253	0,00236518	c.1473C>T	p.Phe491=	Not present
**chr19:1207156:G>A**	STK11	1	3,18897E-06	5,72554E-06	c.243G>A	p.Lys81=	Not present
**chr19:13135414:G>A**	NACC1	1	0,001506468	0,00283554	c.207G>A	p.Leu69=	Present
**chr2:207768449:G>A**	FZD5	1	0	0	c.291C>T	p.Asp97=	Not present
**chr2:189053032:T>C**	COL5A2	1	0,007183256	0,0240203	c.2554-14A>G	Unknown	Not present
**chr9:137792116:T>G**	EHMT1	1	0,001354818	0,0119638	c.2474T>G	p.Leu825Trp	Present
**chr2:165365196:C>T**	SCN2A	1	0,00110923	0,00964785	c.3453C>T	p.Pro1151=	Present
**chr14:21368447:G>C**	SUPT16H	1	0,005388913	0,032068	c.783-6C>G	Unknown	Present
**chr14:64767708:C>T**	SPTB	1	0,001044686	0,0226834	c.6174G>A	p.Thr2058=	Not present
**chr14:101983230:A>G**	DYNC1H1	1	0,000325167	0,00111053	c.1173A>G	p.Gln391=	Present
**chr14:105155880:G>A**	JAG2	1	2,95268E-05	0,00024108	c.585C>T	p.Arg195=	Not present
**chr15:41698936:A>G**	MGA	1	0,000537651	0,00189215	c.2087A>G	p.Asp696Gly	Not present
**chr15:41742785:A>G**	MGA	1	0,000103732	0,00142045	c.4972A>G	p.Thr1658Ala	Not present
**chr15:41754532:G>A**	MGA	1	0,000538151	0,00190005	c.7251G>A	p.Lys2417=	Not present
**chr15:52364711:A>T**	MYO5A	1	0	0	c.3161-9T>A	Unknown	Present
**chr15:67184845:C>T**	SMAD3	1	7,82129E-05	0,00030303	c.990C>T	p.Thr330=	Not present
**chr15:77614797:C>T**	LINGO1	1	0,009089505	0,018732	c.1110G>A	p.Pro370=	Not present
**chr15:98922135:C>A**	IGF1R	1	0,000129314	0,000723589	c.2202-13C>A	Unknown	Not present
**chr16:15708848:CAGAG>C**	MYH11	1	0,000426194	0,00117755	c.5808-11_5808-8del	Unknown	Not present
**chr16:17198265:G>A**	XYLT1	1	1,40858E-05	5,54477E-05	c.1236C>T	p.Thr412=	Not present
**chr16:81946172:C>T**	PLCG2	1	0,002575893	0,0303264	c.3482-3C>T	Unknown	Not present
**chr16:81956850:C>A**	PLCG2	1	0,002551295	0,0300077	c.3726C>A	p.Leu1242=	Not present
**chr17:50568985:AGTGTGT>A**	CACNA1G	1	0,000610452	0,00167038	c.354+29_354+34del	Unknown	Present
**chr19:2217755:C>T**	DOT1L	1	0,001327295	0,00678998	c.2545-17C>T	Unknown	Present
**chr13:110211917:C>T**	COL4A1	1	0,007163232	0,0209196	c.393G>A	p.Glu131=	Not present
**chr13:35649646:A>G**	NBEA	1	0,003570879	0,0422464	c.7771-9A>G	Unknown	Present
**chr12:122323001:C>T**	CLIP1	1	0,00065678	0,00236518	c.4492G>A	p.Glu1498Lys	Not present
**chr11:115504383:T>C**	CADM1	1	0,000906427	0,0089786	c.12A>G	p.Val4=	Present
**chr10:3781714:C>T**	KLF6	1	0,008020343	0,0336158	c.603G>A	p.Arg201=	Not present
**chr10:29532858:C>T**	SVIL	1	0,003160189	0,0362905	c.1509G>A	p.Pro503=	Not present

**chr10:29550713:G>A**	SVIL	1	0,002497887	0,0295731	c.711C>T	p.Ser237=	Not present
**chr10:73769654:G>A**	SEC24C	1	0,000563392	0,0123919	c.2603G>A	p.Arg868His	Not present
**chr11:46542541:C>T**	AMBRA1	1	0,003933212	0,0426181	c.1476G>A	p.Ser492=	Not present
**chr11:78658435:C>T**	TENM4	1	0,003039206	0,0354477	c.7933G>A	p.Val2645Ile	Not present
**chr11:78738538:G>A**	TENM4	1	0,008153865	0,0122873	c.2789C>T	p.Thr930Ile	Not present
**chr12:292952:T>A**	KDM5A	1	0,002594637	0,0163851	c.4673A>T	p.Glu1558Val	Present
**chr12:122322558:C>G**	CLIP1	1	0,000656495	0,00236967	c.4935G>C	p.Glu1645Asp	Not present
**chr12:754249:C>T**	WNK1	1	0,002713284	0,022172	c.684C>T	p.Gly228=	Not present
**chr12:32733738:A>G**	DNM1L	1	0,00902448	0,0127599	c.1470A>G	p.Glu490=	Not present
**chr12:78137211:C>T**	NAV3	1	0,009519497	0,0135187	c.4476C>T	p.Pro1492=	Present
**chr12:108526396:G>A**	SART3	1	0,005382925	0,0218324	c.2073C>T	p.Asp691=	Not present
**chr12:110346405:A>G**	ATP2A2	1	0,000725871	0,00372517	c.3064A>G	p.Ile1022Val	Not present
**chr12:117330734:T>C**	NOS1	1	0,000105011	0,000946074	c.336A>G	p.Thr112=	Not present
**chr12:118036361:G>A**	WSB2	1	0,000334201	0,000815907	c.810C>T	p.Thr270=	Not present
**chr19:14097684:C>A**	PRKACA	1	1,27954E-05	6,06722E-05	c.547-10G>T	Unknown	Not present
**chr19:18860385:C>T**	UPF1	1	0,009980513	0,014977	c.2247C>T	p.Tyr749=	Not present
**chr19:53906860:A>G**	PRKCG	1	0,002169306	0,0246664	c.2059A>G	p.Ser687Gly	Not present
**chr1:23093950:C>A**	LUZP1	1	0,00070416	0,00444793	c.312G>T	p.Arg104=	Not present
**chr10:29532859:G>A**	SVIL	1	0,000300885	0,00189573	c.1508C>T	p.Pro503Leu	Not present
**chr11:7995288:G>A**	EIF3F	1	1,28044E-05	0,000113033	c.917G>A	p.Arg306His	Present
**chr16:72795569:G>A**	ZFHX3	1	2,56152E-05	0,00072324	c.7113C>T	p.Ala2371=	Present
**chr17:8476980:C>T**	MYH10	1	0,000253495	0,00236295	c.5775G>A	p.Thr1925=	Present
**chr12:49051420:G>A**	KMT2D	1	2,06252E-05	0,000211775	c.2263C>T	p.Arg755Trp	Not present
**chr16:72797921:G>C**	ZFHX3	1	0,000259935	0,0024108	c.4761C>G	p.Pro1587=	Present
**chr1:23091388:T>A**	LUZP1	1	0,000873114	0,0048604	c.2874A>T	p.Thr958=	Not present
**chr1:109268900:G>A**	CELSR2	1	0,004572928	0,0327629	c.6523G>A	p.Asp2175Asn	Not present
**chr16:30966149:C>T**	SETD1A	1	0,000261538	0,0016357	c.2268C>T	p.Ala756=	Present
**chr1:116666316:A>G**	IGSF3	1	1,11327E-05	0,00024108	c.11T>C	p.Phe4Ser	Not present
**chr1:154171434:A>C**	TPM3	1	1,59046E-06	3,02151E-05	c.621T>G	p.Ser207=	Not present
**chr1:165205979:C>A**	LMX1A	1	5,04189E-05	0,000331518	c.873G>T	p.Thr291=	Not present

**chr1:186332304:C>T**	TPR	1	0,003694667	0,00651859	c.5495G>A	p.Arg1832His	Not present
**chr2:55346187:C>T**	CCDC88A	1	0,001238993	0,0104071	c.1029G>A	p.Lys343=	Not present
**chr2:55346339:C>T**	CCDC88A	1	0,001228652	0,0103234	c.883-6G>A	Unknown	Not present
**chr2:108771878:CTT>C**	RANBP2	1	6,14847E-05	0,000523766	c.8020+10_8020+11del	Unknown	Not present
**chr6:7558156:G>A**	DSP	1	3,96883E-05	0,000277254	c.314G>A	p.Arg105Gln	Not present
**chr16:72794085:T>C**	ZFHX3	1	3,07314E-05	0,00072324	c.8597A>G	p.Lys2866Arg	Present
**chr20:32795432:G>A**	DNMT3B	1	0,00976398	0,0169347	c.1150G>A	p.Ala384Thr	Not present
**chr7:99011234:C>T**	TRRAP	1	6,40231E-06	0,000478469	c.11121C>T	p.Pro3707=	Present
**chr20:36190742:C>T**	EPB41L1	1	0,001645099	0,0154291	c.1245C>T	p.Pro415=	Not present
**chr20:44428408:C>T**	HNF4A	1	0,004168555	0,00650916	c.1137C>T	p.Asn379=	Not present
**chr21:33550283:C>G**	SON	1	2,94581E-05	0,000339098	c.1052C>G	p.Ala351Gly	Present
**chr21:44103528:A>C**	TRAPPC10	1	0	0	c.3777A>C	p.Thr1259=	Not present
**chr4:3211942:G>A**	HTT	1	0	0	c.6428G>A	p.Ser2143Asn	Not present
**chr6:170288403:G>A**	DLL1	1	1,27273E-05	3,02133E-05	c.506C>T	p.Thr169Met	Present
**chr7:98965881:A>G**	TRRAP	1	3,45678E-05	0,000207297	c.7162A>G	p.Met2388Val	Present
**chr12:6602502:A>T**	CHD4	1	0,000483356	0,00189573	c.101-5T>A	Unknown	Present
**chr15:52353896:T>C**	MYO5A	1	9,54347E-06	0,000242365	c.3542A>G	p.Glu1181Gly	Present
**chr9:128576886:C>T**	SPTAN1	1	2,30507E-05	0,000228634	c.715C>T	p.Arg239Trp	Not present
**chr16:14246939:T>C**	MRTFB	1	7,95322E-06	0,00024108	c.1679T>C	p.Leu560Pro	Present
**chr12:132062553:AGCAG>A**	EP400	1	0,000236334	0,00292914	c.8187_8190del	p.Gln2729HisfsTer47	Present
**chr5:132934732:T>C**	AFF4	1	1,59039E-06	1,43271E-05	c.333A>G	p.Val111=	Not present
**chr10:79292257:G>A**	ZMIZ1	1	3,72323E-05	0,000192901	c.858G>A	p.Ala286=	Present
**chr14:102049722:C>T**	DYNC1H1	1	7,68427E-06	6,53509E-05	c.13524C>T	p.His4508=	Present
**chr5:98869788:AGAG>A**	CHD1	1	5,50758E-05	0,000242954	c.4070_4072del	p.Pro1357del	Present
**chr22:49775698:G>C**	BRD1	1	0,000491904	0,00566129	c.3279C>G	p.Val1093=	Not present
